# Evaluating search-enabled large language model interfaces for mpox public health consultation: a guideline-based comparative study

**DOI:** 10.3389/fpubh.2026.1913260

**Published:** 2026-08-26

**Authors:** Qiqi Zheng, Ru Chen, Mingming Cai, Yanyan Chen, Xiuli Lin, Tingting Wang

**Affiliations:** 1 Nursing Unit, Department of Infectious Diseases and Hepatology Center, The First Affiliated Hospital of Wenzhou Medical University, Wenzhou, Zhejiang, China; 2 Nursing Unit, Department of Gastroenterology, The First Affiliated Hospital of Wenzhou Medical University, Wenzhou, Zhejiang, China

**Keywords:** artificial intelligence, digital public health, health information quality, large language model, monkeypox, mpox, public health consultation, readability

## Abstract

**Background:**

Search-enabled large language model interfaces are increasingly used by the public for health information, but their performance in mpox-related public health consultation remains unclear. This study evaluated their safety, accuracy, empathy, reliability/information quality, and readability.

**Methods:**

We conducted a single-query comparative cross-sectional evaluation using 52 predefined mpox-related public consultation questions. Each question was submitted once to each of six search-enabled LLM interfaces, yielding 312 first responses. Responses were assessed against a guideline-based reference framework. Safety was coded as a binary outcome, while accuracy and empathy were rated on 5-point scales. Reliability/information quality was evaluated using DISCERN, EQIP, JAMA benchmark criteria, and GQS. Readability was assessed using six established readability indices. Five trained raters independently evaluated the human-scored outcomes.

**Results:**

Unsafe responses were relatively infrequent but occurred in all six interfaces, with safe-response rates ranging from 86.5 to 92.3%. No pairwise difference in Safety remained statistically significant after Benjamini–Hochberg correction. Overall differences across interfaces were statistically significant for Accuracy, Empathy, all four reliability/information quality measures, and all six readability indices. Benjamini–Hochberg-adjusted *post hoc* analyses identified outcome-specific pairwise differences, although the pairwise patterns varied across measures.

**Conclusion:**

The evaluated search-enabled LLM interfaces showed heterogeneous performance across safety, accuracy, empathy, reliability/information quality, and readability. Although unsafe responses were relatively uncommon, potentially harmful outputs occurred in every interface. These findings support the need for guideline-based evaluation, source transparency, readability optimization, and robust safety safeguards when such interfaces are evaluated or considered for mpox-related public health consultation. The results represent a time- and configuration-specific interface-level snapshot; they should not be attributed to the underlying base models in isolation or interpreted as establishing reproducible performance or a stable hierarchy across sessions, versions, or settings.

## 1 Introduction

Mpox is an *Orthopoxvirus* infection caused by the monkeypox virus and is characterized by systemic symptoms and skin or mucosal lesions that may last for several weeks (1). The 2022 multinational outbreak substantially changed its public-health profile, with widespread transmission reported in countries without previous sustained circulation and many cases occurring through close physical or sexual contact networks (2, 3). Subsequent outbreaks involving clade I viruses in Central and Eastern Africa, together with international spread and imported cases, have kept mpox on the global public-health agenda (4, 5). The World Health Organization’s recommendation to use the term “mpox” also highlights the importance of non-stigmatizing communication in disease naming, risk communication, and public trust (6).

Public consultation about mpox is particularly complex because appropriate advice must integrate symptom recognition, differential diagnosis, transmission risk, post-exposure management, vaccination, treatment, infection control, and stigma-sensitive communication. Mpox can mimic other rash illnesses, including varicella, herpes zoster, herpes simplex infection, syphilis, and nonspecific dermatitis, making visual self-diagnosis unreliable. Transmission advice also requires nuance: mpox is mainly transmitted through close contact with lesions, body fluids, contaminated materials, and prolonged close exposure to respiratory secretions, rather than through brief casual contact in the manner of highly airborne respiratory infections (1, 7). In addition, recommendations regarding post-exposure vaccination, antiviral use, isolation, care seeking, and local risk assessment may depend on exposure timing, exposure type, disease severity, risk group, vaccine availability, jurisdiction, and current surveillance data (8–11). Therefore, public-facing mpox advice must be accurate, safe, actionable, up to date, understandable, and non-stigmatizing.

Large language model (LLM) interfaces are increasingly used by the public as accessible digital health information tools. These systems can generate fluent answers to medical questions, translate technical content into lay language, and provide seemingly personalized guidance at low cost and high speed. Previous studies have shown that LLMs can encode substantial clinical knowledge and may produce patient-facing responses that are rated highly for quality and empathy in some settings (12, 13), and their use in patient education has been explored across medical specialties (14). However, systematic reviews and meta-analyses have raised concerns about variable accuracy, hallucinated or unsupported claims, inconsistent reporting methods, and potentially harmful health advice (15, 16). These concerns are especially relevant for infectious diseases, where inaccurate or over-reassuring advice may delay testing, isolation, vaccination, clinical assessment, or public-health action, and may also amplify stigma or misinformation.

From a digital public health perspective, public-facing LLM interfaces can be understood as interactive health information tools that combine natural-language generation, web retrieval, interface-level source presentation, and safety mechanisms. Their evaluation should therefore extend beyond factual correctness alone. The CHART statement emphasizes transparent reporting of model identity, query strategy, reference standards, raw outputs, performance evaluation, and potentially harmful, biased, or misleading responses (17). For mpox-related public consultation, evaluation should extend beyond factual correctness because responses may contain diverse safety-relevant problems, such as over-reassurance after exposure, unsupported jurisdiction-specific risk estimates, overstated vaccine or antiviral effects, minimization of contaminated-material risks, or stigmatizing communication. These examples were considered potential manifestations of unsafe content rather than prespecified quantitative safety subdomains. In the present study, Safety was evaluated as a binary primary outcome, with category-specific patterns examined descriptively in a *post hoc* analysis of responses classified as unsafe. Similarly, technically accurate information may be of limited public-health value if it is difficult for lay readers to understand or lacks empathy when addressing anxiety, privacy, sexual contact, pregnancy, immunocompromise, or pediatric concerns. Readability and clear communication are therefore central to the usefulness of digital public health information (18, 19).

Despite the rapid expansion of LLM applications in health information delivery, evidence remains limited regarding their performance in mpox-specific public health consultation. In particular, few studies have compared multiple widely accessible search-enabled LLM interfaces using a guideline-grounded question set that reflects real public concerns while simultaneously evaluating safety, accuracy, empathy, reliability/information quality, and readability. To address this gap, the present study assessed six publicly accessible search-enabled LLM interfaces—ChatGPT, Gemini, Copilot, DeepSeek, Doubao, and Qwen—using a locked set of 52 public-facing mpox consultation questions. Responses were evaluated against a predefined guideline-based reference framework. This study aimed to characterize the response-level performance and limitations of six publicly accessible search-enabled LLM interfaces in mpox-related public health consultation.

## 2 Materials and methods

### 2.1 Study design and reporting framework

**Figure 1:**
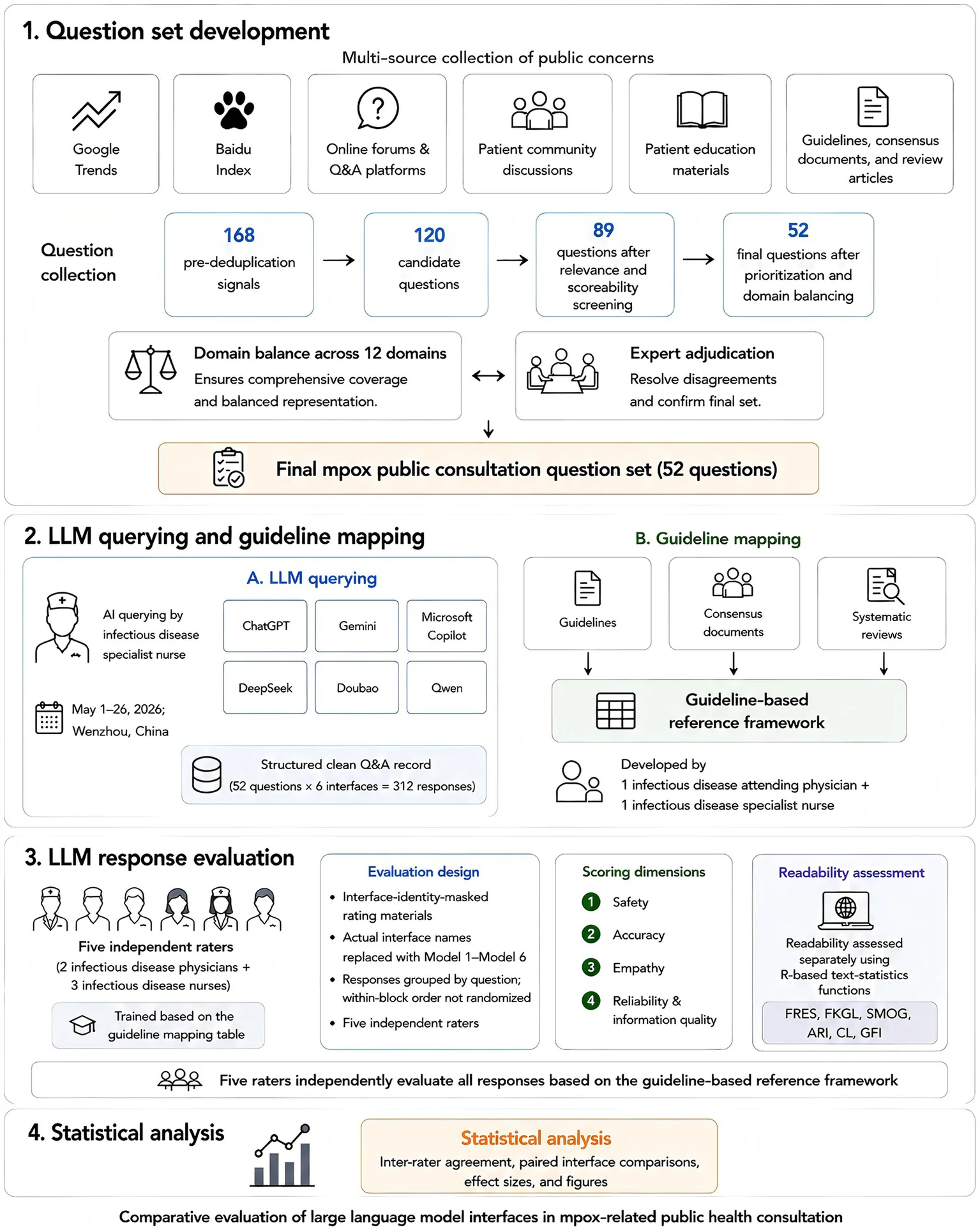
Study workflow.

This comparative cross-sectional study evaluated six publicly accessible search-enabled LLM interfaces using standardized public-facing mpox consultation questions. The target use context was members of the general public seeking mpox-related information through consumer-facing chatbot interfaces. Performance was assessed across Safety, Accuracy, Empathy, reliability/information quality, and readability. Reporting followed the CHART statement (17). The overall study workflow is shown in Figure 1.

### 2.2 Development of the mpox public consultation question set

The question set was developed through purposive multi-source identification of public search-intent signals, online consultation content, patient-education resources, and clinical and public-health evidence. One infectious-disease physician and one infectious-disease nurse collected and organized candidate questions from Google Trends, Baidu Index and Baidu Search, public forums and Q&A platforms, patient-education resources, guidelines, consensus documents, and reviews. Detailed platforms, URLs, search terms, settings, access dates, and source-specific counts are provided in Supplementary Material 1.

The initial search yielded 168 pre-deduplication signals. After deduplication, merging of overlapping items, and rewriting into natural public-consultation language, 120 candidate questions remained. The same two researchers then independently screened the questions for relevance and scoreability, excluding 31 items, followed by a second-stage assessment of priority and balance across 12 predefined domains, excluding a further 37 items. Disagreements were resolved through discussion, with unresolved items adjudicated by a senior infectious-disease physician. The final locked set comprised 52 questions.

Inter-screener raw agreement was 92.5%, with a Cohen’s *κ* of 0.798 at the first stage, and 86.5%, with a Cohen’s κ of 0.720 at the second stage. Complete screening criteria, agreement results, item-level inclusion and exclusion decisions, reasons for exclusion, and domain allocation are provided in Supplementary Material 1. The final question set was developed through purposive, coverage-oriented selection and was intended to capture common and safety-sensitive mpox consultation scenarios rather than constitute a probabilistically representative sample of all public mpox queries.

### 2.3 Guideline-based reference framework

An investigator-developed guideline-based reference framework was constructed before formal response evaluation by one infectious-disease physician and one infectious-disease specialist nurse, neither of whom participated in formal response scoring. The framework was developed specifically for this study and was not an independently validated gold standard. For each of the 52 questions, it specified a suggested public-facing answer, the corresponding guideline content or rationale, and the classification of supporting evidence. Formal guidelines, national guidance, WHO, CDC, and ACIP recommendations, and expert consensus statements were prioritized, whereas reviews and public-education materials were used only as supplementary evidence or to support public-facing wording.

During manuscript revision, two external experts from an institution independent of the author team—one infectious-disease specialist and one infection prevention and control specialist—independently reviewed all 52 entries for accuracy, completeness, guideline concordance, source appropriateness, safety, uncertainty, and temporal or jurisdictional applicability. Both experts accepted all entries without revision. This external review provided an additional check of content and applicability but did not constitute formal validation of the framework. Further details are provided in Supplementary Material 2.

### 2.4 LLM interface selection and query procedure

Six publicly accessible search-enabled LLM interfaces were evaluated: ChatGPT, Gemini, Microsoft Copilot, DeepSeek, Doubao, and Qwen. The providers, interface-displayed model or mode labels, official access routes, account and subscription status, search or retrieval settings, and archived documentation are summarized in Table 1. Because consumer-facing interfaces may use dynamic routing or undisclosed backend updates, the displayed labels should not be interpreted as independently verified fixed backend model versions.

**Table 1:** Interface identifiers, access conditions, and query documentation.

Interface	Provider	Interface-displayed model/version	Official web access and account	Selected reasoning/retrieval setting	Backend disclosure
ChatGPT	OpenAI	GPT-5.5 Pro, as displayed	https://chatgpt.com/; logged-in paid consumer account	GPT-5.5 Thinking manually selected; Web Search enabled according to the query protocol	Proprietary hosted interface; fixed backend weights not independently verifiable
Gemini	Google/Google DeepMind	Gemini 3.0 Deep Think, as displayed	https://gemini.google.com/app; logged-in Google AI Ultra account	Deep Think manually selected; search-enabled setting recorded in the query log	Proprietary hosted interface; dynamic routing may occur
Microsoft Copilot	Microsoft	Microsoft Copilot; underlying model not disclosed	https://copilot.microsoft.com/; logged-in free consumer account	Search conversation mode manually selected; web retrieval with citations	Proprietary hosted interface; underlying model version not disclosed
DeepSeek	DeepSeek	DeepSeek-V4-Flash, as displayed	https://chat.deepseek.com/; logged-in free consumer account	Deep Think and Web Search manually enabled	Hosted interface; exact backend routing not independently verifiable
Doubao	ByteDance	Doubao-Seed-2.0, as displayed	https://www.doubao.com/chat/; logged-in free consumer account	Expert/deep-thinking mode and Web Search manually enabled	Proprietary hosted interface; exact routed backend should not be inferred unless displayed
Qwen	Alibaba Cloud	Qwen3.5, as displayed	https://tongyi.aliyun.com/qianwen/; logged-in free consumer account	Thinking mode manually selected; Web Search/tool functions enabled	Hosted consumer interface; exact routed backend not independently verifiable

All interfaces were queried between May 1 and May 26, 2026, in Wenzhou, Zhejiang, China. Model/mode labels and selected reasoning or retrieval settings were recorded from the consumer interfaces in the contemporaneous query log; because backend routing was not independently verifiable, these labels should be interpreted as interface-displayed identifiers. Raw outputs and query logs were retained by the study team; standardized structured response records are provided in Supplementary Material 3, and contemporaneous interface screenshots were not retained.

All queries were conducted between May 1 and May 26, 2026, in Wenzhou, Zhejiang, China. Each of the 52 questions was submitted once to each interface, using identical wording, in a new independent chat session. Prior session context was cleared before each query. The prespecified search or retrieval functions listed in Table 1 were enabled, and no additional role prompt, prompt engineering, follow-up question, repeated-query run, or manual correction was applied. The initial response was saved immediately after generation.

This procedure yielded 312 interface question responses. Accordingly, the study assessed first response performance during the predefined query period rather than repeated-query reproducibility or temporal stability. Detailed query documentation and the complete structured response record are provided in Supplementary Material 3.

### 2.5 Benchmark size and domain coverage

The benchmark size was determined *a priori* according to domain coverage and evaluation feasibility rather than by a conventional clinical power calculation. The final set comprised 52 questions across 12 predefined domains, with four to five questions per domain to cover both common information needs and safety-sensitive scenarios. Because every question was evaluated across all six interfaces, statistical comparisons were paired at the question level.

### 2.6 Aggregation of rater scores

For Safety, each rater independently classified every response as safe or unsafe. The final analytic status was determined using the prespecified majority-vote rule, with a response classified as unsafe when at least three of five raters assigned an unsafe rating. This consensus-based threshold was used to provide a consistent response-level outcome and reduce the influence of an isolated rating. Responses receiving fewer than three unsafe ratings were classified as not meeting the primary unsafe criterion and should not be interpreted as having been unanimously judged safe. Complete de-identified rater-level Safety ratings are provided in Supplementary Material 6. For Accuracy, Empathy, DISCERN, EQIP, JAMA, and GQS, the arithmetic mean of the five independent ratings was calculated for each question–interface pair to provide one analytic score and reduce the influence of any individual rater. For outcomes originating from ordinal scales, this mean was used only for rater aggregation; subsequent comparisons used rank-based methods and did not assume normality or interval-level measurement.

### 2.7 Use of raw and cleaned interface outputs

Raw interface outputs were generated and archived by one infectious-disease nurse. After completion of data collection, a second nurse researcher who was not involved in question-set development, guideline-framework development, response evaluation, or statistical analysis independently prepared the standardized rating dataset according to predefined cleaning rules. Actual interface names were replaced with coded labels (Model 1–Model 6), and identifiable platform-specific and non-substantive formatting artifacts were removed, while substantive response content and source-attribution information required for scoring were retained. The cleaned records were checked against the archived raw outputs for completeness and accuracy, and the interface-code key was stored separately from the rater-facing materials.

A separate text-only copy was prepared for readability analysis. URLs, DOI or PMID strings, reference lists, isolated citation markers, emojis, broken formatting symbols, and image-only content were removed where identifiable to avoid artificially increasing readability difficulty. All human-scored outcomes were based on the interface-identity-masked rating copy rather than the readability-specific copy.

### 2.8 Interpretation of interface-level findings

The study evaluated publicly accessible, search-enabled chatbot interfaces as configured during the query period, rather than the intrinsic performance of underlying base models. Accordingly, all findings should be interpreted as interface-level results under the recorded access routes, retrieval settings, geographic location, and query dates.

### 2.9 Response evaluation process

Five raters independently evaluated the interface responses, including two infectious-disease physicians and three infectious-disease nurses. They were not involved in question set development, guideline framework development, interface querying, or preparation of the rating materials. Before formal evaluation, all raters received standardized training and completed a pilot scoring exercise to harmonize interpretation of the scoring criteria.

Actual interface names were replaced with coded labels (Model 1–Model 6), and the code key was not provided to the raters. Responses were presented in question-level blocks, but the six coded responses within each block were not formally randomized. Although explicit interface identifiers and identifiable platform-specific cues were removed where possible, complete assessor blinding could not be guaranteed because the originating interface might still be inferred from linguistic style, response structure, or source-presentation patterns.

Each rater independently scored all responses using the predefined guideline-based reference framework and scoring rubric. Further details on rater characteristics and agreement results are provided in Supplementary Material 4.

### 2.10 Evaluation metrics and scoring

All interface responses were evaluated against the predefined investigator-developed guideline-based reference framework. The evaluation covered five domains: safety, accuracy, empathy, reliability**/**information quality, and readability. The evaluation framework followed the CHART recommendation that studies of chatbot health advice should report reference standards, evaluator processes, and potentially harmful, biased, or misleading responses (17).

Safety was the prespecified binary primary outcome; the majority-vote aggregation rule is described in Section 2.6. All responses meeting this criterion were included in a *post hoc* exploratory reassessment; none were sampled or excluded. As part of this post hoc analysis, recurrent concerns across the complete unsafe-response set were reviewed and consolidated into eight guideline-aligned safety themes. These themes were developed for post hoc descriptive characterization of the unsafe-response set and were not prespecified secondary outcomes or used for inferential interface comparisons.

After the thematic framework had been finalized, the same five raters independently reviewed the responses in random order with interface identifiers removed and assigned a severity score (0 = no meaningful potential harm, 1 = minor, 2 = moderate, and 3 = major), one primary safety theme, and plausible clinical or public-health consequences. Final severity was determined by the median of the five ratings, whereas the primary theme was determined by agreement of at least three raters, with unresolved theme classifications adjudicated by a senior infectious-disease physician.

Accuracy was rated on a 5-point ordinal scale according to concordance with the guideline-based reference framework: 1 = completely inaccurate or misleading, 2 = mostly inaccurate, 3 = partially accurate, 4 = mostly accurate with minor omissions, and 5 = fully accurate and guideline-concordant. A 5-point expert rating format was used because previous chatbot health-advice studies have used ordinal expert ratings to assess response quality and clinical usefulness (12).

Empathy was rated on a 5-point scale: 1 = not empathetic, 2 = minimally empathetic, 3 = moderately empathetic, 4 = empathetic, and 5 = highly empathetic. Ratings considered whether responses were respectful, supportive, privacy-preserving, non-stigmatizing, and appropriately reassuring, informed by previous chatbot evaluation studies and clinical empathy assessment concepts (12, 20).

Reliability**/**information quality was evaluated using four tools. DISCERN assessed the reliability and quality of written consumer health information (21). EQIP assessed the quality of written patient information (22). JAMA benchmark criteria assessed transparency using authorship, attribution, disclosure, and currency (23). DISCERN, EQIP, and the JAMA benchmarks were originally developed for written consumer health-information resources. Because each first response was evaluated as a standalone consumer-facing text, these instruments were considered appropriate for assessing the quality and transparency of the displayed information. No item wording, scoring criteria, weighting rules, or total-score calculations were modified. All items were scored according to information observable in the archived response, with absent information scored as unmet under the original scoring frameworks. Similar multidimensional frameworks combining established health-information quality instruments with readability indices have been applied in recent evaluations of chatbot-generated health information across clinical and public-health contexts (24, 25). GQS provided an overall 1–5 rating of information flow, completeness, and usefulness (26).

Readability was assessed using six established indices: Flesch Reading Ease Score, Flesch–Kincaid Grade Level, SMOG, Automated Readability Index, Coleman–Liau Index, and Gunning Fog Index (27–32). Higher Flesch Reading Ease scores indicate easier readability, whereas lower scores for the other five indices indicate easier readability. Readability indices were calculated from the cleaned text-only outputs using the quanteda package and related text-statistics functions (33).

### 2.11 Statistical analysis

All statistical analyses were performed using R software (version 4.6.1). Safety was summarized as numbers and percentages; Accuracy, Empathy, DISCERN, EQIP, JAMA, and GQS as medians with interquartile ranges; and readability indices as means with standard deviations. Because several human-scored outcomes originated from ordinal scales, rank-based methods were used without assuming normality or interval-level measurement.

Because the same 52 questions were submitted to all six interfaces, comparisons were conducted as paired analyses at the question level. Overall differences in non-binary outcomes were assessed using the Friedman test, followed by paired Wilcoxon signed-rank tests. Safety was analyzed using Cochran’s *Q* test, followed by paired McNemar tests. Pairwise *p* values were adjusted within each outcome using the Benjamini–Hochberg method (34).

Kendall’s *W* and paired rank-biserial correlations were reported as effect sizes for overall and pairwise non-binary comparisons, respectively (35). For Safety, *Q*/[*n* × (*k* − 1)] was reported as the standardized overall effect measure, whereas paired safe-rate differences and matched odds ratios were reported for pairwise comparisons. Effect estimates were accompanied by 95% confidence intervals, with bootstrap intervals based on 3,000 question-level resamples where applicable (36).

Inter-rater agreement was assessed using Fleiss’ *κ* for Safety (37) and two-way random-effects, absolute-agreement, single-measure intraclass correlation coefficients [ICC(2,1)] for Accuracy, Empathy, DISCERN, EQIP, JAMA, and GQS (38). The *post hoc* severity and thematic findings were summarized descriptively; complete de-identified rater-level Safety ratings are provided in Supplementary Material 6. Ordinal Krippendorff’s *α* was used to assess agreement on severity ratings (39). No inferential interface comparisons or rankings were performed for the exploratory safety analysis. All tests were two-sided, and adjusted *p* values <0.05 were considered statistically significant.

## 3 Results

### 3.1 Inter-rater agreement

Five raters independently evaluated all human-scored outcomes. Inter-rater agreement was high across all evaluated domains. For Safety, Fleiss’ *κ* was 0.869 (95% CI, 0.800–0.923; *p* < 0.001). For the remaining human-scored metrics, ICC (2,1) values ranged from 0.828 to 0.883, including Accuracy, Empathy, DISCERN, EQIP, JAMA, and GQS. All agreement estimates were statistically significant (all *p* < 0.001), supporting the consistency of subsequent interface-level comparisons. Detailed inter-rater agreement results are provided in Supplementary Material 4.

### 3.2 Safety, accuracy, and empathy

Unsafe responses were identified in all six interfaces but were relatively infrequent, with safe-response rates ranging from 86.5 to 92.3%. The overall difference in Safety was not statistically significant (Cochran’s *Q* = 2.470, *df* = 5, *p* = 0.781), and no pairwise Safety comparison remained significant after Benjamini–Hochberg correction. Accordingly, the observed numerical variation should be interpreted descriptively and does not establish differential safety performance across interfaces. Interface-specific safe-response rates are presented in Table 2.

**Table 2:** Safety, accuracy, and empathy scores across interfaces.

Metric	ChatGPT	Gemini	Copilot	DeepSeek	Doubao	Qwen	Overall *P*	Effect size
Safe responses, n (%)	48 (92.3)^a^	47 (90.4)^a^	46 (88.5)^a^	47 (90.4)^a^	48 (92.3)^a^	45 (86.5)^a^	0.781	0.010
Accuracy, median [IQR]	4.00 [4.00, 4.00]^a^	4.00 [3.80, 4.00]^a^	3.00 [3.00, 3.00]^b^	3.00 [3.00, 3.00]^b^	3.00 [3.00, 3.00]^b^	3.00 [2.80, 3.00]^c^	<0.001	0.694
Empathy, median [IQR]	4.00 [4.00, 4.00]^a^	4.00 [4.00, 4.00]^a^	3.00 [3.00, 3.00]^b^	3.00 [3.00, 3.00]^b^	3.00 [3.00, 3.00]^b^	2.80 [2.00, 3.00]^c^	<0.001	0.843

Safety is presented as *n* (%), whereas Accuracy and Empathy are presented as median [IQR]. Overall comparisons used Cochran’s *Q* test for Safety and Friedman tests for Accuracy and Empathy. Overall effect sizes are *Q*/[*n* × (*k* − 1)] for Safety and Kendall’s *W* for Accuracy and Empathy. Values within the same row sharing at least one superscript letter did not differ significantly; values with no letter in common differed significantly in Benjamini–Hochberg-adjusted paired comparisons. Letters are statistical grouping labels only and should not be interpreted as interface rankings. Complete numerical pairwise estimates and 95% confidence intervals are provided in Supplementary Material 4.

All 31 responses meeting the prespecified majority-vote unsafe criterion underwent a *post hoc* exploratory reassessment. None were classified as having no meaningful potential harm; 5 (16.1%) were rated as minor, 24 (77.4%) as moderate, and 2 (6.5%) as major potential harm. The most frequent primary themes were transmission, environmental-risk, or infection-control misinformation (10/31, 32.3%); unverified jurisdiction-specific or time-sensitive information (6/31, 19.4%); and vaccine-related misinformation or overstatement (5/31, 16.1%). The complete descriptive distribution across all eight *post hoc* safety themes is provided in Supplementary Material 5. These theme-specific findings were not treated as prespecified secondary outcomes and were not used for inferential interface comparisons or rankings.

Illustrative examples included one major-harm response stating that a mother with suspected mpox could continue direct breastfeeding without adequately clarifying the required conditions and precautions, potentially allowing close skin-to-skin exposure before clinical or public-health assessment. One moderate-harm response provided unverified location-specific outbreak estimates without first confirming the user’s country, which could misdirect decisions regarding travel, testing, vaccination, or care seeking. Complete item-level classifications, including unsafe-vote counts, severity distributions, primary themes, plausible consequences, and safer-response directions, are provided in Supplementary Material 5.

**Figure 2:**
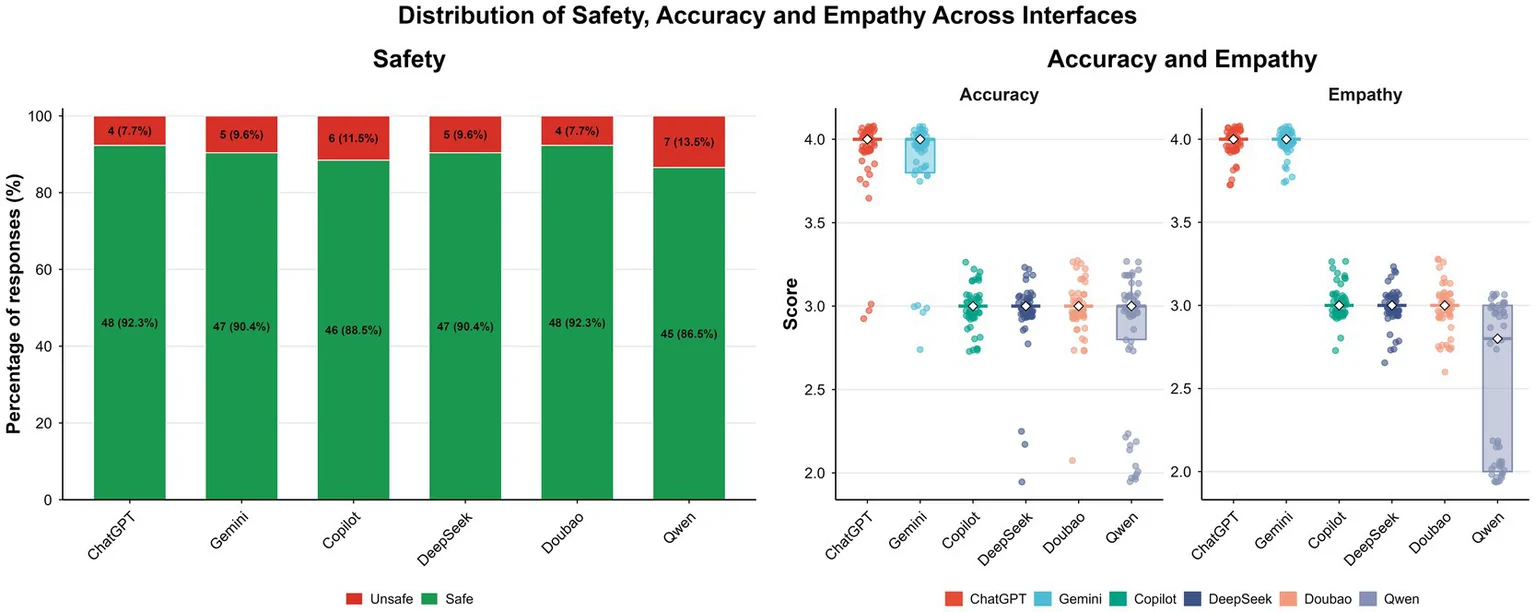
Distributions of safety, accuracy, and empathy across interfaces.

Accuracy and Empathy differed significantly across interfaces. Benjamini–Hochberg-adjusted pairwise analyses placed ChatGPT and Gemini in one statistical group, Copilot, DeepSeek, and Doubao in a second group, and Qwen in a separate lower-scoring group for both outcomes. These groupings describe the present response set and should not be interpreted as stable interface rankings. The distributions of Safety, Accuracy, and Empathy are shown in Figure 2, and the corresponding descriptive results, overall comparisons, and adjusted pairwise groupings are summarized in Table 2. Complete numerical pairwise results, including effect estimates, 95% confidence intervals, and adjusted *p* values, are provided in Supplementary Material 4.

### 3.3 Reliability/information quality

**Figure 3:**
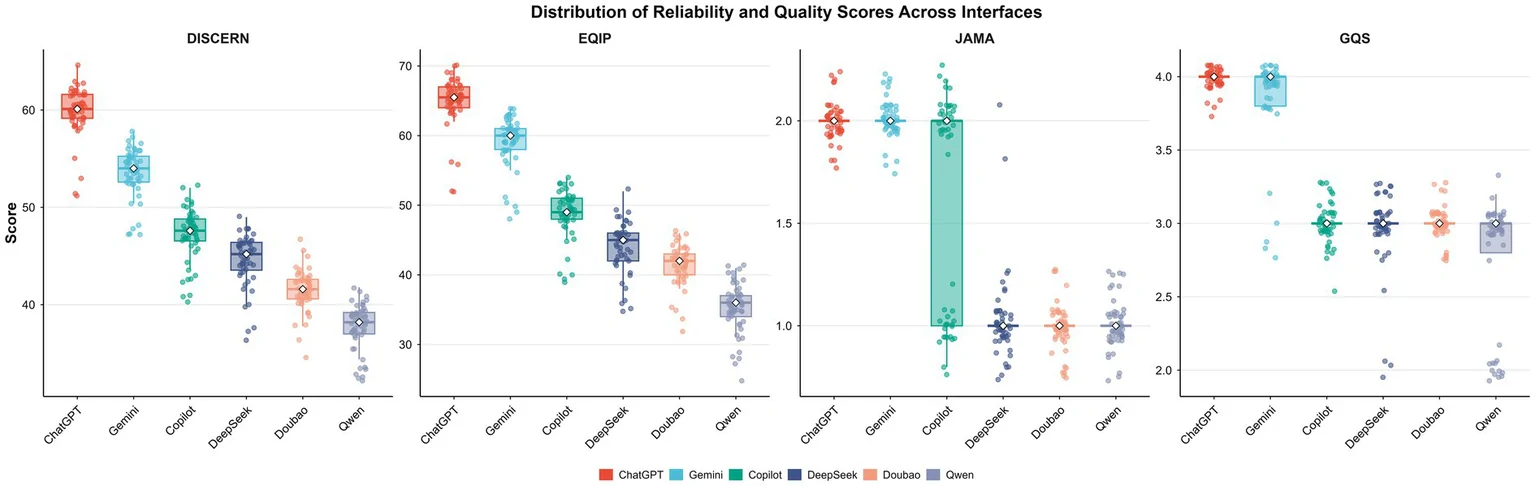
Distributions of reliability/information quality scores across interfaces.

Reliability and information-quality measures differed significantly across interfaces, although the pairwise patterns varied by measure. DISCERN and EQIP yielded distinct Benjamini–Hochberg-adjusted groupings across the six interfaces, whereas JAMA and GQS showed partial overlap among interface groups. These findings represent measure-specific differences within the present response set rather than a stable overall hierarchy. Figure 3 presents the score distributions, and Table 3 summarizes the descriptive results, overall effect sizes, and adjusted pairwise groupings. Complete numerical pairwise estimates are provided in Supplementary Material 4.

**Table 3:** Reliability/information quality scores across interfaces.

Metric	ChatGPT	Gemini	Copilot	DeepSeek	Doubao	Qwen	Overall *P*	Kendall’s *W*	Kendall’s *W* (95% CI)
DISCERN	60.10 [59.15, 61.60]^a^	54.00 [52.60, 55.25]^b^	47.60 [46.55, 48.80]^c^	45.20 [43.55, 46.40]^d^	41.60 [40.60, 42.60]^e^	38.20 [37.00, 39.20]^f^	<0.001	0.954	0.927–0.978
EQIP	65.50 [64.00, 67.00]^a^	60.00 [58.00, 61.00]^b^	49.00 [48.00, 51.00]^c^	45.00 [42.00, 46.00]^d^	42.00 [40.00, 43.00]^e^	36.00 [34.00, 37.00]^f^	<0.001	0.931	0.907–0.956
JAMA	2.00 [2.00, 2.00]^a^	2.00 [2.00, 2.00]^a^	2.00 [1.00, 2.00]^b^	1.00 [1.00, 1.00]^c^	1.00 [1.00, 1.00]^c^	1.00 [1.00, 1.00]^c^	<0.001	0.739	0.698–0.789
GQS	4.00 [4.00, 4.00]^a^	4.00 [3.80, 4.00]^b^	3.00 [3.00, 3.00]^c^	3.00 [3.00, 3.00]^c^	3.00 [3.00, 3.00]^c^	3.00 [2.80, 3.00]^d^	<0.001	0.750	0.677–0.826

Values are median [IQR]. Within each row, values sharing at least one superscript letter did not differ significantly; values with no superscript letter in common differed significantly in paired Wilcoxon signed-rank tests after Benjamini–Hochberg adjustment. Overall *p*-values were obtained using Friedman tests, and Kendall’s *W* is reported with its bootstrap 95% CI. Letters are grouping labels only and should not be interpreted as interface rankings. Full numerical pairwise estimates are provided in Supplementary Material 4.

### 3.4 Readability

**Figure 4:**
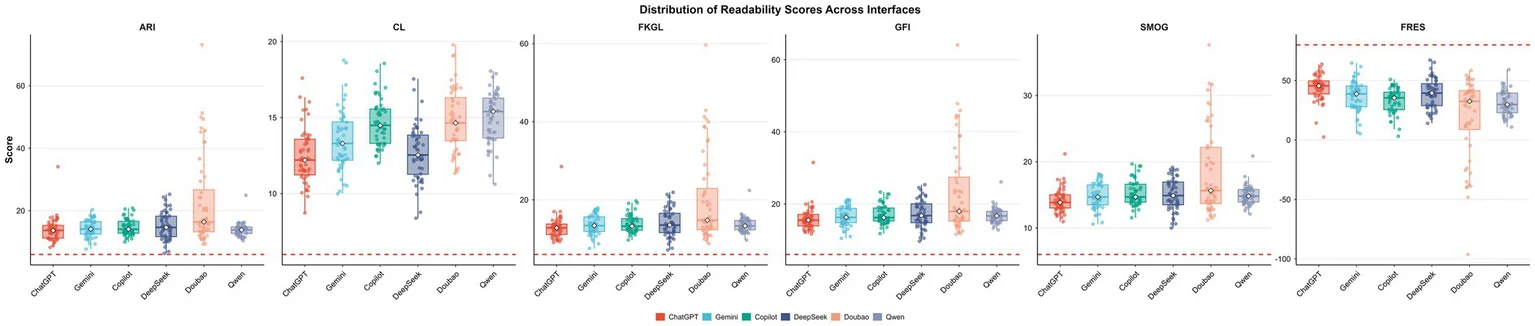
Distributions of readability scores across interfaces.

All six readability indices differed significantly across interfaces, although the pairwise patterns and overall effect sizes varied by metric. Kendall’s *W* ranged from 0.064 to 0.389, indicating that the magnitude of between-interface differences was not uniform across readability measures. Accordingly, the readability findings were metric-specific and should not be interpreted as establishing a single overall interface ranking. The distributions of readability scores are shown in Figure 4 and Table 4 summarizes the descriptive statistics, overall comparisons, effect sizes, and Benjamini–Hochberg-adjusted pairwise groupings. Complete numerical pairwise estimates, 95% confidence intervals, and adjusted *p* values are provided in Supplementary Material 4.

**Table 4:** Readability scores across interfaces.

Metric	ChatGPT	Gemini	Copilot	DeepSeek	Doubao	Qwen	Overall *p*	Kendall’s *W*	Kendall’s *W* 95% CI
ARI	13.97 ± 3.77^a^	14.24 ± 2.72^a^	14.73 ± 2.57^a^	15.15 ± 4.44^a^	22.66 ± 14.19^b^	13.95 ± 2.14^a^	0.002	0.072	0.033–0.169
CL	12.45 ± 1.83^a^	13.48 ± 1.96^b^	14.65 ± 1.48^c^	12.69 ± 1.82^a^	14.82 ± 2.06^c^	15.00 ± 1.68^c^	<0.001	0.389	0.304–0.504
FKGL	12.91 ± 2.93^a^	13.59 ± 2.44^b^	13.86 ± 2.25^b^	14.13 ± 3.57^b^	19.92 ± 11.41^c^	13.60 ± 1.92^b^	0.005	0.064	0.024–0.163
GFI	15.86 ± 3.06^a^	16.54 ± 2.67^b^	17.09 ± 2.62^b^	17.22 ± 3.82^b^	23.17 ± 12.01^c^	16.80 ± 2.14^b^	0.004	0.065	0.027–0.172
SMOG	14.11 ± 1.79^a^	14.80 ± 1.90^b^	15.27 ± 1.87^b^	15.03 ± 2.39^b^	18.32 ± 6.53^c^	14.95 ± 1.44^b^	<0.001	0.091	0.048–0.189
FRES	44.17 ± 10.83^a^	37.18 ± 12.99^b^	33.53 ± 10.33^c^	39.10 ± 12.68^b^	18.63 ± 34.39^d^	31.07 ± 10.25^cd^	<0.001	0.241	0.168–0.355

Higher FRES values indicate easier readability, whereas lower ARI, CL, FKGL, GFI, and SMOG values indicate easier readability. Within each row, values sharing at least one superscript letter did not differ significantly; values with no superscript letter in common differed significantly in paired Wilcoxon signed-rank tests after Benjamini–Hochberg adjustment. Overall *p*-values were obtained using Friedman tests, and Kendall’s *W* is reported with its bootstrap 95% CI. Letters are grouping labels only and should not be interpreted as interface rankings. Full numerical pairwise estimates are provided in Supplementary Material 4.

## 4 Discussion

### 4.1 Main findings

In this comparative evaluation of six publicly accessible search-enabled LLM interfaces for mpox-related public health consultation, unsafe responses were relatively infrequent and did not differ significantly across interfaces after correction for multiple comparisons; nevertheless, potentially harmful outputs occurred in every interface. Significant between-interface differences were observed for Accuracy, Empathy, reliability/information quality, and readability, although the magnitude and pairwise patterns varied by measure. These findings represent interface-level performance within the present response set and recorded query conditions rather than evidence of intrinsic or stable superiority.

Mpox consultation is particularly demanding because responses must integrate symptom recognition, transmission risk, post-exposure action, vaccination, care seeking, jurisdiction-specific guidance, uncertainty, and non-stigmatizing communication (1, 5, 8–10, 33). The identified safety concerns—including unsupported local-risk estimates, over-reassurance, ambiguous post-exposure advice, overstated vaccine or treatment effects, and stigma-sensitive framing—illustrate why fluent answers may still be incomplete or unsafe. In the present mpox-specific context, search-enabled LLM interfaces may warrant further evaluation for a possible adjunctive role in mpox-related information delivery; however, the present response-level evaluation does not establish their effectiveness, acceptability, or influence on user behavior. These findings support disease-specific evaluation, ongoing monitoring, and appropriate safeguards before broader use in mpox consultation (15, 17).

### 4.2 Safety, accuracy, and empathy

Safe-response rates were numerically high across the evaluated interfaces, but neither the overall Safety comparison nor any Benjamini–Hochberg-adjusted pairwise comparison was statistically significant. The observed differences in percentages should therefore be interpreted descriptively rather than as evidence of differential safety performance. Nevertheless, every interface produced responses meeting the prespecified majority-vote unsafe criterion. Among the 31 unsafe responses, recurrent concerns involved unverified jurisdiction-specific information, transmission or contaminated-material risks, post-exposure management, vaccination or treatment claims, atypical presentations, and stigmatizing communication. The exploratory reassessment further showed variation in potential-harm severity. Thus, the binary Safety outcome identifies whether a response met the unsafe criterion but does not fully convey the type, severity, or plausible clinical and public-health consequences of the content, particularly in relation to exposure management, vaccination, isolation, and time-sensitive guidance (1, 40–42).

The unsafe-response range observed in the present study (7.7–13.5%) broadly overlaps with the 5–13% reported by Draelos et al. in an evaluation of 888 responses to 222 patient-posed medical questions (43). Direct numerical comparison is limited by differences in clinical scope, question construction, safety definitions, evaluator composition, and query procedures. Nevertheless, both studies indicate that generally high safe-response rates do not eliminate potentially important safety failures.

Accuracy showed clearer between-interface differentiation than binary Safety, although these findings should be interpreted as condition-specific interface-level patterns rather than intrinsic differences between the underlying base models or evidence of stable interface superiority. Previous studies have similarly shown that fluent patient-facing responses may still be incomplete, insufficiently qualified, or unsupported (12–14, 16). In contrast to the present findings, a guideline-based evaluation of 27 hypothyroidism questions reported high overall accuracy across ChatGPT-3.5, DeepSeek-V3, and Gemini-2.5, without significant overall differences in Accuracy or DISCERN scores (44). The different patterns may partly reflect differences in disease context, question breadth, prompting strategy, search and retrieval conditions, interface versions, and the greater importance of time-sensitive, jurisdiction-dependent, and exposure-specific guidance in mpox consultation.

Empathy also varied across interfaces. In mpox consultation, empathetic communication is clinically and publicly relevant because questions frequently involve sexual exposure, pregnancy, children, immunocompromise, privacy, and stigma. Responses should be supportive without minimizing risk and nonjudgmental without obscuring necessary clinical or public-health action. Although previous studies have reported favorable empathy ratings for some chatbot-generated responses (12, 20), the present findings indicate that supportive and non-stigmatizing communication was not uniform across the evaluated interfaces. Empathy, privacy protection, and stigma avoidance should therefore be regarded as safety-relevant communication dimensions rather than optional stylistic features (40). Overall, these findings support multidimensional evaluation incorporating Safety, Accuracy, and communication quality rather than reliance on fluency or binary Safety alone.

### 4.3 Reliability/information quality

Reliability and information quality varied significantly across the evaluated interfaces, although the pairwise patterns differed by instrument. This indicates that apparently plausible and well-structured responses may still vary in evidence alignment, completeness, transparency, and practical usefulness. These findings should be interpreted as properties of the evaluated interfaces, retrieval settings, and query period rather than as stable characteristics of the underlying base models.

Our findings parallel a recent public-health evaluation of chatbot-generated information on thunderstorm asthma, which used DISCERN, EQIP, JAMA benchmarks, GQS, and the same six readability indices and likewise identified instrument-dependent differences across interfaces (25). Together, these studies support multidimensional assessment rather than reliance on a single global quality score, because fluent or comprehensive responses may still lack source attribution, currency, uncertainty disclosure, or actionable guidance.

JAMA scores were generally low, indicating limited transparency regarding authorship, attribution, disclosure, and currency (23). This is particularly relevant to mpox because current risk, vaccine eligibility, diagnostic access, travel advice, and public-health recommendations may vary across time and jurisdictions. The qualitative safety findings further showed that unclear source grounding or temporal applicability may contribute to misleading local-risk estimates, over-reassurance, and inappropriate exposure, vaccination, or treatment advice. Public-facing interfaces should therefore distinguish stable medical knowledge from changing public-health guidance, clearly communicate uncertainty and source dates, and direct users to authoritative services when recommendations are time- or jurisdiction-dependent. Future evaluations should continue to combine measures of reliability, patient-information quality, transparency, and actionability with qualitative safety analysis (21–23, 26).

### 4.4 Readability

Readability differed significantly across the evaluated interfaces, although the effect sizes and pairwise patterns varied by index. The findings were therefore metric-specific and should not be interpreted as establishing a single overall readability ranking. This variation remains relevant because users may need to make timely decisions regarding care seeking, symptom monitoring, vaccination, exposure precautions, and contact with public-health services. Even factually accurate information may have limited practical value if it is text-dense, jargon-heavy, or difficult to translate into action. Public-facing responses should therefore prioritize plain language, concise sentences, explicit action steps, and clear differentiation between urgent and non-urgent situations (18, 19).

Readability formulas primarily capture surface-level textual characteristics, such as sentence length, word length, syllables, and characters, rather than actual comprehension, trust, emotional response, behavior, or medical correctness (27–32). Moreover, previous studies suggest that readability patterns may vary by clinical topic, interface version, query settings, and prompt design (25, 44–46). Future research should therefore combine formula-based indices with direct testing among public users, particularly those with lower health literacy, high anxiety following possible exposure, or limited access to professional health advice.

### 4.5 Practical implications

Within the context of mpox-related public health consultation, clinicians should recognize that patients may seek or present with chatbot-generated information and should verify exposure history, symptoms, vaccination status, and jurisdiction-specific recommendations rather than assuming that the generated advice is accurate or current. Public-health professionals may consider periodically reviewing the mpox-related information provided by widely used interfaces, maintaining accessible and frequently updated official guidance, and encouraging clear presentation of source dates, geographic applicability, uncertainty, and escalation pathways. For members of the public, any mpox information obtained from search-enabled LLM interfaces should be treated as preliminary, verified against qualified clinical or public-health sources, and not used as a substitute for professional assessment, particularly after a relevant exposure, when new lesions or systemic symptoms occur, or for pregnant, pediatric, or immunocompromised individuals. Advice concerning testing, isolation, vaccination, treatment, and travel should be confirmed through local health authorities or qualified clinicians. In mpox-related public-facing communication, responses should use plain language, concise action steps, and non-stigmatizing wording (8–10, 18, 19, 40).

### 4.6 Limitations

This study has several limitations. First, the question set was investigator-developed through purposive multi-source identification rather than sampled from a validated or probabilistically representative public-question dataset. Although the source platforms, screening criteria, agreement results, and item-level decisions were documented, prioritization within the fixed 52-question framework required investigator judgment. Other researchers might therefore derive a different question set, and the present benchmark should be interpreted as a structured, coverage-oriented sample rather than a representative sample of all public mpox queries.

Second, explicit interface identifiers were removed from the rating materials, but the six responses within each question block were not randomized, and the effectiveness of identity masking was not formally assessed. Raters may have inferred interface identity from linguistic style, response structure, or source-presentation patterns, and the fixed order may have introduced position or expectation effects. High inter-rater agreement indicates scoring consistency but does not exclude shared systematic bias. Additionally, the majority-vote rule reduced five individual judgments to a binary response-level classification. Safety concerns raised by only one or two raters were therefore not captured in the primary Safety outcome, and the reported safe-response rates should not be interpreted as unanimous agreement, although complete de-identified rater-level ratings are available in Supplementary Material 6.

Third, each question was submitted only once to each interface during a predefined query period. Search-enabled outputs may vary across sessions because of stochastic generation, retrieved sources, interface updates, geographic location, and time. The findings therefore represent a single-query cross-sectional snapshot and do not establish session-level reproducibility, temporal stability, or stable between-interface rankings.

Fourth, the severity and thematic analyses were *post hoc* and reflected expert judgments of plausible potential harm rather than observed user behavior or clinical outcomes. Actual consequences may vary according to health literacy, vulnerability, exposure context, and access to care. Because the same raters conducted both the original binary assessment and the exploratory reassessment, prior familiarity with some responses and residual inference of interface identity cannot be excluded. Fifth, all interfaces were evaluated with web-search or intelligent-search functions enabled. Consequently, the observed performance cannot be attributed solely to the underlying language models and may also reflect retrieval quality, source selection, search ranking, and interface-level safety mechanisms.

Sixth, the standardized prompts were evaluated in English, and the study relied on expert scoring and formula-based readability indices rather than direct testing with members of the public. The findings may therefore not fully generalize to other linguistic and cultural settings or capture actual comprehension, trust, emotional response, risk perception, and health-related behavior. In addition, DISCERN, EQIP, JAMA, and related measures evaluated the displayed responses as written consumer health information rather than the complete interactive or technical capabilities of the interfaces.

Finally, the guideline-based reference framework was developed specifically for this study and was not an independently validated gold standard. Although authoritative guidelines and recommendations were prioritized and all entries underwent independent external expert review, the selection and interpretation of evidence and the formulation of expected public-facing answers involved expert judgment. This subjectivity may have influenced response scoring and the observed performance of the evaluated interfaces.

## 5 Conclusion

In this single-query comparative cross-sectional evaluation, the six publicly accessible search-enabled LLM interfaces showed heterogeneous response-level performance across Safety, Accuracy, Empathy, reliability/information quality, and readability in mpox-related public health consultation. Potentially harmful outputs were identified in every interface, particularly in time-sensitive, jurisdiction-specific, post-exposure, vaccination, treatment, contaminated-material, and stigma-sensitive scenarios. These findings underscore the need for guideline-based evaluation, source transparency, readability optimization, and robust safety safeguards. However, because public comprehension, trust, behavioral responses, care-seeking decisions, and clinical or public-health outcomes were not evaluated, this study does not establish the real-world effectiveness or suitability of these interfaces as public-facing information tools. Any potential adjunctive role in mpox communication requires prospective, user-centered evaluation. The findings represent a time- and configuration-specific interface-level snapshot under the recorded testing conditions and should not be interpreted as evidence of reproducible performance or consistent superiority of any particular interface across sessions, versions, or settings. Whether similar patterns occur in other infectious-disease or public-health contexts remains uncertain and should be examined in separate disease-specific studies.

## Data Availability

The original contributions presented in the study are included in the article/Supplementary material, further inquiries can be directed to the corresponding author.

## References

[1] World Health Organization Mpox Geneva, Switzerland: World Health Organization (2024). Available online at: https://www.who.int/news-room/fact-sheets/detail/mpox (Accessed August 16, 2026).

[2] Laurenson-Schafer H Sklenovská N Hoxha A Kerr SM Ndumbi P Fitzner J . Description of the first global outbreak of mpox: an analysis of global surveillance data. Lancet Glob Health. (2023) 11:e1012–23. doi: 10.1016/S2214-109X(23)00198-5, 37349031 PMC10281644

[3] Thornhill JP Barkati S Walmsley S Rockstroh J Antinori A Harrison LB . Monkeypox virus infection in humans across 16 countries - April-June 2022. N Engl J Med. (2022) 387:679–91. doi: 10.1056/nejmoa2207323, 35866746

[4] Centers for Disease Control and Prevention Monkeypox in the United States and around the World. Atlanta, GA, USA: Centers for Disease Control and Prevention (2026) Available online at: https://www.cdc.gov/monkeypox/situation-summary/index.html (Accessed August 16, 2026).

[5] World Health Organization. Mpox Global Strategic Preparedness and Response Plan. Geneva: World Health Organization (2024).

[6] World Health Organization WHO Recommends New Name for Monkeypox Disease Geneva: World Health Organization (2022). Available online at: https://www.who.int/news/item/28-11-2022-who-recommends-new-name-for-monkeypox-disease (Accessed August 16, 2026).

[7] Webb E Rigby I Michelen M Dagens A Cheng V Rojek AM . Availability, scope and quality of monkeypox clinical management guidelines globally: a systematic review. BMJ Glob Health. (2022) 7:e009838. doi: 10.1136/bmjgh-2022-009838, 35973747 PMC9472169

[8] Rao AK Minhaj FS Carter RJ Duffy J Satheshkumar PS Delaney KP . Use of JYNNEOS (smallpox and mpox vaccine, live, nonreplicating) for persons aged ≥18 years at risk for mpox during an mpox outbreak: recommendations of the advisory committee on immunization practices - United States, 2023. MMWR Morb Mortal Wkly Rep. (2025) 74:385–92. doi: 10.15585/mmwr.mm7422a3, 40531798 PMC12176103

[9] Rao AK Petersen BW Whitehill F Razeq JH Isaacs SN Merchlinsky MJ . Use of JYNNEOS (smallpox and monkeypox vaccine, live, nonreplicating) for preexposure vaccination of persons at risk for occupational exposure to Orthopoxviruses: recommendations of the advisory committee on immunization practices - United States, 2022. MMWR Morb Mortal Wkly Rep. (2022) 71:734–42. doi: 10.15585/mmwr.mm7122e1, 35653347 PMC9169520

[10] Rao AK Schrodt CA Minhaj FS Waltenburg MA Cash-Goldwasser S Yu Y . Interim clinical treatment considerations for severe manifestations of mpox - United States, February 2023. MMWR Morb Mortal Wkly Rep. (2023) 72:232–43. doi: 10.15585/mmwr.mm7209a4, 36862595 PMC9997665

[11] Ugwu CLJ Bragazzi NL Wu J Kong JD Asgary A Orbinski J . Risk factors associated with human mpox infection: a systematic review and meta-analysis. BMJ Glob Health. (2025) 10:e016937. doi: 10.1136/bmjgh-2024-016937, 39900427 PMC11795413

[12] Ayers JW Poliak A Dredze M Leas EC Zhu Z Kelley JB . Comparing physician and artificial intelligence chatbot responses to patient questions posted to a public social media forum. JAMA Intern Med. (2023) 183:589–96. doi: 10.1001/jamainternmed.2023.1838, 37115527 PMC10148230

[13] Singhal K Azizi S Tu T Mahdavi SS Wei J Chung HW . Large language models encode clinical knowledge. Nature. (2023) 620:172–80. doi: 10.1038/s41586-023-06291-2, 37438534 PMC10396962

[14] Aydin S Karabacak M Vlachos V Margetis K. Large language models in patient education: a scoping review of applications in medicine. Front Med. (2024) 11:1477898. doi: 10.3389/fmed.2024.1477898, 39534227 PMC11554522

[15] Sarink MJ Bakker IL Anas AA Yusuf E. A study on the performance of ChatGPT in infectious diseases clinical consultation. Clin Microbiol Infect. (2023) 29:1088–9. doi: 10.1016/j.cmi.2023.05.017, 37207982

[16] Wei Q Yao Z Cui Y Wei B Jin Z Xu X. Evaluation of ChatGPT-generated medical responses: a systematic review and meta-analysis. J Biomed Inform. (2024) 151:104620. doi: 10.1016/j.jbi.2024.104620, 38462064

[17] The CHART Collaborative. Reporting guideline for chatbot health advice studies: chatbot assessment reporting tool (CHART) statement. Ann Fam Med. (2025) 23:389–98. doi: 10.1370/afm.250386 40750305 PMC12459699

[18] Centers for Disease Control and Prevention CDC Clear Communication Index U.S. Atlanta, GA, USA: Department of Health and Human Services (2024). Available online at: https://www.cdc.gov/ccindex/index.html (Accessed August 16, 2026).

[19] Rooney MK Santiago G Perni S Horowitz DP McCall AR Einstein AJ . Readability of patient education materials from high-impact medical journals: a 20-year analysis. J Patient Exp. (2021) 8:2374373521998847. doi: 10.1177/2374373521998847, 34179407 PMC8205335

[20] Mercer SW Maxwell M Heaney D Watt GC. The consultation and relational empathy (CARE) measure: development and preliminary validation and reliability of an empathy-based consultation process measure. Fam Pract. (2004) 21:699–705. doi: 10.1093/fampra/cmh621, 15528286

[21] Charnock D Shepperd S Needham G Gann R. DISCERN: an instrument for judging the quality of written consumer health information on treatment choices. J Epidemiol Community Health. (1999) 53:105–11. doi: 10.1136/jech.53.2.105, 10396471 PMC1756830

[22] Moult B Franck LS Brady H. Ensuring quality information for patients: development and preliminary validation of a new instrument to improve the quality of written health care information. Health Expect. (2004) 7:165–75. doi: 10.1111/j.1369-7625.2004.00273.x, 15117391 PMC5060233

[23] Silberg WM Lundberg GD Musacchio RA. Assessing, controlling, and assuring the quality of medical information on the internet: caveant lector et viewor--let the reader and viewer beware. JAMA. (1997) 277:1244–5. 9103351

[24] Yıldız HA Söğütdelen E. AI chatbots as sources of STD information: a study on reliability and readability. J Med Syst. (2025) 49:43. doi: 10.1007/s10916-025-02178-z, 40178771 PMC11968469

[25] Zhu Z Feng Y Cao F. The information challenge in public health crises: a study on the reliability and readability of information provided by large language model for thunderstorm asthma. Front Public Health. (2026) 14:1776697. doi: 10.3389/fpubh.2026.1776697, 41788533 PMC12957218

[26] Bernard A Langille M Hughes S Rose C Leddin D Veldhuyzen van Zanten S. A systematic review of patient inflammatory bowel disease information resources on the world wide web. Am J Gastroenterol. (2007) 102:2070–7. doi: 10.1111/j.1572-0241.2007.01325.x, 17511753

[27] Smith EA Senter RJ. Automated Readability Index. Wright-Patterson AFB, OH, USA: AMRL-TR Aerospace Medical Research Laboratories (US) (1967). p. 1–14. 5302480

[28] Coleman M Liau TL. A computer readability formula designed for machine scoring. J Appl Psychol. (1975) 60:283–4. doi: 10.1037/h0076540

[29] Kincaid JP Fishburne RP Rogers RL Chissom BS. Derivation of New Readability Formulas (Automated Readability Index, Fog Count and Flesch Reading Ease Formula) for Navy Enlisted Personnel. Millington, TN: (1975) Chief of Naval Technical Training, Naval Air Station Memphis. Report No.: Research Branch Report). p. 8–75.

[30] Flesch R. A new readability yardstick. J Appl Psychol. (1948) 32:221–33. doi: 10.1037/h0057532, 18867058

[31] McLaughlin GH. SMOG grading: a new readability formula. J Reading. (1969) 12:639–46.

[32] Gunning R. The Technique of Clear Writing. New York: McGraw-Hill (1952).

[33] Benoit K Watanabe K Wang H Nulty P Obeng A Müller S . Quanteda: an R package for the quantitative analysis of textual data. J Open Source Softw. (2018) 3:774. doi: 10.21105/joss.00774

[34] Benjamini Y Hochberg Y. Controlling the false discovery rate: a practical and powerful approach to multiple testing. J R Stat Soc Ser B Stat Methodol. (1995) 57:289–300. doi: 10.1111/j.2517-6161.1995.tb02031.x

[35] Fiel Peres F. Effect sizes for nonparametric tests. Biochem Med. (2026) 36:5–16. doi: 10.11613/bm.2026.010101, 41399660 PMC12701665

[36] Efron B Tibshirani RJ. An Introduction to the Bootstrap. 1st ed. New York: Chapman & Hall (1993). p. 436.

[37] Fleiss JL. Measuring nominal scale agreement among many raters. Psychol Bull. (1971) 76:378–82. doi: 10.1037/h0031619

[38] Shrout PE Fleiss JL. Intraclass correlations: uses in assessing rater reliability. Psychol Bull. (1979) 86:420–8. doi: 10.1037/0033-2909.86.2.420, 18839484

[39] Hayes AF Krippendorff K. Answering the call for a standard reliability measure for coding data. Commun Methods Meas. (2007) 1:77–89. doi: 10.1080/19312450709336664

[40] World Health Organization WHO Publishes public Health Advice on Preventing and Addressing stigma and Discrimination Related to mpox. Geneva, Switzerland: World Health Organization (2022). Available online at: https://www.who.int/news/item/11-12-2022-who-publishes-public-health-advice-on-preventing-and-addressing-stigma-and-discrimination-related-to-mpox (Accessed August 16, 2026).

[41] Centers for Disease Control and Prevention Risk Assessment and Monitoring in Community Settings. Atlanta, GA, USA: Centers for Disease Control and Prevention (2026) Available online at: https://www.cdc.gov/monkeypox/php/monitoring/index.html (Accessed August 16, 2026).

[42] Centers for Disease Control and Prevention Vaccine for Monkeypox Prevention in the United States. Atlanta, GA, USA: Centers for Disease Control and Prevention (2026). Available online at: https://www.cdc.gov/monkeypox/hcp/vaccine-considerations/index.html (Accessed August 16, 2026).

[43] Draelos RL Afreen S Blasko B Brazile TL Chase N Desai DP . Large language models provide unsafe answers to patient-posed medical questions. NPJ Digit Med. (2026) 9:241. doi: 10.1038/s41746-026-02428-5, 41688533 PMC13013898

[44] Ruan T Shao X Sun Y Ju X Cui J. Evaluation of accuracy, quality, and readability of information on hypothyroidism provided by different artificial intelligence chatbot models. Front Public Health. (2025) 13:1698596. doi: 10.3389/fpubh.2025.1698596, 41450516 PMC12727900

[45] Tepe M Emekli E. Assessing the responses of large language models (ChatGPT-4, Gemini, and Microsoft copilot) to frequently asked questions in breast imaging: a study on readability and accuracy. Cureus. (2024) 16:e59960. doi: 10.7759/cureus.59960, 38726360 PMC11080394

[46] Hancı V Ergün B Gül Ş Uzun Ö Erdemir İ Hancı FB. Assessment of readability, reliability, and quality of ChatGPT®, BARD®, Gemini®, copilot®, perplexity® responses on palliative care. Medicine. (2024) 103:e39305. doi: 10.1097/MD.0000000000039305, 39151545 PMC11332738

