# Supplementary material for: Evaluating search-enabled large language model interfaces for mpox public health consultation: a guideline-based comparative study
Source: Front Public Health. 2026 Aug 26;14:1913260. doi: 10.3389/fpubh.2026.1913260 (PMC13553459; doi:10.3389/fpubh.2026.1913260)
Supplement: Supplementary file 1 [file Supplementary_file_1.docx]

**Development and Screening of the Mpox Public Consultation Question Set**

Detailed Question-Set Development and Screening Record

| **Item** | **Content** |
| --- | --- |
| Record version | V1.0 for locking the original non-journal-adapted question set before formal LLM evaluation |
| Question-set lock date | April 15, 2026 |
| Initial question pool | 120 public-facing questions written in natural consultation style |
| Final included questions | 52 |
| Final excluded questions | 68 |
| Record purpose | To document sources, deduplication, screening, dimensional balancing, and final inclusion/exclusion decisions. |

**1. Question Sources and Initial Development**

| **No.** | **Source/platform and access route** | **Search/access date and period** | **Search terms, settings, or selection approach** | **Pre-merge count** | **Role in question development** |
| --- | --- | --- | --- | --- | --- |
| 1 | **Google Trends**  Stable entry/accessed platform:  https://trends.google.com/trends/ | Accessed April 10, 2026; analysis period: April 10, 2021–April 10, 2026. | Seed terms: “mpox” and “monkeypox.” Geographic setting: Worldwide. Time range: past 5 years. Category: All categories. Search type: Web Search. All TOP related queries were exported and reviewed; RISING results were not used. | 23 trend terms/phrases | Generated candidate questions on basic knowledge, symptoms, outbreak awareness, vaccines, WHO, and cases. |
| 2 | **Baidu Index and Baidu Search**  Baidu Index:  https://index.baidu.com/v2/index.html#/  Baidu Search:  https://www.baidu.com/ | Accessed April 10, 2026; Baidu Index period: April 10, 2021–April 10, 2026. | Seed term: “猴痘.” Baidu Index geographic setting: China; Demand Graph (需求图谱) module. Ordinary Baidu Search was additionally reviewed for related suggestions and commonly presented public questions. Related themes included 猴痘症状, 猴痘传播, 猴痘疫苗, 猴痘图片, and 猴痘会传染吗. | 20 Chinese search signals | Supplemented Chinese public phrasing and local information-seeking patterns. |
| 3 | **Public online forums and Q&A platforms**  Zhihu stable entry: https://www.zhihu.com/search  Accessed search: https://www.zhihu.com/search?q=%E7%8C%B4%E7%97%98  Reddit stable entry: https://www.reddit.com/search/  Accessed searches: https://www.reddit.com/search/?q=monkeypox; https://www.reddit.com/search/?q=mpox  Baidu Health stable entry: https://health.baidu.com/  Accessed page retained in query log: https://health.baidu.com/m/detail/ar_6395042220752913656 | Accessed April 10, 2026. | Zhihu and Baidu Health were searched using “猴痘”; Reddit was searched using “monkeypox” and “mpox.” Public themes included rash recognition, fear/reassurance, privacy, possible misdiagnosis, sexual exposure, post-exposure actions, and care-seeking. No usernames, personal identifiers, or verbatim quotations were retained. | 46 candidate questions | Ensured that questions sounded like real public questions rather than guideline headings. |
| 4 | Professional patient-education resources: WHO, CDC, China public-health authorities, and Mayo Clinic (https://www.chinacdc.cn/jkyj/crb2/yl/hd/gnyq/; https://www.ndcpa.gov.cn/jbkzzx/c100014/common/content/content_1698984403881291776.html; https://search.cdc.gov/search/?query=mpox&dpage=1; https://www.who.int/health-topics/; https://www.mayoclinic.org/) | Accessed April 5–9, 2026. | Relevant mpox/monkeypox patient-education pages were identified through institutional topic pages and site searches. Topics included symptoms, transmission, diagnosis, prevention, vaccination, care-seeking, home care, travel, and special populations. | 29 candidate questions | Added topics on symptoms, transmission, prevention, vaccination, care seeking, and home care. |
| 5 | **Guidelines, consensus documents, and reviews** Full evidence list provided in Supplementary Material 3 | Identified and reviewed April 5–9, 2026. | Diagnosis, prevention and control, post-exposure management, vaccination, treatment, infection prevention, special populations, and non-stigmatizing communication. | 32 candidate questions | Ensured medical coverage and availability of guideline-based evaluation framework. |
| 6 | **Expert supplementation**  Structured infectious-disease/public-health expert discussion; no external platform. | Completed before question-set locking on April 15, 2026. | Evidence sources were purposively identified using “mpox,” “monkeypox,” “guideline,” “consensus,” “diagnosis,” “treatment,” “prevention,” “vaccination,” “post-exposure,” and “special populations.” Formal guidance and consensus documents were prioritized; reviews were used as supplementary context. | 18 ~~supplementary/revised questions~~ candidate questions | Improved discriminative value for safety and empathy evaluation. |
| Total | **Combined pre-deduplication signals** | Question-identification period: April 5–10, 2026; final set locked April 15, 2026. | Substantial overlap existed across sources.After deduplication, merging, and public-language rewriting, 120 candidate questions were retained for independent screening. | 168 items | After deduplication, merging, and wording revision, 120 candidate questions were retained. |

**2. Inclusion and Exclusion Criteria**

| **Type** | **Criterion** |
| --- | --- |
| Inclusion criterion 1 | The question had to be a natural-language question that a member of the public might ask a search engine, Q&A platform, or chatbot. |
| Inclusion criterion 2 | Each item had to contain only one question mark and focus on a single scoring task. |
| Inclusion criterion 3 | The item had to cover high-frequency or high-risk scenarios relevant to safety, accuracy, empathy, reliability, or readability assessment. |
| Inclusion criterion 4 | The item had to be scoreable against publicly available authoritative materials or expert consensus. |
| Inclusion criterion 5 | The final 52-question set had to maintain balance across basic knowledge, symptoms, differential diagnosis, transmission, post-exposure management, diagnosis, treatment, vaccines, special populations, privacy/non-stigmatization, travel/public places, and environmental/misinformation domains. |
| Exclusion criterion 1 | The item substantially overlapped with an included question or could be covered by a more central item. |
| Exclusion criterion 2 | The item depended heavily on real-time local policy, local medical resources, or individualized clinical details that would prevent standardized scoring. |
| Exclusion criterion 3 | The item was too technical, too specialized, or too guideline-like to represent public consultation language. |
| Exclusion criterion 4 | The item contained multiple evaluation tasks or was not sufficiently focused for reliable scoring. |
| Exclusion criterion 5 | The scenario was too narrow or lower priority under the fixed 52-question sample size. |

**3. Screening Flow Summary**

The same two researchers—one attending infectious disease physician and one chief infectious disease nurse—independently screened the candidate questions at both stages using the predefined criteria. Disagreements were first discussed by the two screeners; unresolved items were adjudicated by a chief infectious-disease physician.

**Table S1. Inter-screener agreement summary**

| **Screening stage** | **Items assessed** | **Raw agreement, n (%)** | **Cohen’s κ (95% CI)** | **Disagreements** | **Resolved by discussion** | **Adjudicate** |
| --- | --- | --- | --- | --- | --- | --- |
| Stage 1: relevance and scoreability | 120 | 111 (92.5%) | 0.798 (0.671–0.924) | 9 | 6 | 3 |
| Stage 2: priority and domain balance | 89 | 77 (86.5%) | 0.720 (0.573–0.867) | 12 | 8 | 4 |

**Table S2. Stage 1 independent screening decisions (N = 120)**

|  |  | **Screener B** | |
| --- | --- | --- | --- |
|  | Decision | Include | Exclude |
| Screener A | Include | a = 86 | b =5 |
| Screener A | Exclude | c =4 | d = 25 |

**Table S3. Stage 2 independent screening decisions (N = 89)**

|  |  | **Screener B** | |
| --- | --- | --- | --- |
|  | Decision | Include | Exclude |
| Screener A | Include | a = 47 | b = 6 |
| Screener A | Exclude | c = 6 | d = 30 |

**Calculation note: Observed agreement = (a + d) / N. Cohen’s κ = (Pₒ − Pₑ) / (1 − Pₑ), where Pₑ is calculated from the two screeners’ marginal inclusion/exclusion totals. Observed agreement and Cohen’s κ were calculated from the two screeners’ original independent decisions before discussion or adjudication.**

**Table S4. Screening flow from 168 signals to the final 52-question set**

| **Stage** | **Entered** | **Excluded at stage** | **Retained** | **Notes** |
| --- | --- | --- | --- | --- |
| 1. Multi-source identification | 168 | 0 | 168 | Items were identified from search trends, Chinese search-intent signals, forum/Q&A materials, patient education materials, guidelines/reviews, and expert supplementation. |
| 2. Deduplication, merging, and public-language rewriting | 168 | 48 | 120 | Synonymous items were merged; wording was standardized into public consultation style; each retained item was limited to one question mark. |
| 3. Initial screening for relevance and scoreability | 120 | 31 | 89 | Items that were too narrow, too technical, redundant, or difficult to score against a uniform reference standard were removed. |
| 4. Secondary screening for dimensional balance and expert-prioritized scenarios | 89 | 37 | 52 | Representative high-frequency, high-risk, and empathy-sensitive scenarios were retained across dimensions. |
| 5. Final locking | 52 | 0 | 52 | The final 52-question set was locked for pilot scoring and formal LLM evaluation. |

**4. Exclusion-Reason Summary**

| **No.** | **Exclusion reason** | **Count** |
| --- | --- | --- |
| 1 | Overlapped substantially with included basic knowledge and outbreak-risk questions; more common phrasings were retained. | 6 |
| 2 | Covered by included questions on early symptoms, localized rash, and fever without rash; this avoided overrepresentation within the same dimension. | 5 |
| 3 | Represented narrower differential-diagnosis scenarios; higher-risk or more common differential questions were retained. | 6 |
| 4 | Represented detailed everyday-contact scenarios; more discriminating transmission-risk questions were retained. | 5 |
| 5 | Overlapped with core close-contact management questions or depended heavily on local public-health procedures. | 6 |
| 6 | Focused on relatively narrow testing-process details; care-seeking, sample type, privacy, and urgent-care triggers were prioritized. | 5 |
| 7 | Home-care subtopics were numerous; treatment, pain control, and contaminated item handling were retained as representative questions. | 6 |
| 8 | Vaccine subtopics were numerous; the most common and safety-critical vaccine questions were prioritized. | 6 |
| 9 | Special-population subtopics were numerous; children, pregnancy, HIV, and breastfeeding scenarios were retained. | 6 |
| 10 | Sexual-contact subtopics were numerous; STI framing, exposure response, stigma, and partner notification were retained. | 5 |
| 11 | Travel and public-place scenarios were narrower or depended on institutional policies; travel, flight, hotel, and family-contact decisions were retained. | 6 |
| 12 | Animal, environmental, and misinformation questions were numerous; animal origin, pets, disinfection, and unproven remedies were retained. | 6 |
| Total | Final number of excluded questions | 68 |

**5. Dimensional Distribution of the Final 52 Questions**

| **No.** | **Dimension** | **Included questions** |
| --- | --- | --- |
| 1 | A. Basic knowledge and outbreak awareness | 4 |
| 2 | B. Symptom recognition and early judgment | 5 |
| 3 | C. Differential diagnosis | 4 |
| 4 | D. Transmission routes and everyday contact risk | 5 |
| 5 | E. Post-exposure management and close-contact monitoring | 4 |
| 6 | F. Testing, diagnosis, and care-seeking pathways | 5 |
| 7 | G. Treatment, medication, and home care | 4 |
| 8 | H. Vaccination and immune protection | 4 |
| 9 | I. High-risk and special populations | 4 |
| 10 | J. Sexual contact, privacy, and non-stigmatizing communication | 5 |
| 11 | K. Travel, work, school, and public places | 4 |
| 12 | L. Animals, environmental disinfection, and misinformation | 4 |
| Total | All dimensions | 52 |

**6. Included Question Record**

| **Included No.** | **Original ID** | **Dimension** | **Question text** | **Main source signal** | **Reason for inclusion** |
| --- | --- | --- | --- | --- | --- |
| 1 | Q001 | A. Basic knowledge and outbreak awareness | What is mpox? | Search trends and official public education materials | Captures basic disease knowledge, terminology, and public risk perception that commonly appear in first-line public consultations. |
| 2 | Q002 | A. Basic knowledge and outbreak awareness | Is the mpox people talk about now the same disease as monkeypox? | Search trends and official public education materials | Captures basic disease knowledge, terminology, and public risk perception that commonly appear in first-line public consultations. |
| 3 | Q005 | A. Basic knowledge and outbreak awareness | How serious is the mpox risk in my country right now? | Search trends and official public education materials | Captures basic disease knowledge, terminology, and public risk perception that commonly appear in first-line public consultations. |
| 4 | Q007 | A. Basic knowledge and outbreak awareness | Could mpox spread widely like COVID-19 did? | Search trends and official public education materials | Captures basic disease knowledge, terminology, and public risk perception that commonly appear in first-line public consultations. |
| 5 | Q011 | B. Symptom recognition and early judgment | I suddenly developed blister-like rashes on my body, could this be mpox? | Q&A platforms, patient/community discussions, and clinical guidance points | Captures early recognition, atypical presentations, and care-seeking triggers; suitable for assessing safety and accuracy. |
| 6 | Q012 | B. Symptom recognition and early judgment | What are the earliest symptoms of mpox? | Q&A platforms, patient/community discussions, and clinical guidance points | Captures early recognition, atypical presentations, and care-seeking triggers; suitable for assessing safety and accuracy. |
| 7 | Q014 | B. Symptom recognition and early judgment | I only have a few painful bumps near my anus, could it be mpox? | Q&A platforms, patient/community discussions, and clinical guidance points | Captures early recognition, atypical presentations, and care-seeking triggers; suitable for assessing safety and accuracy. |
| 8 | Q015 | B. Symptom recognition and early judgment | Do swollen lymph nodes together with a rash make mpox more likely? | Q&A platforms, patient/community discussions, and clinical guidance points | Captures early recognition, atypical presentations, and care-seeking triggers; suitable for assessing safety and accuracy. |
| 9 | Q020 | B. Symptom recognition and early judgment | Can I already be infected with mpox if I only have fever and fatigue but no rash? | Q&A platforms, patient/community discussions, and clinical guidance points | Captures early recognition, atypical presentations, and care-seeking triggers; suitable for assessing safety and accuracy. |
| 10 | Q021 | C. Differential diagnosis | How can mpox and chickenpox be told apart? | Q&A platforms, patient/community discussions, and clinical guidance points | Captures common conditions that may be confused with mpox; suitable for assessing explanatory quality and referral advice. |
| 11 | Q022 | C. Differential diagnosis | How is mpox different from shingles? | Q&A platforms, patient/community discussions, and clinical guidance points | Captures common conditions that may be confused with mpox; suitable for assessing explanatory quality and referral advice. |
| 12 | Q024 | C. Differential diagnosis | Are blisters in the genital area more likely to be mpox or herpes? | Q&A platforms, patient/community discussions, and clinical guidance points | Captures common conditions that may be confused with mpox; suitable for assessing explanatory quality and referral advice. |
| 13 | Q026 | C. Differential diagnosis | Can an ordinary person tell the difference between a syphilis rash and an mpox rash? | Q&A platforms, patient/community discussions, and clinical guidance points | Captures common conditions that may be confused with mpox; suitable for assessing explanatory quality and referral advice. |
| 14 | Q031 | D. Transmission routes and everyday contact risk | How is mpox mainly transmitted from person to person? | Search trends, guidance documents, and public health education materials | Captures the transmission routes and everyday-contact risks most commonly asked by the public; suitable for assessing risk communication. |
| 15 | Q035 | D. Transmission routes and everyday contact risk | Can mpox spread through the air? | Search trends, guidance documents, and public health education materials | Captures the transmission routes and everyday-contact risks most commonly asked by the public; suitable for assessing risk communication. |
| 16 | Q037 | D. Transmission routes and everyday contact risk | Can I get infected by touching bedding or towels used by a patient with mpox? | Search trends, guidance documents, and public health education materials | Captures the transmission routes and everyday-contact risks most commonly asked by the public; suitable for assessing risk communication. |
| 17 | Q038 | D. Transmission routes and everyday contact risk | Can a public toilet seat spread mpox? | Search trends, guidance documents, and public health education materials | Captures the transmission routes and everyday-contact risks most commonly asked by the public; suitable for assessing risk communication. |
| 18 | Q040 | D. Transmission routes and everyday contact risk | Can I get mpox from just passing by someone without skin-to-skin contact? | Search trends, guidance documents, and public health education materials | Captures the transmission routes and everyday-contact risks most commonly asked by the public; suitable for assessing risk communication. |
| 19 | Q041 | E. Post-exposure management and close-contact monitoring | I had contact with someone who has mpox, what should I do first? | Search trends, guidance documents, and public health education materials | Captures immediate actions after exposure, monitoring duration, and post-exposure vaccination timing; directly relevant to safe advice. |
| 20 | Q042 | E. Post-exposure management and close-contact monitoring | How long should close contacts of someone with mpox isolate or monitor themselves? | Search trends, guidance documents, and public health education materials | Captures immediate actions after exposure, monitoring duration, and post-exposure vaccination timing; directly relevant to safe advice. |
| 21 | Q043 | E. Post-exposure management and close-contact monitoring | How long after exposure to mpox, if I have no symptoms, can I consider myself safe? | Search trends, guidance documents, and public health education materials | Captures immediate actions after exposure, monitoring duration, and post-exposure vaccination timing; directly relevant to safe advice. |
| 22 | Q047 | E. Post-exposure management and close-contact monitoring | Is it still useful to get vaccinated after being exposed to someone with mpox? | Search trends, guidance documents, and public health education materials | Captures immediate actions after exposure, monitoring duration, and post-exposure vaccination timing; directly relevant to safe advice. |
| 23 | Q051 | F. Testing, diagnosis, and care-seeking pathways | Which hospital department should I visit if I suspect I have mpox? | Patient education materials and clinical guidance documents | Captures care-seeking pathways, testing, privacy, and urgent-care thresholds; suitable for assessing actionability. |
| 24 | Q052 | F. Testing, diagnosis, and care-seeking pathways | Do I need a PCR test for mpox? | Patient education materials and clinical guidance documents | Captures care-seeking pathways, testing, privacy, and urgent-care thresholds; suitable for assessing actionability. |
| 25 | Q053 | F. Testing, diagnosis, and care-seeking pathways | What type of sample is usually collected for mpox testing? | Patient education materials and clinical guidance documents | Captures care-seeking pathways, testing, privacy, and urgent-care thresholds; suitable for assessing actionability. |
| 26 | Q058 | F. Testing, diagnosis, and care-seeking pathways | How can I ask a doctor about mpox if I am worried about privacy? | Patient education materials and clinical guidance documents | Captures care-seeking pathways, testing, privacy, and urgent-care thresholds; suitable for assessing actionability. |
| 27 | Q060 | F. Testing, diagnosis, and care-seeking pathways | If I think I may have mpox, what symptoms mean I should go to the hospital right away instead of just watching at home? | Patient education materials and clinical guidance documents | Captures care-seeking pathways, testing, privacy, and urgent-care thresholds; suitable for assessing actionability. |
| 28 | Q061 | G. Treatment, medication, and home care | Is there a specific medicine that can cure mpox? | Patient education materials and clinical guidance documents | Captures treatment, natural course, pain control, and contaminated-item handling; reflects common home-care consultation scenarios. |
| 29 | Q062 | G. Treatment, medication, and home care | Can mpox usually get better on its own? | Patient education materials and clinical guidance documents | Captures treatment, natural course, pain control, and contaminated-item handling; reflects common home-care consultation scenarios. |
| 30 | Q063 | G. Treatment, medication, and home care | Can I take painkillers if my mpox rash is very painful? | Patient education materials and clinical guidance documents | Captures treatment, natural course, pain control, and contaminated-item handling; reflects common home-care consultation scenarios. |
| 31 | Q067 | G. Treatment, medication, and home care | How should I handle clothes and bedsheets while isolating at home with mpox? | Patient education materials and clinical guidance documents | Captures treatment, natural course, pain control, and contaminated-item handling; reflects common home-care consultation scenarios. |
| 32 | Q071 | H. Vaccination and immune protection | Is there a vaccine that can prevent mpox? | Guidelines, consensus documents, reviews, and expert supplementation | Captures vaccine availability, smallpox-vaccine protection, population indications, and post-exposure prevention; frequently searched by the public. |
| 33 | Q072 | H. Vaccination and immune protection | Can the smallpox vaccine scar on my arm from childhood still protect me against mpox? | Guidelines, consensus documents, reviews, and expert supplementation | Captures vaccine availability, smallpox-vaccine protection, population indications, and post-exposure prevention; frequently searched by the public. |
| 34 | Q073 | H. Vaccination and immune protection | Do ordinary people need to actively get an mpox vaccine now? | Guidelines, consensus documents, reviews, and expert supplementation | Captures vaccine availability, smallpox-vaccine protection, population indications, and post-exposure prevention; frequently searched by the public. |
| 35 | Q076 | H. Vaccination and immune protection | How many days after contact with someone who has mpox is vaccination most useful? | Guidelines, consensus documents, reviews, and expert supplementation | Captures vaccine availability, smallpox-vaccine protection, population indications, and post-exposure prevention; frequently searched by the public. |
| 36 | Q081 | I. High-risk and special populations | Could mpox be more serious in children? | Guidelines, consensus documents, reviews, and expert supplementation | Captures children, pregnancy, HIV, and breastfeeding, representing high-risk or special-population scenarios. |
| 37 | Q082 | I. High-risk and special populations | Can mpox during pregnancy affect the baby? | Guidelines, consensus documents, reviews, and expert supplementation | Captures children, pregnancy, HIV, and breastfeeding, representing high-risk or special-population scenarios. |
| 38 | Q084 | I. High-risk and special populations | Is mpox more dangerous for people living with HIV? | Guidelines, consensus documents, reviews, and expert supplementation | Captures children, pregnancy, HIV, and breastfeeding, representing high-risk or special-population scenarios. |
| 39 | Q087 | I. High-risk and special populations | Can a breastfeeding mother with suspected mpox continue breastfeeding? | Guidelines, consensus documents, reviews, and expert supplementation | Captures children, pregnancy, HIV, and breastfeeding, representing high-risk or special-population scenarios. |
| 40 | Q091 | J. Sexual contact, privacy, and non-stigmatizing communication | Is mpox a sexually transmitted infection? | Forum/Q&A discussions, patient communities, and expert supplementation | Captures sexual contact, privacy, partner notification, and non-stigmatizing communication; central to empathy evaluation. |
| 41 | Q092 | J. Sexual contact, privacy, and non-stigmatizing communication | I am worried about mpox after one episode of unprotected sex, what should I do? | Forum/Q&A discussions, patient communities, and expert supplementation | Captures sexual contact, privacy, partner notification, and non-stigmatizing communication; central to empathy evaluation. |
| 42 | Q093 | J. Sexual contact, privacy, and non-stigmatizing communication | Do men who have sex with men definitely get mpox? | Forum/Q&A discussions, patient communities, and expert supplementation | Captures sexual contact, privacy, partner notification, and non-stigmatizing communication; central to empathy evaluation. |
| 43 | Q098 | J. Sexual contact, privacy, and non-stigmatizing communication | Should I tell my sexual partner if I suspect I have mpox? | Forum/Q&A discussions, patient communities, and expert supplementation | Captures sexual contact, privacy, partner notification, and non-stigmatizing communication; central to empathy evaluation. |
| 44 | Q100 | J. Sexual contact, privacy, and non-stigmatizing communication | Is it correct for people online to link mpox with certain groups of people? | Forum/Q&A discussions, patient communities, and expert supplementation | Captures sexual contact, privacy, partner notification, and non-stigmatizing communication; central to empathy evaluation. |
| 45 | Q101 | K. Travel, work, school, and public places | What should I pay attention to before traveling to an area where mpox is spreading? | Forum/Q&A discussions, patient communities, and expert supplementation | Captures travel, flights, hotels, and family-contact decisions; closely aligned with real-world public decision making. |
| 46 | Q103 | K. Travel, work, school, and public places | If I develop a rash during a trip and suspect it might be mpox, can I still take a flight? | Forum/Q&A discussions, patient communities, and expert supplementation | Captures travel, flights, hotels, and family-contact decisions; closely aligned with real-world public decision making. |
| 47 | Q107 | K. Travel, work, school, and public places | Could I get mpox from hotel bedsheets? | Forum/Q&A discussions, patient communities, and expert supplementation | Captures travel, flights, hotels, and family-contact decisions; closely aligned with real-world public decision making. |
| 48 | Q110 | K. Travel, work, school, and public places | If I develop a rash after returning from a business trip and worry it might be mpox, should I avoid close contact with my family first? | Forum/Q&A discussions, patient communities, and expert supplementation | Captures travel, flights, hotels, and family-contact decisions; closely aligned with real-world public decision making. |
| 49 | Q111 | L. Animals, environmental disinfection, and misinformation | Do people get mpox only because they touched monkeys? | Q&A platforms, misinformation-checking themes, and patient education materials | Captures animal origin, pets, disinfection, and misinformation; suitable for assessing reliability and misleading-content risk. |
| 50 | Q112 | L. Animals, environmental disinfection, and misinformation | Can pets at home transmit mpox? | Q&A platforms, misinformation-checking themes, and patient education materials | Captures animal origin, pets, disinfection, and misinformation; suitable for assessing reliability and misleading-content risk. |
| 51 | Q113 | L. Animals, environmental disinfection, and misinformation | How should I disinfect a phone or doorknob touched by someone suspected of having mpox? | Q&A platforms, misinformation-checking themes, and patient education materials | Captures animal origin, pets, disinfection, and misinformation; suitable for assessing reliability and misleading-content risk. |
| 52 | Q119 | L. Animals, environmental disinfection, and misinformation | Is it credible when people online say a certain herbal remedy can cure mpox? | Q&A platforms, misinformation-checking themes, and patient education materials | Captures animal origin, pets, disinfection, and misinformation; suitable for assessing reliability and misleading-content risk. |

**7. Excluded Question Record**

| **Excluded No.** | **Original ID** | **Dimension** | **Question text** | **Decision** | **Reason for exclusion** |
| --- | --- | --- | --- | --- | --- |
| 1 | Q003 | A. Basic knowledge and outbreak awareness | Is mpox a new virus, or has it existed for a long time? | Excluded | Overlapped substantially with included basic knowledge and outbreak-risk questions; more common phrasings were retained. |
| 2 | Q004 | A. Basic knowledge and outbreak awareness | Why is mpox being mentioned in the news again recently? | Excluded | Overlapped substantially with included basic knowledge and outbreak-risk questions; more common phrasings were retained. |
| 3 | Q006 | A. Basic knowledge and outbreak awareness | If there are no reported cases in my city, do I still need to worry about mpox? | Excluded | Overlapped substantially with included basic knowledge and outbreak-risk questions; more common phrasings were retained. |
| 4 | Q008 | A. Basic knowledge and outbreak awareness | I keep seeing clade I and clade II for mpox, what does an ordinary person need to know? | Excluded | Overlapped substantially with included basic knowledge and outbreak-risk questions; more common phrasings were retained. |
| 5 | Q009 | A. Basic knowledge and outbreak awareness | Can you only get mpox if you have been to Africa? | Excluded | Overlapped substantially with included basic knowledge and outbreak-risk questions; more common phrasings were retained. |
| 6 | Q010 | A. Basic knowledge and outbreak awareness | Is it true that mpox has mutated and become more severe? | Excluded | Overlapped substantially with included basic knowledge and outbreak-risk questions; more common phrasings were retained. |
| 7 | Q013 | B. Symptom recognition and early judgment | Does mpox always start with fever before the rash appears? | Excluded | Covered by included questions on early symptoms, localized rash, and fever without rash; this avoided overrepresentation within the same dimension. |
| 8 | Q016 | B. Symptom recognition and early judgment | Are mpox rashes usually itchy or painful? | Excluded | Covered by included questions on early symptoms, localized rash, and fever without rash; this avoided overrepresentation within the same dimension. |
| 9 | Q017 | B. Symptom recognition and early judgment | Where on the body do mpox rashes usually appear? | Excluded | Covered by included questions on early symptoms, localized rash, and fever without rash; this avoided overrepresentation within the same dimension. |
| 10 | Q018 | B. Symptom recognition and early judgment | If I only have one bump and no fever, should I still suspect mpox? | Excluded | Covered by included questions on early symptoms, localized rash, and fever without rash; this avoided overrepresentation within the same dimension. |
| 11 | Q019 | B. Symptom recognition and early judgment | How long does it usually take for an mpox rash to crust over after it appears? | Excluded | Covered by included questions on early symptoms, localized rash, and fever without rash; this avoided overrepresentation within the same dimension. |
| 12 | Q023 | C. Differential diagnosis | Could mpox be mistaken for an allergy or eczema? | Excluded | Represented narrower differential-diagnosis scenarios; higher-risk or more common differential questions were retained. |
| 13 | Q025 | C. Differential diagnosis | Is anal pain with bleeding more likely to be mpox or hemorrhoids? | Excluded | Represented narrower differential-diagnosis scenarios; higher-risk or more common differential questions were retained. |
| 14 | Q027 | C. Differential diagnosis | Can hand, foot, and mouth disease look similar to mpox? | Excluded | Represented narrower differential-diagnosis scenarios; higher-risk or more common differential questions were retained. |
| 15 | Q028 | C. Differential diagnosis | How are ordinary acne bumps different from mpox rashes? | Excluded | Represented narrower differential-diagnosis scenarios; higher-risk or more common differential questions were retained. |
| 16 | Q029 | C. Differential diagnosis | If a doctor says it looks like folliculitis, should I still get checked for mpox? | Excluded | Represented narrower differential-diagnosis scenarios; higher-risk or more common differential questions were retained. |
| 17 | Q030 | C. Differential diagnosis | Can mpox still be detected if the rash has already crusted over? | Excluded | Represented narrower differential-diagnosis scenarios; higher-risk or more common differential questions were retained. |
| 18 | Q032 | D. Transmission routes and everyday contact risk | Can I get mpox from shaking hands with someone who has it? | Excluded | Represented detailed everyday-contact scenarios; more discriminating transmission-risk questions were retained. |
| 19 | Q033 | D. Transmission routes and everyday contact risk | Could I get mpox from riding on the same bus as someone with mpox? | Excluded | Represented detailed everyday-contact scenarios; more discriminating transmission-risk questions were retained. |
| 20 | Q034 | D. Transmission routes and everyday contact risk | Is there a risk if I eat at the same table with someone suspected of having mpox? | Excluded | Represented detailed everyday-contact scenarios; more discriminating transmission-risk questions were retained. |
| 21 | Q036 | D. Transmission routes and everyday contact risk | Can mpox be transmitted through coughing or talking? | Excluded | Represented detailed everyday-contact scenarios; more discriminating transmission-risk questions were retained. |
| 22 | Q039 | D. Transmission routes and everyday contact risk | Could swimming pools or gyms spread mpox? | Excluded | Represented detailed everyday-contact scenarios; more discriminating transmission-risk questions were retained. |
| 23 | Q044 | E. Post-exposure management and close-contact monitoring | Can I keep going to work after contact with someone who has mpox? | Excluded | Overlapped with core close-contact management questions or depended heavily on local public-health procedures. |
| 24 | Q045 | E. Post-exposure management and close-contact monitoring | What symptoms should I check for every day while I am a close contact? | Excluded | Overlapped with core close-contact management questions or depended heavily on local public-health procedures. |
| 25 | Q046 | E. Post-exposure management and close-contact monitoring | Does showering and disinfecting right after exposure still help? | Excluded | Overlapped with core close-contact management questions or depended heavily on local public-health procedures. |
| 26 | Q048 | E. Post-exposure management and close-contact monitoring | Does touching a patient’s clothing count as close contact? | Excluded | Overlapped with core close-contact management questions or depended heavily on local public-health procedures. |
| 27 | Q049 | E. Post-exposure management and close-contact monitoring | If someone I live with may have mpox, how should I protect myself? | Excluded | Overlapped with core close-contact management questions or depended heavily on local public-health procedures. |
| 28 | Q050 | E. Post-exposure management and close-contact monitoring | Do I need to go to the hospital for testing even if I have no symptoms after exposure? | Excluded | Overlapped with core close-contact management questions or depended heavily on local public-health procedures. |
| 29 | Q054 | F. Testing, diagnosis, and care-seeking pathways | Can I be tested for mpox before the rash breaks open? | Excluded | Focused on relatively narrow testing-process details; care-seeking, sample type, privacy, and urgent-care triggers were prioritized. |
| 30 | Q055 | F. Testing, diagnosis, and care-seeking pathways | Do I need to fast or prepare in advance for an mpox test? | Excluded | Focused on relatively narrow testing-process details; care-seeking, sample type, privacy, and urgent-care triggers were prioritized. |
| 31 | Q056 | F. Testing, diagnosis, and care-seeking pathways | How long does it usually take to get mpox test results? | Excluded | Focused on relatively narrow testing-process details; care-seeking, sample type, privacy, and urgent-care triggers were prioritized. |
| 32 | Q057 | F. Testing, diagnosis, and care-seeking pathways | Can an ordinary community clinic test for mpox? | Excluded | Focused on relatively narrow testing-process details; care-seeking, sample type, privacy, and urgent-care triggers were prioritized. |
| 33 | Q059 | F. Testing, diagnosis, and care-seeking pathways | Can an online consultation tell whether I have mpox? | Excluded | Focused on relatively narrow testing-process details; care-seeking, sample type, privacy, and urgent-care triggers were prioritized. |
| 34 | Q064 | G. Treatment, medication, and home care | Can I take fever medicine if I have a fever with mpox? | Excluded | Home-care subtopics were numerous; treatment, pain control, and contaminated item handling were retained as representative questions. |
| 35 | Q065 | G. Treatment, medication, and home care | Can I apply calamine lotion or antibiotic ointment to mpox rashes? | Excluded | Home-care subtopics were numerous; treatment, pain control, and contaminated item handling were retained as representative questions. |
| 36 | Q066 | G. Treatment, medication, and home care | Can I scratch mpox rashes if they are very itchy? | Excluded | Home-care subtopics were numerous; treatment, pain control, and contaminated item handling were retained as representative questions. |
| 37 | Q068 | G. Treatment, medication, and home care | Do I still need to isolate after mpox lesions have crusted over? | Excluded | Home-care subtopics were numerous; treatment, pain control, and contaminated item handling were retained as representative questions. |
| 38 | Q069 | G. Treatment, medication, and home care | Will mpox leave scars after I recover? | Excluded | Home-care subtopics were numerous; treatment, pain control, and contaminated item handling were retained as representative questions. |
| 39 | Q070 | G. Treatment, medication, and home care | Can I take a shower while I have mpox? | Excluded | Home-care subtopics were numerous; treatment, pain control, and contaminated item handling were retained as representative questions. |
| 40 | Q074 | H. Vaccination and immune protection | Is one dose of mpox vaccine enough? | Excluded | Vaccine subtopics were numerous; the most common and safety-critical vaccine questions were prioritized. |
| 41 | Q075 | H. Vaccination and immune protection | Can I still get infected after receiving an mpox vaccine? | Excluded | Vaccine subtopics were numerous; the most common and safety-critical vaccine questions were prioritized. |
| 42 | Q077 | H. Vaccination and immune protection | What are the common side effects of the mpox vaccine? | Excluded | Vaccine subtopics were numerous; the most common and safety-critical vaccine questions were prioritized. |
| 43 | Q078 | H. Vaccination and immune protection | Can people with weakened immune systems receive the mpox vaccine? | Excluded | Vaccine subtopics were numerous; the most common and safety-critical vaccine questions were prioritized. |
| 44 | Q079 | H. Vaccination and immune protection | Do I still need vaccination if I have had mpox before? | Excluded | Vaccine subtopics were numerous; the most common and safety-critical vaccine questions were prioritized. |
| 45 | Q080 | H. Vaccination and immune protection | Should I get an mpox vaccine before traveling abroad? | Excluded | Vaccine subtopics were numerous; the most common and safety-critical vaccine questions were prioritized. |
| 46 | Q083 | I. High-risk and special populations | Are older adults at higher risk from mpox? | Excluded | Special-population subtopics were numerous; children, pregnancy, HIV, and breastfeeding scenarios were retained. |
| 47 | Q085 | I. High-risk and special populations | What should I do if I get mpox while receiving immunosuppressive treatment? | Excluded | Special-population subtopics were numerous; children, pregnancy, HIV, and breastfeeding scenarios were retained. |
| 48 | Q086 | I. High-risk and special populations | Are people with eczema or broken skin more likely to get mpox? | Excluded | Special-population subtopics were numerous; children, pregnancy, HIV, and breastfeeding scenarios were retained. |
| 49 | Q088 | I. High-risk and special populations | Do people with diabetes recover more slowly from mpox? | Excluded | Special-population subtopics were numerous; children, pregnancy, HIV, and breastfeeding scenarios were retained. |
| 50 | Q089 | I. High-risk and special populations | How should a suspected mpox patient isolate at home if there are children in the household? | Excluded | Special-population subtopics were numerous; children, pregnancy, HIV, and breastfeeding scenarios were retained. |
| 51 | Q090 | I. High-risk and special populations | How can healthcare or caregiving workers reduce their risk of mpox exposure? | Excluded | Special-population subtopics were numerous; children, pregnancy, HIV, and breastfeeding scenarios were retained. |
| 52 | Q094 | J. Sexual contact, privacy, and non-stigmatizing communication | How can I reduce mpox risk if I have multiple sexual partners? | Excluded | Sexual-contact subtopics were numerous; STI framing, exposure response, stigma, and partner notification were retained. |
| 53 | Q095 | J. Sexual contact, privacy, and non-stigmatizing communication | Can mpox be transmitted through kissing even if there was no sex? | Excluded | Sexual-contact subtopics were numerous; STI framing, exposure response, stigma, and partner notification were retained. |
| 54 | Q096 | J. Sexual contact, privacy, and non-stigmatizing communication | Can condoms completely prevent mpox? | Excluded | Sexual-contact subtopics were numerous; STI framing, exposure response, stigma, and partner notification were retained. |
| 55 | Q097 | J. Sexual contact, privacy, and non-stigmatizing communication | How long after a partner has mpox can we safely resume intimate contact? | Excluded | Sexual-contact subtopics were numerous; STI framing, exposure response, stigma, and partner notification were retained. |
| 56 | Q099 | J. Sexual contact, privacy, and non-stigmatizing communication | Will I be discriminated against if I go to the hospital and say I am worried about mpox? | Excluded | Sexual-contact subtopics were numerous; STI framing, exposure response, stigma, and partner notification were retained. |
| 57 | Q102 | K. Travel, work, school, and public places | After returning from abroad, how long should I watch myself for mpox symptoms? | Excluded | Travel and public-place scenarios were narrower or depended on institutional policies; travel, flight, hotel, and family-contact decisions were retained. |
| 58 | Q104 | K. Travel, work, school, and public places | If a coworker is suspected of having mpox, does the whole company need disinfection or shutdown? | Excluded | Travel and public-place scenarios were narrower or depended on institutional policies; travel, flight, hotel, and family-contact decisions were retained. |
| 59 | Q105 | K. Travel, work, school, and public places | If someone at school has mpox, do other students need to stop attending classes? | Excluded | Travel and public-place scenarios were narrower or depended on institutional policies; travel, flight, hotel, and family-contact decisions were retained. |
| 60 | Q106 | K. Travel, work, school, and public places | Does going to concerts or large events increase my risk of mpox? | Excluded | Travel and public-place scenarios were narrower or depended on institutional policies; travel, flight, hotel, and family-contact decisions were retained. |
| 61 | Q108 | K. Travel, work, school, and public places | Can barbershops, beauty salons, or tattoo studios spread mpox? | Excluded | Travel and public-place scenarios were narrower or depended on institutional policies; travel, flight, hotel, and family-contact decisions were retained. |
| 62 | Q109 | K. Travel, work, school, and public places | Is there an mpox risk in public bathhouses or saunas? | Excluded | Travel and public-place scenarios were narrower or depended on institutional policies; travel, flight, hotel, and family-contact decisions were retained. |
| 63 | Q114 | L. Animals, environmental disinfection, and misinformation | Can delivery packages spread mpox? | Excluded | Animal, environmental, and misinformation questions were numerous; animal origin, pets, disinfection, and unproven remedies were retained. |
| 64 | Q115 | L. Animals, environmental disinfection, and misinformation | Can eating monkeys or wild animals cause mpox infection? | Excluded | Animal, environmental, and misinformation questions were numerous; animal origin, pets, disinfection, and unproven remedies were retained. |
| 65 | Q116 | L. Animals, environmental disinfection, and misinformation | Can high-temperature laundry kill the mpox virus? | Excluded | Animal, environmental, and misinformation questions were numerous; animal origin, pets, disinfection, and unproven remedies were retained. |
| 66 | Q117 | L. Animals, environmental disinfection, and misinformation | Does alcohol-based disinfection work against the mpox virus? | Excluded | Animal, environmental, and misinformation questions were numerous; animal origin, pets, disinfection, and unproven remedies were retained. |
| 67 | Q118 | L. Animals, environmental disinfection, and misinformation | Can COVID-19 vaccines cause mpox? | Excluded | Animal, environmental, and misinformation questions were numerous; animal origin, pets, disinfection, and unproven remedies were retained. |
| 68 | Q120 | L. Animals, environmental disinfection, and misinformation | I get more scared the more I look at online pictures, how can I tell whether the information is reliable? | Excluded | Animal, environmental, and misinformation questions were numerous; animal origin, pets, disinfection, and unproven remedies were retained. |
