# Supplementary material for: Evaluating search-enabled large language model interfaces for mpox public health consultation: a guideline-based comparative study
Source: Front Public Health. 2026 Aug 26;14:1913260. doi: 10.3389/fpubh.2026.1913260 (PMC13553459; doi:10.3389/fpubh.2026.1913260)
Supplement: Supplementary file 2 [file Supplementary_file_2.DOCX]

**Mpox Public Q&A–Guideline Content Mapping Table**

Purpose: This document maps each public-facing mpox question to a concise answer, the corresponding guideline content, and the source type that should be used as the evidence basis.

Recommended evidence hierarchy: use formal guidelines and expert consensus as the primary answer basis; use public science/health education materials only for wording; use systematic/scoping reviews only as supplementary epidemiology or evidence context, not as standalone recommendations.

Important limitation: current local risk, vaccine eligibility, and travel restrictions change over time and must be checked against live public-health notices. This file is a mapping framework, not a substitute for local clinical advice.

**1. Source code key and use classification**

| **Code** | **Source** | **Type** | **Use in mapping** |
| --- | --- | --- | --- |
| S1 | WHO. Clinical management and infection prevention and control for mpox: living guideline. | Formal living clinical/IPC guideline | Use as primary basis for clinical severity, IPC, pregnancy, breastfeeding, respectful/privacy-preserving care. Identifier in upload: WHO/B09434; no PMID/DOI identified in the PDF. |
| S2 | National Health Commission / National Administration of Traditional Chinese Medicine, China. Monkeypox Diagnosis and Treatment Guideline (2022 version) / China CDC repost. | Formal national diagnosis and treatment guideline | National Health Commission of the People’s Republic of China and National Administration of Traditional Chinese Medicine. Diagnosis and Treatment Guideline for Monkeypox (2022 Edition). Formal national diagnosis and treatment guideline. Official government document; no DOI assigned. |
| S3 | National Health Commission, China. Technical Guidelines for Monkeypox Control (2022 version). | Formal public-health prevention and control guideline | Use as primary basis for case reporting, epidemiological investigation, close-contact tracing/monitoring, disinfection, and public-health management. DOI in uploaded journal version: 10.16505/j.2095-0136.2022.0050. |
| S4 | National Medical Center for Infectious Diseases / Chinese Preventive Medicine Association. Mpox Prevention Guideline for the Public (2023). | Public guideline | Use as main public-facing source for daily-life risk, non-stigmatizing communication, care seeking, travel, and self-protection. DOI: 10.3760/cma.j.cn311365-20230616-00186. |
| S5 | CDC/MMWR. Interim Clinical Treatment Considerations for Severe Manifestations of Mpox — United States, February 2023. | Clinical treatment considerations/guidance article | Use as primary source for severe manifestations, hospitalization triggers, and therapeutic medical countermeasures. PMID: 36862595; DOI: 10.15585/mmwr.mm7209a4. |
| S6 | ACIP/CDC/MMWR. Use of JYNNEOS vaccine for orthopoxvirus occupational exposure (2022) and use during mpox outbreaks (2025). | Vaccine recommendations | Use as primary basis for vaccine eligibility, JYNNEOS schedule, special populations, and outbreak-specific vaccination policy. 2022 PMID: 35653347; DOI: 10.15585/mmwr.mm7122e1. 2025 PMID: 40531798; DOI: 10.15585/mmwr.mm7422a3. |
| S7 | Jiang et al. Diagnosis, treatment, and prevention of monkeypox in children: an experts’ consensus statement. | Pediatric expert consensus | Use as primary basis for children/adolescents, pediatric testing, isolation, monitoring, and prevention. DOI: 10.1007/s12519-022-00624-3. |
| S8 | HIV-infected persons with mpox: expert consensus on diagnosis and treatment. | Disease-specific expert consensus | Use as primary basis for HIV, advanced immunosuppression, ART coordination, and risk of severe/prolonged disease. DOI: 10.3760/cma.j.issn.1674-2397.2023.04.001. |
| S9 | Expert consensus on healthcare-associated infection prevention and control of mpox. | Healthcare IPC expert consensus | Use as primary basis for hospital triage, PPE, patient isolation, environmental cleaning, and occupational exposure. DOI: 10.3760/cma.j.cn112137-20230723-00075. |
| S10 | A Primer on Monkeypox Virus for Obstetrician–Gynecologists; plus WHO pregnancy/postpartum sections. | Supplementary specialty context | Use as support for pregnancy and obstetric care when formal guideline statements are not detailed enough. DOI for primer: 10.1097/AOG.0000000000004909. |
| S11 | Guideline on integrated traditional Chinese and Western medicine diagnosis and treatment of mpox (2025 version). | Clinical practice guideline | Use only for jurisdiction-specific adjunctive TCM content; do not frame any single herbal remedy as a proven cure. DOI: 10.15932/j.0253-9713.2025.10.001. |
| E1 | Webb et al. Availability, scope and quality of monkeypox clinical management guidelines globally: a systematic review. | Systematic review/evidence supplement | Use only as background on quality/scope of guidelines, not as a direct recommendation source. DOI: 10.1136/bmjgh-2022-009838. |
| E2 | Risk factors associated with human Mpox infection: a systematic review and meta-analysis. | Systematic review/evidence supplement | Use to support risk-factor framing and surveillance context, not as direct medical advice. DOI: 10.1136/bmjgh-2024-016937. |
| E3 | Mpox current knowledge and understanding: a scoping review. | Scoping review/evidence supplement | Use for rapidly evolving background, current knowledge gaps, vaccine effectiveness overview, and clade context, not as direct recommendation. DOI: 10.1093/femsre/fuaf025. |

Mapping principles

Primary evidence field: formal guidelines, national guidance, WHO/CDC/ACIP recommendations, and expert consensus.

Supplementary field: systematic reviews, scoping reviews, or narrative reviews can support background, risk-factor framing, or evidence gaps, but should not override formal guidance.

Public wording field: use plain language, avoid stigma, and distinguish “possible” from “likely” risk.

Localisation field: when the question asks about current national risk, vaccine access, travel restrictions, or designated clinics, the answer must be updated with live local authority information.

**2. Question–answer–guideline mapping table**

A. Basic knowledge and outbreak awareness

| **ID** | **Public question** | **Suggested public answer** | **Mapped guideline content / rationale** | **Source classification** |
| --- | --- | --- | --- | --- |
| Q1 | What is mpox? | Mpox is a contagious disease caused by monkeypox virus, an orthopoxvirus. It can cause fever, swollen lymph nodes, fatigue, and a rash or lesions that may appear on the skin or mucosal areas, including the mouth, genital, or anal region. Most mild cases recover with supportive care, but medical evaluation is important when symptoms follow a relevant exposure or when severe symptoms, high-risk conditions, or special populations are involved. | Guidelines define mpox as an orthopoxvirus infection with systemic symptoms and rash; diagnosis is based on clinical features plus epidemiological history and laboratory confirmation when indicated. The answer should clearly separate mpox from nonspecific acne-like lesions. | Primary: S1, S2, S4. Supplement: E3. |
| Q2 | Is the mpox people talk about now the same disease as monkeypox? | Yes. “Mpox” is the current preferred name for the disease previously called monkeypox. The virus name and older document titles may still use “monkeypox,” but the public answer should use mpox and explain that they refer to the same disease. | WHO and subsequent guidance use mpox to reduce stigma while many older clinical and public-health documents retain “monkeypox.” Map both terms to the same disease entity in the answer base. | Primary: S1, S2, S3, S4. |
| Q3 | How serious is the mpox risk in my country right now? | This cannot be answered from a static guideline alone. The current risk depends on local case numbers, clade, exposure networks, vaccination availability, and public-health alerts. For an individual, risk is higher after close skin-to-skin/sexual contact with a case, household exposure, or contact with contaminated materials. | Guidelines give risk-factor and exposure-based advice, but real-time national risk requires current surveillance reports. The table should flag this as “guideline + live surveillance needed,” not a fixed answer. | Primary: S3, S4, S6. Supplement: E2, E3. Use live national/WHO surveillance for updates. |
| Q4 | Could mpox spread widely like COVID-19 did? | Wide outbreaks can occur, but mpox does not usually spread like COVID-19. Mpox transmission usually requires close physical, intimate, household, or prolonged face-to-face contact, rather than routine brief airborne exposure. | Guidelines emphasize contact, sexual/intimate exposure, respiratory droplets in close/prolonged contact, and contaminated materials. Public wording should avoid over-reassurance but explain that the transmission pattern differs from COVID-19. | Primary: S1, S3, S4, S9. Supplement: E2, E3. |

B. Symptom recognition and early judgment

| **ID** | **Public question** | **Suggested public answer** | **Mapped guideline content/rationale** | **Source classification** |
| --- | --- | --- | --- | --- |
| Q5 | I suddenly developed blister-like rashes on my body, could this be mpox? | It could be mpox, but many other conditions can cause blisters or rashes. Mpox is more concerning if the rash is painful, deep-seated, vesicular or pustular, associated with fever or lymph-node swelling, or follows close/sexual/household exposure. Medical evaluation and testing may be needed. | Diagnostic guidance links suspected cases to compatible rash plus exposure history and/or systemic symptoms. The public answer should encourage care seeking and avoid visual self-diagnosis only. | Primary: S1, S2, S4, S7. |
| Q6 | What are the earliest symptoms of mpox? | Early symptoms may include fever, chills, headache, muscle aches, back pain, fatigue, sore throat, and swollen lymph nodes. A rash may follow, but in some recent cases rash can appear before or without obvious fever. | Guidelines describe a prodromal phase and rash, while recent outbreak descriptions note atypical/localized presentations. Map to “symptoms vary; lymphadenopathy and rash are important clues.” | Primary: S1, S2, S4, S7. Supplement: E3. |
| Q7 | I only have a few painful bumps near my anus, could it be mpox? | Yes, mpox can present with only a few painful genital, anal, or perianal lesions, especially after sexual or close intimate contact. Other sexually transmitted infections can look similar, so clinical assessment and testing are needed. | Guidelines and clinical considerations include mucosal, genital, rectal, and urethral lesions and proctitis. Map the answer to “localized lesions can still be suspected mpox.” | Primary: S1, S2, S4, S5. |
| Q8 | Do swollen lymph nodes together with a rash make mpox more likely? | Yes. Swollen lymph nodes with a compatible rash can make mpox more likely, although it is not diagnostic by itself. It should prompt assessment of exposure history and possible PCR testing. | Guidelines list fever, rash, and lymphadenopathy as major clinical features. Public answer should stress that lymphadenopathy supports suspicion but cannot confirm infection alone. | Primary: S1, S2, S4, S7. |
| Q9 | Can I already be infected with mpox if I only have fever and fatigue but no rash? | It is possible but nonspecific. Fever and fatigue alone are common in many illnesses. If you had a relevant exposure, monitor for rash or mucosal lesions for 21 days and contact a health professional if symptoms develop. | Guidelines describe incubation/prodrome and close-contact monitoring. Testing is usually most informative when lesions are present, so the answer should recommend monitoring and early consultation after exposure. | Primary: S2, S3, S4, S7. |

C. Differential diagnosis

| **ID** | **Public question** | **Suggested public answer** | **Mapped guideline content/rationale** | **Source classification** |
| --- | --- | --- | --- | --- |
| Q10 | How can mpox and chickenpox be told apart? | They can overlap. Mpox lesions are often deep-seated, firm, painful, umbilicated, and may be at a similar stage in one area; lymph-node swelling is common. Chickenpox is often itchier, more superficial, appears in crops at different stages, and often starts on the trunk. Testing may still be required. | Diagnostic guidelines list chickenpox/varicella as an important differential diagnosis and include clinical pattern, exposure history, and laboratory testing in the diagnostic pathway. | Primary: S2, S4, S7. |
| Q11 | How is mpox different from shingles? | Shingles usually causes a painful rash on one side of the body in a nerve distribution and is caused by varicella-zoster virus reactivation. Mpox is an orthopoxvirus infection that may involve genital, anal, oral, face, palm/sole, or generalized lesions and may follow close exposure. | Guidelines list herpes zoster as a differential diagnosis. Map to “do not rely on appearance alone; distribution, exposure history, and testing guide diagnosis.” | Primary: S2, S4, S7. |
| Q12 | Are blisters in the genital area more likely to be mpox or herpes? | Both are possible. Herpes often causes recurrent grouped painful vesicles/ulcers, while mpox lesions may be firm/deep and accompanied by lymph-node swelling or systemic symptoms. A clinician may test for mpox and other STIs at the same visit. | Guidelines include herpes simplex and other STIs in differential diagnosis and recommend laboratory confirmation for suspected mpox. Public answer should normalize STI co-testing. | Primary: S2, S4, S5, S7. |
| Q13 | Can an ordinary person tell the difference between a syphilis rash and an mpox rash? | Usually not reliably. Syphilis, herpes, chickenpox, shingles, bacterial skin infection, allergic reactions, and mpox can overlap. Professional evaluation and targeted tests are safer than self-diagnosis. | Guidelines require differential diagnosis and note that diagnosis should not be made from rash appearance alone. Map to “seek care and test when risk/symptoms fit.” | Primary: S2, S4, S7. |

D. Transmission routes and everyday contact risk

| **ID** | **Public question** | **Suggested public answer** | **Mapped guideline content/rationale** | **Source classification** |
| --- | --- | --- | --- | --- |
| Q14 | How is mpox mainly transmitted from person to person? | Main routes are direct skin-to-skin or mucosal contact with lesions or body fluids, close/intimate or sexual contact, contact with contaminated bedding/clothing/towels, and prolonged close exposure to respiratory secretions. Animal-to-human transmission can also occur in endemic settings. | Guidance consistently prioritizes close contact, lesions/body fluids, contaminated materials, and close respiratory exposure. Sexual contact is a common setting of transmission, but not the only route. | Primary: S1, S3, S4, S9. Supplement: E2, E3. |
| Q15 | Can mpox spread through the air? | Mpox is not mainly a long-range airborne disease. Respiratory droplets or secretions may contribute during close or prolonged face-to-face contact, and special precautions are needed in healthcare or aerosol-generating situations. | IPC guidance recommends precautions for close respiratory exposure and healthcare procedures while emphasizing contact transmission. Map to “not like measles/COVID airborne spread; close exposure matters.” | Primary: S1, S3, S4, S9. |
| Q16 | Can I get infected by touching bedding or towels used by a patient with mpox? | Yes, it is possible because lesion material can contaminate bedding, towels, and clothing. Avoid sharing these items, do not shake laundry, use gloves if handling, wash items appropriately, and clean hands afterward. | Guidelines list contaminated materials as a transmission route and include laundry/disinfection measures for home and healthcare settings. | Primary: S1, S3, S4, S9, S7. |
| Q17 | Can a public toilet seat spread mpox? | The risk from a public toilet seat is generally low, but contaminated surfaces are a theoretical route if fresh lesion material is present and then contacts broken skin or mucosa. Hand hygiene and routine cleaning are appropriate; panic is not. | Guidelines support fomite transmission as possible but place greatest emphasis on close/direct contact. This answer should be framed as low-probability, hygiene-focused, and non-alarmist. | Primary: S3, S4, S9. Supplement: E3. |
| Q18 | Can I get mpox from just passing by someone without skin-to-skin contact? | Briefly passing by someone is very unlikely to transmit mpox. Risk rises with close physical contact, intimate contact, household exposure, shared contaminated items, or prolonged close face-to-face exposure. | Guidelines distinguish close contacts from casual contacts. Map the answer to “brief casual proximity is low risk; close/prolonged exposure triggers monitoring advice.” | Primary: S3, S4, S9. |

E. Post-exposure management and close-contact monitoring

| **ID** | **Public question** | **Suggested public answer** | **Mapped guideline content/rationale** | **Source classification** |
| --- | --- | --- | --- | --- |
| Q19 | I had contact with someone who has mpox, what should I do first? | First, avoid further close or sexual contact, wash hands, note the date/type of exposure, and contact local public health or a healthcare provider. Ask whether you meet criteria for testing, monitoring, or post-exposure vaccination. | Control guidelines emphasize case investigation, exposure classification, close-contact registration/monitoring, self-health observation, and PEP vaccination when indicated. | Primary: S3, S4, S6, S9. |
| Q20 | How long should close contacts of someone with mpox isolate or monitor themselves? | Close contacts should monitor for symptoms for 21 days after their last exposure. Asymptomatic contacts generally do not require the same isolation as confirmed cases, but they should follow local public-health advice, avoid high-risk close or sexual contact, and seek care promptly if symptoms develop. | Guidelines use a 21-day observation/medical surveillance window for close contacts. Public answer should separate “monitoring” from “isolation,” because asymptomatic contacts are often not isolated like confirmed cases. | Primary: S3, S4, S7, S9. |
| Q21 | How long after exposure to mpox, if I have no symptoms, can I consider myself safe? | If 21 days have passed since the last exposure to mpox and you have had no symptoms, mpox from that exposure is unlikely. Seek care earlier if fever, lymph-node swelling, rash, mouth/genital/anal lesions, or eye symptoms appear. | This maps directly to the 21-day maximum monitoring period used in control guidance and pediatric/healthcare guidance. | Primary: S3, S4, S7, S9. |
| Q22 | Is it still useful to get vaccinated after being exposed to someone with mpox? | Yes, for eligible exposures it can still be useful. Vaccination is most useful as soon as possible, ideally within 4 days to help prevent disease; from 4 to 14 days it may reduce severity but may not prevent disease. | Vaccine guidance and reviews of guidance describe post-exposure vaccination timing: within 4 days preferred, up to 14 days for possible symptom reduction. Eligibility should follow local public-health criteria. | Primary: S6, S3/S4 for exposure management. Supplement: E1. |

F. Testing, diagnosis, and care-seeking pathways

| **ID** | **Public question** | **Suggested public answer** | **Mapped guideline content/rationale** | **Source classification** |
| --- | --- | --- | --- | --- |
| Q23 | Which hospital department should I visit if I suspect I have mpox? | Use the local designated pathway: infectious diseases, dermatology, fever clinic, sexual-health/STI clinic, emergency care for severe symptoms, or a public-health designated facility. Call ahead if possible so the facility can arrange privacy and infection-control measures. | Guidelines emphasize triage, isolation of suspected cases, clinical assessment, reporting, and diagnostic testing. The exact department varies by country and hospital system. | Primary: S2, S3, S4, S9. |
| Q24 | Do I need a PCR test for mpox? | If your symptoms and exposure history fit suspected mpox, PCR/NAAT testing is the standard way to confirm it. A clinician or public-health authority decides based on lesions, risk, and differential diagnosis. | Diagnostic guidelines use nucleic-acid amplification/PCR of appropriate specimens to confirm suspected cases. Public answer should not recommend testing for every nonspecific rash without risk assessment. | Primary: S1, S2, S3, S4, S7. |
| Q25 | What type of sample is usually collected for mpox testing? | Usually swabs from skin or mucosal lesions are collected, often from more than one lesion. Vesicle fluid, lesion swabs, crusts/scabs, or mucosal lesion swabs may be used. Blood is generally less useful than lesion material after rash appears. | Guidelines prioritize lesion specimens for PCR confirmation. The answer should explain collection by trained staff and avoid self-collection unless local programs allow it. | Primary: S1, S2, S3, S7. |
| Q26 | How can I ask a doctor about mpox if I am worried about privacy? | You can ask for a private conversation and say: “I may have had a close or sexual exposure and I’m worried about mpox. Can we discuss this confidentially?” Sexual history helps diagnosis and contact tracing; clinicians should protect dignity, privacy, and confidentiality. | WHO guidance explicitly supports patient-centred, respectful care that maintains dignity, privacy, and confidentiality. Public and vaccine guidance also emphasize reducing stigma and barriers to care. | Primary: S1, S4, S6, S9. |
| Q27 | If I think I may have mpox, what symptoms mean I should go to the hospital right away instead of just watching at home? | Seek urgent care for eye pain/redness/vision changes, severe or uncontrolled pain, inability to eat/drink/urinate/pass stool, dehydration, breathing trouble, confusion or neurologic symptoms, rapidly worsening or necrotic/confluent lesions, severe rectal/genital/oral involvement, or if you are pregnant, a young child, or immunocompromised. | Severe-treatment guidance lists ocular, neurologic, cardiac, respiratory, mucosal, obstructive, necrotic/confluent, septic, and immunocompromised presentations as requiring urgent evaluation or specialized care. | Primary: S1, S5, S7, S8, S10. |

G. Treatment, medication, and home care

| **ID** | **Public question** | **Suggested public answer** | **Mapped guideline content/rationale** | **Source classification** |
| --- | --- | --- | --- | --- |
| Q28 | Is there a specific medicine that can cure mpox? | Most mild cases receive supportive care. Antivirals such as tecovirimat and other medical countermeasures may be considered for severe disease, complications, or high-risk patients, but they are not a routine “guaranteed cure” for every mild case. | Clinical management guidance emphasizes supportive care, symptom control, and selective use of therapeutics for severe/high-risk disease. MMWR severe-treatment considerations highlight evidence gaps and the need for clinical judgment. | Primary: S1, S2, S5, S8. Supplement: E1. |
| Q29 | Can mpox usually get better on its own? | Many mild cases recover with supportive care over a few weeks, once lesions crust, scabs fall off, and new skin forms. Some patients can develop complications, so monitoring and infection-control precautions remain important. | Guidelines describe self-limited disease in many cases but recommend isolation/precautions until lesions have healed and new skin has formed; severe/high-risk cases need closer care. | Primary: S1, S2, S4, S5. |
| Q30 | Can I take painkillers if my mpox rash is very painful? | Pain control is appropriate and important, but use medicines according to medical advice, especially for severe rectal/genital/oral pain, pregnancy, kidney/liver disease, or other conditions. Do not apply irritating remedies or pick lesions. | Guidelines and severe-treatment guidance emphasize supportive care and pain control, including management of proctitis and mucosal lesions; severe or uncontrolled pain is a reason to seek care. | Primary: S1, S2, S5, S7. |
| Q31 | How should I handle clothes and bedsheets while isolating at home with mpox? | Keep laundry separate, avoid shaking it, cover lesions, wear disposable gloves when handling contaminated items if possible, wash with detergent according to local instructions, clean hands afterward, and disinfect high-touch surfaces. Do not share towels or bedding. | IPC and public-health guidance includes laundry, cleaning/disinfection, avoidance of shared items, lesion covering, hand hygiene, and safe handling of contaminated materials. | Primary: S1, S3, S4, S7, S9. |

H. Vaccination and immune protection

| **ID** | **Public question** | **Suggested public answer** | **Mapped guideline content/rationale** | **Source classification** |
| --- | --- | --- | --- | --- |
| Q32 | Is there a vaccine that can prevent mpox? | Yes. Vaccines developed for orthopoxviruses, especially JYNNEOS/MVA-BN in many settings, can reduce the risk of mpox. Availability, eligibility, and schedules depend on national policy and outbreak context. | ACIP guidance describes JYNNEOS as a live, nonreplicating vaccine used for occupational risk and outbreak risk groups, with a two-dose schedule in adults unless local guidance differs. | Primary: S6, S3/S4 for public-health deployment. Supplement: E3. |
| Q33 | Can the smallpox vaccine scar on my arm from childhood still protect me against mpox? | A past smallpox vaccination may provide some cross-protection, but protection can wane and a scar does not guarantee immunity. People should still follow exposure precautions and current vaccine guidance. | Orthopoxvirus vaccine guidance recognizes cross-protection, while systematic/review evidence notes waning population immunity after smallpox vaccination stopped. Public answer should avoid treating an old scar as full protection. | Primary: S6, S4. Supplement: E2, E3. |
| Q34 | Do ordinary people need to actively get an mpox vaccine now? | Not usually as a blanket recommendation. Vaccination is targeted to people with specific exposures, risk factors, occupational risks, or outbreak-linked eligibility. Local public-health authorities decide who should be vaccinated. | Vaccine recommendations are risk-based and outbreak-specific. ACIP notes public health authorities issue outbreak-specific guidance and clinicians should avoid stigmatizing eligibility processes. | Primary: S6, S4, S3. |
| Q35 | How many days after contact with someone who has mpox is vaccination most useful? | As soon as possible, ideally within 4 days after exposure. Vaccination from 4 to 14 days may still reduce symptoms, but may not prevent disease. After symptoms start, contact a clinician rather than treating vaccination as treatment. | This is a direct post-exposure prophylaxis mapping: within 4 days preferred; up to 14 days may reduce severity. Use local eligibility criteria. | Primary: S6, S3/S4. Supplement: E1. |

I. High-risk and special populations

| **ID** | **Public question** | **Suggested public answer** | **Mapped guideline content/rationale** | **Source classification** |
| --- | --- | --- | --- | --- |
| Q36 | Could mpox be more serious in children? | Yes, especially in infants, young children, and children with skin disease or immune problems. Children with suspected mpox should be evaluated promptly and protected from close contact with infected caregivers where feasible. | Pediatric consensus and WHO guidance identify children as a special population and include isolation, monitoring, supportive care, and prevention of household/healthcare spread. | Primary: S1, S7, S5. |
| Q37 | Can mpox during pregnancy affect the baby? | Yes. Mpox in pregnancy can be associated with vertical transmission and adverse outcomes such as miscarriage, stillbirth, or neonatal infection, although data are limited. Pregnant patients need coordinated obstetric, infectious-disease, fetal/newborn, and mental-health support. | WHO pregnancy guidance recommends access to skilled, respectful obstetric and neonatal care; it notes limited data suggesting vertical transmission and adverse fetal outcomes. | Primary: S1, S10, S5. |
| Q38 | Is mpox more dangerous for people living with HIV? | It can be, especially for people with advanced or poorly controlled HIV or severe immunosuppression. People with well-controlled HIV may do better, but any suspected mpox should be assessed promptly and HIV care should be coordinated. | HIV consensus and severe-treatment guidance identify advanced HIV/severe immunocompromise as a risk for severe, prolonged, or disseminated disease and potential treatment complexity. | Primary: S5, S8, S1, S6. |
| Q39 | Can a breastfeeding mother with suspected mpox continue breastfeeding? | This should be individualized with clinicians and public-health staff. WHO found evidence insufficient for a strong universal recommendation. Decisions depend on lesions on the breast, infant risk, safe alternatives, and feasibility of IPC. If breastfeeding continues in selected situations, use strict hand hygiene, a medical mask, cover lesions, avoid feeding from a breast with lesions, and monitor the infant closely. | WHO breastfeeding sections explicitly discuss uncertain transmission through milk and direct contact, lack of strong evidence, implementation constraints, lesion location, alternatives, and IPC measures. Avoid a simple yes/no unless local guidance mandates it. | Primary: S1. Support: S7, S10, S6 for vaccine in breastfeeding if indicated. |

J. Sexual contact, privacy, and non-stigmatizing communication

| **ID** | **Public question** | **Suggested public answer** | **Mapped guideline content/rationale** | **Source classification** |
| --- | --- | --- | --- | --- |
| Q40 | Is mpox a sexually transmitted infection? | Mpox can spread during sex because sex involves close skin-to-skin and mucosal contact, but it is not limited to sex and is not defined only by sexual transmission. It can also spread through household, caregiving, contaminated materials, and some animal exposures. | Guidelines map sexual contact as a common close-contact setting rather than the only route. Public answer should avoid labeling mpox in a way that increases stigma or hides nonsexual transmission. | Primary: S3, S4, S6, S9. Supplement: E2. |
| Q41 | I am worried about mpox after one episode of unprotected sex, what should I do? | Assess whether the partner had rash, fever, known mpox, or exposure risk; avoid further close/sexual contact for now; monitor for symptoms for 21 days; seek STI/mpox evaluation if symptoms occur; ask public health or a clinician whether post-exposure vaccination is indicated. | Exposure-management and vaccine guidance support risk assessment, close-contact monitoring, STI co-evaluation, and PEP vaccination when criteria are met. Condoms alone do not prevent all skin contact with lesions. | Primary: S3, S4, S6, S7. |
| Q42 | Do men who have sex with men definitely get mpox? | No. Risk is about exposure, not identity. Some outbreaks have affected sexual networks among MSM, but anyone can get mpox through close contact with an infectious person or contaminated materials. Messaging should focus on behaviors and exposures. | Public and vaccine guidance emphasize targeted risk communication without stigma. ACIP 2025 notes vaccination eligibility should be handled in a way that avoids stigma; not all MSM are automatically at risk without exposure/risk factors. | Primary: S4, S6, S3. Supplement: E2. |
| Q43 | Should I tell my sexual partner if I suspect I have mpox? | Yes. Tell recent sexual and close contacts as soon as possible so they can monitor symptoms, avoid exposing others, seek testing if needed, and ask about post-exposure vaccination. Use respectful, factual language and protect privacy where possible. | Case investigation and contact tracing guidance requires identifying and managing close contacts. Public answer should make partner notification practical and non-blaming. | Primary: S3, S4, S6. |
| Q44 | Is it correct for people online to link mpox with certain groups of people? | No. It is misleading and stigmatizing to blame a group. Mpox risk is linked to exposure to the virus through close contact, not to a person’s identity. Stigma can delay care, testing, vaccination, and contact notification. | Public guidance and vaccine recommendations support non-stigmatizing, privacy-preserving communication and risk-based prevention. Systematic reviews can support context about affected networks, but not stigmatizing conclusions. | Primary: S1, S4, S6. Supplement: E2, E3. |

K. Travel, work, school, and public places

| **ID** | **Public question** | **Suggested public answer** | **Mapped guideline content/rationale** | **Source classification** |
| --- | --- | --- | --- | --- |
| Q45 | What should I pay attention to before traveling to an area where mpox is spreading? | Check current health advisories; avoid close contact with people who have rash or mpox symptoms; avoid unprotected contact with wild animals or animal products in endemic areas; practice hand hygiene; consider vaccination if eligible; know where to seek care if symptoms occur. | Public-health and public guidance include travel-related risk assessment, avoidance of animal and human exposures, symptom monitoring, and local outbreak-specific vaccination advice. | Primary: S3, S4, S6, S7. Supplement: E2/E3 for current outbreak context. |
| Q46 | If I develop a rash during a trip and suspect it might be mpox, can I still take a flight? | If you develop a rash during a trip and suspect mpox, you should not simply continue flying without advice. Cover lesions, wear a mask around others, avoid close contact, and seek medical or public-health guidance before taking a flight. Whether you can travel depends on clinical assessment and local public-health or airline requirements | Guidelines recommend isolation/limiting movement for suspected or confirmed cases and prompt assessment/reporting. Public answer should prioritize evaluation before travel continuation. | Primary: S2, S3, S4, S9. |
| Q47 | Could I get mpox from hotel bedsheets? | It is possible in theory if bedding is contaminated by an infectious person’s lesions and not properly laundered, but the risk is far lower than direct close contact. Use normally cleaned hotel linens, avoid visibly soiled items, and contact staff if bedding appears unclean. | Guidelines recognize contaminated bedding/linens as a transmission route and recommend laundry/disinfection precautions. Public answer should state “possible but generally low with proper laundering.” | Primary: S3, S4, S9, S7. |
| Q48 | If I develop a rash after returning from a business trip and worry it might be mpox, should I avoid close contact with my family first? | Yes. If you develop a rash after returning from a business trip and are worried it might be mpox, you should avoid close contact with family members until evaluated, especially skin-to-skin contact, sex, kissing, cuddling, shared bedding or towels, and contact with children, pregnant people, immunocompromised people, or pets. | Guidelines recommend isolation or movement limitation for suspected/confirmed cases, protection of household contacts, and environmental hygiene while awaiting evaluation. | Primary: S1, S2, S3, S4, S9. |

L. Animals, environmental disinfection, and misinformation

| **ID** | **Public question** | **Suggested public answer** | **Mapped guideline content/rationale** | **Source classification** |
| --- | --- | --- | --- | --- |
| Q49 | Do people get mpox only because they touched monkeys? | No. Mpox is a zoonotic disease, but human infection is not only from monkeys. In outbreaks, person-to-person transmission through close contact is very important. In endemic areas, contact with infected wild animals, meat, or animal products can also be a route. | Guidelines describe zoonotic origin and both animal-to-human and human-to-human transmission. Public answer should correct the misconception created by the old name “monkeypox.” | Primary: S2, S3, S4, S7. Supplement: E3. |
| Q50 | Can pets at home transmit mpox? | Possible animal susceptibility is a concern, so a person with suspected or confirmed mpox should avoid close contact with pets and other animals until cleared. If a pet was exposed or becomes ill, contact veterinary/public-health authorities rather than abandoning the animal. | Guidance advises avoiding contact with animals/wildlife and managing possible exposed animals cautiously. Map to household prevention and public-health/veterinary advice. | Primary: S3, S4, S7. Supplement: E3. |
| Q51 | How should I disinfect a phone or doorknob touched by someone suspected of having mpox? | Clean visible dirt first, then use an appropriate household or healthcare disinfectant according to the product label. Wear gloves if available, avoid shaking dust or contaminated materials, clean high-touch surfaces, and wash hands after cleaning. | IPC and control guidance emphasize cleaning plus disinfection of high-touch objects and environmental surfaces, with hand hygiene and PPE for contaminated materials. | Primary: S1, S3, S4, S9. |
| Q52 | Is it credible when people online say a certain herbal remedy can cure mpox? | Be cautious. No single online herbal remedy should be presented as a proven cure for mpox. Some Chinese guidelines include traditional Chinese medicine approaches as adjunctive, jurisdiction-specific care, but they should not replace diagnosis, isolation, supportive care, vaccination when indicated, or specialist treatment for severe disease. | Use formal guidelines for any TCM statement. The 2025 integrated guideline can support adjunctive TCM wording, while mainstream clinical guidelines emphasize supportive care and selective antivirals for severe/high-risk disease. Do not use social-media claims as evidence. | Primary: S1, S2, S5, S11. Supplement: E1 for guideline quality context. |

Appendix: how this mapping table was used for LLM response evaluation

This mapping table was used as the investigator-developed guideline-based reference framework for evaluating large language model responses. For each of the 52 public mpox consultation questions, evaluators compared the interface-generated response with the suggested public answer and the mapped guideline content. Accuracy was assessed according to consistency with the primary guideline sources; safety was assessed by identifying potentially harmful, misleading, over-reassuring, stigmatizing, or care-delaying statements; reliability was assessed by whether the response reflected the appropriate source hierarchy and acknowledged uncertainty or local-policy dependence when needed; readability was assessed by whether the response was understandable to the general public; and empathy was assessed by whether the response used respectful, non-stigmatizing, privacy-preserving, and supportive language.

For the final Q&A knowledge base, keep formal guidelines/consensuses as the primary answer source. Include non-guideline documents only as supporting materials under controlled labels:

Public education pages/factsheets: include for plain-language wording, myths, and communication tone, but not for detailed clinical recommendations.

Systematic reviews and meta-analyses: include to support background statements such as risk factors, outbreak epidemiology, evidence gaps, and guideline quality; do not use them to decide patient-specific treatment, isolation, or vaccine eligibility unless a guideline cites them.

Narrative reviews/primers: include only when they add useful specialty context (e.g., obstetrics, pediatrics) and the statement is consistent with guidelines.

Duplicate or overlapping CDC/MMWR vaccine files: keep the newest outbreak-specific ACIP recommendation for general vaccine policy and retain the 2022 occupational-risk recommendation for laboratory/occupational questions.

TCM/herbal-content documents: include only as jurisdiction-specific adjunctive guidance and never as proof that an online herbal remedy “cures” mpox.

**3. Independent expert review of the framework**

A. Characteristics and roles of independent framework reviewers

| **Reviewer** | **Institution** | **Professional role** | **Years of experience** | **Area of expertise** | **Other role in this study** |
| --- | --- | --- | --- | --- | --- |
| Reviewer A | Hangzhou Xixi Hospital, Hangzhou, China | Associate chief physician | 18 | Infectious diseases | None |
| Reviewer B | Hangzhou Xixi Hospital, Hangzhou, China | Director, Department of Infection Prevention and Control | 25 | Healthcare-associated infection prevention and control | None |

B. Results of independent framework review

| **Review outcome** | **Number of entries** |
| --- | --- |
| Accepted without revision | 52 |
| Minor clarification | 0 |
| Substantive revision | 0 |
| Insufficient evidence | 0 |
| Total | 52 |
