# Supplementary material for: Evaluating search-enabled large language model interfaces for mpox public health consultation: a guideline-based comparative study
Source: Front Public Health. 2026 Aug 26;14:1913260. doi: 10.3389/fpubh.2026.1913260 (PMC13553459; doi:10.3389/fpubh.2026.1913260)
Supplement: Supplementary file 3 [file Supplementary_file_3.DOCX]

**Model Information, Query Settings, and Structured Clean Q&A Record**

This document provides a clean, structured, question-based record of model responses for the mpox public health consultation evaluation study. One record equals one model answer. Stable markers are retained for readability extraction and later scoring. Duplicated text segments, obvious web-search source-list fragments, isolated source markers, and boundary artifacts were removed where identifiable, while preserving the substantive answer content.

1. **Model information**

| **Model interface** | **Model/version or mode used for querying** | **Search/retrieval setting** |
| --- | --- | --- |
| ChatGPT | GPT-5.5 Pro / Thinking | Web search enabled |
| Gemini | Gemini 3.0 Thinking / Deep Think mode | Search-enabled interface |
| Microsoft Copilot | Microsoft Copilot | Search mode / web-grounded interface |
| DeepSeek | DeepSeek-V4-Flash | Deep-thinking and intelligent-search functions enabled |
| Doubao | Doubao-Seed-2.0 | Web search and deep-thinking functions enabled |
| Qwen | Qwen3.5 Thinking mode | Web-search-enabled / tool-enabled interface |

Mode clarification: Model/version information was recorded according to the model or mode displayed in the public web interface at the time of querying and cross-checked against publicly available official release notes or documentation when available. Because some public chatbot interfaces may update interfaces dynamically or route queries internally without fully disclosing the underlying model, the reported model information should be interpreted as interface-level model/mode information rather than a guarantee of fixed backend model weights.

2. **Query settings**

| **Item** | **Recorded setting** |
| --- | --- |
| Query period | May 1-26, 2026 |
| Query location | Wenzhou, China |
| Question set | Final locked mpox public consultation question set containing 52 questions |
| Session handling | Each question was submitted in a newly opened independent chat session; prior page/session context was cleared before the next query |
| Prompting | The final question wording was used directly. No role prompt, prompt engineering, follow-up question, or manual correction was applied after the initial query |
| Response capture | Model outputs were saved immediately after generation |
| Record structure | Question identifier, model name, and answer text were retained for each response |

3. **Cleaning and validation summary**

| **Item** | **Value** |
| --- | --- |
| Number of questions | 52 |
| Number of interface responses | 312 |
| Expected design | 52 questions x 6 interfaces = 312 answers |
| Answers per question | 6 |
| Empty answers after cleaning | 0 |
| Interfaces | ChatGPT, Gemini, Copilot, DeepSeek, Doubao, Qwen |
| Cleaning note | Duplicated paragraphs, obvious source-list artifacts, isolated source markers, and identifiable record-boundary artifacts were removed. Model names were standardized to ChatGPT, Gemini, Copilot, DeepSeek, Doubao, and Qwen. |

Data check note: The cleaned record contains 52 questions, 312 interface responses, 6 answers per question, and no empty answers.

4. **Structured records**

Q1. What is mpox?

record_id Q1_ChatGPT

question_id Q1

model ChatGPT

answer_text:

Mpox-formerly called monkeypox-is a viral infection caused by the monkeypox virus, which is related to the smallpox virus but not related to chickenpox. It often causes a painful rash or sores, sometimes with fever, swollen lymph nodes, headache, muscle aches, back pain, chills, and fatigue. Symptoms usually begin within 21 days after exposure.

It spreads mainly through close contact, especially skin-to-skin or intimate contact with someone who has mpox, contact with rash fluid or scabs, contaminated items like bedding or towels, and sometimes contact with infected animals. CDC says it does not spread through casual contact like sitting near someone in an office/classroom, travel setting, or pool.

Most people recover with supportive care and pain control, but some people can become seriously ill, especially children, pregnant people, and people with weakened immune systems. Vaccination with JYNNEOS can help prevent mpox before or after some exposures.

record_id Q1_Gemini

question_id Q1

model Gemini

answer_text:

Mpox (formerly known as monkeypox) is a viral disease caused by the mpox virus, which is part of the same family of viruses as smallpox (Orthopoxvirus). While it is clinically less severe than smallpox, it can still cause significant illness.

Key Symptoms

Mpox typically begins with flu-like symptoms followed by a distinct rash. Symptoms usually appear within 1 to 3 weeks after exposure:

Rash: This is the most common sign. It often starts as flat spots that turn into raised bumps, then fluid-filled blisters, which eventually scab over and fall off. The rash can be painful or itchy.

Fever and Chills: Often the first sign of infection.

Swollen Lymph Nodes: A key feature that distinguishes mpox from smallpox or chickenpox.

Exhaustion and Aches: Muscle aches, backaches, and intense fatigue are common.

How It Spreads

The virus is transmitted through close, personal contact:

Person-to-Person: Through direct contact with mpox rashes, scabs, or body fluids. It can also spread through prolonged face-to-face contact (respiratory droplets) or intimate physical contact.

Contaminated Materials: Touching items like clothing, bedding, or towels that have been used by someone with an active rash.

Animal-to-Human: In areas where the virus is endemic (primarily Central and West Africa), it can spread through bites, scratches, or preparing meat from infected wild animals.

Prevention and Treatment

Most people recover within 2 to 4 weeks without specialized treatment, but the following measures are used to manage the disease:

Vaccination: Vaccines originally developed for smallpox (such as JYNNEOS) are highly effective at preventing mpox or reducing the severity of the illness if given shortly after exposure.

Medical Care: Treatment focuses on managing symptoms, such as pain relief and keeping the rash clean. In severe cases or for high-risk individuals, antiviral drugs like tecovirimat (TPOXX) may be prescribed.

Isolation: Infected individuals are advised to isolate until all scabs have fallen off and a fresh layer of skin has formed.

Naming Note

In late 2022, the World Health Organization (WHO) began using the term "mpox" to replace "monkeypox." This change was made to reduce the use of racist and stigmatizing language associated with the original name while ensuring the scientific community could still track the virus effectively.

record_id Q1_Copilot

question_id Q1

model Copilot

answer_text:

Mpox is a viral illness (formerly called monkeypox) that usually causes fever, swollen lymph nodes and a painful skin or mucosal rash; it spreads mainly through close physical contact and can be prevented with vaccines and infection-control measures.

What mpox is

Mpox is caused by the monkeypox virus (an Orthopoxvirus) and has two main genetic groups (clade I and clade II). Most infections cause a self-limited illness lasting 2-4 weeks, though severe disease can occur.

Typical symptoms

Early symptoms: fever, headache, muscle aches, back pain, chills, fatigue, and swollen lymph nodes.

Rash: appears 1-4 days after fever; lesions progress from flat spots to blisters, then scab and fall off over weeks. Lesions may appear on the face, hands, feet, genitals, mouth, or throat. Most people recover without complications, but some require medical care.

How mpox spreads

Main routes: close skin-to-skin contact (including sexual contact), direct contact with lesions or body fluids, respiratory droplets during prolonged face-to-face contact, and contact with contaminated objects (clothing, bedding, needles). Animal-to-human transmission is also possible. People with multiple sexual partners have been at higher risk in recent outbreaks.

Prevention and vaccines

Vaccination is available and recommended for people at higher risk or after exposure; the vaccine is a key tool alongside safer-sex practices and avoiding close contact with symptomatic people.

Everyday precautions: avoid direct contact with rashes or scabs, do not share bedding or clothing with someone who is ill, practice good hand hygiene, and use masks if caring for someone with mpox.

Diagnosis and treatment

Diagnosis is by clinical exam and laboratory testing of lesion swabs.

Treatment is mainly supportive (pain control, hydration, wound care); antiviral drugs developed for smallpox may be used in severe cases or for people at high risk of complications. Seek care if you have a new unexplained rash or mpox symptoms.

Quick, practical steps (Los Angeles context)

If you have a new rash or symptoms, isolate and contact your healthcare provider or local public health for testing.

If you were exposed, ask about post-exposure vaccination and monitoring.

Avoid close contact with pets until you've been evaluated, because animals can be infected.

record_id Q1_DeepSeek

question_id Q1

model DeepSeek

answer_text:

Mpox (formerly known as monkeypox) is a rare viral disease caused by infection with the monkeypox virus. It is part of the same family of viruses as the smallpox virus (Orthopoxvirus), but it is generally much less severe.

Think of it as a less dangerous cousin to smallpox.

Key things to know about mpox:

Symptoms: The most common symptoms include a distinctive rash that goes through several stages (from flat spots to fluid-filled blisters to scabs), along with flu-like symptoms like fever, headache, muscle aches, swollen lymph nodes, and exhaustion. The swollen lymph nodes are a key feature that helps distinguish mpox from smallpox or chickenpox.

Transmission: Mpox spreads from person to person through close, physical contact with an infected person's rash, scabs, or body fluids. It can also spread through respiratory droplets but usually requires prolonged face-to-face interaction. In the current global outbreak (which began in 2022), the virus has primarily spread through close, intimate contact, including sexual contact. It can also spread from infected animals to people.

Severity: The disease is usually self-limiting, meaning it resolves on its own. Symptoms typically last 2-4 weeks. Most people recover fully without treatment. However, it can be severe, especially in children, pregnant people, or those with weakened immune systems.

Prevention & Treatment: There is a vaccine (JYNNEOS) that is effective at preventing mpox. For those who get sick, treatment is generally supportive (managing symptoms). An antiviral drug developed for smallpox (tecovirimat, or TPOXX) can also be used to treat severe cases.

Name Change: The World Health Organization (WHO) renamed "monkeypox" to "mpox" in late 2022 to reduce stigma and racism associated with the original name. Both names refer to the same disease.

In short, mpox is a viral illness that causes a painful rash and flu-like symptoms, spreading mainly through close physical contact. While concerning, it is rarely fatal for most people.

record_id Q1_Doubao

question_id Q1

model Doubao

answer_text:

Mpox (formerly monkeypox) is a viral zoonotic disease caused by the monkeypox virus (MPXV), a member of the Orthopoxvirus genus in the Poxviridae family (same family as smallpox). It causes a distinctive rash, fever, and swollen lymph nodes, with most cases resolving spontaneously but potentially severe in vulnerable groups.

Basic Definition & Nomenclature

Virus classification: Enveloped double-stranded DNA virus with two primary clades:

Clade I (Congo Basin): More severe, ~10% mortality historically

Clade II (West African): Less severe (~0.1% mortality), responsible for 2022 global outbreak

Natural reservoir: Likely small mammals (e.g., squirrels) in Central/West Africa, not monkeys

History

1958: First identified in research monkeys (hence original name)

1970: First human case reported in Democratic Republic of Congo (DRC)

2003: First outbreak outside Africa (United States) linked to imported animals

2025: PHEIC lifted as cases declined globally

Transmission

Mpox spreads through close contact with:

Infected people: Via bodily fluids, skin lesions, respiratory droplets, or contaminated objects

Infected animals: Direct contact with blood, bodily fluids, or lesions of infected animals (e.g., rodents, primates)

Mother-to-child: During pregnancy, birth, or postnatal contact

Key transmission settings:

Sexual contact (primary mode in recent outbreaks)

Household contact

Healthcare settings (via contaminated equipment)

Clinical Presentation

Incubation period: 6-13 days (range: 5-21 days)

Typical progression:

Prodromal phase (1-5 days):

Fever, headache, muscle aches, fatigue

Enlarged lymph nodes (distinguishes from smallpox)

Chills, swollen tonsils

Rash phase:

Starts on face, spreads to extremities (palms/soles often affected)

Lesions progress: macules to papules to vesicles to pustules to scabs

Painful lesions (especially in mucosal areas: mouth, eyes, genitals)

Lasts 2-4 weeks; scars may remain

Atypical presentations in recent outbreaks:

Rash limited to genital/anal areas

Lesions appearing before systemic symptoms

Proctitis (inflammation of rectum)

Severity & Complications

Mortality rate: 0.1-10% (depends on clade, access to care, and patient factors)

High-risk groups:

Children <8 years

Immunocompromised individuals (HIV, transplant recipients)

Pregnant women (risk of fetal/neonatal death)

Complications: Secondary bacterial infections, pneumonia, encephalitis, eye infections (can cause blindness)

Prevention & Treatment

Prevention

Vaccination:

JYNNEOS (Imvamune/Imvanex): Two-dose vaccine approved for prevention, recommended for high-risk individuals (close contacts, healthcare workers)

ACAM2000: Smallpox vaccine with cross-protection (higher risk of side effects)

Protective measures:

Avoid close contact with infected people/animals

Use PPE (gloves, masks) when caring for patients

Practice good hand hygiene

Avoid sharing personal items with infected individuals

Treatment

Supportive care: Hydration, pain management, fever reduction

Antiviral therapy:

Tecovirimat (TPOXX): Approved for treatment of mpox in adults/children

Cidofovir, brincidofovir (used in severe cases)

Wound care: Prevent secondary infections, keep lesions clean/dry

Current Status

Global outbreak peaked in 2022-2023; cases declined significantly by 2025World Health Organization (WHO)

Endemic in Central/West Africa with periodic outbreaks

WHO maintains mpox as a monitorable disease requiring continued surveillance

Mpox is a preventable and treatable disease with proper public health measures and medical care. Most people recover fully within 2-4 weeks, but vigilance remains essential, especially in high-risk settings.

record_id Q1_Qwen

question_id Q1

model Qwen

answer_text:

mpox is a viral zoonotic disease caused by the monkeypox virus (MPXV), characterized by fever, lymphadenopathy (swollen lymph nodes), and a progressive skin rash. Originally named "monkeypox," the World Health Organization (WHO) officially adopted "mpox" as the preferred term in November 2022 to reduce stigma and align with modern disease-naming standards. The current global outbreaks are primarily driven by clade Ib, a more virulent strain with higher transmission efficiency and fatality rates compared to earlier variants. WHO declared mpox a Public Health Emergency of International Concern (PHEIC) for the second time on August 14, 2024, due to rapidly spreading clade Ib cases in Africa and beyond.

1. Pathogen and Classification

Virus type: Double-stranded DNA orthopoxvirus (family Poxviridae), closely related to smallpox virus but less transmissible and less lethal.

Genetic clades:

Clade I (formerly Congo Basin): Higher virulence, fatality rate of 1-10%, with clade Ib now causing widespread human-to-human transmission.

Clade II (formerly West African): Lower fatality rate (<0.1%), responsible for the 2022-2023 global outbreak.

Naming update: "mpox" replaces "monkeypox" as the official disease name, though the virus retains "monkeypox virus" temporarily.

2. Clinical Features

Key symptoms:

Incubation period: 5-21 days (typically 6-13 days).

Prodromal phase: Fever (>38.5 degrees C), headache, muscle/back pain, prominent lymph node swelling (a key distinction from smallpox and chickenpox).

Rash progression: Starts on face or genitals, spreading to limbs; evolves from macules to papules to vesicles to pustules to scabs over 2-4 weeks.

Atypical presentations: In recent outbreaks, rashes often begin in genital/anal/oral areas, sometimes without systemic symptoms.

Severe complications:

High-risk groups: Children, pregnant individuals, and immunocompromised people (e.g., uncontrolled HIV) face higher risks of severe disease or death.

Common complications: Secondary bacterial infections, pneumonia, encephalitis, or corneal scarring leading to vision loss. Clade I infections carry a fatality rate up to 10% in resource-limited settings.

3. Transmission

Primary routes:

Direct contact: With infectious skin lesions, bodily fluids, or scabs (e.g., sexual contact, caregiving).

Respiratory droplets: Requires prolonged face-to-face exposure.

Fomites: Contaminated clothing, bedding, or surfaces.

Animal-to-human: Through bites/scratches or handling infected rodents/primates (mainly in endemic African regions).

Current outbreak dynamics:

Clade Ib spreads more efficiently between humans than prior strains, with documented community transmission outside Africa (e.g., Thailand, Sweden).

Unlike the 2022-2023 outbreak (driven by sexual transmission among men who have sex with men), clade Ib shows broader transmission patterns, including household and pediatric cases.

4. Prevention and Treatment

Prevention:

Vaccines:

JYNNEOS (MVA-BN): The primary vaccine used globally; 85% effective against clade II, but efficacy against clade Ib is still being studied.

Historical smallpox vaccination: Provides partial cross-protection but has waned in most populations since routine vaccination ended after 1980.

Non-pharmaceutical measures: Avoid close contact with infected individuals, practice hand hygiene, and isolate until all scabs fall off (typically 2-4 weeks).

Treatment:

Supportive care: Pain management, wound care, and hydration (most cases are self-limiting).

Antivirals:

Tecovirimat (TPOXX): First-line treatment for severe cases or high-risk patients, though clinical benefit in mild/moderate cases is limited.

Cidofovir/brincidofovir: Reserved for tecovirimat-resistant cases due to toxicity risks.

5. Current Global Situation (as of 2025)

Case burden: Over 15,600 cases and 537 deaths were reported worldwide in 2024 alone, exceeding 2023 totals. Fatality rates are disproportionately high in children (up to 85% in infants under 6 months in some outbreaks).

Critical gaps: Limited vaccine access in Africa, diagnostic shortages, and evolving transmission patterns (e.g., respiratory spread) pose ongoing challenges.

Q2. Is the mpox people talk about now the same disease as monkeypox?

record_id Q2_ChatGPT

question_id Q2

model ChatGPT

answer_text:

Yes. Mpox is the same disease that was long called monkeypox; "mpox" became the preferred WHO term in 2022 to reduce stigma and confusion. WHO describes mpox as a synonym/replacement for monkeypox, and CDC has also used both terms in public-health materials.

One nuance: the virus is still often called monkeypox virus or MPXV, even when the disease is called mpox. Also, current outbreaks may involve different clades or variants of the virus, such as clade IIb from the 2022 global outbreak and clade I outbreaks reported since late 2023, but they are still forms of the same disease caused by MPXV.

record_id Q2_Gemini

question_id Q2

model Gemini

answer_text:

Yes, mpox is the same disease as monkeypox. They are simply different names for the exact same viral illness.

Why the Name Changed

The World Health Organization (WHO) officially recommended the name change in November 2022. The decision was made to move away from "monkeypox" due to concerns about racist and stigmatizing language that surfaced during the 2022 global outbreak.

While the disease is now called mpox, the virus that causes it is still scientifically referred to as the monkeypox virus (MPXV).

Key Facts About Mpox

Origin: Despite the name, monkeys aren't actually the primary source; the virus is more commonly found in small rodents. It was first identified in 1958 in lab monkeys, which is how it got its original name.

Transmission: It spreads primarily through close, personal contact, including:

Direct skin-to-skin contact with rashes or scabs.

Intimate physical contact or sex.

Touching contaminated materials like bedding or towels.

Symptoms: Most people experience a painful rash, fever, swollen lymph nodes, and muscle aches. The rash eventually turns into blisters that scab over and heal.

Why People Are Talking About It Now

The reason mpox remains in the headlines (including in 2026) is due to the emergence of different "clades" or strains.

Clade II: This was the version responsible for the global outbreak in 2022. It is generally less severe.

Clade I (specifically Clade Ib): This strain has caused more recent concern because it can cause more severe illness and has been spreading in parts of Central and Eastern Africa, with occasional cases appearing elsewhere due to travel.

The bottom line: If you hear someone say "mpox," they are just using the updated, preferred medical term for the disease formerly known as monkeypox.

record_id Q2_Copilot

question_id Q2

model Copilot

answer_text:

Yes - "mpox" is the same disease that was previously called "monkeypox"; public health agencies adopted the shorter name to reduce stigma while the virus and illness remain the same.

What changed and why

Mpox is simply a new, less stigmatizing name for the disease caused by the monkeypox virus (MPXV). The World Health Organization and U.S. public health agencies recommended and adopted the name change in late 2022 to avoid unnecessary offense and confusion while keeping clinical and laboratory definitions intact.

What the disease is now called and what causes it

Disease name: Mpox (formerly called monkeypox).

Cause: The illness is caused by the monkeypox virus (MPXV), an Orthopoxvirus related to smallpox and cowpox.

Key facts about symptoms and transmission

Common symptoms include fever, headache, muscle aches, swollen lymph nodes, and a characteristic rash that progresses through blistering and scabbing over 2-4 weeks.

How it spreads: Mainly through close physical contact (skin-to-skin, sexual contact, kissing), contact with contaminated materials (clothing, bedding), and, less commonly, respiratory droplets during prolonged face-to-face contact.

Variants and recent outbreaks

The virus has two main clades (Clade I and Clade II) with subclades; the global outbreak that began in 2022 was driven by Clade IIb.

Prevention and treatment

Vaccination: Smallpox vaccines are effective against mpox and are recommended for people at higher risk or after exposure.

Treatment: Mostly supportive care (pain control, wound care, hydration); antivirals such as tecovirimat may be used in severe cases.

When to see a clinician

Seek medical care if you develop a new unexplained rash, especially with fever or swollen lymph nodes, or if you had close contact with someone known or suspected to have mpox. Testing (PCR of a lesion) confirms the diagnosis.

Bottom line: The illness people now call mpox is the same disease previously called monkeypox; only the public-facing name changed to reduce stigma, while clinical definitions, the causative virus, symptoms, and prevention measures remain the same.

record_id Q2_DeepSeek

question_id Q2

model DeepSeek

answer_text:

Yes, the "mpox" we hear about now is the exact same disease as monkeypox.

The name was officially changed in late 2022 by the World Health Organization (WHO) to reduce stigma. Here's what you need to know:

A New Name for an Old Virus

Official Name Change: The WHO announced in November 2022 that the disease's preferred term would be "mpox", and after a one-year transition period, the old name "monkeypox" was fully phased out-.

Why the Change?: The change was made primarily to avoid the racist and stigmatizing language that emerged during the 2022 global outbreak-12. The WHO also cited its best practices to avoid naming diseases after animals, which can lead to unnecessary negative impacts on animal welfare and public perception

Same Disease, Different Name: Biologically, "mpox" is still caused by the monkeypox virus-, which is in the same virus family as the one that causes smallpox

Quick Comparison: Mpox vs. Monkeypox

For clarity, here's a comparison of the name and its properties:

Feature Mpox (New Name) Monkeypox (Former Name)

Primary Name Mpox (current) Monkeypox (phased out)

Causative Agent Monkeypox virus (MPXV)

Comparison The new, official term for the disease. The historical term, now largely replaced.

record_id Q2_Doubao

question_id Q2

model Doubao

answer_text:

Yes, mpox and monkeypox refer to exactly the same disease.

The World Health Organization (WHO) officially renamed "monkeypox" to mpox in 2022.

The change was made mainly to avoid geographical stigma, discrimination and misleading associations linked to the original name.

The virus, symptoms, transmission routes and clinical characteristics are completely unchanged - it's just an updated official name for the same illness.

record_id Q2_Qwen

question_id Q2

model Qwen

answer_text:

Yes, mpox and monkeypox refer to the exact same disease. The term "mpox" was officially adopted by the World Health Organization (WHO) in November 2022 as a preferred replacement for "monkeypox" to reduce stigma and racial connotations, but both names describe the identical viral illness caused by the monkeypox virus.

1. Name Change Background

In November 2022, WHO announced that "mpox" would become the preferred term for the disease previously known as "monkeypox," with both names used interchangeably for a one-year transition period before fully retiring "monkeypox" .

The change followed concerns that the original name unfairly associated the disease with Africa and contributed to racial stereotypes, as the virus is not primarily linked to monkeys or any specific geographic region .

2. Key Clarifications

a) Same Disease, Updated Terminology

The virus itself remains unchanged; only the preferred naming convention was updated. Clinically, diagnostically, and epidemiologically, mpox and monkeypox are identical .

WHO emphasized that the renaming aligns with global best practices to avoid disease names tied to animals, people, or locations, which can fuel discrimination .

b) Reasons for the Change

Stigma reduction: Early 2022 outbreaks saw misinformation linking the disease to African populations, despite human-to-human transmission being the primary driver in non-endemic regions .

Scientific accuracy: Monkeys are not the natural reservoir of the virus; small rodents in African rainforests are suspected primary hosts .

c) Current Usage

As of 2024-2026, "mpox" is the standard term in WHO communications, scientific literature, and public health guidelines. However, "monkeypox" may still appear in older resources or informal contexts .

The International Classification of Diseases (ICD-11) now lists "mpox" (code 1E71) as the official designation, though "monkeypox" remains searchable for historical continuity .

3. Why This Matters Practically

Diagnosis and treatment protocols are unchanged. Symptoms (e.g., fever, lymph node swelling, progressive skin lesions), transmission routes (close contact with lesions/body fluids), and management (supportive care, antivirals for severe cases) apply equally to both terms .

Public health alerts (e.g., WHO declaring mpox an "international public health emergency" in August 2024) use "mpox" exclusively in current communications .

In short, "mpox" is not a new disease-it is simply the updated, stigma-free name for what was previously called monkeypox. All medical and epidemiological information about the disease remains fully applicable under the new terminology.

Q3. How serious is the mpox risk in my country right now?

record_id Q3_ChatGPT

question_id Q3

model ChatGPT

answer_text:

Overall: mpox risk for most people in the U.S. is still low, but it is not zero. CDC says the clade I outbreak risk to most people in the U.S. remains low; as of May 5, 2026, the U.S. had 20 lab-confirmed clade I cases since November 2024, tied to travel from outbreak areas or links to travelers. Clade II mpox is still circulating at low levels in the U.S.

For California, the risk is more worth paying attention to. CDPH reported rising clade II activity: 14.5 weekly cases in 2026, compared with 5.8 in 2024 and 3.4 in 2025 for the same period, and urged higher-risk Californians to get vaccinated.

The practical takeaway: you probably do not need to worry about everyday public contact like being in an office, classroom, plane, or pool. CDC says mpox spreads mainly through close or intimate contact, contaminated items, or infected animals in endemic areas, and does not spread through the air or casual contact like typical travel/classroom/office contact.

You should take it more seriously if you have had a known exposure, have a sex partner diagnosed with mpox, have multiple/new sexual partners, attend sex-on-premises venues or large events where close skin-to-skin contact may occur, or are traveling to areas with active clade I or new clade II outbreaks. CDC recommends two doses of JYNNEOS for eligible people, and both doses give the best protection.

Watch for a new or unexplained rash, especially with fever, chills, swollen lymph nodes, fatigue, muscle aches, headache, or recent close contact with someone who might have mpox. Symptoms usually start within 21 days after exposure; if you develop a new rash, contact a healthcare provider for testing and avoid close contact until evaluated.

record_id Q3_Gemini

question_id Q3

model Gemini

answer_text:

As of May 2026, the overall risk of mpox to the general public in the United States remains low. However, the risk is categorized as low-to-moderate for specific groups, particularly men who have sex with men (MSM) and their sexual partners.

Public health agencies are currently monitoring two distinct versions of the virus:

1. Clade I (The Newer Concern)

Clade I (specifically subclade Ib) has drawn international attention recently due to outbreaks in Central and Eastern Africa and subsequent travel-related cases in Europe and the Americas.

Current Status: As of early May 2026, there have been 20 laboratory-confirmed cases of Clade I in the U.S.

Transmission: Most U.S. cases have been linked to international travel or close contact with travelers. While there have been instances of limited local transmission, it has not yet led to widespread community spread.

Severity: Although Clade I is historically associated with more severe illness than Clade II, recent cases in the U.S. and Europe have generally been mild, with no reported deaths in these regions.

2. Clade II (The 2022 Outbreak Strain)

This is the version responsible for the global outbreak that began in 2022.

Current Status: Clade IIb continues to circulate in the U.S. at low levels. There was a slight increase in cases in late 2025, but the overall trend has remained stable through early 2026.

Impact: Most cases occur in individuals who are unvaccinated or have only received one dose of the vaccine.

Prevention and Protection

Vaccination remains the most effective tool for those at higher risk.

Vaccination: The JYNNEOS vaccine is highly effective at preventing infection or reducing the severity of symptoms. A full course requires two doses, administered about four weeks apart.

Monitoring Symptoms: Be aware of new, unexplained rashes or sores (which may look like pimples or blisters), fever, chills, and swollen lymph nodes.

Contact Precautions: Avoid close, skin-to-skin contact with anyone who has a rash that looks like mpox, and avoid sharing personal items like bedding or towels with someone who is infected.

If you believe you have been exposed or are at higher risk due to upcoming travel or sexual networks, consulting a healthcare provider about the vaccine is recommended. Most local health departments continue to provide updated testing and vaccination resources.

record_id Q3_Copilot

question_id Q3

model Copilot

answer_text:

Short answer - For most people in the United States right now the mpox (monkeypox) risk is low, but outbreaks continue in specific networks and some travel-linked clade I cases have been detected; people with recent travel to affected areas or who have intimate contact with someone symptomatic should take precautions and consider vaccination if eligible.

Current situation (national and global)

United States: Public health authorities report that the risk to most people in the U.S. remains low. Since November 2024 there have been ~20 laboratory-confirmed clade I cases in the U.S., mostly travel-associated or linked to travelers. Clade II (the 2022 lineage) continues to circulate at low levels.

Global: WHO surveillance shows ongoing transmission worldwide, with tens of thousands of cases reported across regions and continued activity in parts of Africa and Europe; WHO warns that clade Ib and other clades remain active and could seed local outbreaks if not contained.

Local signals: Recent state and local reports show sporadic clusters in some U.S. jurisdictions (for example, recent cases reported in Pennsylvania and Nevada), indicating that local flare-ups can occur even when national risk is low.

Who is at higher risk right now

People with recent travel to areas with active clade I or new clade II outbreaks.

People with close intimate or sexual contact within networks where transmission is occurring (WHO and CDC note sexual networks and household spread are common transmission routes).

Household contacts of a case and some healthcare workers without appropriate PPE in rare situations.

What to watch for (symptoms and testing)

Symptoms: fever, swollen lymph nodes, new rash or lesions (often on genitals, face, hands), and flu-like symptoms. If you have these symptoms and recent exposure or travel, seek testing and isolate until evaluated.

Practical steps and recommendations

If you're not in a high-risk group: continue routine precautions; risk is low for the general public.

If you are in a higher-risk group or had exposure: contact your local health department or healthcare provider about testing and vaccination (two-dose mpox vaccine is recommended for eligible people).

Avoid close skin-to-skin and sexual contact with anyone who has a new unexplained rash; use condoms and reduce number of partners while outbreaks are active.

Bottom line

Mpox is not a major threat to the general U.S. population right now, but it remains an active public-health concern in specific networks and regions; stay informed, watch for symptoms after travel or exposure, and get vaccinated if you meet eligibility criteria.

record_id Q3_DeepSeek

question_id Q3

model DeepSeek

answer_text:

Mpox remains a global health threat, but its risk level currently varies dramatically by country-. While some regions are seeing increased cases, the overall risk to the general public in many places like the U.S. and Singapore is considered low-. To give you a more personalized understanding, here's a breakdown of the current situation.

A Quick Overview of Mpox

As of May 2026, the World Health Organization (WHO) continues to classify the mpox outbreak as a Public Health Emergency of International Concern (PHEIC), which is its highest alert level-. This is due to the continuing spread of two main types, known as Clade I (historically more severe) and Clade II (the type causing the 2022 global outbreak)-.

Risk Levels by Country (as of May 2026)

Region / Country Risk Level to General Public Key Developments

Global Varies by country 1235 confirmed cases across 48 countries in March 2026 alone-.

United States Low- Circulation of Clade I and II at low levels-. Notable clusters reported in California, Nevada, Missouri, and KC-.

Singapore Low- Detected two locally transmitted Clade Ib cases in April 2026-.

United Kingdom N/A Reported 45 Clade Ib cases and a total of 944 Clade IIb cases as of March 2026-.

Liberia & Ghana Endemic / Outbreak Outbreak of Clade II; CDC has issued a "Level 2: Practice Enhanced Precautions" Travel Health Notice-.

Africa (Continental) Varies by country Emergency status has been lifted due to declining trends-. Suspected cases declined 40% from early to late 2025, and the case fatality rate dropped from 2.6% to 0.6%- but areas of concern remain.

Madagascar Increased Reporting an outbreak, with approximately 100 suspected cases and 10 confirmed cases as of December 2025-.

The risk is not uniform; trends show a decline in some areas and local increases in others. Based on the latest reports:

How to Determine Your Personal Risk

Assessing your risk involves looking at two main things: your location and your activities.

Check Your Location: If you're in a high-risk area (e.g., parts of Central/Eastern Africa, or Liberia/Ghana), your general risk is higher. If you're in a low-risk area (e.g., US, Singapore), the general public risk is low, but localized clusters can still occur.

Evaluate Your Activities: The most important factor is your personal exposure. The risk currently isn't about general day-to-day activities, but more about specific situations.

Higher Risk: The primary mode of transmission continues to be direct contact with the rash, scabs, or body fluids of an infected person, or with contaminated materials like clothing or bedding.

Sexual Contact: Many recent cases, including in Singapore, are linked to sexual networks, particularly among men who have sex with men (MSM)-.

Household Contact: Living in close quarters with an infected person is also a risk.

Key Precautions to Take

Regardless of where you live, these steps can help you protect yourself:

Know the Symptoms: Watch for a rash or sores that look like pimples or blisters, often but not always accompanied by flu-like symptoms (fever, chills, swollen lymph nodes).

Limit Close Contact: Avoid close physical or sexual contact with anyone who has a rash or is feeling unwell.

Practice Good Hygiene: Wash your hands frequently with soap and water or use an alcohol-based hand sanitizer.

Get Vaccinated: If you are in a high-risk group (e.g., MSM with multiple partners, or healthcare workers), talk to your healthcare provider. Many countries offer vaccines to at-risk individuals-.

Stay Informed: Monitor updates from your local health department for the most current situation in your area.

Summary

To summarize, mpox is a dynamic global situation, but for most people in most parts of the world, the current health risk is low.

The most accurate and up-to-date information will always come from official sources. I recommend checking the website of your country's health ministry or the WHO for region-specific guidance.

record_id Q3_Doubao

question_id Q3

model Doubao

answer_text:

Current Mpox Risk in China (as of May 11, 2026)

Overall risk for the general population: Low

Key transmission status: Limited, well-controlled outbreaks

Risk Assessment Details

1. Official Evaluations

China CDC/National Disease Control and Prevention Administration (April 2026):

Mpox is listed as a disease requiring increased vigilance due to rising global cases, but overall risk remains low in China . The main concern is importation risk from international travel, especially during holiday periods .

WHO Global Assessment (updated February 2026):

Risk is moderate for men who have sex with men (MSM) with new/multiple partners, sex workers, and those with multiple casual sexual partners; low for the general population without specific risk factors World Health Organization (WHO).

2. Current Transmission Situation

Clade IIb (original global outbreak strain):

Maintains low-level transmission primarily among MSM in China, with no widespread community spread .

Clade Ib (Central African strain):

Cases are mostly imported or limited to close contacts of imported cases, with no sustained community transmission .

Recent case data (March 2026):

National: Limited sporadic cases

Zhejiang Province (where you're located): 5 reported cases, 0 deaths

No reports of widespread outbreaks or community transmission in any Chinese province .

3. Risk Factors and Transmission

Mpox spreads mainly through:

Direct skin-to-skin contact with lesions, body fluids, or contaminated materials

Respiratory droplets during prolonged close contact (not through brief conversations)

Sexual contact (most common mode in current outbreaks)

Higher-risk scenarios:

Recent international travel to mpox-affected regions (especially Central/West Africa, parts of Europe)

Participation in high-risk sexual behaviors (multiple partners, anonymous sex) World Health Organization (WHO)

Close contact with confirmed mpox cases

4. Protective Measures

General population:

Maintain good hygiene (handwashing, avoid contact with unfamiliar skin lesions)

Stay informed about travel advisories to affected areas

Seek medical attention if you develop fever + rash/lesions

Higher-risk groups:

Limit sexual partners, use condoms

Avoid close contact with symptomatic individuals

Consider pre-exposure vaccination if traveling to high-risk regions

Summary

The mpox risk in China is currently low for most people, with limited, well-controlled transmission primarily in specific high-risk groups . The main concern is importation risk from international travel, which is being actively monitored by Chinese health authorities .

record_id Q3_Qwen

question_id Q3

model Qwen

answer_text:

The severity of mpox risk depends entirely on your specific country and personal risk factors, as global transmission patterns vary significantly. As of mid-2026, mpox poses a low risk to the general population in most non-endemic countries, but higher risks persist in certain regions (especially parts of Africa) and for specific groups like men who have sex with men (MSM), immunocompromised individuals, and children under 5. Below is a structured analysis based on the latest data.

1. Global Risk Overview (Mid-2026)

Current Epidemiological Trends

Overall decline in cases: Global mpox cases have decreased since the 2022-2024 peaks, but clade I (particularly Ib subclade) remains active in Africa and shows limited community spread elsewhere. As of March 2026, 48 countries reported 1,235 new cases, with Africa accounting for ~70% of global cases.

Terminated PHEIC status: The WHO ended its "International Health Emergency" declaration for mpox in September 2025 after case numbers fell, but clade I transmission in Africa and sporadic international spread keep it under active surveillance.

Virus evolution concerns: Clade Ib (historically confined to Central Africa) is now causing community transmission in non-African countries (e.g., Spain, Germany, the U.S.), primarily among MSM networks. Three recombinant strains (Ib/IIb) have also been detected, though their clinical impact remains unclear.

Regional Risk Variation

a) High-Risk Regions

Central/East Africa: The Democratic Republic of Congo (DRC) remains the epicenter, with clade I driving severe outbreaks. In 2024-2026, DRC reported over 124,000 suspected cases and 2,200+ deaths, primarily among children under 5 and immunocompromised individuals. Clade I has a higher fatality rate (~1-10%) compared to clade II (~0.1%).

Other affected African nations: Burundi, Kenya, Liberia, and Uganda continue to report significant clade I transmission, with limited healthcare access worsening outcomes.

b) Moderate/Low-Risk Regions (Outside Africa)

Europe and the Americas: Most cases are linked to travel or localized MSM networks. For example:

The U.S. reported 37,000+ total cases (2025-2026) but no sustained community spread beyond high-risk groups. Clade Ib cases (e.g., California, Georgia) were travel-related or involved close-contact transmission.

The EU/EEA saw declining cases in 2025-2026, though Spain, Portugal, and Germany reported clade Ib community transmission among MSM.

Asia-Pacific: Thailand, Malaysia, and Singapore report sporadic cases (often travel-associated), with no evidence of widespread community transmission. China's cases are primarily clade IIb (milder), though 5 imported clade Ib cases were contained in early 2026.

2. Key Risk Factors for Individuals

Your personal risk depends on:

a) Population-Specific Vulnerabilities

Men who have sex with men (MSM): Remain the most affected group globally outside Africa, with >90% of non-African cases linked to sexual contact. Clade Ib's spread in MSM networks is now confirmed in 8+ non-African countries.

Immunocompromised individuals: People with advanced HIV (CD4 <200 cells/microL), organ transplant recipients, or untreated immunosuppressive conditions face higher risks of severe disease, prolonged symptoms, and death.

Children under 5: In endemic regions (e.g., DRC), children account for 39% of clade I cases and 60% of deaths due to limited healthcare access and malnutrition.

b) Behavioral and Contextual Risks

Close skin-to-skin contact (including sexual activity) with infected individuals is the primary transmission route. Respiratory droplets and contaminated objects pose secondary risks.

Travel to endemic areas: Visiting regions with active clade I transmission (e.g., DRC, Burundi) increases exposure risk, especially in crowded settings or conflict zones with weak healthcare systems.

3. Protective Measures and Monitoring

a) For Low-Risk General Populations

No widespread community transmission exists in most non-endemic countries. Routine hygiene (handwashing, avoiding contact with lesions) suffices for most people without specific exposures.

Vaccines are available but targeted: JYNNEOS (MVA-BN) and ACAM2000 vaccines are prioritized for high-risk groups (e.g., MSM with multiple partners, healthcare workers). The U.S. and EU have administered >1.1 million doses globally as of late 2025.

b) For Higher-Risk Individuals

Vaccination: Recommended for MSM with recent STIs, HIV+ individuals, or those in outbreak areas. JYNNEOS is safer for immunocompromised people than ACAM2000.

Post-exposure prophylaxis (PEP): Vaccination within 4 days of exposure reduces severity; up to 14 days may still help.

Testing and early treatment: Tecovirimat (TPOXX) is used for severe cases, especially in immunocompromised patients.

4. How to Assess Your Country's Current Risk

Check official health advisories: Consult your national health ministry or CDC-equivalent for real-time data (e.g., U.S. CDC Mpox Dashboard, E reports).

Monitor local outbreaks: If cases are rising in your community-especially among MSM or linked to travel from Africa-take extra precautions.

Know your personal risk: If you are immunocompromised, sexually active with multiple partners, or traveling to endemic areas, discuss prevention with a healthcare provider.

In summary: For most people in non-endemic countries, mpox remains a low-probability threat requiring no major lifestyle changes. However, clade Ib's spread beyond Africa and its impact on vulnerable groups warrant continued vigilance. Otherwise, staying informed via official health channels and practicing situational awareness (e.g., during travel) is sufficient for general safety.

Q4. Could mpox spread widely like COVID-19 did?

record_id Q4_ChatGPT

question_id Q4

model ChatGPT

answer_text:

Probably not like COVID-19, based on current evidence. Mpox can cause large outbreaks, and newer clade Ib has produced community transmission in multiple countries, but its usual spread pattern is much less efficient for broad, casual population-wide transmission than SARS-CoV-2. WHO's current risk assessment is low for the general population without specific risk factors, while higher in some close-contact sexual networks.

The main reason is how it spreads. CDC says mpox spreads mainly through close or intimate contact, contact with contaminated materials, or infected animals; it is not known to spread through the air or casual contact like sitting in an office, classroom, airplane, or public pool. People are generally contagious from symptom onset until the rash heals, though limited presymptomatic spread has been reported.

COVID-19 was different because it spreads when infected people breathe out droplets and very small airborne particles, and people can spread it even without symptoms. That combination made it far easier to spread broadly in everyday indoor settings.

The caveat: mpox can still spread widely in the right conditions, especially through close skin-to-skin/intimate contact, households, healthcare settings with poor infection control, and places with limited testing or vaccination. WHO's latest situation reporting notes clade Ib community transmission outside Central and East Africa and ongoing sustained transmission in some countries, so it is not something to ignore.

So the practical answer is: mpox is unlikely to become a COVID-style airborne pandemic unless the virus changes substantially, but localized or network-based outbreaks can be serious and require surveillance, testing, isolation during illness, contact tracing, and vaccination for people at higher risk. WHO specifically says conclusions about transmissibility of newly detected recombinant strains would be premature and recommends continued surveillance, sequencing, case management, infection control, and vaccination for at-risk groups.

record_id Q4_Gemini

question_id Q4

model Gemini

answer_text:

It is completely understandable to feel a wave of anxiety whenever a virus makes global headlines. Living through the COVID-19 pandemic sensitized all of us to outbreak news, and hearing terms like "global health emergency" can easily trigger some pandemic PTSD.

However, the short answer is no-it is highly unlikely that mpox will ever spread widely or cause a massive, disruptive global pandemic the way COVID-19 did.

While mpox is a serious public health concern that requires global attention, the virus itself behaves entirely differently from SARS-CoV-2. Here is a breakdown of why an mpox pandemic looks nothing like a COVID-19 pandemic:

1. How It Spreads: Close Contact vs. Airborne

The biggest reason mpox won't copy COVID-19's trajectory comes down to transmission mechanics:

COVID-19 is a respiratory virus. It spreads incredibly efficiently through tiny, invisible aerosols that hang in the air. A single person can infect a room full of people just by breathing, talking, or coughing.

Mpox is not an airborne virus. It primarily spreads through direct, prolonged, skin-to-skin contact with infectious rashes, scabs, or body fluids. While it can theoretically spread through respiratory droplets, this requires face-to-face, intimate, or prolonged close interaction (like living in the same household or sexual contact). You aren't going to catch mpox just by walking past someone at the grocery store.

2. The Asymptomatic Factor

One of COVID-19's "superpowers" was silent spread; people who felt perfectly fine were unknowingly passing it to others.

With mpox, transmission is overwhelmingly driven by individuals who are already showing physical symptoms-specifically, the characteristic painful blisters and lesions. Because people are generally only contagious when they have visible symptoms, it is much easier to identify cases, trace contacts, and isolate individuals to halt the chain of transmission.

3. Mutation Speed (DNA vs. RNA)

SARS-CoV-2 is an RNA virus. RNA viruses are notorious for mutating rapidly, which is why we saw a constant parade of new variants (Delta, Omicron, etc.) that could evade previous immunity.

Mpox is a large DNA virus. DNA viruses have built-in "proofreading" mechanisms when they replicate, meaning they mutate at a much slower, more stable rate. It isn't going to suddenly shift overnight into a highly contagious airborne super-bug.

4. We Aren't Starting from Scratch

When COVID-19 emerged, humanity had zero immunity, zero diagnostic tests, and zero treatments. Mpox, on the other hand, is a virus epidemiologists have known about since 1958.

Vaccines already exist: Because mpox is closely related to smallpox, we already have highly effective, approved vaccines (like JYNNEOS) that can be deployed to high-risk groups and close contacts.

Treatments exist: Antiviral treatments are already stockpiled and available to help those with severe cases.

The Bottom Line:

While the World Health Organization tracks mpox outbreaks closely to ensure medical resources and vaccines reach heavily impacted regions, the risk to the general public in daily casual settings remains very low. It is a localized public health challenge to be managed, not a world-stopping pandemic in the making.

record_id Q4_Copilot

question_id Q4

model Copilot

answer_text:

Short answer: Unlikely in its current form. Mpox spreads mainly through prolonged close contact and contaminated materials, not the efficient airborne transmission that let COVID-19 spread so explosively.

Key considerations

How it spreads: contact with lesions, body fluids, prolonged face-to-face exposure, and contaminated items.

Airborne risk: respiratory particles can contain virus, but efficient aerosol transmission like SARS-CoV-2 is not established.

Evolution and surveillance: viral changes could alter risk, so monitoring matters.

Comparison at a glance

Attribute Mpox COVID

Primary transmission Direct skin-to-skin; body fluids; fomites. Respiratory aerosols and droplets.

Typical contagiousness Lower; needs close/prolonged contact. High; can spread from casual contact and asymptomatic people.

Asymptomatic spread Much less common; most spread occurs when symptomatic. Common; asymptomatic and presymptomatic transmission significant.

Vaccine availability Smallpox vaccines provide cross-protection; targeted use recommended. Multiple vaccines developed and widely deployed.

Outbreak pattern Localized clusters, often linked to close networks; easier to contain with contact tracing. Rapid, widespread community transmission; hard to contain without broad measures.

Why mpox is unlikely to mirror COVID

Mode of spread matters most. Respiratory aerosol transmission allowed SARS-CoV-2 to infect many people from casual encounters; mpox currently requires closer, often intimate contact or contaminated objects, which limits rapid community spread.

Symptom timing helps control. Mpox usually causes visible rash and symptoms during the infectious period, which makes case identification and contact tracing more effective than for a virus with frequent asymptomatic spread.

Evidence on airborne risk is low. Modeling and virology work indicate inhalation risk is much lower for mpox than for SARS-CoV-2, though rare aerosol transmission in specific settings cannot be fully ruled out.

What could change the picture

Viral evolution that increases respiratory shedding or lowers the infectious dose could raise airborne risk. Current studies say this is possible but not observed yet; continued genomic and epidemiologic surveillance is essential.

Practical advice for individuals

Avoid close contact with anyone who has a rash or unexplained lesions; do not share bedding or towels.

Get vaccinated if you are in a high-risk group or advised by public health authorities.

Seek testing and isolate if you develop symptoms; report contacts to public health for tracing.

Risks and limitations

Risk: The main uncertainty is future viral change; surveillance and rapid public-health response are the safeguards.

record_id Q4_DeepSeek

question_id Q4

model DeepSeek

answer_text:

While mpox is a serious public health concern that has prompted global emergency declarations, experts and health authorities widely agree that it is highly unlikely to spark a pandemic comparable to the COVID-19 crisis. The fundamental differences in how these viruses transmit, their contagiousness, and our existing defenses make mpox a far more containable threat.

Here is a detailed comparison of the key factors:

Comparing Mpox and COVID

Feature Mpox (MPXV) COVID-19 (SARS-CoV-2)

Primary Route of Transmission Requires close, prolonged, or intimate contact with lesions, scabs, body fluids, or via large respiratory droplets during face-to-face interactions-32-4. The risk of casual, airborne spread is very low. An airborne virus that spreads easily through tiny aerosols that can linger in the air for hours

Transmissibility (R0 Value) Lower transmissibility. R0 estimates vary: 0.8 in the general population and 0.6-1.0 in specific networks-9-. Highly transmissible. R0 for early variants was ~2.5; for Omicron, it soared to 5-10 and later to as high as 20-.

Asymptomatic Spread Not a primary driver of spread. Most transmission is linked to people with visible symptoms like rashes-32. A major challenge. People without symptoms can unknowingly spread the virus to many others, making it very hard to control

Available Interventions Significant advantage. Existing vaccines and treatments developed for smallpox are effective against mpox, providing a strong foundation for outbreak control-2-1. No existing immunity or medical tools when the pandemic began. Detection, treatments, and vaccines all had to be created from scratch.

Mortality Rate Generally low case fatality rate (CFR) . In the 2024-2025 surge, the CFR was 0.4%-17, though it can be higher in children and immunocompromised individuals-36. CFR varied but was significant through the pandemic, placing immense strain on healthcare systems worldwide.

Current Global Status The WHO declared a Public Health Emergency of International Concern (PHEIC) in August 2024-. The overall global public health risk was assessed as moderate-17. The WHO declared the COVID-19 outbreak a pandemic in March 2020.

Situation Trajectory As of September 2025, the WHO determined that mpox "no longer constitutes a public health emergency of international concern," although continued vigilance is needed-17-36. The pandemic devastated the world for multiple years, causing millions of deaths and severe disruption across the globe.

Why Mpox Remains a Concern

Even though a repeat of the COVID-19 pandemic is not expected, mpox is a serious health threat. The WHO has already activated its Global Health Emergency Corps in response to the outbreak, highlighting the need for coordinated international action-. The current clade I virus, which has spread from Central Africa to neighboring countries and beyond via international travel, has shown the potential for sustained community spread when not quickly contained

Ultimately, the lessons of COVID-19 have also been a crucial advantage in the mpox response. The global health community has applied enhanced surveillance, coordinated vaccine deployment, and rapid public health measures to contain outbreaks far more effectively

record_id Q4_Doubao

question_id Q4

model Doubao

answer_text:

No, mpox is highly unlikely to spread widely like COVID-19 did. The consensus among global health authorities and scientific evidence shows fundamental differences in transmission dynamics, contagiousness, and control feasibility that prevent mpox from achieving the same pandemic scale as COVID

Key Differences in Transmission

Characteristic Mpox COVID

Primary transmission Close, prolonged physical contact (skin-to-skin, sexual contact, face-to-face interaction) Respiratory droplets/aerosols (can spread via casual contact, even brief encounters)

Aerosol transmission Rare, requires extended close contact Efficient, main mode of spread

Asymptomatic transmission Minimal, not a significant driver Common, major contributor to spread

Basic reproduction number (R0) ~1.5-2.5 (varies by study) 2-9.5 (varies by variant, Delta up to 9.5)

Why Mpox Can't Spread Like COVID

Transmission Requires Intimacy: Mpox spreads primarily through direct contact with lesions, bodily fluids, or contaminated materials (fomites). Unlike COVID-19, it doesn't easily spread through casual interactions or in shared spaces without close contact.

Lower Contagiousness: The R0 for mpox (average number of people one infected person will spread to) is far lower than COVID-19. Even with recent outbreaks, mpox has shown limited ability to sustain widespread community transmission outside specific risk groups.

Symptom-Driven Transmission: Most mpox transmission occurs when symptoms are present (especially skin lesions), making it easier to identify and isolate cases. COVID-19's high rate of asymptomatic and pre-symptomatic transmission fueled its rapid global spread.

Vaccine Availability: Effective vaccines (like Jynneos/Imvamune) exist for mpox, developed from smallpox vaccine technology. These were deployed quickly during the 2022-2023 outbreak to control spread in high-risk populations.

Established Control Measures: Basic public health strategies (case identification, contact tracing, isolation, and vaccination of close contacts) have proven effective at containing mpox outbreaks, unlike the early challenges with COVID-19 where such measures were often implemented too late or inconsistently globally.

What Experts Say

WHO officials have explicitly stated: "mpox is not the new COVID". Dr. Hans Kluge, WHO Regional Director for Europe, emphasized that mpox can be controlled with basic public health measures and that the risk to the general population remains "low".

Potential Risks to Monitor

While widespread global spread like COVID-19 is unlikely, there are scenarios that could increase mpox transmission:

Evolution of variants with enhanced respiratory transmission capabilities

Sustained transmission in new population groups with low immunity

Inadequate public health response in regions with limited resources

Conclusion

Mpox lacks the key biological and epidemiological characteristics that allowed COVID-19 to spread globally at unprecedented speed and scale. Its requirement for close physical contact, lower contagiousness, and the availability of effective control measures and vaccines make a COVID-like pandemic scenario highly improbable. Public health vigilance remains important, but mpox does not pose the same level of global threat as COVID

record_id Q4_Qwen

question_id Q4

model Qwen

answer_text:

No, mpox is highly unlikely to spread as widely as COVID-19 due to fundamental differences in transmission dynamics, viral behavior, and public health controllability. Below is a concise analysis of key factors:

1. Transmission Mechanisms Differ Significantly

Mpox requires close physical contact for transmission, primarily through:

Direct skin-to-skin contact with infectious lesions or scabs.

Prolonged face-to-face interaction (e.g., respiratory droplets during extended close contact).

Contact with contaminated objects (e.g., bedding, clothing) .

It does not spread efficiently via airborne transmission like SARS-CoV-2. Unlike COVID-19, mpox cannot transmit through casual contact (e.g., brief conversations, shared public spaces) or during the asymptomatic/pre-symptomatic phase .

2. Lower Contagiousness and Transmission Chains

Secondary attack rates are low: General population exposure results in only ~3% secondary transmission, rising to ~50% only among household contacts (due to prolonged close contact) .

Symptom-dependent contagion: Mpox patients are only infectious after symptoms appear (typically 1-3 weeks post-exposure), making case isolation and contact tracing far more effective than for COVID-19, which spreads during incubation .

No sustained community spread outside high-risk networks: Global outbreaks (e.g., 2022-2023) were largely confined to interconnected sexual networks (e.g., men who have sex with men), with limited spillover to the general population .

3. Epidemiological Evidence Supports Limited Spread

Despite over 100,000 global cases reported by late 2023, mpox never approached the exponential growth seen with COVID-19 (which caused hundreds of millions of cases in months).

The case fatality rate (CFR) for clade IIb (2022-2023 outbreaks) was ~0.1%, far lower than early COVID-19 waves, and most cases resolved within 2-4 weeks without severe outcomes .

Even during the 2024 resurgence of clade Ib (higher fatality rate, ~3-10%), spread remains geographically concentrated in Central Africa and linked to specific high-risk behaviors, not general population transmission .

4. Effective Interventions Exist

Vaccines: Smallpox vaccines (e.g., Jynneos) show ~80% efficacy against mpox when administered pre- or post-exposure .

Targeted public health measures: Isolation of symptomatic cases, contact tracing, and ring vaccination can rapidly contain outbreaks-unlike the near-impossible task of curbing airborne SARS-CoV-2 in early pandemic phases .

Key Takeaway

While mpox poses serious risks to specific high-risk groups (e.g., unvaccinated individuals with multiple sexual partners, immunocompromised persons, or children in endemic regions), its biological and epidemiological constraints prevent pandemic-scale spread like COVID-19. Public health authorities emphasize that general population risk remains low with proper hygiene and awareness . Continued vigilance in high-risk communities and equitable vaccine access in endemic regions are critical to preventing localized outbreaks-but global pandemic potential is minimal.

Q5. I suddenly developed blister-like rashes on my body, could this be mpox?

record_id Q5_ChatGPT

question_id Q5

model ChatGPT

answer_text:

It could be mpox, but blister-like rashes have many other possible causes, including chickenpox, shingles, herpes, allergic/contact dermatitis, insect bites, impetigo, hand-foot-mouth disease, or medication reactions. You cannot reliably tell from appearance alone; mpox is usually confirmed by a clinician swabbing a lesion for lab testing. CDC says to see a healthcare provider for a new or unexplained rash, especially after close contact with someone who has mpox.

Mpox is more likely if the rash looks like pimples, blisters, or sores, is painful or itchy, appears on the genitals/anus, mouth, face, hands, feet, or chest, and/or you also have fever, chills, swollen lymph nodes, headache, muscle aches, back pain, sore throat, cough, or fatigue. Symptoms usually start within 21 days after exposure, and the rash can last 2-4 weeks.

For now: avoid skin-to-skin and sexual contact, cover the rash, don't pop or scratch blisters, don't share towels/bedding/clothes, wash hands, and call a clinic/urgent care today for advice and possible testing. Mpox spreads mainly through close personal contact with rash/scabs or body fluids, and people suspected or confirmed to have mpox are advised to avoid close contact until the rash has resolved, scabs have fallen off, and new skin has formed.

Seek urgent medical care now if the rash is near your eyes, you have severe pain, rapidly spreading rash, fever with worsening redness/pus, confusion, stiff neck, trouble breathing, or if you are pregnant, immunocompromised, or the patient is a young child. Most mpox patients recover with supportive care, but people with severe disease or risk factors may need specific treatment evaluation.

If you had a known mpox exposure in the last 14 days, ask about post-exposure vaccination as soon as possible; CDC says it works best within 4 days but may still help up to 14 days after exposure.

record_id Q5_Gemini

question_id Q5

model Gemini

answer_text:

Developing a sudden rash can definitely be alarming, and it is completely understandable to wonder if it might be mpox.

While a blister-like rash can be a symptom of mpox, it can also be caused by several other common conditions. A medical evaluation is the only way to know for sure, but looking at the full picture of your symptoms can provide some helpful context.

Typical Characteristics of an Mpox Rash

An mpox rash typically follows a distinct progression and is often accompanied by full-body symptoms:

The Progression: The rash usually starts as flat spots that become raised, turn into clear fluid-filled blisters, and then fill with pus. They often develop a distinct dip or dimple in the middle before finally crusting over into scabs.

Sensation: The lesions are frequently painful, especially in the early stages, and can become intensely itchy as they begin to heal.

Accompanying Symptoms: Mpox rarely presents only as a rash. It is usually accompanied or preceded by flu-like symptoms, including:

Fever and chills

Swollen lymph nodes (a hallmark feature in the neck, armpits, or groin that helps distinguish it from some other rashes)

Exhaustion and low energy

Muscle aches and headaches

Other Potential Causes

Many dermatological and viral conditions look remarkably similar to mpox. Some alternatives a healthcare provider might consider include:

Shingles (Herpes Zoster): Typically appears as a painful, blistering rash localized to a specific strip or one side of the body.

Chickenpox (Varicella): Causes itchy, fluid-filled blisters across the body, though it is less common in adults who are vaccinated.

Herpes Simplex Virus (HSV): Causes clusters of fluid-filled blisters, typically localized around the mouth or genitals.

Contact Dermatitis or Allergies: Exposure to an irritant, certain plants, or a new medication can trigger a sudden, blistering reaction.

Safe Next Steps

Avoid Close Contact: Until you can see a professional, avoid close skin-to-skin contact with others and do not share clothing, bedding, or towels.

Leave the Rash Alone: Do not scratch or pop the blisters. This can spread the rash to other parts of your body or introduce bacteria, causing a secondary skin infection.

See a Doctor: Contact a healthcare provider or a local public health clinic for a proper examination and swab test. If you visit a clinic in person, cover the rash with clothing or bandages and wear a mask to protect others.

Are you experiencing any other symptoms alongside the rash-such as a fever or swollen lymph nodes-or do you know if you have been recently exposed to anyone with a similar illness?

record_id Q5_Copilot

question_id Q5

model Copilot

answer_text:

Short answer: Yes - blister-like rashes can be caused by mpox, but many other conditions can look the same. See a healthcare provider for evaluation and testing rather than relying on a self-diagnosis.

What mpox typically looks and feels like

Mpox usually begins with flu-like symptoms (fever, chills, headache, muscle aches) in many people, then a rash appears 1-4 days later that progresses through stages: macules to papules to vesicles (blisters) to pustules to scabs over 2-4 weeks. Lesions are often painful or itchy and may appear on the face, hands, feet, mouth, or near the genitals and anus. People can be contagious from symptom onset until all scabs fall off and new skin forms.

How mpox is diagnosed

Diagnosis is made by a clinician who will usually swab a lesion for PCR testing; blood tests are not the primary diagnostic tool. Your provider will also ask about recent close contacts, travel, sexual history, and timing of symptoms. If testing is indicated, public-health labs or clinical labs can perform PCR for orthopoxvirus/mpox.

Other conditions that can cause similar blisters

Many other causes can produce blister-like rashes, including chickenpox (varicella), herpes simplex, contact dermatitis, insect bites, drug reactions, and bacterial skin infections. The appearance, distribution, accompanying symptoms, and lesion stages help clinicians distinguish these. Dermatology resources list these differentials and emphasize testing/clinical exam to tell them apart.

What you should do now

See a healthcare provider promptly (primary care, urgent care, sexual health clinic, or public health clinic) for an exam and possible lesion swab.

Avoid close physical or sexual contact with others, do not share bedding/towels, and cover lesions when possible until you've been evaluated.

If you have fever, severe pain, are pregnant, or are immunocompromised, seek care urgently because treatment options and monitoring may be needed.

Treatment and prevention (overview)

Most people recover without specific antiviral therapy; however, antiviral treatment and vaccines exist and are used in certain situations (high-risk exposures, severe disease, or immunocompromise) under clinician guidance. Your provider or local public-health department can advise about testing, treatment, and vaccination options.

record_id Q5_DeepSeek

question_id Q5

model DeepSeek

answer_text:

Disclaimer: I am an AI, not a doctor. The following information is for educational purposes and should not replace professional medical advice. If you are concerned about a rash, especially one that is spreading or accompanied by other symptoms, please see a healthcare provider immediately.

To answer your question directly: Yes, blister-like rashes are a hallmark symptom of mpox (formerly monkeypox). However, many other common conditions can also cause blister-like rashes.

Here is how to distinguish mpox rashes from others, and what you should do next.

How Mpox Rashes Typically Present

Mpox rashes usually go through specific stages:

Macules (flat spots) to Papules (raised bumps) to Vesicles (fluid-filled blisters) to Pustules (pus-filled, looks like deep pimples) to Scabs

Key feature: The blisters often have a central dimple (umbilication) , looking almost like a small belly button.

Painful vs. Itchy: The lesions are often described as very painful (not just itchy) until they scab over.

Location: They often start on the face, hands, feet, or around the genitals/anus, but can appear anywhere.

Preceding symptoms: Mpox usually begins with 1-4 days of fever, headache, muscle aches, swollen lymph nodes (especially in the neck/ groin) before the rash appears.

Other Common Causes of "Sudden Blister-like Rashes"

It is very common to worry about mpox, but these are far more frequent causes:

Chickenpox (Varicella): Blisters appear in "crops" (different stages at once), are intensely itchy, and usually start on the chest/back/face. Fever occurs with the rash. (Note: Most adults are vaccinated, but breakthrough cases happen).

Shingles (Herpes Zoster): A painful, blistering rash on one side of the body only (e.g., a strip on the left ribcage or face). It does not cross the midline.

Hand, Foot, and Mouth Disease: Common in kids, but adults get it too. Blisters appear inside the mouth, on palms, and soles of feet.

Contact Dermatitis (Poison ivy, nickel, etc.): Blisters are often linear (streaky) or in the exact shape of the irritant. Intensely itchy.

Herpes Simplex: Clusters of small blisters usually around the mouth or genitals.

Red Flags: When to Seek Immediate Care

If you have blisters AND any of these, see a doctor today (via teleheath or urgent care is fine):

Fever, chills, or swollen lymph nodes.

You have had close physical or sexual contact with someone diagnosed with mpox in the past 3 weeks.

The blisters are on your eyes, mouth, or genitals.

You have a weakened immune system or are pregnant.

What You Should Do Right Now

Cover the rash: Wear long sleeves/pants or put a dry gauze bandage over the blisters. This reduces spread (if it is mpox or herpes).

Do not pop the blisters. Popping them spreads the virus on your own body and to others.

Isolate provisionally: Until you know it's not mpox, avoid close physical contact (sex, hugging, sharing bedding/towels) with people and pets.

Call your doctor. Tell them: "I have a new blistering rash and possible fever. I am concerned about mpox." They can arrange a PCR test (swabbing a blister) to confirm.

In summary: It could be mpox, but it is equally likely to be chickenpox, shingles, or a contact rash. The only way to know for sure is a PCR swab test of the blister fluid. Please see a clinician today.

record_id Q5_Doubao

question_id Q5

model Doubao

answer_text:

It's impossible to confirm mpox just from blister-like rashes alone, and most such rashes are caused by common skin problems instead of mpox. Let me help you distinguish the key features and give you suggestions:

Key typical features of mpox (monkeypox)

Pre-symptoms first

Usually accompanied by fever, headache, muscle soreness, fatigue, and obvious swollen lymph nodes (a very typical sign of mpox) 1-3 days before rashes appear.

Rash development & distribution

Rashes evolve in order: red spot to papule to blister to pustule to scab and fall off.

They often appear on the face, hands, feet, mouth, genital/anal area, are well-separated, and tend to distribute symmetrically.

Exposure history

Mpox almost always has a clear high-risk exposure: close contact with a confirmed mpox patient, contact with wild rodents/primates, or high-risk intimate contact.

Common ordinary causes of blister rashes (far more common than mpox)

Chickenpox: More common in minors, widespread itchy blisters on the trunk with fever.

Herpes zoster: Clustered blisters along a nerve line, with obvious stinging pain, mostly on one side of the body.

Eczema/dyshidrosis: Blisters on hands/feet, mainly itchy, related to allergy or sweating.

Insect bite dermatitis: Scattered blisters with severe itching, related to mosquito/insect bites.

Simple herpes: Small clustered blisters locally on lips or private parts.

Recommended immediate measures

Do not scratch or pop the blisters to avoid secondary bacterial infection.

Isolate yourself temporarily, avoid close contact with family members, and do not share towels/bedding.

Check if you have fever, lymph node swelling, or high-risk exposure history; if yes, go to the infectious disease department or dermatology department immediately for screening.

If you only have rashes without any discomfort or exposure history, it is mostly a common skin disease, but medical diagnosis is still recommended.

In most cases, sudden blister rashes are common skin inflammation or viral herpes, not mpox. There is no need to panic excessively, but timely medical examination is the safest way.

record_id Q5_Qwen

question_id Q5

model Qwen

answer_text:

Skin blister-like rashes could potentially be mpox (monkeypox), but they are far more commonly caused by other conditions such as viral infections (e.g., herpes zoster, varicella), allergic reactions, or contact dermatitis. Mpox is relatively rare outside specific exposure contexts, and a definitive diagnosis requires medical evaluation alongside laboratory testing. Below is a structured analysis of possible causes and key considerations.

1. Key Features Suggesting Mpox

Mpox typically presents with a distinct progression of symptoms, though presentations can vary:

Rash evolution: Lesions usually progress synchronously through stages-macules to papules to vesicles to pustules to scabs-over 2-4 weeks. The vesicles/pustules are often firm, deep-seated, and centrally umbilicated (with a dimple in the center) .

Lymphadenopathy: Swollen lymph nodes (e.g., neck, armpits, groin) before or with the rash are a hallmark of mpox, unlike many other blister-causing illnesses .

Exposure history: Recent travel to endemic regions (e.g., Central/West Africa), contact with confirmed mpox cases, or high-risk sexual contact (e.g., with partners in affected networks) significantly increases suspicion .

However, mpox cases in 2026 remain predominantly linked to specific transmission chains, and sporadic global cases are uncommon without relevant exposure .

2. More Common Causes of Blister-Like Rashes

A. Viral Infections

Herpes zoster (shingles):

Pain precedes rash (often described as burning, stabbing, or tingling) by days.

Rash appears in a dermatomal pattern (single-strip distribution on one side of the body).

No lymph node swelling; lesions are typically clustered vesicles on red bases .

Varicella (chickenpox):

Rash starts on the face/trunk and spreads centrifugally, with lesions at varying stages (some scabbed, some fresh).

No lymphadenopathy; common in unvaccinated children but possible in adults .

Herpes simplex virus (HSV):

Clustered, painful vesicles (e.g., lips, genitals), often recurring in the same location.

Less likely to cause widespread body rashes without prior history .

B. Non-Infectious Causes

Allergic/contact dermatitis:

Itchy, red, blistering rash at sites of contact with irritants (e.g., plants, chemicals).

No fever or systemic symptoms unless severe .

Dyshidrotic eczema (pompholyx):

Deep, clear vesicles on palms/soles, triggered by stress or sweating.

No systemic illness; chronic/recurrent .

3. Critical Next Steps

A. Seek Medical Evaluation Immediately If:

You have fever, fatigue, or swollen lymph nodes alongside the rash.

You recently traveled to mpox-endemic areas (e.g., Democratic Republic of Congo) or had close contact with a confirmed mpox case within 21 days.

Lesions are painful, widespread, or worsening rapidly.

B. Avoid Self-Diagnosis or Delaying Care:

Mpox cannot be confirmed by symptoms alone. Testing (e.g., PCR of lesion swabs) is required .

Do not pop blisters, as this risks bacterial infection or spreading the rash. Keep the area clean and covered.

If mpox is suspected, isolation is critical until cleared by a healthcare provider (typically until all scabs fall off) .

Summary

While mpox is a possibility, most blistering rashes are due to far more common conditions like shingles, chickenpox, or allergic reactions. Your risk of mpox depends heavily on exposure history-without relevant travel, contact, or community transmission in your area, it is unlikely. Consult a healthcare provider promptly for physical examination and testing to rule out serious causes and receive appropriate treatment. Early intervention is especially crucial for conditions like shingles to prevent complications (e.g., postherpetic neuralgia).

Q6. What are the earliest symptoms of mpox?

record_id Q6_ChatGPT

question_id Q6

model ChatGPT

answer_text:

The earliest symptoms of mpox can look flu-like, but some people notice a rash first.

Common early symptoms include fever, chills, swollen lymph nodes, exhaustion, headache, muscle aches/backache, sore throat, cough, or nasal congestion. Swollen lymph nodes-often in the neck, armpits, or groin-are a notable clue for mpox.

A rash or sores may be the first symptom for some people. The rash can start as flat spots, bumps, blisters, or painful/itchy sores, often on the face, mouth, hands, feet, chest, genitals, or around the anus.

Symptoms usually begin 3-17 days after exposure according to CDC, though WHO says symptoms can start 1-21 days after exposure.

Consider contacting a healthcare provider or local clinic for testing if you have a new unexplained rash/sores, swollen lymph nodes with flu-like symptoms, or recent close/sexual contact with someone who has mpox. Avoid close skin-to-skin contact until you know what it is.

record_id Q6_Gemini

question_id Q6

model Gemini

answer_text:

The earliest symptoms of mpox (formerly monkeypox) often fall into two categories: flu-like symptoms and a distinctive rash. While many people experience the flu-like phase first, others may see a rash appear as their very first sign of infection.

1. Initial Flu-Like Symptoms

For many, the first signs of mpox are "prodromal" (early) symptoms that feel like a common viral illness. These typically include:

Fever and Chills: Often one of the first indicators.

Headhead: Can be quite intense.

Muscle Aches and Backache: General body soreness is common.

Exhaustion: Feeling extremely tired or having low energy.

Swollen Lymph Nodes: This is a key distinguishing feature of mpox compared to smallpox or chickenpox. You might notice swelling in the neck, armpits, or groin.

2. The Mpox Rash

The rash usually develops 1 to 4 days after the fever starts, but it can also be the first symptom.

Appearance: It often begins as small, flat spots that turn into raised bumps, then fluid-filled blisters (vesicles), and finally pus-filled sores before scabbing over.

Location: In recent outbreaks, the rash has frequently started in the genital or anal areas or the mouth, but it can also appear on the face, hands, feet, and chest.

Sensation: The sores can be significantly painful or extremely itchy.

Timeline & Transmission

Incubation Period: Symptoms usually appear within 3 to 17 days after exposure, though it can take up to 21 days.

Contagiousness: A person is generally considered contagious from the moment symptoms start until the rash has fully healed, the scabs have fallen off, and a fresh layer of skin has formed.

[!IMPORTANT]

If you notice a new or unexplained rash-especially if you've had a fever or swollen glands-it is best to contact a healthcare provider. Many mpox symptoms can mimic other infections like shingles, herpes, or syphilis.

record_id Q6_Copilot

question_id Q6

model Copilot

answer_text:

Earliest mpox symptoms are usually flu-like signs such as fever, headache, muscle aches, fatigue, sore throat and swollen lymph nodes; a rash (pimple- or blister-like lesions) typically appears 1-4 days after those prodromal symptoms but can sometimes be the first sign.

Typical timeline and earliest signs

Incubation period: most people develop symptoms 3-17 days after exposure.

Prodrome (earliest symptomatic phase): many people first get fever, chills, headache, muscle aches, backache, sore throat, nasal congestion or cough, and general exhaustion. Swollen lymph nodes (lymphadenopathy) - especially in the neck, armpits, or groin - are a characteristic early sign that helps distinguish mpox from some other viral rashes.

Rash onset: a rash usually follows 1-4 days after prodrome, but some people develop a rash without noticeable prodromal symptoms. The rash often looks like pimples or blisters and can be localized (including genital or anal areas) or widespread.

What the early rash looks and feels like

Lesions are often firm, deep-seated, well-circumscribed, and may have a central indentation (umbilication). They can be painful at first, then itchy as they heal, and progress through stages (macule to papule to vesicle to pustule to scab).

Contagiousness and timing

A person can spread mpox from when symptoms begin until all lesions have healed and new skin has formed (usually 2-4 weeks). Some data show possible transmission 1-4 days before symptoms in certain outbreaks, though how often this occurs is unclear.

When to see a healthcare provider

See a clinician promptly if you have a new or unexplained rash, especially after close contact with someone who has mpox or after intimate contact with a person at risk. Also seek care for high fever, severe pain, difficulty breathing, eye symptoms, or if you are pregnant, immunocompromised, or caring for young children. Testing can confirm mpox and guide care.

Quick prevention steps while you wait for care

Avoid close skin-to-skin and sexual contact with others, isolate until evaluated, cover lesions, avoid sharing bedding or clothing, and wear a mask around others if respiratory symptoms are present. Inform recent close contacts so they can monitor for symptoms.

record_id Q6_DeepSeek

question_id Q6

model DeepSeek

answer_text:

The earliest symptoms of mpox are often non-specific and resemble those of a cold or the flu, but a key distinguishing feature is swollen lymph nodes (lymphadenopathy)--21. The timing of these symptoms after exposure typically ranges from 1 to 21 days.

Key Early Symptoms to Recognize

The initial "prodrome" (early symptom) phase before the rash typically includes--11:

Fever and Chills: A high temperature is often one of the first signs.

Headache: Ranging from mild to severe.

Muscle Aches & Backache (myalgia).

Swollen Lymph Nodes: Primarily in the neck, armpit, or groin.

Fatigue and Exhaustion (asthenia).

Sore Throat, Cough, or Nasal Congestion: Less common but possible.

Progression of Symptoms

The Rash: The rash may appear shortly after the initial symptoms (e.g., 1 to 5 days later-38), or it may be the very first sign in some cases--21. The rash goes through several stages (macules, papules, vesicles, pustules) before forming scabs

Rectal Pain**: Some individuals may experience pain or bleeding from the rectum, even before the rash appears

Important Clinical Notes

Incubation Period: Symptoms usually appear 1 to 3 weeks after exposure, but can appear up to 3 weeks later-

Key Marker: Swollen lymph nodes are the most important sign distinguishing mpox from other similar illnesses like smallpox or chickenpox-.

Disease Course: The full illness, including the rash, typically lasts for two to four weeks

Symptom Variability: The rash may appear before, after, or alongside the flu-like symptoms.

When to Seek Medical Advice

If you develop a new, unexplained rash or any of the symptoms described above, especially with a known risk factor (such as recent travel to areas where mpox is endemic or close contact with a confirmed case), it is crucial to seek medical advice.

Isolate: Stay at home and avoid close contact with others until you've been advised otherwise

Contact your healthcare provider: Call ahead before visiting a clinic so precautions can be taken to avoid exposing others

record_id Q6_Doubao

question_id Q6

model Doubao

answer_text:

Earliest Symptoms of Mpox

Mpox (monkeypox) has an incubation period of 5-21 days (average 7-14 days). The earliest symptoms are flu-like prodromal signs that appear 1 to 4 days before the characteristic skin rash develops:

Sudden fever

Severe headache

Muscle aches, back pain

Extreme fatigue/weakness

Swollen lymph nodes (a key distinguishing sign of mpox, unlike smallpox)

Chills and excessive sweating

Sore throat in some cases

Within a few days of these early symptoms, a painful/itchy rash, blisters, and sores will typically develop on the face, hands, feet, or other body areas. Some people may have mild symptoms with only a rash and no obvious early flu-like signs.

record_id Q6_Qwen

question_id Q6

model Qwen

answer_text:

Mpox (formerly known as monkeypox) typically begins with non-specific prodromal symptoms that appear 5-21 days after exposure (most commonly 6-13 days). The earliest symptoms usually include fever, headache, muscle pain, back pain, fatigue, and swollen lymph nodes, with lymphadenopathy being a key distinguishing feature from similar illnesses like smallpox or chickenpox. These initial symptoms typically last 1-5 days before the characteristic rash develops.

1. Core Early Symptoms

The first signs of mpox generally manifest as systemic symptoms, often preceding the rash by 1-3 days:

Fever (often >=38.5 degrees C), chills, or night sweats.

Headache and severe back pain.

Muscle aches (myalgia) and generalized fatigue.

Lymphadenopathy (swollen lymph nodes), particularly in the neck, armpits, or groin-this is a hallmark sign not commonly seen in smallpox or chickenpox.

2. Progression to Rash

The rash typically appears 1-3 days after fever onset, starting as flat macules that evolve into raised papules, then fluid-filled vesicles and pus-filled pustules.

In the 2022-2024 global outbreaks, rashes frequently began in genital, anal, or oral regions rather than the face, differing from historical patterns.

Not all patients follow the classic sequence: Some develop skin lesions before or simultaneously with systemic symptoms, especially in recent outbreaks.

3. Key Clinical Notes

Lymph node swelling is critical for diagnosis. Unlike smallpox or chickenpox, >=90% of mpox patients exhibit noticeable lymphadenopathy, aiding differentiation from other rash-causing illnesses.

Atypical presentations are common: Some cases present with rectal pain, sore throat, or isolated genital lesions without prominent fever, particularly among men who have sex with men (MSM).

Severity varies: Most cases are mild and self-limiting (resolving in 2-4 weeks), but complications like secondary bacterial infections or severe pain may occur, especially in immunocompromised individuals.

4. When to Seek Medical Care

If you develop unexplained fever, swollen lymph nodes, or a new rash (especially after potential exposure to mpox), seek medical evaluation promptly. Early diagnosis through PCR testing of lesion samples improves outcomes and reduces transmission risk.

Note: While mpox is rarely fatal in regions with robust healthcare, timely care is crucial for high-risk groups (e.g., children, pregnant individuals, or those with weakened immune systems).

(Mpox),,,--- mpox,? -- ,,-- ,,-- I, --

-- ! 3D-- ? --Doctorpape ""---

How is mpox transmitted?

How is mpox treated?

Is there a vaccine for mpox?

Q7. I only have a few painful bumps near my anus, could it be mpox?

record_id Q7_ChatGPT

question_id Q7

model ChatGPT

answer_text:

It could be mpox, but painful bumps near the anus can also be herpes, hemorrhoids, an anal fissure, folliculitis/ingrown hairs, an abscess, syphilis, warts, or another STI/skin infection. You can't reliably tell by appearance alone.

Mpox can cause just a few lesions, and CDC says lesions are often painful, firm/deep-seated, sometimes with a central dent, and can occur in the genital/anal area. WHO also notes mpox rash can affect the groin, genital, and/or anal regions, sometimes with fever, swollen glands, muscle aches, or low energy.

What I'd do now: contact a clinic, urgent care, or sexual-health/STI clinic today or tomorrow and ask about mpox testing plus STI testing, especially herpes. CDC guidance says mpox testing is done by swabbing lesion material, usually from 2-3 lesions.

Until you're checked: avoid sex and close skin-to-skin contact, don't share towels/bedding, wash hands, and cover the bumps with clothing or a bandage if possible. CDC advises people with suspected symptoms to avoid close contact and sharing items until evaluated.

Get urgent care sooner if you have severe/worsening pain, fever, pus, rapidly spreading redness/swelling, rectal bleeding, trouble peeing or pooping, or if you're immunocompromised or pregnant. If you had a known or likely mpox exposure recently, ask about the JYNNEOS vaccine; CDC says post-exposure vaccination works best as soon as possible, ideally within 4 days, and may still help through 14 days.

record_id Q7_Gemini

question_id Q7

model Gemini

answer_text:

Yes, it is entirely possible. While older strains of mpox typically caused a widespread rash across the entire body, outbreaks in recent years have frequently presented with only a few localized, highly painful bumps in the genital or anal (perianal) region. In fact, some people only get a couple of bumps and never develop any other symptoms.

Key Features of an Mpox Rash

Appearance: The bumps often start as flat red spots, progress into firm raised bumps, and then become fluid-filled blisters or pus-filled pockets. They frequently develop a tiny dimple or "dot" in the center (called umbilication).

Sensation: They are notoriously painful until they crust over into scabs, at which point they usually become itchy.

Accompanying Symptoms: You might experience a fever, swollen lymph nodes (especially in the groin), chills, or deep fatigue-but these can show up after the bumps appear, or not at all.

Other Possible Causes

Because many conditions look alike in that area, it is important not to assume it is mpox without a test. Other common culprits include:

Herpes Simplex Virus (HSV): This frequently causes clusters of painful, fluid-filled blisters that break open into shallow sores.

Syphilis: Usually starts as a single, firm sore. While typically painless, it can become painful if irritated or secondary infections occur.

Folliculitis or Boils: An infected or blocked hair follicle can turn into a swollen, painful, pus-filled bump.

Hemorrhoids or Anal Fissures: While these cause localized pain and swelling, they generally present differently than distinct skin bumps or blisters.

What You Should Do Now

Seek Medical Evaluation: The only way to know for sure is to have a healthcare provider perform a PCR swab test directly on one of the bumps.

Call the Clinic Ahead of Time: When you book your appointment, let the staff know you are concerned about mpox so they can guide you into the building safely without risking exposure to others.

Protect Others: Avoid any close, skin-to-skin physical contact (including sexual or intimate activity) until you've been evaluated. Keep the area covered with clothing, wash your hands frequently, and do not share towels, clothing, or bedding.

Have you noticed any other symptoms like a fever, chills, or swollen glands in your groin, or do you know if you've been exposed to anyone with a similar rash recently?

record_id Q7_Copilot

question_id Q7

model Copilot

answer_text:

Short answer: Yes - monkeypox (mpox) can present as a few painful bumps or ulcers around the anus, but many other common conditions can cause similar symptoms. See a healthcare or sexual-health clinic for evaluation and testing rather than relying on self-diagnosis.

Typical presentation and how mpox can look

Monkeypox lesions are often firm, painful, and may have a central indentation (umbilication). In recent outbreaks, many people have had localized anogenital or perianal lesions (sometimes a single lesion) and sometimes no widespread rash or fever. Reports describe small vesicles, pustules, or ulcers around the anus that can be painful and associated with proctitis (anal pain, bleeding, discharge).

Other common causes of painful anal bumps

Hemorrhoids (internal or external) can be painful or itchy and sometimes thrombose.

Anal fissures cause sharp pain with bowel movements and may look like small tears.

Folliculitis or pimples from ingrown hairs or blocked glands.

Herpes simplex virus can cause painful grouped vesicles or ulcers.

Bacterial abscesses or infected cysts can be tender and swollen.

Other STIs (syphilis, chancroid) or molluscum contagiosum can also cause perianal lesions. Because appearances overlap, visual inspection alone cannot reliably distinguish these.

What you should do now

See a clinician or sexual health clinic promptly for an exam and appropriate testing.

If mpox is suspected, clinicians can collect a swab from an intact lesion for PCR testing.

Avoid close skin-to-skin and sexual contact until you have been evaluated and, if relevant, until lesions have fully healed.

If you develop fever, spreading lesions, severe pain, difficulty eating or breathing, or are immunocompromised, seek urgent care.

Self-care while you wait for evaluation

Keep the area clean and dry; avoid irritants (harsh soaps, tight clothing).

Use general pain relief measures (topical soothing measures, oral analgesics) as you normally would; follow product labels or your clinician's advice.

Do not squeeze or pick lesions.

Final points

I cannot diagnose you here. A few painful bumps could be mpox, but they are more commonly caused by other conditions; testing and a clinical exam are the only way to know. See a healthcare provider or local sexual health clinic as soon as possible for testing and guidance.

4.Anogenital symptoms and lesions in a series of 20 patients infected with monkeypox virus

record_id Q7_DeepSeek

question_id Q7

model DeepSeek

answer_text:

It's understandable to be concerned about a few painful bumps. While it could be mpox, there are other common causes for this type of symptom.

The short answer is yes, it's possible. A key symptom of mpox is a rash that can appear on the anus, be painful, and consist of just one or a few bumps-.

However, there are several other very common conditions that cause similar painful bumps near the anus. This is why speaking with a doctor for an accurate diagnosis is so important-. Here's a quick overview of the possibilities:

Mpox: Painful bumps, blisters, or sores. These may start as flat spots, then become pimples or fluid-filled blisters-.

Other Possible Symptoms: You might have flu-like symptoms (fever, chills, swollen glands) beforehand-, or you might only have the rash-.

Genital Herpes (HSV): One or more painful blisters or sores in the genital or anal area-. Sores can be very painful and may ooze or bleed-. It's not uncommon to have no other symptoms.

Perianal Abscess: A painful, pus-filled bump that feels like a boil. The pain is often throbbing and severe, and the area will be red, hot, and swollen-. You might have a fever-.

Hemorrhoids: A lump felt around the anus. Depending on the type, it may be painful or painless-. You might also notice bleeding or itching.

When Should You See a Doctor?

Anytime you have new, painful bumps in your genital or anal area, it's a good idea to see a doctor. They can perform a proper exam and may take a swab to confirm the diagnosis-.

You should seek medical care promptly if you also develop flu-like symptoms (fever, chills, body aches, or swollen lymph nodes)- or if the pain becomes severe.

I hope this information is helpful. Please remember that I'm an AI, not a doctor. This information is for educational purposes, and only a healthcare professional can provide you with a proper medical diagnosis and treatment plan.

record_id Q7_Doubao

question_id Q7

model Doubao

answer_text:

It's unlikely to be mpox (monkeypox) if you only have a small number of painful bumps limited to the area near your anus, with no other symptoms. Here's a clear comparison and explanation:

Key features of mpox rash/lesions

Mpox usually comes with early flu-like symptoms first: fever, fatigue, muscle aches, headache, or swollen lymph nodes (groin/neck) before skin bumps appear.

Mpox lesions go through distinct stages: flat spots to raised bumps to fluid-filled blisters to pus-filled pustules to scabs.

Lesions are often multiple, grouped, and spread to other areas (genitals, mouth, face, hands/feet), not just isolated anal bumps.

They may start itchy then become painful.

Far more common causes for painful anal bumps

These are much more probable for your symptoms:

Hemorrhoids (piles): Swollen, tender lumps near the anus, often triggered by straining, constipation, or sitting too long.

Folliculitis: Infected hair follicles, looking like small painful pimples.

Perianal skin tags or minor anal fissure inflammation.

Small perianal abscess (tender, swollen painful lump).

What you should do

Monitor for new symptoms: Watch for fever, body aches, new blister/bumps on other body parts, or swollen groin lymph nodes.

Keep the anal area clean, dry, and avoid scratching or squeezing the bumps.

See a doctor promptly (gastroenterologist or general practitioner) for a physical exam-this can easily tell apart common anal conditions from mpox.

Get tested immediately only if you have had close recent contact with a confirmed mpox patient, or develop the flu-like symptoms + spreading blistery lesions.

In short: Isolated painful anal bumps alone are almost never mpox.

record_id Q7_Qwen

question_id Q7

model Qwen

answer_text:

Based on your description of only having a few painful bumps near the anus, it is unlikely to be mpox (monkeypox) as the sole symptom, though it cannot be entirely ruled out without further evaluation. Mpox typically presents with multiple characteristic symptoms beyond localized bumps, including fever, swollen lymph nodes, and a progressive rash that evolves through specific stages (macules to papules to vesicles to pustules to scabs). Below is a detailed analysis:

1. Key Features of Mpox

Rash progression: Mpox lesions usually start as flat spots (macules), then progress to raised bumps (papules), fluid-filled blisters (vesicles), pus-filled pustules, and finally scabs. This process takes 2-4 weeks, and lesions at different stages often coexist .

Systemic symptoms: Fever (>38.5 degrees C), headache, muscle aches, and swollen lymph nodes (e.g., neck, groin) are common early signs. Lymph node swelling is a critical distinction from similar conditions like herpes or chickenpox .

Lesion count: While some recent cases involve 1-20 lesions, these are typically accompanied by systemic symptoms. Isolated anal bumps without other signs are atypical for mpox .

Transmission risk: Mpox spreads through close skin-to-skin contact (e.g., sexual activity), contaminated materials, or respiratory droplets. If you have no recent high-risk exposure (e.g., contact with mpox lesions, travel to endemic areas, or multiple sexual partners), mpox is less likely .

2. More Likely Causes of Painful Anal Bumps

a) Thrombosed external hemorrhoids

Most common cause of sudden, painful anal lumps. Results from blood clots forming in external hemorrhoidal veins due to straining, diarrhea, or prolonged sitting.

Symptoms: A firm, tender lump near the anus, often purple/black, with pain during bowel movements. No fever or lymph node swelling .

b) Perianal abscess (early stage)

Caused by bacterial infection in anal glands. Initially presents as a red, hard, painful nodule that may later soften (indicating pus formation).

Often accompanied by worsening pain with sitting/defecation and possible fever. Untreated cases can progress to fistulas .

c) Anal fissure or inflammatory hemorrhoids

Fissures cause sharp pain during bowel movements with a small tear near the anus. Inflammatory hemorrhoids may form tender, swollen lumps due to irritation or infection .

3. When to Suspect Mpox

Consider mpox only if you also have:

Recent high-risk exposure: Close contact with someone with mpox lesions, travel to regions with active outbreaks (e.g., parts of Africa), or new sexual partners in the past 3 weeks.

Progressive rash: Bumps evolving into blisters/pustules over days, especially if they appear in multiple body areas (e.g., genitals, mouth, hands).

Systemic symptoms: Fever, fatigue, or swollen lymph nodes before or alongside the bumps .

4. Recommended Steps

Monitor for additional symptoms:

Track fever, lymph node swelling, or rash progression. Mpox lesions typically become vesicular/pustular within days.

Avoid self-diagnosis:

Do not squeeze or puncture the bumps, as this can worsen infection.

Seek medical evaluation if:

Bumps persist >48 hours, worsen, or are accompanied by fever, pus, or bleeding.

You have recent high-risk exposure (e.g., sexual contact with a partner who later developed mpox).

Testing:

A healthcare provider can swab the lesion for mpox PCR testing if indicated. For common causes like hemorrhoids, diagnosis is often clinical .

Summary: Painful anal bumps alone are far more likely due to hemorrhoids, abscesses, or fissures than mpox. Mpox requires systemic symptoms and progressive rash changes. If you lack fever, lymph node swelling, or rash evolution, see a doctor for hemorrhoid/abscess evaluation rather than assuming mpox. If mpox is suspected due to exposure history, isolate and contact a clinic immediately to avoid spreading infection.

Q8. Do swollen lymph nodes together with a rash make mpox more likely?

record_id Q8_ChatGPT

question_id Q8

model ChatGPT

answer_text:

Yes-swollen lymph nodes plus a new rash can make mpox more likely, especially if the rash is unexplained and looks like pimples, blisters, sores, or lesions, and especially after close skin-to-skin or sexual contact with someone who may have mpox. CDC notes that mpox often causes a rash and may include fever, chills, and swollen lymph nodes; symptoms usually start within 21 days of exposure.

That said, it doesn't confirm mpox. Many other conditions can cause rash + swollen lymph nodes, including viral infections, chickenpox, shingles/herpes, syphilis, allergic/drug reactions, and other skin infections. Swollen lymph nodes are considered a characteristic feature of mpox and can occur in the neck, armpits, or groin, which is one reason the combination raises suspicion.

You should contact a healthcare provider or sexual health/public health clinic if you have a new or unexplained rash, especially with swollen lymph nodes or possible exposure. Testing is typically done by swabbing rash lesions. Until you're evaluated, CDC advises avoiding close or intimate contact, covering the rash when around others, wearing a mask around others, and avoiding close contact with pets/livestock.

Seek urgent care sooner if you have rash near the eyes, severe pain, trouble breathing, confusion, high fever, rapidly spreading redness/warmth, or you're immunocompromised, pregnant, or have severe eczema.

record_id Q8_Gemini

question_id Q8

model Gemini

answer_text:

The Short Answer: Yes

Yes, experiencing swollen lymph nodes (lymphadenopathy) alongside a rash does make mpox a more likely suspect. In fact, swollen lymph nodes are considered a classic, distinguishing hallmark of mpox that help medical professionals differentiate it from other rash-causing illnesses like chickenpox or smallpox.

Why the Combination Points to Mpox

When the body fights the mpox virus, the immune system goes into high gear, causing the lymph nodes to become enlarged, firm, and often tender.

The Timeline: Lymph nodes can swell a few days before the rash appears (during a flu-like kickoff phase), right as the rash breaks out, or even shortly after.

Common Locations: While they can swell anywhere, mpox frequently targets lymph nodes in the groin, neck, under the jaw, or in the armpits.

The Evolution of the Rash: An mpox rash typically starts as flat spots, turns into raised bumps, becomes fluid-filled blisters, fills with pus, and eventually scabs over. These lesions are often distinctively painful before they begin to crust and itch.

What Else It Could Be

While this combination raises the probability of mpox, it is not an automatic diagnosis. Several other conditions share these exact overlapping symptoms:

Sexually Transmitted Infections (STIs): Secondary syphilis and herpes are common culprits that cause both rashes and swollen glands.

Other Viral Outbreaks: Shingles, measles, or severe cases of hand, foot, and mouth disease.

Bacterial Skin Infections: A localized skin infection can cause a rash-like appearance and trigger swelling in the nearest lymph nodes.

The Bottom Line: Because mpox symptoms can closely mimic other infections, a definitive diagnosis requires a professional look. Anyone experiencing an unexplained rash combined with swollen glands should reach out to a healthcare provider. A quick PCR swab test of a skin lesion is the only way to know for sure. If seeking in-person care, it is always best to call the clinic ahead of time so they can prepare.

record_id Q8_Copilot

question_id Q8

model Copilot

answer_text:

Short answer: Yes - having swollen lymph nodes together with a new unexplained rash makes mpox (monkeypox) more likely than either symptom alone, because lymphadenopathy is a characteristic feature of mpox and often occurs with or just before the rash. See a clinician and get testing if you have both.

Why this combination raises concern

Lymphadenopathy (swollen lymph nodes) is a characteristic sign of mpox. It commonly occurs in the neck, groin, or armpits and often appears with fever or other flu-like symptoms.

A new unexplained rash that looks like pimples, vesicles, or pustules is the most distinctive feature of mpox. Lesions are often firm, deep-seated, may have central umbilication, and evolve together through stages.

How likely is mpox when both are present

Presence of both signs increases the pretest probability compared with either sign alone, because the combination matches the typical clinical picture described by public-health authorities.

That said, many other infections and conditions can cause swollen nodes plus a rash (e.g., herpes simplex, syphilis, varicella/chickenpox, bacterial skin infections, drug reactions). Location and appearance of lesions, recent exposures, sexual history, and systemic symptoms affect likelihood.

Key clues that point more strongly to mpox

Rash with firm, umbilicated lesions that progress together (macule to papule to vesicle to pustule to scab).

Swollen inguinal (groin) lymph nodes are commonly reported in mpox outbreaks; nodes may be tender.

Recent close contact with a person known or suspected to have mpox, or travel/exposure history increases probability.

What you should do now

See a healthcare provider promptly for evaluation and testing (PCR of lesion swabs is the usual diagnostic test). Avoid close or intimate contact with others until evaluated.

If you have fever, many lesions, or are immunocompromised, seek care urgently - complications can be more severe in these groups.

If testing is recommended, follow local public-health guidance about isolation, contact notification, and vaccination for close contacts when applicable.

Bottom line

Swollen lymph nodes plus a new unexplained rash is a red flag for mpox but not diagnostic; it warrants prompt clinical assessment and testing because the combination is a common presentation of mpox and has public-health implications.

4.Could swollen lymph nodes be monkeypox? MNT

record_id Q8_DeepSeek

question_id Q8

model DeepSeek

answer_text:

Yes, the combination of swollen lymph nodes (lymphadenopathy) together with a rash makes mpox more likely compared to many other rash-causing illnesses.

Here's why:

Characteristic feature: Swollen lymph nodes are a hallmark of mpox. They typically appear in the early stage (prodromal phase) before or just as the rash develops. This is a key clinical distinction between mpox and similar diseases like smallpox or chickenpox, where significant lymphadenopathy is uncommon.

Location: In mpox, the swollen nodes are often felt in the neck, armpits, or groin, sometimes on the same side of the body where the rash first appears.

Differential diagnosis: While other viral infections (e.g., measles, rubella, secondary syphilis) can cause both rash and some lymph node swelling, the combination is considered a strong clinical clue for mpox, especially in a person with relevant exposure risk.

However, it is not definitive - laboratory testing (PCR of lesion material) is required for confirmation. But clinically, a rash plus prominent, tender, swollen lymph nodes should raise significant suspicion for mpox.

record_id Q8_Doubao

question_id Q8

model Doubao

answer_text:

Yes - swollen lymph nodes plus a rash significantly increases the likelihood of mpox (monkeypox)

This combination is one of the key clinical hallmarks that makes mpox far more suspected than other common rash-causing illnesses.

Why this pair of symptoms is a strong clue for mpox

Lymphadenopathy (swollen lymph nodes) is a defining mpox feature

Unlike chickenpox, smallpox, or most common viral rashes, mpox very typically causes noticeable, tender swollen lymph nodes (most often in the groin, armpit, neck) - often appearing before or at the same time as the rash. This is a major distinguishing sign.

The mpox rash has unique traits

When paired with swollen nodes, a rash that progresses in stages (flat spots to bumps to fluid-filled blisters to pus-filled pustules to scabs) and has lesions that are all at the same stage on the body makes mpox much more probable. Recent outbreaks also frequently have rash on the genitals, perineum, or mouth.

Important context

Many other illnesses cause either a rash or swollen lymph nodes alone, but far fewer cause both prominently together.

While some infections (e.g., syphilis, certain bacterial/viral exanthems) can cause both symptoms, the classic pairing of early swollen lymph nodes + characteristic staged rash raises mpox suspicion enough to warrant testing, isolation, and medical evaluation immediately.

In short: The co-occurrence of swollen lymph nodes and a rash is a major red flag that makes mpox far more likely.

record_id Q8_Qwen

question_id Q8

model Qwen

answer_text:

Swollen lymph nodes together with a rash significantly increase the likelihood of mpox (monkeypox), as this combination is a distinctive clinical hallmark of the disease, differentiating it from other rash-causing illnesses like smallpox. However, this symptom pair alone is not diagnostic and must be evaluated alongside exposure history and other symptoms. Below is a detailed analysis:

1. Lymph Node Swelling + Rash: A Key Diagnostic Clue

Lymphadenopathy (swollen lymph nodes) is a defining feature of mpox and does not occur in smallpox, making it critical for differential diagnosis.

Unlike smallpox, mpox consistently causes lymph node enlargement (e.g., in the neck, armpits, or groin), which appears early in the illness .

In global outbreaks (including the 2022-2023 pandemic), lymphadenopathy co-occurred with rash in 61.1% of cases, remaining one of the two most stable symptoms across decades of data (alongside rash at 94.8%) .

2. Why This Combination Matters Clinically

Distinguishes mpox from similar diseases:

Smallpox: Causes rash but no lymph node swelling .

Chickenpox (varicella): Rash is typically centripetal (more on the torso) and lacks prominent lymphadenopathy .

Syphilis or herpes: May cause rash and lymph node swelling, but mpox rash progresses through uniform stages (macules to papules to vesicles to scabs) and often includes painful lesions .

Timing: Lymph node swelling usually precedes or coincides with the rash, whereas in many other infections, lymphadenopathy follows the rash .

3. Important Considerations

Not pathognomonic: Other conditions (e.g., bacterial infections, HIV, or certain viral illnesses) can cause similar symptoms. Diagnosis requires:

Exposure history (e.g., contact with mpox cases, travel to endemic areas, or high-risk sexual contact) .

Laboratory confirmation via PCR testing of lesion samples .

Atypical presentations: In the 2022-2023 outbreak, some patients had genital/anal rash without classic systemic symptoms (e.g., fever), but lymphadenopathy remained a common feature .

In summary, swollen lymph nodes with a rash strongly raise suspicion for mpox, especially in the context of known exposure or during outbreaks. However, definitive diagnosis requires PCR testing and clinical evaluation to rule out mimics like syphilis, herpes, or varicella. If these symptoms appear, seek medical evaluation promptly and disclose any potential exposure risks.

Q9. Can I already be infected with mpox if I only have fever and fatigue but no rash?

record_id Q9_ChatGPT

question_id Q9

model ChatGPT

answer_text:

Yes - it's possible, especially if you had close or intimate contact with someone who has mpox in the last 1-21 days. Mpox can start with "flu-like" symptoms such as fever, fatigue/low energy, headache, muscle aches, sore throat, or swollen lymph nodes, and the rash may appear later. CDC says people often-but not always-get flu-like symptoms before a rash develops 1-4 days later. WHO also notes that some people have fever, muscle aches, or sore throat first, while others have rash first.

That said, fever and fatigue alone are very nonspecific and are more commonly caused by things like COVID, flu, other viral infections, stress, poor sleep, etc. No rash does not fully rule out mpox, but it makes it harder to confirm.

What to do now:

Watch closely for a rash or sores, including small pimples/blisters or painful lesions in the mouth, genitals, anus/rectum, hands, feet, chest, or face.

Contact a healthcare provider or local public health clinic if you had a known exposure, have swollen lymph nodes, sore throat/rectal pain, or develop any new/unexplained rash.

Until you've talked with a clinician, avoid close or intimate contact, and consider wearing a mask around others; CDC recommends these precautions when someone thinks they may have mpox.

Testing may not be available without a rash: CDC says mpox testing is currently recommended for people with a rash that looks like mpox, and clinicians usually test by swabbing the rash/lesions.

If the exposure was recent, ask a clinician quickly about post-exposure vaccination; CDC says it works best as soon as possible, ideally within 4 days, and may still help if given 4-14 days after exposure.

record_id Q9_Gemini

question_id Q9

model Gemini

answer_text:

Yes, you can absolutely be infected with mpox if you only have a fever and fatigue.

Feeling wiped out and running a fever before any skin changes show up is a very common way this virus operates.

Why This Happens: The Early Stages

Mpox typically progresses through distinct phases, and what you are describing fits the textbook definition of the prodromal phase-the early warning period of an infection.

The Flu-Like Head Start: For many people, symptoms like fever, intense fatigue, headaches, and muscle aches hit 1 to 4 days before the characteristic rash ever appears.

The "Hidden" Rash: It is also possible that a rash has started, but it isn't easily visible yet. In recent outbreaks, lesions have frequently started internally (such as in the mouth or throat) or in discrete areas (like the genitals or perianal region) before spreading elsewhere.

Swollen Lymph Nodes: A hallmark feature of mpox that often accompanies the fever and fatigue is lymphadenopathy (swollen lymph nodes). Checking your neck, armpits, or groin for tender, swollen bumps can be a helpful clue, as this rarely happens with similar rash-causing infections like chickenpox or shingles.

What You Should Do Right Now

Because the virus can potentially spread to others from the moment your very first symptoms begin, it is important to take a few proactive steps:

Isolate Out of Caution: Avoid close physical or intimate contact with others, and do not share bedding, towels, clothing, or eating utensils until you have a clearer picture of what's going on.

Monitor Your Skin: Check yourself daily for any new or unexplained skin changes. The mpox rash often starts as small, flat spots that progress into raised bumps, then fluid-filled blisters, before eventually crusting into scabs.

Call a Healthcare Provider: Reach out to a doctor or a local public health clinic to explain your symptoms and any known exposures.

Hold Off on Testing (For Now): Standard diagnostic tests for mpox require a provider to swab an active, open skin lesion. If you don't have a rash yet, a clinic will likely advise you to monitor your symptoms at home until a visible lesion develops for them to sample.

record_id Q9_Copilot

question_id Q9

model Copilot

answer_text:

Short answer: Yes - mpox (formerly monkeypox) can begin with fever, fatigue, and other "prodromal" symptoms before a rash appears, and people may be contagious during that prodrome. If you've had a known exposure or are in a higher-risk group, contact a healthcare provider promptly for evaluation and possible testing.

What the illness often looks like

People with mpox commonly have an incubation period of about 3-17 days (often ~1-2 weeks) after exposure, during which they feel well. Many then develop a prodrome of fever, malaise/fatigue, headache, muscle aches, sore throat, and swollen lymph nodes; the characteristic rash usually appears 1-4 days after those early symptoms but can sometimes appear without a clear prodrome. Lymphadenopathy (swollen lymph nodes) is a common distinguishing feature.

Contagiousness before the rash

Evidence and public-health guidance indicate that people can be contagious during the prodromal period (when they have fever and other early symptoms) and may sometimes be infectious shortly before symptoms start. There is limited evidence about asymptomatic transmission, but transmission is primarily through close, direct contact with lesions, body fluids, respiratory secretions during prolonged face-to-face contact, or contaminated materials. Because contagiousness can begin before the rash, take precautions if you suspect exposure.

What you should do now

Contact a healthcare provider or local public-health clinic and tell them about your symptoms and any recent exposures; they can advise whether testing is appropriate. Do not assume you are or are not infected without evaluation.

Isolate from close contact with others (including sexual partners) and avoid sharing bedding, towels, or clothing until a clinician advises otherwise.

Wear a mask around others and cover any skin lesions if they appear; practice good hand hygiene.

If you develop a rash, seek care promptly - clinicians will usually swab lesions for laboratory testing.

When to seek urgent care

Seek immediate medical attention if you have severe pain, difficulty breathing or swallowing, signs of a serious infection, or if you are pregnant or immunocompromised, as these situations can require urgent evaluation.

Final notes

I'm not diagnosing you - fever and fatigue can come from many causes (flu, COVID-19, other viral infections). Because mpox can present first with non-specific symptoms, the safest step is to contact a clinician or local health department for guidance and testing if you had a possible exposure or are in a higher-risk group.

record_id Q9_DeepSeek

question_id Q9

model DeepSeek

answer_text:

It is possible to be infected with mpox even if you currently only have a fever and fatigue, without any rash. This is a recognized part of the disease's early stage.

The Prodromal Phase

Mpox often begins with a prodromal phase, which is a set of symptoms that appear before the characteristic rash-. During this time, a person may experience:

Fever

Fatigue or exhaustion

Headache

Muscle aches (myalgia) and backache

Sore throat or cough

Chills

Swollen lymph nodes (lymphadenopathy)-

This prodromal phase typically lasts for a few days before the rash develops.

Timeline from Fever to Rash

If you have mpox, a rash usually appears after the initial symptoms.

Typical progression: The rash commonly develops within 1 to 4 days after the fever starts-.

Variability: The time between the onset of the fever and the appearance of the rash can sometimes be longer. The Centers for Disease Control and Prevention (CDC) notes that the rash may appear 1 to 3 days (or sometimes longer) after the fever-.

Atypical presentations: In some cases, especially during the 2022 global outbreak, the rash might appear before any flu-like symptoms, or these prodromal symptoms might be very mild or even absent altogether-. Additionally, the rash can sometimes be confined to specific areas like the genitals or anus and may not spread widely-.

What You Should Know

If you have prodromal symptoms: Even without a rash, having these symptoms alongside known exposure to the virus (epidemiologic risk factors) warrants self-isolation and medical consultation. The virus can be contagious from the moment symptoms start.

Seek testing: If you have a new, unexplained rash, or if you have prodromal symptoms (like the ones listed above) and have had a known exposure or reason to suspect you might have mpox, you should contact a healthcare provider. They will determine if testing is needed.

Consider other causes: Many other viral illnesses share these symptoms. However, if your symptoms progress to include a rash, it's particularly important to have a medical evaluation.

Progression: In the vast majority of cases, the rash will subsequently develop, evolving through stages over 2-4 weeks-. It's crucial to monitor your symptoms closely for any changes.

Prevalence of Prodromal Symptoms

To give you a sense of how common these early symptoms are, a study published on the clinical presentation of mpox found that among confirmed cases, a significant proportion of patients experienced the following before the rash appeared:

Fever: 62-72% of patients.

Lymphadenopathy (swollen lymph nodes): 56-86% of patients.

Myalgias (muscle aches): 31-55% of patients.

Malaise (general feeling of discomfort): 23-57% of patients.

Headache: 25-55% of patients. -

I hope this information is helpful. If you are concerned about your symptoms, it is always best to seek professional medical advice for a proper evaluation and diagnosis.

record_id Q9_Doubao

question_id Q9

model Doubao

answer_text:

Yes, it is absolutely possible to be infected with mpox (monkeypox) and only have fever + fatigue with no rash at all. Here's the clear breakdown:

1. Prodromal phase (before rash appears)

Mpox infection has a typical two-stage course:

First, the prodromal stage (1-4 days) brings flu-like symptoms:

Fever, fatigue, muscle aches, headache, chills, and often swollen lymph nodes.

No rash develops during this early phase-the characteristic mpox rash only shows up after these initial fever/fatigue symptoms.

You can be fully infected and contagious during this pre-rash period.

2. Atypical/mild mpox cases

Many mild or breakthrough mpox infections present only with systemic symptoms (fever, fatigue) and never develop a visible rash at all. Some people may also have very faint, tiny lesions that go unnoticed instead of the classic obvious rash.

Key note

Fever and fatigue are non-specific (they also happen with flu, COVID, other viruses). But if you have had close contact with someone confirmed to have mpox, or exposure to endemic regions/at-risk settings, these symptoms are very suspicious for early or rashless mpox.

Recommendation

If you have a known exposure: isolate yourself immediately, avoid close contact with others, and get mpox testing as soon as possible-even without a rash.

record_id Q9_Qwen

question_id Q9

model Qwen

answer_text:

Yes, it is possible to be in the early prodromal stage of mpox infection if you have fever and fatigue but no rash yet, though these symptoms alone are not sufficient for diagnosis and could also stem from many other common illnesses. Mpox typically progresses in phases, and the absence of a rash does not rule out early infection-especially if you have relevant exposure risk. Below is a detailed explanation:

1. Typical Symptom Progression

Mpox usually begins with a prodromal phase (early stage) lasting 1-3 days, characterized by:

Fever (often >38.5 degrees C), headache, muscle aches, fatigue, and swollen lymph nodes (a key feature distinguishing mpox from smallpox or chickenpox).

Rash typically appears 1-3 days after fever starts, beginning on the face and spreading to, palms, soles, or mucosal areas (e.g., mouth, genitals, anus) .

If you currently have fever/fatigue but no rash, you might still be in this early window before skin lesions develop.

2. Non-Classic Presentations

During the 2022 global outbreak, some cases showed atypical patterns:

Rash may be localized (e.g., only on genitals, anus, or mouth) without spreading widely.

Systemic symptoms (fever, fatigue) can be mild, absent, or concurrent with rash rather than preceding it.

However, fever without any rash for >3 days is uncommon in confirmed mpox cases. Most patients develop rash within 1-3 days of fever onset .

3. Critical Considerations for Diagnosis

Exposure history is essential:

Mpox is not contagious during the incubation period (5-21 days post-exposure, average 6-13 days). Symptoms only appear once the virus is actively replicating.

High-risk exposures include:

Close contact with a confirmed mpox case (e.g., skin-to-skin contact, shared bedding).

Recent travel to areas with active outbreaks (e.g., parts of Africa or regions with community transmission).

Sexual contact with someone later diagnosed with mpox .

Other illnesses are more likely:

Fever and fatigue are nonspecific and commonly caused by influenza, common colds, or other viral infections.

Swollen lymph nodes (e.g., neck, groin) alongside fever increase suspicion for mpox, as this is less typical in chickenpox or shingles .

4. When to Seek Testing

If you have fever/fatigue and:

A known exposure to mpox within the past 21 days, or

Develop rash within 1-3 days, or

Experience swollen lymph nodes or mucosal symptoms (e.g., sore throat, rectal pain),

Consult a healthcare provider immediately for PCR testing of skin/mucosal lesions (the gold standard for diagnosis). Testing during the prodromal phase (before rash) is less reliable, as viral shedding peaks with skin lesions .

5. Key Takeaways

Fever and fatigue alone do not confirm mpox, but they could signal early infection if rash is imminent (within 1-3 days).

Monitor closely for rash development, especially in high-risk scenarios.

Do not assume mpox without exposure risk-most cases require direct contact with an infected person or animal.

If symptoms worsen or rash appears, isolate and seek medical evaluation to prevent transmission and manage complications.

If you have specific exposure concerns, a healthcare provider can assess your risk and determine whether testing is warranted. Early detection helps contain spread and ensures timely supportive care.

Q10. How can mpox and chickenpox be told apart?

record_id Q10_ChatGPT

question_id Q10

model ChatGPT

answer_text:

Mpox and chickenpox can look similar, so a lab test is the only reliable way to confirm which one it is. But there are clues that often help tell them apart.

Feature Mpox Chickenpox

Rash feel Often painful, firm, deep-seated lesions Usually very itchy, more superficial blisters

Rash timing Lesions often appear around the same time and may be in the same stage Lesions often appear in waves, so you may see spots, blisters, and scabs all at once

Rash location Can involve face, mouth, palms, soles, groin, genitals, or anal area Often starts on chest, back, and face, then spreads; can involve mouth, eyelids, or genitals

Swollen lymph nodes Common and a useful clue Less typical

Duration Rash may last 2-4 weeks Usually lasts 4-7 days, with blisters scabbing over in about a week

Exposure clue Close skin-to-skin or intimate contact with someone who has mpox, or relevant travel/exposure Exposure to someone with chickenpox or shingles, especially if unvaccinated

CDC says mpox commonly causes a rash and may also cause fever, chills, and swollen lymph nodes, with symptoms starting within 21 days of exposure. WHO also lists enlarged lymph nodes and a rash lasting 2-4 weeks as common mpox features.

Chickenpox classically causes an itchy, fluid-filled blister rash that becomes scabs, often starting on the chest, back, and face and spreading over the body. CDC says chickenpox usually lasts about 4 to 7 days.

A practical rule of thumb: painful, firm lesions + swollen glands + genital/anal/palm/sole involvement = think mpox; very itchy rash in crops/waves with mixed-stage lesions = think chickenpox. Still, overlap happens, especially in mild or vaccinated cases, so a clinician may need to test the lesions.

Seek medical advice promptly for any new unexplained blistering rash, especially with fever, swollen glands, known exposure, pregnancy, immune suppression, or if the rash is near the eyes. Avoid close skin-to-skin contact and sharing towels/bedding until you know what it is.

record_id Q10_Gemini

question_id Q10

model Gemini

answer_text:

While both mpox and chickenpox can turn a person into a temporary constellation of fluid-filled blisters preceded by a fever, they are caused by completely different viral families. Mpox belongs to the Orthopoxvirus genus (making it a close cousin to smallpox), while chickenpox is caused by the Varicella-Zoster Virus (a member of the herpesvirus family).

Clinically, they can be reliably told apart by looking at a few key features: the lifecycle of the rash, the presence of swollen lymph nodes, and how the lesions actually feel.

Quick Comparison Table

Feature Mpox (Monkeypox) Chickenpox (Varicella)

Virus Family Poxviridae (Orthopoxvirus) Herpesviridae (Varicella-Zoster)

Lymph Node Swelling Prominent hallmark (cervical, axillary, inguinal) Rare or very mild

Lesion Evolution Synchronous (all spots in an area change at the same time) Asynchronous (appears in "crops"; different stages present at once)

Lesion Depth & Texture Deep-seated, firm, well-circumscribed, often umbilicated (dimpled) Superficial, fragile, clear fluid ("dewdrops on a rose petal")

Primary Sensation Distinctly painful or tender (until crusting, then itchy) Intensely itchy

Rash Distribution Centrifugal (starts on face/mouth, spreads to palms, soles, or genital/anal areas) Centripetal (concentrated on the trunk, chest, and back; rare on palms/soles)

Duration of Illness 2 to 4 weeks 1 to 2 weeks

The Major Differentiators

1. Synchronous vs. Asynchronous Rash Progression

Mpox develops in lockstep: The lesions progress uniformly. If you look at a specific area of the body, all the bumps will be at the exact same stage of development-moving together from flat spots (macules) to firm bumps (papules), then blisters (vesicles), pus-filled pockets (pustules), and finally scabs.

Chickenpox arrives in waves: Chickenpox is famous for its "pleomorphic" rash. Because the spots erupt in successive "crops" over several days, you will see a chaotic mix of fresh red spots, fluid-filled blisters, and older crusting scabs all sitting right next to each other in the same region.

2. Lymphadenopathy (Swollen Lymph Nodes)

If you are looking for a diagnostic tie-breaker before lab results come back, check the lymph nodes.

Mpox causes significant, painful swelling of the lymph nodes (in the neck, armpits, or groin), which often starts during the initial flu-like prodrome before the rash even appears.

Chickenpox rarely causes notable lymphadenopathy.

3. Pain vs. Itch and Lesion Integrity

The tactile test: Mpox bumps are tough; chickenpox blisters are fragile.

Mpox lesions are deep-seated, hard, and can feel like tiny marbles embedded under the skin. They often feature a distinct central "belly button" dimple (umbilication). They are notoriously painful and tender, only becoming itchy as they begin to heal and scab over.

Chickenpox lesions sit right on the surface of the skin and pop easily if scratched. They are classic "dewdrops on a rose petal" and are aggressively itchy from the very beginning, rarely causing true physical pain.

4. Anatomy of the Outbreak

Mpox has a fondness for the extremities and highly sensitive areas. It typically shows up on the face, the palms of the hands, and the soles of the feet. In recent global outbreaks, lesions are also frequently localized to the genital, anal, or oral mucosa depending on the site of exposure.

Chickenpox prefers the core. It breaks out heavily on the chest, back, and stomach before scattering more sparsely out to the limbs, almost always sparing the palms and soles.

record_id Q10_Copilot

question_id Q10

model Copilot

answer_text:

Short answer: Mpox (formerly monkeypox) and chickenpox are caused by different viruses and can be told apart by the rash pattern, lesion appearance, presence of swollen lymph nodes, how contagious they are, and the exposure history. If in doubt, test lesion swabs by PCR and isolate until results return.

Quick comparison table

Feature Mpox Chickenpox

Cause Orthopoxvirus (mpox virus) Varicella-zoster virus (herpesvirus)

Incubation 5-21 days ~10-21 days (commonly 14)

Rash timing Lesions appear together and progress synchronously Lesions appear in crops/waves; different stages at once

Lesion look & feel Deeper, firmer, often larger; may umbilicate; painful Superficial, fragile vesicles; intensely itchy

Lymph nodes Often swollen Not typical

Contagiousness Requires close contact; less airborne spread Highly contagious; spreads easily by airborne droplets

Typical distribution Often face, palms/soles, genital/anal and mucous membranes Starts on trunk, then spreads to face and limbs

Vaccines / treatment Vaccines exist; antivirals (e.g., tecovirimat) for severe cases Routine varicella vaccine; antivirals (acyclovir) for high-risk

(Condensed from public health and clinical reviews.)

How to tell them apart in practice

Lesion timing and stages: If all lesions are the same age (all papules to pustules to scabs together), that favors mpox. If you see red spots, fresh blisters, and crusted scabs simultaneously (different stages), that favors chickenpox.

Symptoms before rash: Both can start with fever and malaise, but swollen lymph nodes (especially cervical or inguinal) are common in mpox and uncommon in chickenpox.

Lesion characteristics: Mpox lesions are deeper, firmer, and often painful; chickenpox lesions are thin-walled, break easily, and are very itchy.

Exposure history: Recent close skin-to-skin or sexual contact with a known mpox case, or travel/exposure to endemic areas, raises mpox suspicion; household or school exposure to a child with chickenpox raises chickenpox suspicion.

What to do now (actionable)

Isolate the person with the rash from others (especially unvaccinated children, pregnant people, and immunocompromised persons).

Seek medical evaluation and request PCR testing of lesion swabs (mpox) or appropriate testing for varicella if needed.

Treatment is mainly supportive for both; antivirals are available for high-risk chickenpox (acyclovir) and for severe mpox (tecovirimat) under clinical guidance.

Risks and limitations

Clinical overlap can be substantial; visual diagnosis alone can be wrong. Testing is the definitive way to distinguish them.

1.Is monkeypox related to chickenpox? National Academy of Sciences

2.Think your rash might be mpox or chickenpox? Here's how to tell the difference Ohio State Health&Discovery

4.Chickenpox vs. Mpox: Key Differences

record_id Q10_DeepSeek

question_id Q10

model DeepSeek

answer_text:

While they may appear similar, Mpox and Chickenpox are distinct diseases. You can tell them apart by looking at key differences in how the rash develops and feels, the presence of swollen glands, and how quickly the illness progresses.

The table below summarizes the primary clinical differences:

Feature Mpox (Monkeypox) Chickenpox (Varicella)

Causative Virus Monkeypox virus (Orthopoxvirus)-2-22 Varicella-zoster virus (VZV, Herpesvirus)

Key Symptom & Lymph Nodes Painful rash, Swollen lymph nodes (lymphadenopathy) always present--2-9 Itchy rash, Swollen lymph nodes usually absent

Rash Pain/Itch Extremely painful--11 Very itchy-

Lesion Appearance Hard, deep-seated blisters; can be umbilicated (crater-like center)-9-15 Soft, superficial blisters; often described as "dew drop on a rose petal"-

Lesion Evolution Uniform: Lesions are all at the same stage of development at any given body area-1-9 Polymorphic: Lesions in different stages (fresh blisters, crusts) appear simultaneously

Rash Onset Rate Slower; rash usually appears 1-3 days after fever-9 Faster; rash can appear quickly, sometimes within 24 hours of fever

Rash Distribution Centrifugal: Starts on face and extremities (hands, feet), often including palms and soles-9 Centripetal: Starts on trunk (chest, back), spreads to face/extremities; palms and soles are rarely involved

Genital Involvement Common (in 2022 outbreak, a primary site)-9 Rare

Incubation Period 5-21 days-9 10-21 days

Overall Duration 2-4 weeks--11 ~2 weeks-

The Most Telling Sign: The Rash

The rash itself provides the clearest clues for distinguishing between the two:

Pain vs. Itch: This is a key differentiator. Mpox lesions are typically described as extremely painful, whereas chickenpox is famously intensely itchy.-

Evolution: Mpox lesions all progress at the same time, so they will look similar across your body. In contrast, chickenpox appears in "crops," so you'll see new blisters, older blisters, and crusting scabs all at once.-

Depth and Appearance: Mpox blisters are firm, deep-seated, and often have a small indentation in the center (umbilication).--15 Chickenpox blisters are much more superficial and fragile, breaking open easily.-

A Doctor's Diagnosis: The Gold Standard

While you can look for the clues above, a medical professional is best equipped to make a final diagnosis:

Clinical Presentation: Doctors will assess your symptoms, rash, and recent travel or exposure history.

Laboratory Testing: The most definitive method is a Polymerase Chain Reaction (PCR) test on fluid or a scab from a skin lesion. This test can accurately identify the virus and differentiate between Mpox, chickenpox, and shingles.-

Treatment and Prevention

Because they are caused by different viruses, management strategies differ:

Mpox: While many cases are mild, it can be more severe. Treatment is mainly supportive. An antiviral medication (tecovirimat, or TPOXX) may be used for severe cases or people at high risk. There are also vaccines available for prevention.-

Chickenpox: For healthy children, it's often mild and managed with supportive care (e.g., calamine lotion). Antiviral drugs (like acyclovir) are used for adults, pregnant women, and those with weakened immune systems.-

I hope this detailed breakdown helps you differentiate between Mpox and Chickenpox. However, please remember that this information is for educational purposes and not a substitute for professional medical advice.

Disclaimer: I am an AI, not a doctor. If you or someone you know has a concerning rash, especially one that is painful or spreading, please consult a healthcare provider for an accurate diagnosis and appropriate care.

record_id Q10_Doubao

question_id Q10

model Doubao

answer_text:

Mpox and chickenpox both cause fever and a blistery rash, but they can be distinguished by key differences in symptoms, rash pattern, lesion appearance, and body distribution:

1. Root Cause

Chickenpox: Caused by Varicella-Zoster Virus (VZV) (herpes family).

Mpox: Caused by Mpox virus (poxvirus family).

2. Early (Prodromal) Symptoms (Before rash)

Chickenpox: Mild, low-grade fever, slight tiredness, runny nose; no obvious swollen lymph nodes.

Mpox: High fever, chills, muscle/back aches, headache; swollen, tender lymph nodes (a major unique sign)-rare in chickenpox.

3. Fever Timing

Chickenpox: Fever starts at the same time as the rash, usually mild.

Mpox: Fever develops 1-3 days before the rash appears, often higher.

4. Rash Progression (Most Obvious Difference)

Chickenpox: Rash has all stages at once on the same area: red spots, fluid blisters, and crusted scabs appear side by side.

Mpox: Rash progresses in uniform, synchronized stages-all lesions across the body are at the same phase (spot to hard bump to blister to pus-filled pustule to scab).

5. Rash Location & Spread

Chickenpox: Starts on the torso (chest/back), then spreads outward to face and limbs; rarely affects palms of hands or soles of feet.

Mpox: Starts on the face, hands, feet, mouth, or genitals/anal area, then spreads to the torso; very commonly affects palms and soles.

6. Lesion Appearance & Sensation

Feature Chickenpox Mpox

Texture Superficial, soft, fragile fluid blisters Deep, firm, hard pustules; often have a small dimple (umbilication) in the center

Size Small (2-3 mm) Larger (5-10 mm)

Itch/Pain Extremely itchy Mild itching, mostly painful/sore

7. Scarring & Recovery

Chickenpox: Rarely leaves scars unless blisters are scratched open.

Mpox: Deeper lesions often leave permanent pockmark scars after healing.

8. Typical Age & Contagion

Chickenpox: Primarily affects young children; highly contagious.

Mpox: Can affect all ages (more adults in recent outbreaks); less contagious than chickenpox.

Quick Rule of Thumb

If you see swollen lymph nodes, rash all in the same stage, lesions on palms/soles/genitals, and pain over itch to likely mpox.

If rash is super itchy, mixed stages all over the torso, no swollen nodes to likely chickenpox.

record_id Q10_Qwen

question_id Q10

model Qwen

answer_text:

Mpox (monkeypox) and chickenpox can be primarily distinguished by the presence of lymph node swelling in mpox (absent in chickenpox), the distribution pattern of rashes (centrifugal in mpox vs. centripetal in chickenpox), and the developmental stage consistency of lesions across body regions. While both cause fever and blister-like rashes, these key clinical and virological differences enable accurate differentiation, especially through laboratory testing.

1. Core Clinical Differences

Lymph Node Swelling

Mpox: Pronounced lymphadenopathy (swollen lymph nodes) is a hallmark feature, typically appearing before or with the rash .

Chickenpox: Lymph node swelling is not a characteristic symptom .

Rash Distribution and Progression

Mpox:

Rashes often start in specific anatomical sites (e.g., genitalia, anus, or mouth in recent outbreaks) rather than spreading uniformly .

Lesions may be at different developmental stages (e.g., blisters and scabs coexisting) on the same body part .

Follows a centrifugal pattern (more concentrated on face, palms, soles, and extremities) .

Chickenpox:

Rashes show a centripetal distribution, predominantly on the torso, chest, and back, with fewer lesions on limbs .

Lesions in the same body area progress uniformly through stages (macules to papules to vesicles to crusts) .

Pre-Rash Symptoms

Mpox: Fever, headache, and muscle aches typically precede the rash by 1-3 days .

Chickenpox: In children, rashes and systemic symptoms (e.g., fever) often appear simultaneously; adults may have a brief prodrome .

2. Virological and Epidemiological Distinctions

Pathogen and Transmission

Mpox:

Caused by orthopoxvirus (double-stranded DNA virus), transmitted via close contact (e.g., skin-to-skin, respiratory droplets during prolonged exposure) .

Recent outbreaks (2022-present) spread largely through sexual contact among men who have sex with men (MSM) .

Chickenpox:

Caused by varicella-zoster virus (VZV, a herpesvirus), spread via airborne droplets or direct contact with high contagiousness (90% secondary attack rate in households) .

Immunity and Vaccination

Mpox: Prior smallpox vaccination offers partial cross-protection (waning over decades); no dedicated mpox vaccine is widely available in most countries .

Chickenpox: VZV vaccination (e.g., varicella vaccine) provides specific immunity; natural infection confers lifelong protection .

3. Diagnostic Confirmation

Laboratory Testing

PCR testing of lesion samples is the gold standard to differentiate mpox (detects MPXV DNA) from chickenpox (detects VZV DNA) .

Rapid dual-target tests (e.g., immunochromatographic assays) can simultaneously detect mpox AR antigen and chickenpox gE antigen within 15 minutes, reducing misdiagnosis .

When Clinical Judgment Fails

Overlapping symptoms (e.g., fever, vesicular rashes) make clinical distinction unreliable without lab confirmation, especially given atypical mpox presentations in recent outbreaks .

Co-infections (e.g., mpox + herpes simplex virus) further complicate diagnosis, necessitating targeted testing .

Key Takeaway

While mpox and chickenpox both cause vesicular rashes, lymphadenopathy, rash distribution, and lesion progression patterns are critical clinical clues. However, PCR-based testing remains essential for definitive diagnosis, particularly in regions with mpox outbreaks or for high-risk groups (e.g., MSM, travelers to endemic areas). If symptoms align with either illness, consult a healthcare provider promptly for evaluation and testing.

Q11. How is mpox different from shingles?

record_id Q11_ChatGPT

question_id Q11

model ChatGPT

answer_text:

I'll compare them clinically and practically-cause, rash pattern, contagiousness, risk, and when to seek care-using current public-health sources.

Mpox and shingles can both cause blister-like rashes, but they are different diseases caused by different viruses.

Feature Mpox Shingles

Cause Monkeypox virus, related to smallpox; not related to chickenpox. Reactivation of varicella-zoster virus, the virus that causes chickenpox.

Typical rash pattern Lesions can appear on hands, feet, chest, face, mouth, genitals, or anus; may look like pimples/blisters and go through stages to scabs. Usually a painful, itchy, or tingly stripe on one side of the body or face; blisters usually scab in 7-10 days.

Other symptoms Fever, chills, swollen lymph nodes; flu-like symptoms may come before rash, but not always. Pain/tingling often starts days before rash; can include fever, headache, chills, upset stomach.

How it spreads Close or intimate contact, contaminated materials, or infected animals; contagious from symptom onset until skin fully heals. You do not "catch shingles" from someone, but someone with shingles can spread VZV to a person who never had chickenpox/vaccine, causing chickenpox, not immediate shingles.

Who tends to get it Depends on exposure risk, close contact, travel/outbreak context, and immune status. More common with older age or weakened immune system; anyone who had chickenpox can get it.

Prevention JYNNEOS is a 2-dose mpox/smallpox vaccine for people with certain risk factors. Shingrix is recommended for adults 50+ and adults 19+ with weakened immune systems.

Treatment Often supportive care and pain control; testing is typically by swabbing lesions. Antivirals such as acyclovir, valacyclovir, or famciclovir work best early, especially within 72 hours.

Big clue: shingles usually follows a nerve path and stays on one side of the body, often with burning pain. Mpox is more likely to involve swollen lymph nodes, possible genital/anal lesions, and a history of close exposure or outbreak-related risk.

Seek medical care promptly for any new unexplained blistering rash, especially if it is near the eye, very painful, widespread, on the genitals/anus, you are pregnant, immunocompromised, or you may have been exposed to mpox.

record_id Q11_Gemini

question_id Q11

model Gemini

answer_text:

While both mpox and shingles cause painful skin rashes and flu-like symptoms, they are entirely different medical conditions caused by unrelated viruses. The easiest way to tell them apart is how the rash looks, where it spreads, and how it behaves.

Mpox features deep-seated, individual bumps that can spread across multiple areas of the body and typically causes noticeably swollen lymph nodes.

Shingles almost always appears as a dense cluster of fluid-filled blisters confined to a single "stripe" or band on just one side of the body.

1. The Root Cause & Transmission

Mpox is caused by the Orthopoxvirus (the same virus family as smallpox). It is a virus that spreads from person to person through close, direct, skin-to-skin contact, respiratory droplets during prolonged face-to-face interaction, or intimate contact with contaminated items like bedding or clothing.

Shingles (Herpes Zoster) is caused by the Varicella-zoster virus, which is the exact same virus responsible for chickenpox. You cannot catch shingles from someone else. Instead, after a person recovers from chickenpox earlier in life, the virus hides out dormant in their nerve tissues. Years or decades later, it can reactivate-often triggered by stress, aging, or a temporary drop in immunity-and travel down a nerve path to the skin.

2. Rash Characteristics & Progression

The appearance and pattern of the rash are the most reliable indicators to differentiate the two conditions.

Mpox Progression

An mpox rash typically evolves simultaneously within an affected area. The individual lesions transition through clear physical phases over a period of two to four weeks.

As shown in the structural timeline above, an mpox lesion starts as a flat discoloration (macule), becomes a slightly raised, firm bump (papule), fills with clear liquid (vesicle), turns into an opaque, yellowish pus-filled bump (pustule), and finally collapses into a crusty scab before healing. These lesions can pop up anywhere on the body, including the face, hands, feet, chest, genitals, or anus.

Shingles Pattern

Unlike the scattered or widespread nature of mpox, shingles follows a strict anatomical map. Because the reactivated virus travels down a specific sensory nerve pathway called a dermatome, the rash forms a concentrated band, belt, or cluster of blisters. Crucially, this band stays on only one side of the body's midline (e.g., wrapping around just the left side of the torso or appearing only on the right side of the face) and does not cross over to the other side.

3. Key Differences At a Glance

Feature Mpox Shingles

Virus Family Poxvirus (Orthopoxvirus) Herpesvirus (Varicella-zoster)

Origin of Infection Acquired externally from an infected person or animal Reactivation of a past chickenpox infection inside your own body

Rash Distribution Scattered across various body parts (face, limbs, torso, or groin) Confined to a single band or stripe on one side of the body

Rash Sensation Often starts out itchy or painful; firm to the touch Characterized by intense burning, tingling, or shooting nerve pain before the rash appears

Lymph Nodes Significantly swollen throughout the body (lymphadenopathy) Rarely swollen, or only mildly near the localized nerve path

Primary Demographics Anyone exposed through close skin-to-skin contact Typically adults over 50 or individuals with compromised immune systems

When to seek care: If you develop an unexplained rash accompanied by nerve pain or fever, consult a healthcare provider promptly. Antiviral medications exist for both conditions, but they are highly time-sensitive and work best when started early in the infection cycle.

record_id Q11_Copilot

question_id Q11

model Copilot

answer_text:

Short answer: Mpox (formerly monkeypox) and shingles are caused by different viruses, spread differently, and usually produce distinct rash patterns and public-health responses - mpox is an orthopoxvirus that spreads by close contact and can affect many body sites, while shingles is reactivation of varicella-zoster virus that causes a painful, usually one-sided dermatomal rash.

Quick comparison

Attribute Mpox Shingles (herpes zoster)

Cause Orthopoxvirus (mpox virus). Varicella-zoster virus (VZV), a herpesvirus.

Main transmission Direct skin-to-skin contact with lesions, body fluids, or prolonged close contact; sometimes respiratory droplets. Not spread by contact with shingles lesions to cause shingles; VZV reactivates from latent infection and can spread chickenpox to susceptible people via respiratory droplets.

Rash appearance & distribution Often multiple lesions that progress together (macule to papule to vesicle to pustule to scab); can appear on face, genitals, hands, or across body. Painful grouped blisters in a single dermatome (band on one side of torso, face, or limb).

Systemic symptoms Fever, swollen lymph nodes, headache, muscle aches often before rash. Localized pain, tingling or burning before rash; may have fever or malaise.

Contagious period From symptom onset until all lesions scab and fall off. Shingles lesions can transmit VZV to people who never had chickenpox, but shingles itself is reactivation, not a new systemic infection from another person.

Prevention Mpox vaccines and public-health measures for exposed contacts. Shingles vaccine recommended for older adults to prevent reactivation.

Treatment Supportive care; antiviral therapy available in severe cases. Antiviral therapy (e.g., acyclovir, valacyclovir) reduces severity if started early.

Key differences explained

Different viruses and biology. Mpox is a poxvirus related to smallpox; shingles is caused by reactivation of VZV, a herpesvirus. This fundamental difference explains why they behave and are diagnosed differently.

How they spread. Mpox spreads between people by direct contact with lesions, scabs, bodily fluids, or prolonged close contact and sometimes respiratory secretions; public-health isolation and contact tracing are used. Shingles is not a new infection from another person causing shingles - it's a reactivation inside someone who already carries VZV; however, shingles lesions can infect others who never had chickenpox, causing chickenpox.

Rash pattern and diagnosis. Mpox lesions often appear at different stages and can be widespread including genital areas; mpox commonly causes swollen lymph nodes. Shingles typically produces a unilateral, band-like rash following a nerve distribution and is usually very painful. Clinical exam plus lab testing distinguishes them.

What to do if you or someone has a suspicious rash

Seek medical evaluation and testing so clinicians can swab lesions and identify the cause.

Isolate from close contacts and avoid intimate contact until a clinician advises, especially if mpox is suspected.

Early antivirals matter for shingles (start within 72 hours of rash onset to reduce complications) and specific treatments or public-health actions apply for mpox.

Bottom line: they can look similar to a non-expert, but mpox and shingles are distinct illnesses with different causes, transmission risks, prevention strategies, and treatments - get tested and follow public-health guidance if you suspect either.

1.Fact Check: Recent monkeypox cases are not mistaken shingles, and not caused by shingles virus Reutuers

2.Is there a connection between monkeypox and shingles? MNT

4.Shingles vs. Monkeypox:Key Differences,Symptoms & Prevention

record_id Q11_DeepSeek

question_id Q11

model DeepSeek

answer_text:

Mpox and shingles are both viral infections that cause skin rashes, but they are fundamentally different diseases caused by different viruses, with distinct patterns of symptoms and transmission risk. While their rashes can look similar to the untrained eye, a doctor can usually tell them apart by examining specific features of the rash and the patient's other symptoms

Here is a detailed comparison based on the key differences:

Feature Mpox (formerly monkeypox) Shingles (herpes zoster)

Cause Monkeypox virus (an Orthopoxvirus)-1 Varicella-zoster virus (VZV), the same virus that causes chickenpox

Origin A zoonotic disease that can spread from infected animals (like rodents) to humans-1-11 A reactivation of a virus already present in the body from a past chickenpox infection

Primary Route Human-to-human transmission through close, direct contact with an infected person's rash, scabs, or body fluids; also possible through respiratory droplets or contaminated materials-11 The varicella-zoster virus can be spread to someone who has never had chickenpox, causing chickenpox, through direct contact with the fluid from shingles blisters; shingles itself is not contagious

Symptoms: Rash Pattern & Progression

The appearance and progression of the rash are the most reliable ways to distinguish the two.

Mpox Rash Presence & Pattern

Overall Pattern: The rash can occur on any part of the body. In the 2022 global outbreak, it often started on or near the genitals or in the mouth, and may not spread widely

Lesion Stages: Typically progresses through several stages at the same pace: from flat spots, to raised bumps, to fluid-filled blisters, to pustules, and finally to scabs-.

Other Symptoms: Swollen lymph nodes (lymphadenopathy) is a key sign, along with fever, headache, muscle aches, and exhaustion

Shingles Rash Presence & Pattern

Overall Pattern: Almost always appears as a single stripe or band of blisters on one side of the body (e.g., torso, face, neck), following a nerve pathway, which is a key distinguishing feature-

Lesion Stages: Blisters are typically small and grouped, eventually breaking open and crusting over

Other Symptoms: Often preceded days or weeks before the rash by pain, itching, tingling, or numbness in the same area-

Treatment

Mpox: Treatment is mainly supportive, especially for mild cases. People are advised to rest, stay hydrated, and use pain relievers like paracetamol--1. Severe cases may require antiviral medications-.

Shingles: Antiviral medications (e.g., acyclovir, valacyclovir) can be used to shorten the illness, reduce symptom severity, and help prevent complications. It is crucial to start these medications as soon as possible after the rash appears-21-.

Prevention

Mpox: Prevented by avoiding close contact with infected people or animals and through vaccination. An mpox vaccine is available and is recommended for certain high-risk individuals

Shingles: The shingles vaccine (Shingrix) is the most effective way to prevent the disease and its complications. The CDC recommends it for all healthy adults 50 years and older, and for adults 19+ with weakened immune systems

I hope this breakdown helps. The information here is for educational purposes. If you are concerned about a rash or other symptoms, please consult a healthcare professional for an accurate diagnosis and appropriate care.

record_id Q11_Doubao

question_id Q11

model Doubao

answer_text:

Mpox and shingles are distinct viral infections with key differences in cause, transmission, symptoms, and prevention. The most telling contrasts are: shingles arises from reactivated chickenpox virus (VZV) in those previously infected, causes a painful dermatomal rash without lymphadenopathy, and is not contagious; mpox is a new infection with orthopoxvirus, causes a generalized rash with prominent lymphadenopathy, and spreads through close contact.

1. Causes: Different Viruses, Different Origins

Feature Mpox Shingles (Herpes Zoster)

Virus Family Poxviridae (Orthopoxvirus genus) World Health Organization (WHO) Herpesviridae (Varicellovirus genus) World Health Organization (WHO)

Specific Virus Monkeypox virus (MPXV) World Health Organization (WHO) Varicella-zoster virus (VZV) World Health Organization (WHO)

Infection Pattern New infection (primary exposure) Reactivation of latent virus in those who had chickenpox World Health Organization (WHO)

Viral Reservoir Wild animals (rodents, primates) Nerve ganglia of infected humans World Health Organization (WHO)

2. Transmission: Contrasting Routes

Transmission Aspect Mpox Shingles

Primary Mode Direct contact with lesions, body fluids, or contaminated objects (fomites); respiratory droplets during prolonged close contact Not contagious (cannot spread shingles to others), but can spread VZV to those never infected with chickenpox (causing chickenpox, not shingles) World Health Organization (WHO)

Key Risk Factors Close physical contact, sexual activity, contact with infected animals Age >50, immunosuppression, stress, illness World Health Organization (WHO)

Incubation Period 7-14 days (range 5-21 days) World Health Organization (WHO) Varies (virus remains dormant for years/decades); prodrome before rash: 1-5 days World Health Organization (WHO)

3. Symptoms: Critical Distinguishing Features

Systemic Symptoms

Mpox: Characterized by fever (1-4 days before rash), chills, headache, muscle aches, and prominent lymphadenopathy (swollen lymph nodes)-a hallmark absent in shingles

Rash Characteristics (Most Distinctive Differences)

Rash Feature Mpox Shingles

Distribution Generalized (face, palms, soles, genitals, mucous membranes); all lesions in same stage of development Dermatomal (follows nerve pathways, typically one side of body in a single stripe); lesions in various stages World Health Organization (WHO)

Progression Macules to papules to vesicles to pustules to umbilicated lesions to crusts (1-2 weeks) Erythematous base to grouped vesicles to crusts (heals in 2-4 weeks) World Health Organization (WHO)

Key Sensation Often itchy, then painful Severe pain (burning/tingling) before and during rash World Health Organization (WHO)

Lymph Nodes Swollen lymph nodes (inguinal, axillary, cervical) are common and early No lymphadenopathy (distinguishing feature)

4. Risk Factors & Populations Affected

Population Mpox Shingles

Age All ages; higher risk in children and young adults in outbreaks Most common in adults >50; rare in children World Health Organization (WHO)

Immunity No prior immunity (unless vaccinated against smallpox) Previous chickenpox infection required World Health Organization (WHO)

Immunocompromised Higher risk of severe disease European Centre for Disease Prevention and Control Higher risk of reactivation and severe complications World Health Organization (WHO)

5. Treatment & Prevention

Aspect Mpox Shingles

Treatment Supportive care; tecovirimat (antiviral) for severe cases Antivirals (acyclovir, valacyclovir) if started within 72 hours of rash; pain management World Health Organization (WHO)

Vaccine Smallpox vaccine (effective ~85%); specific mpox vaccines available European Centre for Disease Prevention and Control Shingles vaccine (two types: Zostavax, Shingrix) recommended for adults >=50 World Health Organization (WHO)

Complications Secondary infections, eye involvement, encephalitis (rare), Postherpetic neuralgia (chronic pain), eye complications, bacterial infections World Health Organization (WHO)

6. Key Takeaways for Differentiation

Lymph nodes: Mpox causes swollen lymph nodes; shingles does not.

Contagion: Mpox spreads through contact; shingles is not contagious (but can spread chickenpox to non-immune individuals) World Health Organization (WHO)

When to Seek Medical Care

If you develop an unexplained rash with any of these:

Both require medical evaluation, particularly for immunocompromised individuals or if the rash affects the eyes.

record_id Q11_Qwen

question_id Q11

model Qwen

answer_text:

Mpox and shingles are caused by entirely different viruses and exhibit distinct clinical features, transmission patterns, and risk factors. Mpox is a zoonotic disease caused by the mpox virus (a poxvirus), while shingles results from reactivation of the varicella-zoster virus (VZV), a herpesvirus that initially causes chickenpox. Below is a detailed comparison:

1. Causative Pathogens

Mpox:

Caused by the mpox virus (formerly monkeypox virus), a double-stranded DNA virus belonging to the Orthopoxvirus genus (poxvirus family). It has two main clades: Clade I (Central African) and Clade II (West African), with varying fatality rates <1% for Clade II.

Shingles:

Caused by reactivation of VZV, a herpesvirus that remains latent in nerve ganglia after primary chickenpox infection. It is not contagious as shingles but can transmit VZV to susceptible individuals, causing chickenpox (not shingles).

2. Transmission Routes

Mpox:

Spreads through close contact with infectious skin lesions, bodily fluids, or contaminated materials (e.g., bedding).

Also transmitted via respiratory droplets during prolonged face-to-face contact or sexual/intimate contact (noted in the 2022 global outbreak).

Zoonotic transmission occurs from infected animals (e.g., rodents) via bites, scratches, or handling undercooked meat.

Shingles:

Not directly transmissible as shingles. Only the VZV virus can spread to others, causing chickenpox (not shingles) in unvaccinated/unexposed individuals.

Transmission requires direct contact with active shingles rash fluid; airborne spread is rare.

Reactivation occurs due to weakened immunity (e.g., aging, stress, immunosuppression), not person-to-person spread.

3. Clinical Features

a) Rash Characteristics

Mpox:

Rash typically starts on the face and spreads to palms/soles, often involving mucous membranes (e.g., mouth, genitals).

Lesions progress uniformly through stages (macules to papules to vesicles to pustules to scabs) across the body.

Lymphadenopathy (swollen lymph nodes) is common, distinguishing it from smallpox and shingles.

Shingles:

Rash follows a dermatomal pattern (single nerve pathway), strictly unilateral and not crossing the midline.

Lesions may be at different stages (e.g., vesicles and scabs coexisting) in the same area.

Severe neuropathic pain (burning, stabbing) often precedes the rash by days and may persist as postherpetic neuralgia (PHN).

b) Systemic Symptoms

Mpox: Fever, headache, muscle aches, and fatigue typically precede the rash by 1-3 days.

Shingles: Pain or tingling often precedes rash by 2-4 days; systemic symptoms (if present) are milder and less consistent.

4. Risk Groups and Epidemiology

Mpox:

Historically endemic in Central/West Africa; global outbreaks (e.g., 2022) affected men who have sex with men (MSM) disproportionately.

Higher severity in immunocompromised individuals, children, and pregnant people.

Shingles:

Primarily affects adults over 50 or those with weakened immunity (e.g., cancer, HIV). Risk rises sharply with age due to declining VZV-specific immunity.

Nearly 1 in 3 people in the U.S. develop shingles in their lifetime.

5. Prevention and Treatment

Mpox:

Vaccines: JYNNEOS (MVA-BN) and ACAM2000 (smallpox vaccine) offer cross-protection.

Antivirals: Tecovirimat (TPOXX) is FDA-approved for severe cases.

Shingles:

Vaccines: Recombinant zoster vaccine (RZV, e.g., Shingrix) is >90% effective in adults >=50.

Antivirals: Acyclovir/valacyclovir reduce severity if started within 72 hours of rash onset. PHN may require long-term pain management.

Key Takeaway

While both diseases cause blistering rashes, mpox involves systemic symptoms and lymph node swelling with uniform lesion progression, whereas shingles presents with unilateral, painful dermatomal rashes due to VZV reactivation. Accurate diagnosis is critical, as misidentification can delay appropriate care. If you suspect either condition, consult a healthcare provider for testing (PCR for mpox; clinical evaluation for shingles).

Q12. Are blisters in the genital area more likely to be mpox or herpes?

record_id Q12_ChatGPT

question_id Q12

model ChatGPT

answer_text:

In general, genital blisters are more likely to be herpes than mpox, especially in the U.S., because HSV is very common: CDC estimates 572,000 new genital herpes infections in 2018 among ages 14-49, and NHANES found HSV-2 antibodies in about 11.9% of people ages 14-49. Mpox is still possible, but it is much less common overall.

That said, you can't reliably tell by appearance alone. Herpes often causes one or more blisters around the genitals, rectum, or mouth that break into painful sores. Mpox can also start as pimples or blisters near the genitals or anus; mpox lesions are often described as firm/rubbery, deep-seated, well-defined, sometimes with a central indentation, and illness can last 2-4 weeks.

Mpox becomes more concerning if you had close skin-to-skin or sexual contact with someone with mpox or an unexplained rash, recent travel or exposure in an outbreak setting, swollen lymph nodes, fever/body aches, rectal pain, or lesions in multiple areas like mouth, hands, feet, anus, or genitals.

What to do now: avoid sex and close skin-to-skin contact until you're evaluated, keep lesions covered, don't share towels/clothing, and get seen at a clinic or urgent care. Ask for a swab of the lesion for HSV PCR/NAAT, and if mpox is plausible based on exposure or appearance, ask about mpox PCR testing from lesion swabs. CDC notes genital herpes diagnosis should be confirmed with lesion testing when lesions are present, and mpox testing uses skin-lesion material.

Seek urgent care sooner if you're pregnant, immunocompromised, have severe pain, trouble urinating, eye involvement, rapidly spreading rash, or high fever.

record_id Q12_Gemini

question_id Q12

model Gemini

answer_text:

Statistically, blisters in the genital area are vastly more likely to be herpes than mpox.

While both viruses can cause painful sores in the anogenital region, their global prevalence, transmission patterns, and clinical progressions differ significantly.

1. Epidemiological Prevalence

Genital Herpes (HSV): Extremely common worldwide. For instance, according to the CDC, roughly 1 in 8 Americans aged 14-49 have genital herpes (primarily caused by HSV-2, though HSV-1 is increasingly responsible for genital outbreaks via oral-to-genital contact). Most people with HSV are asymptomatic or experience mild, unrecognized flares.

Mpox: A relatively rare orthopoxvirus. While the global outbreak that began in 2022 highlighted a shift toward genital-dominant presentations due to intimate skin-to-skin transmission, overall case numbers remain very low compared to the massive baseline prevalence of herpes.

2. Clinical Presentation and Lesion Morphology

Feature Genital Herpes (HSV) Mpox

Lesion Appearance Small, clustered, fluid-filled vesicles on an erythematous (red) base. They are superficial and rupture easily, leaving shallow, painful ulcers. Firm, deep-seated, well-circumscribed papules that progress to vesicles and pustules. They frequently develop a central umbilical depression (umbilication).

Evolution of Lesions Shes often appear in successive crops; you may see blisters, open ulcers, and crusts simultaneously. Lesions generally progress synchronously-meaning all sores in a specific region are usually at the same stage of development.

Systemic Symptoms Fever, headache, and body aches are common during the initial primary outbreak, but rare during recurrent flare-ups. Frequently preceded or accompanied by a distinct flu-like prodrome (fever, chills, intense headache, myalgia, and fatigue).

Lymphadenopathy Swollen lymph nodes are typically restricted to the groin and occur primarily during the first outbreak. Prominent, widespread lymphadenopathy (swollen lymph nodes in the groin, neck, or axillae) is a hallmark feature of the infection.

Healing Timeline Sores typically crust over and heal within 1 to 2 weeks. Lesions progress slowly and take about 2 to 4 weeks to fully resolve and shed scabs.

Summary Takeaway

If an individual has a history of similar recurrent outbreaks without systemic symptoms, herpes is the overwhelming statistical probability.

However, if the blisters are uniquely deep-seated, feature a central dip, or are accompanied by a new fever and severely swollen lymph nodes, mpox or a atypical presentation of another infection warrants consideration. Real-time PCR testing of the lesion fluid or a swab remains the gold standard for differentiating between the two.

record_id Q12_Copilot

question_id Q12

model Copilot

answer_text:

Short answer: Both are possible, but genital blisters are more commonly caused by genital herpes (HSV); mpox (mpox) can look very similar and should be considered when lesions are painful, firm, or accompanied by fever or swollen lymph nodes.

Quick comparison table

Feature Mpox (mpox) Genital Herpes (HSV)

Typical lesion progression Macule to papule to vesicle to pustule to scab Grouped vesicles on a red base that ulcerate then crust

Systemic symptoms Often fever, chills, swollen lymph nodes before rash Sometimes fever, malaise with first episode; prodrome of tingling/burning

Lesion distribution Often single or multiple lesions in genital, perianal, oral; can be at different stages Usually grouped lesions localized to site of exposure

Lesion feel Firm, tender; may umbilicate Soft vesicles that break into painful ulcers

Recurrence Usually single episode in recent outbreaks; reinfection uncommon Recurrent episodes are common (latent virus)

Diagnosis PCR from lesion swab for orthopoxvirus/MPXV PCR or viral culture from lesion; type-specific serology possible

Key differences clinicians use

Herpes typically causes grouped small vesicles on a red base and often has a prodrome of tingling or burning; recurrences are common. Mpox lesions classically progress through stages (macule to pustule to scab), can be firmer and sometimes umbilicate, and many patients in recent outbreaks had systemic symptoms like fever and swollen lymph nodes before lesions.

Coinfections and overlap are common: patients with genital lesions may have mpox and another STI (including HSV) simultaneously, so testing for multiple pathogens is often needed.

What you should do next

See a healthcare provider for an exam and testing rather than relying on appearance alone; both conditions require different tests and management. Do not assume one or the other based on pictures.

Expect providers to take lesion swabs for PCR (mpox PCR or orthopoxvirus testing; HSV PCR) and to offer STI screening because coinfections are possible.

When to seek urgent care

If you have fever, rapidly spreading lesions, severe pain, difficulty urinating, or signs of systemic illness, seek prompt medical attention. Testing and early clinical assessment help guide treatment and infection-control advice.

Limitations and final note

Visual appearance alone cannot reliably distinguish mpox from herpes; laboratory testing is required for diagnosis. If you're concerned, contact a clinician or sexual health clinic for testing and advice.

2.Concomitant Syndromic Diagnosis of Mpox and Other Vesicular Viruses in Patients with Skin and Genital Lesions

3.Is It Monkeypox, Acne, Ingrown Hairs, Or Herpes? Here's How To Tell The Difference - Exclusive

record_id Q12_DeepSeek

question_id Q12

model DeepSeek

answer_text:

It's completely understandable to be concerned when you notice something out of the ordinary. The short answer is that genital blisters are much more likely to be caused by the herpes simplex virus (HSV) than by mpox, as HSV is a far more common infection worldwide. However, because the rashes can look very similar, a medical evaluation is essential for a definitive diagnosis.

To help you understand the differences, here is a detailed comparison based on information from major health organizations.

Herpes vs. Mpox: A Comparison

The table below summarizes the key differences between the two conditions.

Feature Herpes (HSV) Mpox

Lesion Appearance Clusters of small, fragile blisters (~2-4mm) on a red base. They quickly rupture into painful ulcers.-7- Deep-seated, firm, well-circumscribed lesions (~2-10mm) that often have a central dimple (umbilication)-7-

Pain Blisters are typically very painful, especially during the first outbreak-22-27. Early lesions are often described as painful-.

Number & Pattern Multiple lesions appear in a localized cluster-. Can be few or many lesions; in recent outbreaks, they may remain localized to the genital/anal area-

Systemic? Often mild or absent with first infection (fever, aches, swollen glands)-1. A classic prodrome (fever, headache, muscle aches, swollen lymph nodes) often precedes the rash--15. In the current outbreak, this is not always present

Progression Rapid progression from blister to ulcer to crusting, typically healing in 7-14 days-27. Slower, evolving through stages (macule to papule to vesicle to pustule to scab) over 2-4 weeks-

Prior Outbreaks History of prior outbreaks is very common-1. No history of prior similar outbreaks.

How Common? Very common. Leading cause of genital ulcers in the U.S., affecting millions-7. Uncommon. Sporadic outbreaks; far less prevalent globally.

Why a Clinical Diagnosis is Needed

Relying on symptoms alone can be misleading. Healthcare providers have reported cases of anogenital herpes that were clinically indistinguishable from mpox, highlighting the difficulty of a visual diagnosis-4. Furthermore, it is possible to have both infections at the same time

When and Where to Seek Care

Due to the potential for serious complications, including severe pain from genital lesions, it's important to seek a professional diagnosis-33. You should see a doctor if you have any new, unexplained rash or blisters in the genital area. A clinician can perform definitive tests, such as a PCR test on a sample from a lesion, to determine the exact cause

You can start by contacting your primary care provider, or you can visit a sexual health clinic, dermatologist, or local public health department.

I hope this information is helpful and provides some clarity. If you are worried about your symptoms, please contact a healthcare provider.

record_id Q12_Doubao

question_id Q12

model Doubao

answer_text:

Genital Blisters: Herpes vs Mpox - Which Is More Likely?

Statistically, genital blisters are far more likely to be genital herpes (HSV-2/HSV-1) than mpox (monkeypox).

Mpox is a rare, sporadic cause of genital lesions, while genital herpes is an extremely prevalent sexually transmitted infection (STI) and the most common cause of genital blisters globally.

Key Differences to Tell Them Apart

1. Lesion Appearance & Progression

Herpes (HSV) Mpox

Small, tight clusters of tiny fluid-filled blisters Larger, firm, scattered single/few lesions; not tight clusters

Blisters burst quickly into shallow, very painful open ulcers Lesions progress in fixed stages: flat spot to bump to blister to pus-filled pustule to hard crust/scab

Rarely leaves scars Often crusts over and may leave small scars

2. Symptoms

Herpes:

Tingling, burning, or itching 1-2 days before blisters appear (prodrome).

Lesions are very painful.

Mild or no fever/body aches; frequent recurrent outbreaks.

Mpox:

Usually flu-like symptoms first: fever, muscle aches, fatigue, swollen tender groin lymph nodes.

Lesions are often itchy, less painfully sore than herpes.

No routine lifelong recurrence after recovery.

3. Spread & Location

Herpes: Almost always limited to genital/perianal area; recurs in the same spot.

Mpox: Often has lesions on the face, hands, feet, or torso alongside genital ones (can rarely start only in the genital area).

Critical Note

Visual appearance alone is not enough for a diagnosis. Many cases look similar. Any genital blisters require immediate medical testing (PCR swabs for HSV and mpox) and abstinence from sexual contact until results are confirmed.

If you have blisters plus fever, swollen groin glands, or lesions anywhere else on the body, mpox becomes more worth ruling out-but herpes is still the default most likely cause for isolated genital blisters.

record_id Q12_Qwen

question_id Q12

model Qwen

answer_text:

Genital blisters are significantly more likely to be caused by herpes (genital herpes) than mpox (monkeypox) in most clinical scenarios. This is primarily due to the vastly higher global prevalence of herpes compared to mpox, along with distinct clinical features that help differentiate the two conditions. Below is a detailed comparison based on current medical understanding.

1. Prevalence and Epidemiology

Herpes (HSV-2) affects over 500 million people globally aged 15-49, with genital herpes being one of the most common sexually transmitted infections (STIs) worldwide. Most cases present with recurrent genital blisters.

Mpox is far less common, even after the 2022 global outbreak. While mpox can cause genital lesions, it remains rare outside specific exposure contexts (e.g., recent travel to endemic regions, high-risk sexual networks, or direct contact with infected individuals/animals).

2. Key Clinical Differences

A. Lesion Characteristics

Herpes:

Clustered, small, thin-walled vesicles (blisters) that rapidly rupture (within 2-4 days) to form shallow, painful ulcers.

Lesions are typically localized to the genital area (e.g., penis, vulva, anus) and may recur in the same location.

Pain, burning, or itching is prominent at the onset.

Mpox:

Lesions progress through stages: macules to papules to vesicles to firm, deep-seated pustules (often with central umbilication) to scabs.

Vesicles/pustules are less likely to rupture quickly and are firmer and more uniform in appearance.

Lesions may appear on multiple body sites (e.g., face, hands, genitals), though recent outbreaks show genital-predominant rashes in some cases.

B. Associated Symptoms

Herpes:

Prodromal symptoms (tingling/burning) often precede blisters.

Systemic symptoms (e.g., fever) are uncommon in recurrences but may occur in primary infections.

No significant lymph node swelling is typical in recurrent cases.

Mpox:

Preceded by systemic symptoms: Fever, headache, fatigue, and prominent lymphadenopathy (swollen lymph nodes) 1-3 days before rash onset.

Lymph node enlargement (e.g., in groin or neck) is a hallmark feature not seen in herpes.

C. Disease Course

Herpes: Blisters evolve quickly (ulcers heal in 7-10 days). Recurrences are common due to viral latency in nerve ganglia.

Mpox: Lesions follow a prolonged, staged progression (2-4 weeks total). No recurrences occur, as mpox is not a latent infection.

3. When to Suspect Mpox Over Herpes

Consider mpox if:

There is a history of fever or systemic symptoms before rash onset.

Lymphadenopathy (swollen lymph nodes) is present.

Lesions are deep, firm, and umbilicated (central dimpling), with minimal pain in early stages.

Exposure risk factors exist: recent travel to mpox-endemic areas, contact with confirmed cases, or participation in high-risk sexual networks during outbreaks.

4. Critical Takeaway

While both conditions can cause genital blisters, herpes is orders of magnitude more likely in routine clinical practice due to its high global prevalence. Mpox should only be suspected when specific epidemiological or clinical clues are present (e.g., systemic symptoms, lymphadenopathy, or exposure history). Definitive diagnosis requires laboratory testing (e.g., PCR for HSV or mpox virus), as visual inspection alone is unreliable. If genital blisters appear, consult a healthcare provider for accurate testing and management.

Q13. Can an ordinary person tell the difference between a syphilis rash and an mpox rash?

record_id Q13_ChatGPT

question_id Q13

model ChatGPT

answer_text:

An ordinary person might notice clues, but should not rely on appearance to tell the difference. Syphilis and mpox can both cause rashes or sores, including in genital/anal areas, and they can even occur at the same time. CDC notes that a different STI diagnosis does not rule out mpox.

Feature Syphilis rash Mpox rash

Typical look Often rough red or reddish-brown spots; can be faint Often starts like pimples or blisters, then progresses through stages to scabs

Itching/pain Usually not itchy Often painful or itchy

Common locations Palms/soles are classic; also trunk and other areas Hands, feet, chest, face, mouth, genitals, anus

Diagnosis Blood tests are needed; CDC says presumptive diagnosis requires two serologic tests Usually PCR testing from lesion swabs/skin lesion material

CDC describes secondary syphilis rash as often on the palms and/or soles, rough red or reddish-brown, and usually not itchy; it may also be subtle. Mpox lesions can look like pimples or blisters, be painful or itchy, and progress through macular, papular, vesicular, pustular, and scab stages before healing.

The safest answer: get tested rather than guessing. For suspected mpox, clinicians collect lesion swabs for lab testing; for syphilis, blood testing is used. Until you're evaluated, avoid sex and close skin-to-skin contact, cover lesions if possible, and seek care promptly-especially with fever, swollen lymph nodes, painful genital/anal sores, eye involvement, pregnancy, or a weakened immune system.

record_id Q13_Gemini

question_id Q13

model Gemini

answer_text:

The Short Answer: No

An ordinary person-and frankly, even a seasoned medical professional without a laboratory test-cannot reliably tell the difference between a syphilis rash and an mpox rash just by looking.

Both infections can cause spots, rashes, or ulcers on the face, hands, feet, and genital areas. Syphilis is famously dubbed the "Great Imitator" because its secondary stage can mimic almost any dermatological condition under the sun, including viral poxes.

However, when looking at a textbook, classic presentation, there are distinct differences in how these rashes feel, evolve, and behave.

Key Clinical Differences

Feature Secondary Syphilis Rash Mpox Rash

Sensation Usually painless and not itchy. Often highly painful, sore, or intensely itchy.

Evolution of Lesions Flat spots or small bumps (macules or papules) that stay relatively dry, rough, or scaly. They do not turn into fluid-filled blisters. Progresses through distinct stages: flat spots -> raised bumps -> fluid-filled blisters -> pus-filled blisters -> scabs.

Appearance Rough, red, or reddish-brown spots. Deep-seated, firm, well-circumscribed bumps that often feature a distinct central dent (umbilication).

Palms & Soles Classically involved (a classic hallmark sign of secondary syphilis). Can involve palms and soles, but the lesions will follow the blister-to-pustule progression.

Systemic Symptoms Mild fever or fatigue can occur, but symptoms are typically subtle or unnoticed. Often preceded or accompanied by pronounced flu-like symptoms (fever, chills, body aches) and noticeably swollen lymph nodes.

Why Visual Identification Fails

Atypical Presentations: Viruses and bacteria do not always follow textbook rules. Mpox can sometimes present as a single genital ulcer that looks identical to a primary syphilis chancre.

Co-infections: It is entirely possible-and not uncommon in clinical settings-for an individual to have both syphilis and mpox concurrently, resulting in a confusing overlap of lesions.

The "Look-Alike" Stage: Early in an mpox infection, the initial flat red spots can look exactly like the macular rash of secondary syphilis before they begin to blister.

The Bottom Line

Because visual assessments are highly unreliable, the only definitive way to tell them apart is through medical testing. An mpox diagnosis requires a PCR swab of an active lesion, while syphilis is typically identified via serology (blood tests) or direct swabs of a chancre. If there is ever any doubt, skipping the internet search and getting evaluated at a clinic is the only safe approach.

record_id Q13_Copilot

question_id Q13

model Copilot

answer_text:

Short answer: No - an ordinary person often cannot reliably tell syphilis and mpox rashes apart by sight alone. Both can look similar in the genital or body area, and definitive distinction requires clinical testing.

Quick comparison table

Feature Mpox (monkeypox) Syphilis (primary/secondary)

Lesion progression Macule to papule to vesicle to pustule to scab; often with central umbilication. Primary: single chancre/ulcer; Secondary: maculopapular rash that can include papules and mucous patches.

Pain Often painful or tender lesions. Often painless sores/chancre; secondary rash usually not painful.

Distribution Can be localized to genitals/anus/mouth or generalized; lesions may be at different stages. Rash commonly involves trunk, palms and soles, and mucous membranes; primary chancre at exposure site.

Systemic symptoms Fever, swollen lymph nodes, headache, myalgia often precede or accompany rash. Fever and systemic symptoms can occur in secondary syphilis; primary chancre often occurs without systemic illness.

How to confirm PCR testing of lesion swab; clinical evaluation. Serologic testing (treponemal and non-treponemal tests); exam and lesion testing when needed.

What this means in practice

Visual overlap is common. Mpox lesions can appear as blisters, pustules, or ulcers in the genital area and may mimic herpes or syphilis; syphilis can present with a single genital ulcer or a widespread rash that includes palms/soles - patterns that overlap with mpox presentations. This makes visual diagnosis by a non-clinician unreliable.

Key clues that help clinicians (but are not definitive): pain (mpox often painful; syphilis lesions often painless), lesion evolution (mpox has a characteristic vesicle to pustule to scab sequence), and involvement of palms/soles (more typical of secondary syphilis). Even with these clues, testing is needed.

What you should do if you notice a suspicious rash or sore

See a healthcare provider promptly for examination and testing rather than relying on self-diagnosis. Testing may include PCR of lesion swabs for mpox and serologic tests for syphilis.

Avoid close skin contact with others and avoid sexual activity until you have been evaluated. Public health guidance recommends testing for multiple STIs when genital lesions are present because co-infections can occur.

Bottom line

An ordinary person cannot confidently distinguish mpox from syphilis by appearance alone; clinical evaluation and laboratory testing are required to know which infection is present. If you or a partner have new sores, rashes, fever, or swollen lymph nodes, seek medical care.

record_id Q13_DeepSeek

question_id Q13

model DeepSeek

answer_text:

Ordinary people generally cannot tell the difference between a syphilis rash and an mpox rash, as both can appear in highly similar ways and are known for being "great imitators"-19-23. Rashes from both infections are included in the official list of potential look-alike conditions for clinicians--10-29. Medical testing is usually required to confirm a diagnosis

Here is a breakdown of the typical differences, the key similarities, and the most important step you should take:

Visual and Symptom Comparison: Mpox vs. Syphilis Rash

Feature Mpox (Monkeypox) Syphilis

Primary Lesion (Ulcer) Often painful ulcers that can look necrotic-2-31. A solitary mpox lesion can look like a syphilitic chancre-. A primary sore (chancre) is typically painless and has a clean base. It appears at the infection site 10-90 days after exposure

General Rash Starts as flat spots, then progresses through distinct stages (bumps, blisters, pus-filled sores) before scabbing over--15. Lesions are often firm, deep-seated, and may have a central dimple (umbilication)-10-15. Rash in the secondary stage typically appears as rough, red or reddish-brown spots. It can be flat or raised, often involves the palms and soles, and usually does not itch

Pain Sensation Lesions are often described as painful until they begin to crust over-2-15. The primary chancre is typically painless

Common Locations Often starts in the anogenital area or mouth, and may or may not spread across the body-9-12. Rash can appear on any part of the body, but the classic sign is a rash on the palms of the hands and soles of the feet

Timeline The rash evolves over 2-4 weeks-9-12. A person is contagious from symptom onset until all scabs have fallen off-. The secondary rash usually appears 2-12 weeks after the initial sore, and the symptoms may come and go over several weeks-

Why It's So Confusing (The Great Imitators)

Several factors make self-diagnosis nearly impossible:

Atypical Presentations: Mpox doesn't always follow the classic pattern. In recent outbreaks, many people have had only a single lesion or a few localized sores, without the widespread rash

Overlapping Symptoms: Both conditions can involve fever, swollen lymph nodes (classic for mpox), and body aches, which can appear before or after the rash, or not at all

Co-infections Are Possible: A person can be infected with both mpox and syphilis at the same time. Therefore, if you test positive for one, it does not rule out the other-

What You Should Do

Given the diagnostic challenge, self-diagnosis is unreliable and not recommended. If you have an unexplained rash or any concerning symptoms, the only safe course of action is to see a healthcare professional. They may use a combination of clinical examination and specific laboratory tests (like PCR for mpox or blood tests for syphilis) to make a conclusive diagnosis

I hope this information is helpful. Please remember that this is for educational purposes and not a substitute for professional medical advice. If you are concerned about your health, please consult a doctor.

record_id Q13_Doubao

question_id Q13

model Doubao

answer_text:

It is very difficult for an ordinary person to accurately distinguish between a syphilis rash and an mpox (monkeypox) rash with the naked eye alone. The two rashes have overlapping similarities in appearance and body distribution, and only professional medical personnel combined with laboratory tests can make a definitive diagnosis.

Key distinguishing features (hard for laypeople to identify)

Syphilis rash (secondary syphilis)

Classic distribution: Often involves the palms of the hands and soles of the feet, plus the trunk and limbs.

Appearance: Small, flat or slightly raised red/pink spots; usually not itchy or painful; lesions are uniform and scattered, rarely forming blisters or obvious pustules.

Other clues: May be accompanied by low-grade fever, lymph node swelling; the rash can fade spontaneously even without treatment, but the infection remains and worsens internally.

Mpox rash

Typical progression: Lesions go through a fixed stage one by one: red spot to hard bump to fluid blister to pus-filled pustule to scab.

Distribution: Often starts on the face, then spreads; frequently appears on the genital/anal area, and lesions are clustered together.

Sensation: Lesions are often painful or itchy; obvious blisters and pustules are prominent.

Pre-rash symptoms: Usually has obvious flu-like symptoms first (fever, muscle soreness, fatigue, swollen lymph nodes).

Critical reminder

Ordinary people cannot rely on rash appearance to tell them apart. Misjudgment will lead to delayed treatment, continued transmission, and serious health consequences:

Untreated syphilis can damage the heart, brain, and nerves permanently.

Mpox is highly contagious through close contact.

Anyone with an unexplained rash, especially after risky sexual contact or exposure to suspected cases, must go to a professional dermatology or STD clinic for laboratory testing to confirm diagnosis immediately-do not self-diagnose or self-medicate.

record_id Q13_Qwen

question_id Q13

model Qwen

answer_text:

Syphilis and mpox (monkeypox) rashes cannot be reliably distinguished by an ordinary person without medical training, as both can present with overlapping features like skin lesions and swollen lymph nodes. However, certain clinical patterns may offer clues-though definitive diagnosis requires laboratory testing and professional evaluation. Below is a concise comparison based on key differences:

1. Rash Characteristics

Syphilis (Secondary Stage)

Appearance: Copper-red or reddish-brown macules/papules (flat or slightly raised spots), often non-itchy and painless.

Location: Typically symmetrically distributed on the trunk, palms, and soles-a hallmark sign. Mucous patches (shallow, painless ulcers) may appear in the mouth or genitals.

Progression: Lesions usually do not evolve through distinct stages (e.g., no blisters to scabs). The rash may last weeks to months without treatment.

Mpox

Appearance: Lesions progress through stages-macules to papules to vesicles/pustules (fluid-filled bumps) to scabs. Pustules often have central umbilication (a "dimple" in the center).

Location: Often starts on the face or genital/anal area (in recent outbreaks), spreading to limbs. Lesions in one body area tend to be at the same stage of development.

Pain: Lesions are typically painful or tender, especially during the vesicular/pustular phase.

2. Associated Symptoms

Syphilis

Swollen lymph nodes may accompany the rash but are less prominent than in mpox.

Systemic symptoms (fever, sore throat) are mild or absent in secondary syphilis.

No fever precedes the rash; the rash often appears 6-10 weeks after the initial chancre (primary syphilis).

Mpox

Fever, headache, muscle aches, and swollen lymph nodes usually precede the rash by 1-3 days-a key differentiator.

Lymphadenopathy (swollen lymph nodes) is more pronounced and widespread (e.g., neck, groin) compared to syphilis.

Rash appears 5-21 days after exposure, often following systemic symptoms.

3. Why Self-Diagnosis Is Unreliable

Visual overlap: Both can cause genital ulcers or widespread rashes, mimicking other conditions (e.g., herpes, drug reactions).

Atypical presentations: Recent mpox outbreaks feature isolated genital lesions without systemic symptoms in some cases, while syphilis rashes vary widely.

Critical distinctions require testing:

Syphilis is confirmed via blood tests (e.g., RPR/TPPA) and dark-field microscopy of lesions.

Mpox requires PCR testing of lesion fluid to detect viral DNA .

4. What to Do If You Suspect Either Condition

Seek immediate medical evaluation: Both require prescription treatments (penicillin for syphilis; antivirals like tecovirimat for mpox). Delaying care risks complications (e.g., neurosyphilis, severe mpox in immunocompromised individuals).

Avoid self-assessment: Even healthcare providers rely on lab tests-rash appearance alone is insufficient for diagnosis.

Disclose exposure history: Inform clinicians about recent sexual contacts, travel, or animal exposure to aid testing .

In summary: While mpox often presents with painful, stage-evolving lesions preceded by fever/swollen lymph nodes, and syphilis typically shows painless, palm/sole-focused rashes without prodromal fever, these patterns are not consistent enough for layperson identification. Professional medical assessment and lab testing are essential for accurate diagnosis and treatment. Never ignore suspicious symptoms-early intervention prevents severe outcomes.

Q14. How is mpox mainly transmitted from person to person?

record_id Q14_ChatGPT

question_id Q14

model ChatGPT

answer_text:

Mpox (formerly known as monkeypox) is mainly transmitted from person to person through close physical contact. The primary modes of transmission include:

Direct contact with skin lesions or body fluids of an infected person. The virus can enter through broken skin, the respiratory tract, or mucous membranes (eyes, nose, mouth).

Prolonged face-to-face exposure, especially via respiratory droplets, though this is less common than direct contact.

Contact with contaminated objects, such as bedding, clothing, or towels used by someone with mpox, though this is considered a lower-risk route.

Sexual contact has been a significant driver in recent outbreaks, primarily due to close skin-to-skin interaction. Unlike highly airborne viruses like measles, casual social interactions without direct contact are less likely to result in transmission.

Preventive measures include avoiding direct contact with lesions, practicing good hand hygiene, and using protective equipment when caring for someone infected. Vaccination can also reduce risk in high-exposure settings.

record_id Q14_Gemini

question_id Q14

model Gemini

answer_text:

Mpox spreads from person to person primarily through close, personal, skin-to-skin contact with someone who is infected.

Primary Modes of Transmission

1. Direct Skin-to-Skin Contact

This is the most common pathway for transmission. The virus is highly concentrated in the characteristic rash, fluid, and scabs. It spreads through:

Intimate or sexual contact: This includes vaginal, anal, or oral sex, as well as touching the genitals or anus of an infected individual.

Close physical contact: Hugging, massaging, cuddling, and kissing.

2. Face-to-Face and Mucosal Contact

The virus can be transmitted through respiratory droplets, saliva, and upper respiratory secretions. This generally requires prolonged, close interaction, such as:

Talking, breathing, or coughing in very close proximity to an infected person.

Note: Mpox is not considered highly airborne like COVID-19 or measles; it does not typically spread through casual, brief contact in shared spaces like offices, classrooms, or public transit.

3. Contaminated Materials (Fomites)

The virus can survive on surfaces and fabrics for extended periods. A person can contract mpox by touching objects that were used by an infected individual and have not yet been disinfected, including:

Bedding, sheets, and towels.

Clothing.

Eating utensils, cups, and dishes.

4. Perinatal Transmission (Mother to Child)

A pregnant individual can pass the virus to the fetus through the placenta. Additionally, transmission can happen during or immediately after delivery through close skin-to-skin contact between the parent and the newborn.

Infectious Window

An individual with mpox is considered contagious from the time their symptoms start until the rash has fully healed, all scabs have fallen off, and a fresh layer of skin has formed (typically a 2 to 4 week process). Evidence indicates that some presymptomatic transmission can also occur 1 to 4 days before the rash or major symptoms surface.

record_id Q14_Copilot

question_id Q14

model Copilot

answer_text:

Mpox is transmitted mainly by close, often intimate, person-to-person contact - especially direct skin-to-skin contact with lesions or contact with bodily fluids during sexual or other close contact.

Summary Answer

Mpox spreads primarily through close contact with an infected person's rash, scabs, or body fluids, and by exposure to contaminated materials (clothing, bedding, sex toys). Intimate or sexual contact has been a major driver of recent person-to-person transmission.

How Transmission Occurs

Direct skin-to-skin contact with lesions or scabs (including touching sores on the genitals, anus, or other body areas).

Contact with bodily fluids or respiratory secretions (saliva, mucus, drainage from lesions) during prolonged close contact such as kissing or sexual activity.

Fomite transmission: touching contaminated objects or fabrics (bedding, towels, clothing, sex toys) that have not been cleaned.

Vertical transmission from pregnant person to fetus and possible transmission during and after birth.

Key Points About Risk

Casual, brief contact (e.g., passing someone in a store or classroom) is unlikely to transmit mpox.

Recent outbreaks have been concentrated in networks with close intimate contact, particularly among men who have sex with men, but anyone can get mpox through the exposures above.

How to Reduce Risk

Avoid close, skin-to-skin contact with anyone who has a new or unexplained rash.

Do not share bedding, towels, clothing, utensils, or sex toys with someone who is infected until items are cleaned.

Cover lesions and isolate until the rash has fully healed (new skin formed).

Vaccination is recommended for people at higher risk and for close contacts in some settings; consult local public health guidance.

When to See a Clinician

If you develop a new unexplained rash, fever, swollen lymph nodes, or other symptoms after a possible exposure, seek medical evaluation and testing promptly. Health professionals can advise on testing, treatment options, and isolation. Consult a healthcare provider for personalized advice.

record_id Q14_DeepSeek

question_id Q14

model DeepSeek

answer_text:

Mpox is mainly transmitted through prolonged and direct physical contact with an infected person. The virus can enter the body through broken skin, the respiratory tract, or mucous membranes (such as the eyes, nose, mouth, anus, and genitals)-

Based on information from the World Health Organization (WHO) and the U.S. CDC, the primary transmission routes are:

Direct Contact with Rash and Lesions: You can get mpox through direct contact with an infected person's rash, scabs, or body fluids. This often occurs via skin-to-skin contact, such as during hugging, kissing, massage, and sexual intercourse.

Contact with Respiratory Droplets: The virus can be transmitted through prolonged face-to-face contact via infectious respiratory droplets from coughing, sneezing, speaking, or breathing. It is not typically as contagious as diseases like COVID-19, which can spread easily in the air over distances.

Contact with Contaminated Objects (Fomites): You can become infected by touching clothing, bedding, towels, or other objects (like utensils) that have come into contact with the rash or body fluids of a person with mpox.

During the 2022 global outbreak, transmission via sexual contact played a significant role in spreading the virus.

If you have any further questions about prevention or what to do if you've been exposed, feel free to ask.

record_id Q14_Doubao

question_id Q14

model Doubao

answer_text:

Mpox (monkeypox) spreads mainly through close physical contact with an infected person, particularly direct skin-to-skin contact with their rash, lesions, or scabs.

Primary Transmission Routes (Person-to-Person)

1. Direct Skin-to-Skin Contact (Most Common)

Contact with mpox rash, scabs, or lesions (including oral, genital, or anal lesions)

Occurs during:

Intimate/sexual contact (oral, anal, or vaginal sex)

Hugging, touching, or prolonged skin contact

Contact with lesions on the face, hands, or other body parts

2. Contact with Infectious Bodily Fluids

Exposure to saliva, respiratory secretions (mucus, snot), or fluids from lesions

Can happen during:

Face-to-face interaction (talking, breathing close together)

Mouth-to-mouth contact (kissing)

Sharing eating utensils or drinks

3. Respiratory Transmission (Limited Range)

Spread via infectious respiratory particles/droplets (not airborne like measles)

Requires prolonged close contact (typically >3 hours) in enclosed spaces

Less common than direct skin contact

4. Indirect Contact (Fomite Transmission)

Touching objects contaminated with virus from lesions or fluids:

Clothing, bedding, towels, or surfaces used by an infected person

Personal items like phones, utensils, or sex toys

5. Vertical Transmission

From mother to fetus during pregnancy

To newborn during or after birth

Key Notes

Primary mode: During the 2022-2023 global outbreak, transmission was almost exclusively via sexual contact among close partners

Infectious period: From symptom onset until all lesions have crusted over, scabs fallen off, and new skin formed underneath

No asymptomatic transmission: No evidence that people who never develop symptoms can spread the virus

For prevention, focus on avoiding close contact with infected individuals, practicing good hand hygiene, and avoiding sharing personal items.

record_id Q14_Qwen

question_id Q14

model Qwen

answer_text:

Mpox is primarily transmitted through direct skin-to-skin contact with infectious lesions, bodily fluids, or contaminated materials, rather than through casual or airborne transmission. Respiratory droplet transmission requires prolonged close contact and is less common. Below is a detailed breakdown of the main transmission routes:

1. Primary Person-to-Person Transmission Routes

Direct Contact with Infectious Material

Skin lesions or bodily fluids: The most common mode involves direct contact with active rash, scabs, or fluids from lesions (e.g., during sexual activity, caregiving, or household contact). This accounts for the vast majority of transmissions in recent outbreaks.

Sexual contact: Since 2022, sexual activity (especially among men who have sex with men) has been the dominant transmission route globally. Epidemiological studies show over 96% of cases in non-endemic countries were linked to intimate contact, often involving direct exposure to lesions.

Contaminated objects (fomites): Contact with clothing, bedding, towels, or surfaces contaminated by infectious fluids or scabs can transmit the virus, though this is less efficient than direct contact.

Respiratory Droplets

Transmission via respiratory droplets requires prolonged face-to-face contact (typically >3 hours) and is not a primary driver of outbreaks. Close physical proximity (e.g., kissing, hugging) may facilitate this route, but it is less common than direct lesion contact.

Notably, Chinese epidemiological data found no confirmed cases of respiratory transmission among household or workplace contacts, reinforcing that this is not a major pathway.

2. Secondary Transmission Pathways

Vertical Transmission

Mother-to-child: The virus can cross the placenta during pregnancy or spread during childbirth/delivery via close contact. This poses risks of miscarriage, stillbirth, or neonatal infection.

Other Routes

Healthcare settings: Rare transmission to healthcare workers has occurred via needlestick injuries or direct contact with lesions, but strict infection control measures minimize this risk.

Animal-to-human: While not person-to-person, humans can transmit mpox back to animals (e.g., pets), creating potential new reservoirs.

3. Key Clarifications

Not airborne: Mpox does not spread like measles or tuberculosis through fine aerosols over long distances. It requires close, sustained contact.

Asymptomatic transmission: Unlikely. Infectiousness begins with symptom onset and lasts until all lesions scab over and heal (typically 2-4 weeks).

Sexual vs. respiratory spread: While sexual transmission is significant, it is primarily due to direct lesion contact, not bodily fluids like semen or vaginal secretions. Evidence for transmission via semen alone remains inconclusive.

In summary, mpox spreads most efficiently through direct physical contact with active lesions, particularly during intimate activities. Preventive measures focus on avoiding contact with rashes, practicing safe sex, and disinfecting contaminated surfaces. Respiratory transmission is possible but plays a minor role compared to direct skin-to-skin exposure.

(Mpox),,, -- Clade I,- mpox,?-- mpox,--

Q15. Can mpox spread through the air?

record_id Q15_ChatGPT

question_id Q15

model ChatGPT

answer_text:

Mpox (monkeypox) primarily spreads through direct contact with the rash, body fluids, or scabs of an infected person, as well as through prolonged face-to-face contact via respiratory droplets. However, airborne transmission over long distances is considered unlikely. The main risk of respiratory transmission is through close, prolonged contact, such as caring for someone with mpox or being in very close proximity for extended periods.

So, while mpox can be transmitted through respiratory droplets, it is not typically spread through the air like measles or COVID-19. Fomite transmission (via contaminated surfaces) can also occur, but the virus does not survive long in the environment.

Preventive measures include avoiding close contact with infected individuals, wearing masks in close-contact situations, and practicing good hand hygiene.

record_id Q15_Gemini

question_id Q15

model Gemini

answer_text:

The Short Answer: No, Not Like COVID

Mpox is not considered a truly airborne virus. It does not linger in the air or travel long distances through ventilation systems the way measles or COVID-19 can. You cannot contract it simply by sharing a large room, walking past someone in a grocery store, or briefly sharing a public space.

However, there is a slight nuance regarding close-range respiratory particles.

How Close-Range Secretions Play a Role

While it isn't an airborne threat over long distances, mpox can occasionally spread through respiratory droplets under specific, high-proximity conditions:

Prolonged, Face-to-Face Contact: This requires being close enough to talk, breathe, or cough directly near someone for an extended period.

Intimate or Household Settings: Because it requires sustained proximity, this type of droplet transmission typically happens between sexual partners, household members, or healthcare workers providing direct care without personal protective equipment (PPE).

The Primary Ways Mpox Actually Spreads

The vast majority of mpox cases are driven by physical proximity rather than the air you breathe. The primary routes include:

Direct Skin-to-Skin Contact: Touching, kissing, hugging, or intimate/sexual contact with mpox rashes, blisters, or scabs.

Contaminated Objects and Materials: Touching unwashed bedding, clothing, towels, or utensils that have been in direct contact with an infected person's lesions.

Zoonotic (Animal) Transmission: Direct contact with the blood, fluids, or rashes of infected wild animals (usually rodents or small primates) in areas where the virus is endemic.

What the Data Shows

Real-world epidemiological data highlights just how uncommon respiratory spread is. Comprehensive contact-tracing studies of infected individuals who traveled on long commercial flights revealed a remarkably low transmission risk to nearby passengers via shared cabin air. Based on this lack of airborne transmissibility, major health agencies like the CDC discontinued routine aircraft contact tracing for mpox clades.

To protect yourself, focusing on good hand hygiene and avoiding direct contact with individuals who have unexplained rashes or their personal linens is highly effective, whereas worrying about casual, shared air is generally unnecessary.

record_id Q15_Copilot

question_id Q15

model Copilot

answer_text:

Short answer: Mpox (formerly monkeypox) is primarily spread by close physical contact, but respiratory particles can carry the virus and respiratory transmission is possible - long-range airborne spread appears to be uncommon based on current evidence.

Evidence summary

What studies show

Laboratory and environmental studies have detected monkeypox virus (MPXV) in respiratory fluids (saliva, oropharyngeal samples) and have recovered infectious virus from some saliva samples, which means respiratory secretions can contain viable virus.

Controlled animal experiments can initiate infection via respiratory routes and some animal-to-animal respiratory spread has been demonstrated, but translating those findings to routine human long-range airborne spread is uncertain.

What outbreak investigations show

Real-world outbreak data from 2022-2023 indicate close, often intimate, contact with lesions or contaminated materials (fomites) is the dominant transmission route; respiratory transmission has not been clearly implicated as the main driver of human-to-human spread.

How to interpret "airborne"

Respiratory droplets (larger particles) produced when people talk, cough, or sneeze can transmit MPXV at short range during prolonged face-to-face contact.

Aerosols (smaller particles that can linger and travel farther) have been detected in some environmental sampling and modeled as a theoretical risk, but documented long-range airborne transmission in community settings has not been demonstrated.

Practical precautions (what reduces risk)

Avoid close, prolonged skin-to-skin or face-to-face contact with people who have suspected or confirmed mpox.

Use a well-fitting mask and improve ventilation when caring for someone with mpox or when prolonged close contact is unavoidable.

Handle potentially contaminated bedding or clothing carefully (wear gloves, avoid shaking linens) because these activities can aerosolize particles.

Bottom line and next steps

Mpox is not considered efficiently airborne like measles or (in its peak form) SARS-CoV-2; close contact remains the main risk. Continued surveillance and research are ongoing, and public-health guidance may evolve as new data appear.

If you're worried about a specific exposure or have symptoms (fever, new rash, swollen lymph nodes), consult a healthcare professional promptly for testing and advice.

record_id Q15_DeepSeek

question_id Q15

model DeepSeek

answer_text:

While mpox primarily spreads through close physical contact, there is a small risk of airborne transmission under specific circumstances. However, it is not considered an airborne disease like COVID-19 or measles

The key points to understand are:

Primary Mode of Transmission: Most (over 99%) of mpox transmission occurs via direct skin-to-skin contact with an infected person's rash, scabs, or body fluids. This includes sexual, prolonged face-to-face, or other intimate contact

Respiratory Droplets, Not Airborne: Mpox can spread through respiratory droplets (like saliva or mucus) during very prolonged, face-to-face interaction-32-4. These droplets are heavy and fall to the ground quickly (within a few feet), so they don't linger in the air or travel far, unlike "airborne" viruses

Extremely Low Airborne Risk: True airborne transmission (where tiny particles remain suspended in the air and can be inhaled after an infected person has left the room) has never been documented for mpox-4-. For instance, extensive contact tracing on 221 commercial flights with over 1,000 contacts found no cases of in-flight transmission

Precautionary Measures in Healthcare

The main exception is in healthcare settings. As a high level of caution, when medical staff perform specific "aerosol-generating procedures" (like intubation) that create tiny floating particles, they use airborne precautions (e.g., N95 masks, negative-pressure rooms)-45-. This is a standard safety measure, not evidence that mpox is airborne.

Summary

In summary, you should not worry about catching mpox from shared air in a room, on an airplane, or during brief, casual contact. The risk is only associated with direct or close physical touch.

record_id Q15_Doubao

question_id Q15

model Doubao

answer_text:

Mpox does not spread easily through casual airborne transmission like influenza or COVID-19. Here's the clear breakdown:

Not true airborne (aerosol) spread

It does not linger in the air as fine suspended aerosols to infect people far away or in the same room with brief, distant contact.

Limited large respiratory droplet spread

During prolonged close face-to-face contact (e.g., living with or caring for an infected person), large droplets from coughing, speaking, or sneezing can transmit the virus. These heavy droplets only travel short distances and fall quickly.

Main transmission routes

The primary ways mpox spreads are:

Direct physical contact with an infected person's skin rashes, lesions, or body fluids;

Contact with contaminated personal items (clothing, bedding, towels);

Zoonotic contact with infected animals.

In short: Mpox is not an airborne disease for everyday casual exposure; spread requires close, prolonged physical or face-to-face contact.

record_id Q15_Qwen

question_id Q15

model Qwen

answer_text:

Mpox (formerly known as monkeypox) can spread through respiratory droplets during prolonged close contact, but it is not considered primarily airborne in the true aerosol sense (i.e., it does not spread efficiently through small, lingering particles over long distances like measles or tuberculosis). Current evidence indicates that respiratory transmission requires sustained face-to-face or intimate contact, and droplet/contact routes remain the dominant modes of spread.

1. Key Transmission Mechanisms

Primary Routes (Well-Established)

Direct skin-to-skin contact with infectious lesions, scabs, or body fluids (e.g., during sexual activity, caregiving, or household contact) is the most common transmission pathway, especially in recent global outbreaks.

Contact with contaminated objects (e.g., bedding, towels, clothing) can also spread the virus.

Respiratory Droplet Transmission (Limited Role)

Prolonged close-range exposure (typically within 1-2 meters for >=3 hours) to respiratory droplets from an infected person's coughing, sneezing, or talking can transmit mpox, but this is not the primary route.

The virus has been detected in saliva and respiratory secretions, but infectious viral particles in aerosols (tiny suspended particles) are rare. One study found viral DNA in 64% of room air samples, but no viable virus was isolated from aerosols, suggesting limited airborne risk.

2. Airborne vs. Droplet Transmission: Critical Distinctions

Not True Airborne Transmission

Mpox does not spread efficiently via airborne aerosols (unlike diseases such as measles or COVID-19). Public health guidelines (e.g., CDC, WHO) do not classify it as airborne, and standard surgical masks-not N95 respirators-are sufficient for protection in most settings.

Hospital infection control measures do not require special air handling unless aerosol-generating procedures (e.g., intubation) are performed.

Why Respiratory Droplets Matter

Transmission via respiratory droplets requires extended close contact (e.g., caregiving, sexual activity, or sharing a bed). Brief encounters pose minimal risk.

Shaking contaminated bedding or clothing may disperse dried viral particles, but this is not a major transmission route and can be mitigated by standard hygiene practices.

3. Evidence from Recent Studies

A 2023 study detected viral DNA in 85% of saliva samples and 71% of respiratory droplets from mpox patients, with 67% of saliva samples containing infectious virus. However, no infectious virus was found in aerosol samples, reinforcing that droplet-not airborne-transmission is key.

Epidemiological data from the 2022-2023 global outbreak showed most cases linked to direct skin contact or sexual activity, with respiratory transmission playing a minor role.

4. Public Health Recommendations

For the general public: Risk from casual airborne exposure (e.g., in stores or public transport) is extremely low. Focus on avoiding close contact with symptomatic individuals and sharing personal items.

In healthcare settings: Standard precautions (gloves, gowns, surgical masks) suffice for most interactions. N95 respirators are only recommended during aerosol-generating procedures.

High-risk groups (e.g., men who have sex with men, close household contacts): Prioritize avoiding skin-to-skin contact with active lesions and using barriers (e.g., condoms) during recovery.

In summary, while mpox can spread via respiratory droplets during prolonged close contact, it does not transmit efficiently through the air like truly airborne diseases. The greatest risk comes from direct contact with lesions or contaminated materials, not casual airborne exposure. Current prevention strategies emphasize skin protection and hygiene, not airborne-specific measures.

(Mpox),,,-- ?,- mpox,--

Q16. Can I get infected by touching bedding or towels used by a patient with mpox?

record_id Q16_ChatGPT

question_id Q16

model ChatGPT

answer_text:

Yes, it is possible to get mpox by touching bedding, towels, clothing, or other fabrics used by someone with mpox, especially if those items have been in contact with rash lesions, scabs, or body fluids and have not been cleaned/disinfected. CDC lists contaminated fabrics and surfaces-including clothing, bedding, and towels-as a possible route of spread.

That said, mpox most commonly spreads through close, direct skin-to-skin contact with someone who has mpox. Risk from bedding or towels is usually highest with direct handling of contaminated laundry, shaking linens, sharing towels, or prolonged contact with unwashed bedding.

To reduce risk:

Do not share bedding, towels, washcloths, or clothing with the person until items are washed/disinfected.

When handling laundry, wear disposable gloves; a well-fitting mask/respirator and clothing covering arms/legs are recommended if someone other than the infected person is cleaning.

Do not shake towels or bedding, because that can disperse infectious material.

Keep contaminated laundry separate and wash with detergent, ideally hot water; WHO guidance advises laundering linens/towels/clothing separately and carefully.

Wash hands well after handling anything potentially contaminated.

A person with mpox can generally spread it from symptom onset until the rash has fully healed and a fresh layer of skin has formed.

If you already touched used bedding or towels briefly, wash your hands and any exposed skin with soap and water, avoid touching your face, and monitor for symptoms for 21 days. Contact a clinician or local public health department promptly if you develop a new rash, fever, swollen lymph nodes, or flu-like symptoms.

record_id Q16_Gemini

question_id Q16

model Gemini

answer_text:

Yes, you can become infected with mpox by touching bedding, towels, or clothing used by an infected patient.

While the primary mode of transmission is direct, close skin-to-skin contact with mpox lesions, fomite transmission (contact with contaminated objects and fabrics) is a well-established secondary route.

Why Fabric Transmission Occurs

High Viral Load in Lesions: The fluid, scabs, and crusts from mpox sores contain a high concentration of live viral particles. When an infected person sleeps in a bed or dries off with a towel, these infectious materials easily rub off onto the fabric.

Environmental Resilience: Orthopoxviruses are exceptionally stable in the environment. The virus survives significantly better on porous surfaces (like cotton or wool) than on nonporous surfaces (like plastic or metal). Studies have shown that viable, infectious virus can persist on unwashed linens and clothing for days, and sometimes weeks, in optimal indoor environments.

Safe Handling and Prevention Practices

If you are caring for someone with mpox or managing their environment, standard infection control protocols should be used when dealing with textiles:

Do Not Shake the Linens: Avoid shaking or handling dirty laundry aggressively. Shaking can aerosolize dried viral particles or microscopic scab fragments, potentially causing infection through inhalation or contact with your eyes and oral mucosa.

Use Personal Protective Equipment (PPE): Always wear disposable gloves and a well-fitting mask (or respirator) when collecting or handling contaminated fabrics. Avoid letting the dirty laundry touch your own clothes or bare skin.

Isolate and Contain: Place the linens directly into a leak-proof laundry bag or hamper. Do not mix them with the laundry of non-infected household members if possible.

High-Temperature Washing: Wash the items in a standard washing machine using regular laundry detergent and hot water (ideally at or above 60 degrees C / 140 degrees F). Follow this with a complete machine-drying cycle on a high-heat setting.

Immediate Hand Hygiene: Wash your hands thoroughly with soap and water, or use an alcohol-based hand rub, immediately after removing your gloves and completing the laundry process.

record_id Q16_Copilot

question_id Q16

model Copilot

answer_text:

Short answer: Yes - it's possible but uncommon. Mpox virus (MPXV) can survive on smooth surfaces and has been detected on bedding and towels from infected people, so touching contaminated linens could transmit infection if you then touch your face or broken skin; however, most transmission is from direct, prolonged skin-to-skin contact.

Key evidence

Viable virus on surfaces: Laboratory studies show MPXV can remain infectious for days on nonporous surfaces (glass, stainless steel, plastic), and survival is shorter on porous materials. This supports the biological plausibility of fomite transmission.

Environmental detection in real settings: Investigations have found MPXV DNA on many household surfaces and on porous items such as blankets and towels used by infected people; in some studies live virus was not recovered from every PCR-positive sample, but contamination was common.

Public health guidance: Health agencies advise avoiding sharing bedding, towels, clothing, and to launder these items and disinfect surfaces when caring for someone with mpox. They note the overall risk from fomites is lower than from direct skin contact but still real.

How big is the risk

Risk is lower than direct skin-to-skin exposure but not zero. Transmission by contaminated linens requires enough viable virus to be present and a route into your body (e.g., touching contaminated fabric then touching broken skin, mucous membranes, or eyes). Routine household contact with laundered items and basic hygiene makes transmission unlikely.

Practical precautions (what to do)

Avoid sharing towels, bedding, clothing, or utensils with someone who has mpox until they are fully healed.

Handle soiled linens carefully: Wear disposable gloves if available; avoid shaking linens (reduces dispersal of particles); place them in a sealed bag until laundering.

Launder normally: Wash with regular detergent and the warmest appropriate water setting; dry thoroughly. Household detergents and heat inactivate the virus.

Disinfect hard surfaces: Use an EPA-registered disinfectant effective against orthopoxviruses or follow local public-health instructions. Clean high-touch areas regularly.

Hand hygiene: Wash hands with soap and water or use alcohol-based sanitizer after handling linens or being near the patient.

When to seek care or testing

If you develop a new rash, lesions, fever, swollen lymph nodes, or eye symptoms after contact with contaminated items or an infected person, contact your healthcare provider or local public health department promptly for evaluation and testing.

Bottom line: Treat bedding and towels used by someone with mpox as potentially infectious, follow basic infection-control steps (avoid sharing, launder, disinfect, hand hygiene), and the chance of getting infected from touching those items is real but lower than from direct skin contact.

1.Stability of mpox virus on different commonly contacted surfaces

record_id Q16_DeepSeek

question_id Q16

model DeepSeek

answer_text:

Yes, you can get infected by touching bedding or towels used by a patient with mpox, though this is not the primary route of transmission.

While mpox spreads mainly through direct skin-to-skin contact with an infected person's rash, scabs, or body fluids, contact with contaminated objects (known as fomites) is also a recognized route of infection-12-

How the Virus Persists on Surfaces

Mpox is a poxvirus, and studies show this family of viruses can survive on materials for extended periods, particularly in dark, cool, and low-humidity environments

Porous vs. Non-Porous Surfaces: Research indicates that porous surfaces like bedding, clothing, and towels may pose a greater risk for virus exposure compared to non-porous surfaces (e.g., metal, plastic)-1-19. Contaminated bedding and towels can remain a potential source of infection for up to 15 days or longer under favorable conditions-1-24-.

Viral Load: Detected virus levels are generally low, suggesting a limited potential for indirect transmission-19. However, the risk is real, especially if contact with the material occurs before it's been properly disinfected.

Understanding the Risk from Bedding and Towels

The primary concern with contaminated linens is not just casual contact but also specific actions that can release the virus into the air.

Primary Route is Direct Contact: Mpox is most commonly spread through direct physical contact with an infected person-1. Surface transmission is a secondary route, which is why it's considered uncommon compared to direct skin-to-skin contact.

Airborne Resuspension: While not considered airborne in the way COVID-19 is, handling contaminated linens (e.g., making a bed or shaking out a towel) can release the virus into the air. Inhaling these particles could lead to infection, making this a particular risk for caregivers and household members-.

How to Reduce the Risk of Infection

To protect yourself and others, it is crucial to follow strict hygiene and cleaning practices when dealing with items potentially contaminated by a person with mpox.

Avoid Direct Contact: Do not share or directly touch bedding, towels, clothing, or other linens used by a person infected with mpox-27-.

Proper Laundry Procedures:

Containment: Carefully place used or dirty linens into a plastic bag before carrying them to the washing machine. Avoid shaking them, as this can spread virus particles into the air-

Separation: Wash these items separately from other people's laundry. Use a designated container for the contaminated items that can be disinfected afterward-.

Washing: Use hot water and a regular laundry detergent. Washing with soap significantly reduces viral load

Personal Protective Equipment (PPE) for Caregivers: If an uninfected person must handle contaminated linens, they should protect themselves by wearing:

Disposable gloves

A well-fitting mask (e.g., an N95 respirator) to prevent inhalation of any particles

Clothing that fully covers the arms and legs

Disinfection: Clean and disinfect hard surfaces (like floors, counters, and light switches) with an EPA-registered disinfectant known to be effective against emerging viral pathogens-27-.

Summary Recommendation

To summarize, while the risk of fomite transmission is lower than direct contact, it is a genuine concern. Strictly avoid sharing bedding or towels with a person who has mpox. If you must handle these items, follow the proper laundry and PPE guidelines to significantly reduce the potential for infection. Meticulous hand hygiene is also your most important defense; wash your hands thoroughly with soap and water or an alcohol-based hand rub after any potential contact with contaminated surfaces

record_id Q16_Doubao

question_id Q16

model Doubao

answer_text:

Yes, you can get infected with mpox by touching bedding, towels, clothes or other personal items used by an mpox patient.

Key facts

The mpox virus is present in the patient's rash blisters, scabs, and body secretions. It can contaminate bedding, towels, and fabrics.

The virus can survive for days on fabric surfaces.

Infection happens when the virus enters your body through broken skin, or the mucous membranes of your eyes, nose, and mouth after you touch contaminated items.

Risk level

Indirect contact via bedding/towels is less risky than direct close skin-to-skin contact, but it is still a valid transmission route, especially if you share these items or have skin wounds.

Simple prevention

Do not share towels, bedding, tableware or personal items with mpox patients.

Contaminated linens should be washed with hot water, fully dried in sunlight, or disinfected.

Wash your hands thoroughly after contacting any potentially used fabrics, and avoid touching your face.

record_id Q16_Qwen

question_id Q16

model Qwen

answer_text:

Yes, you can get infected with mpox (monkeypox) by touching bedding or towels used by a patient with mpox, though this mode of transmission is less common than direct skin-to-skin contact with infectious lesions. The risk depends on factors like the duration of contact, viral load on the item, and whether your skin has cuts or mucosal exposure. Below is a concise summary of key details:

1. Transmission Risk via Contaminated Items

Mpox virus (MPXV) can survive on porous surfaces like fabrics (bedding, towels, clothing) for days to weeks, especially in cool, dry environments. Studies confirm viable virus on household surfaces for up to 15 days under certain conditions .

Transmission occurs when the virus enters your body through broken skin, mucous membranes (eyes, nose, mouth), or respiratory tract after touching contaminated items .

While less efficient than direct contact with lesions, this route is still a documented risk, particularly if the items are heavily soiled with bodily fluids (e.g., pus from lesions) .

2. Factors Influencing Infection Risk

Key variables:

Viral persistence:

MPXV survives longer on fabrics than non-porous surfaces. For example, it remains viable for up to 30 days at 4 degrees C (e.g., in refrigerated conditions) but degrades faster at room temperature (days to weeks) .

Heat and disinfectants rapidly inactivate the virus: heating to 56 degrees C for 30 minutes or using 75% ethanol, bleach, or hydrogen peroxide eliminates infectivity .

Exposure conditions:

Risk increases with prolonged contact (e.g., sharing a bed) or if you have open wounds/skin lesions .

Casual contact (e.g., brief handling of contaminated items) poses lower risk, but avoid touching your face afterward to prevent self-inoculation .

3. Prevention and Safety Measures

If exposed to contaminated items:

Immediately wash hands with soap and water or use alcohol-based hand sanitizer (>=60% alcohol) .

Do not share bedding, towels, or clothing with someone who has mpox until items are properly disinfected .

Disinfect contaminated fabrics:

Machine-wash in hot water (>=60 degrees C) with detergent, or

Soak in 500-1000 mg/L chlorine solution for 30 minutes before washing .

For high-risk scenarios (e.g., caring for a patient):

Wear gloves and a mask when handling soiled items.

Place contaminated items in sealed bags until laundering to avoid spreading viral particles .

4. Important Context

Most mpox transmission occurs via direct contact with infectious lesions (e.g., during sex or close household contact), not fomites (contaminated objects) .

The Ib variant (spreading globally since 2024) may have enhanced environmental stability, but data on its transmissibility via surfaces remains limited .

No evidence suggests mpox spreads through casual contact with laundered items or brief public surface contact (e.g., doorknobs) .

In summary, touching contaminated bedding or towels can transmit mpox, but the risk is moderate and preventable with proper hygiene and disinfection. If you suspect exposure, monitor for symptoms (fever, rash, swollen lymph nodes) for 21 days and consult a healthcare provider if they develop. Always prioritize avoiding direct contact with active lesions-this remains the highest-risk exposure pathway.

(Mpox),,,- -

Q17. Can a public toilet seat spread mpox?

record_id Q17_ChatGPT

question_id Q17

model ChatGPT

answer_text:

Yes, in theory, but a public toilet seat is not a likely way to get mpox.

Mpox spreads mainly through close, personal skin-to-skin contact with someone who has mpox, especially contact with rash, sores, scabs, body fluids, or intimate contact. CDC says it can also spread from contaminated objects, fabrics, and surfaces that were used by someone with mpox and not disinfected.

For a toilet seat to be a realistic risk, several things would likely need to line up: someone infectious recently contaminated the seat, enough live virus remained, and it contacted broken skin, a cut, or a mucous membrane. WHO notes surface transmission is more concerning when someone touches contaminated items and then touches the eyes, nose, mouth, or other mucous membranes, or has cuts/abrasions.

A CDC household investigation did find mpox virus DNA on a toilet seat, but the culture test was negative, meaning they did not grow live infectious virus from that sample. CDC also notes documented transmission from contaminated objects or materials is rare.

Practical takeaway: using a public toilet normally is very low risk. Wash your hands, avoid touching your face afterward, and use a seat cover or wipe the seat if you're worried. If you had direct contact with someone's mpox rash or contaminated towels/bedding/clothing, that is a much more meaningful exposure.

record_id Q17_Gemini

question_id Q17

model Gemini

answer_text:

The short answer is theoretically possible, but highly unlikely in practice.

While the orthopoxvirus that causes mpox is resilient and can survive on surfaces for days, the risk of catching it from a quick encounter with a public toilet seat is extremely low.

Here is a breakdown of why the risk is minimal and how transmission actually works:

1. The Right Conditions Must Align

For transmission to occur via a toilet seat, a specific chain of events would have to happen almost perfectly:

Active Lesions: An infected individual with active, open mpox sores or fluid-filled blisters on their upper thighs or buttocks would have to sit on the seat and leave fresh, viable fluid behind.

Surface Survival: The virus must survive on the non-porous material (like plastic or porcelain) long enough for the next person to use it.

Portal of Entry: The next person would need to have an open cut, scratch, or broken skin on their own thighs or buttocks that makes direct, prolonged contact with that specific patch of fluid, or they would have to touch the fluid and then immediately touch a mucous membrane (like their eyes, nose, or mouth) without washing their hands.

2. Primary vs. Secondary Transmission

Public health data shows that the vast majority of mpox cases are spread through sustained, intimate, skin-to-skin contact (such as kissing, cuddling, or sexual activity) or direct contact with contaminated materials like bedding and towels used heavily by an infected person over a long period. Fomite (surface) transmission from brief contact in public spaces is incredibly rare.

3. Basic Hygiene Completely Eliminates the Risk

Because the virus is wrapped in a lipid envelope, it is highly susceptible to standard cleaning agents.

Wiping the seat: Standard disinfectant wipes or alcohol-based sanitizers easily destroy the virus on contact.

Hand hygiene: Washing your hands thoroughly with soap and water after using a public restroom eliminates the risk of transferring anything you touched to your face or mouth.

If you are concerned about public restrooms, using a toilet seat cover or wiping the seat down with a disinfectant wipe before sitting provides an extra layer of reassurance.

record_id Q17_Copilot

question_id Q17

model Copilot

answer_text:

Short answer: It's very unlikely that a public toilet seat will spread mpox; transmission mainly requires close skin-to-skin contact with lesions or contaminated materials that directly touch broken skin.

How mpox spreads

Mpox spreads primarily through close, direct contact with a person who has an active rash, scabs, or bodily fluids, and by touching contaminated items (linens, clothing, sex toys) that have not been disinfected. Respiratory spread is possible during prolonged face-to-face contact, but casual contact in public spaces is not a common route. .

Risk from toilet seats and bathroom surfaces

Toilet seats are low-risk because transmission requires viable virus contacting broken skin or mucous membranes; intact skin is an effective barrier. Brief contact with a toilet seat is unlikely to transfer enough virus to cause infection. .

Higher risk scenarios involve direct contact with lesions or contaminated materials (e.g., sharing towels, underwear, or bedding) or using a bathroom immediately after someone with active lesions without cleaning/disinfection. WHO and CDC guidance emphasize contaminated objects and shared living/bathroom spaces as potential risks in congregate settings. .

Practical precautions for public restrooms

Avoid touching your face after contacting surfaces; use a paper towel or tissue to touch handles and the seat if you're concerned. Wash hands thoroughly with soap and water for at least 20 seconds after using a public restroom. .

If you or someone in your household has mpox, use a separate bathroom if possible and disinfect commonly touched surfaces (toilet seat, faucet handles, doorknobs) with an EPA-registered disinfectant or household bleach solution following label instructions.

When to be concerned and seek care

See a healthcare provider if you develop a new unexplained rash, lesions, or flu-like symptoms, or if you believe you had close contact with someone with mpox. Testing and clinical evaluation are the right next steps. Public health authorities can advise on isolation and cleaning. .

Key takeaways

Very low risk from brief contact with toilet seats.

Main risks are direct skin-to-skin contact with lesions and contaminated personal items.

Hand hygiene and surface disinfection are simple, effective precautions. .

If you're worried because of a specific exposure or symptoms, please contact a local healthcare provider or public health clinic for testing and personalized advice.

3. Public health advice on mpox and congregate settings: Settings in which people live, stay or work in proximity

record_id Q17_DeepSeek

question_id Q17

model DeepSeek

answer_text:

The short answer is no, you are not at risk of catching mpox from a public toilet seat under normal circumstances. The virus is almost always spread through direct, close contact with an infected person, not from casual contact with a hard surface in a shared space

The (Very Low) Risk in a Lab Setting

Scientifically, the risk isn't zero, but it is extremely low for the general public. The reason stems from studies looking at the homes of infected people.

Viral DNA vs. Live Virus: A CDC study did find DNA from the mpox virus on a toilet seat in the home of two infected patients-2. However, it's crucial to understand that detecting genetic "fragments" of a virus is not the same as finding a live virus capable of infecting someone. In that same test, no live virus was found on any of the surfaces tested

The Home Environment: The detection of the virus happens in a specific, high-risk environment-the home of someone with a known mpox infection where contamination is heavy and repeated-11. To catch it from a surface, you would typically need to have direct, fresh contact with certain body fluids from an infected person on that surface-6. Routine daily life does not put you in this kind of contact with an infected person's contaminated fluids.

The Bleach Myth & Wastewater Fears

You may have seen headlines suggesting mpox can be spread through toilet water or survive for an implausibly long time on seats. Here's a reality check:

Don't pour bleach in the bowl: You might have seen advice about doing this to prevent infection-this is not a recommended measure. Disinfecting a toilet seat with a standard household cleaner is sufficient if you are concerned about general hygiene, but the CDC does not advise adding bleach to the water for mpox prevention

Wastewater detection != infection risk: The virus has been found in community wastewater. This is a public health surveillance tool (like a community-wide early warning system), not evidence that the water is a source of infection-. There is no evidence that anyone has ever caught mpox from toilet water.

The Final Verdict: How to Stay Safe

While a public toilet seat is not a concern for mpox, everyday hygiene remains important. So, you can use a public restroom without worry.

Your real risk is close contact: Mpox spreads primarily through direct skin-to-skin contact (including sexual contact) with the rash or scabs of someone who has it, or by sharing items like unwashed clothing, bedding, or towels with an infected person-

Wash your hands: This is the simplest and most effective action you can take. Always wash your hands with soap and water or use an alcohol-based hand sanitizer after using the bathroom and before touching your face-. This protects you from mpox and many other common infections.

Know the real symptoms: Being aware of mpox symptoms is more useful than worrying about a surface. The infection usually starts with flu-like symptoms (fever, chills, swollen lymph nodes) followed by a rash that turns into fluid-filled blisters or sores on the skin

The bottom line is that your daily life is not at risk from mpox on a toilet seat. Simple, good hygiene practices are all you need to stay protected.

record_id Q17_Doubao

question_id Q17

model Doubao

answer_text:

Short answer: No, mpox is very rarely, almost never spread through public toilet seats. It is not a common or realistic transmission route.

Key facts

Primary mpox transmission

Mpox spreads mainly through:

Direct close skin-to-skin contact with an infected person's rash, lesions, or body fluids

Contact with contaminated personal items (towels, bedding, clothing)

Prolonged face-to-face respiratory droplet exposure

Why toilet seats are low-risk

The mpox virus cannot easily pass through intact, unbroken skin-most people's skin on the buttocks/thighs has no open wounds.

The virus does not persist well on hard, non-porous surfaces like plastic/ceramic toilet seats in high enough quantities to cause infection in normal use.

Health authorities (WHO, CDC) confirm casual contact with toilet seats, doorknobs, or other public surfaces is not a typical way to catch mpox.

Extremely rare theoretical risk

There is only a tiny hypothetical risk if you have open sores, cuts, or broken skin that directly touches a toilet seat freshly contaminated with live mpox virus. This scenario is highly uncommon in real life.

Simple precautions

Use disposable toilet seat covers or place toilet paper over the seat

Avoid direct seat contact if you have open skin lesions

Practice regular hand washing after using public restrooms

None of these steps are specifically necessary for mpox prevention, but they reduce general germ exposure.

record_id Q17_Qwen

question_id Q17

model Qwen

answer_text:

Monkeypox (mpox) transmission via public toilet seats is theoretically possible but extremely unlikely in real-world settings, with no documented cases of infection occurring this way. The primary transmission route remains direct skin-to-skin contact with infectious lesions or bodily fluids, particularly during prolonged intimate contact.

1. Transmission Risk Assessment

Key factors limiting public toilet seat transmission:

Viral viability requirements: While mpox virus DNA can persist on surfaces for days to weeks under lab conditions (e.g., up to 11 days on stainless steel at room temperature), infectious live virus degrades rapidly in real environments. Studies confirm that surface contamination alone rarely leads to transmission without direct exposure to fresh lesion fluids .

Exposure pathway barrier: Infection requires:

Direct contact between open wounds/mucous membranes and fresh, high-concentration viral material (e.g., active lesion fluid).

Public toilet seats are unlikely to retain sufficient infectious virus from brief contact, as viral load diminishes quickly outside the human body .

No epidemiological evidence: Health authorities globally have not documented a single confirmed case of mpox transmission via toilet seats or similar public surfaces. Most indirect transmission occurs in households with prolonged exposure to contaminated bedding/clothing, not public facilities .

2. Practical Risk Context

Compared to dominant transmission routes:

High-risk exposure: Unprotected sexual contact with an infected person (especially involving active lesions) accounts for >90% of recent global cases. Lesion fluids contain the highest viral concentration, making direct skin contact the primary driver of spread .

Low-risk exposure: Casual contact with surfaces (e.g., toilet seats, doorknobs) poses negligible risk because:

Viral particles on surfaces are typically inactivated by environmental factors (drying, temperature changes).

Intact skin acts as an effective barrier; infection requires direct entry via broken skin/mucous membranes at the contamination site-a highly improbable alignment in public restrooms .

3. Prevention Guidance

For general public: No special precautions are needed for public toilets. Standard hygiene (e.g., handwashing) suffices, as mpox is not spread via casual surface contact.

Higher-risk scenarios: Only individuals with active mpox lesions should avoid sharing personal items (towels, clothing) or surfaces that directly contact broken skin. Household contacts of cases require stricter disinfection of high-touch surfaces .

In summary, while mpox virus can technically survive on surfaces, public toilet seats are not a meaningful transmission route based on current evidence. Focus prevention efforts on avoiding direct contact with active rashes or bodily fluids from infected individuals.

Q18. Can I get mpox from just passing by someone without skin-to-skin contact?

record_id Q18_ChatGPT

question_id Q18

model ChatGPT

answer_text:

Usually, no. Just briefly passing by someone, with no skin-to-skin contact and no close face-to-face interaction, is considered very low risk for mpox.

Mpox spreads mainly through close personal contact, especially direct contact with an mpox rash/scabs, body fluids, saliva/respiratory secretions during close contact, or contaminated items like bedding or towels used by someone with mpox.

It is not spread like COVID through casual air sharing in a hallway. There can be transmission from being close face-to-face, such as talking or breathing close to someone with mpox, but that implies close/prolonged contact, not simply walking past them.

You'd be more concerned if you had:

direct skin contact with their rash or lesions

kissing, sex, cuddling, or prolonged close face-to-face contact

contact with their towels, bedding, clothing, or sex toys

After a known exposure, CDC advises monitoring for symptoms for 21 days, especially a new unexplained rash, fever, chills, swollen lymph nodes, or body aches.

record_id Q18_Gemini

question_id Q18

model Gemini

answer_text:

No. You cannot get mpox from casually passing by someone in public, such as walking past them on the street, browsing the same aisle in a grocery store, or briefly sharing an elevator.

Mpox requires much closer, more sustained contact to travel from person to person.

How Mpox Actually Spreads

Health organizations like the CDC and WHO emphasize that mpox is not easily transmissible in casual, everyday public settings. It primarily spreads through:

Direct, Skin-to-Skin Contact: This is the primary driver of transmission. It happens through close, personal, and often intimate or sexual contact with an infected person's rash, scabs, or body fluids.

Contaminated Materials: Touching items that have recently been used by someone with mpox and haven't been disinfected-such as bedding, clothing, towels, or shared utensils.

Prolonged Face-to-Face Contact: The virus can be carried in respiratory droplets when an infected person breathes, talks, coughs, or sneezes. However, this requires prolonged, close-range exposure (such as living in the same household or intimately interacting for an extended period), not a fleeting interaction.

Why Casual Public Contact is Low-Risk

Mpox does not float through the air or linger across long distances the way highly airborne viruses like COVID-19 or measles do. Because it relies heavily on direct friction with infectious lesions or intensive, prolonged contact, the risk of catching it simply by being in the vicinity of an infected individual without touching them is exceptionally low.

record_id Q18_Copilot

question_id Q18

model Copilot

answer_text:

Very unlikely. Mpox is usually spread by close, intimate, or direct skin-to-skin contact or by touching contaminated items; simply passing someone in a hallway without skin contact is not considered a typical route of transmission.

How mpox spreads

Primary routes: direct skin-to-skin contact with a person's rash or scabs, contact with bodily fluids or mucosal lesions (oral, genital, anal), and contact with contaminated objects (clothing, bedding, sex toys).

Respiratory secretions: virus can be present in saliva and respiratory secretions, but transmission by short, casual airborne exposure (briefly passing someone) has not been shown to be a common route; respiratory spread would generally require prolonged, close face-to-face contact.

Risk from merely passing by someone

Very low to negligible. Casual encounters such as walking past someone, brief conversations at a distance, or being in the same classroom or office are not considered typical ways mpox spreads.

Higher risk situations are hugging, kissing, sexual contact, prolonged close conversation at close range, or sharing contaminated fabrics or utensils.

Rare or uncertain scenarios to be aware of

Fomites: touching recently contaminated clothing, bedding, or objects and then touching your face or broken skin can transmit the virus; this route is considered possible but lower risk than direct skin contact.

Presymptomatic and asymptomatic infections: some studies suggest people can shed virus before symptoms or have undetected infections that may contribute to spread; these findings mean transmission patterns are still being studied and rare, non-sexual or unclear transmission events have been reported. This does not change that casual passing remains low risk, but it underscores why close contacts are prioritized for public-health follow up.

What to do if you're worried

If you had direct contact with someone who has mpox lesions (skin, mucosa) or shared bedding/clothing, monitor for symptoms and contact a healthcare provider or local public-health authority for testing and advice.

Basic precautions: avoid direct skin contact with people who have unexplained rashes, don't share personal items, wash hands and laundry thoroughly, and follow local public-health guidance about vaccination or testing if you had higher-risk exposures.

I'm not a doctor. If you have specific exposure concerns or symptoms (fever, swollen lymph nodes, new rash), please contact a healthcare professional promptly for evaluation and testing.

4.Mpox study reveals that hidden infections may fuel spread

record_id Q18_DeepSeek

question_id Q18

model DeepSeek

answer_text:

This is a very reasonable question, and I can give you a clear answer based on current public health data.

No, you cannot get mpox from simply passing by someone in a store, on the street, or in a similar setting.

Here's a quick breakdown of why, and the one very specific exception you should know about.

How Mpox Actually Spreads

The virus requires relatively prolonged or direct contact. The main routes are:

Direct Skin-to-Skin Contact: Touching the rash, scabs, or body fluids of an infected person. This includes intimate contact.

Contact with Contaminated Objects: Sharing towels, unwashed bedding, clothing, or utensils that have touched the rash.

Respiratory Droplets (the key for your question): This requires prolonged, face-to-face interaction (e.g., living in the same household, caring for a sick person, or being within 3 feet for many hours). A quick "hello" or walking past someone isn't long enough or close enough for the heavy droplets to reach you.

The One Exception: Very Crowded Spaces

The "passing by" risk is virtually zero in normal daily life. However, public health agencies note a theoretical risk in extreme crowding and poor ventilation, such as:

An underground nightclub packed shoulder-to-shoulder for hours, where faces are very close.

A crowded prison cell or a refugee camp tent.

In these specific, high-density environments, you could potentially inhale enough of an infected person's respiratory droplets without direct skin contact. But this is not the same as passing someone at the grocery store or on a sidewalk.

Simple Analogy

Think of mpox as being much closer to a very contagious skin rash than to the airborne flu.

Flu virus travels far through the air and lingers. You can catch it by simply walking through a room where a sick person coughed 20 minutes ago.

Mpox virus is heavy and falls to the ground quickly. You almost always need touch or very close, extended face time.

Bottom Line

Low risk activity (safe): Walking past someone on the street, sitting across from someone on the bus, using the same restroom.

High risk activity: Sharing a bed, sharing towels, prolonged intimate contact, or living in the same small apartment without isolating.

If you are not touching an infected person's rash, their bedding, or having long, close conversations with them (like a nurse with a patient), your risk from "just passing by" is negligible to nonexistent.

If you have more specific concerns about exposure or prevention, the CDC website has excellent, regularly updated resources on mpox.

record_id Q18_Doubao

question_id Q18

model Doubao

answer_text:

No, you almost cannot get mpox (monkeypox) just by casually passing someone by with no skin-to-skin contact and no prolonged close exposure.

Key facts about mpox transmission:

Main spread route: Direct, close skin-to-skin contact with an infected person's rash, blisters, scabs, or bodily fluids.

Not spread easily by brief casual proximity: Walking past someone, passing each other in a crowd, or quick distant conversation does not pose a meaningful risk. It does not spread like colds or COVID through fleeting airborne droplets at a distance.

Rare secondary routes: Prolonged close face-to-face exposure (hours of close contact), or touching contaminated personal items (bedding, clothing, towels) used by an infected person.

In short: Briefly passing by someone with no physical contact is not a way to catch mpox.

record_id Q18_Qwen

question_id Q18

model Qwen

answer_text:

Mpox (formerly known as monkeypox) cannot be transmitted through brief, casual contact like passing by someone without skin-to-skin interaction. The virus requires direct exposure to infectious lesions, prolonged close contact, or contaminated materials to spread. Below is a concise summary followed by key details based on current evidence.

1. Transmission Requires Specific Conditions

Mpox spreads primarily through:

Direct skin-to-skin contact with infectious lesions, scabs, or bodily fluids (e.g., during sexual activity, hugging, or prolonged intimate contact).

Prolonged close respiratory exposure (typically >3 hours in enclosed spaces), not brief encounters like passing someone in a hallway or on public transit.

Contact with contaminated objects (e.g., bedding, clothing) that have touched infectious lesions.

Casual interactions-such as sharing (public transportation), brief conversations, or walking past someone-do not pose a transmission risk.

2. Why Brief Contact Is Not a Risk

Key factors limiting transmission:

Virus entry requires mucous membranes or broken skin: Mpox cannot penetrate intact skin. Transmission needs direct contact with infectious fluids or lesions.

Respiratory droplets require prolonged exposure: Unlike airborne viruses (e.g., SARS-CoV-2), mpox droplets settle quickly and do not linger in the air. Infection via respiratory route typically requires sustained close contact (e.g., >3 hours in a confined space), not fleeting encounters.

Low environmental stability: While the virus can survive on surfaces for weeks, transmission via fomites (objects) is rare and requires direct contact with contaminated items followed by touching mucous membranes.

3. Real-World Context

Most cases involve intimate contact: Over 90% of global mpox cases since 2022 have occurred through close sexual or skin-to-skin contact, primarily among men who have sex with men (MSM).

Household transmission is uncommon: Even among cohabitants, secondary attack rates are low (typically <3%) unless there is direct contact with lesions.

No evidence of casual transmission: Public health agencies (including WHO and CDC) emphasize that daily activities like commuting or dining together pose negligible risk.

4. When to Be Cautious

While passing by someone poses no risk, take precautions if:

You have prolonged skin-to-skin contact (e.g., cuddling, sexual activity) with someone who has unexplained rashes or lesions.

You share bedding, towels, or clothing with an infected person.

You are in prolonged close proximity (e.g., household settings) with someone actively symptomatic.

If you develop symptoms (fever, swollen lymph nodes, or painful rash/lesions), seek medical advice and avoid close contact with others until evaluated.

In summary: Mpox is not spread through brief, non-contact interactions. Transmission requires direct exposure to infectious fluids or lesions, making casual encounters like passing someone in public extremely low risk. Focus on avoiding direct contact with active lesions or prolonged intimate exposure for effective prevention.

Q19. I had contact with someone who has mpox, what should I do first?

record_id Q19_ChatGPT

question_id Q19

model ChatGPT

answer_text:

If you had contact with someone who has mpox (monkeypox), the first steps are about isolation, monitoring, and seeking medical guidance:

Limit contact with others

Avoid close physical contact, especially with people who are immunocompromised, pregnant, or young children.

Do not share bedding, towels, or clothing.

Monitor for symptoms

Symptoms usually appear 5-21 days after exposure, most commonly 7-14 days.

Watch for fever, headache, muscle aches, swollen lymph nodes, chills, exhaustion, and skin lesions or rashes (often starting on the face and spreading).

Seek medical advice promptly

Contact your healthcare provider or local public health authority for assessment and potential testing.

They may recommend post-exposure vaccination (if eligible) or antiviral treatment in certain situations.

Practice good hygiene

Wash hands frequently with soap and water.

Disinfect surfaces you touch regularly.

Stay alert for any symptoms

If you develop a rash or other symptoms, isolate immediately and follow your healthcare provider's instructions.

Follow public health guidance

Your local health department may provide specific recommendations for monitoring and quarantine.

Mpox is contagious through direct contact with lesions, bodily fluids, contaminated materials, or prolonged face-to-face exposure. Early detection and isolation are key to preventing spread.

record_id Q19_Gemini

question_id Q19

model Gemini

answer_text:

Immediate Steps to Take

If you have been exposed to someone with a confirmed or suspected case of mpox, your immediate focus should be on getting evaluated for a preventative vaccine and initiating a health watch.

Here is what you need to do first:

1. Contact a Healthcare Provider or Public Health Authority

Seek Post-Exposure Prophylaxis (PEP): You may be eligible to receive the mpox vaccine, which can prevent the onset of the disease or drastically reduce its severity.

Timing is critical: For the highest chance of fully preventing the illness, the vaccine should ideally be administered within 4 days of your exposure. However, it can still provide significant protection and lessen symptom severity if given up to 14 days post-exposure.

2. Monitor Your Health for 21 Days

Track how you feel daily for exactly 21 days following your last contact with the individual. Watch closely for:

Fever (100.4 degrees F / 38 degrees C or higher) or chills

Swollen lymph nodes (particularly in the neck, armpits, or groin)

Headache, muscle aches, or profound exhaustion

A new, unexplained rash or skin lesions (which can look like pimples, blisters, or sores and may appear on the face, hands, feet, mouth, or genital areas)

3. Implement Prevention Measures

Public health guidelines generally do not require you to isolate or quarantine if you are completely asymptomatic, but you should practice extra caution to protect those around you:

Avoid close physical contact: Pause intimate or direct skin-to-skin contact with others.

Do not share personal items: Avoid sharing unwashed bedding, towels, clothing, utensils, or cups.

Practice strict hand hygiene: Frequently wash your hands with soap and water or use an alcohol-based hand sanitizer.

What to Do If Symptoms Develop

If you develop a fever, rash, or any other early symptoms at any point during your 21-day window:

Isolate immediately in a separate room away from other household members and pets.

Call ahead to the clinic or hospital before arriving so that the staff can take appropriate infection-control precautions to evaluate you safely.

Keep any skin rashes entirely covered with clothing or bandages if you must leave your home for medical care.

Do you know roughly how many days have passed since your exposure, and are you currently experiencing any symptoms?

record_id Q19_Copilot

question_id Q19

model Copilot

answer_text:

If you were exposed to someone with mpox, contact a healthcare provider or your local health department right away, monitor for symptoms for 21 days, and get the mpox vaccine as soon as possible (ideally within 4 days of exposure).

Immediate actions

Contact a healthcare provider or local health department now. They can advise on post-exposure vaccination (PEP) and next steps.

Ask about the JYNNEOS vaccine for post-exposure prophylaxis. Getting vaccinated ideally within 4 days of exposure gives the best chance to prevent illness; vaccination can still help up to 14 days after exposure.

What to monitor and for how long

Watch your health for 21 days after the last exposure for symptoms such as fever, chills, swollen lymph nodes, headache, muscle aches, sore throat, or a new unexplained rash.

If a rash appears: avoid contact with others, cover the rash, wear a mask around people, and seek medical evaluation and testing. Testing is recommended when a rash consistent with mpox develops.

Isolation and infection control if you develop symptoms

Isolate from others until the rash has healed, scabs have fallen off, and a fresh layer of intact skin has formed (this can take 2-4 weeks).

Avoid close physical and sexual contact, do not share bedding, towels, clothing, or utensils, and routinely disinfect commonly touched surfaces with an EPA-registered disinfectant. Wear a well-fitting medical mask when around others.

Who should be especially careful

People who are immunocompromised, pregnant, infants under 1 year, or with eczema/atopic dermatitis are at higher risk for severe disease; notify healthcare providers promptly if you fall into these groups.

Practical next steps for you right now

Call your healthcare provider or local health department to report the exposure and ask about PEP vaccination.

Begin daily symptom checks and perform a thorough skin and oral exam in good lighting for 21 days.

Avoid contact with others and pets if you develop symptoms; cover any rash and seek testing.

Key takeaway: Act quickly to get medical advice and PEP vaccination, monitor for 21 days, and isolate and seek testing immediately if symptoms or a rash develop.

record_id Q19_DeepSeek

question_id Q19

model DeepSeek

answer_text:

If you've had close contact with someone who has Mpox (monkeypox), it is crucial to act quickly. Your first steps should be to assess the nature of your contact, isolate from others, and seek medical advice about a vaccine that can prevent the disease.

Here are the evidence-based steps you should take immediately:

First: Assess Your Risk

The level of risk depends on the type of contact you had:

High-Risk Contact: Direct, skin-to-skin contact with the infected person's rash or lesions, or any sexual or intimate contact. This includes contact with items they've used, like bedding or towels-.

Intermediate/Lower-Risk Contact: Brief or indirect contact, being in the same room for an extended period, or being within 6 feet for over 3 hours.

Step 1: Get Vaccinated (PEP) Immediately

This is your most critical step. A vaccine can be given after (post-exposure prophylaxis, or PEP) to help prevent the disease or make it much less severe-.

For best results, you need to get the vaccine within 4 days of the exposure to prevent the infection entirely-.

It can still be given up to 14 days after exposure to reduce the severity of the illness if infection occurs-.

Where to go: Call your doctor, local health department, or a sexual health clinic immediately. Tell them you've had an mpox exposure and need a PEP vaccine. The vaccine (JYNNEOS) is generally recommended for all unvaccinated close contacts-.

Step 2: Monitor for Symptoms for 21 Days

After an exposure, be vigilant for any signs of mpox for 21 days from your last contact-.

Symptoms to watch for: A new rash (blisters or pimples, on any part of the body, including inside the mouth, anus, or vagina)-, fever, chills, sore throat, headache, muscle aches, backache, and swollen lymph nodes-.

When symptoms start: Symptoms usually begin within a week but can take anywhere from 1 to 21 days to develop-.

What to do: Take your temperature daily-. If you develop a fever or rash, isolate immediately and call your healthcare provider for testing-.

Step 3: Take Precautions to Protect Others

While monitoring for symptoms, you are not considered contagious unless you develop signs. However, to be safe, follow these precautions:

Avoid Close Contact: Do not have any sexual or intimate contact (including skin-to-skin) for the entire 21-day monitoring period-. This is crucial to prevent silent spread.

Practice Good Hygiene: Wash your hands frequently with soap and water or use an alcohol-based hand sanitizer-.

Protect Vulnerable People: Avoid close contact with people who have weakened immune systems, pregnant individuals, and young children for the 21-day period-.

Cover Sores: If you have any lesions or a rash, keep them covered with clothing or a bandage-.

Travel: It is generally recommended to avoid international travel for the entire 21-day monitoring period-.

Step 4: Seek Medical Care if You Become Ill

If you develop any symptoms of mpox (like a rash, fever, or swollen lymph nodes), it becomes a medical issue.

Call ahead: Before going to a clinic or hospital, call ahead and tell them you have a known mpox exposure and are now developing symptoms. This allows them to prepare for your safe arrival and prevent exposing others-.

Isolate at home: While awaiting testing, you should stay in a separate room away from other people and pets, use a separate bathroom if possible, and avoid sharing any items-.

Be careful with facial contact: The virus can also be spread through respiratory secretions during prolonged, face-to-face contact-.

Summary Checklist

To summarize, here's your immediate action plan:

Stop any intimate or close physical contact.

Call your doctor or local health department immediately about getting the PEP vaccine.

Monitor yourself closely for any symptoms (rash, fever, etc.) for 21 days.

Protect others by practicing good hygiene and avoiding high-risk settings.

record_id Q19_Doubao

question_id Q19

model Doubao

answer_text:

First Steps After Mpox (Monkeypox) Exposure

Follow these immediate, priority steps in order:

Isolate yourself right away

Stay at home completely; avoid close physical contact (kissing, hugging, sharing beds) with family, friends, and pets. Do not share towels, bedding, utensils, cups, or personal items with others. Avoid going to work, school, or public gatherings.

Immediately contact local public health or a healthcare provider

Call ahead (do not walk into a clinic unannounced) and clearly state you've had close contact with a confirmed mpox case. They will guide you on post-exposure vaccination (most effective if given within 4 days of exposure, still beneficial up to 14 days), testing, and official quarantine rules.

Start daily health monitoring for 21 days

The mpox incubation period is 5-21 days. Check yourself every day for these early symptoms:

Fever, chills, headache, muscle/back aches

Swollen lymph nodes (a key mpox sign)

Later: Rash, blisters, or pus-filled bumps (often starts on face/hands)

Strict hand & surface hygiene

Wash hands with soap and water frequently, or use 75% alcohol sanitizer. Disinfect high-touch surfaces (phone, doorknobs, tables) daily; avoid touching your eyes, nose, and mouth.

If you develop any symptoms at all during the 21 days, isolate fully immediately and tell your doctor right away for testing and treatment.

record_id Q19_Qwen

question_id Q19

model Qwen

answer_text:

If you've had contact with someone who has mpox (formerly known as monkeypox), your immediate priority is to assess your exposure risk level and contact a healthcare provider or public health authority for guidance-do not wait for symptoms to appear. Most close contacts do not require quarantine but must begin 21 days of active self-monitoring for symptoms like fever, swollen lymph nodes, or rash. Avoid close physical contact with others until cleared by a professional.

1. Immediate First Steps

Assess Your Exposure Risk

Determine whether your contact qualifies as high-risk exposure, which typically includes:

Direct skin-to-skin contact with the infected person's rash, scabs, or bodily fluids (e.g., during sexual activity, caregiving, or sharing towels/bedding) .

Prolonged face-to-face contact (e.g., within 3 feet for >3 hours without masks) in enclosed spaces, potentially exposing you to respiratory droplets .

Contact with contaminated items (e.g., unwashed clothing, bedding, or surfaces touched by the infected person) .

If your contact was brief (e.g., passing in a hallway) or involved no direct contact with lesions or bodily fluids, your risk is likely low. Still, consult a professional for confirmation.

Contact Health Authorities Immediately

Reach out to a healthcare provider or local public health department to report the exposure. Many regions require formal risk assessment by officials to determine next steps .

Do not delay: Early evaluation ensures timely access to vaccines (e.g., JYNNEOS) or antivirals if you're eligible for post-exposure prophylaxis (PEP), ideally within 4 days of exposure for maximum effectiveness .

2. Self-Monitoring and Prevention Measures

Begin 21-Day Symptom Monitoring

Check daily for fever (>37.3 degrees C/99.1 degrees F), swollen lymph nodes, or unusual rashes (especially on hands, face, mouth, or genitals) .

Avoid high-risk activities during this period:

Refrain from sexual contact or skin-to-skin intimacy.

Do not donate blood, organs, or semen.

Wear a mask and cover lesions if you must be near others .

Practice Strict Hygiene

Wash hands frequently with soap and water or use alcohol-based sanitizer.

Disinfect surfaces you've touched using EPA-approved disinfectants (e.g., bleach solutions or 70%+ alcohol) .

Launder contaminated clothing/bedding separately in hot water (>=60 degrees C/140 degrees F) or use bleach .

3. When to Seek Medical Care

Act Immediately If Symptoms Develop

Isolate yourself from others and contact a healthcare provider right away. Mention your mpox exposure history.

Wear a mask and cover all lesions (e.g., with long sleeves/pants) when seeking care to prevent transmission .

Do not self-treat: Antivirals like tecovirimat are reserved for severe cases or high-risk groups (e.g., immunocompromised individuals) and require medical supervision .

Special Cases Requiring Urgent Attention

Eye involvement (pain, redness, vision changes): Seek emergency ophthalmology care to prevent permanent damage .

Severe pain, dehydration, or secondary infections (e.g., pus, fever worsening after rash onset) .

Key Notes

Most mpox cases are mild and resolve in 2-4 weeks with supportive care (e.g., pain relief, hydration) .

Vaccines (JYNNEOS) may be offered post-exposure to high-risk contacts, but availability varies by region .

False reassurance is dangerous: Even if you feel fine, strict 21-day monitoring is non-negotiable, as mpox can incubate for up to 3 weeks .

Consult your national health authority (e.g., CDC, WHO, or local equivalents) for region-specific protocols, as guidelines may differ slightly. Early action significantly reduces transmission risk and severity.

Q20. How long should close contacts of someone with mpox isolate or monitor themselves?

record_id Q20_ChatGPT

question_id Q20

model ChatGPT

answer_text:

According to the U.S. Centers for Disease Control and Prevention (CDC) guidance on mpox (formerly called monkeypox):

Monitoring After Exposure

Close contacts of someone with mpox (mpox exposure) should monitor their health for symptoms for **21 days after the last exposure to the infected person. This is because symptoms of mpox can develop up to three weeks after exposure.

Isolation for Close Contacts

People who were exposed but **do **not have symptoms are not required to isolate or quarantine during this 21-day period. They can continue normal activities like work, school, and daily life as long as they remain symptom-free.

If symptoms do develop (such as rash, fever, swollen lymph nodes, fatigue, or other signs), then the person should immediately isolate and avoid contact with others while seeking medical evaluation and testing.

What to Watch For

Monitor for common mpox symptoms including fever, chills, swollen lymph nodes, headache, muscle aches, exhaustion, and rash that may look like blisters or pimples.

Vaccination and Other Steps

Close contacts should consider post-exposure vaccination (ideally within 4 days of exposure) to prevent or lessen disease severity, and up to 14 days after exposure in some cases.

Summary:

As a close contact without symptoms, you do not need to isolate, but you should monitor for symptoms for 21 days after the last exposure. If symptoms develop during that period, you should immediately isolate and seek medical care.

record_id Q20_Gemini

question_id Q20

model Gemini

answer_text:

If you have been identified as a close contact of someone with mpox, the guidelines for symptom monitoring and isolation depend on whether you are experiencing symptoms.

Symptom Monitoring Period

Duration: You should monitor your health for 21 days after the date of your last exposure to the infected individual.

What to track: Check yourself daily for signs of illness, including:

A new, unexplained skin rash or blister-like lesions (which can appear anywhere on the body, including the face, hands, feet, chest, genitals, or anus)

Fever ($\ge 100.4^\circ\text{F}$ or $38^\circ\text{C}$) or chills

New swollen lymph nodes (in the neck, armpits, or groin)

Headache, muscle aches, or exhaustion

Quarantine vs. Isolation Guidelines

If you DO NOT have symptoms (Asymptomatic):

No strict quarantine required: According to public health standards (such as the CDC), you do not need to isolate or stay home from work or school if you feel perfectly well and have no rash or fever. You can continue routine daily activities.

Precautions to take: During the 21-day monitoring window, you should avoid sexual or close intimate physical contact with others. You should also refrain from donating blood, cells, tissue, breast milk, or semen.

If you DEVELOP symptoms (Symptomatic):

Immediate self-isolation: If you develop a fever, rash, or any other consistent symptoms, you must immediately isolate yourself from others, including household members and pets.

Next steps: Cover any rashes or lesions, put on a well-fitting mask, and contact a healthcare provider or your local public health department right away for guidance on testing and medical evaluation.

Note on Post-Exposure Prophylaxis (PEP): If you know you have been exposed, contact a healthcare provider or public health official as soon as possible. Getting vaccinated within 4 days of exposure offers the best chance of preventing the disease entirely, though vaccination up to 14 days post-exposure can still significantly reduce the severity of symptoms if infection occurs.

record_id Q20_Copilot

question_id Q20

model Copilot

answer_text:

Close contacts should monitor for symptoms for 21 days after their last exposure and seek testing or isolate immediately if any symptoms appear; if symptoms develop, they should isolate until all lesions have healed and a fresh layer of skin has formed (typically 2-4 weeks).

Overview

Incubation period for mpox is usually 7-14 days (range 3-17 days), so public-health guidance uses a 21-day monitoring window after the last known exposure to cover the full risk period.

What close contacts should do

Monitor for 21 days after the last exposure for fever, new rash, swollen lymph nodes, sore throat, cough, or other symptoms. If no symptoms appear, routine activities can continue while observing precautions.

If any symptom appears (even a single lesion), isolate immediately and contact a healthcare provider for testing and care. People with symptoms should remain isolated until all lesions have fully healed and a fresh layer of skin has formed, which typically takes 2-4 weeks.

Additional precautions while monitoring

Avoid close, intimate, or sexual contact with others during the 21-day monitoring period if you develop symptoms or if you cannot reliably avoid skin-to-skin contact with the exposed person.

Do not share bedding, towels, clothing, or utensils with the person who has mpox and launder/disinfect items that may be contaminated. Wash hands frequently.

Post-exposure vaccination and healthcare worker guidance

Post-exposure vaccination (PEP) may be offered to eligible close contacts and is most effective when given within 4 days of exposure to prevent disease; it may still reduce severity if given up to 14 days after exposure. Discuss eligibility with public health or your clinician.

Healthcare settings and congregate facilities follow specific monitoring and work-restriction rules; exposed healthcare workers should follow employer and public-health guidance.

Practical steps if you are a close contact

Mark a 21-day calendar from last exposure and check temperature and skin daily.

Call your clinic or local health department right away if you notice symptoms; ask about testing and PEP.

Key takeaways: Monitor for 21 days, isolate immediately if symptoms appear, and remain isolated until lesions have healed (usually 2-4 weeks).

record_id Q20_DeepSeek

question_id Q20

model DeepSeek

answer_text:

Close contacts of someone with mpox should monitor themselves for symptoms daily for 21 days after their last exposure and are generally not required to isolate unless they develop symptoms.

The World Health Organization (WHO), the US Centers for Disease Control and Prevention (CDC), and the UK Health Security Agency (UKHSA) all agree on this 21-day monitoring period-.

What Monitoring Involves

If you are a close contact, you are expected to do the following for the entire 21-day period:

Daily Self-Checks: Monitor your temperature twice a day and be vigilant for any early symptoms, like fever, chills, or swollen lymph nodes

Continue Daily Activities: As long as you feel healthy and have no symptoms, you can generally go to work, school, and carry on with daily life. Isolating is not required

However, during this monitoring period, you should take these extra precautions to lower any risk of unknowingly spreading the virus:

Avoid High-Risk Contact: Refrain from sexual contact, intimate contact, and sharing items like bedding, towels, or clothing

Protect Vulnerable People: Try to avoid close contact with those at higher risk for severe illness, such as children, pregnant women, older people, and those with weakened immune systems

Don't Donate: Do not donate blood, cells, tissue, breast milk, or semen

Practice Good Hygiene: Wash your hands frequently and practice good respiratory etiquette (covering coughs and sneezes)

Get Vaccinated: If you're eligible, consider post-exposure prophylaxis (PEP) vaccination. It's generally recommended for high-risk contacts and is most effective when given within 4 days of exposure but can be given up to 14 days later-.

Some documents may refer to the 21-day period as "exclusion" from high-risk settings like childcare or healthcare facilities. This means you should avoid such settings unless it is for work or medical care

When to Isolate and Seek Medical Care

If during the 21 days you notice any mpox symptoms (like a new rash, fever, chills, or swollen lymph nodes), you must isolate immediately from other people and pets, and contact a healthcare provider or your local public health unit right away for testing and further advice-

This information is for general guidance. If you're in this situation, you should follow the advice given by your local public health authorities, as specific protocols can vary. Is there anything specific about monitoring or exposure that you'd like more details on?

record_id Q20_Doubao

question_id Q20

model Doubao

answer_text:

Close contacts of someone with mpox should monitor themselves for symptoms for 21 days after their last exposure. Asymptomatic contacts do not need routine isolation or quarantine during this period, but should self-isolate immediately if symptoms develop.

Key Guidelines

Monitoring Period: 21 days post-last exposure (matches the maximum incubation period of mpox, which is 5-21 days)

Monitoring Frequency: At least daily self-assessment for signs and symptoms

Symptoms to Watch For: Fever >=100.4 degrees F (38 degrees C), chills, swollen lymph nodes, new skin rash, headache, muscle aches, fatigue, sore throat Stacks

Asymptomatic Precautions: Avoid close physical contact with others, especially those at high risk of severe disease; practice good hand hygiene and respiratory etiquetteEuropean Centre for Disease Prevention and Control

If Symptoms Develop

No Rash: Follow isolation and prevention practices for 5 days after symptom onset (even if this extends beyond the 21-day monitoring period)

With Rash: Isolate until all lesions have crusted over, scabs have fallen off, and fresh skin has formed underneath (typically 2-4 weeks)European Centre for Disease Prevention and Control

General: Seek medical attention promptly, get tested, and avoid contact with others until cleared by a healthcare providerCenters for Disease Control and Prevention

Exceptions & Additional Notes

Post-exposure prophylaxis (PEP) vaccination may be recommended for high-risk contacts, ideally within 4 days of exposure

Healthcare workers with unprotected exposures should follow workplace-specific monitoring protocols while continuing work if asymptomatic

Specific guidance may vary slightly by country or local health authority, so follow their instructions

record_id Q20_Qwen

question_id Q20

model Qwen

answer_text:

Close contacts of someone with mpox (monkeypox) should undergo 21 days of self-health monitoring, starting from the last day of exposure to the infected person. This is not strict isolation-individuals can continue normal daily activities but must avoid close physical contact (especially sexual contact) and blood donation during this period.

1. Key Details of the 21-Day Monitoring Protocol

Monitoring Requirements

Duration: Exactly 21 days from the last contact with the mpox case, aligning with the virus's maximum incubation period (typically 5-21 days, most commonly 6-13 days) .

Daily self-checks: Monitor for symptoms including:

Fever (>37.3 degrees C / 99.1 degrees F).

Rash or skin lesions (often starting on the face, spreading to hands/feet).

Swollen lymph nodes (a distinguishing feature of mpox compared to similar illnesses) .

No mandatory isolation: Close contacts may continue working and living normally but must avoid high-risk activities like sexual contact, sharing personal items, or donating blood .

Follow-Up and Reporting

Health authority check-ins: Public health officials will contact close contacts at days 7, 14, and 21 for symptom updates and guidance .

Immediate action if symptomatic: Anyone developing symptoms must seek medical care promptly, inform providers of potential mpox exposure, and wear a mask/cover lesions to prevent transmission .

2. Why 21 Days?

The 21-day window covers the full potential incubation period of mpox, as established by global health authorities (including WHO, CDC, and Chinese health agencies) .

Evidence shows transmission risk is negligible beyond 21 days post-exposure. For example, Chinese surveillance data from 2023 indicated that among 374 non-sexual close contacts of cases, only 1 infection occurred (in someone sharing a bed), confirming low secondary spread risk outside intimate contact .

3. Practical Guidance for Close Contacts

Avoid high-risk behaviors: Refrain from sexual activity, sharing towels/bedding, or close skin-to-skin contact until monitoring ends .

Hygiene practices: Wash hands frequently; disinfect surfaces that may have contacted lesions (e.g., using 75% alcohol or bleach solutions) .

Travel considerations: Those entering from affected regions with exposure history must self-monitor for 21 days post-arrival and avoid close contact with others if symptoms arise .

This approach balances public health safety with practicality, as mpox primarily spreads through prolonged skin-to-skin contact (especially sexual) rather than casual interaction. General populations face very low risk, but vigilance during the 21-day window ensures early detection and containment .

Q21. How long after exposure to mpox, if I have no symptoms, can I consider myself safe?

record_id Q21_ChatGPT

question_id Q21

model ChatGPT

answer_text:

After a single exposure, if you have no symptoms for 21 days after your last exposure, you can generally consider mpox infection from that exposure very unlikely. CDC guidance says exposed people should monitor for symptoms for 21 days after their last exposure, and symptoms usually start within that window.

A few practical points:

Count from the last possible exposure, not the first. Ongoing contact resets the 21-day clock.

During those 21 days, watch for a new or unexplained rash/lesions, fever, chills, swollen lymph nodes, exhaustion, muscle aches, headache, sore throat, cough, or rectal/genital pain.

You generally do not need to quarantine if you remain symptom-free; CDC says exposed people can continue routine activities as long as they have no mpox symptoms.

If the exposure was recent and close/high-risk, vaccination soon after exposure may help: ideally within 4 days to prevent disease, and up to 14 days may reduce symptoms.

So: day 22 after your last exposure with no symptoms is the usual "all clear" point, though no timeline is a 100% guarantee. If you develop any rash or concerning symptoms, avoid close/intimate contact and contact a healthcare provider or local health department.

record_id Q21_Gemini

question_id Q21

model Gemini

answer_text:

The 21-Day Monitoring Window

You can generally consider yourself clear 21 days after your last known exposure to mpox, provided you have developed no symptoms.

Public health organizations, including the World Health Organization (WHO) and the CDC, establish this 3-week timeline because the incubation period for the virus ranges from 1 to 21 days. While the average person begins showing symptoms within 7 to 8 days of exposure, the virus can occasionally take the full 21 days to manifest.

What to Watch For During the 21 Days

Even if you feel completely fine right now, it is important to self-monitor daily until the 21-day window completely closes. Keep an eye out for:

The Signature Rash: Lesions that can look like pimples, blisters, or sores. They may appear on the face, inside the mouth, or on other parts of the body such as the hands, feet, chest, or genitals.

Early "Flu-Like" Symptoms: Fever, chills, headache, muscle aches, backaches, and profound fatigue.

Swollen Lymph Nodes: This is a hallmark feature that often helps distinguish mpox from other viral rashes like chickenpox or shingles.

Precautions to Take While Waiting

If you are currently inside this 21-day monitoring window, public health guidelines recommend a few proactive steps:

Consider Post-Exposure Vaccination: If your exposure was very recent, getting vaccinated within 4 days (and up to 14 days) can either prevent the onset of the disease entirely or significantly reduce the severity of the symptoms. Check with a healthcare professional or local public health clinic immediately to see if you qualify.

Practice Temporary Caution: Minimize close skin-to-skin or intimate contact with others, and avoid sharing items like towels, unwashed bedding, or utensils during this period.

Have an Action Plan: If you develop a fever, a new rash, or any other unexplained symptom, isolate yourself from others right away and contact a healthcare provider for guidance on testing.

record_id Q21_Copilot

question_id Q21

model Copilot

answer_text:

Short answer: Monitor for 21 days after your last known exposure; if you remain symptom-free at 21 days you are very unlikely to be infectious. If any new symptom (especially a rash) appears at any time, isolate and seek medical evaluation and testing.

Incubation and infectious window

What experts say: The incubation period for mpox is typically about 3-17 days, but public-health guidance uses a 21-day monitoring window after the last exposure because a small number of cases fall outside the typical range and to allow for conservative public-health practice.

When someone can spread mpox: People are generally considered infectious from when symptoms begin (often a prodrome or rash) until all lesions have healed and new skin has formed. Some evidence shows people can be infectious 1-4 days before symptoms start. There is currently no clear evidence that people who never develop symptoms (true asymptomatic infection) commonly transmit mpox, but uncertainty remains.

What to do after exposure (practical steps)

Monitor your health daily for 21 days after the last exposure; check for fever, swollen lymph nodes, new unexplained rash, mouth lesions, sore throat, or other flu-like symptoms.

Avoid sexual or intimate contact during the 21-day monitoring period and avoid sharing bedding or towels with others.

If a rash or any symptom appears, isolate immediately, contact a healthcare provider, and get tested for mpox as recommended. Continue isolation until a clinician rules out mpox or until lesions have fully healed.

Ask public-health authorities or your clinician about post-exposure vaccination (PEP) if you had a high-risk exposure; PEP is sometimes offered and is most effective when given early.

When you can "consider yourself safe"

If 21 days pass with no symptoms and a thorough skin/mouth exam shows no lesions, public-health guidance considers monitoring complete and the risk of being infectious extremely low.

If you develop any new symptom during or after the 21 days, follow the steps above and contact health services.

Final note

This information is general. Talk with a healthcare provider or your local public-health department for advice tailored to your exposure level and local guidance; they can advise about testing, PEP vaccination, and whether any work or activity restrictions apply.

Signs and Symptoms CDC

Mpox incubation and infectious periods

LAC DPH Mpox Evaluation

record_id Q21_DeepSeek

question_id Q21

model DeepSeek

answer_text:

The Based on current public health guidelines, if you have been exposed to mpox and remain without any symptoms for 21 days, you can consider yourself in the clear and no longer at risk of developing the disease-

Here is the timeline to help you understand the risk period:

The Waiting Period (21 Days): This is the maximum time it can take for symptoms to appear after exposure--21. During these 21 days, you are not contagious--11 and do not need to quarantine--1, but you should monitor yourself closely for symptoms like fever, muscle aches, and especially a new rash

Being Safe (After 21 Days): If you have passed 21 days with no symptoms, public health authorities consider the incubation period over, and you are not going to develop mpox from that specific exposure

If Symptoms Appear

If you develop a fever or a rash during the 21-day monitoring period, it's crucial to isolate yourself immediately and contact a healthcare provider to discuss testing-11-1. You are considered infectious from the moment your first symptoms begin-

I hope this timeline helps provide some clarity. If you have any other questions, please feel free to ask.

record_id Q21_Doubao

question_id Q21

model Doubao

answer_text:

You can generally consider yourself safe 21 days after exposure to mpox (monkeypox) if you have no symptoms CDC. This is the standard maximum monitoring period recommended by global health authorities including the CDC, WHO, E, and Australian CDC CDC.

Incubation Period Details

Exception: A very small proportion of people might develop symptoms after 21 days, but this is extremely rare

Monitoring Guidance

CDC specifically recommends healthcare providers monitor exposed individuals for 21 days after exposure CDC

During this period, watch for: fever, headache, muscle aches, swollen lymph nodes, and rash (which often appears 1-4 days after flu-like symptoms) CDC

If no symptoms develop by day 21, your risk of mpox infection from that exposure is considered negligible CDC

Note: This timeline applies to the vast majority of cases. If you have concerns about a specific exposure, consult a healthcare provider for personalized advice.

record_id Q21_Qwen

question_id Q21

model Qwen

answer_text:

Based on current guidance from the CDC and WHO, here is what you need to know:

Monitoring Period: 21 Days

If you have been exposed to mpox, you should monitor your health for signs or symptoms for 21 days after your last exposure, regardless of whether you have received post-exposure prophylaxis (PEP) or vaccination

Incubation Period Context

The median incubation period is approximately 7 days, but it can range from 1 to 21 days

During the 2022 global outbreak, most cases developed symptoms within 7-10 days of exposure

Rarely, symptoms may appear slightly beyond 21 days, particularly with certain clades (e.g., clade Ib has shown a median of ~18.6 days in some studies)

, which is why the 21-day window remains the conservative public health standard.

When Can You Consider Yourself "Safe"?

If you remain completely asymptomatic through day 21 after your last known exposure:

You can generally consider yourself no longer at risk for developing mpox from that exposure.

You may resume normal activities without mpox-specific restrictions.

Important caveats:

If any symptoms develop during the 21-day period (rash, fever, swollen lymph nodes, sore throat, muscle aches), begin isolation immediately and contact a healthcare provider for evaluation

If symptoms appear without rash, isolate for at least 5 days after symptom onset; if no rash develops and symptoms resolve, isolation may end earlier with clinical guidance.

Even if asymptomatic, avoid donating blood, semen, or tissues during the monitoring period as a precaution

Risk-Based Considerations

Your exposure risk level affects recommendations:

Risk Level Examples Monitoring PEP Recommended

High Skin-to-skin/sexual contact with active lesions; contact with contaminated materials Yes (21 days) Yes

Intermediate Contact with intact skin/clothing of infected person; prolonged close proximity (<6 ft) to symptomatic person Yes (21 days) Individualized decision

Low/None Brief, casual contact; no direct exposure Usually not required No

Practical Tips During Monitoring

Perform daily self-checks: examine skin (including genitals, anus, mouth), monitor temperature, and note any new symptoms.

You do not need to quarantine if asymptomatic-you may continue work, school, and daily activities

Practice good hand hygiene and avoid sharing personal items (towels, bedding) as an added precaution.

Post-Exposure Prophylaxis (PEP)

If you're within 4 days of exposure (up to 14 days if asymptomatic), ask a healthcare provider about the JYNNEOS vaccine for PEP-it can reduce your risk of developing symptomatic infection

Bottom line: If you have had a documented mpox exposure and remain completely symptom-free for 21 full days after your last exposure, you can reasonably consider yourself safe from developing mpox from that exposure. Continue to follow local public health guidance, as recommendations may evolve with new evidence.

Always consult your local health department or healthcare provider for personalized advice based on your specific exposure circumstances.+3

Q22. Is it still useful to get vaccinated after being exposed to someone with mpox?

record_id Q22_ChatGPT

question_id Q22

model ChatGPT

answer_text:

Yes. Mpox vaccination can still be useful after an exposure, especially if you get it quickly.

The CDC says post-exposure mpox vaccine should be given as soon as possible, ideally within 4 days of exposure. If given 4-14 days after exposure, it may still provide some protection, and may reduce illness severity even if it does not fully prevent infection. After 14 days, it becomes more case-by-case, but a clinician may still recommend it in some situations.

A few practical points:

Get vaccinated promptly if you have not already had the mpox vaccine.

The JYNNEOS mpox vaccine is a 2-dose series, with the second dose recommended 28 days after the first.

Monitor for symptoms for 21 days after your last exposure, including fever, swollen lymph nodes, and any new or unexplained rash or lesions.

If symptoms have already started, especially a rash, contact a healthcare provider or local health department about testing and care; vaccination is mainly for prevention/post-exposure, not treatment.

Because timing matters, it is worth contacting a clinic, sexual health clinic, healthcare provider, or local public health department today.

record_id Q22_Gemini

question_id Q22

model Gemini

answer_text:

Yes, But Timing is Everything

If you've been exposed to someone with mpox, getting vaccinated can still be highly effective. Public health agencies like the CDC recommend it as Post-Exposure Prophylaxis (PEP). However, its ultimate effectiveness depends entirely on the clock.

Here is how the timeline breaks down:

Within 4 days of exposure: This is the ideal window. Getting vaccinated within four days offers the best chance of completely preventing the disease from developing.

Between 4 and 14 days of exposure: The vaccine may no longer entirely stop you from getting infected, but it can significantly reduce the severity of your symptoms and keep the illness mild.

After 14 days: The benefits are generally weighed on a case-by-case basis. Your doctor might still recommend it if you are immunocompromised or face an ongoing risk of being exposed again.

Important Caveats

The Symptom Cutoff: Vaccination is not expected to provide a benefit if you have already started developing symptoms (like a rash, fever, chills, or swollen lymph nodes). Once symptoms appear, the vaccine cannot reverse or treat the active virus; instead, the focus shifts to isolation, symptom management, or antiviral treatments like TPOXX (tecovirimat).

If you know or strongly suspect you have been exposed, your best move is to contact a healthcare provider or your local public health department immediately. They can help map out your exact timeline and get you a dose of the vaccine (typically JYNNEOS) as quickly as possible.

record_id Q22_Copilot

question_id Q22

model Copilot

answer_text:

Short answer: Yes - getting the mpox (JYNNEOS) vaccine after a known exposure can still help. If given within about 4 days of exposure, it may prevent infection; if given up to 14 days it may still reduce disease severity, but protection is less certain the later it's given.

Key evidence and what it means

Public-health guidance: Health authorities recommend post-exposure prophylaxis (PEP) with the JYNNEOS vaccine for people with a known high-risk exposure. The vaccine is the preferred option in recent outbreaks. PEP is most effective when given as soon as possible after exposure.

Timing matters: Observational data show many breakthrough infections occurred when vaccination happened several days after exposure, suggesting that if the virus is already incubating vaccination may not prevent infection but can still reduce severity. Giving the vaccine within 4 days offers the best chance to prevent illness; vaccination up to 14 days may still blunt symptoms.

Real-world effectiveness is still being studied. Several cohort and observational studies support ring (post-exposure) vaccination reducing cases, but measuring exact effectiveness is challenging because exposures, timing, and behaviors vary. This means recommendations are based on the best available evidence and expert judgment.

Practical steps to take now

Get vaccinated as soon as possible - contact your local health department, sexual health clinic, or primary care provider and tell them you had a recent exposure. Ask specifically for JYNNEOS if available.

Watch for symptoms for 21 days after exposure (fever, new rash, swollen lymph nodes, sore throat, etc.) and seek testing/treatment immediately if symptoms appear.

Avoid close contact (sexual or skin-to-skin) with others until the incubation window has passed or you are cleared by public-health guidance.

Risks, limitations, and what to expect

Vaccine may not prevent infection if given late in the incubation period; some vaccinated people still developed mpox, often when vaccinated several days after exposure.

Side effects from JYNNEOS are generally mild (injection-site reactions, fatigue); ACAM2000 is another vaccine but has more side effects and is used less commonly.

Evidence gaps remain. Ongoing studies continue to refine how well PEP prevents infection and how timing, dose, and route affect outcomes.

Bottom line: If you were exposed, seek vaccination immediately - the sooner the better. Even if it's been several days, vaccination can still be worthwhile to reduce the chance of severe disease; follow local public-health advice and monitor for symptoms.

record_id Q22_DeepSeek

question_id Q22

model DeepSeek

answer_text:

Yes, getting vaccinated after exposure to someone with mpox can still be useful. The vaccine (typically JYNNEOS) is recommended for post-exposure prophylaxis (PEP). For the best chance of preventing the disease, the vaccine should ideally be given within 4 days of exposure. If given between 4 and 14 days after exposure, it may not prevent the disease but can still reduce the severity of symptoms. If you've been exposed, it's important to contact a healthcare provider as soon as possible to discuss vaccination and other preventive measures.

record_id Q22_Doubao

question_id Q22

model Doubao

answer_text:

Yes, getting vaccinated after exposure to mpox is still useful, but its effectiveness depends on how soon after exposure you receive it and whether you develop symptoms

Key Timing & Effectiveness

Ideal: Within 4 days of exposure

Offers the highest chance of preventing infection entirely.

Still beneficial: 4-14 days after exposure

May not prevent infection, but strongly reduces symptom severity, risk of hospitalization, and complications.

After 14 days (asymptomatic only):

Benefits are limited but may still be considered case-by-case for high-risk individuals (e.g., severely immunosuppressed).

Once symptoms appear:

Vaccination is no longer recommended for post-exposure prophylaxis (PEP).

Why It Works

Mpox vaccines (e.g., JYNNEOS/MVA-BN) can:

Trigger a rapid immune response even after exposure

Reduce viral load and disease progression

Provide cross-protection if you were previously unvaccinated

Important Notes

Post-exposure vaccination is less effective than pre-exposure vaccination but still a critical tool.

Complete the full 2-dose series (4 weeks apart) for optimal protection, even if you received the first dose as PEP.

Monitor symptoms for 21 days after exposure, regardless of vaccination status.

If you're already fully vaccinated (2 doses) or have recovered from mpox, additional vaccination after exposure is not recommended.

Next Steps

If you've had a high-risk exposure to someone with mpox:

Contact your healthcare provider immediately.

Get vaccinated as soon as possible (ideally within 4 days).

Avoid close/intimate contact with others for 21 days.

record_id Q22_Qwen

question_id Q22

model Qwen

answer_text:

Yes, getting vaccinated after exposure to mpox (monkeypox) is still useful and recommended as part of post-exposure prophylaxis (PEP), but timing is critical. Vaccination within 4 days of exposure offers the best chance to prevent infection, while administration within 14 days may still reduce symptom severity even if infection occurs. However, protection is not guaranteed, and effectiveness is lower compared to pre-exposure vaccination. Below is a detailed analysis based on current guidelines and evidence.

1. Key Timing Windows for Post-Exposure Vaccination

Critical timeframes:

Optimal window: Vaccination within 4 days of exposure significantly reduces the risk of developing symptomatic mpox.

Extended window: Vaccination up to 14 days after exposure may still lessen disease severity (e.g., milder symptoms, fewer lesions, shorter illness duration), though it may not prevent infection entirely.

Beyond 14 days: Vaccination is not recommended once symptoms appear, as it offers no therapeutic benefit at that stage.

2. Effectiveness of Post-Exposure Vaccination

Protection levels vary by context:

General population:

Single-dose efficacy for PEP is estimated at ~76-84% for preventing symptomatic infection if given promptly, but two doses (28 days apart) provide stronger and more durable protection.

Even if infection occurs, vaccinated individuals typically experience milder symptoms, fewer skin lesions, and lower transmission risk.

Immunocompromised individuals:

People with HIV (especially untreated), autoimmune conditions, or on immunosuppressive therapy may have reduced vaccine response. For example, HIV-positive individuals show slower antibody development and faster waning of immunity after vaccination.

Two doses are strongly advised for high-risk groups, and antiviral treatment (e.g., tecovirimat) may be added for severe cases.

3. Limitations and Important Considerations

Factors affecting PEP success:

Vaccine type matters:

MVA-BN (JYNNEOS) is the preferred vaccine for PEP due to its safety profile (non-replicating) and suitability for immunocompromised individuals.

ACAM2000 (replicating vaccine) is not recommended for PEP in people with eczema, HIV, or pregnancy due to higher risks of adverse events.

Immunity wanes over time:

Antibody levels decline significantly after ~12-18 months, particularly in those without prior smallpox vaccination or natural mpox exposure. This may reduce long-term PEP efficacy.

Not 100% protective:

No vaccine guarantees complete prevention. Continued precautions (e.g., avoiding close contact, using condoms) are essential even after vaccination.

4. Practical Recommendations

If exposed to mpox:

Seek vaccination immediately: Contact public health authorities or a healthcare provider within 4 days for PEP.

Complete the full series: Receive two doses of MVA-BN (28 days apart) for optimal protection, especially if immunocompromised.

Monitor symptoms: Self-isolate and monitor for 21 days (fever, rash, swollen lymph nodes). Report symptoms promptly.

Combine with other measures: Use PPE during caregiving, avoid sharing personal items, and practice strict hand hygiene.

In summary, post-exposure vaccination is valuable for reducing mpox risk or severity, but prompt action within 4-14 days of exposure is essential. While not foolproof, it remains a cornerstone of public health strategy alongside surveillance and behavioral precautions. Consult local health authorities for region-specific protocols, as guidelines may evolve with new variants (e.g., clade Ib).

Q23. Which hospital department should I visit if I suspect I have mpox?

record_id Q23_ChatGPT

question_id Q23

model ChatGPT

answer_text:

For suspected mpox, the best "department" is usually Infectious Diseases or a Sexual Health/STI clinic if your hospital has one. If you can't access those, go through urgent care/primary care first. Use the Emergency Department only if symptoms are severe or you cannot get timely care elsewhere.

Call before you go and say you may have mpox. The hospital may route you to a specific entrance or room so you do not wait in a general area. CDC says to see a healthcare provider promptly if you have a new or unexplained rash, especially after possible exposure, and to cover the rash and wear a mask around others, including healthcare providers.

Go to the ER now or call emergency services if you have trouble breathing, chest pain, confusion, seizures, severe/unmanageable pain, eye pain/vision changes, or lesions near the eye. Eye involvement needs urgent ophthalmology evaluation.

Until you're evaluated, avoid close contact, cover any rash, wear a well-fitting mask, and avoid sharing towels, bedding, clothing, or sex/skin-to-skin contact. Mpox can spread from symptom onset until the rash has fully healed and new skin has formed.

record_id Q23_Gemini

question_id Q23

model Gemini

answer_text:

If you suspect you have mpox, the most appropriate hospital department depends on the facility's structure and your current symptoms. However, the most critical first step is to call the hospital or clinic before you arrive so they can prepare to isolate you immediately and prevent exposing other patients.

When you contact or arrive at the hospital, you will generally be directed to one of the following departments:

1. Infectious Diseases Department

This is the primary department equipped to handle viral outbreaks and contagious diseases. They specialize in testing, diagnosing, and managing infections like mpox, and they possess the necessary isolation infrastructure.

2. Dermatology Department

Because the most defining symptom of mpox is a distinct rash (which can look like pimples, blisters, or sores), many patients initially present to dermatology. Dermatologists are trained to differentiate mpox lesions from other conditions like shingles, syphilis, or herpes and can collect the necessary fluid swabs for diagnostic testing.

3. Fever Clinic or Emergency Department

Fever Clinic: If you are experiencing systemic symptoms like a high fever, chills, swollen lymph nodes, or body aches alongside a rash, the fever clinic is often the designated entry point for screening communicable diseases.

Emergency Department: If you are experiencing severe pain (particularly rectal or oral pain), difficulty breathing, or if it is outside of regular outpatient clinic hours, proceed to the emergency room.

Important Precautions for Your Visit

Wear a Mask: Cover your nose and mouth with a well-fitting surgical or medical mask.

Cover All Lesions: Wear long sleeves, pants, or bandages to fully cover any visible rash or sores to prevent skin-to-skin or surface contamination.

Inform Staff Immediately: As soon as you arrive, notify the triage nurse or front desk that you suspect you have mpox so you can be placed in a private room right away.

record_id Q23_Copilot

question_id Q23

model Copilot

answer_text:

If you suspect mpox, first contact your regular primary care provider or a local sexual health/STD clinic; if you have severe symptoms (high fever, severe pain, dehydration, trouble breathing, or inability to urinate/defecate) go to the Emergency Department right away.

Quick answer and next steps

Where to go first: Primary care, urgent care, or a dedicated sexual health/STD clinic are appropriate first stops for evaluation and testing for mpox when symptoms are mild to moderate (rash, fever, lymphadenopathy).

When to go to the Emergency Department: Severe or complicated illness (widespread painful lesions, severe proctitis, dehydration, signs of bacterial superinfection, or if you are immunocompromised) requires ED evaluation.

Call ahead: Phone your clinic or ED before arrival to let them know you suspect mpox so they can prepare infection-control measures and direct you to the right entrance or clinic.

What each department can do

Primary care / family medicine: Triage symptoms, perform a skin/mucosal exam, collect lesion swabs for orthopoxvirus testing, manage symptoms, and refer for treatment or public-health reporting if needed.

Sexual health / STD clinic: Often experienced with genital/anorectal lesions, can test for mpox and other STIs concurrently, and provide counseling on isolation and partner notification.

Urgent care: Appropriate for non-severe symptoms when primary care is unavailable; confirm they can collect lesion swabs and will notify public health if positive.

Emergency Department: For severe pain, systemic illness, dehydration, inability to eat/drink, severe proctitis, or suspected complications; EDs can admit, provide IV fluids, pain control, and specialist consults.

Practical precautions and what to tell staff

Tell them you suspect mpox when you call or arrive so staff use appropriate infection-control precautions.

Cover lesions, avoid close contact, and avoid sexual activity until evaluated and cleared.

Bring a list of symptoms and recent exposures or travel (especially travel to areas with known mpox cases) and any immune-suppressing conditions.

Final note

Seek medical evaluation promptly-a clinician will decide whether testing (lesion swab for orthopoxvirus PCR) and treatment are needed and will coordinate with public health if required. If you have severe symptoms or are at high risk for complications, go to the Emergency Department.

Mpox(Monkeypox) Clinical Pathway - Emergency Department, Outpatient Specialty Care, and Primary Care Children's Hospital of Philadelphia

Mpox Guidance-CDPH

LAC DPH Mpox Provider Hub

record_id Q23_DeepSeek

question_id Q23

model DeepSeek

answer_text:

If you suspect you have mpox, the most appropriate hospital departments to visit are typically Dermatology or Infectious Disease. If you are experiencing severe symptoms, go directly to the Emergency Department.

Recommended Departments

Department When to Choose

Dermatology Mainly for skin-related issues like rashes, blisters, or pustules-

Infectious Disease Best if you've had close contact with a confirmed case or for a comprehensive diagnosis-.

Emergency Department Go here if you have a high fever, difficulty breathing, or severe symptoms-

Important Tip: Remember to tell your doctor about your contact and travel history-.

To find the nearest hospital, you can search online terms like "yourcityname mpox designated hospital" or "yourcityname infectious disease hospital."

Disclaimer: I am an AI, not a doctor. This information is for reference only and is not a substitute for professional medical advice. If you are unwell, please consult a medical professional as soon as possible.

record_id Q23_Doubao

question_id Q23

model Doubao

answer_text:

If you suspect you have mpox, you can visit these hospital departments:

Infectious Diseases Department (the first choice, since mpox is a viral infectious disease)

Dermatology Department (for diagnosis of mpox's typical skin rashes, blisters and lesions)

Fever Clinic

It is recommended to take full personal protection (wear a medical mask), avoid close contact with others, and truthfully tell the doctor your travel and exposure history. You can also go directly to a designated infectious disease hospital for consultation.

record_id Q23_Qwen

question_id Q23

model Qwen

answer_text:

If you suspect you have mpox (monkeypox), you should visit a dermatology department, fever clinic, or infectious diseases department at a general hospital. These departments are best equipped to assess symptoms like rash, fever, and lymph node swelling while following proper infection control protocols. Below are key details to guide your visit:

1. Recommended Hospital Departments

Dermatology department: Primary choice for evaluating characteristic mpox rashes, which often appear on the face, limbs, or mucosal areas (e.g., mouth, genitals, anus) .

Fever clinic: Designed to screen and isolate patients with infectious symptoms like fever and rash, minimizing transmission risk .

Infectious diseases department: Critical if systemic symptoms (e.g., high fever, severe pain) or immunocompromised status is present, as they specialize in viral infections .

2. What to Do Before and During Your Visit

Disclose critical information:

Mention recent travel to mpox-endemic regions (e.g., Central/West Africa or areas with current outbreaks).

Report close contact with confirmed/suspected mpox cases or exposure to high-risk settings (e.g., sexual contact with multiple partners).

Note symptom onset timing, as mpox has a 5-21-day incubation period (typically 6-13 days) .

Wear a mask and cover lesions: Reduce transmission risk by wearing a surgical mask and covering visible rashes with clothing or bandages .

Avoid public transport: Use private transport if possible, and inform the hospital in advance to prepare isolation protocols .

3. What to Expect at the Hospital

Immediate isolation: Suspected cases are typically directed to a separate isolation area to prevent community spread .

Diagnostic testing: Swab samples from skin/mucosal lesions are tested via PCR for mpox virus; blood tests are less reliable .

No routine antiviral treatment: Most cases are self-limiting (resolving in 2-4 weeks), so care focuses on symptom relief (e.g., pain management, infection prevention for lesions) .

4. When to Seek Urgent Care

Visit an emergency department if you experience:

Severe complications: Difficulty swallowing, vision changes (from eye involvement), or secondary bacterial infections (e.g., pus-filled sores).

High-risk factors: Being immunocompromised, pregnant, or under 8 years old, as these groups face higher risks of severe disease .

Note: Mpox is rarely fatal in healthy individuals, but early medical evaluation ensures proper diagnosis (ruling out similar conditions like chickenpox or herpes) and reduces transmission risk. Always inform healthcare providers about potential exposure history to expedite testing. For real-time guidance, check local health authority updates, as recommendations may evolve with outbreaks.

Q24. Do I need a PCR test for mpox?

record_id Q24_ChatGPT

question_id Q24

model ChatGPT

answer_text:

You usually do not need an mpox PCR test unless you have a rash or lesions that look like mpox. CDC guidance says testing is currently recommended for people with a suspicious rash, and a healthcare provider can help decide if testing is needed after symptoms or close contact.

When mpox testing is done, it is typically a real-time PCR test from swabs of skin lesions, not a routine blood, nasal, or home test. Clinicians usually swab material from lesions, often from more than one lesion. WHO notes that blood PCR is not recommended for diagnosis because it can miss cases.

You should contact a healthcare provider or sexual health clinic soon if you have new painful or unusual bumps/blisters/scabs, especially in the genital, anal, mouth, face, hands, or body areas, or if you had close skin-to-skin or sexual contact with someone diagnosed with mpox. While waiting, avoid close physical/sexual contact and don't share towels, bedding, or clothing.

If you only had a possible exposure but no rash or lesions, testing usually is not done right away; instead, monitor for symptoms and ask a clinician or local health department whether vaccination or other precautions are appropriate.

record_id Q24_Gemini

question_id Q24

model Gemini

answer_text:

Yes, if an mpox infection is suspected, a real-time polymerase chain reaction (PCR) test is the definitive, gold-standard method required for confirmation.

Whether you or someone you are evaluating needs a test depends on clinical presentation and exposure history.

When a PCR Test is Needed

Testing is indicated for individuals who present with:

An unexplained acute rash or distinct skin lesions (which may progress from macules to papules, vesicles, pustules, and eventually crusts).

Systemic prodromal symptoms-such as fever, prominent lymphadenopathy (swollen lymph nodes), profound fatigue, headache, or myalgia-accompanied or followed by a rash.

An epidemiological link, such as close, intimate, or direct physical contact with a confirmed or suspected mpox case within the past 21 days, or travel to a region with active clade-specific outbreaks.

Why PCR is the Standard

High Sensitivity and Specificity: PCR accurately detects the presence of monkeypox virus (MPXV) DNA.

Clade Differentiation: Current public health and commercial protocols utilize multiplex PCR assays to differentiate between Clade II (the strain responsible for the 2022 global outbreak) and Clade I (including subclade Ib), which is critical for clinical management and public health reporting.

Serology Limitations: Antibody testing is rarely used for acute diagnosis because cross-reactivity with other orthopoxviruses (or prior smallpox/mpox vaccinations) makes interpretation difficult.

Specimen Collection Requirements

If a test is necessary, the sample collection protocol is highly specific:

Lesion Swabs Only: The specimen must be collected by vigorously swabbing the surface, exudate, or crust of active skin lesions using sterile, synthetic swabs (e.g., Dacron or polyester).

Avoid Blood/Serum for Diagnosis: Blood samples are not suitable for primary diagnostic PCR. The viremic phase of the virus is transient and typically resolves by the time the hallmark rash appears, meaning a blood PCR can easily return a false negative.

Are you asking because you or someone you are evaluating is currently experiencing a characteristic rash or has had a known exposure?

record_id Q24_Copilot

question_id Q24

model Copilot

answer_text:

Short answer: You only need a PCR test for mpox (monkeypox) if you have a new, unexplained rash that looks like mpox and a clinician agrees testing is appropriate. If you have such a rash, a healthcare provider will swab lesions and order a real-time PCR test.

When testing is recommended

Key rule: Test only if you have a rash consistent with mpox (new vesicles, pustules, or ulcers) or a clinician suspects mpox after evaluating symptoms and exposure.

Typical triggers for testing

New unexplained rash, especially with fever, swollen lymph nodes, or recent close contact with a person who has mpox.

Clinical judgment by a healthcare provider who can order the test.

What the test is and how it's done

Test type: Real-time PCR is the standard diagnostic test for mpox.

Specimen: Clinicians collect swabs from lesions (usually 2 swabs from each lesion, from 2-3 lesions) to ensure enough material for testing and possible clade-specific follow-up.

Turnaround: Results are usually available in 2-4 days, though timing varies by lab.

Where to get tested and cost

Only a healthcare provider can order the test. They may collect the specimen in clinic or refer you to a lab. Public health departments often offer testing and may do so at low or no cost; commercial labs may charge. Contact your local health department or clinician for options.

What to do while waiting for results

Isolate from close contacts and avoid intimate or household contact until you know the result or a clinician advises otherwise. Follow infection-control guidance from public health authorities.

If your test is positive or negative

Positive: Follow clinical guidance for isolation, care, and notifying close contacts; public health reporting will occur.

Negative: A negative result usually means mpox was not detected, but continue precautions if symptoms persist or if your clinician recommends repeat testing.

Important considerations and limitations

Testing is not for screening asymptomatic people; it's for symptomatic individuals with a suspicious rash.

Specimen quality matters: inadequate swabbing can lead to inconclusive results and repeat testing.

record_id Q24_DeepSeek

question_id Q24

model DeepSeek

answer_text:

Whether or not you need a PCR test for mpox depends entirely on your situation. PCR is the reference standard for diagnosis, but it's not typically required for general travel. Here's how to know if it might be necessary for you.

Why Get a PCR Test for Mpox?

A PCR test is a laboratory method used to detect the genetic material of the mpox virus. Health experts strongly recommend it for confirming an infection in anyone with symptoms or a known exposure

If you have symptoms: The main symptom is an unexplained rash that can look like pimples or blisters, sometimes accompanied by fever, swollen lymph nodes, headache, or muscle aches-15-2. If you notice such a rash, you should see a healthcare provider for evaluation.

If you have had contact with a confirmed case: Testing might be recommended even before a rash appears, depending on your symptoms.

It's the "Gold Standard": Health authorities like the WHO consider PCR the most accurate method for mpox diagnosis, capable of confirming an infection with a single positive result.

Should You Get Tested for Travel?

Generally, no. There are no widespread global requirements for a pre-travel mpox PCR test. Countries do not usually ask for a negative test certificate for entry.

However, be aware of the following:

Post-travel with symptoms: If you develop symptoms within 21 days of returning from a trip, a PCR test may be needed to confirm a suspected case-15. It's important to provide your healthcare provider with a complete travel history so they can best assess your risk.

Airport Health Screening: Some airports may conduct health screenings, but research suggests PCR tests at these points are not foolproof and miss a significant number of infected travelers.-

A Note on Testing Availability

Please check your local situation for the most current information.

Global Guidance: The WHO recommends testing for all suspected cases and has approved several PCR tests for emergency use to improve global access.-

The United States (as of May 2026): It's important to know that the CDC has paused its own diagnostic testing for mpox due to an internal review.-49 Mpox testing is still available, but you may need to seek it from state, local, or commercial laboratories and not the federal CDC lab directly.

To Summarize:

Do you need a pre-travel PCR test? No. Usually not.

Do you need a PCR test for diagnosis? Yes. If you have symptoms compatible with mpox or a known exposure, see a healthcare provider. They can assess your risk and determine if testing is necessary.

record_id Q24_Doubao

question_id Q24

model Doubao

answer_text:

Yes, a PCR test is required for confirming mpox (monkeypox) infection - it is the reference standard recommended by WHO, CDC, and other global health authorities.

When You Need a PCR Test for Mpox

You should get a PCR test if you:

Have mpox-like symptoms (fever, headache, muscle aches, swollen lymph nodes, followed by a rash with lesions)

Are a suspected or probable case based on clinical and epidemiological criteria (e.g., close contact with a confirmed case)IRIS

Had close contact with someone diagnosed with mpox and develop symptoms

Why PCR is the Preferred Test

Only recommended method for routine diagnosis (serology and viral culture are not recommended for routine use)

Highly accurate: A single positive lesion-specific PCR confirms mpox

Detects viral DNA directly: Most reliable during active infection, especially from skin lesions

Required for confirmation: Clinical diagnosis alone is not sufficient without laboratory confirmation

What If You Don't Have Symptoms?

PCR testing is generally not recommended for asymptomatic individuals unless specifically advised by public health authorities for surveillance purposes.

Testing Details

Best specimens: Skin lesion swabs (fluid, crusts, or surface) - vigorous swabbing is recommended

Alternative specimens: Throat or anal swabs if no skin lesions are present

Blood tests are not recommended for diagnosis

Negative results may need repeat testing if clinical suspicion remains high

How to Get Tested

Contact your healthcare provider or local public health department if you have symptoms or concerns about mpox exposure. They will arrange for PCR testing through appropriate laboratories.

record_id Q24_Qwen

question_id Q24

model Qwen

answer_text:

You only need a PCR test for mpox if you have symptoms suggestive of mpox and meet specific epidemiological risk criteria. PCR testing is not recommended for asymptomatic individuals or routine screening but is the gold standard for confirming suspected cases when clinical or exposure risks are present. Below is a detailed breakdown:

1. When Testing Is Recommended

Key indications for mpox PCR testing include:

Presence of characteristic symptoms:

Unexplained skin or mucosal lesions (e.g., rash, blisters, pustules, or scabs), often accompanied by fever, swollen lymph nodes, or pain in the affected areas. Lesions may appear on the face, hands, genitals, or anus.

Atypical presentations, such as lesions limited to genital/anal regions without systemic symptoms (common in recent outbreaks).

Epidemiological risk factors:

Close contact with a confirmed mpox case within the past 21 days.

Being part of a high-risk group, such as men who have sex with men (MSM), sex workers, or individuals with multiple recent sexual partners.

Travel to or residence in regions with active mpox transmission (e.g., parts of Africa where mpox is endemic).

For children/adolescents: Unexplained rash with fever in areas where mpox is circulating.

Clinical suspicion despite negative initial tests:

If symptoms persist but initial tests (e.g., for sexually transmitted infections) are negative, mpox should still be ruled out, as co-infections are common (e.g., 25-50% of mpox cases may overlap with STIs like chlamydia or gonorrhea).

2. When Testing Is Not Needed

Asymptomatic individuals: Routine screening is not recommended, even for close contacts. Instead, monitor for symptoms for 21 days.

Blood testing for diagnosis: PCR on blood samples is unreliable because mpox viremia is brief and occurs early in infection. False negatives are common.

Self-testing: No validated at-home mpox tests exist. Suspected cases must be evaluated clinically.

3. How the Test Is Performed

Preferred sample type:

Skin/mucosal lesion material (e.g., fluid from blisters, scabs, or vigorous swabs of lesion surfaces). This is the most reliable method, with near-100% sensitivity when lesions are present.

Alternative samples (if no lesions):

Oropharyngeal, anal, or rectal swabs may be used but have lower sensitivity. A negative result does not rule out mpox.

Procedure:

A healthcare provider collects samples using sterile swabs, avoiding disinfectants on lesions beforehand.

Testing is conducted in specialized labs via real-time PCR, which detects mpox viral DNA with high accuracy (sensitivity/specificity >95%).

4. Important Considerations

Do not delay care: If you have a new, unexplained rash with risk factors, seek medical evaluation immediately. Early diagnosis reduces transmission risk and may guide treatment (e.g., antivirals like tecovirimat for severe cases).

Co-testing is critical: Due to high rates of co-infection with STIs, testing for HIV, syphilis, gonorrhea, and chlamydia is strongly advised alongside mpox testing.

No self-diagnosis: Symptoms of mpox overlap with herpes, syphilis, and other conditions. Only PCR or viral culture can confirm mpox.

If you lack symptoms or risk factors, testing is unnecessary. For personalized advice, consult a healthcare provider who can assess your specific situation.

Q25. What type of sample is usually collected for mpox testing?

record_id Q25_ChatGPT

question_id Q25

model ChatGPT

answer_text:

Mpox testing usually uses skin lesion material, most often collected by swabbing the surface of a rash/lesion, lesion fluid/exudate, or crusts/scabs. CDC says clinicians generally collect two swabs from each lesion, often from 2-3 lesions, for PCR testing.

A blood sample is not usually the main diagnostic sample for mpox. Collection details can vary by lab, so a clinician or public health lab should confirm the exact swab type and transport requirements.

record_id Q25_Gemini

question_id Q25

model Gemini

answer_text:

The primary and most effective sample collected for mpox testing is skin lesion material.

Because the virus replicates heavily in the skin manifestations, taking a sample directly from the rash provides the highest accuracy for diagnostic testing.

Key Details of Specimen Collection

What is sampled: Healthcare providers will target active skin lesions. This includes swabbing the lesion surface, the fluid/exudate inside a pustule or vesicle, or collecting crusts and scabs from healing lesions.

The Swab Type: Samples are collected using sterile, synthetic swabs (such as polyester, nylon, or Dacron) with plastic or aluminum shafts. Cotton swabs or those with wooden shafts are avoided because they can interfere with the molecular test results.

The Process: A provider will vigorously rub the swab across the lesion to ensure they gather enough viral DNA. Testing guidelines explicitly state not to lance or "unroof" (pop) the blisters beforehand, as this is unnecessary and increases the risk of accidental needle or sharp stick injuries for medical staff.

Quantity: Typically, providers will collect two swabs from each lesion and may sample two or more different lesions on the body to ensure an adequate sample size.

Why Blood Tests Aren't Used

While you might think a blood draw would work, blood samples are not recommended for routine mpox diagnosis. The virus is only present in the bloodstream for a very short window early in the infection, meaning a blood test could easily return a false negative even if a person is actively sick.

Alternative Samples

If a patient has symptoms but doesn't have a visible skin rash, providers may look to alternative sites based on clinical presentation. These can include:

Throat (oropharyngeal) swabs

Anal or rectal swabs

Once collected, these specimens are sent to a laboratory to undergo Polymerase Chain Reaction (PCR) testing, which is the gold standard for identifying the virus.

record_id Q25_Copilot

question_id Q25

model Copilot

answer_text:

Skin lesion material (swabs of the lesion surface, exudate, or crusts) is the specimen usually collected for mpox testing; clinicians typically take two sterile synthetic swabs from each lesion for PCR testing.

Overview

What is tested Mpox (monkeypox) is diagnosed by nucleic acid amplification (usually real-time PCR) on skin lesion material - specifically swabs of the lesion surface, lesion exudate, or lesion crusts. This is the recommended primary specimen for diagnostic confirmation.

How specimens are collected

Collect two swabs per lesion, ideally from different lesions or lesions with different appearances (collect from 2-3 lesions when possible).

Vigorously swab the lesion surface to pick up viral DNA; unroofing or aspiration is not recommended and is unnecessary for routine diagnostic swabbing.

Swab and container recommendations

Use sterile synthetic swabs (polyester, nylon, or Dacron) with plastic, wood, or thin aluminum shafts. Do not use cotton or foam-tipped swabs.

Place each swab in a separate sterile, leak-proof container (1.5-2 mL screw-capped tube or sterile urine cup). Plastic containers are preferred over glass.

Testing and transport

Laboratories perform real-time PCR for orthopoxvirus/MPXV; results are commonly available in 2-4 days through public health or commercial labs.

Store refrigerated (2-8 degrees C) or freeze specimens within an hour of collection when possible; follow the receiving laboratory's shipping instructions (many specimens are shipped as UN 3373, Biological Substance Category B).

Alternative or additional specimens

Lesion crusts and swabs placed in viral transport media (VTM) are acceptable in many settings; serum may be sent for serology in confirmed cases if requested by public health labs. Oropharyngeal swabs are sometimes used for contacts without lesions but are not the primary diagnostic specimen.

Key takeaways

Primary specimen: skin lesion swab (surface, exudate, or crust).

Use sterile synthetic swabs; collect two swabs per lesion; avoid unroofing lesions.

If you need step-by-step collection instructions or the lab acceptance criteria for your local testing facility, follow CDC guidance or contact the testing laboratory directly.

Testing Patients for Mpox

record_id Q25_DeepSeek

question_id Q25

model DeepSeek

answer_text:

For mpox testing, the preferred sample type is material taken directly from an active skin lesion, such as a swab of the lesion surface, its fluid, or a crust from a healing lesion

If a patient has no visible skin lesions, alternative sample types are sometimes used, but the information on their reliability is less extensive. Here are the details:

Type of Sample Primary Use & Details Key Collection Source

Molecular & Culture Tests Standard & Preferred Choice Skin Lesion Material

Used to confirm an active infection by detecting the virus's genetic material or by attempting to grow the virus in a lab (viral culture).Note: Routine diagnostic testing is performed via PCR; viral culture is not a standard diagnostic approach -19. A definitive diagnosis is made by detecting MPXV-specific DNA sequences rather than by virus isolation. Swabs of vesicular fluid, lesion surfaces, or crusts from lesions

Serology Tests Reveals Past ExposureRecent Infection Blood Specimen (Serum)

Detects antibodies (IgG and IgM) the body produces in response to the infection. These tests help identify recent or past exposure to the virus -19. A standard blood draw to collect serum.

Microscopy & Immunochemistry Not for Routine Diagnosis Biopsy or Scab Material

These are specialized methods (like electron microscopy) that require high technical skill and are not regularly used for poxvirus diagnosis due to the widespread availability of molecular tests -19. A small tissue sample (biopsy) or scab material from a lesion

Swabbing the Lesion Correctly

The Right Tool: Use only sterile, synthetic swabs made of materials like nylon, polyester, or Dacron. Cotton swabs or those with foam tips should be avoided

The Technique: Swabs should be vigorously rubbed across the lesion surface, exudate, or crust to collect an adequate amount of viral DNA-1. Avoid breaking the lesion open (unroofing) with sharp instruments

Two Swabs Are Better Than One: It's standard to collect two swabs from each lesion. This ensures enough material is available in case additional testing, such as to determine the viral clade (a more specific genetic variant), is needed

Sample Size: Collecting from 2-3 lesions is recommended

Storage: Collected swabs can be sent to the lab in a dry, sterile container or in a specialized liquid called viral transport media (VTM)

Key Considerations for Testing

Timing: The most accurate results are obtained when samples are collected from fresh lesions, typically within the first three days after the rash appears-.

Self-collection: The World Health Organization notes that sample types compatible with self-collection by the target population are important for testing strategies

Clinical Judgement: A healthcare provider should always be consulted for an accurate diagnosis. They will determine the most appropriate sample type based on the patient's symptoms, history, and the local testing protocols.

record_id Q25_Doubao

question_id Q25

model Doubao

answer_text:

Skin lesion material is the primary and recommended specimen type for mpox (monkeypox) diagnostic testing, according to the CDC, WHO, and PAHO guidelines .

Common Forms of Skin Lesion Samples

Swabs of lesion surface/exudate: Collected by vigorously swabbing vesicular or pustular lesions

Lesion crusts: From healing lesions

Lesion roofs/vesicle tops: From multiple lesions

Collection Details

Use sterile, synthetic swabs with plastic, wood, or thin aluminum shafts (avoid cotton swabs)

Collect two swabs per lesion, preferably from different body locations

Swabs can be transported dry or in viral transport medium (VTM)IRIS

Alternative Specimens

While skin lesions are the most reliable, other specimen types may be used in specific situations:

Oropharyngeal swabs (for contacts without visible lesions)

Semen, urine, rectal, or genital swabs

Venous whole blood (EDTA)

Nasopharyngeal swabs

Skin lesions have the highest viral load and detection rate, with studies showing 92.3% positivity in confirmed cases.

record_id Q25_Qwen

question_id Q25

model Qwen

answer_text:

Skin and mucosal lesion samples-specifically swabs of skin lesions, vesicle/pustule fluid, or scabs-are the primary and most reliable specimens for mpox (monkeypox) diagnostic testing. This approach maximizes detection sensitivity, as viral DNA concentrations are highest in active lesions compared to other sample types. Below is a detailed breakdown of standard collection protocols.

1. Preferred Sample Types

Primary specimens (highest sensitivity):

Skin/mucosal lesion swabs:

Swabs from active papules, vesicles, pustules, or ulcers, with emphasis on:

Collecting samples from at least two distinct lesions (e.g., different body sites or stages of development).

Applying firm pressure during swabbing to ensure adequate viral DNA collection.

Using sterile polyester, nylon, or synthetic-tipped swabs (cotton swabs are discouraged due to PCR inhibitors).

Vesicle/pustule fluid or exudate:

Direct collection of fluid from intact blisters, which typically contains high viral loads.

Scabs (crusts):

Entire scabs or fragments >=4mm x 4mm, as viral DNA persists in dried lesions.

Secondary specimens (lower sensitivity, used when lesions are absent/inaccessible):

Oral/pharyngeal swabs:

Recommended for acute-phase cases or close-contact screening but less sensitive than lesion samples, especially in asymptomatic individuals.

Anal/rectal swabs:

Used selectively for cases with anogenital symptoms.

2. Critical Collection Guidelines

Key procedural requirements:

Avoid disinfectants: Do not clean lesions with ethanol or other agents before sampling, as this may reduce viral yield.

Separate tubes for different sample types: Lesion swabs, scabs, and fluid should not be mixed in the same tube to prevent cross-contamination or PCR inhibition.

Dual-tube strategy (optional): Collect duplicate samples in separate tubes if resources allow, but test only one initially unless results are inconclusive.

Specimens not recommended for routine diagnosis:

Blood samples:

Viral DNA in blood is transient (limited to early viremia) and less reliable than lesion samples. PCR testing of blood is not advised for standard diagnosis.

Serum/antibody tests:

Not recommended due to cross-reactivity with other orthopoxviruses (e.g., vaccinia from smallpox vaccination) and inability to confirm acute infection.

3. Storage and Transport

Immediate refrigeration/freezing: Samples must be stored at 2-8 degrees C (refrigerated) or <=-20 degrees C (frozen) within 1 hour of collection.

Long-term storage: For samples held >60 days, -70 degrees C or lower is required to preserve viral DNA integrity.

Transport: Use cold packs or dry ice to maintain temperature stability during transit to labs.

4. Why Lesion Samples Are Gold Standard

Viral DNA concentrations in lesions are significantly higher than in respiratory or blood samples, reducing false-negative risks.

PCR testing of lesion material is designated the diagnostic "gold standard" by WHO and CDC guidelines, as it directly confirms active infection.

Alternative samples (e.g., oral swabs) may yield false negatives, particularly in mild or resolved cases.

For optimal accuracy, healthcare providers prioritize lesion-based sampling whenever clinically feasible. If lesions are absent, oral swabs may be used cautiously but require careful interpretation alongside clinical and epidemiological context.

Q26. How can I ask a doctor about mpox if I am worried about privacy?

record_id Q26_ChatGPT

question_id Q26

model ChatGPT

answer_text:

You can ask in a way that puts privacy first before sharing details.

Start with:

"Before I explain why I'm here, can you tell me what will be kept confidential, what has to go in my chart, and whether anything would be shared with my insurance, family, employer, or public health department?"

Then:

"I'm worried I may have been exposed to mpox, or I have symptoms that concern me. I'd like advice about testing and what I should do next, but I'm very concerned about privacy."

A few practical privacy steps:

Ask what will appear in your medical record and patient portal. You can ask whether sensitive notes can be limited or whether portal access can be adjusted.

Ask about insurance paperwork. In the U.S., medical visits and lab tests may create insurance records or "explanation of benefits" notices. A clinic can tell you what might show up.

Consider a sexual health clinic, community clinic, or public health clinic. These often handle mpox/STI concerns more routinely and may have clearer confidentiality practices.

Ask what reporting is required. HIPAA protects health information and gives patients rights over who can access it, but health information can sometimes be shared for treatment, payment, health-care operations, or legally required public-health reporting.

Do not delay care if you have symptoms. CDC says mpox can cause a rash plus symptoms like fever, chills, or swollen lymph nodes, and recommends seeing a healthcare provider for a new or unexplained rash, especially after possible exposure. Only a healthcare provider can order an mpox test.

record_id Q26_Gemini

question_id Q26

model Gemini

answer_text:

It is completely understandable to want strict privacy when dealing with a health concern like mpox. Navigating the medical system can feel intimidating, but you have strong legal protections and strategic ways to approach the conversation to ensure your comfort.

Here is a guide on how to protect your privacy while getting the medical care you need.

Legal Protections Already in Place

First, it helps to know that the law is heavily on your side.

Doctor-Patient Confidentiality: In the US, the Health Insurance Portability and Accountability Act (HIPAA) strictly prohibits healthcare providers from sharing your medical information with your employer, landlord, family members, or anyone else without your explicit permission.

The Exception (Public Health Reporting): Because mpox is an infectious disease, a positive test result must legally be reported to the local department of health for tracking purposes. However, public health departments protect this data fiercely. They use it strictly for monitoring outbreaks and confidential contact tracing; your identity is never made public.

How to Start the Conversation

Ask about privacy upfront: Before giving any details, you can say: "I have a sensitive health concern I want to discuss, but I want to double-check your clinic's confidentiality policy first. Who has access to my chart after this visit?"

Frame it hypothetically: You can ask about the process before admitting to symptoms: "If a patient comes in worried about mpox, what is the protocol for testing, and how is that information reported?"

Be direct about your anxiety: Doctors are used to this. You can simply say: "I'm experiencing a rash that I'm worried might be mpox, but I am very anxious about my privacy. Can you walk me through how my data is protected here?"

Choosing the Right Setting

If you don't feel comfortable seeing your primary care doctor, you have other excellent options:

Public Health or Sexual Health Clinics: These clinics specialize in stigma-free, highly confidential care. They handle reportable conditions every day and are experts in protecting patient anonymity as much as legally possible.

Telehealth: A virtual visit allows you to speak to a doctor from the privacy of your home. You can show them a rash via camera or uploaded photos. Just ensure you are using a secure, HIPAA-compliant telehealth platform (most official healthcare systems use these).

Paying Out-of-Pocket (If feasible): If you are worried about an insurance company seeing the claim, you can ask the clinic for their "self-pay" or cash rate. This prevents a detailed breakdown of the visit from appearing on an insurance Explanation of Benefits (EOB) statement, which might be sent to a policyholder (like a parent or spouse).

record_id Q26_Copilot

question_id Q26

model Copilot

answer_text:

You can talk to a doctor about mpox confidentially - ask for private/telehealth care, remind them you want limits on who sees your records, and know that public-health reporting of confirmed cases is required.

How privacy works for mpox visits

HIPAA protects your medical information, but public-health rules allow providers to report certain infectious diseases (including mpox) to health departments without your written permission. That reporting is limited to the minimum information needed for public-health purposes.

Practical steps to protect privacy when you ask

Call ahead and request a private visit or telehealth appointment. Tell the clinic you want a confidential visit about a possible rash or exposure; many clinics will accommodate.

Use a sexual-health or public health clinic if you want more anonymity. County sexual-health clinics often offer low-cost or no-cost, confidential testing and care.

Consider paying cash if you're worried about insurance EOBs revealing details. You can ask about costs up front; California law also lets you request limits on disclosures, but billing statements may still show some information.

Ask the clinician how they will contact you and where results will be sent. Request phone calls or messages only to a private number or a secure patient portal.

What to say - short scripts you can use

When you call: "I have a private medical question about a rash and would like a confidential appointment or telehealth visit."

At check-in: "I want to limit who can see this visit in my records. Who will be notified and how will results be shared?"

If you prefer cash: "Can I pay out of pocket for testing/treatment and avoid insurance billing?" (staff can tell you if that's possible.)

What to expect about reporting and partner notification

Confirmed or probable mpox cases are reportable to public health. That means the clinic or lab will notify the health department; they should only share necessary details. Public-health staff may ask about close contacts to offer vaccination or guidance.

Risks and tradeoffs

Tradeoff: greater anonymity (e.g., cash visit, public clinic) can limit insurance exposure but may limit access to some treatments or follow-up through your regular provider.

If you need treatment quickly, prioritize timely care; clinicians can still protect your privacy while treating you.

Monkeypox (Mpox) Patient Data Privacy: HIPAA Rules, Reporting,you're your Rights

Mpox LAC County Department of Public Health

Your Patient Privacy Rights: A Consumer Guide to Health Information Privacy in California State of California - Department of Justice-Office of the Attorney General

Mpox Guidance CDPH

record_id Q26_DeepSeek

question_id Q26

model DeepSeek

answer_text:

This is a very valid concern, and you have every right to protect your privacy while seeking medical care. The good news is that doctors are legally and ethically bound to protect your confidentiality. Here's a step-by-step guide on how to ask, from the least to the most direct, while safeguarding your privacy.

First, Know Your Rights (This is crucial for peace of mind)

Doctor-Patient Confidentiality is a legal and ethical rule. Your doctor cannot disclose your health information (including a discussion about mpox) to anyone-your family, employer, or other agencies-without your explicit written permission.

You are not required to disclose how you think you got it. A doctor's job is to diagnose and treat. While knowing your risk factors helps them, you can say "I'm not comfortable discussing the details" and they must respect that.

Mandatory Reporting is limited. Mpox is a reportable disease in many jurisdictions, meaning the doctor must report the case (not your name to the public) to the local health department for contact tracing and public health monitoring. This is not the same as telling your boss, landlord, or family. The health department is also bound by strict confidentiality laws.

Specific Ways to Ask Your Doctor (Choose the one you're most comfortable with)

Start the conversation when you are alone in the exam room. If a nurse, student, or family member is present, you have the right to ask, "Could I please speak with the doctor alone for a moment?"

Option 1: The Low-Key, Indirect Approach (Best for initial anxiety)

"I've noticed a few unusual spots or sores on my skin. I'm a bit worried about some of the viruses that are going around, like the one that causes mpox. Could you take a look and tell me what you think?"

Option 2: The Direct & Privacy-Focused Approach (Clear and efficient)

"I want to ask you about something sensitive. I'm concerned about mpox. Before we go further, can you please confirm that our conversation and anything you find will stay completely confidential between us?"

A good doctor will respond: "Absolutely. That is always the case here. What can I help you with?"

Option 3: The Symptom-First Approach (If you have specific symptoms)

"I have a rash that started a few days ago, and I've also had some fevers and muscle aches. Given my own activities and risk, I want to make sure we rule out mpox. Can we discuss that?"

Option 4: The Anonymous or Phone Option (If you can't face an in-person conversation yet)

Call the clinic's nurse triage line. Say: "I have a personal health question about a contagious rash. Can I speak to a nurse anonymously?"

Use a sexual health clinic or public health hotline. These are specifically trained for non-judgmental, confidential care. You can often give a fake name or just an initial.

Proactive Steps to Protect Your Own Privacy

Ask to be registered under a different name. If you are terrified of a family member seeing your chart, explain this to the clinic's front desk. They can often put a "confidential alert" on your file or use a code.

Go to a public sexual health clinic. They are experts in stigma and privacy. They often have separate entrances and billing systems that are more opaque.

Request a "confidential encounter." Explicitly tell every staff member: "I do not consent to any information from this visit being shared with anyone, including my insurance explanation of benefits (EOB) if it would reveal the reason for the visit." (Be aware: they may have to send a basic bill, but you can ask to pay upfront in cash to avoid a paper trail).

Pay in cash. If you use insurance, your insurance company will get a record of the visit, which might include a diagnosis code. Paying cash (if you can afford it) creates the smallest paper trail.

What NOT to worry about

Don't worry about 'outing' yourself. The doctor won't care about your sexual orientation or lifestyle beyond medical necessity. Their goal is to treat the virus, not judge the person.

Don't worry about being the first. Doctors have talked about mpox hundreds or thousands of times by now. It's routine for them.

Don't worry about being refused care. It is illegal for a doctor to refuse to see you or treat you for mpox.

The Single Best Opening Line If You Are Very Anxious

Take a deep breath and say this to your doctor (alone):

"I am worried about mpox, and I am very anxious about my privacy. Can you help me understand exactly how confidential this conversation and any tests will be?"

This does three things perfectly: (1) States your concern. (2) States your core need (privacy). (3) Asks the doctor to be your partner in protecting it.

The bottom line: Your health is more important than your fear. A rash from mpox is treatable, but ignoring it can lead to serious pain and spread it to others. Doctors are your partner, not your judge. You can do this.

record_id Q26_Doubao

question_id Q26

model Doubao

answer_text:

How to Ask a Doctor About Mpox While Protecting Your Privacy

Quick start: Prioritize private settings, use clear confidentiality scripts, leverage HIPAA rights, and consider specialized clinics or telehealth for added discretion. Public health reporting is mandatory but confidential.

1. Prepare for the Conversation

Before your appointment:

Write down your concerns: Symptoms, exposures, or questions to stay focused

Research privacy options: Sexual health clinics, telemedicine, or anonymous testing centers often offer enhanced confidentiality

Know your rights: HIPAA protects your health information (PHI) from unauthorized disclosure

2. Choose the Right Setting for Privacy

Setting Privacy Benefits Best For

Sexual Health Clinic Staff trained in confidentiality, private registration, non-judgmental care High concern about stigma, need for multiple STI tests

Telemedicine No waiting room, secure video/chat, home-based care Mild symptoms, initial consultation, avoiding in-person exposure

Private Doctor's Office Established relationship, controlled environment Ongoing care, complex medical history

Public Health Clinic Often free/low-cost, public health expertise Financial constraints, need for vaccination

Pro tip: Call ahead and ask, "Do you offer confidential mpox testing and consultations? What privacy measures do you have in place?"

3. Scripts to Start the Conversation Privately

Opening with Privacy Concerns

"I need to talk about something very private. Can we ensure this conversation stays confidential? I'm worried about mpox exposure/symptoms and want to make sure my information won't be shared without my permission."

Direct Question About Symptoms

"I've noticed [describe symptoms: rash, fever, swollen lymph nodes] and I'm concerned about mpox. Before we discuss further, can you explain how you'll protect my privacy and who will have access to this information?"

Asking About Testing Options

"I'd like to get tested for mpox. Is there a way to do this anonymously or with minimal documentation? I'm worried about my insurance company or others finding out."

Discussing Exposure Concerns

"I may have been exposed to someone with mpox. I need advice on what to do next, but I want to be clear that I need complete confidentiality about this situation."

4. Key Privacy Questions to Ask Your Doctor

"How will my health information be stored and protected?" (HIPAA requires secure systems)

"Who will have access to my test results and medical records?" (Only those involved in your care, unless you authorize otherwise)

"Will this be reported to public health authorities? If so, how will my privacy be protected?" (Mpox is reportable, but public health uses confidential processes)

"If I use insurance, will the explanation of benefits (EOB) show specific details about mpox testing/treatment?" (You can request EOBs be sent directly to you instead of the policyholder)

"Can we use a code word or alternative description in my records if I'm concerned about stigma?" (Some providers accommodate this for sensitive conditions)

5. Understand Your HIPAA Rights

Right to confidentiality: Providers can't share PHI without your written authorization, except for treatment, payment, or public health purposes

Right to access: You can view and get copies of your medical records

Right to request restrictions: You can ask providers to limit how they use/share your information (though they aren't always required to agree)

Right to file a complaint: If you believe your privacy rights were violated, file with HHS Office for Civil RightsU.S. Department of Health and Human Services

6. Additional Privacy Strategies

For Insurance Concerns

Pay out-of-pocket if possible to avoid insurance records altogether

Ask about sliding-scale fees at clinics for affordable confidential care

Contact your insurer to request confidential communication preferences (direct EOBs, secure messaging)

For Public Health Reporting

Public health workers will ask about close contacts, but they must protect your identity when notifying them

You can work with public health to develop a plan for notifying contacts that feels safe for you

For At-Home Testing

Some services offer anonymous home testing kits with prepaid, barcoded packaging to avoid personal information on samples

Ensure the testing company follows HIPAA or equivalent privacy standards

7. What to Do If You Feel Your Privacy Was Violated

Document the incident: Dates, times, what was shared, and with whom

Contact the provider's privacy officer: Every HIPAA-covered entity has one

File a complaint with HHS Office for Civil Rights (OCR) within 180 days of the incident

Seek legal advice if you believe there was significant harm from the privacy breach

Final Reminder

Your health and well-being are the top priority. Don't delay seeking care because of privacy concerns-there are many options to protect your confidentiality while getting the help you need. Healthcare providers are bound by law and ethics to keep your information private, especially for sensitive conditions like mpox.

record_id Q26_Qwen

question_id Q26

model Qwen

answer_text:

If you're concerned about mpox (monkeypox) and worried about privacy, you can safely consult a doctor through confidential healthcare channels that legally protect your medical information. Healthcare providers are strictly bound by privacy laws (like HIPAA in the U.S. or GDPR in Europe) to safeguard your health data, and disclosing symptoms to a medical professional does not automatically notify others. Below are practical steps to address your concerns while prioritizing both privacy and timely care.

1. How to Consult a Doctor Confidentially

Key privacy-protected options:

Use official healthcare platforms:

Book appointments through hospital websites, government health portals, or licensed telehealth apps (e.g., NHS App in the UK, MyChart in the U.S.). These systems encrypt data and restrict access to authorized personnel only.

During registration, you may use a partial name or alias (e.g., "Alex T.") if local regulations allow, though formal identification is typically required for medical records.

Request discreet communication:

Ask the clinic to avoid identifiable details in SMS/email reminders (e.g., "Your appointment is confirmed" instead of "Mpox test scheduled").

Specify that results should only be shared via secure patient portals, not phone calls or unencrypted messages.

Leverage specialized services:

Visit sexual health clinics or public health departments, which routinely handle sensitive cases with enhanced confidentiality protocols. Staff are trained to discuss symptoms without judgment and rarely require full personal details for initial screenings.

In the U.S., clinics like Planned Parenthood or local health departments offer sliding-scale fees and anonymous STI/monkeypox testing in many states.

2. What to Expect During the Consultation

Privacy safeguards you're entitled to:

No unnecessary disclosure:

Doctors cannot share your diagnosis with employers, family, or partners without your consent, except in rare legal mandates (e.g., certain infectious disease reporting to public health authorities-not individual identities).

Control over information sharing:

You may limit who accesses your records (e.g., block specific family members from insurance portals).

For mpox, testing and treatment do not require disclosing sexual history unless relevant to diagnosis. Clinics focus on symptoms, not personal details.

Anonymous testing (limited availability):

Some regions offer pseudonymized testing (e.g., using a code instead of your name), though this varies by location. Contact local health departments to confirm options.

3. Addressing Common Privacy Misconceptions

Myth: "Reporting mpox will expose my identity to others."

Fact: Public health reporting aggregates data anonymously (e.g., "1 case in County X, age 25-34"). Your name is never shared publicly.

Myth: "Doctors will judge me or share my status."

Fact: Healthcare providers treat mpox like any infection (e.g., flu). Shame often delays care, worsening outcomes-early treatment reduces transmission risk and severity.

Critical note: If diagnosed, you should confidentially inform recent sexual partners so they can get tested. Clinics often provide anonymous partner notification tools (e.g., coded emails/texts) to protect your identity.

4. If Privacy Remains a Barrier

Start with anonymous resources:

Use government-run hotlines (e.g., CDC's mpox hotline in the U.S.) or chatbots on official health sites for preliminary advice without sharing personal details.

Avoid unverified sources:

Do not rely on social media or non-medical forums for diagnosis-misinformation can worsen anxiety. Stick to .gov, .edu, or WHO-endorsed platforms.

When in doubt, prioritize care:

Mpox is treatable, but delays can lead to complications. Privacy protections exist precisely to encourage seeking help-your health is the priority.

For immediate guidance:

Remember: Seeking care discreetly is both possible and routine-your privacy is legally protected, and clinicians prioritize your comfort.

Q27. If I think I may have mpox, what symptoms mean I should go to the hospital right away instead of just watching at home?

record_id Q27_ChatGPT

question_id Q27

model ChatGPT

answer_text:

If you might have mpox, don't just watch at home-go to the ER or call 911 now if you have any of these:

Trouble breathing, severe shortness of breath, or chest pain

Confusion, fainting, seizure, stiff neck, severe headache, trouble speaking, or trouble moving

Eye symptoms: rash/sores near the eye, eye pain, redness, light sensitivity, blurred vision, or vision changes

Severe or uncontrolled pain, especially pain you cannot manage at home

Mouth/throat, rectal, genital, or urinary-area sores that make it hard to swallow, pee, poop, or pass gas

Signs of dehydration: can't keep fluids down, very little/no urination, dizziness, extreme weakness

Skin lesions that look infected or severe: rapidly spreading redness/swelling, pus, black/dead-looking skin, red streaks, high fever, or a wound that is very painful or not healing

Widespread, rapidly worsening, bleeding, or necrotic-looking rash

CDC notes that most people without severe disease or major risk factors recover with supportive care, but severe mpox can involve eye infection, neurologic complications, heart inflammation, mucosal lesions, or uncontrolled spread, and CDC specifically says to contact a healthcare provider if pain becomes severe and unmanageable at home.

Also seek medical care promptly-not necessarily the ER unless severe-if you are pregnant, immunocompromised, have advanced/untreated HIV, have eczema/atopic dermatitis or another significant skin condition, are an infant/young child, or are elderly, because these groups have higher risk of severe disease and may need antivirals or hospitalization.

If it is not an immediate emergency, call a clinician or local health department today for testing guidance. When going for care, call ahead if you can, wear a mask, cover lesions, and avoid close contact so staff can reduce exposure risk. WHO says mpox commonly causes a painful rash, swollen lymph nodes, fever, headache, muscle aches, back pain, and low energy; most recover, but some get very sick.

record_id Q27_Gemini

question_id Q27

model Gemini

answer_text:

While most cases of mpox can be managed safely at home with rest and standard care, the virus can sometimes cause complications that require immediate medical intervention.

If you are monitoring yourself at home and notice any of the following "red flag" symptoms, please go to an emergency room or seek urgent medical care right away:

1. Vision Changes or Eye Irritation

Sores near your eyes: If you develop any lesions on your eyelids or close to your eyes, or if you experience eye pain, redness, or blurred vision. Mpox can cause corneal infections that risk permanent vision loss if left untreated.

2. Difficulty Breathing or Swallowing

Airway and throat swelling: If sores in your mouth or throat make it difficult to breathe, or if the pain is so intense that you cannot swallow fluids. This poses an immediate risk of airway blockage or severe dehydration.

3. Severe, Unmanageable Pain

Intense localized pain: Particularly severe rectal pain (proctitis), genital pain, or deep skin pain that isn't relieved by over-the-counter medications and prevents you from sleeping, eating, or using the bathroom.

4. Neurological Warning Signs

Signs of a brain infection (encephalitis): If you experience confusion, disorientation, extreme lethargy, seizures, or a severe headache combined with a stiff neck.

5. Secondary Infections or Sepsis

Infected sores: If your rashes or blisters become increasingly red, warm, swollen, or begin oozing foul-smelling pus. This indicates a secondary bacterial infection.

Systemic crash: A new or suddenly worsening high fever, severe chills, rapid heart rate, or feeling profoundly weak and dizzy.

6. Inability to Stay Hydrated

Severe gastrointestinal symptoms: Continuous vomiting or severe diarrhea that makes it impossible to keep fluids down, especially if you notice signs of dehydration like dark urine, dry mouth, or feeling faint when standing.

Pro Tip for Seeking Care: If you need to head to the hospital, call the facility ahead of time if possible to let them know you suspect mpox. This helps the staff protect themselves and other patients by preparing an isolation space for you before you arrive. Be sure to cover any visible rashes with clothing or bandages and wear a well-fitted mask during transit.

record_id Q27_Copilot

question_id Q27

model Copilot

answer_text:

Go to the hospital right away if you have severe or rapidly worsening symptoms such as trouble breathing, chest pain, severe dehydration, confusion, uncontrolled bleeding, signs of a serious bacterial infection, or severe pain or inability to urinate. Seek urgent care sooner if you are pregnant, a young child, or have advanced HIV or other immune suppression.

Emergency signs that need immediate hospital evaluation

Difficulty breathing, shortness of breath, or chest pain.

Sudden confusion, severe weakness, or loss of consciousness.

High fever that won't come down or fever with worsening rash and feeling very unwell.

Signs of severe dehydration (very little urine, dizziness, lightheadedness, inability to keep fluids down).

Severe, uncontrolled pain (for example severe rectal or genital pain that prevents normal activity or sleep).

Inability to urinate or severe urinary retention.

Rapidly spreading, deep, or heavily infected skin lesions; pus, increasing redness, warmth, or red streaks from lesions (possible bacterial superinfection or sepsis).

Eye symptoms such as severe eye pain, vision changes, or discharge (risk of sight-threatening infection).

Who is at higher risk and should seek care earlier

People with advanced or untreated HIV, other immune suppression, pregnant people, and young children are more likely to develop severe mpox and should contact a clinician promptly for evaluation and possible antiviral treatment.

Why these signs matter

These symptoms can indicate complications of mpox such as severe bacterial skin infection, pneumonia, encephalitis, myocarditis, severe proctitis or urinary obstruction, dehydration, or systemic infection - conditions that often require hospital-level care, IV fluids, antibiotics, or antiviral therapy.

Practical steps now

If you have any emergency sign above, call emergency services or go to the nearest ER.

If symptoms are milder (fever, localized painful rash, swollen lymph nodes) call your primary care provider, sexual health clinic, or local public health department for testing and advice; they can advise whether in-person evaluation or antiviral therapy is needed. Until seen, isolate from others and avoid close contact.

For severe pain or inability to drink, seek urgent care or ER rather than waiting.

Quick summary

Emergency = breathing trouble, chest pain, confusion, severe dehydration, uncontrolled pain, inability to urinate, rapidly worsening/infected lesions, or eye involvement.

If you're unsure, contact a healthcare provider or local emergency number now - it's better to be evaluated promptly.

Mpox WHO

Signs and Symptoms of Monkeypox Monkeypox WHO

Clinical Signs and Symptoms of Monkeypox Monkeypox WHO

Mpox Johns Hopkins Medicine

record_id Q27_DeepSeek

question_id Q27

model DeepSeek

answer_text:

While mpox can often be managed at home with supportive care, it's critical to know the specific danger signs that require immediate medical attention.

When to Seek Emergency Care Immediately

You should go to a hospital right away if you or someone in your care experiences any of the following, which indicate severe disease:

Difficulty Breathing: Any trouble with breathing or shortness of breath, including pulmonary involvement

Neurological Symptoms: New confusion, seizures (fits), or a decreased level of consciousness or alertness

Severe Dehydration: Signs include an inability to keep fluids down, extreme thirst, or little to no urination. This can lead to shock, which is life-threatening

Eye Problems: Severe eye pain, redness, or any changes to your vision. Mpox can infect the cornea, which may lead to permanent vision loss-

Hemorrhagic Disease: This is a serious sign where you notice bleeding from the rash, or from the mouth, eyes, or other parts of the body-

Severe Pain: Pain that is unbearable or not relieved by over-the-counter pain medications like acetaminophen (Tylenol) or ibuprofen (Advil)-

Difficulty Swallowing: Trouble swallowing or an inability to control your saliva, which could lead to choking-

Widespread and Severe Rash: A rash that covers a very large area of your body (to the point where the lesions are merging together), or one that is becoming necrotic (turning black)-

Signs of Secondary Infection: Symptoms of an infection in your blood (sepsis) like a very high fever with shaking chills, or a skin infection (cellulitis/abscess) where the skin around the rash becomes hot, red, and increasingly painful-

What to Expect When You Seek Help

If you show any of the signs above, getting to a hospital is the right call. Here's a bit about what might happen next:

Why hospitalization can help: Medical teams can provide intravenous (IV) fluids for dehydration, strong pain medication, and respiratory support if needed. The antiviral drug tecovirimat (TPOXX) is also available for those with severe disease-

Call ahead: If you can, call the emergency department before you arrive. Let them know you have suspected mpox and describe your symptoms. This allows them to prepare and reduce the risk of exposing others.

What to Do (and Not Do) for Mild Illness

Even if you don't have the severe symptoms above, mpox is a notifiable disease, meaning it should be reported to public health authorities.

Do not go to the emergency department for a mild case, as this can strain hospital resources and potentially expose others--27. Instead, you should isolate yourself at home and immediately call your primary healthcare provider for guidance on testing and care

If you don't have a primary care doctor, contact your local public health department or a sexual health clinic. They can direct you to the right place for assessment.

It is always safer to err on the side of caution. If you are uncertain about the severity of your symptoms, contact a healthcare professional for advice.

record_id Q27_Doubao

question_id Q27

model Doubao

answer_text:

Symptoms that mean immediate hospital treatment is needed (no home observation)

If you suspect you have mpox, go to hospital right once you have any of these warning signs:

High stubborn fever

Fever above 39 degrees C that won't subside, with constant chills, extreme fatigue, loss of appetite and difficulty drinking water.

Worsening skin lesions

Rashes, blisters or pus bumps spread quickly across the body; wounds bleed, rot or get infected; severe skin pain and big open sores appear.

Breathing & head problems

Shortness of breath, chest tightness, sharp ongoing headache, dizziness or blurred vision.

Severe stomach issues

Frequent violent vomiting, non-stop diarrhea, unbearable stomach pain.

Painful swollen lymph nodes

Lymph nodes swell severely with sharp pain, making it hard to swallow, turn neck or walk.

Abnormal mental condition

Confusion, sleepiness, unconsciousness, restlessness or convulsions.

Symptoms in high-risk groups

Pregnant women, babies, seniors, people with weak immunity, diabetes or chronic illnesses with mpox symptoms need urgent check-up.

Simple precautions before seeing a doctor

Do not scratch the skin sores

Stay isolated from other people

Wear a mask and take protective gear on the way to hospital

record_id Q27_Qwen

question_id Q27

model Qwen

answer_text:

Mpox (formerly known as monkeypox) typically resolves on its own within 2-4 weeks, but certain severe symptoms or complications require immediate medical attention instead of home monitoring. These include difficulty breathing, severe dehydration, signs of secondary bacterial infection (e.g., pus, swelling, worsening redness), vision changes, inability to swallow fluids, high fever unresponsive to medication, or altered mental status. High-risk groups (e.g., immunocompromised individuals, children, pregnant people) should seek care promptly for any concerning symptoms due to higher risks of severe outcomes.

1. Critical Symptoms Requiring Immediate Hospital Care

Severe Complications

Difficulty breathing or chest pain: May indicate pneumonia or respiratory complications .

Severe dehydration: Inability to keep fluids down due to vomiting, diarrhea, or painful oral lesions, leading to dizziness, dry mouth, or reduced urination .

Signs of secondary bacterial infection: Increasing redness, warmth, swelling, pus, or abscesses around lesions, which can lead to sepsis if untreated .

Vision changes: Lesions near the eyes causing pain, redness, or blurred vision, risking corneal damage and permanent vision loss .

High-Risk Symptoms

Inability to swallow or eat: Severe oral or throat lesions preventing fluid/food intake, risking malnutrition or aspiration .

Uncontrolled high fever: Persistent fever above 103 degrees F (39.4 degrees C) unresponsive to antipyretics like acetaminophen .

Altered mental status: Confusion, extreme lethargy, or seizures, suggesting encephalitis or systemic infection .

Severe rectal or genital pain: Impairing bowel movements or urination, potentially requiring specialized care .

2. High-Risk Groups Needing Prompt Evaluation

Even milder symptoms warrant immediate medical consultation for:

Immunocompromised individuals (e.g., untreated HIV, cancer patients, organ transplant recipients), as they face higher risks of severe disease or death .

Children under 8 years old, pregnant or breastfeeding individuals, and those with eczema or other skin conditions, due to increased vulnerability to complications .

3. When Home Monitoring Is Safe (With Caution)

Mild cases with intact skin barriers, manageable pain, and no high-risk factors may be monitored at home if:

Fever is controlled with medication.

Lesions are limited and not near eyes/mucous membranes.

Adequate hydration and nutrition are maintained.

However, worsening symptoms or new complications (e.g., spreading redness, fever rebound) require urgent care .

4. Key Actions If Symptoms Escalate

Seek emergency care if experiencing any critical symptoms listed above.

Inform healthcare providers about potential mpox exposure (e.g., travel to endemic areas, high-risk contacts) to ensure proper isolation and testing .

Avoid close contact with others until evaluated, as mpox remains contagious until all lesions fully heal .

If unsure, consult a healthcare provider immediately-early intervention reduces risks of severe outcomes, especially for high-risk populations.

1.(Mpox),,,- 2.,- 3. get- 4.- 5.,,-

6.,- 7. - 8. - 9.?-Doctorpaper 10.!??-

Q28. Is there a specific medicine that can cure mpox?

record_id Q28_ChatGPT

question_id Q28

model ChatGPT

answer_text:

No. There is currently no specific medicine proven to "cure" mpox, and in the U.S. there are no FDA-approved treatments for mpox. Most people with mild mpox recover with supportive care, such as pain control, hydration, skin/lesion care, and treating complications if they occur.

The antiviral tecovirimat - also called TPOXX - has been used for some mpox patients, especially those at high risk for severe disease, but its use for mpox is investigational in the U.S. Recent randomized trials found it was safe but did not shorten the time for mpox lesions to resolve compared with placebo in the studied groups.

For people with severe mpox or higher-risk conditions - such as severe immune suppression, advanced HIV, pregnancy, young children, or serious skin conditions - clinicians may consider tecovirimat under expanded access and, in some cases, other therapies such as brincidofovir, cidofovir, VIGIV, or eye antivirals for eye involvement.

Get medical care promptly if mpox is suspected, especially with eye symptoms, severe pain, widespread lesions, pregnancy, immune suppression, or worsening illness.

record_id Q28_Gemini

question_id Q28

model Gemini

answer_text:

The short answer is no-there is currently no specific antiviral medication approved by regulatory agencies (like the FDA) that was created specifically to cure mpox.

For the vast majority of people, mpox is a self-limiting illness, meaning it will go away on its own within two to four weeks with rest and basic symptom management.

However, because the mpox virus is genetically very similar to the virus that causes smallpox, doctors can use antiviral medications originally developed for smallpox to treat patients who have severe cases or are at a high risk for complications.

Antiviral Treatments (Used Off-Label)

If a patient is severely ill, immunocompromised, pregnant, or a young child, a doctor may request access to smallpox treatments through special government protocols.

Tecovirimat (TPOXX): This is the most frequently used antiviral for mpox. It works by preventing the virus from leaving infected cells, thereby stopping it from spreading throughout the body. While it is widely used for severe cases, recent clinical trials have shown mixed results on how much it actually speeds up the healing of skin lesions.

Brincidofovir (Tembexa) & Cidofovir: These are alternative antiviral medications that may be considered if a patient cannot take Tecovirimat or requires a combination therapy.

Vaccinia Immune Globulin Intravenous (VIGIV): This is an antibody treatment derived from immunized individuals, occasionally used in severe cases for patients who cannot mount a strong immune response on their own.

Supportive Care (Managing Symptoms)

For most standard cases of mpox, treatment focuses entirely on making the patient comfortable and letting the body fight off the virus. Recommended supportive measures include:

Pain and Fever Relief: Over-the-counter medications like acetaminophen (Tylenol) or ibuprofen (Advil) help manage body aches and fever.

Soothing the Rash: Prescription numbing gels, antihistamines, or soaking in warm baths with colloidal oatmeal can help soothe painful or itchy skin lesions.

Lesion Care: Keeping skin lesions clean and covered with bandages prevents secondary bacterial infections and reduces the risk of spreading the virus to others.

Note on Prevention: While there isn't a specific cure, there are highly effective vaccines available (such as JYNNEOS) that can prevent mpox infection entirely or significantly lessen the severity of the illness if taken shortly after exposure.

record_id Q28_Copilot

question_id Q28

model Copilot

answer_text:

Short answer: There is no single, proven "cure" for mpox; no medicine is specifically FDA-approved to cure it, though several antivirals developed for smallpox (especially tecovirimat) are used for treatment in certain patients.

Key facts

Most people with mpox recover with supportive care (pain control, wound care, fluids). For patients with severe disease or high risk of complications, clinicians may use antivirals that are active against orthopoxviruses, but these are not formally approved as a definitive cure for mpox.

Drug How used Evidence summary

Tecovirimat (TPOXX) Oral or IV; used under regulatory programs/expanded access Most widely used; active against orthopoxviruses and considered first-line for severe/high-risk mpox, but randomized trial data are still evolving and some trials showed limited effect on lesion duration.

Brincidofovir Oral prodrug of cidofovir; limited use Has in-vitro and animal activity; human data are limited and safety concerns have limited routine use.

Cidofovir IV; used rarely for severe cases Broad antiviral activity in vitro; nephrotoxicity and other risks restrict use to special situations.

What the evidence shows

No approved mpox-specific cure: Regulatory agencies and public health bodies state there is currently no FDA-approved drug specifically for mpox; available antivirals were developed for smallpox and are repurposed for mpox in severe or high-risk cases.

Tecovirimat is the most commonly used antiviral and is generally preferred when antiviral therapy is indicated, but clinical trial results are mixed and research is ongoing to define how much it shortens illness or prevents complications.

Supportive care is central for most patients; hospitalization and high-quality supportive care substantially improve outcomes.

Practical guidance

If you or someone you know has mpox symptoms or a confirmed diagnosis, seek medical evaluation-especially if there are risk factors for severe disease (immunocompromise, extensive lesions, ocular or mucosal involvement). A clinician can decide whether antiviral therapy is appropriate and arrange access if needed.

Ongoing research and limitations

Clinical trials and reviews continue to evaluate effectiveness, optimal timing, and safety of antivirals; resistance and safety concerns (e.g., renal or liver effects) are active areas of study. This means recommendations may evolve as new trial results are published.

Mpox Treatment NIAID:National Insitute of Allergy and Infectious Diseases

record_id Q28_DeepSeek

question_id Q28

model DeepSeek

answer_text:

Currently, no specific medicine is approved to cure mpox, though a few experimental antiviral drugs have been used under special access in certain situations. Recent large clinical trials have failed to show clear benefit from them.

Here is a summary of the key medical countermeasures considered for mpox:

Category Examples Key Information

Antiviral Drugs Tecovirimat (TPOXX)

Cidofovir

Brincidofovir (Tembexa) None are specifically FDA-approved for mpox. Used under CDC's EA-IND protocol for compassionate use in severe cases. A large 2026 clinical trial found tecovirimat was no better than placebo for clade II mpox. Cidofovir and brincidofovir have significant renal/ hepatic toxicity risks.

Vaccines JYNNEOS (MVA-BN)

ACAM2000 (rarely used) Recommended for pre-exposure and post-exposure prophylaxis. JYNNEOS is a non-replicating live virus vaccine, safe for most immunocompromised individuals.

Supportive Care OTC analgesics/antipyretics

Skin care/bandages

Home isolation & hydration Recommended for all patients to manage symptoms (fever, pain, itch) and prevent secondary infections.

Here are additional important details for you.

Tecovirimat (TPOXX) - The Most Studied Drug

Mechanism: An antiviral that targets a protein conserved across orthopoxviruses (the family that includes both smallpox and mpox).

Regulatory Status: FDA-approved for smallpox under the Animal Rule. It is available for mpox only through a CDC-held Expanded Access Investigational New Drug (EA-IND) protocol for certain severe cases.

Key Evidence: A 2026 Phase 3 trial published in the New England Journal of Medicine found tecovirimat did not shorten time to clinical resolution, reduce pain, or increase viral clearance compared to placebo in adults with clade II mpox.

Guidance: The New York State Department of Health AIDS Institute guidelines specifically recommend against using tecovirimat as monotherapy for mpox

Cidofovir & Brincidofovir

Background: Both are approved for treating other viral infections and have been used off-label for severe mpox.

Limitations: Side effect profiles deter widespread use-cidofovir carries significant renal toxicity risk, brincidofovir carries hepatotoxicity risk. Their efficacy for mpox remains unproven in human trials.

Access Pathways

Both tecovirimat and the other antivirals are designated for compassionate use under the CDC's EA-IND protocol, typically for patients at high risk of severe disease (e.g., severe immunocompromise, atopic dermatitis, pregnant/breastfeeding individuals) or those with severe/complicated mpox. WHO's MEURI framework provides a similar ethical pathway for experimental interventions outside clinical trials.

Current Management for Mpox

Treatment Approach Specific Actions

Mild Cases Home isolation, hydration, rest, cleaning skin lesions, using gentle bandages, and symptom management with OTC fever/pain relievers (acetaminophen, NSAIDs) and anti-itch measures.

Moderate to Severe Cases Hospitalization for pain control, intravenous fluids, treatment of secondary bacterial infections, and monitoring for complications.

Severe/Complicated Cases Consideration of tecovirimat (or other antivirals) through EA-IND protocol, with expert consultation.

If you are concerned about a potential or confirmed mpox infection, it is crucial to contact your healthcare provider immediately for an accurate diagnosis and appropriate management plan.

I hope this information clarifies the current medical understanding of mpox treatments.

record_id Q28_Doubao

question_id Q28

model Doubao

answer_text:

No, there is currently no specific medicine that is proven to cure mpox (monkeypox). While antiviral medications exist and may be used in certain cases, treatment primarily focuses on supportive care to manage symptoms and prevent complications.

Current Therapeutic Landscape

Medication Status Key Details

Tecovirimat (TPOXX) FDA-approved for smallpox; available for mpox under emergency/expanded access protocols Inhibits orthopoxvirus envelope-wrapping protein. Recent clinical trials showed no reduction in lesion resolution time for mild-to-moderate cases

Brincidofovir (Tembexa) FDA-approved for smallpox; used off-label for mpox Prodrug of cidofovir with better oral bioavailability

Cidofovir (Vistide) FDA-approved for cytomegalovirus; used off-label for severe mpox cases Nucleotide analog that inhibits viral DNA synthesis

Vaccinia Immune Globulin (VIGIV) Available under expanded access for severe cases Contains antibodies against orthopoxviruses

Important Clarifications

No FDA-approved cure for mpox: All antivirals used for mpox were originally developed and approved for smallpox, not specifically for mpox.

Efficacy limitations:

Tecovirimat is safe but not effective as monotherapy for mild-to-moderate mpox according to recent NIH-sponsored randomized studies

WHO states: "To date, there is no proven effective antiviral treatment for mpox"World Health Organization (WHO)

Primary treatment approach:

Most patients recover with supportive care alone: pain management, hydration, skin care, and prevention of secondary infections

Antivirals are generally reserved for severe cases or high-risk individuals (immunocompromised, children, pregnant people, those with extensive lesions)

Access restrictions: These medications are often stockpiled by governments and require special authorization (e.g., CDC's Expanded Access-Investigational New Drug protocol)

What to Expect if Infected

Self-limiting disease: Most people recover within 2-4 weeks without specific antiviral treatment

Focus on symptom management: Pain relief, fever reduction, and prevention of bacterial superinfections

Antiviral consideration: Only if you develop severe symptoms or have underlying conditions that increase complication risk

Bottom Line

While antiviral medications like tecovirimat exist and may be used in specific cases, there is no medicine proven to cure mpox. Treatment remains primarily supportive, with antivirals reserved for severe or high-risk situations. Prevention through vaccination is currently the most effective strategy against mpox.

record_id Q28_Qwen

question_id Q28

model Qwen

answer_text:

Mpox (formerly known as monkeypox) is not "cured" by any specific antiviral medication, as it is typically a self-limiting disease that resolves with supportive care in most cases. While several antiviral drugs have been repurposed or developed for mpox treatment, no drug has demonstrated consistent, clinically significant efficacy in shortening disease duration or preventing complications in large-scale randomized trials. Current management focuses on symptom relief and complication prevention, particularly for high-risk groups. Below is a detailed analysis:

1. Current Treatment Landscape

Primary Approach: Supportive Care

Most patients recover spontaneously within 2-4 weeks with hydration, pain management (e.g., acetaminophen or ibuprofen), wound care, and treatment of secondary bacterial infections.

Severe cases (e.g., immunocompromised individuals, children, or those with extensive lesions) may require hospitalization for intravenous fluids, antibiotics for superinfections, or ophthalmologic care for eye involvement.

Antivirals Under Investigation

Three antivirals have been studied most extensively, but none are FDA-approved specifically for mpox, and recent trials show limited clinical benefit:

a) Tecovirimat (TPOXX)

Mechanism: Inhibits the viral envelope protein VP37 (F13L), blocking viral spread.

Status: Approved for smallpox (not mpox) in the U.S. and EU; used for mpox under expanded-access protocols.

Efficacy Evidence:

Recent large trials show no significant clinical benefit. A 2025 NEJM study of 597 patients with Clade I mpox in the Democratic Republic of Congo found no difference in lesion resolution time between tecovirimat and placebo (median 7 vs. 8 days; hazard ratio 1.13, p=0.14).

The STOMP trial (2024) for Clade II mpox showed no improvement in clinical resolution (83% vs. 84% at day 29) or pain reduction compared to placebo.

U.S. NIH terminated its tecovirimat trial for mpox in 2024 due to lack of efficacy.

b) Brincidofovir (CMX001)

Mechanism: Lipid-conjugated derivative of cidofovir; inhibits viral DNA polymerase.

Status: FDA-approved for smallpox (2021); limited use for mpox under emergency protocols.

Efficacy Evidence:

Shows strong in vitro activity against mpox (EC50 ~0.08 microM) but lacks robust human trial data.

Associated with gastrointestinal toxicity and liver injury, limiting its utility.

No large randomized trials have confirmed clinical benefit for mpox.

c) Cidofovir

Mechanism: Nucleotide analog that inhibits viral DNA polymerase.

Status: Not FDA-approved for mpox; used off-label.

Efficacy Evidence:

Effective in animal models but nephrotoxic in humans, restricting use to severe cases when alternatives fail.

No conclusive human efficacy data for mpox.

2. Key Limitations of Current Antivirals

Lack of proven clinical impact: Recent trials confirm antivirals do not significantly shorten lesion resolution, reduce pain, or clear viral DNA faster than supportive care alone.

Virus-specific challenges: Efficacy may vary by mpox clade (Clade I in Africa vs. Clade II globally), with Clade I showing higher fatality rates (3.6% vs. <1% for Clade II).

High-risk populations: Immunocompromised patients (e.g., advanced HIV) may derive modest benefit from early antiviral use, but evidence remains anecdotal.

3. Promising Research Directions

Novel Therapeutics in Development

ODE-(S)-HPMPA formate: A Chinese-developed compound showing 40-fold stronger anti-mpox activity in vitro than brincidofovir (EC50 ~40 nM). Currently entering clinical trials.

AI-identified repurposed drugs: Deep learning platforms (e.g., DEEPScreen-Pox) identified HIV drugs like bictegravir as potential broad-spectrum inhibitors of mpox DNA polymerase.

Core protease inhibitors: New inhibitors targeting mpox virus core protease (e.g., compound A3-A6) show nanomolar-range efficacy in preclinical studies.

Critical Gaps

No antiviral has yet met the threshold for routine clinical use in mpox.

Research prioritizes high-risk groups (e.g., immunocompromised patients), where supportive care alone may be insufficient.

Summary

No specific antiviral "cures" mpox, and tecovirimat-the most studied option-lacks robust evidence of clinical benefit in recent trials.

Treatment remains supportive care for most patients, with antivirals reserved for severe or high-risk cases despite unproven efficacy.

Promising next-generation drugs (e.g., ODE-(S)-HPMPA formate) are in development but not yet clinically available.

Vaccination (e.g., JYNNEOS) remains the most effective preventive measure for at-risk populations.

Q29. Can mpox usually get better on its own?

record_id Q29_ChatGPT

question_id Q29

model ChatGPT

answer_text:

Yes. Mpox often gets better on its own, especially in people who are otherwise healthy and not severely immunocompromised. The CDC says most people recover fully in 2 to 4 weeks without special medicines, using supportive care like fluids, nutrition, skin care, and pain/fever relief.

That said, you should contact a healthcare provider if you think you have mpox, because testing, pain control, isolation guidance, and sometimes antiviral treatment may be needed. Some people are at higher risk for severe illness, including people with weakened immune systems, advanced or uncontrolled HIV, pregnancy or recent pregnancy, newborns, and children.

Seek care urgently if you have severe pain, eye symptoms, trouble swallowing, lesions near the eyes/genitals/anus, signs of skin infection, dehydration, confusion, breathing problems, or symptoms that are worsening rather than improving.

record_id Q29_Gemini

question_id Q29

model Gemini

answer_text:

Yes, Mpox Usually Resolves on Its Own

Typical Recovery Timeline: For most individuals, mpox is a self-limiting illness, meaning it typically runs its course and clears up without the need for specialized medical treatment. Most people recover fully within 2 to 4 weeks.

Managing Symptoms: Because the virus usually resolves on its own, care generally focuses on managing symptoms. This includes getting plenty of rest, staying hydrated, and utilizing over-the-counter medications (like ibuprofen or acetaminophen) and topical treatments to help manage pain, fever, and discomfort from the rash.

When Additional Care is Required

While the majority of cases are mild, some individuals are at a higher risk for complications or severe illness and may require medical intervention:

High-Risk Groups: Individuals with severely weakened immune systems (such as advanced or uncontrolled HIV), infants under 1 year old, pregnant or breastfeeding individuals, and people with a history of severe skin conditions like eczema.

Potential Complications: Severe cases can lead to intense pain, secondary bacterial infections on the skin, or lesions in highly sensitive areas (like the eyes, mouth, or genitals). In these instances, healthcare providers may prescribe specialized pain management or antiviral medications.

Next Steps If You Suspect an Infection

Take Action Wisely: If you suspect you or someone you know has been exposed to or infected with mpox, it is highly recommended to speak with a healthcare professional for an official assessment. To protect others, isolate at home and avoid close, skin-to-skin contact with people or pets until all rash lesions have completely scabbed over, fallen off, and a fresh layer of healthy skin has formed.

record_id Q29_Copilot

question_id Q29

model Copilot

answer_text:

Most people with mpox recover without specific antiviral treatment; symptoms usually improve within 2-4 weeks, but some people can develop serious complications and should seek care.

What usually happens

Mpox is often self-limiting. For the common West African/clade II type seen in recent outbreaks, most people have mild illness and recover in about 2-4 weeks with supportive care (rest, fluids, pain control, wound care).

Who is more likely to need treatment

People with weakened immune systems, young children, pregnant people, and newborns are at higher risk of severe disease and complications.

Complications can include secondary bacterial skin infection, pneumonia, encephalitis, severe anorectal or genital inflammation, and eye involvement that can threaten vision.

When to seek medical care now

Seek prompt medical attention if you have mpox and any of the following:

High or worsening fever, severe pain, breathing problems, confusion, vision changes, or signs of a spreading skin infection (increasing redness, warmth, pus).

If you are pregnant, very young, or have advanced HIV or other immune suppression, contact a clinician early because antiviral therapy or hospitalization may be recommended.

Treatments and supportive care

Most care is supportive: pain control, fever reducers, wound hygiene, and treating any bacterial superinfection.

Antiviral medicines (for example, tecovirimat) exist and are used for severe cases or high-risk patients; they are not required for routine mild illness. Decisions about antivirals are made by clinicians based on severity and risk factors.

How long someone is contagious

People can spread mpox from symptom onset until all lesions have crusted, scabs have fallen off, and new skin has formed-typically several weeks. Isolation and careful wound covering reduce transmission.

Practical steps at home

Isolate until lesions are fully healed.

Keep lesions covered, avoid close skin-to-skin contact, and do not share bedding or clothing.

Manage symptoms with pain relief, hydration, and clean dressings; seek care for worsening signs.

Bottom line: Yes - mpox usually gets better on its own in 2-4 weeks for most people, but people at higher risk or with severe symptoms should get medical evaluation because antivirals and supportive hospital care can be needed.

Mpox Johns Hopkins Medicineent

Mpox WHO

Monkeypox FAQs Penn Medicine

Mpox - Prognosis BMJ Best Practice US

record_id Q29_DeepSeek

question_id Q29

model DeepSeek

answer_text:

Generally, yes, mpox is usually a self-limiting illness, and most people get better on their own without needing specific antiviral treatment.

For the majority of people, the infection resolves within a few weeks. Here's what you need to know if you or someone you know gets mpox.

Prognosis: What to Expect for Most People

Recovery Time: The illness usually lasts for 2 to 4 weeks-. The WHO notes that in many cases, symptoms resolve on their own after 14 to 21 days-.

Excellent Recovery Rate: The vast majority of people (over 90%) recover fully without any lasting complications-.

Self-Management: Most people can manage their symptoms at home with rest, fluids, and over-the-counter pain relievers, much like caring for a common cold-. The WHO has a helpful guide with five tips for a safe recovery at home, which you can find on their official website-.

When is Medical Care Necessary?

While mpox is mild for most, it can be more severe and lead to complications for certain high-risk groups. It is crucial to seek medical advice if you or the person who is sick falls into one of these categories:

People with a Weakened Immune System: This includes individuals with advanced HIV, those undergoing cancer treatment, or those who have had an organ transplant-. The illness can be more severe and last longer in these cases-.

Young Children: Children under 5 years old are at a higher risk for a more serious course of the disease-.

Pregnant Individuals: The infection can pose higher risks to both the parent and the baby-.

Severe cases may require hospital care to manage pain, treat secondary infections with antibiotics, and ensure proper hydration-.

Beyond these specific groups, you should also see a healthcare provider if:

The rash becomes very painful or spreads to sensitive areas (like the eyes or genitals)-.

You have trouble swallowing or breathing.

You develop a secondary bacterial infection, which can happen if lesions become severely contaminated.

Symptoms worsen instead of improving after the first few days.

record_id Q29_Doubao

question_id Q29

model Doubao

answer_text:

Most cases of mpox (monkeypox) are self-limiting and can get better on their own.

Key points:

For otherwise healthy adults and older children with mild symptoms

The illness usually resolves on its own within 2-4 weeks without specific antiviral treatment. Symptoms like fever, rash, blisters, and swollen lymph nodes will gradually fade, scab over, and heal naturally.

High-risk groups cannot rely on self-recovery

People at higher risk may develop severe complications and need medical treatment instead of waiting to heal on their own:

Infants and young children

Pregnant people

Those with weakened immune systems (e.g., HIV infection, long-term use of immunosuppressants)

People with severe underlying diseases

Even if it heals itself

Isolation is still required to avoid spreading the virus to others. Scratching the rash should be avoided to prevent secondary bacterial infection and scarring.

record_id Q29_Qwen

question_id Q29

model Qwen

answer_text:

Yes, mpox is typically a self-limiting disease that resolves on its own in most immunocompetent individuals within 2-4 weeks, without specific antiviral treatment. However, severe or fatal outcomes can occur in high-risk groups, such as those with weakened immune systems, young children, or pregnant individuals. Below is a detailed breakdown:

1. General Course and Self-Limiting Nature

Most cases are mild and self-resolving: The majority of mpox infections follow a self-limiting course, with symptoms (e.g., fever, rash, lymph node swelling) gradually improving over 2-4 weeks. No specific antiviral therapy is required for typical cases, and management focuses on symptomatic relief (e.g., pain control, hydration, and wound care) .

Viral clearance: The immune system typically clears the virus without long-term complications in healthy individuals. Skin lesions progress through stages (macules to papules to vesicles to pustules to crusts) and heal completely, though scarring may persist for months .

2. High-Risk Groups Requiring Medical Attention

While mpox usually resolves independently, certain populations face higher risks of severe disease or complications:

Immunocompromised individuals: Those with advanced HIV (especially CD4 counts <50 cells/microL), untreated AIDS, or other immunosuppressive conditions are at significantly elevated risk of prolonged illness, secondary infections (e.g., bacterial sepsis), or death. For example, a 2025 case report documented fatal mpox in a 23-year-old HIV-positive patient with a CD4 count of 8 cells/microL .

Children and pregnant individuals: Higher rates of severe complications (e.g., pneumonia, encephalitis) and mortality are observed in these groups.

Severe complications: These may include secondary bacterial infections, bronchopneumonia, sepsis, or vision-threatening corneal involvement, necessitating hospitalization and targeted interventions .

3. Treatment and Prevention

No specific antivirals: There are no universally approved antiviral drugs for mpox. Tecovirimat (TPOXX) may be used off-label in severe cases, but evidence remains limited. Most care is supportive (e.g., pain management, hydration, and preventing secondary infections) .

Prevention:

Vaccination: Prior smallpox vaccination (e.g., ACAM2000 or MVA-BN) offers partial cross-protection, though immunity wanes over time.

Early isolation: Infected individuals remain contagious until all scabs fall off and new skin forms, typically 2-4 weeks.

High-risk groups: Immunocompromised individuals should prioritize HIV treatment (ART) to maintain CD4 counts >200 cells/microL, drastically reducing severe mpox risks .

In summary, mpox usually resolves spontaneously in healthy people, but vigilance is critical for vulnerable populations. If symptoms worsen (e.g., difficulty swallowing, high fever, or widespread skin breakdown), prompt medical evaluation is essential to manage complications.

1. - 2. ?,- 3. ?-

4. , - 5.,,- 6. 0.1%,-

Q30. Can I take painkillers if my mpox rash is very painful?

record_id Q30_ChatGPT

question_id Q30

model ChatGPT

answer_text:

Yes. For many people with mpox, over-the-counter painkillers such as acetaminophen/paracetamol or ibuprofen can help with painful rash symptoms, and CDC guidance specifically lists these as options for mpox pain control.

Because you said the rash is very painful, you should contact a healthcare provider promptly, especially if home pain relief is not enough. CDC advises seeking care if pain becomes severe or unmanageable at home; prescription pain relief or other treatment may be needed.

Use painkillers safely:

Acetaminophen/paracetamol: Follow the label exactly, and do not combine multiple products that contain it. Too much can cause severe liver damage; FDA lists 4,000 mg in 24 hours as the adult maximum for people age 12+ and advises extra caution with liver disease or heavy alcohol use.

Ibuprofen: Take only as directed and preferably with food. Avoid or ask a clinician/pharmacist first if you have a stomach ulcer, kidney/heart/liver disease, blood-clotting problems, asthma/allergy to NSAIDs, are pregnant/trying to get pregnant, or take blood thinners, steroids, some antidepressants, or blood-pressure medicines.

Also seek urgent medical advice if the rash is near/in your eyes, you have trouble urinating, severe rectal/genital pain, pus/spreading redness/warmth, trouble swallowing, dehydration, confusion, or you are pregnant, immunocompromised, or have a significant skin condition. CDC notes some higher-risk or severe cases may need specific mpox treatments.

record_id Q30_Gemini

question_id Q30

model Gemini

answer_text:

Yes, you can take painkillers to help manage the discomfort. Mpox rashes can be notoriously painful, and managing that pain is a core part of taking care of yourself while you recover.

Here is a breakdown of over-the-counter (OTC) options and strategies recommended by public health guidelines:

Over-the-Counter Pain Relievers

Systemic Painkillers: Medications like acetaminophen (Tylenol) or non-steroidal anti-inflammatory drugs (NSAIDs) such as ibuprofen (Advil, Motrin) and naproxen (Aleve) can help reduce general pain and lower any accompanying fever.

Topical and Local Relief

Numbing Gels: Over-the-counter topical anesthetics containing lidocaine or benzocaine can offer temporary, localized relief. Note: Use these cautiously on broken skin or open, draining wounds, and always wear disposable gloves when applying them to prevent spreading the virus to other parts of your body.

Soothe the Skin: Calamine lotion or petroleum jelly (Vaseline) can help protect the skin and manage itching as the lesions heal.

Soaking: If the rash is in the genital or anal area, sitting in a warm sitz bath for 10 to 15 minutes a few times a day can significantly ease the inflammation. Adding baking soda or Epsom salt to the water can be especially soothing.

Mouth Lesions: If you have painful sores in your mouth, frequent rinses with clean saltwater can help keep them clean and ease discomfort.

When to Contact Your Doctor

Mpox pain can sometimes become severe enough to require professional medical management. You should reach out to your healthcare provider if:

Over-the-counter medications are not making the pain manageable.

The pain is preventing you from sleeping, eating, drinking fluids, or using the bathroom.

You notice signs of a secondary bacterial infection, such as rapidly spreading redness, swelling, warmth, or pus around the sores.

A doctor can evaluate your symptoms and may prescribe stronger prescription pain relievers, specialized numbing rinses, or an antiviral treatment like tecovirimat (TPOXX) if you qualify.

record_id Q30_Copilot

question_id Q30

model Copilot

answer_text:

Short answer: Yes - many people with mpox use over-the-counter painkillers (acetaminophen or NSAIDs) and topical analgesics for lesion pain, but you should check with a clinician before starting stronger or combined medicines and seek urgent care for severe or worsening pain.

Painkillers and mpox

What is commonly used

Mild-moderate pain: Acetaminophen (paracetamol) or nonsteroidal anti-inflammatory drugs (NSAIDs) are commonly recommended as first-line systemic options for mpox-related pain.

Topical measures: Topical analgesics, local wound care, and topical anesthetics (for mouth or genital lesions) are part of routine symptomatic care.

Severe pain: Some patients require stronger prescription analgesics (including short courses of opioids) or specialist pain management, especially for severe anogenital or oropharyngeal lesions. Hospital admission for pain control has been reported.

Important safety points (do not skip)

Talk to a healthcare professional before combining medicines (for example, taking NSAIDs plus other prescription pain drugs) because interactions, allergies, or underlying conditions change what's safe for you.

Do not exceed label doses on over-the-counter products; I cannot give personalized dosing here - your clinician or pharmacist can advise what's safe for your age, weight, liver/kidney function, and other medicines.

Watch for signs of secondary bacterial infection (increasing redness, warmth, pus, spreading pain) - these may need antibiotics or procedural care.

When to seek urgent care

Severe, uncontrolled pain despite OTC medicines.

Inability to eat, drink, urinate, or breathe because of lesions (especially mouth, throat, or perineal lesions).

High fever, spreading redness, or signs of sepsis.

Other treatments your clinician may consider

Antiviral therapy (e.g., tecovirimat/TPOXX) is reserved for certain patients with severe disease or high risk of complications and may be considered when pain is part of severe manifestations.

Multimodal pain strategies (topical + systemic + specialist input) are often needed for mucosal or anogenital pain.

Bottom line: Using common painkillers and topical analgesics is an appropriate first step for mpox lesion pain, but consult a healthcare provider before escalating therapy or if pain is severe or worsening. If your pain is intense or you have red flags, seek urgent medical care.

record_id Q30_DeepSeek

question_id Q30

model DeepSeek

answer_text:

Mpox-related pain can be intense, but there are proven ways to manage it. Yes, you can take specific over-the-counter painkillers, and your doctor can prescribe stronger ones if needed.

Here's a quick overview of the pain relief options available:

Method Options Key Notes

Oral Painkillers Acetaminophen (Tylenol), NSAIDs like Ibuprofen (Advil, Motrin)- First-line for general, mild-to-moderate pain

Topical Treatments Lidocaine, benzocaine gels/creams- Apply only to intact skin, wearing gloves-1. Provides local relief.

Prescription Options Gabapentin, opioids (e.g., oxycodone)--6 For severe, persistent, or neuropathic pain. Used short-term under strict medical guidance

Oral Care Saltwater rinses, viscous lidocaine-4 For painful mouth or throat sores.

Soothing Soaks Warm baths with oatmeal, baking soda, or Epsom salts-4-18 Helps soothe itching and discomfort from body sores.

When to Seek Medical Help

While you can manage many symptoms at home, you should contact your doctor if:

Pain becomes severe and unmanageable

Your rash shows signs of infection, such as increased redness, swelling, or pus

You develop a fever or your pain increases significantly

I hope this information is helpful. It is important to remember that this is for informational purposes, and you should consult with a healthcare professional for personalized medical advice.

record_id Q30_Doubao

question_id Q30

model Doubao

answer_text:

Yes, you can take over-the-counter painkillers to relieve significant pain from mpox (monkeypox) rash, following safe guidelines:

Safe pain relief options

Paracetamol (acetaminophen): First choice for easing rash pain, soreness and fever.

Ibuprofen: Works for pain plus reducing inflammation from the rash.

Critical precautions

Always follow the recommended dose on the packaging; do not take more than the maximum daily amount.

Avoid aspirin, especially in children and teenagers-it carries a risk of Reye's syndrome.

Do not combine multiple painkillers randomly without medical advice.

If you have liver/kidney problems, stomach ulcers, or medication allergies, consult a doctor first before taking any painkillers.

If the pain is unbearable, the rash becomes infected (pus, severe redness), or you have a high persistent fever, see a healthcare provider right away-do not only rely on painkillers.

You can also ease discomfort by keeping the rash clean and dry, wearing loose soft clothing, and avoiding scratching the lesions. It's still best to consult a healthcare professional for personalized advice if you have confirmed mpox.

record_id Q30_Qwen

question_id Q30

model Qwen

answer_text:

Yes, you can take painkillers for mpox (monkeypox)-related pain, as pain management is a standard part of supportive care for the condition. However, the choice of painkiller depends on the severity of pain, your health status, and medical guidance. Below is a structured overview:

1. Recommended Painkillers for Mpox

a) Mild to Moderate Pain

Non-steroidal anti-inflammatory drugs (NSAIDs) like ibuprofen or acetaminophen (paracetamol) are commonly recommended to reduce pain and fever. These help alleviate discomfort from skin lesions, headaches, or muscle aches.

Topical options such as lidocaine gel or 5% lidocaine patches may be applied directly to painful rash sites (e.g., genital or anal areas) to numb localized pain without systemic side effects.

b) Severe Pain

For intense pain (e.g., from mucosal lesions, swollen lymph nodes, or deep skin ulcers), tramadol or short-term opioids (e.g., morphine) may be prescribed under medical supervision. These are reserved for cases where NSAIDs are insufficient.

Avoid self-medicating with strong opioids, as they carry risks of dependence, respiratory depression, or gastrointestinal issues.

2. Key Considerations

a) Safety and Medical Guidance

Always consult a healthcare provider before taking painkillers, especially if you:

Have kidney, liver, or gastrointestinal conditions (NSAIDs can worsen these).

Are pregnant, immunocompromised, or taking other medications (e.g., anticoagulants).

Experience severe symptoms like high fever, difficulty swallowing, or signs of secondary infection (e.g., pus, redness, swelling).

b) Complementary Measures

Avoid scratching lesions, as this can cause secondary bacterial infections. Wear loose clothing and use mittens or gloves at night if needed.

For mucosal pain (e.g., mouth or rectal sores), adjunctive care like saline rinses, stool softeners, or sitz baths may help.

Topical antibiotics (e.g., mupirocin) are only used if bacterial infection occurs-not preventively.

3. What to Avoid

Do not use topical anesthetics containing benzocaine (e.g., over-the-counter numbing creams), as they may cause allergic reactions or worsen skin irritation.

Avoid ice directly on lesions, as extreme cold can damage already compromised skin.

Never pop blisters, as this increases infection risk and delays healing.

Mpox is typically self-limiting, with most cases resolving in 2-4 weeks. Pain management focuses on improving comfort while lesions heal, but antiviral drugs (e.g., tecovirimat) are reserved for severe/high-risk cases and require prescription. If pain persists or worsens, seek medical evaluation to rule out complications like bacterial superinfection or nerve involvement. Always prioritize professional medical advice over self-treatment.

Q31. How should I handle clothes and bedsheets while isolating at home with mpox?

record_id Q31_ChatGPT

question_id Q31

model ChatGPT

answer_text:

While isolating with mpox, treat clothes, towels, and bedsheets as potentially contaminated until your rash has fully healed, scabs have fallen off, and new skin has formed; CDC says this often takes 2-4 weeks.

For laundry:

Keep your laundry separate. Do not mix your clothing, bedding, towels, or washcloths with anyone else's laundry.

Bag items carefully. Put used clothes/linens in a waterproof bag/container that can be disinfected or thrown away, or in a washable fabric laundry bag that goes into the washer with the items.

Do not shake sheets, towels, or clothes. Shaking can spread contaminated particles into the air.

Wash in a regular washing machine with detergent. Follow the detergent/fabric label instructions. Laundry sanitizer is optional, not required.

Handle your own laundry if you can. If someone else must do it, they should avoid shaking it and wear disposable gloves, a well-fitting mask or respirator, and clothing that covers arms and legs.

Do not share bedding, towels, clothing, or washcloths with anyone else. CDC also says not to share other potentially contaminated items like cups or utensils.

Wash hands after handling laundry, especially after touching rash material, clothes, linens, or surfaces that may have contacted lesions.

For bedsheets and mattresses: sleep alone if possible, keep lesions covered, and use washable sheets or a protective cover over surfaces that cannot easily be laundered. CDC recommends avoiding contamination of porous items like upholstered furniture or mattresses by using coversheets, waterproof mattress covers, blankets, or tarps.

If you do not have laundry at home: CDC says, if possible, wait until isolation ends before washing outside the home. If laundry must be done elsewhere, you can do it yourself while following precautions, or a helper can do it with gloves, a mask/respirator, and covered skin. Do not send contaminated clothes for dry cleaning; coordinate with public health and the facility before using commercial laundry services.

Call your healthcare provider or local health department for advice if bedding or a mattress is heavily contaminated with drainage from lesions, or if you cannot safely manage laundry at home.

record_id Q31_Gemini

question_id Q31

model Gemini

answer_text:

Handling laundry carefully is one of the most critical steps to prevent the virus from spreading to others in your home or contaminating your living space.

According to guidelines from the Centers for Disease Control and Prevention (CDC), here is the safest way to manage your clothes, towels, and bedsheets during isolation:

Essential Handling Rules

Never shake the laundry: This is the most crucial rule. Shaking sheets, blankets, or clothing can launch infectious virus particles into the air, where they can be inhaled or settle onto other clean surfaces.

Keep it strictly separate: Do not mix your contaminated laundry with the items of anyone else in your household.

Contain it immediately: Put your dirty items directly into a fabric laundry bag (which can be thrown right into the washing machine with the clothes) or a plastic trash bag/waterproof bin that you can easily discard or disinfect afterward.

Gather laundry first: If you are cleaning your isolation area, always gather and bag your dirty laundry before you start wiping down hard surfaces or vacuuming.

Washing and Drying Guidelines

Standard Machines Work: You do not need specialized equipment. A standard home washer and dryer are perfectly capable of destroying the virus.

Use Hot Water: Wash the items using your regular laundry detergent. Opt for the hot water cycle or the highest temperature setting recommended on the fabric care labels.

Dry on High Heat: Dry the clean laundry completely in a standard dryer on the highest temperature setting allowed for those fabrics.

Sanitizers Are Optional: You can add a laundry sanitizer if it gives you peace of mind, but standard detergent and high heat are fully effective on their own. Do not take items to a dry cleaner.

If Someone Else is Helping You

Ideally, you should handle and wash your own laundry. However, if you are too unwell and a caregiver or housemate needs to assist, they must protect themselves:

Wear Personal Protective Equipment (PPE): At a minimum, they should wear disposable medical gloves, a well-fitting mask or respirator (like an N95), and clothing that completely covers their arms and legs.

Immediate Cleanup: They must avoid extensive contact with the items, immediately launder the clothes they wore while handling your bedding, and wash their hands thoroughly with soap and water as soon as they finish.

Are you isolating entirely alone, or are there other people or pets in the household you need to share spaces with?

record_id Q31_Copilot

question_id Q31

model Copilot

answer_text:

Keep your laundry separate, handle it gently (don't shake), wash in the hottest setting allowed with regular detergent, dry on high, and clean/disinfect surfaces and laundry baskets after - and ask a clinician or local health department if you're unsure.

Isolation laundry essentials

Who should handle laundry: If possible, you should do your own laundry while isolating. If someone else must help, they should wear gloves and a mask and avoid direct contact with lesions.

Avoid shaking or brushing items to reduce the chance of dispersing infectious material. Gently place items into the washer.

Washing and drying

Washer settings: Use hot water or the highest temperature allowed for the fabric and a normal amount of detergent. You do not need special disinfecting detergents or household bleach for routine loads.

Drying: Dry on the highest heat setting allowed by the garment label; heat helps inactivate virus particles. For items that cannot be machine-dried, air-dry at home.

Handling non-machine-washable items and small personal items

Hand-washable items: Wash in a sink or tub with detergent and hot water; rinse and dry thoroughly.

Items that can't be cleaned: If you cannot wash or disinfect an item (e.g., some leather goods, hats), seal it in a plastic bag for 21 days to allow any virus on surfaces to become noninfectious, or follow local public-health advice.

Protecting surfaces and people

Disinfect laundry containers and surfaces (washer/dryer knobs, laundry baskets, folding surfaces) with an EPA-registered disinfectant effective against emerging viral pathogens or follow product label instructions.

Hand hygiene: Wash hands with soap and water immediately after handling soiled laundry; use alcohol-based hand sanitizer if soap/water are not available.

If you use a shared laundromat

Minimize contact: Limit time in shared spaces, avoid close contact with others, and carry laundry in a washable bag. Remove clothes directly into the washer and put dry clothes back into the clean bag before leaving. Fold at home if possible.

When to ask for more help

Talk to a healthcare provider or local public health authority if you have extensive lesions, heavy drainage, or questions about household cleaning; they can give guidance tailored to your situation. CDC and WHO guidance are available for household infection control.

Quick checklist

Do your own laundry if possible; wear gloves if someone else helps.

Don't shake items; use hot water and regular detergent; dry on high.

Disinfect baskets and surfaces; wash hands after handling.

How to Safely Wash Your Laundry if You Have Mpox

Disinfectants for Emerging Viral Pathogens (EVPs): List Q

record_id Q31_DeepSeek

question_id Q31

model DeepSeek

answer_text:

If you have mpox and are isolating at home, the most important thing is to think of your clothing and bedsheets as potentially infectious. Mpox spreads through direct contact with the rash or by touching contaminated items like these--3. To prevent the virus from spreading to others, you'll need to handle, launder, and store your laundry with care.

Here is the step-by-step guidance you should follow:

A Step-by-Step Guide to Handling Your Laundry

Keep it Separate: Do not share your clothes, bedding, towels, or washcloths with anyone else, including family members--3-25. Your laundry should be washed completely separately from everyone else's.

Wear Disposable Gloves: Always put on disposable gloves before you touch any of your dirty laundry. If you have a rash on your hands, wearing gloves is even more critical-

Roll, Don't Shake: Fold or gently roll up your used clothes and linens. Never shake them out. Shaking can release virus particles into the air and spread the infection-.

Use a Dedicated Laundry Bag: Place all rolled-up laundry directly into a plastic bag or a dedicated laundry bin that won't be used by anyone else. This helps contain any virus on the fabric.

The best approach is to handle your laundry yourself-. If someone else must do it, they should also wear disposable gloves and a well-fitting mask-.

How to Wash Your Clothing and Linens

Use the Hottest Water Possible: Wash the items in a washing machine with regular laundry detergent, using the warmest appropriate water setting. A water temperature of at least 60 degrees C (140 degrees F) is highly recommended to effectively kill the virus-.

Add Household Bleach if Safe: For an extra precaution, you can add a disinfectant like household bleach to the wash, but always check the item's care label to avoid damaging your clothes-.

Dry Thoroughly on High Heat: After washing, dry the items completely on the highest heat setting suitable for the fabric. The combination of high temperatures from washing and drying is very effective at inactivating the virus-.

Cleaning and Disinfecting Your Space

In addition to laundry, you should also clean and disinfect surfaces you touch often, like doorknobs, light switches, and bathroom counters-3-25. The virus can survive on household surfaces for at least 15 days, so regular cleaning is important to reduce the risk of lingering contamination-.

For more detailed guidance on the overall cleaning process, including specific product recommendations, you can refer to the CDC's page on Cleaning and Disinfecting Your Home.

How long do I need to do this for?

You should continue these precautions until your mpox rash has fully healed-that is, all the scabs have fallen off and a new layer of intact skin has formed underneath, which usually takes 2 to 4 weeks

record_id Q31_Doubao

question_id Q31

model Doubao

answer_text:

How to Handle Clothes & Bed Sheets During Mpox Home Isolation

Mpox spreads easily through direct contact with contaminated clothing, bed linens, body fluids and skin lesions. Follow these strict, safe guidelines to avoid spreading the virus to household members:

1. Basic Isolation Rule

Keep all your clothes, bed sheets, quilts, towels and pajamas completely separate from other family members at all times.

Never share bedding, pillows, blankets, bath towels or personal clothing with anyone.

2. Disposal of Dirty Used Items

Change bed sheets, pillowcases daily (or every 1-2 days); change undergarments and daily clothes every day.

Place dirty clothes/bed sheets immediately into a lidded dedicated laundry basket or sealed plastic bag - do not leave them on chairs, sofas or floors.

Do not shake dirty linen/clothes to prevent virus-laden dander and droplets from spreading in the air.

3. Washing Requirements

Wear disposable gloves and a mask when handling dirty laundry.

Wash separately from family's clothes only in your own dedicated washing cycle; never mix laundry.

Use hot water (>=60 degrees C) for machine washing - high temperature effectively inactivates the mpox virus.

Add regular laundry detergent, or a clothing-safe disinfectant (follow product dilution instructions).

Avoid hand-washing heavily soiled bed sheets/clothes to prevent direct skin contact with contaminants.

4. Disinfection & Drying

After washing, dry all items completely in direct sunlight for at least 4 hours; UV sunlight kills residual virus.

For fabrics that cannot withstand high heat: soak in diluted household disinfectant for 15-20 minutes before rinsing.

Disinfect your dirty laundry basket and storage bag regularly with chlorine-based disinfectant wipes.

5. Storage After Cleaning

Store fully dried clothes and bed linen in your separate private cabinet/area, away from family's personal items.

6. Post-Recovery Final Disinfection

After you recover and finish isolation:

Re-wash all your used bedding and clothes with hot water again, then sun-dry thoroughly before regular use.

Disinfect your bedroom, mattress and laundry storage area completely.

7. Personal Protection

Always wash your hands thoroughly with soap and running water after handling laundry or bed sheets.

Discard used disposable gloves in a sealed trash bag immediately after use.

record_id Q31_Qwen

question_id Q31

model Qwen

answer_text:

Monkeypox (mpox) patients can safely handle their own laundry at home if they are physically able, but contaminated clothing and bedding must be washed using hot water (>=60 degrees C/140 degrees F) with detergent or disinfected with appropriate chemical methods to inactivate the virus. Avoid shaking items to prevent dispersing viral particles, and always practice strict hand hygiene after handling.

1. Key Handling Principles

Critical Safety Measures

Do not shake or (agitate) contaminated items, as this may release virus particles into the air.

Wear disposable gloves when handling soiled laundry if someone else must manage it (e.g., if the patient is too ill).

Immediately wash hands with soap and water for at least 20 seconds after contact with contaminated items.

2. Step-by-Step Laundry Protocol

For Patients Able to Self-Manage

Pre-treatment (if needed):

For visible contaminants (e.g., pus, blood), gently soak items in 60 degrees C+ water with detergent before washing. Avoid scrubbing, which may spread viral material.

If using chemical disinfectants, soak in 500mg/L chlorine solution (e.g., diluted bleach) for 30 minutes, then rinse thoroughly before regular washing.

Washing Method:

Hot water cycle (>=60 degrees C/140 degrees F) is essential to inactivate the virus. Standard detergents are sufficient.

If hot water is unavailable, use chlorine-based disinfectants (follow product instructions) or quaternary ammonium compounds during soaking.

Drying and Storage:

Dry items completely in direct sunlight or using a high-heat dryer setting, as UV light and heat further reduce viral survival.

Store cleaned items in a sealed bag until fully dry to avoid recontamination.

For Caregivers Handling Laundry

Wear disposable gloves, a medical mask, and an apron if assisting the patient.

Place soiled items directly into a leak-proof plastic bag before transport to the washing machine.

Disinfect the washing machine drum afterward with a chlorine-based cleaner.

3. Special Considerations

Non-Washable Items

Items that cannot withstand hot water (e.g., delicate fabrics) should be:

Sealed in a plastic bag for >=3 weeks (virus degrades over time in dry environments).

Alternatively, treated with 70% alcohol spray (ensure full coverage and air-dry completely).

High-Risk Situations

If lesions have ruptured or leaked onto fabrics, treat items as potentially infectious medical waste. Consult local health authorities for disposal guidelines.

Never share unwashed clothing, towels, or bedding with others until fully cleaned.

4. Why These Steps Matter

Monkeypox virus survives for weeks to months on fabrics in cool, dry conditions but is inactivated by heat (60 degrees C for 10 minutes) or standard disinfectants.

Improper handling (e.g., shaking laundry) risks aerosolizing viral particles, increasing transmission risk to others.

Following these protocols reduces household transmission risk while allowing safe reuse of textiles.

If symptoms worsen or you're unsure about managing laundry, contact a healthcare provider for personalized guidance. Always prioritize physical isolation and hand hygiene alongside laundry safety.

Q32. Is there a vaccine that can prevent mpox?

record_id Q32_ChatGPT

question_id Q32

model ChatGPT

answer_text:

Yes. JYNNEOS is the main vaccine used to help prevent mpox. It is a 2-dose vaccine, with the second dose recommended 4 weeks after the first; getting both doses gives the best protection.

In the U.S., JYNNEOS and ACAM2000 are approved to prevent mpox, but CDC says only JYNNEOS has been used in recent outbreaks. ACAM2000 has more restrictions because of potential side effects.

It is not 100% protective, so vaccinated people can still get mpox, but vaccination lowers the chance of infection and may reduce severity. CDC cites effectiveness estimates of about 75% after one dose and 86% after two doses in a multijurisdictional study.

It can also be used after exposure: CDC says it should be given as soon as possible, ideally within 4 days of exposure; giving it 4-14 days after exposure may still provide some protection.

CDC does not recommend routine mpox vaccination for everyone; it is targeted to people at higher risk or after certain exposures. A healthcare provider or local health department can help decide whether you are eligible.

record_id Q32_Gemini

question_id Q32

model Gemini

answer_text:

Yes, there are vaccines available that can effectively prevent mpox. The primary vaccine used globally is JYNNEOS, though other options exist depending on a person's health status and vaccine availability.

JYNNEOS (also known internationally as Imvamune or Imvanex) is the primary vaccine used for mpox prevention.

Vaccine Type: It is a third-generation, live vaccine containing Modified Vaccinia Ankara (MVA)-a virus from the same family as mpox and smallpox.

How it Works: The virus in JYNNEOS is non-replicating, meaning it cannot multiply inside the human body. This makes it significantly safer than older smallpox vaccines.

Dosing Schedule: It is administered as a two-dose series, given 4 weeks (28 days) apart. Full immunity is typically achieved about 14 days after the second dose.

Safety Profile: Because it is non-replicating, it can be safely given to individuals who are immunocompromised (such as people living with HIV) or those with skin conditions like eczema.

2. ACAM2000 (Alternative Vaccine)

ACAM2000 is an older, second-generation vaccine originally developed for smallpox that has been authorized for use against mpox under specific protocols.

Vaccine Type: It contains a live, replicating vaccinia virus.

Dosing Schedule: It is given as a single dose using a specialized, multiple-puncture needle technique that creates a small lesion (or "take") at the injection site.

Considerations: Because the virus replicates, ACAM2000 carries a higher risk of adverse side effects. It is strictly contraindicated for individuals who are pregnant, immunocompromised, or have active skin conditions like eczema or psoriasis.

Who is Recommended to Get Vaccinated?

Public health agencies (such as the CDC and WHO) do not currently recommend mass, population-wide vaccination for mpox. Instead, vaccination strategies focus on individuals at higher risk of exposure:

Post-Exposure Prophylaxis (PEP): People who have had known or suspected close contact with someone diagnosed with mpox. Ideally, the vaccine should be given within 4 days of exposure to prevent onset, or within 14 days to reduce symptom severity.

Pre-Exposure Prophylaxis (PrEP): Individuals with certain behavioral or occupational risk factors, including laboratory personnel working with orthopoxviruses, or individuals with multiple or anonymous sexual partners in regions experiencing active outbreaks.

record_id Q32_Copilot

question_id Q32

model Copilot

answer_text:

Yes. Two vaccines can prevent mpox; the one most commonly used and recommended today is JYNNEOS, which is safer and the primary vaccine for most people at risk.

Vaccine overview

JYNNEOS (also called Imvamune or Imvanex) is a non-replicating live vaccine approved and recommended for prevention of mpox and smallpox; it is the vaccine most widely used in recent outbreaks. ACAM2000 is an older, replicating vaccinia vaccine that can prevent mpox but has more side effects and is used only in special circumstances.

Quick comparison table

Type Typical use Safety profile

JYNNEOS Live non-replicating MVA Primary vaccine for at-risk adults; 2 doses Lower risk of serious adverse events; suitable for more people.

ACAM2000 Live replicating vaccinia Backup or special use; available under protocols Higher risk (skin lesion transmission; heart inflammation risk).

How well the vaccine works

JYNNEOS is given as two doses about 4 weeks apart. Protection increases after the second dose and is considered most complete about two weeks after dose two. One dose provides partial protection; two doses give substantially better protection.

Observational data from the 2022 outbreak estimated ~68% effectiveness after one dose and ~89% after two doses against diagnosed infection in one study, underscoring the value of completing the series.

Who should get vaccinated and when

Vaccination is recommended for people at higher risk of exposure, not the general population. This includes people with recent exposure to mpox, and people with sexual or occupational risk factors identified by public health guidance (for example, certain men who have sex with men, partners of at-risk people, and some laboratory workers). Post-exposure vaccination can be effective if given promptly.

Risks, limitations, and practical steps

Risks: ACAM2000 carries higher risks (cardiac inflammation, autoinoculation, transmission from the vaccination site). JYNNEOS has a more favorable safety profile but may cause local or systemic reactions.

Limitations: No vaccine is 100% protective; breakthrough infections can occur. Full protection takes time after vaccination.

Actionable steps: If you think you're at risk or were exposed, contact your local health department or healthcare provider to ask about JYNNEOS vaccination and timing for post-exposure prophylaxis.

Mpox FAD - U.S.Food and Drug

What Is the Mpox Vaccine and How Does It Work? tment Scienceinsights

JYNNEOS(Smallpox and Monkeypox Vaccline,Live,Non-replicating)

record_id Q32_DeepSeek

question_id Q32

model DeepSeek

answer_text:

Yes, effective vaccines are available to prevent mpox. These vaccines were originally developed for smallpox, as the viruses are closely related, and they provide strong cross-protection.

Currently, there are three main vaccines in use globally, with the MVA-BN vaccine serving as the primary choice for most vaccination campaigns today

The Leading Option: MVA-BN (JYNNEOS / Imvanex)

This is the most widely used mpox vaccine due to its strong safety and effectiveness profile.

Vaccine Type: A third-generation, live, non-replicating vaccine-6-10. This means it cannot cause disease or reproduce in the human body, making it incredibly safe, including for people with weakened immune systems like those with HIV

Brand Names: Sold under the names JYNNEOS (in the US) and Imvanex (in Europe).

Real-World Effectiveness: Studies show two doses offer very strong protection, with effectiveness estimated between 66% and 90%--6. A single dose provides a quicker, albeit lower and potentially shorter-lived level of protection-

Global Authority: It is the first mpox vaccine to receive prequalification from the World Health Organization (WHO), a key step to enable access in lower-income countries

Other Available Vaccines

Two other vaccines are also available, typically in specific situations or regions:

ACAM2000: A second-generation vaccine containing a live, replicating virus-10. While highly effective, it has a significantly higher risk of serious side effects (including heart inflammation) and is generally reserved for situations where MVA-BN cannot be used-

LC16m8: A third-generation vaccine used primarily in Japan and recommended for high-risk groups in affected regions-

What's on the Horizon?

Efforts are ongoing to increase the global supply of vaccines:

GEO-MVA (GeoVax): A next-generation MVA-based vaccine being developed in the U.S., which could help diversify the global supply-

Other candidates: Several other vaccines are in development, including MVA1974-, TNX-801-, and OrthopoxVac-26, among others.

Who Should Get Vaccinated & How (US CDC Guidelines)

In the United States, the CDC officially recommends the 2-dose JYNNEOS vaccine for certain groups--13. If you think you might be at risk, talk to your healthcare provider.

The table below outlines the CDC's key vaccination recommendations for the JYNNEOS vaccine:

Context Recommendation Details

Before Exposure The CDC recommends it for people 18 and older who may be at risk for mpox infection-. This includes: gay, bisexual, and other men who have sex with men (MSM) who have had multiple or new sexual partners in the last 6 months; individuals with a new STI diagnosis; people with sexual partners at risk; and those who anticipate any of these activities

After Exposure For post-exposure prophylaxis (PEP), the vaccine should be given within 4 days of the most recent exposure for the best chance to prevent the disease. It may help reduce symptoms if given within 4 to 14 days after exposure-.

Schedule The vaccine is given as a 2-dose series, spaced 28 days apart--14. For international travelers anticipating sexual activity in countries with ongoing transmission (including Clade I), the CDC recommends starting the vaccination series at least six weeks before travel-.

In summary, the MVA-BN (JYNNEOS/Imvanex) vaccine is a safe and effective tool for preventing mpox. If you believe you or someone you know may be at risk, consulting with a healthcare provider or local public health authority is the best way to get personalized advice on vaccination.

record_id Q32_Doubao

question_id Q32

model Doubao

answer_text:

Yes, there are approved vaccines to prevent mpox (monkeypox). The most widely used and recommended vaccine globally is JYNNEOS (also known as Imvanex, Imvamune, or MVA-BN), with additional options including ACAM2000 and LC16m8World Health Organization (WHO).

Available Mpox Vaccines

Vaccine Name Alternative Names Type Key Features

JYNNEOS Imvanex, Imvamune, MVA-BN Live, non-replicating vaccinia virus FDA-approved for >=18 years (>=12 in EU)U.S. Food and Drug Administration Two-dose series (4 weeks apart) Primary vaccine used in recent outbreaks WHO-prequalified

ACAM2000 - Live, replicating vaccinia virus FDA-approved for smallpox/mpox preventionU.S. Food and Drug Administration Single-dose Not preferred due to higher risk profile

LC16m8 - Live, attenuated vaccinia virus Used in some countries Provides good protection when fully vaccinated

Vaccine Effectiveness

One dose JYNNEOS: 35-86% effective against mpox infection

Two doses JYNNEOS: 66-90% effective against mpox infection

Vaccination also reduces symptom severity in breakthrough infections

Who Should Get Vaccinated

Vaccination is recommended for:

People at high risk of mpox exposure (e.g., certain sexual behaviors, close contacts of cases)

Healthcare and public health workers at risk of occupational exposure

Laboratory personnel working with mpox virus

Important Notes

Mpox vaccines are not 100% effective, but significantly reduce infection risk and severity

JYNNEOS is the preferred vaccine due to its favorable safety profile compared to ACAM2000World Health Organization (WHO)

Availability varies by country - check with local health authorities for access

For the most current guidance, consult your national public health agency (e.g., CDC in the U.S., WHO globally).

record_id Q32_Qwen

question_id Q32

model Qwen

answer_text:

Yes, several vaccines can prevent mpox (monkeypox), though none are specifically designed only for mpox. Most are repurposed smallpox vaccines that provide cross-protection due to the genetic similarity between mpox and smallpox viruses (both belong to the Orthopoxvirus genus). The most widely used and WHO-prequalified vaccine is MVA-BN (JYNNEOS/Imvanex), with others like ACAM2000 and LC16m8 also approved in specific regions. However, no mpox-specific vaccine is yet universally available for mass public use, and recommendations prioritize high-risk groups over general populations.

1. Approved Vaccines for Mpox Prevention

a) MVA-BN (JYNNEOS/Imvanex)

A non-replicating modified vaccinia Ankara (MVA) strain vaccine, originally developed for smallpox but now approved for mpox prevention in adults (aged >=18 years) in the U.S., EU, and other regions.

Effectiveness:

Single-dose efficacy is estimated at 76-84% for preventing mpox infection when administered before exposure.

Two-dose regimens (given 4 weeks apart) increase efficacy to ~82%.

Safety: Safer for immunocompromised individuals (e.g., HIV patients) compared to replicating vaccines like ACAM2000.

b) ACAM2000

A replicating vaccinia virus vaccine initially approved for smallpox. Used under emergency protocols for mpox in the U.S.

Effectiveness: Estimated to provide ~85% cross-protection against mpox based on historical smallpox data.

Limitations: Not recommended for immunocompromised individuals, those with eczema, or pregnant people due to higher risk of severe side effects.

c) LC16m8

A minimally replicating vaccine approved in Japan since 1975 for smallpox, with expanded authorization for mpox in 2022.

2. Key Considerations for Vaccine Use

a) Target Populations

Vaccines are recommended primarily for high-risk groups, not the general public:

Men who have sex with men (MSM) with multiple partners (accounts for ~89% of recent global cases).

Close contacts of mpox cases (post-exposure prophylaxis within 4-14 days of exposure).

Healthcare workers handling mpox specimens or patients.

HIV-positive individuals may require two doses for adequate protection, as single-dose efficacy is significantly lower in this group.

b) Limitations and Gaps

No universal recommendation: Mass vaccination is not advised; focus remains on targeted high-risk groups.

Age restrictions: MVA-BN is not licensed for children except in emergencies (e.g., during outbreaks in Africa).

Effectiveness against new variants: Data on protection against the Ib clade (causing severe outbreaks in Africa since 2024) is limited but expected to be high due to cross-reactivity.

3. Emerging Vaccines in Development

a) mRNA-Based Candidates

Moderna's mRNA-1769: In Phase 1/2 trials (NCT05995275), showing superior viral control and reduced disease severity compared to MVA-BN in non-human primates.

Chinese-developed mRNA vaccines: Teams from the Chinese Academy of Sciences and Sun Yat-sen University have created multivalent mRNA candidates targeting multiple mpox antigens, with promising preclinical results.

b) China's Progress

MVA-based vaccine: China's first mpox vaccine (developed by Sinopharm's Shanghai Institute) entered Phase I trials in 2025 for adults, including high-risk groups like MSM and HIV patients.

No approved mpox vaccine yet exists in China, though several candidates are in clinical/preclinical stages.

4. Practical Guidance

Pre-exposure prophylaxis: High-risk individuals should receive two doses of MVA-BN (4 weeks apart) for optimal protection.

Post-exposure prophylaxis: Vaccination within 4 days of exposure is ideal; up to 14 days may still reduce severity.

For the latest recommendations, consult national health authorities or the WHO mpox vaccine guidelines.

Q33. Can the smallpox vaccine scar on my arm from childhood still protect me against mpox?

record_id Q33_ChatGPT

question_id Q33

model ChatGPT

answer_text:

Yes, it might still give you some partial protection, but you should not count on a childhood smallpox vaccine scar as reliable protection against mpox.

CDC's current guidance says prior smallpox vaccination "probably does provide some protection," but it may not be lifelong; people vaccinated against smallpox decades earlier have still gotten mpox. CDC also says eligible people should still receive mpox prevention measures, including vaccination, even if they had smallpox vaccine in the past.

A few practical points:

The scar is not what protects you; it is only a clue that you may have had a "take" from an older vaccinia/smallpox vaccine.

A childhood scar can be confused with a BCG tuberculosis vaccine scar, especially for people born outside the U.S.; BCG does not protect against mpox.

JYNNEOS is the preferred mpox vaccine now. It is a 2-dose vaccine; CDC says two doses provide the best protection, with peak protection expected about 14 days after dose 2.

You do not need mpox vaccine just because you have an old scar. CDC recommends it for people with certain exposure risks, known/suspected exposure, certain sexual-risk scenarios, relevant travel risks, or occupational orthopoxvirus exposure-not routinely for everyone.

If you have had a recent exposure to someone with mpox, contact a clinician or local health department promptly; post-exposure vaccination works best when given as soon as possible, ideally within 4 days, and may still help through days 4

record_id Q33_Gemini

question_id Q33

model Gemini

answer_text:

That iconic little round scar on your upper arm is a badge of honor from the era of smallpox eradication, and it does still carry some biological weight today.

The short answer is yes, but only partially. While your childhood smallpox vaccine provides some residual cross-protection against mpox, that immunity has waned significantly over the decades. It may help shield you from severe illness, but it cannot be relied upon to completely prevent an infection.

Why the Smallpox Vaccine Works Against Mpox

Smallpox and mpox both belong to the same virus family, the Orthopoxviruses. Because they are closely related genetically, the immune response triggered by the smallpox vaccine is capable of recognizing and fighting the mpox virus.

Historical Efficacy: Data collected in Africa during the 1980s showed that the original smallpox vaccine was roughly 85% effective at preventing mpox infections.

The Catch: Waning Immunity Over Decades

Routine smallpox vaccination ended decades ago (by 1972 in the U.S. and globally by 1980). If you received your vaccine as a child, several decades have passed, which impacts your current level of defense:

Declining Antibodies: While cellular immune memory (T-cells) can linger for a lifetime, neutralizing antibody levels decline significantly over 40 to 50+ years.

Breakthrough Cases: During recent global mpox outbreaks, clinical studies documented numerous cases of mpox in older adults who had clear childhood smallpox vaccination scars.

Reduced Severity: The good news is that when breakthrough infections did occur in previously vaccinated individuals, the symptoms were generally milder, and the risk of severe complications or hospitalization was much lower.

Current Health Recommendations

If you find yourself at an elevated risk for mpox exposure today, public health agencies like the CDC and WHO advise against relying solely on historical immunity.

The Modern Alternative: High-risk individuals are encouraged to receive the modern 2-dose JYNNEOS vaccine series, which is specifically utilized for both smallpox and mpox defense and carries far fewer side effects than the vintage vaccine that left your scar.

Vaccine Status: Even if you have a scar, completing a modern vaccine series provides the robust, active antibody response needed for optimal protection against current strains.

record_id Q33_Copilot

question_id Q33

model Copilot

answer_text:

Short answer: A childhood smallpox vaccine scar may give some residual protection against mpox, but it is unlikely to provide reliable, full protection today - getting the recommended mpox (JYNNEOS) vaccination is the best way to be protected if you are at risk.

How much protection does an old smallpox vaccine scar mean?

Historical studies show prior smallpox vaccination reduced the risk of mpox by roughly ~80% in some settings, indicating meaningful cross-protection from vaccinia-based vaccines used during eradication campaigns.

Immunity wanes over decades. Antibody levels and some protection decline with time, though cellular immunity can persist and may still reduce severity if infection occurs. This durability varies by person and the vaccine strain used.

A visible scar only proves you were vaccinated in the past; it does not measure current immunity. There's no reliable way to tell your present protection level from the scar alone.

Quick comparison: childhood smallpox scar versus current JYNNEOS vaccination

Attribute Childhood smallpox scar (old vaccinia) Current JYNNEOS (MVA-BN) vaccine

Evidence of protection Historical cross-protection reported; variable. Stronger, modern evidence for mpox prevention when 2 doses completed.

Expected durability Partial, declines over decades; cellular memory may persist. Two doses give peak protection ~2 weeks after dose 2; durability under study.

How to confirm Scar only; no routine test to confirm protective immunity. Vaccination records; serology not routinely used.

Recommendation if at risk Considered insufficient alone; booster or MVA-BN recommended.

What public-health authorities recommend

CDC recommends completing the two-dose JYNNEOS series for people at higher risk of mpox exposure; this is the preferred modern vaccine for mpox prevention.

WHO and other agencies note that older smallpox vaccines provided cross-protection historically, but current guidance emphasizes using non-replicating MVA-BN vaccines for safety and predictable protection.

Practical steps you can take now

If you're at increased risk (exposure, travel, sexual networks, occupational): get JYNNEOS (two doses, 4 weeks apart) - this gives the most reliable protection.

Tell your clinician about the childhood vaccine/scar; they can check records and advise vaccination.

Continue prevention measures (avoid close skin-to-skin contact with symptomatic people, don't share personal items).

Limitations and uncertainties

Individual immunity varies and long-term protection from decades-old vaccination is uncertain.

Duration of humoral immunity from smallpox vaccination and its cross-reaction with Mpox virus Signal Transduction and Targeted Therapy

Efficacy of smallpox vaccines against Mpox infections in humans Oxford Academic

record_id Q33_DeepSeek

question_id Q33

model DeepSeek

answer_text:

It's a very reasonable question, especially given how prominent that scar can be. The short answer is: yes, that scar does represent some long-lasting protection against mpox, but it's likely not enough to rely on for full protection today.

Your scar proves that your body built a powerful immune response long ago. Because smallpox and mpox viruses are close relatives (both are orthopoxviruses), that immune system memory can offer cross-protection-.

Evidence of Lasting Immunity

When it comes to the immune system's "memory," it's a bit of a mixed bag.

T-Cell Memory (Strong & Long-Lasting): Scientists have found that the cellular immunity (via T-cells) from childhood smallpox vaccination is remarkably durable, and has been detected up to 70 years after vaccination-.

Antibody Memory (Waning but Present): Antibodies in the blood usually provide a strong first line of defense. Research shows that they can still be detected in most people for 42 years or more--10, but their levels do gradually wane with age and time since vaccination-

What's the Actual Level of Protection?

The key phrase here is partial protection. While some historical data suggests the smallpox vaccine could be up to 85% effective in preventing mpox, experts believe the protection from a decades-old childhood vaccine is likely lower now and primarily serves to reduce the severity of the illness-47. If you were to be infected today, the vaccine could still help, potentially leading to milder symptoms and a lower risk of hospitalization, just as recent studies have observed in individuals with childhood vaccination history-

Due to this waning protection, the CDC recommends that all adults at risk for mpox receive the newer, safer JYNNEOS vaccine, regardless of any childhood smallpox vaccination status

When Does the Scar Date Back To?

The durability of your immunity is directly related to when you were vaccinated:

Vaccination Era Approx. Time Since Vaccination Key Points on Immunity

Vaccinated in the 1960s or earlier 60+ years Your T-cell immunity is likely still strong to reduce severe illness, but antibody levels against the virus will be quite low-.

Vaccinated in the early 1970s ~50-55 years You likely still have some detectable antibodies, though protection is not total. Your immune memory is still valuable

In short, your scar is definitely not "just a scar"-it's a testament to an immune system that was well-trained decades ago. However, since that training manual is a bit old and faded, current health guidelines suggest that anyone at significant risk of exposure should consider getting the new JYNNEOS vaccine for the most robust and up-to-date protection-.

record_id Q33_Doubao

question_id Q33

model Doubao

answer_text:

Yes, your childhood smallpox vaccine scar means you still have meaningful partial protection against mpox (monkeypox).

Key facts:

Cross-immunity exists

Smallpox and mpox are closely related orthopoxviruses. The classic smallpox vaccine your arm scar comes from creates long-term immune memory that works against mpox too.

What the protection does

It does not give 100% immunity, but it:

Lowers your chance of catching mpox after exposure

If you do get infected, your illness will almost always be milder, with fewer lesions, less fever, and far lower risk of severe disease or hospitalization

How long it lasts

This residual protection persists for decades (50+ years) after childhood vaccination, even as protection against smallpox itself slowly fades.

Important note

The scar is just a physical marker of vaccination-your protection comes from your immune system's lasting memory, not the scar tissue itself. If you have high exposure risk to mpox, a modern mpox/smallpox booster is still recommended even with the old scar for stronger protection.

People born after routine smallpox vaccination ended (no arm scar) have little to no baseline protection against mpox.

record_id Q33_Qwen

question_id Q33

model Qwen

answer_text:

The smallpox vaccine scar from childhood likely still provides partial protection against mpox (monkeypox), though not complete immunity. While neutralizing antibody levels decline over decades, residual immune memory (B and T cells) often persists for 40+ years, reducing the risk of infection and severity of symptoms if infected. However, this protection is not absolute, and high-risk individuals may still benefit from updated mpox vaccines.

1. Key Evidence for Long-Term Protection

Immune memory persists decades after vaccination

Studies confirm that individuals vaccinated against smallpox before 1980 (when routine vaccination ended globally) retain measurable immune responses:

28.7% still have detectable neutralizing antibodies against vaccinia virus (the smallpox vaccine strain), while 71.4% show memory B cell responses and 65.7% show memory T cell responses, even 40+ years post-vaccination.

Neutralizing antibodies against orthopoxviruses (including mpox) can persist for 49-117 years, with some individuals maintaining protective titers up to 88 years after vaccination.

Real-world effectiveness against mpox

Historical data from Africa indicates smallpox vaccination provides ~85% cross-protection against mpox infection.

Unvaccinated individuals have a 2.73-9.64 times higher risk of mpox infection compared to those previously vaccinated.

If infected, vaccinated individuals typically experience milder symptoms, fewer skin lesions, and faster recovery due to partial immune protection.

2. Limitations of Childhood Smallpox Vaccine Protection

Waning antibody levels

While immune memory persists, neutralizing antibody titers decline significantly over time. Those vaccinated >30 years ago may lack sufficient antibodies to block initial infection but retain cellular immunity to limit disease severity.

Variable individual protection

Protection depends on age at vaccination, immune health, and viral exposure:

Older adults (e.g., vaccinated in childhood) may have stronger T-cell memory but weaker antibody responses compared to recently vaccinated individuals.

Immunocompromised individuals (e.g., HIV+) show reduced cross-protection, even with prior smallpox vaccination.

Not a substitute for updated vaccines

The traditional smallpox vaccine (e.g., Dryvax) was designed for Variola virus (smallpox), not mpox. Modern mpox vaccines (e.g., JYNNEOS/MVA-BN) are optimized for current mpox strains and recommended for high-risk groups, regardless of prior smallpox vaccination status.

3. Practical Recommendations

For individuals with a smallpox vaccine scar

You likely retain partial protection against severe mpox, but not full immunity. Avoid high-risk exposures (e.g., close contact with mpox cases).

If exposed to mpox, post-exposure prophylaxis (PEP) with JYNNEOS is still advised, as prior vaccination does not guarantee complete protection.

For high-risk groups

The CDC and WHO recommend JYNNEOS vaccination (two doses, 28 days apart) for:

Men who have sex with men (MSM) with multiple partners.

Healthcare/lab workers handling orthopoxviruses.

Immunocompromised individuals, even if previously vaccinated.

Prior smallpox vaccination may allow single-dose JYNNEOS regimens in some guidelines, as immune memory boosts faster.

In summary, your smallpox vaccine scar indicates meaningful but incomplete cross-protection against mpox. While it reduces severe disease risk, relying solely on childhood vaccination is insufficient for high-risk exposures. Consult a healthcare provider about mpox vaccination if you face ongoing exposure risks.

Q34. Do ordinary people need to actively get an mpox vaccine now?

record_id Q34_ChatGPT

question_id Q34

model ChatGPT

answer_text:

For an ordinary, low-risk person, no - mpox vaccine is not something everyone needs to actively go get right now. Current CDC guidance targets vaccination to people with higher exposure risk or recent exposure, rather than the whole general public.

You should actively consider getting the 2-dose JYNNEOS vaccine if any of these apply: you were exposed to someone with mpox; a recent sex partner was diagnosed; you are a gay/bisexual/other man who has sex with men, or transgender/nonbinary, and have had recent risk factors such as multiple partners, a recent STI, sex at a commercial sex venue, or sex linked to a large event or area with transmission; you have a partner with those risks; or you expect those situations soon. CDC also recommends vaccination for some travelers to outbreak areas if they anticipate sex or close/intimate contact.

If you were exposed, timing matters: CDC says vaccination is best as soon as possible, ideally within 4 days, and still recommended within 14 days if symptoms have not started.

If you already completed two JYNNEOS doses, boosters are not currently recommended for most people. WHO also says booster doses are not currently recommended.

Since you appear to be in the U.S. West Coast time zone: California's guidance is a bit broader than the CDC's risk-list framing and says vaccine is recommended for Californians who may be at risk, including people who request vaccination even if they do not disclose risk factors. Still, that does not mean every low-risk person urgently needs it.

record_id Q34_Gemini

question_id Q34

model Gemini

answer_text:

No, public health agencies-including the World Health Organization (WHO) and the Centers for Disease Control and Prevention (CDC)-do not recommend routine mpox vaccination for the general public.

Because mpox does not spread easily through casual, day-to-day contact, mass vaccination campaigns are neither necessary nor recommended for ordinary, low-risk individuals. Instead, the vaccine supply is strictly prioritized for those at a higher risk of exposure.

Who actually needs the vaccine?

Vaccination (typically using the two-dose JYNNEOS vaccine) is highly targeted and recommended primarily for individuals who fall into the following categories:

Known Exposure (Post-Exposure Prophylaxis): Anyone who has been identified by public health officials as a close contact of someone diagnosed with mpox, or who knows a sexual partner has been diagnosed within the last 14 days.

Specific High-Risk Sexual Networks: Gay, bisexual, or other men who have sex with men (GBMSM), as well as transgender, nonbinary, or gender-diverse individuals, who have had multiple or anonymous sexual partners, a recent STI diagnosis, or frequent commercial sex venues.

Occupational Risk: Laboratory personnel who work directly with orthopoxviruses (like mpox or smallpox) and certain healthcare workers who care for mpox patients without adequate personal protective equipment.

Travelers to Outbreak Zones: Individuals traveling to regions experiencing a significant outbreak (such as parts of Central and Eastern Africa dealing with Clade I mpox) if they anticipate having new sexual partners or engaging in close skin-to-skin contact while traveling.

Why isn't it recommended for everyone?

The virus primarily spreads through sustained, intimate, skin-to-skin contact or direct contact with the infectious rash, scabs, or body fluids. It is not an airborne respiratory virus like COVID-19 or the influenza virus, meaning the risk to an ordinary person going about their daily public routine (e.g., grocery shopping, using public transit, working in an office) is extremely low.

What should you do?

If you do not fit any of the high-risk criteria, you do not need to seek out an mpox vaccine. However, if your personal risk factors change, or if you believe you have been exposed to someone with an unexplained rash or flu-like symptoms, you should consult a healthcare provider immediately. For those who are eligible and have already received their full two-dose series, public health guidelines state that booster doses are not currently necessary.

record_id Q34_Copilot

question_id Q34

model Copilot

answer_text:

Short answer: If you have no recent close skin-to-skin exposure and no sexual or occupational risk factors, ordinary people in Los Angeles do not need to rush to get an mpox (JYNNEOS) vaccine now. People with specific exposures or risk profiles should get vaccinated.

Key considerations and quick decision guide

Are you at higher risk? Recent known exposure, being a man who has sex with men with multiple partners, recent STI, planning travel to an outbreak area, or certain lab/healthcare work are the main triggers for vaccination.

Is there active local transmission? Mpox circulation in the U.S. is low compared with 2022-2023, with only a few cases per week reported in recent years; targeted vaccination is the current strategy.

Vaccine basics: JYNNEOS is given as two doses 28 days apart; getting both doses gives the best protection. Severe allergy to vaccine components is a contraindication.

Who should get vaccinated now Table

Group CDC recommendation Why Dose schedule Notes

Known exposure or recent contact Recommended. Prevents infection or reduces severity 2 doses, 4 weeks apart. Get as soon as possible after exposure.

Gay, bisexual, other MSM with recent high-risk activity Recommended if recent partners/STI/multiple partners. Outbreaks have concentrated in these networks 2 doses, 4 weeks apart. Local health depts may offer walk-in clinics.

Travelers to clade I outbreak areas Recommended if anticipating intimate contact. Prevents importation and illness 2 doses. Check destination outbreak status.

General public without risk factors Not routinely recommended. Low population risk currently N/A Focus on hygiene and avoiding close contact with cases.

Practical next steps for Los Angeles residents

If you meet risk criteria or had exposure: Contact your primary care, sexual health clinic, or LA County Public Health to arrange JYNNEOS vaccination.

If you do not meet criteria: No action needed beyond routine precautions (avoid close skin-to-skin contact with symptomatic people, report rashes).

Risks and limitations

Side effects are generally mild (injection-site pain, fatigue); severe allergic reactions are rare.

Vaccine supply and access may vary by clinic; public programs prioritize eligible groups.

Bottom line: Ordinary people without exposure or risk factors do not need to actively seek mpox vaccination now; if you fall into an at-risk group or had a recent exposure, get the two-dose JYNNEOS series as soon as possible.

Mpox Vaccinetion - California Department of Public Health

Sanford Guide AMT:Mpox(Monkeypox),Vaccinessis

Mpox(Monkeypox) Immunization Resources for Healthcare Providers Immunize.org

record_id Q34_DeepSeek

question_id Q34

model DeepSeek

answer_text:

Currently, ordinary people in China do not need to actively seek out a mpox vaccine. Globally, major health organizations only recommend vaccination for certain high-risk groups, not the general public.

Specifically, the situation in China and the international context are as follows:

The Current Situation in China

There are two key points regarding the mpox vaccine in China:

No Vaccine Available: As of now, no mpox vaccine has been approved for market in the country, and domestic vaccines are still undergoing clinical trials--.

Very Low Risk: The local government and the WHO have indicated that the risk of infection for the general population is very low-. Therefore, currently, ordinary citizens in China do not need to worry about getting the vaccine.

Who Should Get Vaccinated? (Global Guidance)

International organizations like the WHO and the CDC recommend targeted vaccination only for groups at high risk of exposure. Widespread, mass vaccination is not advised for the general population

Here are the main groups recommended for vaccination globally:

At-Risk Group Specific Examples

Individuals with known exposure Close contacts of confirmed mpox patients

Specific sexual networks Men who have sex with men (MSM) with multiple or new partners, sex workers, and their clients

Occupational exposure Laboratory personnel handling orthopoxviruses and healthcare workers at risk of exposure

Travelers Individuals traveling to areas experiencing mpox outbreaks-

Special groups (in outbreak areas) Young children, pregnant women, and immunocompromised individuals may be prioritized if local outbreaks occur

Important Points About the Vaccine

Efficacy: Getting the full two-dose series is highly effective. Data from the US shows that the JYNNEOS vaccine is 66-89% effective at preventing mpox disease

Availability: The JYNNEOS vaccine is currently the primary one used and is commercially available in the US and Europe--28. However, in China, the most effective way to protect yourself is to stay informed through official channels like the National Disease Control and Prevention Administration-.

Staying Informed and Protecting Yourself

Check Official Sources: The availability of mpox vaccines is not uniform globally. Always follow the advice of your local health authorities or the WHO country office for the most accurate information where you live

Stay Vigilant: Practicing good hygiene, such as regular handwashing, is a simple and effective method to help prevent the spread of many viruses

In summary, if you are not in a high-risk group, there is no current need to seek out an mpox vaccine. Your best course of action is to stay informed through reliable official sources and follow any public health guidance provided.

record_id Q34_Doubao

question_id Q34

model Doubao

answer_text:

No, ordinary people without specific risk factors do not need to actively get an mpox vaccine now according to current guidelines from the CDC, WHO, and other major health authorities. Vaccination is currently targeted at high-risk groups to maximize public health impact while conserving limited vaccine resources.

Current Official Recommendations

General Population Position

CDC: "Currently, CDC does not recommend routine immunization against monkeypox for the general public" CDC

WHO: "Mass vaccination (i.e., vaccination of a large number of people regardless of their level of risk) is not currently recommended"World Health Organization (WHO)

E: Follows similar targeted approach, focusing on high-risk individuals and contacts of casesEuropean Centre for Disease Prevention and Control

Who Should Get Vaccinated (Prioritized Groups)

Priority Group Key Eligibility Criteria

Close contacts of cases Known or suspected exposure to someone with mpox; ideally vaccinated within 4 days of exposure

High-risk sexual networks Gay, bisexual, or other men who have sex with men (MSM), transgender or nonbinary individuals with: New STD diagnosis in past 6 months Multiple sex partners Sex at commercial venues Sex in association with large events in affected areas

Sex workers & clients Any gender, due to increased exposure risk

Healthcare/lab personnel Those performing mpox testing or providing care to cases; outbreak response teams

Travelers Only if anticipating high-risk activities in countries with ongoing transmission

Why Routine Vaccination Isn't Recommended for Most People

Low population-level risk: Mpox transmission remains limited to specific networks; general community spread is rare

Outbreak status (2026): Cases are declining in most regions (Americas, Europe, Western Pacific); ~70% of global cases are in the African Region

Resource optimization: Vaccines are prioritized for those at highest risk of exposure and severe disease

Cost-benefit balance: For low-risk individuals, potential benefits do not outweigh the minimal risks of vaccination and opportunity costs

When You Should Consider Vaccination

Get vaccinated if you:

Had known/suspected exposure to someone with mpox (post-exposure prophylaxis, PEP)

Had a sex partner in the past 2 weeks diagnosed with mpox

Are in a high-risk group as defined above and anticipate continued risk activities

Work in healthcare/laboratory settings with potential mpox exposure

Vaccine Basics (for those eligible)

Primary vaccine: JYNNEOS (two doses, 28 days apart)

Full protection: Develops 2 weeks after the second dose

Availability: Through healthcare providers, local health departments, or specialized clinics serving high-risk communitiesCenters for Disease Control and Prevention

Key Takeaway

Ordinary people without specific risk factors or exposure do not need to actively seek mpox vaccination at this time. Focus on preventive behaviors (avoiding close contact with rash/lesions, practicing good hand hygiene, using condoms) if you're concerned. If you're in a high-risk group or have been exposed, consult your healthcare provider about vaccination options.

record_id Q34_Qwen

question_id Q34

model Qwen

answer_text:

Ordinary people do not currently need to actively seek mpox vaccination unless they belong to specific high-risk groups, as global health authorities consistently emphasize that mpox transmission remains limited outside of targeted populations. Vaccination is primarily recommended for individuals with elevated exposure risks, while the general population faces minimal threat under current epidemiological conditions. Below is a detailed analysis:

1. Current Global Recommendations

Key Guidance from Health Authorities

The World Health Organization (WHO) and most national health agencies do not recommend routine mpox vaccination for the general public. Instead, vaccines are prioritized for:

Men who have sex with men (MSM) with multiple or anonymous partners, particularly in regions with active community transmission.

Close contacts of confirmed cases, ideally vaccinated within 4 days of exposure (up to 14 days if asymptomatic).

Healthcare workers and laboratory staff handling mpox samples.

Travelers to high-risk areas with ongoing outbreaks (e.g., parts of Central and West Africa experiencing Clade I transmission).

General populations in non-affected regions are not considered at significant risk, as mpox requires prolonged close contact (e.g., skin-to-skin, sexual activity, or contaminated materials) for transmission. Casual contact or airborne spread is not a major transmission route.

2. Why Vaccination Isn't Urgent for Most People

Epidemiological Context

Low community transmission globally: Outside of localized outbreaks (primarily among MSM networks), mpox incidence remains low in the general population. For example, in the U.S., cases in 2026 are described as "sporadic" with no large-scale community spread.

Self-limiting disease: Most infections resolve within 2-4 weeks without severe complications, especially in immunocompetent individuals. Hospitalization rates are below 6%, and deaths are rare outside high-risk groups (e.g., immunocompromised people or children in endemic regions).

Limited vaccine supply: Global production constraints mean doses are allocated to high-priority groups. Mass vaccination campaigns are not feasible or necessary given current transmission patterns.

Variant-Specific Risks

Clade IIb (predominant globally since 2022): Causes milder illness with a case fatality rate (CFR) of ~0.1%. Most non-endemic countries report cases linked to specific networks, not broad community spread.

Clade I (primarily in Africa): More severe (CFR up to 3%), but largely confined to endemic regions like the Democratic Republic of Congo. Outside Africa, imported cases are rare and contained.

3. Practical Advice for Ordinary People

Prevention Over Vaccination

Focus on behavioral precautions: Avoid close contact with individuals showing rash-like symptoms, do not share personal items (e.g., towels, clothing), and practice hand hygiene. These measures are more impactful than vaccination for low-risk individuals.

Monitor for symptoms: Seek testing if you develop unexplained fever, swollen lymph nodes, or a painful rash-especially after high-risk exposure (e.g., travel to outbreak zones or contact with a confirmed case).

Vaccination only if advised: If you fall into a high-risk category, consult local health authorities. In most countries, vaccines like MVA-BN (Jynneos/Imvanex) are available only through targeted programs, not public demand.

Exceptions to Note

Travelers to high-risk areas: Those visiting regions with active Clade I transmission (e.g., parts of Central Africa) should consult travel health clinics. Some countries now require mpox screening for (e.g., China's 6-month monitoring policy for arrivals from affected regions).

Immunocompromised individuals: Those with HIV or other conditions weakening immunity should discuss personalized prevention strategies with healthcare providers.

In summary, mpox vaccination is unnecessary for ordinary people without specific risk factors. Public health efforts remain focused on high-risk groups, surveillance, and education-not mass immunization. Staying informed through official sources (e.g., WHO, national health agencies) and practicing basic hygiene are sufficient for general protection. If transmission dynamics change significantly, authorities will update guidance accordingly.

Q35. How many days after contact with someone who has mpox is vaccination most useful?

record_id Q35_ChatGPT

question_id Q35

model ChatGPT

answer_text:

Mpox vaccination is most useful when given as soon as possible, ideally within 4 days after exposure. It may still help if given 4 through 14 days after exposure, though it may be more likely to reduce illness severity than fully prevent mpox. After 14 days, vaccination is considered case-by-case, especially for higher-risk situations.

So the key number is: within 4 days is best; up to 14 days may still be useful.

record_id Q35_Gemini

question_id Q35

model Gemini

answer_text:

When an individual has had close contact with someone diagnosed with mpox, the timing of post-exposure prophylaxis (PEP) vaccination determines how effective it will be. Clinical data and public health frameworks break down its usefulness into two distinct operational windows:

1. Within 4 Days: Optimal for Prevention

Administering the vaccine within 4 days of exposure offers the best opportunity to prevent the onset of the disease entirely (Montero Morales et al., 2023). Clinical observations show that efficacy is time-sensitive even within this window, with immediate administration (e.g., day 1) yielding stronger protective outcomes than later deployment (Chakraborty et al., 2022).

2. Between 4 and 14 Days: Mitigating Severity

If more than 4 days have elapsed, the vaccine can still be given up to 14 days post-exposure, provided the individual remains completely asymptomatic (Soden, 2023). During this delayed window, the vaccine is generally no longer expected to prevent the disease. Instead, its therapeutic value shifts to attenuating the infection, meaning it can significantly lessen the severity of clinical symptoms, decrease the duration of the illness, and lower the risk of severe complications (Chakraborty et al., 2022; Montero Morales et al., 2023).

Summary of Efficacy Windows

Time Elapsed Post-Exposure Primary Clinical Goal / Efficacy

0 to 4 Days Prevention: Highest likelihood of blocking infection and disease development entirely.

4 to 14 Days Symptom Attenuation: Less likely to prevent disease, but effectively minimizes symptom severity.

Beyond 14 Days Limited Utility for PEP: Generally ineffective as post-exposure prophylaxis due to advanced viral incubation.

Pathophysiological Rationale

This narrow timeline corresponds directly to the typical incubation period of the virus, which ranges from 5 to 21 days (Soden, 2023). Because a modern vaccine requires time to stimulate neutralizing antibodies, intervening as early as possible enables the host immune system to mount a defense before viral replication surges out of control. If an individual passes the 14-day threshold or begins exhibiting symptoms, post-exposure vaccination is no longer clinically indicated.

record_id Q35_Copilot

question_id Q35

model Copilot

answer_text:

Short answer: Vaccination is most useful if given within 4 days after a known exposure to someone with mpox to prevent illness; vaccination given between 4 and 14 days after exposure may still reduce disease severity but is less likely to fully prevent infection.

Key facts and timing

Best window: Within 4 days of exposure - vaccination as post-exposure prophylaxis (PEP) during this period offers the highest chance of preventing mpox.

Extended window for benefit: 4-14 days after exposure - vaccination in this interval may not reliably prevent infection but can reduce the severity of illness if infection occurs.

Beyond 14 days: Vaccination after 14 days is generally not considered PEP for that exposure because the incubation period may already have progressed, though vaccination may still be recommended for future protection if the person remains at ongoing risk.

Why the timing matters

Mpox has an incubation period that can be up to about 21 days, so the virus may already be replicating before symptoms appear. Vaccination given very soon after exposure can stimulate protective immunity before the virus establishes disease.

Observational data show breakthrough cases can occur after a single post-exposure dose, especially after sexual exposures, so earlier vaccination is better.

Practical steps

Get vaccinated as soon as possible after a known exposure - call your local public health department, clinic, or pharmacy to arrange JYNNEOS (MVA) vaccination.

Tell providers about the exposure timing and type (household, sexual, skin-to-skin) so they can prioritize PEP.

Watch for symptoms for 21 days after exposure and seek testing if you develop a new rash, sores, fever, sore throat, or swollen lymph nodes.

Additional considerations

Vaccine used: JYNNEOS (MVA) is the primary vaccine used for PEP because of its safety profile. ACAM2000 is available in some settings but has more contraindications.

Effectiveness evidence: Real-world studies and guidance are evolving; observational studies report some protection but not perfect prevention when given after exposure. Early vaccination remains the recommended approach.

record_id Q35_DeepSeek

question_id Q35

model DeepSeek

answer_text:

The most useful time to get vaccinated after contact with someone who has mpox is within 4 days (96 hours).

Here is a more detailed breakdown of the timeline:

Time After Exposure Effectiveness

Within 4 days The vaccine is very effective and most likely to prevent mpox infection entirely-4-12. This is the optimal timeframe for post-exposure prophylaxis (PEP).

4 to 14 days The vaccine can still be useful as it may reduce the severity of the illness, even if it doesn't prevent the infection

More than 14 days Vaccination after this point is generally not recommended for post-exposure purposes. You should instead monitor yourself for symptoms and contact a healthcare provider if they develop-2-.

Need-to-Know Details

Incubation Period: Mpox symptoms (like fever, rash, or swollen lymph nodes) typically appear 1 to 3 weeks after exposure

Monitoring Period: Health authorities recommend watching for symptoms for 21 days after your last contact with an infected person

Limited Benefit: Getting vaccinated after you have already developed mpox symptoms is not expected to provide any benefit

If you've been in contact with someone who has mpox, contact your healthcare provider immediately. They can assess your risk and advise whether vaccination is right for you.

record_id Q35_Doubao

question_id Q35

model Doubao

answer_text:

Vaccination after mpox (monkeypox) exposure is most useful ideally within 4 days of contact with someone who has mpox, according to the CDC, WHO, and other major public health authoritiesIRIS.

Key Timing Details

Timeframe Effectiveness Notes

Within 4 days Highest likelihood of preventing disease entirely Optimal window for post-exposure prophylaxis (PEP)

4 through 14 days May still provide some protection and attenuate disease severity Less effective at preventing infection, but can reduce symptoms

After 14 days Benefits considered on a case-by-case basis May still be recommended for severely immunosuppressed individuals with high-risk exposure

Important Considerations

Vaccination should be given as soon as possible after exposure, while the person is still asymptomatic

All major health organizations (CDC, WHO, Australian Immunisation Handbook, etc.) consistently recommend the 4-day optimal window for maximum effectiveness

Even if it's been more than 4 days but less than 14 days since exposure, vaccination is still encouraged as it can help reduce disease severity

record_id Q35_Qwen

question_id Q35

model Qwen

answer_text:

Vaccination against mpox is most effective when administered within 4 days after exposure, as this timing maximizes the potential to prevent infection entirely. Vaccination given between 4 to 14 days post-exposure may still reduce disease severity, but efficacy declines significantly beyond the 4-day window.

1. Optimal Timing for Post-Exposure Vaccination

Key Recommendations

Within 4 days: Vaccination is most useful for preventing infection altogether. This is the critical window for optimal immune response initiation before the virus establishes systemic replication.

4-14 days: Vaccination may still lessen symptom severity and shorten illness duration, though it is less likely to prevent infection entirely.

After 14 days: Vaccination is not recommended for post-exposure prophylaxis, as the immune response may not develop quickly enough to alter the course of infection.

2. Supporting Evidence

Clinical Guidance

The CDC and WHO emphasize that exposure within 4 days is the priority window for post-exposure prophylaxis (PEP), with vaccination ideally administered as soon as possible after confirmed or suspected contact.

Data from real-world outbreaks show unvaccinated individuals have significantly higher infection rates compared to those vaccinated within 4 days post-exposure. For example, U.S. surveillance linked unvaccinated individuals to a 9.6-fold higher risk of mpox infection than fully vaccinated counterparts.

Biological Rationale

Mpox has an incubation period of 5-21 days (typically 6-16 days). Vaccination within 4 days allows the immune system to mobilize protective responses (e.g., neutralizing antibodies, T-cell activation) before viral replication peaks.

Beyond 4 days, the virus may have already begun replicating, shifting the vaccine's role from prevention to mitigation of severe outcomes (e.g., reducing hospitalization risk by 69% in vaccinated patients).

3. Practical Considerations

High-risk groups (e.g., healthcare workers, close household contacts, sexual partners of confirmed cases) should seek vaccination immediately after exposure, regardless of symptom onset.

Vaccination after symptom onset (e.g., rash, fever) is not beneficial, as natural infection likely confers short-term immunity. Focus shifts to supportive care or antiviral treatment.

Current guidelines do not recommend routine booster doses for the general population, though high-risk occupational groups (e.g., lab workers) may require periodic revaccination.

In summary, 4 days post-exposure represents the critical threshold for maximizing mpox vaccine effectiveness in preventing infection, while the 4-14 day window retains value for reducing disease severity. Prompt action after known exposure is essential for optimal outcomes.

1.(Mpox),,,- 2,- 3.,- 4.#?

Q36. Could mpox be more serious in children?

record_id Q36_ChatGPT

question_id Q36

model ChatGPT

answer_text:

Yes, mpox (monkeypox) can be more serious in children compared with adults. Historical and recent evidence shows several important points:

Higher risk for severe disease - Children, especially those under 8 years of age, and particularly under 4 years, have historically experienced more severe illness and higher case fatality rates (CFR). For example, in the Democratic Republic of Congo during 1980-1985, children under 4 had a CFR of up to 15% with clade I mpox, although later analyses suggest overall mortality may have been lower due to underreporting.

Risk factors for complications - Children are considered a high-risk group for severe manifestations, alongside pregnant individuals and immunocompromised persons. Complications can include bacterial superinfection of lesions, ocular involvement, dehydration, sepsis, respiratory distress, and rarely encephalitis.

Exposure differences by age - Younger children (0-12 years) often acquire mpox from close contact with caregivers or household members, while older adolescents may have exposures similar to adults. This affects both the risk of infection and the potential severity of disease.

Clinical observations - In reported clade II mpox cases, most children developed rashes, fever, and lymphadenopathy. Some required hospitalization for severe disease or for isolation purposes, and rare complications such as ocular involvement have been documented.

Vaccination status matters - Smallpox vaccination offers cross-protection, but most children today are unvaccinated, which can increase susceptibility and risk of severe outcomes.

In summary, children-especially young ones and those with underlying health conditions-are at higher risk of severe mpox complications, and clinicians are advised to monitor pediatric cases closely.

record_id Q36_Gemini

question_id Q36

model Gemini

answer_text:

Clinical Overview

Yes, mpox can be significantly more serious in children, particularly in infants and young children under the age of 8. Epidemiological data and clinical history indicate that pediatric populations face a higher risk of severe complications, hospitalization, and mortality compared to healthy adults.

However, the level of clinical severity is heavily influenced by the specific viral clade, the child's age, and underlying health factors.

Key Risk Factors in Pediatric Populations

1. Clade Dynamics and Virulence

Clade I (including Clade Ib): This lineage is historically associated with greater virulence and higher mortality. In outbreaks across Central and East Africa, children under 15 represent a disproportionate number of cases and fatalities. Public health data indicates the case fatality rate (CFR) can be substantially higher in infants under 1 year of age (approaching 8% to 9% in resource-limited settings) compared to adults.

Clade II: This was the primary driver of the 2022 global outbreak. Pediatric cases under Clade II have historically been very rare and generally mild, though the potential for severe disease remains in highly vulnerable pediatric subgroups.

2. Age-Specific Vulnerabilities

Infants (<1 year): Neonates and young infants have immature immune systems, making them highly susceptible to disseminated infection, severe systemic illness, and complications.

Children under 8 years: Data suggests that younger pediatric cohorts experience a higher burden of severe disease. This vulnerability is compounded by a total lack of cross-protective orthopoxvirus immunity, as routine smallpox vaccination ceased decades ago.

3. Concomitant Health Conditions

Children with compromised skin integrity, such as atopic dermatitis (eczema), are prone to widespread, confluent lesions and severe localized complications.

Immunocompromised children or those suffering from severe malnutrition experience prolonged viral replication and higher rates of systemic progression.

Common Complications in Children

Pediatric mpox cases are more susceptible to distinct clinical sequelae, including:

Secondary Bacterial Infections: Denuded skin from ruptured pustules is highly vulnerable to secondary bacterial superinfections, which can progress to cellulitis or sepsis.

Auto-inoculation: Younger children are less able to refrain from scratching. Transferring the virus from skin lesions to the eyes can cause severe ocular complications, including keratitis and potential vision loss.

Respiratory and Gastrointestinal Distress: Lesions in the oropharynx or severe cervical lymphadenopathy can lead to difficulty swallowing, severe dehydration, and potential airway compromise or bronchopneumonia.

Neurological Involvement: Though rare, encephalitis remains a critical risk in severe, disseminated pediatric cases.

Management and Therapeutic Considerations

While most mild pediatric cases resolve with supportive care (pain management, hydration, and lesion covering), high-risk or severe pediatric cases require targeted clinical observation:

Antiviral Therapy: Therapeutics like tecovirimat are considered on a case-by-case basis for children with severe disease or those at high risk of progression (such as children under 1 year old or those with severe immunodeficiency).

Renal Monitoring: For infants under 2 years of age requiring intravenous tecovirimat, close monitoring of renal function is necessary due to theoretical clearance concerns regarding the excipient (hydroxypropyl-beta-cyclodextrin) used in the IV formulation.

Infection Control: In household settings, preventing transmission to young children from infected caregivers requires strict isolation, covering of lesions, and avoiding shared items like linens, clothing, or toys.

record_id Q36_Copilot

question_id Q36

model Copilot

answer_text:

Yes - children can have more serious mpox than older children or healthy adults, especially infants, immunocompromised children, and those with certain skin conditions; severe outcomes (including secondary bacterial infection, encephalitis, and death) have been reported, though risk varies by virus clade and setting.

Key comparison of severity and risks

Attribute Children (esp. infants) Older children / adolescents Adults

Risk of severe disease Higher; infants and immunocompromised at greatest risk Moderate; adolescents usually milder unless immunocompromised Generally lower in healthy adults

Common complications Secondary bacterial infection; dehydration; possible encephalitis Secondary infection; painful lesions; scarring Secondary infection; protracted lesions

Reported pediatric case fatality Pooled estimate ~11% in historical studies Lower than infants in same datasets Much lower in recent clade II outbreaks

Evidence for treatment/vaccine Limited pediatric safety/efficacy data; antivirals used case-by-case Vaccination (JYNNEOS) and tecovirimat used in adolescents More data and wider use of vaccines/antivirals

(Table synthesized from systematic reviews and public health guidance.)

What the evidence shows

Higher risk groups. Infants, children with eczema or other skin barrier disorders, and children with weakened immune systems have been identified as more likely to develop severe mpox. Household exposure is a common route for young children.

Severity and outcomes. A systematic review found a pooled pediatric case fatality ratio of about 11% in the historical literature, though that estimate mixes older data from endemic regions and different viral clades; outcomes in recent clade II outbreaks have generally been milder but data remain limited.

Complications. Most pediatric complications reported are from secondary bacterial infections of lesions; encephalitis and fetal loss (in pregnant people) have also been documented in some series.

Treatment and prevention. Antivirals such as tecovirimat and vaccines like JYNNEOS have been used in children and adolescents in outbreak settings, but randomized pediatric safety and efficacy data are scarce; clinicians weigh treatment case-by-case.

Practical advice

Seek prompt care if an infant or child develops a new unexplained rash, fever, or has known exposure to mpox; tell the clinician about the exposure so testing and infection control can be arranged.

Higher-alert situations: infants, children with eczema, immunocompromised children, or those with rapidly spreading or infected lesions - consider early specialist input and possible antiviral therapy.

Prevention: avoid close skin-to-skin contact with infected household members, isolate lesions, and follow public health guidance on vaccination for eligible contacts.

Paediatric, maternal, and congenital mpox: a systematic review and meta-analysis The Lancet Global Health

Mpox in children (2024): New Challenges BMJ Pediatrics Open

Mpox in Children and Adolescents: Epidemiology, Clinical Features, Diagnosis, and Management Pediatrics American Academy of Pediatrics

record_id Q36_DeepSeek

question_id Q36

model DeepSeek

answer_text:

Based on the available data, the answer is yes: mpox can be significantly more serious in children. While mpox is often a self-limiting illness, multiple global health authorities and studies have identified children, particularly young children, as a high-risk group for severe disease, complications, and death-

Higher Rates of Infection and Mortality in Children

The data from recent outbreaks, particularly those involving the more severe clade I virus, shows a stark reality for children:

Disproportionate Infection Rates: During the 2024 clade I outbreak, 67% of confirmed cases were in children under 15 years old-1. In the Democratic Republic of Congo (DRC), children under 15 accounted for approximately 70% of cases

Significantly Higher Mortality: A systematic review and meta-analysis published in 2024 provided a pooled estimate, finding a case fatality ratio of 11% (95% CI 4-20) in children--39. This risk is not uniform across all age groups; infants under one year old face a case fatality rate of 8.6%, compared to 2.4% in those aged 15 and older.

Mpox Clades and Children's Susceptibility

The severity of mpox in children is closely linked to the virus clade and the age of the child.

The Role of Virus Clades: There are two main genetic groups, or clades, of the mpox virus. Clade I is more virulent and is associated with significantly higher rates of severe illness and death in children-1-6. Clade II, which caused the 2022 global outbreak, typically causes less severe disease in children-

Higher Risk in Younger Children: Age is a critical factor. Children under 5 years old are at the highest risk for severe disease and complications-2-21-31. The risk is particularly acute for infants, including newborns-.

Greater Risk of Complications in Children

Infection can lead to more severe outcomes in pediatric cases due to several factors:

Underdeveloped Immune Systems: Young children have developing and less robust immune systems compared to healthy adults, making them more vulnerable to severe infection--2. Other key risk factors for severe disease include eczema and other chronic skin conditions, malnutrition, and immunocompromising conditions like poorly controlled HIV-

Potential for Serious Complications: In severe cases, children can experience serious complications, including:

Secondary bacterial infections, especially of the skin lesions-

Respiratory distress, such as pneumonia-.

Life-threatening conditions like sepsis, a severe response to infection-

Brain inflammation, known as encephalitis (though this is rare)

Protection, Treatment, and Next Steps

Understanding the risks, several important steps can be taken to protect children.

Prevention Strategies: Reducing a child's risk of exposure is the most effective form of protection. The best ways to do this include keeping children away from individuals with a suspected or confirmed mpox infection and isolating infected household members. Post-exposure prophylaxis (PEP) with vaccination may be an option for children who have had close contact with an infected person, and should be discussed with a doctor

Treatment Support: Treatment for children with mpox is primarily supportive. This involves managing pain and fever, ensuring proper hydration to prevent dehydration, and caring for skin lesions to prevent secondary bacterial infections--2. The antiviral drug tecovirimat may be considered for those with severe disease or at high risk, but it is not an approved medication for mpox and is used on a case-by-case basis

Seek Timely Medical Care: It is crucial to seek medical attention as soon as possible if a child develops a rash, fever, and swollen lymph nodes, especially if they have been in contact with a confirmed case. Studies show that delaying care for more than seven days after symptoms begin is linked to higher disease severity

I hope this information is helpful. Please remember that this overview is for informational purposes.

record_id Q36_Doubao

question_id Q36

model Doubao

answer_text:

Yes, mpox can be more serious in children, especially infants and those with underlying vulnerabilities. The severity varies by age, virus clade, immune status, and access to care.

Key Severity Differences in Children

1. Higher Mortality Risk

Clade I (Congo Basin): Children <=4 years have CFR up to 15% vs. adults 1-3%

Global averages (2024): Under-5 CFR 3.2% vs. >=15 years 1.8% (almost double)

Infants <6 months: Highest risk-DRC data shows 50% of cases and all deaths in this group in some regions

WHO 2024: Children <=15 years account for 67-70% of cases and 83-85% of fatalities in some African outbreaks; child mortality is 4x higher than adults

2. More Severe Clinical Manifestations

Children, particularly younger ones, often experience:

More extensive/widespread skin lesions with higher risk of secondary bacterial infections (cellulitis, abscesses, necrotizing soft tissue infections)

Higher rates of complications including pneumonia, retropharyngeal abscesses, encephalitis, and sepsis

Ocular involvement (corneal lesions) that can lead to permanent vision loss

Fulminant disease in infants: multi-organ failure, hepatosplenic involvement, severe lymphadenopathy

Why Children Are More Vulnerable

Factor Explanation

Immune naivety Young children lack prior smallpox vaccination or natural exposure; immature immune systems less able to control orthopoxviruses

Skin barrier differences Thinner skin, higher surface area-to-body mass ratio, and conditions like eczema increase infection risk and severity

Higher exposure risk Close contact in households, daycare; more likely to touch/scratch lesions, increasing autoinoculation

Comorbidities Immunocompromised status, eczema, malnutrition, or concurrent infections amplify severity

Important Context: Clade & Outbreak Variations

Clade I (Congo Basin): Historically associated with greater severity in children; CFR up to 10% in children <10 vs. 1-3% in adultsAmerican Academy of Pediatrics

Clade II (West African): Generally milder; 2022-2023 global outbreak had <1.3% of cases in <18 years with 0 reported deaths in children; ICU admissions were rare

2024 DRC outbreak (Clade Ib): Severe resurgence with 5.3% CFR; children <=15 years dominated cases/fatalities

Most At-Risk Pediatric Groups

Infants <1 year (especially <6 months)

Children with eczema or other skin disorders

Immunocompromised children (HIV, cancer treatment, transplant recipients)

Unvaccinated children (no smallpox vaccine-derived immunity)

Children with malnutrition or concurrent infections

Summary

Mpox is more serious in children, particularly infants and those with underlying vulnerabilities, especially with Clade I/Ib viruses. While the 2022-2023 global outbreak (Clade II) saw mild pediatric cases, historical and recent African data confirm children face higher mortality and complication rates than adults. Early recognition, supportive care, and targeted prevention (vaccination for contacts, post-exposure prophylaxis) are critical for reducing pediatric severity.

record_id Q36_Qwen

question_id Q36

model Qwen

answer_text:

Yes, mpox (monkeypox) is consistently documented to be more severe in children, particularly in young children under 5-8 years of age, with significantly higher rates of complications, hospitalization, and death compared to adults. This disparity is especially pronounced for clade I (particularly the Ib variant) currently driving outbreaks in Africa, though children remain at elevated risk even with clade II infections. Below is a detailed analysis based on current evidence:

1. Higher Case Fatality Rates in Children

Children under 5 years account for 39% of confirmed clade I cases but 62% of mpox-related deaths in the Democratic Republic of the Congo (DRC), with mortality estimates as high as 8% in endemic regions. Infants and toddlers (1-4 years) face the highest risk of fatal outcomes compared to other age groups.

Globally, children's fatality rate is approximately four times higher than adults, with children representing over 70% of cases and 85% of deaths in current clade I outbreaks.

2. Key Risk Factors for Severe Disease in Children

a) Age-Specific Vulnerabilities

Immature immune systems reduce children's ability to control viral replication, leading to prolonged illness and higher viral loads.

Malnutrition and comorbidities (e.g., HIV coinfection) are more prevalent in pediatric populations in endemic regions like DRC, exacerbating disease severity.

b) Clade I vs. Clade II Severity

Clade I (Ib variant): Causes more severe disease in children, with higher viral loads and prolonged symptoms. In DRC, 61% of confirmed cases involve children/adolescents (<=15 years), with a median age of 9 years.

Clade II (IIb variant): Less lethal overall (case fatality rate <0.2%), but children still face elevated risks of complications like secondary infections, encephalitis, or pneumonia compared to adults.

c) Healthcare Access Barriers

In resource-limited settings (e.g., DRC), diagnostic delays, limited treatment access, and overcrowded living conditions (e.g., displacement camps) contribute to worse pediatric outcomes. Only 37-40% of suspected cases in DRC receive laboratory confirmation, suggesting true mortality is underestimated.

3. Common Complications in Pediatric Cases

Children are more likely to experience:

Severe skin complications: Extensive lesions, secondary bacterial infections, and scarring.

Systemic issues: Dehydration from oral lesions (56% of pediatric cases report swallowing difficulties), respiratory distress, or encephalitis.

Higher hospitalization rates: Though overall hospitalization rates for mpox are ~7-8%, children in endemic regions face longer stays and more intensive care needs due to complications.

4. Prevention and Treatment Considerations

Vaccination: The MVA-BN vaccine (JYNNEOS) is now approved for children as young as 12-17 years by WHO and EMA. For younger children, exposure-based risk assessment guides off-label use, though data remains limited.

Antiviral limitations: Tecovirimat (TPOXX), the primary antiviral, showed no significant clinical benefit for clade I in DRC trials, though supportive care reduced overall mortality to 1.7% (vs. 3.6% community rates).

Critical gaps: Children are excluded from most vaccine trials and often lack access to diagnostics/treatment in endemic areas, worsening outcomes.

In summary, children-especially those under 5-are disproportionately affected by severe mpox, with clade I infections posing the greatest threat. The combination of biological vulnerability, socioeconomic barriers, and limited pediatric-specific interventions underscores the urgent need for targeted vaccination campaigns, improved diagnostics, and age-appropriate treatment protocols in high-risk regions. For non-endemic countries, vigilance remains critical as clade I spreads beyond Africa.

1.!??- 2.mpox,?--

Q37. Can mpox during pregnancy affect the baby?

record_id Q37_ChatGPT

question_id Q37

model ChatGPT

answer_text:

Yes. Mpox during pregnancy can affect the baby, although not every infection will.

Mpox virus can be passed to the fetus during pregnancy, likely through the placenta, and can also be passed to the newborn during or after birth through close contact. Reported adverse outcomes include pregnancy loss, stillbirth, preterm birth, and severe infection in newborns, though the available pregnancy data are still limited.

For the pregnant person, symptoms are generally similar to other adults, but CDC says pregnant, recently pregnant, and breastfeeding people should be prioritized for treatment when needed because of the possible increased risks to both parent and baby.

Seek medical advice promptly from an OB/GYN, midwife, or urgent care clinician if you are pregnant and have had a possible exposure or symptoms such as a new blister-like or pimple-like rash, fever, swollen lymph nodes, body aches, or sores in the genital/anal area or mouth. They can discuss testing, isolation, treatment options, and fetal monitoring.

record_id Q37_Gemini

question_id Q37

model Gemini

answer_text:

Yes, mpox infection during pregnancy can significantly affect the fetus and the newborn. The mpox virus (MPXV) is capable of crossing the placenta (vertical transmission), and it can also infect the infant through close contact during or immediately after delivery (perinatal transmission).

Because of the immunological and hormonal shifts during gestation, pregnant individuals face an increased risk of severe illness, and the virus is now increasingly viewed as a congenital pathogen.

Primary Fetal and Perinatal Risks

Fetal Loss and Stillbirth: Infection carries a high risk of spontaneous abortion (miscarriage) and intrauterine fetal demise. Recent cohort data indicates that fetal loss is exceptionally frequent when maternal infection occurs during the first trimester.

Congenital Mpox Syndrome: Fetuses infected in utero can develop severe clinical manifestations. Pathological evaluations of affected fetuses have revealed diffuse cutaneous maculopapular or vesicular lesions (similar to adult skin lesions), hepatomegaly (enlarged liver), hydrops fetalis (severe fluid accumulation), and chorioamnionitis.

Preterm Birth and Neonatal Mortality: Severe maternal infection can trigger premature labor. Neonates born with congenital mpox face elevated mortality and morbidity due to immature immune systems and potential systemic, respiratory, or neurological complications.

Pathogenicity by Viral Clade

The severity of fetal outcomes depends heavily on the specific viral clade involved:

Feature / Impact Clade I (including Ia and Ib) Clade II (including IIb)

Epidemiological Context Endemic to Central Africa; driven recent regional outbreaks (e.g., Clade Ib). Responsible for the 2022-2023 global outbreak.

Maternal & Fetal Severity High. Associated with high viral loads and systemic complications. Generally Milder. Lower overall case fatality and maternal morbidity.

Risk of Fetal Loss Severe. Prospective data shows adverse outcomes in up to 50% of pregnancies, soaring to over 90% in first-trimester infections. Lower. Human case registries show significantly fewer adverse perinatal outcomes, though the risk remains present.

Vertical Transmission Strongly confirmed via molecular and histopathological evidence in placental tissues. Documented in animal models (rhesus macaques) and rare human case reports.

Clinical Management and Prevention

Because of these risks, international public health guidelines recommend specific protocols for pregnant patients:

Prioritization of Therapeutics: Pregnant individuals are prioritized for antiviral treatments (such as tecovirimat) if clinically indicated, due to the high risk of severe maternal-fetal escalation.

Safe Vaccination: When exposure risk is high, non-replicating vaccines (such as JYNNEOS) are preferred. Animal studies and limited human data have shown no evidence of fetal harm or vaccine-associated developmental risks.

Postpartum Separation: To safeguard the neonate from high-consequence horizontal transmission via skin-to-skin contact, clinical guidelines often recommend temporary separation or strict contact precautions between an infectious mother and her newborn until all maternal lesions have fully healed.

record_id Q37_Copilot

question_id Q37

model Copilot

answer_text:

Short answer: Yes - mpox (monkeypox) during pregnancy can harm the fetus or newborn. Reports show probable vertical (in-utero) transmission, pregnancy loss, preterm birth, and severe neonatal infection; evidence is limited but concerning, so contact a clinician urgently if you are pregnant and exposed or symptomatic.

What the evidence shows

Vertical transmission has been documented. Viral DNA and infection have been detected in fetal tissues, placenta, and umbilical cord in reported cases, demonstrating that the virus can cross the placenta.

Adverse pregnancy outcomes have been reported. Case series and systematic reviews report spontaneous miscarriage, intrauterine fetal demise, preterm birth, and neonatal death in pregnancies affected by mpox. The pooled estimates are imprecise because sample sizes are small, but the proportions of adverse outcomes are high in the available reports.

Risks to the baby and newborn

Fetal loss and miscarriage: Reports include first- and second-trimester losses and stillbirths after maternal infection.

Congenital infection: Newborns have been born with generalized rash and evidence of infection; some neonates have required intensive care and have died.

Preterm delivery and neonatal severe disease: Maternal illness has been associated with preterm birth and severe neonatal mpox when transmission occurs around delivery.

How certain is this evidence

Limited but consistent signals: Most data come from case reports, small case series, and recent cohort work; this limits precision and generalizability. However, multiple independent reports and reviews converge on the conclusion that mpox can cause serious pregnancy and neonatal outcomes.

What to do if pregnant and exposed or symptomatic

Seek medical care promptly. Pregnant people with suspected exposure or symptoms should contact their obstetric provider or local public health authority immediately for evaluation and testing. Prioritize clinical assessment because pregnancy may increase risk of severe disease and because treatment and infection-control decisions affect both mother and baby.

Testing and management decisions (including antiviral therapy, vaccination, and delivery planning) should be made by clinicians experienced in maternal-fetal medicine and infectious disease; these choices depend on timing in pregnancy, severity of maternal illness, and local guidance.

Prevention and planning

Avoid exposures and follow public-health guidance about mpox transmission (close contact with lesions, respiratory secretions, contaminated materials). Vaccination and post-exposure prophylaxis policies vary; discuss risks and benefits with your clinician.

Key takeaways

Mpox during pregnancy can affect the baby through vertical transmission and is associated with serious outcomes. Evidence is limited but consistent enough to warrant urgent clinical evaluation for any pregnant person with exposure or symptoms.

Fetal and maternal outcomes following mpox infection in pregnancy - The Lancet

Mpox in Pregnancy - Risks, Vertical Transmission, Prevention, and Treatment

record_id Q37_DeepSeek

question_id Q37

model DeepSeek

answer_text:

Yes, mpox during pregnancy can severely impact the baby. Evidence shows the virus can be transmitted from mother to fetus in the womb, leading to high rates of serious adverse outcomes, with first-trimester infections posing the greatest risk-.

Complications for the Baby

Data shows that infection during pregnancy can lead to a range of serious complications for the baby and the pregnancy:

High Overall Risk: A meta-analysis found that approximately 63% of pregnancies with mpox infection resulted in at least one adverse outcome

Fetal Loss (Miscarriage & Stillbirth): A large study in the DRC found 45% of pregnancies with mpox infection ended in fetal loss-2. This is also supported by the meta-analysis, which found a rate of 62% for miscarriage or fetal death

Congenital Infection (Mpox at Birth): Babies can be born with mpox, known as congenital infection. In the DRC study, 10.5% (4 out of 38) of live births resulted in neonates with mpox-like lesions at birth

Other Risks: Other documented risks include congenital anomalies (50%), chorioamnionitis (78%), and preterm birth (especially noted with the Clade I virus)

Understanding the Key Risks

The risk to the baby is influenced by a few key factors:

Viral Clade (strain): The Clade I strain, particularly the more severe Clade Ib variant, is clearly linked to the most severe pregnancy outcomes-. The data for the milder Clade II strain is less robust-.

Trimesters: The risk of transmission and adverse outcomes is not the same throughout pregnancy.

First Trimester: This is the period of highest vulnerability, with the strongest association with fetal loss and severe outcomes

Placental Transmission: The virus has been detected in placental tissue, confirming its ability to cross the placenta and infect the fetus

Maternal Health: Pregnant individuals with HIV co-infection may be at a higher risk of severe disease and adverse outcomes

Impact on the Mother

While the primary focus is often on the baby, mpox during pregnancy also poses significant health risks to the mother. Pregnant women are considered a vulnerable group who are more likely to develop severe illness-25. Reports indicate a 25% risk of severe maternal disease and, in some cases, high mortality and morbidity-

Prevention and Care

Recognizing the significant risks, pregnant women are considered a high-priority group for mpox prevention and management.

Vaccination: The CDC recommends that pregnant women who have been exposed to the mpox virus get vaccinated within 4 days of exposure to potentially prevent or mitigate the disease

Antiviral Medication: If treatment is needed, tecovirimat is considered the first-line antiviral for pregnant women-.

Active Monitoring: Close fetal surveillance and perinatal care are crucial for managing pregnancies affected by mpox to improve outcomes-

While research is still evolving, the data is clear: mpox during pregnancy, especially with the Clade I virus, carries a high risk of severe adverse outcomes for the baby. Pregnant women should follow public health guidelines to protect themselves.

record_id Q37_Doubao

question_id Q37

model Doubao

answer_text:

Yes, mpox (monkeypox) during pregnancy can absolutely affect the fetus and newborn baby, with potential serious risks. Here are the key impacts and risks:

1. Mother-to-child (vertical) transmission

The mpox virus can spread to the baby in three ways:

During pregnancy (in utero): Virus crosses the placenta to infect the fetus.

During childbirth: Direct contact with infectious lesions as the baby passes through the birth canal.

After birth: Close skin-to-skin contact or contact with contaminated bodily fluids/lesions.

2. Risks to the baby

Increased chance of miscarriage, stillbirth, or preterm birth.

Congenital mpox infection: The baby is born with mpox lesions, fever, and possible damage to internal organs. Newborns have weak immune systems, so this infection can be life-threatening.

Low birth weight.

3. Risks to the pregnant parent

Pregnancy can weaken immune defenses, making mpox symptoms more severe and raising the risk of complications like secondary bacterial skin infections or respiratory issues.

Key guidance

Pregnant people with mpox exposure or suspected infection need immediate medical assessment. Doctors will provide close monitoring, isolation, and tailored supportive care; specific antivirals may be used if the benefits outweigh risks. Mpox vaccination is generally avoided in routine pregnancy but may be considered for high-risk exposure under medical advice.

record_id Q37_Qwen

question_id Q37

model Qwen

answer_text:

Yes, mpox (monkeypox) during pregnancy can significantly affect the baby, with risks including vertical transmission from mother to fetus, adverse pregnancy outcomes (such as miscarriage, stillbirth, or preterm birth), and neonatal infection after delivery. The severity of these risks depends largely on the viral clade (I vs. II) and the mother's clinical condition.

1. Vertical Transmission to the Fetus

Mpox virus can cross the placenta and infect the fetus during pregnancy, with placental trophoblast cells serving as targets for viral replication .

This transmission may lead to congenital mpox infection, which has been linked to severe fetal complications, including widespread skin lesions, organ damage, and fetal demise .

2. Adverse Pregnancy Outcomes

Miscarriage and Stillbirth

Studies report up to 50% fetal loss rates in pregnancies affected by clade I (formerly Congo Basin) mpox virus. For example:

A 2023-2024 Democratic Republic of Congo outbreak study found 4 out of 8 pregnancies ended in miscarriage or stillbirth .

Historical data from 2001-2011 showed 3 out of 4 pregnant women with mpox experienced fetal death or miscarriage, with histological evidence confirming intrauterine viral transmission .

Preterm Birth

Premature delivery occurs in ~15-20% of cases, often due to maternal complications or clinician decisions to expedite delivery for maternal/fetal safety

3. Neonatal Infection After Birth

Newborns can contract mpox during delivery (via contact with maternal lesions) or postpartum through close contact with infectious skin/fluids.

Documented cases include:

Two infants in a U.S. study developed mpox within 2 weeks of birth after their mothers tested positive post-delivery. Both required antiviral treatment (tecovirimat), though outcomes were favorable .

In another report, 2 out of 6 live-born infants developed mpox-related rashes within 10 days of birth .

Neonatal mpox carries higher risks of severe disease, including skin complications, sepsis, and prolonged illness .

4. Differences by Viral Clade

Clade I (more severe): Associated with higher rates of fetal loss (up to 50%) and severe neonatal outcomes. This clade is predominant in Central Africa and has driven recent outbreaks with elevated mortality .

Clade II (less severe): Linked to milder pregnancy outcomes in global outbreaks (e.g., 2022-2023). One study of 10 clade II cases reported no vertical transmission, though others noted limited fetal/newborn complications .

5. Management Considerations

Antiviral treatment: Tecovirimat (TPOXX) has been safely used in pregnant patients without reported adverse effects, potentially reducing transmission risk .

Fetal monitoring: Regular ultrasounds and non-stress tests are recommended to detect complications like fetal growth restriction or distress .

Delivery planning: For mothers with active genital lesions, cesarean delivery may be advised to reduce intrapartum transmission, though prenatal transmission might already have occurred .

Newborn isolation: Infants born to infected mothers should be temporarily separated and monitored for symptoms until maternal infectiousness resolves .

Key Takeaway

While not all pregnancies with mpox result in poor outcomes, clade I infection poses substantial risks to the fetus, including miscarriage, stillbirth, and severe neonatal disease. Early diagnosis, antiviral therapy, and specialized obstetric care significantly improve prognosis. Pregnant individuals with mpox exposure or symptoms should seek immediate medical evaluation.

Q38. Is mpox more dangerous for people living with HIV?

record_id Q38_ChatGPT

question_id Q38

model ChatGPT

answer_text:

Yes - mpox can be more dangerous for people living with HIV, especially when HIV is advanced or not well controlled.

For people with well-controlled HIV - on ART, viral load suppressed, and CD4 count not severely low - mpox usually behaves more like it does in people without HIV and often resolves with supportive care. But people with advanced or uncontrolled HIV, particularly CD4 counts under 200 cells/mm3, have a higher risk of prolonged, severe, disseminated, or life-threatening mpox, including hospitalization and death.

The CDC says it is not known whether HIV itself makes someone more likely to catch mpox after exposure, but recent outbreaks show higher risk of severe outcomes in people with uncontrolled or advanced HIV.

Practical steps: stay on HIV meds, keep viral load suppressed, get the two-dose JYNNEOS mpox vaccine if eligible, and contact a clinician promptly if exposed or if symptoms appear. CDC considers JYNNEOS safe for people with HIV, and WHO recommends rapid ART initiation for people diagnosed with both HIV and mpox; people already on ART with suppressed viral load should continue treatment.

Seek medical care quickly if mpox is suspected and you have HIV, especially with a low CD4 count, uncontrolled viral load, widespread/necrotic lesions, eye symptoms, severe rectal/genital pain, trouble urinating, confusion, breathing symptoms, or worsening illness.

record_id Q38_Gemini

question_id Q38

model Gemini

answer_text:

The short answer is it depends entirely on how well the HIV is managed.

Well-Controlled HIV vs. Advanced HIV

Well-Controlled HIV: People who are consistently taking antiretroviral therapy (ART), have a fully suppressed viral load, and maintain a healthy immune system (a normal CD4 count) do not appear to be at a higher risk for severe mpox. Their symptoms, progression, and recovery are generally identical to those of someone without HIV.

Advanced or Uncontrolled HIV: People with a low CD4 count (particularly below 200 cells/microL) or an unsuppressed viral load face a significantly higher risk of severe illness, prolonged disease, hospitalization, and death.

Why Advanced HIV Changes the Risk

When the immune system is severely weakened by advanced HIV, the body struggles to keep the mpox virus in check. In these cases, clinical data shows that mpox can behave like a completely different, much more aggressive disease:

Severe, Necrotizing Lesions: Instead of a few localized sores, the rash can become widespread, confluent, and necrotizing (causing extensive tissue and skin death).

Organ Involvement: The virus is much more likely to spread internally, leading to severe complications like lung disease (pneumonia), secondary bacterial infections, or inflammation of the brain.

Higher Mortality Rates: Studies have shown that for individuals with advanced immunosuppression (CD4 counts under 200), the mortality rate can sit around 15%, and it approaches 30% for those with CD4 counts below 100. Because of this extreme severity, global health experts have classified disseminated, necrotizing mpox as an AIDS-defining opportunistic infection.

Prevention and Protection

There are clear, highly effective strategies to mitigate these risks:

Consistent HIV Treatment: Staying on HIV medication to maintain an undetectable viral load is the single best way to ensure the immune system is strong enough to handle an mpox exposure.

Safe Vaccination: The primary mpox vaccine (JYNNEOS) is a non-replicating virus vaccine. This makes it completely safe and highly effective for people living with HIV, including those who are immunocompromised.

Dual Testing: Because the stakes are higher for uncontrolled HIV, health organizations recommend that anyone seeking care for a suspected mpox infection also be tested for HIV if their status is unknown.

Important Note: If you or someone you know is living with HIV and suspects an exposure to mpox, it is crucial to consult a healthcare provider right away. They can fast-track a CD4 count check, discuss the mpox vaccine, or evaluate the need for specialized antiviral treatments.

record_id Q38_Copilot

question_id Q38

model Copilot

answer_text:

Short answer: Yes - mpox can be more severe for people living with HIV, especially those with advanced or uncontrolled HIV (low CD4 counts or detectable viral load). People with well-controlled HIV on effective antiretroviral therapy (ART) usually have outcomes similar to people without HIV.

Key evidence summary

Higher risk when HIV is advanced or uncontrolled: Multiple case series and guideline reviews found more hospitalizations, complications (necrotizing skin lesions, lung involvement, secondary infections), and deaths among people with CD4 counts \<200 cells/mm3, with the worst outcomes at \<100 cells/mm3.

Well-controlled HIV reduces excess risk: People living with HIV who are on ART with suppressed viral load and higher CD4 counts generally had clinical courses similar to HIV-negative patients.

Vaccination and antivirals help: Smallpox/mpox vaccines and antiviral treatment (e.g., tecovirimat) are effective tools; vaccine effectiveness was high in cohort analyses and guidelines recommend vaccination and early treatment for higher-risk people.

Quick comparison (risk by HIV status)

Group Typical immune status Relative mpox severity Key concerns

Well-controlled HIV On ART; CD4 >=350; suppressed viral load Similar to HIV-negative Routine care; vaccination recommended.

Partially controlled On ART but lower CD4 (200-349) or intermittent viremia Moderately increased Higher hospitalization risk; monitor closely.

Advanced/uncontrolled HIV Not on ART or CD4 \<200; detectable viral load Markedly increased Higher rates of severe complications and death; urgent care and antivirals advised.

What this means and practical steps

If you or someone you care for has HIV: Staying on or starting ART and achieving viral suppression is the single most important way to lower the risk of severe mpox.

Prevention: JYNNEOS (smallpox/mpox vaccine) is recommended for people at higher risk and is considered safe for people with HIV; vaccine reduces risk of mpox.

If infected: Seek medical care early - clinicians may consider antivirals (tecovirimat) and closer monitoring for people with low CD4 counts or other immunosuppression.

Bottom line

Mpox is more dangerous mainly for people with advanced or uncontrolled HIV; with effective ART, vaccination, and timely medical care the excess risk is substantially reduced.

Mpox in people with advanced HIV infection: a global case series - The Lancet

Mpox in People With HIV: Predictors of Diagnosis, Outcomes, and Vaccine Effectiveness in a Multisite Cohort Clinical Infectious Diseases OXFORD

record_id Q38_DeepSeek

question_id Q38

model DeepSeek

answer_text:

Yes, mpox (formerly known as monkeypox) can be more dangerous for people living with HIV, specifically for those who have a low CD4 count and are not on effective treatment. This is a key concern for global health organizations like the WHO and CDC.

Key Risks of Mpox for People with HIV

The primary factor determining the severity of mpox in people living with HIV is the level of immune suppression, typically measured by the CD4 cell count.

Risk of Severe Disease and Death: The most significant danger is for individuals with advanced or poorly controlled HIV. For these individuals, mpox acts as an opportunistic infection and carries a high risk of severe illness and death-1-31-3. Studies have shown that a large percentage of severe mpox cases and related deaths occur in people with HIV, particularly those with very low CD4 counts-

Hospitalization Rates: People living with HIV who are severely immunosuppressed have a much higher chance of being hospitalized for mpox compared to those with healthy immune systems or HIV under control-32. For example, hospitalization rates can reach nearly 30% in individuals with a CD4 count below 100 cells/microL-.

Protective Effect of HIV Treatment: This risk is dramatically lower for people whose HIV is well-managed on antiretroviral therapy (ART) with a healthy CD4 count. Their risk of severe mpox is generally similar to that of people without HIV

Comparing Mpox Virus Clades

The danger also depends on which type of mpox virus is causing the infection. There are two main clades, with different levels of severity:

Clade I (Historically more severe): Found in Central Africa, associated with more severe illness and higher overall death rates

Clade II (Historically less severe): Responsible for the 2022 global outbreak, with a lower overall death rate (<1%)

The added danger for immunocompromised individuals is present with both clades--11. Recent global health emergencies have been declared due to both clade IIb in 2022 and new clade Ib outbreaks in Africa

What People Living with HIV Should Do

For those living with HIV, taking these steps can significantly reduce the risk:

Seek Medical Advice Immediately: If you develop mpox symptoms or have been in contact with someone who has mpox, speak with a healthcare provider right away

Start or Continue ART Promptly: If not on treatment, rapid initiation of ART (within 7 days) is strongly recommended-22. The WHO states that early treatment of HIV is even more critical for those with mpox

Access Vaccination if Offers: Smallpox/mpox vaccines are effective and recommended as pre-exposure prophylaxis for people with HIV who may be at high risk of mpox exposure.

If you are living with HIV, effectively managing the condition with ART is the most powerful step you can take to minimize the risk of severe mpox. This protection is similar to the immune support that helps prevent other opportunistic infections in many people.

record_id Q38_Doubao

question_id Q38

model Doubao

answer_text:

Yes, mpox is more dangerous for people living with HIV (PLWH), but this risk is strongly tied to HIV disease stage and treatment status.

Key Risk Distinctions

HIV Status Mpox Risk Level Clinical Outcomes

Advanced/untreated HIV (CD4 <200 cells/mm3, high viral load, not on ART) Significantly elevated Higher hospitalization rates (OR=2.84, 95% CI: 1.68-4.79), progressive/disseminated rash, protracted illness, complications (sepsis, ocular disease, encephalitis), mortality up to 15-27%World Health Organization (WHO)

Virally suppressed HIV (on effective ART, CD4 >200 cells/mm3) Similar to general population Mild, self-limiting illness, no increased risk of severe disease or death

Why the Difference Matters

Severe mpox in PLWH is primarily driven by immunocompromise from untreated HIV, which impairs the body's ability to control the virus.

Viral suppression through antiretroviral therapy (ART) restores immune function, eliminating the elevated risk of severe mpox.

Most fatal mpox cases in PLWH occur in those with unknown HIV status prior to mpox diagnosis or those not engaged in HIV care.

Clinical Implications

CDC specifically identifies PLWH with CD4 <200 cells/mm3 as being at high risk for severe mpox and recommends prioritized antiviral treatment.

WHO and CDC advise immediate ART initiation for PLWH diagnosed with mpox who are not already on treatment.

Conclusion

Mpox is not inherently more dangerous for all PLWH. The critical factor is HIV control: individuals with well-managed HIV through effective ART and viral suppression face similar risks to the general population, while those with advanced, untreated HIV experience significantly higher rates of severe disease and death.

record_id Q38_Qwen

question_id Q38

model Qwen

answer_text:

Mpox (monkeypox) is not inherently more dangerous for all people living with HIV, but it becomes significantly more severe and life-threatening for those with advanced HIV disease (CD4+ count <200 cells/microL) or uncontrolled viral replication due to untreated/poorly managed HIV. For individuals with HIV who maintain effective antiretroviral therapy (ART) and CD4+ counts >350 cells/microL, mpox severity and outcomes are generally comparable to HIV-negative individuals. The key determinant of risk is immune status, not HIV infection alone.

1. Critical Role of Immune Status

a) CD4+ Count Thresholds

CD4+ <200 cells/microL: This group faces markedly higher risks of severe mpox, including systemic spread, prolonged illness (beyond 4 weeks), and death. Studies report mortality rates of 27-38% in this subgroup due to complications like necrotizing pneumonia, multi-organ failure, and secondary infections .

CD4+ 200-350 cells/microL: Moderate risk of atypical presentations (e.g., widespread necrotic skin lesions, proctitis), but outcomes improve significantly with prompt ART initiation .

CD4+ >350 cells/microL with viral suppression: Disease severity and viral clearance rates mirror those of HIV-negative individuals, as effective ART preserves immune function .

b) ART Adherence Matters

Untreated HIV or interrupted ART correlates with 8.3-fold higher hospitalization rates for mpox compared to virally suppressed patients. Early ART resumption (within 7 days of mpox diagnosis) reduces complications by restoring immune control .

In one study, 71.4% of ART-adherent HIV+ patients recovered fully from mpox, versus 50% mortality in untreated cases .

2. Clinical Severity and Mortality Evidence

a) High-Risk Groups

Advanced HIV (CD4+ <50 cells/microL): Nearly all fatal mpox cases occur in this group. A Beijing study found 100% mortality (6/6 cases) in late-stage HIV patients (CD4+ <51 cells/microL), with median survival of 59 days from symptom onset due to sepsis and multi-organ failure .

Systemic complications: Low CD4+ counts enable mpox to spread beyond skin/mucosa, causing pneumonia (38.1% mortality), encephalitis, and bacteremia. Viral loads in skin lesions remain elevated longer, delaying healing .

b) Conflicting Data Explained

Some studies (e.g., a Spanish cohort) found no significant difference in severity between HIV+ and HIV- patients. However, this cohort had median CD4+ counts >400 cells/microL and high ART adherence, highlighting that immune status-not HIV status alone-drives outcomes .

Global data consistently shows worse outcomes when CD4+ counts are low: In U.S. hospitalizations, 27% of uncontrolled HIV patients required hospitalization versus 3% of HIV-negative cases .

3. Key Prevention and Management Strategies

a) For HIV+ Individuals

Prioritize ART adherence: Maintaining viral suppression is the most effective way to reduce mpox severity. WHO recommends starting ART within 7 days of mpox diagnosis for untreated HIV .

Vaccination: Mpox vaccines (e.g., JYNNEOS) show strong efficacy in HIV+ individuals with CD4+ >200 cells/microL. Vaccination is especially critical for those with lower CD4+ counts, though immune responses may be weaker .

b) Clinical Monitoring

High-risk patients (CD4+ <200 cells/microL) require aggressive monitoring for systemic spread (e.g., chest imaging for pneumonia) and early intervention for secondary infections.

Avoid misattribution: Mpox symptoms in advanced HIV may mimic other opportunistic infections (e.g., molluscum contagiosum or fungal diseases), necessitating PCR testing for confirmation .

In summary, mpox severity in HIV+ individuals depends critically on immune preservation through ART. Those with well-controlled HIV face minimal added risk, but untreated or advanced HIV dramatically increases mortality. Public health efforts should focus on HIV testing, ART access, and targeted mpox vaccination for immunocompromised populations.

Q39. Can a breastfeeding mother with suspected mpox continue breastfeeding?

record_id Q39_ChatGPT

question_id Q39

model ChatGPT

answer_text:

It depends on which guidance you're following, and the infant-feeding setting.

In the U.S., CDC guidance says a mother with suspected or confirmed mpox should not directly breastfeed while symptomatic or in isolation. Breastfeeding should be delayed until mpox is ruled out or, if confirmed, until all lesions have resolved, scabs have fallen off, and a fresh layer of intact skin has formed. CDC also says expressed milk from someone symptomatic or isolated should be discarded, and a healthy caregiver can feed pasteurized donor milk or formula.

WHO's 2025 living guideline is different: it conditionally suggests that mothers with mpox may continue breastfeeding while limiting direct contact with the uninfected infant and using infection-prevention measures, because the recommendation weighs breastfeeding benefits against uncertain transmission risk. WHO says precautions should include limiting contact except during feeds, covering lesions, handwashing, wearing a medical mask, and not breastfeeding from a breast with areolar lesions.

For a suspected case, the safest immediate step is to contact a clinician/public health service urgently, arrange testing, and follow local guidance. Until mpox is ruled out, treat it as potentially infectious: avoid skin-to-skin contact with the baby, cover lesions, wear a mask, wash hands, and have a healthy caregiver provide feeds when following CDC-style guidance. Infants and newborns can have severe mpox, so this should be handled promptly with medical support.

record_id Q39_Gemini

question_id Q39

model Gemini

answer_text:

Overview of Public Health Guidance

Whether a mother with suspected or confirmed mpox can continue breastfeeding depends on the specific public health guidelines being followed. Because mpox spreads primarily through close, skin-to-skin contact and respiratory droplets, and newborns face a higher risk of severe illness, health organizations emphasize strict safety measures.

CDC Recommendations (United States)

The Centers for Disease Control and Prevention (CDC) takes a highly precautionary approach to protect newborns from potential severe infection:

Temporary Pause: The CDC recommends that direct breastfeeding be delayed or suspended during the mother's infectious period.

Clearance Criteria: Direct breastfeeding should only resume once criteria for discontinuing isolation are fully met-meaning all skin lesions have completely healed, scabs have fallen off, and a fresh layer of intact skin has formed.

Discarding Expressed Milk: It is currently unknown if the virus can be transmitted directly through breast milk. However, due to the proximity required to pump and handle milk, the CDC advises that breast milk expressed while a mother is symptomatic or isolated should be pumped and discarded to maintain milk supply without feeding it to the infant.

WHO Recommendations (Global)

The World Health Organization (WHO) offers guidance that balances the risk of viral transmission against the health risks of interrupting breastfeeding (such as malnutrition or lack of clean water for formula), particularly in resource-limited settings:

Conditional Breastfeeding: If the mother has suspected or confirmed mpox but does not have any lesions on her breasts or areolas, she may continue breastfeeding, provided rigorous infection control steps are taken.

When to Stop: If there are active lesions on the breast or areola, direct breastfeeding from that side must stop immediately, and any milk expressed from that breast must be discarded. If only one breast is affected, feeding from the unaffected breast may continue.

Strict Precautions Required

If a mother continues breastfeeding under these circumstances, the following safeguards must be followed:

Wear Protection: The mother must wear a well-fitting medical mask and clean gloves during feeds.

Eliminate Skin Contact: Both the mother and the infant should be fully clothed or swaddled so that the infant cannot accidentally touch any rash or lesions.

Practice Hand Hygiene: Wash hands thoroughly with soap and water before and after any contact with the infant.

Important Next Steps: Anyone experiencing symptoms or suspected exposure to mpox should isolate from others and contact a healthcare provider immediately. A doctor can provide a definitive diagnosis and help navigate local medical protocols, while a lactation consultant can offer specialized support to safely manage or protect a mother's milk supply during recovery.

record_id Q39_Copilot

question_id Q39

model Copilot

answer_text:

Short answer: Yes - if a breastfeeding mother is only suspected of mpox but has no symptoms, current guidance supports continuing breastfeeding while monitoring the infant. If the mother has symptoms (especially lesions on the breast or active skin lesions anywhere), direct breastfeeding should be avoided and alternative feeding/expressing strategies used until a clinician advises otherwise.

Summary of the evidence and expert guidance

Asymptomatic exposed mothers: WHO-based guidance and infant-feeding emergency FAQs advise that mothers who were exposed but show no symptoms should continue breastfeeding, because the benefits of breastmilk outweigh the unknown risk of transmission via milk.

Symptomatic mothers or breast lesions: If a mother has confirmed or suspected mpox with symptoms, especially lesions on the breast or nipples, direct skin-to-skin contact and breastfeeding are not recommended until lesions have healed and a clinician clears breastfeeding. Expressed milk may be considered if handled safely.

Priority for treatment and evaluation: Pregnant, recently pregnant, and breastfeeding women are prioritized for clinical assessment and treatment when needed because of potential risks to the newborn.

Practical steps a mother with suspected mpox should take

Contact a healthcare provider immediately for testing and individualized advice; prioritize clinical evaluation.

If no symptoms: continue direct breastfeeding, monitor the infant closely for any signs of illness, and follow local public-health infection-control advice (hand hygiene, cover any lesions on mother's body).

If symptoms or breast lesions: avoid direct breastfeeding from the affected breast(s). Consider expressing milk with strict hand and pump hygiene and have a healthy caregiver feed the infant, or use safe alternatives if advised by clinicians. Expressed milk handling should follow infection-control guidance.

Ensure the infant is evaluated promptly if the mother is symptomatic or tests positive.

Risks, tradeoffs, and why guidance leans toward continuing breastfeeding

Breastfeeding provides important protection and nutrition; separation or stopping breastfeeding can increase infant risk from other causes. Because evidence about mpox in breastmilk is limited, guidance balances known breastfeeding benefits against uncertain transmission risk and therefore favors continuation when the mother is asymptomatic.

If you or someone you care for is in this situation, seek medical evaluation now so clinicians can test, advise on safe feeding options, and arrange follow-up for the infant.

FREQUENTLY ASKED QUESTIONS:Breastfeeding in the context of mpox - information for health workerst IYCF

Mpox and breastmilk: for once, can we act in time? - The Lancet

Monkeypox Clinical Care and Treatment During Pregnancy Monketypox CDC

record_id Q39_DeepSeek

question_id Q39

model DeepSeek

answer_text:

Based on the most up-to-date and authoritative guidelines, a mother with suspected mpox is generally recommended to continue breastfeeding her infant, provided she can take precautions to limit direct contact. This recommendation is based on the significant health benefits of breastmilk, which must be balanced against the still-uncertain transmission risk.

The table below summarizes key guidance on breastfeeding in the context of mpox.

Organization Recommendation Key Rationale

World Health Organization (WHO) Mothers with mpox should continue breastfeeding while limiting direct physical contact with their non-infected infant. This is a conditional recommendation based on low-certainty evidence.- Preserves the protective benefits of breastfeeding while taking precautions against contact transmission.

Africa CDC Breastfeeding should continue, even for suspected mpox cases, unless viral antibodies are detected in the milk itself.- Prioritizes infant nutrition and immunity, as the risk of transmission via breastmilk is not the primary concern.

WHO (for recovered mothers) For mothers who previously stopped breastfeeding, feeding should resume as soon as their lesions have healed.- After healing from a confirmed infection, the risk of transmission is considered past.-

US CDC (considerations) Recommends that eligible pregnant or breastfeeding women be offered antiviral treatment.- Balances maternal health needs with unknown risks, based on a shared decision-making model.

A Few Important Considerations

Understanding the Main Risk: The transmission of mpox from mother to infant is believed to occur primarily through direct contact with skin lesions, respiratory droplets, and contaminated bedding or clothing.- This is why the WHO's core advice on "limiting direct contact" is so important.

Unknowns about Breastmilk: It is still not known for certain whether mpox can be transmitted through breastmilk itself.-

If You Have Sores on Your Breasts: The presence of skin lesions on or near the breast or nipple raises a potential concern for contamination of expressed or pumped milk.- In this specific situation, current guidelines suggest that expressed breast milk, with careful hygiene and proper sanitization, may be a safer alternative to direct breastfeeding.-

Importance of Isolation: To reduce the risk of transmission, the infected mother should isolate herself from her infant as much as possible. Healthy family members should be involved in the infant's care to minimize the baby's contact with the infected parent.

Ultimately, the decision should be made in close consultation with your healthcare provider, who can assess your specific case and help you weigh the risks and benefits to come up with a plan that works best for you and your baby.

I hope this helps you have a clearer and more informed discussion with your doctor. Please remember that this information is not a substitute for professional medical advice.

record_id Q39_Doubao

question_id Q39

model Doubao

answer_text:

Core Conclusion

It is NOT recommended to continue direct breastfeeding if a breastfeeding mother has suspected mpox. Immediate temporary suspension of direct breastfeeding and separation from the infant are advised.

Key Rationale

Multiple transmission risks

Mpox spreads via close skin-to-skin contact, respiratory droplets, body fluids, and lesion secretions. Direct breastfeeding involves intimate contact with the baby; any rash/lesions on the breast, chest, or mother's body can easily transmit the virus to the infant (infants are more vulnerable to severe mpox symptoms).

Virus presence in breast milk

Mpox virus has been detected in human breast milk. There is a potential risk of viral transmission through oral intake of breast milk.

Official Recommended Measures

Stop direct breastfeeding right away and implement mother-infant isolation; let a healthy caregiver take care of the baby.

The mother may pump breast milk to relieve breast engorgement, but pumped breast milk should be discarded completely-do not feed it to the baby.

The mother should wear a high-standard mask, disposable gloves, practice strict hand hygiene, and disinfect all contacted items while isolated. Seek medical testing and confirmation immediately.

The infant should be monitored closely for fever, rash, and other mpox symptoms. Use formula milk as an alternative feeding method during the suspension period.

When to resume breastfeeding

Breastfeeding can only be safely resumed after all mpox skin lesions have fully crusted over and fallen off, all symptoms (fever, fatigue, etc.) have completely resolved, and isolation is completed with medical evaluation.

record_id Q39_Qwen

question_id Q39

model Qwen

answer_text:

No, direct breastfeeding is not recommended for a mother with suspected Mpox. You should temporarily suspend direct breastfeeding until all lesions have crusted over, scabs have fallen off, and a new layer of intact skin has formed .

However, you should continue to pump and express your milk to maintain your milk supply for the future, although the expressed milk must be discarded.

Here are the specific safety guidelines and actions you should take:

Risks of Transmission

The primary risk of infection comes from direct skin-to-skin contact between the mother and the infant.

Contact Transmission: Even if you do not have lesions on your breasts, the close physical contact required for breastfeeding poses a significant risk of transmitting the virus to the baby .

Breast Lesions: If you have active lesions on your breast or nipple, the risk is even higher. In this specific case, you should pump and discard milk from the affected breast to prevent congestion or mastitis, while milk from a completely healthy breast could theoretically be fed to the baby if handled by someone else with strict hygiene .

Feeding Your Baby During This Period

Alternative Nutrition: You should feed your baby with infant formula or pasteurized donor human milk .

Discard Expressed Milk: Although it is currently unclear if the virus is present in breast milk, the CDC recommends discarding milk expressed by mothers with suspected or confirmed Mpox as a precaution .

Maintaining Milk Supply (Pumping)

To ensure you can breastfeed safely once you are no longer infectious, you need to keep making milk.

Pump Regularly: Pump frequently to empty the breasts and prevent engorgement or infection .

Hygiene: Wash your hands thoroughly before and after pumping.

Cleaning: A healthy caregiver should handle the cleaning of the pump parts and feeding the baby.

Protecting the Baby

Hand Hygiene: Wash your hands with soap and water or use alcohol-based hand sanitizer before touching the baby or any feeding equipment .

Mask Wearing: Wear a medical mask when near the baby to reduce the risk of respiratory transmission .

Cover Lesions: Ensure any lesions on your body are completely covered with clothing or bandages to prevent accidental contact with the infant .

Monitor the Infant: Watch closely for signs of Mpox in the baby, such as fever, swollen lymph nodes, or rash, and seek medical attention immediately if symptoms appear .

To provide more specific advice on your recovery and isolation, Do you currently have visible skin lesions? If so, are there any on your breasts or chest area?

Q40. Is mpox a sexually transmitted infection?

record_id Q40_ChatGPT

question_id Q40

model ChatGPT

answer_text:

I'll answer this from current public-health guidance and keep the distinction clear between "sexually transmissible" and "classified only as an STI."

Yes - mpox can be sexually transmitted, but it is not only a sexually transmitted infection.

Mpox spreads mainly through close contact with someone who has mpox, including skin-to-skin contact, kissing, oral/anal/vaginal sex, and other intimate contact. CDC says mpox can spread through "close, sexual, or intimate contact," and WHO notes that during the global outbreak beginning in 2022, it mostly spread through sexual contact.

The nuance: mpox is not like classic STIs that require sexual fluids or genital contact. It can also spread through non-sexual close contact, contaminated materials, contact with infected animals, or pregnancy/birth transmission. Researchers have also continued studying the role of semen and other body fluids.

So the best practical answer is: mpox is sexually transmissible and often handled by sexual-health/STI services, but it can spread outside sex too. If you think you were exposed or have a new unexplained rash, avoid close/sexual contact and contact a clinician or local public-health clinic about testing and vaccination.

record_id Q40_Gemini

question_id Q40

model Gemini

answer_text:

The short answer is that mpox is sexually transmissible, but it is not classified strictly or exclusively as a sexually transmitted infection (STI).

While sexual and intimate contact has been the primary driver of global outbreaks since 2022, the virus spreads through any form of close, sustained physical contact. This means it can easily be passed along in non-sexual settings as well.

How Mpox Transmits

The mpox virus travels primarily through direct contact with infectious rashes, scabs, or bodily fluids. This happens in a few main ways:

Intimate and Sexual Contact: The vast majority of cases in global outbreaks (specifically driven by Clade II of the virus) have spread within sexual networks. Transmission happens through skin-to-skin contact during oral, anal, or vaginal sex, as well as through kissing.

Non-Sexual Close Contact: Because the virus relies on proximity rather than the nature of the interaction, it can spread through routine household contact. This includes hugging, cuddling, or caring for an infected family member.

Contaminated Objects (Fomites): Touching items that have been in direct contact with an infected person's sores or fluids-such as bedding, towels, clothing, or sex toys-can transfer the virus.

Respiratory Droplets: Prolonged, face-to-face interactions (like talking closely or breathing near someone for an extended period) can spread the virus through respiratory particles, though this plays a much smaller role than direct skin contact.

Why the Distinction Matters

Public health agencies like the World Health Organization (WHO) and the CDC view mpox as an infection that is frequently acquired via sexual contact, but they avoid labeling it solely as an STI for practical reasons.

A Overlapping Diagnosis: Because mpox sores can look remarkably similar to classic STIs like herpes or syphilis, and because it frequently spreads through sexual networks, healthcare providers routinely screen suspected mpox patients for a full panel of STIs concurrently.

Classifying it exclusively as an STI could cause people to overlook non-sexual household risks. Conversely, ignoring its sexual component would hinder targeted prevention, education, and vaccination campaigns (such as the JYNNEOS vaccine) aimed at protecting communities currently at the highest risk.

record_id Q40_Copilot

question_id Q40

model Copilot

answer_text:

Short answer: Yes - mpox can be transmitted during sexual activity and has been spread predominantly through sexual contact in recent outbreaks, but it is not limited to sexual transmission and can spread through any close skin-to-skin or respiratory contact.

What "sexually transmitted" means for mpox

Mpox (formerly called monkeypox) is a viral infection that spreads mainly through close, sustained physical contact. That includes skin-to-skin contact during sex, kissing, and contact with mucosal lesions, and in some cases exposure to contaminated materials like bedding or sex toys. During the 2022-2023 global outbreak, transmission was observed almost exclusively in sexual and social networks, especially among men who have sex with men, which supports sexual encounters as a major route of spread.

Evidence about sexual fluids and infectiousness

Viral DNA and infectious virus have been detected in semen and other sexual fluids in some cases, but researchers are still clarifying whether semen is a primary vehicle of transmission or whether transmission mainly occurs from direct contact with lesions during sex.

Several expert groups argue that mpox meets common definitions of a sexually transmitted infection because it is frequently transmitted during sexual activity and can be passed via bodily fluids and mucosal/skin contact.

Practical implications and what to do

If you have symptoms (fever, new rash, painful anogenital or oral lesions) avoid sexual contact and close physical contact until evaluated. Seek testing and medical care; clinicians may also test for other sexually transmitted infections because co-infections are common. Vaccination is available in many places for people at higher risk and can be used for prevention or post-exposure protection.

Key takeaways

Mpox can be sexually transmissible and has been spread predominantly through sexual contact in recent outbreaks.

It is not exclusively an STI - nonsexual close contact, contaminated objects, and respiratory exposure can also transmit the virus.

If exposed or symptomatic, get evaluated, avoid sex, and follow public health guidance on testing, isolation, and vaccination.

A Position Statement on Mpox as a Sexually Transmitted Disease Clinical Infectious Diseases CDC

record_id Q40_DeepSeek

question_id Q40

model DeepSeek

answer_text:

Whether mpox is classified as a sexually transmitted infection (STI) depends on whether you are using a strict or a practical definition. The key is that mpox is not exclusively an STI, but it is very commonly transmitted that way, and for public health purposes, it's treated as one.

Here's a breakdown of the nuance.

The "Yes" vs. "No" Debate

The Official 'No' Definition (Strict View): Many health authorities state that mpox is not an STI. This is because it can also be spread through non-sexual close contact, such as touching lesions, sharing bedding or clothing, or through respiratory droplets-51--22. The virus isn't biologically "designed" exclusively for sexual transmission.

The Practical 'Yes' Approach (Epidemiological View): In actual outbreaks, sexual contact is a primary driver. Since 2022, the vast majority of cases in non-endemic countries have been linked to sexual networks, particularly among men who have sex with men (MSM)-1-47. Because of this, the CDC recommends that anyone evaluated for mpox also be screened for other STIs, which is a standard practice for STIs-

How Mpox Spreads: The Mechanisms

The confusion comes from the different ways mpox can be transmitted, as outlined by the WHO:

Sexual Contact: The primary route in recent global outbreaks. Any close, intimate contact during sex-skin-to-skin, kissing, oral, or penetrative sex-can spread the virus-43-. The virus is present in lesions, bodily fluids, and respiratory droplets, all of which are exchanged during sex.

Non-Sexual Close Contact: This can be through direct contact with the rash, scabs, or body fluids of an infected person

Indirect Contact: This happens when you touch contaminated objects like clothing, bedding, towels, or surfaces an infected person has used

Respiratory Droplets: While less common, prolonged face-to-face contact can spread the virus through large respiratory droplets-.

Household Transmission: This is often how children become infected, through close, non-sexual physical contact with an infected family member-.

Epidemiological Nuance: Clades and Transmission

The way mpox spreads can differ depending on which viral clade is involved.

Clade IIb: Drove the 2022 global outbreak, with spread almost entirely through sexual contact

Clade Ib: Fuels the ongoing outbreak in Central/Eastern Africa. Its primary spread is also through sexual networks, but it also transmits more easily within households, often affecting children more significantly

Clade Ia: The original endemic form, historically linked to contact with infected animals and non-sexual human-to-human transmission in Central/West Africa

Public Health Consensus: Treating Mpox as an STI

In practice, health experts agree that to control mpox, it must be treated as an STI.

Regardless of the strict definition, sexual transmission is a crucial pillar of the virus's spread-.

The populations most at risk-and therefore prioritized for vaccination-are individuals in high-risk sexual networks, such as MSM and sex workers

As expert Dr. Hunter Handsfield notes, "To not call it an STI is nearly equivalent to saying syphilis isn't an STI because exceptions to sexual transmission are common in some settings"

The Real Significance: Why This Matters for You

For prevention, the distinction is less about definition and more about action:

Risk is based on exposure, not identity. While some groups may be more affected, anyone who has skin-to-skin or intimate contact with an infected person can catch the virus

Safer sex practices, such as talking to partners about symptoms, avoiding contact with rashes or sick people, and considering vaccination if at risk, are highly effective ways to prevent mpox

If you test positive for mpox, health guidelines recommend also getting tested for other STIs like HIV, syphilis, and chlamydia due to the overlap in transmission networks-.

In short, while mpox is not strictly an STI, its primary mode of spread in the current global outbreak is through sexual networks. Therefore, using STI prevention strategies-like vaccination and safer sex practices-is the most effective way to protect yourself.

record_id Q40_Doubao

question_id Q40

model Doubao

answer_text:

Short answer: Mpox is not formally classified as a traditional exclusive STI, but sexual and intimate close contact is the dominant transmission route in the ongoing global outbreak, and it spreads very commonly through sexual activity.

Key details

Multiple transmission routes (not only sexual)

Mpox spreads primarily via direct physical contact with infectious skin lesions, rashes, scabs, or bodily fluids of an infected person. It can also spread through:

Prolonged face-to-face respiratory droplet exposure

Contact with contaminated bedding, clothing, or towels

Zoonotic exposure (infected animals, the original source of the virus)

Non-sexual close personal contact (e.g., household care for an infected person)

Strong link to sexual transmission in current outbreaks

Nearly all cases in the global 2022-present outbreak are linked to sexual/intimate skin-to-skin contact (oral, anal, vaginal sex, kissing, close bodily touching) with an infected person. Lesions frequently develop first on the genitals, anus, or perineum-making sexual networks the main driver of spread.

Critical distinction from classic STIs

Unlike chlamydia, gonorrhea, or syphilis (which spread almost exclusively via sexual contact), mpox can spread through non-sexual close contact too. However, in current epidemiology, it behaves like an STI for public health prevention purposes.

Important note

Condoms do not fully prevent mpox infection, as transmission occurs via skin-to-skin contact with lesions (not just bodily fluid exchange).

record_id Q40_Qwen

question_id Q40

model Qwen

answer_text:

Mpox (formerly known as monkeypox) is not technically classified as a Sexually Transmitted Infection (STI), but it is highly sexually transmissible.

While it spreads very efficiently during sexual contact, its primary mode of transmission is close physical contact rather than sexual fluids alone. Here is a breakdown of the distinction and the risks:

The Distinction: STI vs. Sexually Transmissible

Why it is not a typical STI: Classic STIs (like HIV or syphilis) rely on sexual fluids (semen, vaginal secretions) to spread. Mpox, however, spreads primarily through direct contact with skin lesions (rashes) or sores. It can also spread through contaminated bedding, towels, or prolonged face-to-face respiratory droplets .

Why it is sexually transmissible: Sexual activity involves the close skin-to-skin contact and friction that allows the virus to transfer easily. Recent outbreaks have shown that sexual contact is the predominant route of transmission in the community .

How Transmission Actually Happens

Contact with Sores: The highest risk comes from touching the rash or scabs of an infected person. These sores often appear on the genitals, anus, or thighs .

Intimate Contact: The virus can enter through microscopic tears in the skin or mucous membranes during oral, anal, or vaginal sex.

Body Fluids: While the virus has been found in semen and anal/sexual fluids, the concentration is generally lower than in skin lesions. However, transmission via fluids is still possible .

Prevention Strategies

To protect yourself, you should treat mpox similarly to other STIs but with extra caution regarding skin contact:

Avoid Contact with Rashes: Do not touch the skin or sores of a partner, even if the rash is not in the genital area.

Communication: Ask sexual partners about any recent rashes or symptoms before becoming intimate.

Vaccination: Vaccination is the most effective way to prevent infection, especially for high-risk groups.

Hygiene: Avoid sharing towels, unwashable sex toys, or bedding with someone who is infected.

In summary, while you can catch mpox through sex, you can also catch it from simply hugging or sleeping in the same bed as an infected person. Therefore, it is safer to view it as a disease of close physical contact rather than just a sexual one.

Q41. I am worried about mpox after one episode of unprotected sex, what should I do?

record_id Q41_ChatGPT

question_id Q41

model ChatGPT

answer_text:

I'm sorry you're dealing with that anxiety. A single episode of unprotected sex does not automatically mean you were exposed to mpox. Mpox risk is highest if your partner had a new/unexplained rash, sores, scabs, fever, swollen lymph nodes, rectal pain, or a known/suspected mpox diagnosis. Mpox spreads through close/intimate contact, especially skin-to-skin contact with rash or scabs; condoms can reduce some exposure but do not fully prevent mpox because lesions can be on uncovered skin.

What to do now:

1. If the sex was within the last 72 hours, treat HIV prevention as time-sensitive.

Contact urgent care, an ER, a sexual health clinic, or your doctor today to ask about HIV PEP. CDC says PEP must be started within 72 hours after a possible HIV exposure, and sooner is better.

2. If your partner has confirmed/suspected mpox or had a rash/sores, ask about the JYNNEOS mpox vaccine now.

Post-exposure vaccination is recommended as soon as possible, ideally within 4 days, and can still provide some protection if given 4-14 days after exposure.

3. Monitor for symptoms for 21 days.

Mpox symptoms usually start within 21 days. Watch for a new rash or sores, especially on/near the genitals, anus, mouth, hands, feet, chest, or face; also fever, chills, swollen lymph nodes, exhaustion, muscle aches, headache, sore throat, cough, or rectal pain/bleeding.

4. Get tested only if you develop a mpox-like rash or lesions.

Mpox testing is usually done by a clinician swabbing lesions; routine testing without a rash is generally not recommended. While waiting for results, avoid sex and close skin-to-skin contact.

5. Also handle the other unprotected-sex risks.

Ask for STI testing for gonorrhea, chlamydia, syphilis, HIV, and hepatitis B status. If pregnancy is possible, emergency contraception works best as soon as possible and is used within 5 days after unprotected sex.

Seek care urgently if you develop painful genital/anal sores, a spreading rash, fever with swollen lymph nodes, eye pain, severe rectal pain/bleeding, or if you are immunocompromised, pregnant, or have severe eczema.

record_id Q41_Gemini

question_id Q41

model Gemini

answer_text:

It is completely understandable to feel anxious right now, but taking proactive steps can give you both control over your health and peace of mind.

Here is a clear breakdown of what you should do next:

1. Check Your Timeline for the Mpox Vaccine (PEP)

If you suspect or know that your partner has mpox, you can get the JYNNEOS vaccine as Post-Exposure Prophylaxis (PEP). This helps your body fight off the virus or significantly reduce its impact.

Within 4 Days: Getting vaccinated within 4 days of the exposure gives you the best chance of preventing the disease entirely.

Days 4 to 14: Receiving the vaccine during this window can still help make the illness much milder if you do happen to contract it.

2. Monitor Yourself for 21 Days

The standard monitoring period for mpox is 21 days from the date of the encounter. Check yourself daily for:

Early Signs: Fever, chills, exhaustion, headache, muscle aches, or swollen lymph nodes (especially in the neck, armpits, or groin).

A Rash or Lesions: Sores can look like pimples or blisters and may be painful or itchy. In cases transmitted sexually, they often show up first in the genital, anal, or oral areas before appearing on other parts of the body.

Important: If you develop a rash or flu-like symptoms, isolate from others, keep the sores covered, and call a healthcare provider right away.

3. Consider Other Sexual Health Exposures

Since this was an episode of unprotected sex, it is highly recommended to look at your overall sexual health right now:

HIV nPEP: If the encounter happened within the last 72 hours, you can take emergency medication called Non-Occupational Post-Exposure Prophylaxis (nPEP) to prevent HIV. It is most effective the sooner you start it.

STI Screening: Plan to get tested for other common STIs (like chlamydia, gonorrhea, and syphilis). A clinician can advise you on the correct testing timeline, as different infections take varying amounts of time to show up on a lab test.

What to Do Right Now

Call a local sexual health clinic, an urgent care facility, or your county public health department as soon as possible. Let them know you had a potential exposure and want to evaluate your options for preventative vaccines or medications.

To help me give you the most relevant information for your situation, roughly how many hours or days ago did the encounter occur, and do you know if your partner has a confirmed or suspected case of mpox?

record_id Q41_Copilot

question_id Q41

model Copilot

answer_text:

If you had one episode of unprotected sex, your immediate steps are: avoid further sexual contact, watch for symptoms for 21 days, and contact a healthcare provider or local public health for testing and possible post-exposure vaccination.

What to do right now

Avoid sexual contact and close skin-to-skin contact with others until 21 days after the exposure or until a clinician rules out mpox. Do not share bedding, towels, or clothing that touched the genital or oral area.

Contact a healthcare provider or your local public health department as soon as possible to report the exposure and get advice about testing and post-exposure prophylaxis (PEP) vaccination. In Los Angeles, county public health can advise on testing and vaccination locations.

What to watch for and how long

Incubation period is usually 3-21 days. Watch for fever, new swollen lymph nodes, headache, muscle aches, and especially a new rash or lesions on the genitals, anus, mouth, hands, or elsewhere. If any symptoms appear, seek medical evaluation promptly.

If you develop a rash or lesions, isolate (stay home, avoid close contact) and call your clinician or local health department before visiting so they can arrange safe testing.

Testing and treatment

Testing is done by swabbing lesions; if you have no lesions yet, testing is usually not possible until symptoms appear. Your clinician or public health authority will advise.

Treatment for most people is supportive care. Antiviral therapy exists for severe cases or people at high risk; a clinician decides if it's needed.

Post-exposure vaccination (PEP)

PEP vaccination may be offered to people with higher-risk exposures; vaccination is most effective if given within 4 days of exposure to prevent disease and can be considered up to 14 days to reduce severity. Discuss eligibility with public health or your clinician right away.

When to seek urgent care

Go to urgent care or the emergency department if you have severe pain, difficulty breathing, signs of bacterial superinfection (increasing redness, swelling, pus), or high fever. Tell them you may have been exposed to mpox so staff can use appropriate precautions.

Final points

Tell recent sexual partners so they can monitor for symptoms and seek care.

A clinician or public health official is the best next step for testing, PEP decisions, and local resources.

LAC DPH Mpox Infection Control

Treatment and prevention of mpox (formerly monkeypox)

record_id Q41_DeepSeek

question_id Q41

model DeepSeek

answer_text:

It's completely understandable to feel worried after an episode of unprotected sex. I've put together the most important information on mpox risk, symptoms, and the specific steps you can take right now, including how to get tested and the option of post-exposure vaccination.

Understanding Your Risk of Mpox

Mpox is primarily spread through close physical contact, and sexual contact is a significant route of transmission. Here's what the data shows about the risk:

Global Risk: A large systematic review found that the overall secondary attack rate-meaning the chance of catching mpox from an infected person-is around 2.74%--1. However, this rate varies significantly based on the type of contact.

Sexual Transmission Risk: The risk is substantially higher in cases of sexual contact. The same study reported a secondary attack rate of 11.35% for sexual contact, compared to just 1.34% for non-sexual contact

Specific Sexual Practices: The risk is also not uniform for all sexual activities. One analysis suggests that for gay and bisexual men, being the receptive partner during unprotected anal sex is a leading risk factor, increasing the chance of infection five-fold-.

It's important to remember that while your concern is valid, the overall risk of transmission from a single exposure event remains relatively low.

Immediate Steps to Take: Prevention & Testing

Time is of the essence for some preventive measures, but even if you're past that window, you are not out of options.

1. Seek Post-Exposure Prophylaxis (PEP) Vaccination

Vaccination after exposure (PEP) can help prevent the disease or reduce its severity.

Key Timeframes: It is most effective when given within 4 days of first exposure--30. It can still be offered up to 14 days after exposure if you remain symptom-free, but its efficacy is highest within the first 4 days-

How to Access: You should contact a healthcare provider, sexual health clinic, or your local public health department immediately to ask about PEP for mpox. You can tell them: "I had a possible exposure to mpox through unprotected sex [X] days ago and I'm seeking post-exposure prophylaxis (PEP) vaccination."

2. Get Tested for Mpox

If you are still within the incubation period (5-21 days, with 6-13 days being most common) or begin to show symptoms, testing is crucial-

When to Test: Testing is recommended if you develop a rash that resembles mpox (see below) or if you've had a known close contact-.

How It Works: A healthcare professional will use a sterile swab to vigorously swab a lesion (rash) to collect a sample. This sample is then tested using PCR to detect the virus's DNA-.

Where to Get Tested: Call or visit a sexual health clinic, your primary care doctor, or a travel medicine clinic. Many public health laboratories and some commercial labs offer this testing-. There is no reliable home self-test for mpox

What to Watch For: Symptoms of Mpox

Mpox symptoms usually begin within 21 days of exposure, with most people showing signs within 6 to 13 days-

Common Early Symptoms (Prodrome): The illness often starts with flu-like symptoms lasting 1 to 5 days before the rash appears-, including:

Fever

Headache, muscle aches, and backache

Swollen lymph nodes (a key distinguishing feature from smallpox or chickenpox)

Chills and exhaustion-

The Mpox Rash: The rash usually appears 1 to 5 days after the fever-. It can appear on the genitals, anus, face, mouth, hands, or feet-. The rash evolves through stages:

Macules: Flat red spots.

Papules: Raised bumps.

Vesicles: Fluid-filled blisters.

Pustules: Blisters filled with pus, which eventually crust over and fall off

The rash can be painful or itchy-.

Ongoing Prevention & General Advice

Regardless of vaccination status, you can take these steps to protect yourself and others:

Avoid Close Contact: Steer clear of anyone who has a rash that looks like mpox or who is known to have the virus-.

Practice Safer Sex: The act of sex involves the close contact through which mpox spreads. The WHO advises practicing safer sex as a way to reduce risk-.

Maintain Good Hygiene: Wash your hands often with soap and water or use an alcohol-based hand sanitizer-.

Consider Pre-Exposure Vaccination (PrEP): If you are at ongoing risk (e.g., have a new STI diagnosis, have multiple sex partners), getting the two-dose JYNNEOS vaccine series can provide strong protection-48. CDC recommends it for people with certain risk factors

Staying Healthy & When to Call a Doctor

Self-Quarantine: If you develop any symptoms consistent with mpox, you should isolate at home in a separate room if possible, avoid physical contact with people and pets, and cover any lesions. You should remain isolated until all scabs have fallen off and a fresh layer of skin has formed-.

Consult a Healthcare Provider: It is very important to see a doctor if you notice a new or unexplained rash, have been in close contact with someone with mpox, or develop any of the early flu-like symptoms.

I hope this gives you a clear path forward and some peace of mind. Please reach out to a healthcare provider as soon as you can to discuss your situation. If you have any other questions, I'm here to help.

record_id Q41_Doubao

question_id Q41

model Doubao

answer_text:

It's understandable to feel worried after unprotected sexual exposure to mpox (monkeypox). Below is clear, actionable, evidence-based steps you should take right away, organized by priority:

1. Act immediately for Mpox Post-Exposure Prophylaxis (PEP Vaccine)

This is the most important step to lower your risk of infection:

The mpox vaccine is most effective if given within 4 days of unprotected exposure; it still offers benefit up to 14 days after exposure.

Contact your local sexual health clinic, STI clinic, public health department, or primary care provider right now to ask about mpox PEP vaccination. Don't delay.

2. Self-monitor for symptoms for 21 full days

The mpox incubation period is 5-21 days (most people develop symptoms in 7-14 days). Check yourself daily for:

Early flu-like symptoms: Fever, chills, headache, muscle aches, extreme fatigue

Swollen, tender lymph nodes (a key sign that differentiates mpox from other rashes)

A painless or painful rash: Starts as small bumps, turns to blisters/pustules, then scabs. Often first appears on the genitals, anus, mouth, face, hands or feet.

3. Avoid all intimate/close contact for 21 days

To prevent spreading mpox to others if you are infected:

No oral, vaginal, or anal sex; no kissing, hugging, or close skin-to-skin contact.

Do not share bedding, towels, razors, underwear, or eating utensils with others.

Keep personal items separate from household members.

4. Get tested immediately if any symptoms appear

If you develop fever, swollen lymph nodes, or a suspicious rash, go straight to a sexual health/STI clinic or healthcare provider.

Mpox testing uses a swab of rash lesions for confirmation.

Isolate yourself fully at home until you can be seen and tested. Never pop or scratch blisters/rash (this spreads the virus).

5. Get full STI screening

Unprotected sex also carries risks of other sexually transmitted infections: HIV, syphilis, chlamydia, gonorrhea, herpes. Ask your clinic for a complete STI panel at the same time you inquire about mpox PEP.

6. If you test positive for mpox

Most mpox cases are mild and resolve in 2-4 weeks with home care.

Isolate alone until all rash scabs fall off completely, new skin forms, and you have no fever/symptoms.

Use over-the-counter pain/fever relievers; avoid touching the rash.

Follow your healthcare provider's guidance for care.

Reassurance

Mpox is rarely severe for otherwise healthy people. Taking the above proactive steps (especially early vaccine PEP) drastically reduces your chance of getting infected or developing serious symptoms. Try to avoid excessive anxiety-focus on acting quickly with the steps above.

record_id Q41_Qwen

question_id Q41

model Qwen

answer_text:

It is understandable that you are worried, but try to stay calm. Mpox (monkeypox) is primarily spread through direct skin-to-skin contact with a rash or sores, not just through bodily fluids like semen.

Here are the specific steps you should take now:

Monitor for Symptoms (The 21-Day Rule)

The incubation period for mpox is typically 7 to 14 days, but it can take up to 21 days for symptoms to appear.

What to look for: Watch for fever, headache, muscle aches, backache, swollen lymph nodes, chills, and exhaustion. Most importantly, check for a rash or lesions on your genitals, anus, mouth, or buttocks .

Action: If you develop any of these symptoms, avoid close contact with others and contact a healthcare provider immediately.

Contact a Doctor Before Visiting

If symptoms do appear, do not walk directly into a clinic or hospital emergency room without calling ahead.

Why: You need to notify the medical staff of your potential exposure so they can prepare a private room or specific protective measures to prevent spreading the virus to others in the waiting room .

Consider Other STIs

Unprotected sex carries a risk for various sexually transmitted infections (STIs).

Action: Since mpox is just one possibility, it is highly recommended to visit a sexual health clinic for a full STI screening (including HIV, syphilis, and herpes) if you haven't done so recently.

Hygiene and Prevention

Hand Washing: Wash your hands regularly with soap and water, especially after using the bathroom or touching your face.

Avoid Contact: Until the 21-day risk period has passed, consider avoiding sexual contact or close physical contact with others to be safe .

To give you better advice, could you tell me when did this unprotected contact happen, and do you know if the other person has any visible rashes or sores?

Q42. Do men who have sex with men definitely get mpox?

record_id Q42_ChatGPT

question_id Q42

model ChatGPT

answer_text:

No. Men who have sex with men do not "definitely" get mpox. Mpox can affect anyone; risk depends on exposure, especially close skin-to-skin or intimate contact with someone who has mpox, not on sexual orientation itself. The 2022-present global outbreak has affected primarily, but not only, gay, bisexual, and other men who have sex with men because transmission has occurred through certain close-contact sexual networks.

Risk is higher if someone has close/intimate contact with a person who has mpox, has multiple or new sex partners in an area where mpox is circulating, has sex at commercial sex venues or large events, or is not fully vaccinated. CDC recommends the 2-dose JYNNEOS mpox vaccine for people with certain risk factors; the second dose is given 4 weeks after the first.

Condoms may reduce some risk, but they do not fully prevent mpox, because mpox can spread through skin contact, rashes/lesions outside condom-covered areas, kissing, cuddling, and contact with contaminated bedding or clothing.

Watch for a new or unexplained rash, sores/blisters, fever, chills, swollen lymph nodes, muscle aches, or exhaustion; symptoms usually start within 21 days after exposure. If you think you were exposed or have a rash, avoid close/intimate contact and contact a healthcare provider or sexual health clinic for testing and vaccine guidance.

record_id Q42_Gemini

question_id Q42

model Gemini

answer_text:

The Short Answer

No, men who have sex with men (MSM) do not definitely get mpox.

Contracting mpox is not an inevitability for anyone based on their sexual orientation or identity. Mpox is an infectious disease caused by a virus. Catching it requires direct, close exposure to the virus itself-not simply belonging to a specific demographic. The vast majority of MSM have never had mpox.

Why Is There an Association?

During the global outbreak that began in 2022, the virus spread primarily within interconnected social and sexual networks of gay, bisexual, and other men who have sex with men.

The Mechanism: The virus spreads exceptionally well through close, skin-to-skin contact, which naturally happens during intimate or sexual encounters.

The Network: Because the virus entered these specific, closely connected networks early on, it circulated more heavily within them.

Public health agencies emphasize that anyone who has close physical contact with an infected person can contract mpox, regardless of their gender or sexual orientation. It is a matter of exposure, not identity.

How Mpox Actually Spreads

Transmission is about physical proximity and contact, not who you are. It primarily spreads through:

Direct skin-to-skin contact with mpox rashes, scabs, or body fluids.

Intimate physical contact, such as kissing, cuddling, massaging, or sex.

Contaminated items, like sharing bedding, towels, or clothing used by someone with an active infection (though this is less common).

Prevention and Protection

Because it is a viral illness, there are highly effective tools available to prevent transmission and protect your health:

Vaccination: The two-dose JYNNEOS vaccine is safe and highly effective at preventing infection or significantly reducing the severity of symptoms if an exposure does occur.

Awareness & Communication: Being aware of unexplained rashes or lesions on yourself or your partners, and pausing close physical contact if someone feels unwell, drastically reduces risk.

Low-level transmission still occurs occasionally, but proactive health measures and vaccination offer excellent, robust protection.

record_id Q42_Copilot

question_id Q42

model Copilot

answer_text:

Short answer: No - men who have sex with men (MSM) do not automatically or definitely get mpox. Mpox can infect anyone through close skin-to-skin or intimate contact, but recent outbreaks have disproportionately affected gay, bisexual, and other men who have sex with men.

How mpox spreads

Mpox is transmitted mainly by close, direct contact with an infected person's rash, scabs, or body fluids, and by prolonged face-to-face contact that can spread respiratory secretions. Sexual or intimate contact is a common route because it involves prolonged skin-to-skin exposure. However, the virus is not limited to any one sexual orientation or gender.

Why MSM were disproportionately affected

During the 2022-2024 outbreaks, most diagnosed cases occurred among MSM because of transmission networks and exposure patterns, not because of an inherent biological susceptibility. Public-health analyses show that outbreak size and recurrence risk depend on levels of immunity and behavior in affected networks.

Asymptomatic and undiagnosed infections

Some infections can be asymptomatic or mild, and undiagnosed cases likely contributed to spread; recent studies found asymptomatic infections among MSM and suggested many infections go undetected. This means someone can carry and potentially transmit mpox without obvious symptoms.

What this means for you

You are not destined to get mpox simply because you are MSM. Risk depends on exposure to an infectious person and on protective measures.

Vaccination (JYNNEOS) reduces risk; two doses give better protection than one. Public-health agencies recommend vaccination for people at higher risk.

Avoid close contact with anyone who has a new unexplained rash, sores, or flu-like symptoms; do not share bedding, towels, or clothing with someone who is infected.

When to seek care and testing

See a healthcare provider if you have a new rash, sores (including on genitals or anus), fever, swollen lymph nodes, or unexplained symptoms. Testing can confirm mpox and guide treatment and isolation.

Tell recent partners and follow public-health guidance about isolation until cleared by a clinician.

Final note

If you're worried about exposure, vaccination, or symptoms, contact a local sexual-health clinic or your healthcare provider for testing and advice. Public-health measures and vaccination are the best ways to reduce personal and community risk.

Potential for Recurrent Mpox Outbreaks Among Gay, Bisexual, and Other Men Who Have Sex with Men - United States, 2023 MMWR

Understanding Mpox And Your Sexual Health American Sexual Health Association

Asymptomatic transmission plays a prominent role in mpox infection patterns

record_id Q42_DeepSeek

question_id Q42

model DeepSeek

answer_text:

That's an important question, and the short answer is no. Men who have sex with men (MSM) do not definitely get mpox.

Here's a more detailed breakdown:

The Key Facts

Anyone can get mpox. Mpox is not a "gay disease." It spreads through close, physical (including skin-to-skin) contact with an infected person or contaminated materials (like bedding or towels). This means anyone-regardless of sexual orientation or gender identity-can catch it if exposed.

Why the misconception exists: During the 2022 global outbreak, the vast majority of cases in non-endemic countries were initially reported among MSM. This was not because the virus inherently targets MSM, but because the virus was introduced into and spread rapidly through certain interconnected social and sexual networks. Public health messaging focused on these groups to target resources and awareness effectively.

The virus doesn't care about identity. From a biological standpoint, mpox transmits through specific behaviors (prolonged close contact, contact with lesions, respiratory droplets during extended face-to-face interaction). Your sexual orientation doesn't change how a virus functions.

So, who is at risk?

High risk: Anyone who has direct contact with an infected person's rash, scabs, or body fluids, regardless of that person's sexual orientation.

Elevated risk (during outbreaks): People with multiple or anonymous sexual partners, especially if mpox is circulating in their community. This can include MSM, but also heterosexual people in similar networks.

Bottom Line for an MSM individual:

If you have no contact with an infected person or their contaminated belongings, your risk is effectively zero.

If you have close physical or sexual contact with someone who has mpox, your risk is high (again, this is true for anyone).

Vaccination (the JYNNEOS vaccine) is highly effective at preventing infection or reducing severity.

Conclusion:

Definitely not. It is a virus that spreads through specific forms of contact, not through sexual orientation. Stigmatizing mpox as an "MSM disease" is scientifically inaccurate and harmful. The best protection is vaccination (if you're at risk), avoiding close contact with people who have symptoms, and practicing good hygiene.

If you're an MSM individual concerned about mpox, talk to a healthcare provider about the vaccine, especially if you have multiple or new sexual partners. But don't assume you will inevitably get it just because of your sexual identity.

record_id Q42_Doubao

question_id Q42

model Doubao

answer_text:

No, men who have sex with men (MSM) definitely do not inevitably contract mpox.

Key facts to clarify the misunderstanding:

Mpox is transmitted by virus exposure, not by sexual orientation itself

Mpox spreads mainly through close physical contact, direct contact with mpox lesions, respiratory droplets, or contaminated personal items. Simply being an MSM does not mean you carry or will catch the virus. Infection only happens if you have contact with an infected person or contaminated sources.

Higher risk != certain infection

In global mpox outbreaks, MSM groups had higher infection rates mainly due to frequent close intimate contact and clustered social activities, which increased the chance of virus exposure. This is a difference in exposure risk, not an inevitable result.

Infection is preventable

Avoiding close contact with confirmed cases, using proper protection in intimate contact, maintaining good hygiene, and getting mpox vaccination can effectively prevent infection. Many MSM have never been infected with mpox at all.

Anyone can get mpox

People of any sexual orientation or gender can contract mpox if they have close contact with the virus. It is not a disease exclusive to MSM.

record_id Q42_Qwen

question_id Q42

model Qwen

answer_text:

No, men who have sex with men (MSM) do not "definitely" get mpox. Being part of this group does not guarantee infection.

However, recent outbreaks have shown that MSM are a high-risk population. During the recent global outbreaks, studies indicated that approximately 98% of confirmed mpox cases were gay or bisexual men .

Here is the distinction between "high risk" and "definite infection," along with how to stay safe:

Why is the risk higher for this group?

The high number of cases is not due to biology, but rather mode of transmission and behavioral factors:

Close Skin-to-Skin Contact: Mpox spreads primarily through prolonged, close physical contact, including sexual contact.

Anogenital Transmission: Research suggests that sexual activity, particularly unprotected sex with multiple or anonymous partners, is a major route of transmission .

Network Effects: In communities where sexual networks are dense, the virus can spread rapidly if an outbreak is introduced.

It is not inevitable (Not "Definite")

Mpox is a preventable disease. Many men who have sex with men never contract the virus because they take precautions. Infection depends on whether you are exposed to the virus, not just who you have sex with.

Actionable Steps for Prevention

To avoid infection, the following measures are highly effective:

Vaccination: Getting vaccinated against mpox (and potentially HIV/hepatitis) is the most effective way to prevent severe disease and infection.

Safe Sex Practices: Using condoms and dental dams can reduce the risk, although mpox can spread through skin contact that condoms do not cover.

Avoid High-Risk Situations: Limiting the number of sexual partners and avoiding sexual contact with individuals who have rashes or symptoms of mpox significantly lowers risk.

Monitor Symptoms: If symptoms appear (fever, swollen lymph nodes, or a rash that looks like pimples or blisters), seek medical attention immediately and avoid close contact with others.

Conclusion: While the statistics show a high prevalence in the MSM community, getting mpox is not a certainty. It is a matter of exposure. With the right precautions, anyone can protect themselves.

Q43. Should I tell my sexual partner if I suspect I have mpox?

record_id Q43_ChatGPT

question_id Q43

model ChatGPT

answer_text:

Yes. Tell them now, especially if you have a new/unexplained rash, sores, fever, swollen lymph nodes, or flu-like symptoms after close contact. CDC guidance says that if you or a partner has mpox or thinks they may have mpox, you should avoid sex of any kind, kissing, and skin-to-skin contact while sick, and get checked by a healthcare provider as soon as possible.

A simple message could be:

Hey, I wanted to let you know I'm having symptoms that might be mpox. I'm going to get checked/tested, but until I know more we should avoid sex, kissing, and close skin-to-skin contact. You may want to contact a clinic or health department too, especially if you notice any rash, sores, fever, or swollen lymph nodes.

You should also pause sex and close physical contact, avoid sharing towels/bedding/sex toys, and contact a clinician or sexual health clinic for testing. Mpox is considered contagious from symptom onset until all scabs have fallen off and fresh skin has formed.

Your partner may be eligible for post-exposure vaccination if exposure was recent; CDC says it should be given as soon as possible, ideally within 4 days, and may still help 4-14 days after exposure.

Seek care urgently if you have lesions near your eyes, severe rectal/genital pain, trouble urinating, worsening symptoms, or if you are pregnant, immunocompromised, or have severe eczema.

record_id Q43_Gemini

question_id Q43

model Gemini

answer_text:

What You Should Do Immediately

Yes, you should tell your sexual partner right away, and you should pause all physical intimacy.

Here is the step-by-step guidance recommended by public health officials if you suspect you have mpox:

1. Pause All Intimate Contact

Stop physical contact: This includes vaginal, anal, and oral sex, as well as kissing, hugging, or close skin-to-skin cuddling.

Do not share personal items: Avoid sharing towels, bedding, clothing, fetish gear, or sex toys. The virus can easily spread through materials that have touched an active rash or bodily fluids.

2. Talk to Your Partner

Be direct about your symptoms: Let them know if you have a new or unexplained rash, fluid-filled sores, or flu-like symptoms (like fever, chills, body aches, or swollen lymph nodes).

Why timing matters: If you do have mpox, your partner may have already been exposed. If they seek medical care quickly, getting the mpox vaccine within 4 to 14 days of exposure can prevent the infection entirely or significantly reduce how severe it gets.

3. Seek Medical Evaluation

Call ahead to a clinic: Contact a primary care doctor, local community health clinic, or urgent care center to get tested. Be sure to tell them before you walk in that you suspect mpox so they can guide you to the right room safely.

Isolate while you wait: Stay home and isolate from others (including pets) until a healthcare provider evaluates you or your test results come back negative. If you must be around others in your household, keep all rashes fully covered and wear a well-fitting mask.

If you need help notifying them: If you are uncomfortable reaching out to a partner yourself, or if you don't have their direct contact info, your local public health department can assist. Public health professionals can contact your partners anonymously to notify them of a potential exposure and connect them to vaccines or care, without ever revealing your name or details.

record_id Q43_Copilot

question_id Q43

model Copilot

answer_text:

Yes - you should tell sexual partner(s) if you suspect you have mpox. Avoid sex and close contact until you've been evaluated, and encourage partners to seek testing, vaccination, or medical advice as recommended by public health.

Quick comparison of options

Attribute Tell partner Don't tell partner

Risk to partner Reduces risk; allows them to seek care/vaccine. Leaves partner unknowingly exposed.

Public health Enables contact tracing and PEP. Prevents timely public health response.

Trust/relationship Supports honesty and safer choices. May damage trust if discovered later.

Immediate actions available Testing, isolation, vaccination, monitoring. No timely interventions.

Legal/ethical Aligned with public health guidance. May have ethical/legal implications in some places.

Key considerations and decision points

Do you have symptoms (rash, lesions, fever, swollen lymph nodes)?

Have you had recent sexual or close intimate contact with others since symptoms began?

Can you exchange contact info so partners can be notified and followed up? These questions guide whether and how urgently partners should be notified.

What to tell them

Be direct and factual: say you have symptoms that could be mpox or that you're awaiting test results.

Ask them to avoid sex and close contact with others until they get medical advice.

Tell them where/when you were exposed or had sex if relevant, and offer to share clinic/testing info. Public health guidance recommends exchanging contact information with new partners so follow-up is possible.

What to do now (practical steps)

Avoid all sexual activity, kissing, and close skin-to-skin contact until evaluated.

See a healthcare provider promptly and ask specifically for mpox testing if you have a rash or lesion; testing is a swab of a lesion.

Notify local public health if you test positive - they can offer contact tracing and post-exposure prophylaxis (PEP) for eligible contacts.

How to talk about it (phrasing tips)

Use short, factual statements: "I have a new rash and I'm getting tested for mpox. Please avoid sex and see a provider."

Offer resources: local sexual health clinic, mpox vaccine sites, or your clinic contact.

Risks, protections, and follow-up

Condoms may reduce but do not eliminate mpox risk because lesions can be on other body parts.

Vaccination (two doses) and timely PEP can reduce risk for exposed partners; public health can advise eligibility.

Mpox Q&A - California Department of Public Health

Mpox Healthcare Provider Resources - Department of Public Health

English MPV & SAFER SEX Fact Sheet 052223 FINAL

record_id Q43_DeepSeek

question_id Q43

model DeepSeek

answer_text:

Yes, you should absolutely tell your sexual partner if you suspect you have mpox.

Here's why this is critical:

Mpox is highly contagious through close, intimate contact. This includes skin-to-skin contact with the rash or lesions, respiratory droplets during prolonged face-to-face contact, and contact with contaminated clothing, bedding, or towels. Sexual activity involves all of these routes.

Your partner needs to know to protect their own health. While mpox is often not severe, it can be very painful, can lead to scarring, and in rare cases, causes serious complications (especially for immunocompromised individuals, pregnant people, and young children). Your partner may also live with or be in close contact with someone at higher risk.

They need to monitor for symptoms. Early symptoms (fever, swollen lymph nodes, muscle aches, headache, fatigue) can appear before the rash. Knowing they were exposed allows them to isolate quickly if symptoms develop, get tested, and seek treatment (like tecovirimat, or TPOXX) which is most effective early on.

It prevents further spread. If your partner doesn't know they were exposed, they might unknowingly pass it to others during the incubation period. Telling them breaks the chain of transmission.

What you should do right now:

Stop all close physical contact with your partner and anyone else (including household members, using separate towels/bedding, and ideally sleeping in a different room if possible).

Tell your partner honestly: "I've developed some symptoms that could be mpox, so I'm getting tested. I wanted you to know right away so you can take precautions and watch for symptoms."

Get tested immediately. Contact your healthcare provider, a sexual health clinic, or your local public health department. They can confirm whether it's mpox or something else (like herpes, syphilis, or chickenpox).

Follow medical advice on isolation. If confirmed, you'll need to isolate until all lesions have crusted over, fallen off, and a fresh layer of healthy skin has formed (typically 2-4 weeks).

What about embarrassment or fear of their reaction? It's completely understandable to feel anxious. But remember: telling them is the responsible, caring, and ethical choice. Hiding a suspected infection puts them at risk without their consent. Most partners will appreciate your honesty, especially because early warning allows them to take protective steps like getting vaccinated (JYNNEOS vaccine can prevent or lessen illness if given soon after exposure).

Are you sure it's mpox? Common symptoms include a new rash (flat spots that turn into fluid-filled blisters, then pustules, then scabs), often starting in the genital or anal area, plus fever, swollen lymph nodes, and headache. But other infections can look similar. Only testing can confirm.

Don't wait. Tell your partner today, avoid contact until you have a diagnosis, and get tested.

record_id Q43_Doubao

question_id Q43

model Doubao

answer_text:

Yes, you absolutely should tell your sexual partner right away.

This is critical for their health, your shared safety, and public health, and it's an ethical, responsible choice. Here's why and what to do next:

Key reasons to disclose

Mpox spreads easily via intimate/sexual contact

Mpox transmits mainly through direct skin-to-skin contact, sexual activity, and close personal contact with someone who has the virus. Your partner needs to know to watch for symptoms and avoid unknowingly spreading it to others.

Their right to informed health choices

They deserve the chance to protect their own body, pause sexual/close contact, get tested, and seek medical care early if needed.

Stops the spread chain

Early disclosure prevents passing mpox to more people and lets both of you isolate and recover safely.

What to do immediately after telling them

Pause all close, intimate, and sexual contact until you've been medically evaluated, tested, and any symptoms (rashes, blisters, sores, fever, swollen lymph nodes) have fully healed.

Both of you see a healthcare provider right away for mpox testing, screening, and guidance on isolation, monitoring, and care.

Monitor for symptoms for up to 3 weeks after potential exposure.

It can feel awkward to have this conversation, but it is kind, responsible, and necessary to protect your partner and yourself. Healthcare providers can also support you with how to talk about this and next steps.

record_id Q43_Qwen

question_id Q43

model Qwen

answer_text:

If you suspect you have mpox (monkeypox), you should immediately inform recent sexual partners and avoid all intimate contact until you receive a medical evaluation and diagnosis. This is critical because mpox spreads primarily through close skin-to-skin contact, including sexual activity, and early disclosure allows partners to monitor symptoms, seek testing, and prevent further transmission.

1. Key Reasons to Disclose

Why disclosure is medically and ethically necessary:

Transmission risk is high during symptomatic periods: Mpox spreads through direct contact with infectious rashes, scabs, or bodily fluids during sexual activity. Even asymptomatic individuals may transmit the virus briefly before symptoms appear, though this is less common.

Sexual contact is a major transmission route: In global outbreaks since 2022, over 95% of cases were linked to sexual contact, particularly among people with multiple or new partners.

Early intervention prevents complications: Partners who know their exposure risk can seek testing and care faster, reducing the chance of severe outcomes (e.g., secondary infections or prolonged illness).

Legal and ethical obligations:

Many regions require disclosure of infectious diseases that pose transmission risks through intimacy. While mpox-specific laws vary, failing to warn partners could lead to civil liability if they become infected due to negligence.

Ethically, partners have a right to (informed consent) about health risks affecting their safety. Withholding this information undermines trust and autonomy in relationships.

2. Practical Steps to Take

If you suspect mpox:

Stop all sexual contact immediately and avoid close physical interaction (e.g., hugging, kissing) until cleared by a healthcare provider.

Contact a healthcare provider promptly for testing. Mention your symptoms (e.g., fever, swollen lymph nodes, or unusual rashes on genitals, anus, or mouth) and potential exposure history.

Notify recent sexual partners discreetly:

Share only necessary details (e.g., "I have symptoms consistent with mpox; get tested if you develop a rash or fever").

Avoid naming others or sharing stigmatizing language to protect privacy.

Follow isolation guidelines: Stay isolated until all scabs fall off and new skin forms (typically 2-4 weeks).

If diagnosed with mpox:

Continue notifying partners from the 21 days before symptoms began, as this is the maximum incubation period.

Use condoms for 12 weeks post-recovery if sexually active, as the virus may persist in bodily fluids.

3. Addressing Common Concerns

"What if I'm embarrassed or fear stigma?"

Mpox is not exclusive to any group-anyone can contract it through close contact. Healthcare providers are trained to handle disclosures confidentially, and many clinics offer anonymous partner notification services.

"Could I face legal consequences for transmitting it?"

Deliberately hiding a confirmed diagnosis and exposing others may lead to civil lawsuits for medical costs or harm. However, simply suspecting mpox (without a diagnosis) carries no legal penalty for disclosure.

Mpox is usually mild and self-limiting, but timely communication protects both your health and others'. If unsure how to approach the conversation, ask a healthcare provider for guidance-they can help frame the discussion sensitively. Always prioritize honesty and care over silence

Q44. Is it correct for people online to link mpox with certain groups of people?

record_id Q44_ChatGPT

question_id Q44

model ChatGPT

answer_text:

It depends what "link" means.

It is not correct to say mpox "belongs to" or is caused by any identity group, such as gay men, LGBTQ+ people, African people, or any race/nationality. Mpox spreads through close or intimate contact, contact with contaminated materials, and in some regions contact with infected animals; CDC says it can spread to anyone through close personal contact and does not spread through casual contact like being in an office, classroom, pool, or airplane.

It can be correct for public-health messaging to note that some outbreaks have disproportionately affected particular networks. WHO says the 2022 global outbreak affected primarily, "but not only," gay, bisexual, and other men who have sex with men, and spread through sexual networks. That kind of information can help target vaccines, testing, and outreach without blaming anyone.

The problem is when people turn an epidemiological pattern into a stereotype-e.g., "mpox is a gay disease" or using it to shame LGBTQ+ people or Africans. WHO says stigma and discrimination around mpox are never acceptable and can undermine public-health efforts; UNAIDS similarly warned that stigmatizing language can reinforce homophobic or racist stereotypes and drive people away from care.

A good rule: talk about behaviors, exposures, and outbreak context, not blame or identity. "People with close skin-to-skin or sexual exposure to someone with mpox may be at risk" is accurate. "Those people are the problem" is not.

record_id Q44_Gemini

question_id Q44

model Gemini

answer_text:

When evaluating how mpox is discussed online, it is essential to separate epidemiological data-which tracks how and where a virus is currently spreading-from social stigmatization, which mischaracterizes the virus as being inherently tied to a specific identity.

While public health data does show that certain demographics have been disproportionately affected by specific outbreaks, online narratives that frame mpox as an exclusive or identity-based disease are scientifically incorrect and public health failures.

1. The Epidemiological Reality (Transmission Networks)

Public health agencies, including the World Health Organization (WHO) and the CDC, acknowledge that certain transmission chains are concentrated within specific communities:

Clade IIb Outbreaks: The global outbreak that began in 2022 (and continues to circulate at lower levels) has predominantly affected interconnected social and sexual networks of gay, bisexual, and other men who have sex with men (MSM).

Clade I (Ia and Ib) Outbreaks: Conversely, outbreaks in Central and Eastern Africa have historically demonstrated widespread transmission through heterosexual contact, non-sexual household contact, and fomites (contaminated linens/clothing), frequently affecting women and children.

The critical distinction is that this distribution is driven by network mechanics and close physical contact, not by any biological vulnerability unique to a specific group of people.

2. Why "Identity-Linking" is Scientifically Incorrect

Viruses are biologically neutral. They do not have the capacity to choose a host based on sexual orientation, race, or nationality.

Mode of Transmission vs. Identity: Mpox spreads through close, personal, often skin-to-skin contact. Anyone-regardless of their identity-who has sustained, intimate contact with an infectious rash, body fluids, or respiratory droplets can contract the virus.

Network Effects: If a virus is introduced into a tightly knit social or sexual network where close physical contact is common, it will spread rapidly within that network before jumping to others. This is a matter of proximity and exposure opportunity, not demographic destiny.

3. The Public Health Hazard of Online Stigma

When online commentary shifts from epidemiological tracking to moralizing or generalizing a disease to specific groups, it causes severe real-world harm to outbreak control:

Driving Disease Underground: Stigma deters individuals who might have been exposed from seeking medical attention, getting tested, or pursuing vaccination. Fear of judgment or discrimination leads to hidden cases.

Flawed Risk Perception: If the general public believes mpox only affects "certain groups," individuals outside those groups may ignore symptoms or assume they are not at risk, which can lead to delayed diagnoses and unintended wider community spread.

Public Health Consensus: Acknowledging which populations are currently at the highest statistical risk is necessary for allocating vaccines and resources effectively. However, weaponizing that data to label a disease as belonging to a specific community is scientifically inaccurate and actively undermines efforts to contain it.

Are you looking at this issue from a public health messaging and communication perspective, or are you tracking the specific epidemiological differences between the various mpox clades?

record_id Q44_Copilot

question_id Q44

model Copilot

answer_text:

No - linking mpox to specific groups is inaccurate and harmful. Mpox spread is driven by exposure and behaviour, not identity, and blaming groups increases stigma, reduces testing and vaccination, and worsens outbreak control.

Why linking mpox to certain groups is wrong

Mpox can infect anyone who is exposed to the virus; early outbreaks affected some networks more, but that is not the same as the virus being a disease of a particular identity. Public-health reviews show the 2022-2025 mpox outbreaks disproportionately affected some men who have sex with men, but researchers stress that stigma around identity discouraged testing and care and impeded control efforts.

How stigma harms public health and people

Discourages care seeking - people avoid testing, treatment, and vaccination for fear of being labelled.

Amplifies misinformation - online conspiracies and anti-vaccine narratives have targeted LGBTQ+ people, increasing harassment and false beliefs.

Creates barriers in healthcare - anticipated discrimination leads to delayed or clandestine care, worsening outcomes.

What accurate, non-stigmatizing communication looks like

Frame risk by behaviour and exposure, not identity. Explain who is at higher risk because of specific exposures (e.g., close skin-to-skin contact, certain sexual networks) rather than naming groups.

Use clear prevention steps: vaccination where recommended, avoid close contact with symptomatic people, seek testing for suspicious lesions.

Highlight that anyone can be infected and that public-health measures protect whole communities.

What you can do when you see group-blaming online

Correct misinformation with facts: point to behaviour-based transmission and official guidance rather than repeating identity-based claims.

Call out stigma calmly: explain how blaming harms people and public health; offer resources for accurate information.

Report abusive or false content to platform moderators and share trusted public-health links.

Quick practical takeaways

Fact: Mpox spreads by close contact; anyone exposed can get it. .

Action: Encourage testing and vaccination where available; avoid language that ties disease to identity. .

If you're worried: seek care from a trusted clinician or community health service; many LGBTQ+ health organizations provide nonjudgmental mpox resources. .

record_id Q44_DeepSeek

question_id Q44

model DeepSeek

answer_text:

No, it is not correct or fair to link mpox with specific groups of people. Mpox can affect anyone, regardless of their background, identity, or community. Associating the virus with particular groups not only spreads misinformation but also fuels stigma and discrimination, which can discourage people from seeking testing, treatment, or vaccination. Public health experts emphasize that diseases should be discussed based on scientific evidence, not used to target or blame certain populations. Responsible communication about mpox focuses on transmission routes, symptoms, and prevention measures that apply to everyone.

record_id Q44_Doubao

question_id Q44

model Doubao

answer_text:

It is incorrect, unscientific and inappropriate to arbitrarily link mpox (monkeypox) with specific groups of people online. Here are the key reasons:

Medical fact: Mpox does not target any specific group

Mpox is a zoonotic viral infectious disease. It spreads mainly through close physical contact, exposure to infected animals or contaminated items, and respiratory droplets. People of all ages, ethnicities, lifestyles and social groups are equally susceptible to infection if they have exposure risks. It is never an exclusive disease of any particular crowd.

It causes unfair stigma and discrimination

Labeling certain groups with mpox will trigger unnecessary social prejudice, exclusion and discrimination against innocent groups. This kind of stereotyped labeling is unfair to individuals in those groups and harms their legitimate rights and dignity.

Hinders public health prevention and control

Stigma makes some people who have potential infection risks reluctant to seek medical treatment actively or hide their symptoms for fear of being labeled. This will delay diagnosis, isolation and treatment, and further increase the risk of virus transmission in society.

Clear official stance

Global health authorities have clearly opposed associating mpox with any specific population. They emphasize focusing on transmission behaviors and exposure scenarios rather than attaching inappropriate labels to groups.

In short, we should view mpox from a scientific perspective, abide by public health guidelines, reject group labeling and stigma, and avoid spreading biased and misleading remarks online.

record_id Q44_Qwen

question_id Q44

model Qwen

answer_text:

No, it is not correct to link mpox (formerly known as monkeypox) to any specific group of people. Doing so is both scientifically inaccurate and socially harmful.

Here is a breakdown of why this happens and why it is misleading:

1. Viruses do not discriminate

Mpox is an infectious disease caused by a virus. It does not target people based on their sexual orientation, gender identity, race, or ethnicity. Anyone, regardless of who they are, can contract mpox if they have close, often skin-to-skin contact with someone who has the virus, or through contact with contaminated materials (like bedding or clothing).

2. The danger of stigma

When a disease is unfairly linked to a specific group (such as the LGBTQ+ community, which was heavily stigmatized during the 2022 global outbreak), it creates several serious problems:

Discourages testing: People outside that group may falsely believe they are "safe" and ignore symptoms, leading to further spread.

Delays treatment: People within the targeted group may avoid seeking medical help due to fear of judgment or discrimination.

Fuels discrimination: It leads to bullying, harassment, and social isolation of innocent people.

3. How it actually spreads

Mpox spreads primarily through:

Direct skin-to-skin contact with mpox rashes, scabs, or body fluids.

Prolonged face-to-face contact (respiratory droplets).

Touching items (such as clothing or linens) that previously touched the infectious rash or body fluids.

In summary: While certain outbreaks may statistically affect specific networks of people due to patterns of social or sexual contact, the virus itself is not exclusive to any group. Public health experts emphasize that we should focus on behaviors and transmission methods (like close contact) rather than labeling or blaming specific identities.

Need me to cover the key prevention measures for mpox?

Q45. What should I pay attention to before traveling to an area where mpox is spreading?

record_id Q45_ChatGPT

question_id Q45

model ChatGPT

answer_text:

Before traveling to an area where mpox is spreading, focus on risk activities, vaccination timing, and what to do if symptoms appear.

Before you go: Check the CDC Travel Health Notice for your destination, because recommendations vary by country and clade. CDC specifically says travelers should consider travel health/medical evacuation insurance, discuss prevention with a healthcare provider, and get vaccinated before travel if eligible.

Vaccination: Mpox vaccination is not automatically recommended for every traveler, but CDC recommends it for people with certain risk factors, including travelers to countries with clade I outbreaks who anticipate sex with a new partner, sex at commercial sex venues, sex in exchange for money/goods/drugs, or sex associated with large events such as raves, parties, or festivals. JYNNEOS is a 2-dose vaccine; CDC says the second dose is given 4 weeks after the first, and best protection takes about 2 more weeks after dose two, so plan about 6 weeks ahead when possible.

Avoid higher-risk contact: Mpox spreads mainly through close or intimate contact with someone who has mpox, contact with contaminated items, or direct contact with infected animals. CDC notes it does not spread through casual contact like you would have in public spaces, classrooms, offices, pools, or routine travel settings.

During travel: Avoid skin-to-skin contact with anyone who has a rash or sores; do not kiss, hug, cuddle, or have sex with someone who may have mpox; do not share towels, bedding, clothing, cups, dishes, or utensils with someone who is sick. In areas where mpox circulates in animals, avoid rodents, primates, dead animals, bushmeat, and products made from wild animals.

Know the symptoms: Watch for a new or unexplained rash, including on the hands, feet, chest, face, mouth, genitals, or anus. Fever, chills, swollen lymph nodes, headache, muscle aches, back pain, sore throat, cough, congestion, and exhaustion can also occur. Symptoms usually appear within 21 days after exposure.

Take extra care if you are at higher risk for severe illness: Pregnant people, infants under 1 year, people with eczema or active skin conditions, and people who are immunocompromised should be especially cautious and should discuss travel plans with a clinician.

If symptoms appear during or after travel: Seek medical care promptly, avoid close contact with others, and tell the clinician where you traveled in the 21 days before symptoms began. CDC says people with mpox should isolate until symptoms are gone and the rash has fully healed, meaning scabs have fallen off and fresh skin has formed.

record_id Q45_Gemini

question_id Q45

model Gemini

answer_text:

Traveling to an area with active mpox transmission requires careful planning, a clear understanding of local transmission dynamics, and strict adherence to prevention protocols. Because of the distinct behavior of the two main viral subtypes-Clade I (currently causing outbreaks in parts of Central and Eastern Africa and generally associated with more severe outcomes) and Clade II (responsible for the ongoing global outbreak)-your preparation should be tailored to your specific destination and activities.

Here is what you need to pay attention to before and during your travel:

1. Pre-Travel Medical Preparation

Evaluate Vaccination Eligibility: The two-dose JYNNEOS vaccine is highly effective but is not routinely recommended for all travelers. It is primarily indicated if you are a healthcare worker deploying to an outbreak area or if you anticipate high-risk exposure (such as skin-to-skin or sexual contact with new partners in the affected area).

Factor in the Immunity Timeline: If vaccination is required, you must plan well in advance. The protocol requires two doses spaced 4 weeks apart, and peak immunity is achieved 2 weeks after the second dose. Ideally, start the process at least 6 weeks before departure.

Pack a Medical Kit: Ensure you have ample hand sanitizer (minimum 60% alcohol), disinfectant wipes for high-touch surfaces, disposable gloves, and a high-filtration mask (like a KN95 or N95) in case you must navigate crowded environments or assist someone who is unwell.

2. Transmission Prevention & Behavior Modification

Mpox relies heavily on close, personal contact. Adjusting your daily logistics minimizes risk:

Avoid Direct Skin-to-Skin Contact: The primary driver of transmission is direct contact with mpox rashes, scabs, or bodily fluids. Minimize skin-to-skin contact in crowded social venues (such as clubs, raves, or festival environments) where skin exposure is high.

Mitigate Fomite Transmission: The virus can survive on materials. Avoid handling textiles-such as bedding, towels, or clothing-or sharing utensils and cups with anyone who has suspected or confirmed lesions.

Prevent Zoonotic Spillover: If traveling to areas in Central or West Africa where the virus is endemic in wildlife, strictly avoid contact with wild animals, particularly rodents and primates. Do not handle, cook, or consume wild game (bushmeat).

Maintain Frequent Hand Hygiene: Wash hands regularly with soap and water or use an alcohol-based rub, especially before eating, touching your face, or after utilizing public transit.

3. Destination Logistics & Local Border Policies

Screening and Health Passes: Many countries have implemented strict entry requirements to curb Clade I entry. Check your destination's Ministry of Health or local embassy updates before flying; you may be required to complete digital health declaration forms or undergo temperature screenings at ports of entry.

Secure Medical Evacuation Insurance: Ensure your travel insurance covers clinical care, medical isolation, and potential medical evacuation. If you test positive or exhibit symptoms abroad, local health regulations will likely restrict you from boarding commercial flights until all lesions have completely crusted over and healed.

4. Monitoring Symptoms and Post-Exposure Protocols

Understand the Incubation Window: The incubation period ranges from 5 to 21 days. If you experience a potential exposure or return from a high-transmission zone, monitor your health closely for a full 3 weeks.

Recognize Early Signs: Watch for the prodromal phase-fever, chills, headache, severe fatigue, and lymphadenopathy (swollen lymph nodes, which helps distinguish it from chickenpox or smallpox)-before the distinctive rash appears.

Do Not Mask Symptoms: If you suspect exposure, avoid taking antipyretics like acetaminophen or ibuprofen unnecessarily, as they can mask the initial febrile phase and delay self-isolation.

Action Plan for Illness: If symptoms develop while traveling or immediately upon return, isolate yourself from others, do not use public or commercial transport, and contact a local healthcare facility before arriving so they can implement appropriate infection control measures.

record_id Q45_Copilot

question_id Q45

model Copilot

answer_text:

If you're traveling to an area with mpox spread, prioritize vaccination if eligible, avoid close skin-to-skin and sexual contact with people who have rashes, and plan for medical care and insurance before you go.

Key checklist before travel

Item Why it matters What to do

Vaccination Reduces risk of infection and severe disease. Get the 2-dose JYNNEOS series if eligible; start at least 6 weeks before travel when possible.

Exposure risks Mpox spreads mainly by close skin contact, sexual contact, and contaminated items. Avoid intimate/skin contact with people who have rashes; don't share bedding, towels, or utensils.

Health plan and evacuation Some areas may have limited treatment or isolation facilities. Buy travel health and medical evacuation insurance that covers infectious disease care.

Know symptoms and testing Early recognition speeds care and reduces onward spread. Watch for fever, swollen lymph nodes, painful rash; seek testing if symptoms appear within 21 days of exposure.

Local guidance and stigma Local outbreaks and affected groups vary; stigma can hinder care. Check local public health guidance and respect privacy when seeking care.

What to pay attention to before you leave

Vaccination timing and eligibility. CDC recommends vaccination for people who plan travel to countries with mpox outbreaks and who anticipate sexual or other close contact with new partners; the 2-dose schedule gives best protection when completed before travel.

How mpox spreads. Transmission is mainly through close skin-to-skin contact (including sexual contact), face-to-face respiratory exposure during prolonged contact, and contact with contaminated materials like bedding or clothing. Avoid raves, parties, or situations with minimal clothing and frequent skin contact.

Symptoms and incubation. Typical symptoms include fever, headache, muscle aches, swollen lymph nodes, and a painful rash or mucosal lesions that can last 2-4 weeks; incubation is usually up to 21 days. Seek testing and isolate if symptoms develop.

Prevention behaviors. Avoid kissing, hugging, cuddling, or sex with anyone who has a rash; do not share eating utensils or bedding; wash hands frequently; use gloves if handling potentially contaminated materials. If caring for someone ill, use PPE (gloves, gown, eye protection).

Plan for care and reporting. Know where to get local testing, treatment, and public health reporting contacts; consider travel health insurance that covers evacuation. Some jurisdictions recommend notifying public health for suspected clade I cases.

Risks, tradeoffs, and practical tips

Risk groups. People with multiple sexual partners and those in sexual networks with known cases have higher risk; vaccination programs often prioritize these groups.

Tradeoffs. Avoiding crowded intimate venues reduces exposure but may limit social plans; vaccination reduces risk but may not be available everywhere.

If you develop symptoms abroad. Isolate, seek local medical care, avoid travel until cleared, and notify local health authorities.

LAC/CDC Health Update Mpox Clade I & II -Prevention in the US and Before Travel

record_id Q45_DeepSeek

question_id Q45

model DeepSeek

answer_text:

Planning a trip to a region with mpox might feel concerning, but taking the right steps before you go can make your travels much safer. The key is to prepare ahead of time, protect yourself during your trip, and know what to do if you get sick.

Here's a practical guide to help you navigate it all.

What to Know Before You Go

Check Travel Advisories: Stay up-to-date on health advisories from the CDC or WHO for your destination. Countries in Central and Eastern Africa are currently experiencing Clade I mpox outbreaks-21-.

Consult a Healthcare Provider: Plan ahead and see a doctor or visit a travel clinic 4-6 weeks before your departure. They can provide a personalized risk assessment and discuss vaccination-.

Get Vaccinated (If Eligible): The 2-dose JYNNEOS vaccine is a key tool for prevention-1. It's particularly recommended for travelers who anticipate:

Sex with a new partner, especially in commercial venues or in exchange for money, goods, or drugs

Direct skin-to-skin contact, such as at a rave, party, or festival

Timing is Key: Ideally, you should get your first dose at least 6 weeks before your trip, and the second dose 4 weeks after that. You'll be best protected about 2 weeks after the second dose

Secure Travel Insurance: Consider purchasing travel health and medical evacuation insurance. It's a good idea to confirm that your policy covers any potential mpox-related medical care

Know the Two Clades: The illness can be mild or severe depending on which type, or "clade," of the virus. The table below outlines the key differences:

Feature Clade I (Central African) Clade II (West African)

Severity More severe illness, with a higher documented mortality rate-. A "high consequence infectious disease"-. Generally a mild, self-limiting illness-.

Mortality Rate Up to 10% in some documented outbreaks-. Less than 1% (the strain behind the 2022 global outbreak)-.

Primary Region Circulates in Central and Eastern Africa-. Primarily found in West Africa-.

Transmission Human-to-human contact has increased, but also zoonotic (from animals to humans)-6. Clade IIb has shown sustained human-to-human transmission worldwide

How to Protect Yourself During Your Trip

Avoid Direct Contact: Mpox spreads through direct skin-to-skin contact with an infected person's rash, lesions, or body fluids-2. Avoid hugging, kissing, cuddling, and sex with anyone showing symptoms

Steer Clear of Contaminated Objects: The virus can linger on surfaces. Do not touch or share bedding, towels, clothing, or eating utensils of a person with mpox

Practice Good Hand Hygiene: Wash your hands often with soap and water or use an alcohol-based hand sanitizer (with at least 60% alcohol), especially after using the bathroom or being in public areas

Avoid Certain Animals: In areas where mpox is endemic, avoid contact with wild or stray animals, especially rodents, non-human primates (like monkeys and apes), and sick or dead animals-4-13. Also, avoid eating or preparing meat from wild animals, often called "bushmeat"

Maintain Physical Distance: While not airborne, the virus can be transmitted through prolonged, face-to-face contact. Try to avoid being within 1 meter (approx. 3 feet) of someone who may be ill

What if You Get Sick?

Monitor Your Health: Mpox symptoms usually appear 3 to 21 days after exposure-. Be on the lookout for:

Fever, chills, swollen lymph nodes, headache, muscle aches, and fatigue

A rash of blisters, pimples, or sores that can appear on the face, hands, feet, genitals, or inside the mouth

Seek Immediate Medical Care: If you develop a rash with or without a fever, seek medical attention right away. Before going to a clinic or hospital, call ahead, inform them of your symptoms and travel history, and get advice on where to go

Post-Exposure Prophylaxis (PEP): If you have a known high-risk exposure (e.g., skin-to-skin contact), getting vaccinated within 4 days can help prevent the disease. It can still be offered up to 14 days after exposure to reduce the severity of illness-.

Isolate if You're Sick: If you are diagnosed with mpox, isolate yourself from others (and from pets) until all lesions have crusted over, the scabs have fallen off, and a fresh layer of skin has formed

Delay Your Return Travel: If you get sick while traveling, isolate yourself and do not use public transportation until you've been cleared by a healthcare professional or public health official to prevent spreading the virus further-.

record_id Q45_Doubao

question_id Q45

model Doubao

answer_text:

Precautions before traveling to an mpox-affected area

1. Check the epidemic situation in advance

Learn about the latest mpox epidemic updates, risk levels and official health reminders of the destination. Avoid going to high-risk crowded closed places such as crowded entertainment venues, bath centers and large gathering events with dense crowds.

2. Self-health assessment before departure

Postpone your trip immediately if you have fever, rash, swollen lymph nodes, skin ulcers or other discomforts similar to mpox symptoms. Learn the typical symptoms of mpox (fever, fatigue, followed by rash, blisters, pustules and lymphadenopathy) for self-identification.

3. Strengthen personal hygiene

Carry alcohol-based hand sanitizer, disinfectant wipes and disposable masks.

Keep good hand hygiene: do not touch your mouth, nose and eyes with unwashed hands.

Never share personal items such as towels, razors, tableware, clothes and bedding with others.

4. Avoid high-risk exposure

Avoid close physical contact, intimate contact with strangers or people with unexplained rashes/skin lesions.

Do not touch, feed or come into contact with wild animals (rodents, primates, etc., the main hosts of mpox), and do not eat wild game.

Avoid contact with contaminated bedding, clothing and secretions of suspected patients.

5. Accommodation and environmental protection

Choose regular and hygienic accommodation. Prioritize well-ventilated rooms; use disposable toiletries when possible, and keep the living space ventilated regularly.

6. Post-travel health monitoring

Monitor your health for 21 days after returning from the trip. Once fever, rash, blisters or swollen lymph nodes appear, isolate yourself at home immediately, seek medical treatment promptly, and take the initiative to inform doctors of your travel history and contact history to avoid close contact with others.

7. Follow local regulations and vaccine consultation

Abide by local public health and epidemic prevention rules. If you are in a high-risk group, you can consult a medical institution in advance about mpox vaccination recommendations for pre-travel protection.

record_id Q45_Qwen

question_id Q45

model Qwen

answer_text:

Before traveling to an area where mpox is spreading, the most critical step is to minimize close physical contact and prepare a hygiene kit.

Mpox primarily spreads through direct contact with skin lesions, bodily fluids, or contaminated objects.

Here are the specific precautions you should take:

Avoid High-Risk Interactions

No Touching: Strictly avoid touching the skin or sores of anyone who is sick or has suspicious rashes.

Social Distancing: Avoid crowded places like nightclubs, concerts, or religious gatherings where close contact is common.

Safe Intimacy: Be aware that condoms alone do not fully prevent the spread of mpox, as the virus can be transmitted via skin-to-skin contact in areas not covered by the condom. Avoid sexual contact with individuals who have symptoms or unknown health status.

Protect Yourself from Animals and Food

Wildlife: Do not touch dead or alive animals, particularly rodents (like rats and squirrels) and primates.

Food Safety: Avoid eating "bushmeat" (wild animal meat). Ensure all food, especially meat products, is thoroughly cooked before consumption.

Pack Essential Hygiene Items

Hand Sanitizer: Carry alcohol-based hand sanitizer or soap to clean your hands frequently, especially after touching public surfaces.

Protective Gear: Pack N95 masks (to wear in crowded indoor spaces) and disposable gloves (for handling potentially contaminated items like laundry).

Wound Care: If you have any open cuts or wounds on your skin, cover them completely with waterproof bandages to prevent the virus from entering through broken skin.

Consider Vaccination

If possible, consult a travel medicine specialist about receiving the mpox vaccine beforehand. This is highly recommended for travelers who may have high-risk exposures.

Entry Protocols

Health Declaration: Upon entering or leaving countries, you must declare if you have had contact with mpox patients or if you have symptoms like fever, rash, or swollen lymph nodes.

To provide more specific advice, which country or region are you planning to visit? (Specific areas like the Democratic Republic of Congo currently have different risk levels compared to other regions).

Q46. If I develop a rash during a trip and suspect it might be mpox, can I still take a flight?

record_id Q46_ChatGPT

question_id Q46

model ChatGPT

answer_text:

Generally, you should not take a flight if you develop a rash and reasonably suspect mpox, especially before you've been evaluated. WHO says that if you develop symptoms, you should refrain from travelling, isolate, and seek medical care immediately.

CDC guidance is similar: people with mpox should isolate until symptoms are gone and the rash has healed, meaning all scabs have fallen off and a fresh layer of skin has formed. CDC adds that it is especially important not to travel if you have fever, mouth sores, sore throat, nasal congestion, or cough. CDC's "if you are sick" guidance also advises staying home/isolation until the rash has healed and a new layer of skin has formed.

What to do now:

Do not board yet. Call a healthcare provider, travel clinic, urgent care, or local public health line for advice and possible testing.

Contact the airline to rebook; say you have a potentially contagious rash and are seeking medical guidance.

Isolate from others as much as possible. Cover the rash, wear a well-fitting mask around people, wash/sanitize hands, and avoid sharing towels, bedding, clothing, cups, or utensils.

Seek urgent care if you have eye involvement, severe pain, trouble swallowing, confusion, shortness of breath, dehydration, pregnancy, a weakened immune system, or a rash spreading rapidly.

One nuance: CDC research found aircraft transmission risk appears low and routine flight contact tracing is often not warranted, but CDC still recommends that people with mpox isolate and delay travel until no longer infectious. So the practical answer is: delay the flight unless a healthcare/public-health professional clears you or gives specific instructions.

record_id Q46_Gemini

question_id Q46

model Gemini

answer_text:

Developing a sudden rash while traveling is incredibly stressful, but no, you should not board a flight if you suspect you have mpox.

According to public health agencies like the CDC and the World Health Organization (WHO), individuals with a suspected or confirmed case of mpox must not travel and should avoid public transportation entirely.

Why You Cannot Fly

Contagiousness: Mpox spreads through close skin-to-skin contact, respiratory droplets during prolonged face-to-face interactions, and contact with contaminated materials (like blankets or airplane seats). A person is contagious from the very start of their symptoms until the rash has completely healed, meaning all scabs have fallen off and a fresh layer of skin has formed.

Airline Policies: Commercial airlines have strict rules prohibiting passengers with contagious conditions from flying. If flight crew or gate agents notice a visible, unexplained rash, they have the authority to deny boarding.

Border Screenings: Many international borders utilize health declarations or visual screenings. Attempting to travel with a suspected highly contagious illness could result in being flagged by border authorities and forced into a local government quarantine facility.

What to Do Right Now

Isolate in Place: Stay in your hotel room or current accommodation. Avoid close physical contact with others, and do not share bedding, towels, cups, or utensils. Keep the rash covered with clothing or bandages if you must be around anyone.

Call a Doctor Before Going In: Contact a local clinic, hospital, or public health authority. Call them before you arrive and explicitly tell them you suspect mpox. This allows the staff to prepare an isolated area for you so you do not accidentally expose patients in the waiting room.

Contact Your Airline and Travel Insurance: Once you have a medical assessment, get in touch with your airline to postpone your flight. If you have travel insurance (or bought the ticket with a premium credit card), check your policy-medical emergencies that legally prevent you from flying are typically covered for trip delays or extended lodging.

Taking care of your health locally is the safest, most responsible move for both your own recovery and the safety of your fellow travelers.

record_id Q46_Copilot

question_id Q46

model Copilot

answer_text:

If you develop a rash and suspect mpox, do not board a flight if you can avoid it - public health authorities advise delaying travel and getting evaluated; if travel is unavoidable, cover lesions, wear a well-fitting mask, and notify the airline and a clinician.

Immediate steps to take

Stop and seek medical evaluation right away - a clinician can examine the rash, arrange testing, and advise on infectiousness.

Delay travel if mpox is suspected or confirmed; public health guidance explicitly recommends postponing trips while symptomatic or infectious.

If you must travel for an urgent reason, cover all lesions with clothing or dressings and wear a well-fitting mask to reduce risk of spread; inform the airline and local health authorities so they can advise on next steps.

Why authorities advise against flying when symptomatic

Mpox spreads mainly through direct contact with lesions, scabs, or bodily fluids, and less commonly via prolonged respiratory exposure; close contact on a plane could transmit infection.

Investigations of past flights found very low documented transmission on aircraft, but public health agencies still recommend isolation while infectious to prevent any risk and to allow treatment and contact tracing.

Practical checklist before you consider flying

Get tested and follow clinician/public health advice; do not rely on self-diagnosis.

If test is positive, follow isolation guidance until lesions have healed and scabs fallen off (this is when infectiousness is generally considered to have ended).

Contact your airline to learn their policy; some carriers may refuse boarding for contagious illnesses or require medical clearance.

Consider travel insurance and medical evacuation coverage if you're abroad and may need care or extended stay.

Risks, limitations, and special considerations

Risk to others: even if in-flight transmission is uncommon, close prolonged contact (e.g., shared seats, caregiving) increases risk.

Local rules vary: some regions treat clade I mpox as higher priority for public health action; if you've traveled to or from areas with clade I circulation, authorities may require faster reporting and stricter measures.

If you're immunocompromised or traveling with high-risk people, avoid travel until cleared by a clinician.

Quick decision guide

Can you postpone the flight? - If yes, postpone and get evaluated.

Is the trip urgent and unavoidable? - Get rapid clinical advice, cover lesions, wear a mask, notify the airline, and follow public health instructions.

Mpox: Advice for travellers - Travel.gc.ca

Mpox Quicksheet - California Department of Public Health

record_id Q46_DeepSeek

question_id Q46

model DeepSeek

answer_text:

No, you should not fly if you suspect you have mpox. According to the World Health Organization (WHO), individuals showing signs and symptoms compatible with mpox should refrain from non-essential travel and close contact with others-4-18. This guidance is in place to prioritize passenger safety and prevent the potential spread of the virus in the confined environment of an aircraft.

What You Should Do Now

If you suspect you have mpox, the most important step is to seek medical care immediately. A healthcare professional can assess your condition and, if needed, confirm a diagnosis.

Delay Travel: You should postpone your travel until you have been cleared by a healthcare professional who confirms you are no longer infectious-. The CDC recommends people with mpox remain isolated until the rash has fully healed, meaning all scabs have fallen off and a fresh layer of skin has formed-.

Report Your Symptoms: If your symptoms develop while on the flight, you should inform a flight attendant before landing or a border services officer upon arrival-.

Why Air Travel is Discouraged

While mpox does not spread easily from person to person, the risk on an aircraft is the primary reason health authorities advise against flying when symptomatic.

Means of Transmission: Mpox primarily spreads through prolonged, close, skin-to-skin contact with someone who has an active rash-. It can also be transmitted through respiratory droplets during prolonged face-to-face interactions and via contact with contaminated items like clothing or bedding-. An airplane cabin is a potential environment for such contact.

In-Flight Risk is Low: It's important to know that the specific risk of mpox transmission on a flight is considered very low. Research from the US Centers for Disease Control and Prevention (CDC) has found no documented cases of in-flight transmission to nearby passengers, which led them to discontinue routine aircraft contact tracing-10-30. However, the general public health guidance to avoid travel when sick remains in effect.

Potential Consequences of Flying

Attempting to fly while knowingly infected with a contagious disease can have serious repercussions.

Denied Boarding and Legal Risks: Airlines have the right to refuse to board passengers with contagious illnesses that could pose a risk to the health and safety of others-. Furthermore, flying while knowingly infected could potentially expose you to legal penalties in some jurisdictions, as intentionally exposing others to a contagious disease may be considered a misdemeanor or a more serious offense-.

No General Travel Bans: It's also helpful to note that, at this time, no international organizations are recommending blanket travel restrictions, entry screening, or vaccination requirements for international travel related to mpox-.

record_id Q46_Doubao

question_id Q46

model Doubao

answer_text:

No, you should NOT take a flight if you develop a rash during a trip and suspect it might be mpox. All major health authorities (CDC, WHO, E, IATA) strongly recommend that people with suspected or confirmed mpox avoid travel until fully recoveredEuropean Centre for Disease Prevention and Control.

What to do instead

Seek medical care immediately

Call ahead to a local healthcare provider or public health authority to inform them of your symptoms and travel history

Avoid contact with others while seeking care

Get tested to confirm or rule out mpox

Isolate yourself

Stay in your accommodation or a designated isolation location

Avoid close contact with others, including sharing personal items

Cover your rash completely with clothing or dressings and wear a well-fitting mask if you must be around others

Delay travel

Postpone your flight until you are no longer infectious

Infectious period ends when all scabs have fallen off and a fresh layer of skin has formed (usually 2-4 weeks)European Centre for Disease Prevention and Control

It's especially important to avoid travel if you also have fever, mouth sores, or respiratory symptoms

If travel is absolutely necessary (medical emergency)

If you must travel for urgent medical care:

Cover all rash lesions completely with clothing or dressings

Wear a well-fitting mask at all times around others

Avoid touching shared surfaces without gloves

Minimize contact with other passengers and crew

Notify airline staff about your condition before boarding

If you develop symptoms during a flight

Inform the flight attendant immediately before landing

Cover your rash and wear a mask

Cooperate with health authorities upon arrival for assessment and possible isolation

Key reasons for this advice

Mpox spreads through close contact with infected individuals or contaminated objects

While the risk of transmission on planes is considered low (especially with proper precautions), it's not zero

Early isolation helps prevent further spread of the diseaseEuropean Centre for Disease Prevention and Control

Airlines may refuse boarding to passengers with visible mpox lesions

Remember

The most responsible course of action is to delay travel until you've been evaluated by a healthcare provider and cleared of mpox. If you test negative, you can travel once your symptoms improve; if positive, wait until fully recovered.

record_id Q46_Qwen

question_id Q46

model Qwen

answer_text:

If you develop a rash during travel and suspect it might be mpox (monkeypox), you should NOT take a flight. International health guidelines explicitly advise against traveling while symptomatic or under suspicion of mpox infection, as it poses transmission risks and may violate public health regulations. Below is a detailed explanation based on current recommendations.

1. Immediate Travel Restrictions

Suspected mpox cases must avoid all travel, including flights, until cleared by health authorities. The World Health Organization (WHO) states that individuals with suspected, probable, or confirmed mpox should refrain from any travel until symptoms resolve and lesions fully heal (all scabs, with new skin formed) .

Airlines and border authorities may deny boarding to passengers with unexplained rashes or symptoms consistent with mpox, especially if linked to outbreak regions. In some countries (e.g., Thailand), travelers from high-risk areas must register in health passport systems, and symptomatic individuals face entry denial .

2. Risks of Flying with Suspected Mpox

Key concerns include:

Transmission risk: Mpox spreads through direct contact with lesions, bodily fluids, or contaminated surfaces. In-flight close proximity to others (e.g., shared armrests, lavatories) increases the chance of spreading the virus .

Legal consequences: Many countries require travelers to declare symptoms like fever or rash at customs. Failure to report may lead to quarantine enforcement, fines, or criminal liability under laws like China's Frontier Health and Quarantine Law .

Personal health risks: Delaying medical care could worsen complications (e.g., secondary infections, severe pain), particularly for immunocompromised individuals.

3. Recommended Actions Instead of Flying

If you suspect mpox during travel:

Seek medical evaluation immediately:

Contact local healthcare providers and disclose your symptoms and travel history. Testing via PCR of lesion samples is required for confirmation .

Avoid public transport; use private vehicles or emergency services if available.

Isolate until cleared:

Remain in isolation until a healthcare professional confirms you are no longer infectious (typically 2-4 weeks after symptom onset, once all lesions heal) .

Cover lesions with clothing/dressings and wear a well-fitted medical mask to reduce transmission risk if brief contact with others is unavoidable.

Notify relevant authorities:

Report suspected cases to local public health agencies. Some countries (e.g., the U.S.) require healthcare providers to notify within 24 hours .

If already at an airport, inform staff immediately-do not attempt to board.

4. Exceptions and Special Cases

Medical evacuation: In life-threatening emergencies (e.g., severe complications), specialized air ambulances with infection-control protocols may be arranged, but routine flights are prohibited .

Post-recovery travel: If diagnosed, you may resume travel only after healthcare providers confirm non-infectious status (full lesion healing). Some countries require medical clearance certificates for re-entry .

Summary

Do not board a flight if you suspect mpox. Prioritize medical evaluation and isolation to protect public health and comply with international regulations. Most cases are mild and self-limiting with proper care, but traveling while infectious risks legal penalties and community spread. For real-time guidance, consult local health authorities or resources like the WHO's Mpox Travel Advice.

Q47. Could I get mpox from hotel bedsheets?

record_id Q47_ChatGPT

question_id Q47

model ChatGPT

answer_text:

Yes, it's possible, but from hotel bedsheets it's generally low risk-especially if the sheets were changed and laundered normally.

Mpox mainly spreads through close skin-to-skin or intimate contact with someone who has mpox. It can also spread by touching contaminated fabrics or objects, including bedding or towels, that were used by someone with mpox and not disinfected/laundered afterward. CDC specifically lists bedding, towels, and clothing as possible contaminated materials. WHO also says contaminated clothing or linen can transmit mpox.

For a hotel stay, the realistic risk would be higher only if the linens were not changed or not properly washed after a person with active mpox lesions used them. Evidence suggests routine laundering greatly reduces risk from fabrics.

What to do:

If the sheets looked clean and you have no symptoms, there's usually no need to panic.

Watch for symptoms for up to 21 days: rash/blisters, fever, swollen lymph nodes, sore throat, headache, muscle aches, or low energy.

If you know you had contact with linens used by someone confirmed/suspected to have mpox, contact a clinician or local health department. Post-exposure vaccination works best as soon as possible, ideally within 4 days, and may still help within 14 days.

A normal hotel bed with laundered sheets is not considered a common way people get mpox.

record_id Q47_Gemini

question_id Q47

model Gemini

answer_text:

The Short Answer

While it is theoretically possible to contract mpox from contaminated bedding, your actual risk of getting it from a standard hotel bedsheet is extremely low.

The mpox virus can survive on porous surfaces like fabric, but standard commercial laundering practices easily destroy it, meaning properly cleaned hospitality linens do not pose a practical threat.

How Transmission via Linens Works

Mpox is primarily spread through direct, skin-to-skin contact with infectious rashes, scabs, or body fluids. However, it can also spread via fomites (contaminated objects or materials):

Viral Stability: The mpox virus is relatively resilient and can survive for days or even weeks on porous materials like sheets, blankets, and towels if they remain unwashed.

Direct Exposure: In documented cases where bedding transmission has occurred, it almost exclusively happens within households or intimate settings where individuals directly share unwashed sheets or clothing recently used by someone with an active infection.

Why the Risk in Hotels is Minimal

The hospitality industry follows routine cleaning and sanitation protocols that effectively eliminate the risk of surface transmission:

Commercial Laundering: Standard hotel laundry procedures utilize high-temperature water, industrial detergents, and often bleaching agents. Because mpox is an enveloped virus, it is highly susceptible to these standard washing chemicals.

High-Heat Drying: The thermal processing used in industrial dryers completely inactivates any remaining viral particles.

Fresh Turnovers: Because sheets are completely replaced and laundered between guests, the chain of transmission is broken before you ever enter the room.

Standard Precautions for Travel

Inspect the Room: Ensure the room looks clean and properly turned over. Visibly soiled or heavily wrinkled sheets may indicate poor housekeeping oversight.

Request Fresh Linens: If you ever doubt whether the bedding was changed, you can ask the front desk for a fresh set of sheets directly from the laundry facility.

Practice Hand Hygiene: Washing your hands regularly with soap and water or using an alcohol-based hand sanitizer remains the most effective baseline defense against traveling pathogens.

record_id Q47_Copilot

question_id Q47

model Copilot

answer_text:

Short answer: Yes, mpox can be transmitted from contaminated bedding or clothing, but the risk from briefly using hotel sheets is generally low unless those items were recently in contact with an infectious person's rash, scabs, or body fluids. Take simple precautions (avoid direct contact with visible lesions, wash hands, launder or request fresh linens) to reduce an already small risk.

Transmission risk overview

How mpox spreads: The virus spreads mainly through close, skin-to-skin contact with a person who has symptoms (rash, scabs), contact with respiratory secretions during prolonged face-to-face contact, and by touching contaminated objects (fomites) such as bedding, towels, or clothing that have contacted lesions or body fluids.

Survival on fabrics: Mpox virus can persist on porous surfaces (blankets, sheets, clothing) longer than on hard surfaces; studies have detected live virus on bedding and clothing days to weeks after contamination under some conditions. This means contaminated linens can be infectious if not cleaned.

Practical risk for hotel stays

Low for casual exposure: Briefly lying on hotel sheets that were recently changed and laundered by the hotel poses low risk. Hotels typically wash linens between guests; properly laundered fabrics are not expected to transmit the virus.

Higher if linens were recently used by an infectious person: If bedding was recently in contact with an infected person's open lesions, scabs, or bodily fluids and not cleaned, transmission is possible.

What to do if you're concerned

Inspect bedding for visible stains, scabs, or damage; ask for fresh linens if anything looks suspicious.

Avoid direct contact with any visible rash or scabs on linens. Wash hands after handling hotel linens.

Request freshly laundered sheets or a room change if you suspect contamination; reputable hotels will replace and launder linens.

If you handled potentially contaminated items, launder your clothes in hot water and detergent or use hotel laundry services. The CDC and public health agencies recommend cleaning and disinfection of contaminated materials.

When to seek care

If you develop fever, swollen lymph nodes, or a new rash/sores after a possible exposure, contact a healthcare provider or local public health department for testing and guidance. Early evaluation is important for diagnosis and treatment options.

Bottom line

Contaminated bedding can transmit mpox, but the everyday risk from properly laundered hotel sheets is low. Use common-sense precautions-ask for clean linens, avoid touching visible lesions, and wash hands-to keep the risk minimal.

Mpox - Preventing Mpox

record_id Q47_DeepSeek

question_id Q47

model DeepSeek

answer_text:

It's understandable to wonder about this, but the chance of you getting mpox from hotel bedsheets is extremely low.

Mpox generally doesn't spread easily from surfaces alone. The vast majority of cases happen through prolonged, direct, skin-to-skin contact with an infected person's rash or bodily fluids-5-22. While the virus can survive on fabrics like bedsheets, this usually only poses a real risk in a specific scenario-like a household where an infected person is actively using them day after day-

Here is a more detailed breakdown:

Understanding the Real Risk

The Virus on Surfaces: The mpox virus is known to survive on porous materials, like bedding and clothing, potentially for days or even weeks under ideal (cool, dark, dry) conditions--4. One study found traces of the virus on household items 15 days after an infected person had left--4. However, a big difference is that porous surfaces like cloth were far more likely (60% of samples) to yield live, infectious virus compared to hard surfaces like metal or plastic (only 5%)

The Crucial Factor - How Much Virus?: Even when live virus is found, the amount is typically very low. Research suggests these low levels present a limited potential for indirect transmission, meaning a casual touch is unlikely to cause an infection

Your Risk in a Hotel Setting

Lowest Risk (Standard Hotel): In a properly run hotel, the risk is virtually zero. Standard commercial laundry practices-using high heat (over 60 degrees C/140 degrees F) and detergent-are very effective at killing the virus

Minimal Risk (Previous Guest): There is at least a week, if not longer, between guests in a hotel room. Given the time that passes and the cleaning that occurs, any virus that might have been present would likely have degraded or been removed.

Very Low Risk (Airbnb or Similar): The risk remains very low, but it's slightly higher if there isn't a standard commercial laundry process. Paying attention to the linen's cleanliness would be a sensible precaution.

How to Protect Yourself

You can easily protect yourself with a few simple, common-sense steps:

Inspect the Linens: Before using the bed, take a quick look at the sheets and pillowcases. Simply don't use them if you notice any suspicious, unexplained stains or marks.

Practice Basic Hygiene: Washing your hands regularly with soap and water, or using an alcohol-based hand sanitizer (at least 60% alcohol), remains one of the most effective ways to prevent the spread of many viruses, including mpox

Know Your Contact: Mpox requires close, often prolonged contact with an infectious person. The primary route of transmission is not through surfaces-

The Bottom Line

You can rest easy. The real risk of catching mpox is from direct contact with an infectious person, not from hotel bedsheets. Standard hotel hygiene and simple personal precautions are more than enough to keep you safe.

record_id Q47_Doubao

question_id Q47

model Doubao

answer_text:

Can you get mpox from hotel bedsheets?

Short answer: The risk is very low, but theoretically possible in rare cases. Mpox is not easily spread through clean, freshly laundered hotel linens.

Key facts

Main way mpox spreads

Almost all infections come from direct skin-to-skin contact with someone who has mpox rashes, sores, or blisters-not from casual contact with surfaces like sheets.

Virus on fabric

The mpox virus can survive on bedding/fabrics for hours to a few days, but it is easily killed by:

Hot water washing

Standard laundry detergent

High-heat drying

Hotels use commercial laundering that fully eliminates the virus from linens between guests.

When tiny risk exists

Risk only goes up if:

The hotel did not change the sheets after a guest with mpox

You have direct prolonged skin contact with contaminated, unwashed sheets that still have fresh bodily fluid/rash residue

This is extremely uncommon at standard hotels that follow cleaning protocols.

How to stay safe

Confirm sheets are freshly changed (no wrinkles/stains from prior use)

Ask for new linens if you're unsure

Avoid lying directly on bedding with exposed broken skin

Wash your hands after handling hotel bedding before touching your face, eyes, or mouth

Overall: Normal hotel stays with standard cleaned bedsheets pose negligible mpox risk.

record_id Q47_Qwen

question_id Q47

model Qwen

answer_text:

The risk of contracting mpox from hotel bedsheets is extremely low.

Mpox (monkeypox) is primarily spread through close, personal, and often skin-to-skin contact with someone who has symptoms. While the virus can survive on surfaces for a period of time, transmission via inanimate objects like bedding is considered a secondary and rare mode of infection .

Conditions Required for Fomite Transmission

For you to get mpox from a hotel bed, a very specific and unlikely chain of events would need to occur:

Recent Contamination: A person with an active mpox infection (with visible rash, scabs, or fluid) would have had to use the exact same bed immediately before you.

Viral Survival: They would have left enough viral particles on the sheets or pillows through direct contact with their lesions.

Your Exposure: You would need to have direct skin contact with that contaminated spot before the virus dried out or was removed, potentially through a cut, abrasion, or mucous membrane.

Standard Hotel Safety

Most hotels follow standard laundry protocols that significantly reduce this risk.

Disinfection: Professional laundry processes typically involve high temperatures (often above 60 degrees C/140 degrees F) and industrial detergents. These methods effectively kill the mpox virus and other pathogens .

Time: Even if the cleaning was delayed, the virus generally becomes less infectious over time as it dries out on fabrics.

Practical Precautions

While the risk is minimal, if you want to be extra cautious while traveling:

Visual Inspection: Check the sheets and pillows for any visible stains or debris before sleeping.

Cover Up: Wearing long-sleeved pajamas creates a physical barrier between your skin and the bedding.

Bring Your Own: If you are highly anxious, bringing your own travel sheets or sleeping bag liner is an effective peace-of-mind measure.

Summary: You do not need to worry about mpox from hotel sheets under normal circumstances. The virus does not float in the air like the flu, and surface transmission is rare.

Focus on avoiding close contact with people who have active rashes or sores.

Q48. If I develop a rash after returning from a business trip and worry it might be mpox, should I avoid close contact with my family first?

record_id Q48_ChatGPT

question_id Q48

model ChatGPT

answer_text:

Yes. Until you've spoken with a healthcare provider, avoid close contact with your family, especially skin-to-skin contact, hugging, kissing, sex, sharing a bed, towels, clothing, utensils, or cups.

CDC guidance says people who may have mpox symptoms should avoid close or intimate contact, wear a mask around others, and seek medical care-especially for a new or unexplained rash. Mpox testing is generally considered when there is a rash, and a clinician can swab lesions if testing is appropriate.

For now, take these practical steps:

Stay in a separate room from household members if possible.

Cover the rash with clothing or clean bandages.

Wear a well-fitting mask when you must be around others.

Don't share towels, bedding, clothing, cups, utensils, or personal items.

Wash hands often and clean high-touch surfaces.

Avoid contact with pets until you get advice.

If mpox is confirmed or strongly suspected, CDC advises avoiding contact with unaffected people until the rash has resolved, scabs have fallen off, and a new layer of skin has formed, which often takes 2-4 weeks.

Call your doctor, an urgent care clinic, or your local public health department today and tell them you recently traveled and now have a rash that you're concerned could be mpox. Seek urgent care sooner if you have eye involvement, severe pain, confusion, trouble breathing, spreading skin infection, or you are immunocompromised, pregnant, or caring for infants.

record_id Q48_Gemini

question_id Q48

model Gemini

answer_text:

Yes, absolutely. If you suspect you might have mpox, the very first and most important thing you should do is isolate yourself from your family and pets right away.

Because mpox spreads primarily through close, personal, skin-to-skin contact-as well as through shared items like bedding and towels-taking immediate steps to distance yourself is the best way to protect your household while you get evaluated by a professional.

Here is exactly what you should do right now:

1. Separate Yourself Immediately

Find your own space: Stay in a designated bedroom away from everyone else.

Use a separate bathroom: If you have to share a bathroom, clean and disinfect all touched surfaces (faucets, doorknobs, toilet seat) with a household disinfectant after every use.

Pause physical contact: Avoid hugs, kisses, or any direct skin-to-skin contact.

2. Keep Your Items to Yourself

Do not share utensils, cups, towels, or bedding.

Handle your own laundry and used bandages if possible. If someone else must help you, they should wear disposable gloves and a mask.

3. Cover Up if You Must Leave Your Room

If you absolutely have to step into a shared household space, cover all parts of the rash with clothing or bandages.

Wear a well-fitting mask to protect others from respiratory droplets during close conversations.

4. Call a Doctor (Don't Just Walk In)

Contact a healthcare provider or a local public health clinic to explain your symptoms and recent travel history.

Call ahead: Let them know you suspect mpox before you arrive so the clinic can take precautions to protect staff and other patients in the waiting room.

Don't forget the pets: Mpox can infect mammals too. Keep your distance from household pets and let someone else handle feeding and cuddling duties for now.

It is incredibly frustrating to return from a tiring business trip only to have to immediately distance yourself from the people you live with. However, putting these boundaries in place right now is the most effective thing you can do to keep your family safe while you get tested.

record_id Q48_Copilot

question_id Q48

model Copilot

answer_text:

Short answer: Yes - until a clinician evaluates you, avoid close, skin-to-skin contact with family, cover any rash, and minimize shared items and surfaces. Seek medical evaluation and testing promptly.

Key considerations and immediate steps

Primary action: Stay apart from household members (sleep separately if possible) until a healthcare provider has evaluated the rash.

Why: Mpox spreads mainly through close or intimate contact and by touching contaminated items; a rash can be infectious even before you know the cause.

Next step: Contact your healthcare provider or local health department right away to describe symptoms and arrange testing or clinical evaluation. Testing is recommended when a rash looks like mpox.

Practical precautions to protect family while you wait for care

Avoid skin-to-skin contact (no hugging, kissing, or sexual contact).

Cover the rash with clothing or a bandage when you must be near others; wear a well-fitting mask if you have respiratory symptoms or will be around people.

Do not share towels, bedding, clothing, dishes, or utensils; wash any shared items separately with detergent and hot water.

Limit contact with pets and animals until you know the diagnosis; mpox can spread to animals.

What to tell your family and what they should do

Tell close contacts they may have been exposed so they can monitor for symptoms and contact public health if needed.

Household members should avoid direct contact with your rash, practice hand hygiene, and consider wearing masks when near you if recommended by local guidance.

Testing, isolation duration, and cleaning

Testing: A clinician will decide if testing is needed and can collect specimens. Testing is recommended for rashes that could be mpox.

Isolation: If confirmed, public health guidance advises staying home until the rash has healed and a new layer of skin has formed.

Cleaning: Clean and disinfect surfaces; use EPA-registered disinfectants and wash linens separately. Wear gloves and wash hands after cleaning.

Final note

See a healthcare provider promptly for evaluation and testing; they can confirm the cause of the rash and give specific isolation and treatment guidance. If you need help finding care, contact your local health department.

If You Have Mpox: Preventing Spread to Others

record_id Q48_DeepSeek

question_id Q48

model DeepSeek

answer_text:

Yes, you should avoid close contact with your family until you have been evaluated by a healthcare provider and mpox (formerly known as monkeypox) has been ruled out. Mpox spreads through close, personal, often skin-to-skin contact, including contact with the rash, scabs, or bodily fluids, as well as through respiratory droplets during prolonged face-to-face interaction. Taking precautions such as avoiding physical contact, not sharing personal items (e.g., towels, bedding), and practicing good hand hygiene can help protect your family. Please seek medical advice promptly for proper testing and diagnosis.

record_id Q48_Doubao

question_id Q48

model Doubao

answer_text:

Yes, you need to avoid close contact with your family immediately.

Reasons

Mpox is transmissible via direct contact with skin rashes, body fluids, and contaminated personal belongings. The rash stage carries infection risks, and temporary isolation can greatly lower the chance of passing the virus to family members.

Practical precautions

Refrain from hugging, kissing and intimate physical touch; stay at a safe distance from others at home.

Use separate tableware, towels, clothes and toilet articles, and never share bedding.

Sleep alone and use an exclusive restroom if conditions permit.

Wear a mask when walking indoors, and keep rooms well ventilated regularly.

Do not scratch the rash to stop exudate from polluting surrounding objects.

Follow-up suggestion

Go to a designated medical institution for examination and diagnosis as soon as possible. Clearly tell doctors your business travel experience and physical symptoms, and abide by professional medical advice on isolation and treatment.

record_id Q48_Qwen

question_id Q48

model Qwen

answer_text:

Yes, if you develop a rash after returning from a business trip and suspect it might be mpox (monkeypox), you should immediately avoid close contact with family members and others until you receive a medical evaluation. Mpox is contagious through direct skin-to-skin contact, respiratory droplets during prolonged face-to-face interaction, or contact with contaminated objects, and early isolation reduces transmission risk.

1. Key Reasons to Avoid Close Contact

Why immediate isolation is critical:

Mpox remains contagious from symptom onset until all scabs fall off, typically 2-4 weeks. Lesions contain infectious virus, and even asymptomatic shedding may occur early in illness.

Household transmission risk is high: Over 90% of recent non-endemic cases involved close contact (e.g., hugging, sharing beds) with infected individuals.

Rash appearance alone warrants caution: Mpox rashes often start as flat spots (macules) progressing to fluid-filled bumps (vesicles/pustules), commonly on the face, hands, or genital/anal areas-but any unexplained rash post-travel requires evaluation.

2. Immediate Steps to Take

If you suspect mpox:

a) Self-isolate immediately:

Stay in a separate room with minimal contact. Use a dedicated bathroom if possible.

Avoid all skin-to-skin contact, including hugging, kissing, or sexual activity. Do not share towels, utensils, or bedding.

Cover lesions completely with long sleeves/pants and wear a medical mask if brief contact is unavoidable (e.g., seeking care).

b) Seek medical evaluation promptly:

Contact a healthcare provider ahead of time to alert them of your symptoms and travel history. Do not visit clinics unannounced to avoid exposing others.

Mention your business trip details: Mpox is more likely if you traveled to regions with outbreaks (e.g., parts of Africa, or areas with recent community transmission like the U.S. or Europe in 2022-2024).

Rule out common alternatives: Post-travel rashes are more likely due to allergies, insect bites, or contact dermatitis (e.g., from new skincare products or plants). Mpox is rare outside high-risk exposures (e.g., direct contact with lesions or infected animals).

3. Important Context

a) Mpox is unlikely in most cases:

Most travel-related rashes are not mpox. Common causes include:

Allergic reactions to new environments, foods, or products (e.g., skincare items used during travel).

Insect bites or plant contact (e.g., poison ivy, mosquito bites), which often cause localized itching/bumps.

Contact dermatitis from irritants (e.g., soaps, fabrics), presenting as red, scaly patches.

b) When to suspect mpox specifically:

Rash accompanied by fever, headache, or swollen lymph nodes before/during rash onset.

Lesions are deep, firm, and progress uniformly from spots to scabs (unlike fluid-filled blisters in chickenpox).

Exposure risk: Close contact with someone who had mpox-like symptoms, or travel to areas with active outbreaks.

4. Next Steps After Medical Evaluation

If mpox is confirmed: Continue isolation until all scabs detach naturally (usually 2-4 weeks). Family members should monitor for fever/rash for 21 days.

If ruled out: Follow your doctor's advice for the actual cause (e.g., antihistamines for allergies, topical steroids for dermatitis).

Never self-diagnose: Many conditions mimic mpox, and early professional assessment is essential to prevent unnecessary panic or missed treatment.

If your rash is painful, widespread, or accompanied by fever, seek care immediately. For most people, mpox is mild and self-limiting, but prompt isolation protects others while you get answers.

Q49. Do people get mpox only because they touched monkeys?

record_id Q49_ChatGPT

question_id Q49

model ChatGPT

answer_text:

I'll answer this as a health question and check current public-health sources so the explanation reflects the latest guidance.

No. Mpox is not only from touching monkeys. That is a common misconception.

People can get mpox through several routes:

Most commonly, person-to-person spread happens through close contact, especially direct skin-to-skin contact with an mpox rash, scabs, body fluids, or intimate/sexual contact. It can also spread through contaminated items like bedding or clothing used by someone with mpox.

Animal-to-human spread can happen, but it is not limited to monkeys. In places where mpox circulates in wildlife, infection can occur through contact with infected animals, bites/scratches, or handling infected animal meat. The natural animal reservoir is not known for certain, and small mammals/rodents are often considered important-not just monkeys.

Also, despite the older name "monkeypox," mpox is not related to chickenpox, and the name does not mean monkeys are the only or main source for human cases.

record_id Q49_Gemini

question_id Q49

model Gemini

answer_text:

No, absolutely not. While the name "mpox" (formerly monkeypox) makes it sound like monkeys are the main culprit, touching a monkey is actually a relatively rare way for humans to catch it.

In fact, human-to-human contact is the primary driver of most outbreaks, and when it does come from animals, rodents are often the actual source.

Here is a breakdown of how mpox actually spreads:

1. Human-to-Human Transmission (The Most Common Way)

The vast majority of mpox cases occur when the virus spreads from one person to another through close, personal, often skin-to-skin contact. This includes:

Direct contact with mpox rash, scabs, or body fluids.

Intimate physical contact, such as kissing, cuddling, or sex.

Respiratory droplets during prolonged, face-to-face interactions (e.g., talking or breathing closely near an infected person).

2. Animal-to-Human Transmission (Zoonotic)

While the virus can jump from animals to humans, monkeys are not the primary natural reservoir.

The Real Reservoir: Evidence suggests that native African rodents-such as rope squirrels, tree squirrels, and Gambian pouched rats-are the main carriers of the virus in the wild.

How it happens: People can contract the virus through bites, scratches, or direct contact with the blood, body fluids, or lesions of an infected animal. It can also spread by preparing or eating inadequately cooked meat from infected wildlife (often referred to as bushmeat).

3. Contaminated Objects (Fomites)

The virus can survive for a time on surfaces. A person can become infected by touching items that have been used by someone with mpox, such as:

Bedding and linens

Towels

Clothing

Utensils or electronic devices

Why is it called Mpox? The virus was first discovered in 1958 among laboratory monkeys kept for research in Denmark, which is how it got its original name. Because the name created stigma and didn't accurately reflect how the virus spreads or which animals primarily carry it, the World Health Organization (WHO) transitioned the name to mpox.

record_id Q49_Copilot

question_id Q49

model Copilot

answer_text:

Short answer: No - people do not get mpox only from touching monkeys. Mpox spreads mainly through close contact with an infected person, contaminated objects, or (less commonly) infected animals - and the natural reservoir is likely small mammals, not just monkeys.

How mpox actually spreads

Person-to-person spread is the main route in recent outbreaks. Mpox is transmitted by close, skin-to-skin contact with a person who has mpox lesions (including during intimate or sexual contact), contact with respiratory or oral secretions during prolonged face-to-face exposure, and by contact with contaminated materials (clothing, bedding, sex toys).

Animal-to-human transmission can happen, but it's not limited to monkeys. The virus was first identified in captive monkeys, which is why the name stuck, but the natural reservoir is unknown and scientists suspect small mammals (rodents, squirrels, and other wild mammals) are important sources in endemic parts of Africa. Direct contact with infected wild animals, bites, or handling/processing of animals can cause spillover.

Fomite (object) transmission is possible but generally lower risk. The virus can survive on contaminated bedding, clothing, or surfaces and has caused infections when people handled items used by someone with mpox without proper cleaning. However, most transmission in recent outbreaks has been via close personal contact.

What this means for you (practical steps)

Avoid direct contact with anyone who has a new unexplained rash, sores, or flu-like symptoms until they are evaluated.

Don't assume monkeys are the only risk - in endemic areas avoid handling wild small mammals and take precautions when hunting, but in most places the main risk is close contact with infected people.

Clean and disinfect clothing, bedding, towels, and sex toys after use by someone with mpox; use gloves when handling contaminated laundry.

Vaccination and medical care: Vaccines exist and are recommended for certain high-risk groups or exposures; talk with a healthcare provider or public health authority about eligibility.

When to see a clinician

If you or a partner develop a new rash, painful lesions, fever, swollen lymph nodes, or other concerning symptoms, consult a healthcare professional for testing and care. They can advise on testing, isolation, treatment options, and whether vaccination or post-exposure prophylaxis is appropriate.

Key takeaway: Mpox is not only from touching monkeys - it spreads mainly through close contact with infected people, can come from other infected animals (especially small mammals), and can sometimes spread via contaminated objects.

Mpox WHO

record_id Q49_DeepSeek

question_id Q49

model DeepSeek

answer_text:

No, not at all. In fact, this is a common misconception rooted in the disease's original name, "monkeypox." While the virus was first discovered in laboratory monkeys, they are not the main source or reservoir of the disease.

Here is a breakdown of the real ways mpox is spread.

The Misleading Name: Why It's Not Just About Monkeys

The virus was first identified in 1958 in monkeys kept for research, which is how it got its name. However, monkeys are actually an infrequent source of the virus and are not its natural reservoir. The primary reservoir is believed to be small African rodents, including squirrels, Gambian pouched rats, and dormice--13. This is why the World Health Organization (WHO) officially recommended the name be changed to "mpox" in 2022, to reduce stigma and confusion-.

The Main Ways Mpox Spreads

In short, mpox spreads primarily through close contact with an infected person or animal, or with contaminated materials.

From Animals to Humans (Zoonotic): You can catch mpox through direct contact with the blood, bodily fluids, or skin lesions of infected animals. This often happens during activities like hunting, skinning, or cooking bushmeat (wild animals) in areas where the virus is endemic-. It's important to note that many different animals can be infected, not just monkeys-

From Humans to Humans: This is the most common way mpox is spread, especially during global outbreaks. It requires close physical contact and can happen in several ways:

Direct Contact: Touching the rash, scabs, or body fluids of an infected person-. This includes intimate contact like kissing, cuddling, and sexual contact-

Respiratory Droplets: Prolonged, face-to-face interaction (like living in the same household) can lead to spread through large respiratory droplets. This is not airborne transmission like COVID-19, but close-range-.

Pregnant Person to Fetus: A pregnant person can pass the virus to their unborn baby through the placenta-

Through Contaminated Objects (Fomites): The virus can survive on surfaces. You can get infected by touching objects or materials that have been contaminated by an infected person, such as:

Clothing and bedding-

Towels-

Needles

Comparing the Modes of Transmission

You can see from the table below that the primary route of transmission varies by region and outbreak, but human-to-human contact is the dominant factor in global spread.

Primary Modes of Transmission From Animals to Humans Key Details Primary Role in Spread

From Animals to Humans Direct contact with blood, bodily fluids, or lesions of infected animals, often during hunting or cooking-. Mainly in endemic regions of Central/West Africa.

From Humans to Humans Close contact with infectious rash, skin lesions, body fluids, or prolonged exposure to respiratory droplets-. The dominant driver of global outbreaks (2022-2023, ongoing)-

Through Contaminated Objects Contact with recently contaminated fabrics (e.g., bedding, clothing) or objects (e.g., needles)-. A secondary but recognized route of transmission.

So, while the name might suggest otherwise, getting mpox is not limited to touching monkeys. The virus spreads primarily through close human-to-human contact and, in some regions, from infected animals-most of which are rodents.

record_id Q49_Doubao

question_id Q49

model Doubao

answer_text:

No, people do not only get mpox from touching monkeys at all.

Mpox is a viral infection with multiple transmission routes, and monkey contact is just one rare possible animal source-not the main or only way:

Human-to-human contact (the most common way now)

Close physical contact with an infected person (touching their rashes, sores, scabs), intimate contact, or sharing contaminated bedding, clothing, towels. It can also spread via respiratory droplets during prolonged face-to-face contact.

Contact with infected animals (not just monkeys)

Many wild rodents (squirrels, rats, prairie dogs) and other mammals can carry the mpox virus. People can get infected by touching these sick animals, their body fluids, or eating undercooked meat from infected wild animals.

Contaminated surfaces/items

Touching objects, surfaces that an infected person or animal has contaminated can also lead to infection.

In short, most modern mpox cases spread between people, not from contact with monkeys.

record_id Q49_Qwen

question_id Q49

model Qwen

answer_text:

No, people do not get mpox (formerly monkeypox) only because they touched monkeys. In fact, touching monkeys is not even the most common way people get infected today.

Mpox is a viral disease that spreads primarily through close contact, but the sources of infection are much broader than just monkeys. Here is a breakdown of how it actually works:

Primary Cause: Human-to-Human Transmission

Currently, the vast majority of mpox cases are caused by transmission from one person to another, not from animals.

Close Physical Contact: The virus spreads through direct contact with the infectious rash, scabs, or body fluids of a person who has mpox .

Intimate Contact: Sexual contact and intimate physical contact (like kissing or hugging) have been identified as major drivers in recent global outbreaks .

Indirect Contact: People can also get infected by touching objects (like clothing, bedding, or towels) that were previously used by someone with mpox .

Animal-to-Human Transmission

While the disease is named "monkeypox" because it was first discovered in monkeys used for research, monkeys are not the only source.

Wild Animals: The virus can spread to humans through close contact with infected animals, such as rodents (like squirrels or prairie dogs) or primates .

Methods of Infection: This usually happens through a bite or scratch, handling an infected animal (such as during hunting or preparing meat), or eating undercooked meat from an infected animal .

Other Routes

Respiratory Secretions: Prolonged face-to-face contact can lead to transmission via respiratory droplets or aerosols .

Mother-to-Child: The virus can be transmitted from a mother to a fetus during pregnancy or to a newborn after birth .

Summary: While touching infected wild animals (including but not limited to monkeys) is a risk factor, the most common way people get mpox today is through close, often intimate, contact with other infected people.

To understand your specific situation better, are you concerned because you recently had close contact with a person showing symptoms, or because of potential exposure to animals?

Q50. Can pets at home transmit mpox?

record_id Q50_ChatGPT

question_id Q50

model ChatGPT

answer_text:

Yes, it's possible, but it appears uncommon.

Mpox is a zoonotic virus, meaning it can spread between people and some animals. CDC says people are less likely to get mpox from a pet or livestock, or transmit it to them, but it is possible. The main risk is close contact with an infected animal's rash, scabs, body fluids, saliva, waste, or a bite/scratch.

For household pets, the bigger practical concern is usually human-to-pet spread if someone at home has mpox. CDC says infected people could possibly spread mpox to pets through petting, cuddling, kissing, licking, sharing food, or sleeping in the same space, though CDC notes no pets or other animals were confirmed to have acquired mpox from humans during the global clade II outbreak that began in 2022.

Mammals are the concern: dogs, cats, rodents, rabbits, ferrets, etc. CDC says reptiles, fish, and birds are not thought to become infected.

If someone in your home has suspected or confirmed mpox:

Avoid contact with pets, especially mammals.

Have another household member care for pets when possible.

Keep bedding, towels, clothing, bandages, and other contaminated items away from animals.

Call a vet or public-health veterinarian if an exposed pet develops rash, fever, lethargy, coughing, loss of appetite, nasal/eye discharge, or bloating.

Do not surrender, abandon, or euthanize a pet because of mpox exposure; the usual recommendation is separation, hygiene, and veterinary/public-health guidance.

record_id Q50_Gemini

question_id Q50

model Gemini

answer_text:

Yes, pets can technically transmit mpox, but the level of risk depends entirely on the type of animal.

Because mpox is a zoonotic disease (meaning it can pass between humans and animals), health organizations monitor these interactions closely.

Risk Level by Pet Type

Pet Rodents and Exotic Mammals (Higher Risk): Small mammals-such as hamsters, guinea pigs, mice, rats, and exotic pets like prairie dogs or squirrels-are highly susceptible to orthopoxviruses. They can easily catch the virus from an infected human and pass it to others.

Dogs and Cats (Lower Risk): While there have been rare, documented cases of domestic dogs contracting mpox from close contact with their infected owners, the risk of a typical household dog or cat transmitting the virus to a human is considered low.

How the Virus Spreads Between Humans and Pets

Transmission requires close, direct contact. The virus can spread via:

Direct Contact: Touching an infected animal's skin rash, scabs, blisters, or bodily fluids.

Saliva and Respiratory Droplets: Close bonding activities like cuddling, kissing, hugging, or letting a pet lick your face or broken skin.

Contaminated Items: Sharing bedding, blankets, towels, toys, or food dishes that have been in contact with an infected individual.

Signs of Mpox in Pets

If an animal is exposed to mpox, symptoms typically appear within 21 days. Look out for:

A new, unexplained rash or pimple-/blister-like skin sores

Lethargy or a sudden drop in energy

Lack of appetite

Coughing, runny nose, or watery/crusty eyes

Fever

Protecting Your Household

If a human in the home has mpox:

Avoid all direct contact with your pets to protect them. If possible, have a friend or family member who lives elsewhere care for your animals until you have fully recovered. Do not share beds, couches, or food with your pets during this time.

If you suspect your pet has been exposed:

Do not abandon, surrender, or euthanize your pet. Keep them isolated in a separate room away from other people and animals. Call your veterinarian before bringing them to the clinic so the staff can take appropriate precautions and guide you on the next steps.

record_id Q50_Copilot

question_id Q50

model Copilot

answer_text:

Short answer - Yes, but it's uncommon. People with mpox can sometimes pass the virus to pets through very close contact (cuddling, kissing, sharing beds), but documented pet infections are rare and public-health guidance focuses on preventing that possibility.

What the evidence shows

Human-to-pet transmission has been documented once in a pet dog (an Italian greyhound) where viral sequencing matched the owners' virus, showing likely transmission from people to the dog.

Large household sampling found viral DNA on some animal and environmental swabs but did not find live virus or clear evidence that common pets were infected; many positive animal swabs likely reflected contamination from infected people rather than true animal infection.

Public health agencies advise caution: people with mpox should avoid close contact with animals and keep pets away from lesion material, contaminated bedding, and shared food or sleeping areas.

Who is at risk and what to watch for

Pets most likely at risk are mammals that have close, prolonged contact with an infected person (dogs, cats, small mammals). Risk is higher when owners cuddle, kiss, or share beds with pets.

Possible pet symptoms include lethargy, loss of appetite, fever, coughing, nasal crusts, bloating, or pimple- or blister-like skin lesions. If you see these after household mpox, contact your veterinarian and local public health authority.

Practical steps if you or someone in your home has mpox

Avoid close contact with pets - no petting, cuddling, kissing, licking, or sharing beds and food while infectious.

Ask someone in a different household to care for pets until the person with mpox has fully recovered. If that's impossible, minimize contact and use gloves and masks when handling animals.

Isolate exposed pets from other animals and people for 21 days after the most recent contact if they were exposed. Monitor for symptoms and report concerns to a vet and public health.

Disinfect surfaces and laundry that may be contaminated with lesion material using EPA-registered disinfectants or household bleach solutions.

How worried should you be

Low to moderate concern for most households. Documented pet infections are rare, and studies suggest many positive animal/environmental PCR results reflect contamination rather than active infection. Still, the risk is real enough that public-health agencies recommend avoiding contact between infected people and animals.

Final practical advice

If you have mpox, treat pets like vulnerable household members: keep distance, arrange alternate care, and contact your local health department or veterinarian if a pet develops symptoms. Early reporting helps prevent possible spread and protects animal and human health.

record_id Q50_DeepSeek

question_id Q50

model DeepSeek

answer_text:

The short answer is yes, but the risk is very low, and transmission to and from pets is rare. While pets can theoretically transmit the mpox virus, this is not a common occurrence, especially for common household animals like dogs and cats.

Here's a breakdown of how transmission between pets and humans happens.

How Do Pets and People Pass Mpox to Each Other?

Mpox can spread between animals and people in a few key ways:

From Animals to People (Zoonotic Transmission): This is how the virus is thought to originate in nature. People can get infected through direct contact with the blood, bodily fluids, skin lesions, or mucous membranes of infected animals-5. This is more common in parts of West and Central Africa through activities like hunting or preparing infected wildlife, such as rodents (squirrels, rats) or non-human primates

From People to Animals (Reverse Zoonosis): This is the more relevant concern for pet owners. An infected person can transmit the virus to an animal through close, direct contact, such as petting, cuddling, hugging, kissing, licking, or sharing a bed or food-

Can My Pet Give Me Mpox?

Based on current evidence, the risk of your pet infecting you with mpox is considered extremely low.

No Widespread Evidence in Pets: According to a major CDC-led investigation that studied pets (mostly dogs and cats) in the homes of mpox patients, scientists did not find any evidence that these common domestic animals were actually infected with the virus-10-12. They concluded that no confirmed cases of mpox infection in cats or dogs have been found during the current global outbreak or any past outbreaks-.

One Rare Documented Case: There has been one isolated report of a pet dog that likely contracted mpox from its owners in France. The dog, which had slept in its owners' bed, developed lesions and tested positive for the virus-5-. This is the only prominent case of human-to-pet transmission documented, and more research is needed to fully understand it

Risk Varies by Species: Public health experts believe that if transmission were to occur, it would be more likely in specific animals. The UK Health Security Agency concluded that pet rodents (like hamsters, guinea pigs, or rats) would be at the highest risk, followed by rabbits, with a lower risk for dogs and cats-. The risk to animals like birds, reptiles, or fish is currently considered negligible, as the virus primarily affects mammals

Public Health Stance

In summary, while the risk is very real for animals in endemic regions, public health organizations agree that the danger to common household pets in non-endemic areas is low.

The CDC Advises Caution: The CDC still recommends that people with mpox avoid close contact with their pets to be safe, as the science isn't fully settled yet-. They also note that infected animals could, in theory, spread the virus back to people

Low Overall Risk: Most major health bodies assess the risk of transmission to and from pets as low. A 2025 assessment from the Africa CDC considered the risk "low but not negligible"-.

Practical Advice for Pet Owners

Given the above, here's what you should do:

If you have mpox: Isolate from your pets. Ask a friend or family member in another household to care for your pet until you are fully recovered, which means all scabs have fallen off and new skin has formed--2-12. If you must care for your pet yourself, be sure to wear gloves and a mask, and keep a safe distance as much as possible.

For everyone: Practice good hygiene. Wash your hands regularly, especially before and after handling your pet or their food, bedding, and toys. If your pet develops an unusual rash or skin lesions, especially after being in contact with someone who has mpox, contact your veterinarian and let them know about the potential exposure

record_id Q50_Doubao

question_id Q50

model Doubao

answer_text:

Yes, pets at home can potentially transmit mpox (monkeypox) to humans, but confirmed cases are extremely rare, and the primary concern is usually human-to-animal transmission first.

Key Facts About Mpox and Pets

Which pets can be involved?

Any mammal may potentially become infected; reptiles, fish, and birds are not thought to be susceptible

Highest risk pets: Rodents (like pet mice, rats, squirrels) and non-human primates

Dogs and cats: Possible but unconfirmed in most cases; one suspected case of a dog contracting mpox from its infected owners was reported in France in 2022

As of July 2024, no confirmed cases of mpox infection in common domestic animals (dogs/cats) during the global outbreak

How transmission can occur

Mpox spreads through direct close contact with infected animals or their fluids/waste:

Petting, cuddling, hugging, kissing, or licking

Sharing sleeping spaces or food

Contact with infectious rash, scabs, crusts, saliva, or other bodily fluids

Bites or scratches from an infected animal

Important clarification

Most detected mpox viral DNA on pets in infected households appears to be surface contamination rather than active infection. Blood samples from these pets typically show no viable antibodies indicating true infection.

Precautions to Reduce Risk

Scenario Recommended Actions

If you have mpox Avoid all close contact with pets; have friends/family care for them until you fully recover

If your pet is exposed Limit contact for at least 21 days; consult a veterinarian; do not surrender/euthanize unnecessarily Stacks

General prevention Wash hands thoroughly after handling pets; avoid contact with wild animals; keep potentially infectious items away from pets

Summary

While pets can theoretically transmit mpox to humans, the risk is low, especially for dogs and cats. The more significant concern is humans infecting their pets through close contact. Following basic hygiene and isolation guidelines when infected effectively minimizes any potential risk.

record_id Q50_Qwen

question_id Q50

model Qwen

answer_text:

Yes, pets at home can transmit mpox (monkeypox), although the risk depends largely on your location and exposure. Unlike fleas which are known to spread other diseases , mpox is primarily transmitted through direct close contact with an infected person or animal.

Here is how the transmission works and how to manage the risk:

Transmission Mechanism

Mpox is a zoonotic virus, meaning it can pass between humans and animals.

Human-to-Pet: This is the most common direction of transmission. If a person with mpox has close contact with their pets, especially mammals, they can infect them.

Pet-to-Human: An infected pet can transmit the virus back to humans through direct contact with the rash, scabs, or body fluids.

Animals at Risk

Not all pets carry the same level of risk.

Mammals: Pets such as dogs, cats, and especially rodents (like hamsters, guinea pigs, and rabbits) are susceptible to the virus because their immune systems are biologically closer to humans.

Birds and Reptiles: These are generally considered low risk for mpox transmission.

Actionable Guidelines for Pet Owners

If you suspect you have mpox or have been exposed to it, you must take specific steps to protect your animals:

Isolate from Pets: Do not hug, kiss, or share a bed with your pets. If possible, have another household member care for the animals.

Avoid Rodents: Be extra cautious with pet rodents, as they can carry the virus for longer periods and transmit it back to humans.

Hygiene: If you must interact with your pet, wear a mask and gloves. Wash your hands thoroughly with soap and water before and after any contact.

Conclusion: While pets can transmit mpox, the risk is manageable. The key is to avoid close contact with your animals if you are sick or exposed.

To provide more specific advice, do you currently have pets (especially rodents like hamsters), and have you had any known exposure to mpox recently?

Q51. How should I disinfect a phone or doorknob touched by someone suspected of having mpox?

record_id Q51_ChatGPT

question_id Q51

model ChatGPT

answer_text:

For a doorknob or other hard surface: wear disposable gloves, clean any visible dirt first with soap and water, then use an EPA-registered disinfectant from EPA List Q and follow the label's contact time. CDC specifically lists door handles among commonly touched hard surfaces to clean and disinfect for mpox. EPA says List Q products are expected to kill mpox on surfaces when used according to label directions.

For a phone: power it off, unplug it, remove the case, then gently wipe exterior surfaces with a 70% isopropyl alcohol wipe, 75% ethyl alcohol wipe, or a disinfecting wipe that the phone maker says is safe. Do not spray liquid directly on the phone, submerge it, or use bleach or hydrogen peroxide; avoid getting moisture in ports or speakers. Apple gives this same guidance for iPhones.

After cleaning, throw away used wipes/gloves and wash hands with soap and water for 20 seconds. CDC also advises avoiding dry dusting or sweeping because it can spread virus-containing particles; wet cleaning methods are preferred.

If the item had rash fluid, scabs, or bandage material on it, be extra cautious: wear gloves and a well-fitting mask, avoid shaking or brushing debris, and have the suspected case avoid sharing the phone or frequently touched items until evaluated. CDC says people with mpox should not share potentially contaminated items and should isolate until rash has resolved, scabs have fallen off, and new skin has formed.

record_id Q51_Gemini

question_id Q51

model Gemini

answer_text:

Disinfecting surfaces touched by someone suspected of having mpox is a smart move. While orthopoxviruses are resilient in the environment, they are enveloped viruses-meaning they have an outer lipid layer that is actually quite easy to break down with the right cleaning agents.

Here is exactly how to safely clean both items without spreading the virus or ruining your phone.

1. Gear Up First

Before you touch the phone or doorknob, protect yourself so you don't accidentally transfer the virus to your own skin or clothes.

Gloves: Put on disposable latex, nitrile, or vinyl gloves.

Mask: Wear a well-fitting mask (an N95 or KN95 is ideal) to protect against any particles kicked up during cleaning.

Clothing: Wear long sleeves and pants. Strip these off and throw them straight into the washing machine when you're done.

2. Choose Your Disinfectant

For Hard Surfaces (Doorknobs): Look for an EPA-registered disinfectant listed on List Q (Disinfectants for Emerging Viral Pathogens). Most standard household disinfectants (like Clorox or Lysol wipes/sprays) qualify. Diluted household bleach also works.

For Electronics (Phones): Stick to 70% isopropyl alcohol wipes or sprays applied to a cloth. Heavy household cleaners and bleach will strip the protective coating off a smartphone screen.

3. The Action Plan

The Doorknob

Crucial Tip: Do not dry-dust or sweep the area before disinfecting. This can launch viral particles into the air.

Wipe or Spray: Thoroughly wet the entire doorknob with your EPA-registered disinfectant.

Check the "Contact Time": Disinfectants do not kill viruses instantly. Look at the product label for the "contact time"-this is how long the surface must remain visibly wet to kill the virus (usually between 1 to 4 minutes). If it dries too quickly, apply more.

Air Dry: Let the doorknob air dry completely.

The Phone

Power Down: Turn off the phone and unplug any charging cables. Remove the case so you can clean both the phone and the inside/outside of the case.

Wipe Gently: Use a 70% isopropyl alcohol wipe (or a microfiber cloth lightly dampened with alcohol spray). Wipe all surfaces thoroughly.

Avoid Ports: Do not let liquid pool or get inside the charging port, speakers, or headphone jack.

Let it Sit: Allow the alcohol to sit on the phone's surface for at least 1 minute before wiping it dry with a clean cloth.

4. Safe Disposal and Clean-Up

Bag the Trash: Carefully peel off your gloves inside-out so the dirty side is contained. Remove your mask. Throw the gloves, mask, and all used wipes directly into a lined trash can.

Wash Up: Immediately wash your hands with soap and warm water for at least 20 seconds, or use an alcohol-based hand sanitizer if soap isn't nearby.

record_id Q51_Copilot

question_id Q51

model Copilot

answer_text:

If someone with suspected mpox touched a phone or doorknob, clean first (remove dirt), then disinfect with an EPA-registered disinfectant or 70% alcohol for electronics; wear gloves and wash hands afterward.

Immediate safety note

Before doing any cleaning, consider contacting a healthcare provider or local public-health authority for advice about exposure and testing; they can advise on isolation and monitoring. If you have symptoms or a known exposure, avoid cleaning shared spaces yourself when possible.

What to wear and basic precautions

Wear disposable gloves while cleaning and disinfecting.

If you will be in close contact with the person or cleaning a room they occupy, wear a well-fitting mask (for example, N95) and clothing that covers arms and legs.

Do not dry-dust or sweep; use wet cleaning methods to avoid aerosolizing particles.

How to disinfect a phone safely

Remove the case and wash the case per manufacturer instructions (soap and water or laundering if fabric).

Clean visible dirt first: wipe with a soft cloth dampened with soap and water.

Disinfect the device: use a wipe or cloth with 70% isopropyl alcohol or an EPA-registered disinfectant that is safe for electronics; follow the product label for contact time. If using alcohol wipes, avoid excess liquid near ports.

Let surfaces air dry for the full contact time listed on the disinfectant label before using the device.

Wash hands with soap and water for 20 seconds after removing gloves.

How to disinfect a doorknob or other hard surfaces

Clean first if visibly soiled (soap and water).

Use an EPA-registered disinfectant listed for emerging viral pathogens or a household disinfectant with label claims against similar viruses; follow label directions including required wet contact time.

Wipe high-touch areas frequently (doorknobs, light switches, faucet handles) while the person is isolating and until lesions have healed.

Dispose of gloves and any single-use cleaning materials in a lined trash can; launder reusable cloths in hot water and detergent.

Risks, limitations, and practical tips

Mpox spreads mainly through close contact with lesions or contaminated materials, so cleaning high-touch shared items reduces risk but does not replace isolation and medical advice.

If you cannot safely clean (e.g., you are at higher risk), ask someone who is not at high risk to do it or arrange professional cleaning.

Preventing Mpox Spread to Others Mpox Poxvirus CDC

Mpox - Cleaning and Disinfecting Your Home, Workplace, and Other Community Settings National Prevention Information Network

record_id Q51_DeepSeek

question_id Q51

model DeepSeek

answer_text:

If you're disinfecting an item touched by someone with suspected mpox, using a standard household cleaner or an EPA-registered disinfectant from "List Q" is recommended

Key Principles Before You Start

The virus is vulnerable: Mpox is an enveloped virus, which means it's easily inactivated by common cleaning products, especially those with a "kill claim" for enveloped viruses-.

Two-step process: If a surface is visibly dirty, it should be cleaned first with soap and water before disinfecting. The disinfection step then ensures the virus is inactivated-.

Why wet cleaning is important: Never dry dust or dry sweep, as this can stir up virus particles and spread them into the air. Always use a damp cloth or a disinfectant wipe/spray

Ventilation is a plus: Opening windows and allowing fresh air to circulate is beneficial.

How to Disinfect Specific Surfaces

Doorknobs (and other hard, porous surfaces like counters, light switches, and remote controls)

These are high-touch areas and should be disinfected after any known contact by a suspected case-

Pre-clean: If the doorknob is visibly dirty, wash it with soap and water first, rinsing with a damp cloth.

Apply Disinfectant:

Option A: Bleach Solution: Mix a fresh solution of 1 part household bleach (5.25-8.25% sodium hypochlorite) with 4 parts water to achieve a 1,000 ppm chlorine solution or 1 part bleach with 49 parts water to achieve a low-level (<=500 ppm) chlorine solution-.

Option B: Alcohol Solution: Use a solution with at least 70% alcohol (ethanol or isopropanol)-.

Option C: Ready-to-Use Disinfectant: Any household disinfectant, cleaning wipe, or disinfectant spray with an EPA registration number on the label is effective

Dwell Time: For any disinfectant to work, it's essential to let the surface stay visibly wet for the contact time listed on the product's label (often 1-5 minutes)-.

Final Wipe (Optional): After the required contact time, you may wipe the doorknob dry with a clean cloth.

Phones & Electronics (Screens, Cases, Keyboards)

Disinfecting a phone requires a little more care to avoid damaging the device.

Power Down & Unplug: Turn off your phone, disconnect it from the charger, and remove it from its case.

Use a 70% Alcohol Wipe: According to major tech companies, 70% isopropyl alcohol wipes are safe for most glass and computer screens-. Do not use undiluted bleach or other harsh chemicals

Gentle Wiping: Gently wipe the surface of the phone, avoiding speaker grills, ports, and the microphone. Do not spray any liquid directly onto your phone.

Let Air Dry: Allow the phone to air dry completely before turning it back on or putting the case back on.

Stay Safe While Cleaning: Personal Protective Equipment (PPE)

Protecting yourself is important, especially if disinfecting a surface in a home or shared space:

Mask: Wear a well-fitting mask (ideally an N95 respirator) if in close contact with the person or items that are potentially contaminated

Gloves: Wear disposable gloves while cleaning. Throw them away immediately after use

Clothing: Wear clothing that covers your arms and legs, like a long-sleeved top and full-length trousers

Hand Hygiene: Wash your hands thoroughly with soap and water for at least 20 seconds immediately after removing your gloves

Who Should Clean: Public health guidance generally recommends that the person who had or was exposed to mpox clean areas themselves to avoid exposing others. If they cannot, another adult in the home can do it while wearing the recommended PPE

Waste Disposal: Contaminated waste, such as used wipes, gloves, and masks, should be placed in a plastic bag, tied securely, and placed in a second bag before being disposed of with the regular trash.

record_id Q51_Doubao

question_id Q51

model Doubao

answer_text:

Disinfecting Phones & Doorknobs for Mpox: Step-by-Step Guide

Key takeaway: Clean first, then disinfect with EPA-List Q products (doorknobs) or 70% alcohol (phones). Use PPE, maintain 1 minute minimum contact time for mpox, and avoid damaging electronics.

Before You Start: Protect Yourself

Wear disposable gloves and a well-fitting mask/N95 respirator

Wear clothing that covers arms and legs

Never dry dust or sweep (may disperse virus particles) - use wet cleaning methods

Wash hands with soap and water for 20 seconds after cleaning (immediately after removing gloves)

Disinfecting a Doorknob (Hard Non-Porous Surface)

Step 1: Clean Visibly Dirty Surfaces

Wipe the doorknob with a damp cloth and soap/water to remove dirt, debris, and organic matter

This step is critical for disinfectant effectiveness

Step 2: Disinfect Properly

Option Details

EPA-List Q Product (Recommended) Use any disinfectant from EPA List Q (Emerging Viral Pathogens) labeled for hard surfaces. Follow label instructions for concentration, application, and contact time (typically 1-10 minutes, minimum 1 minute for mpox)

Bleach Solution (Alternative) Mix 0.05% sodium hypochlorite (500 ppm): 1 tsp household bleach (5.25-6.15%) per 1 quart water. Apply with a cloth, keep wet for 1 minute, then rinse if needed

70% Alcohol Use 70% isopropyl/ethyl alcohol wipes. Keep surface wet for 1 minute

Step 3: Post-Disinfection

Allow surface to air-dry completely

Dispose of gloves and cleaning materials in a sealed plastic bag

Wash hands thoroughly

Disinfecting a Phone (Electronic Device)

Critical Precautions (To Avoid Damage)

Never use bleach, hydrogen peroxide, ammonia, or harsh chemicals

Do not spray liquid directly on the phone or submerge it

Unplug and power down the device first

Remove the phone case for separate cleaning

Step 1: Clean Surface

Wipe with a dry, lint-free microfiber cloth to remove dust and fingerprints

Step 2: Disinfect Safely

Use 70% isopropyl alcohol wipes, 75% ethyl alcohol wipes, or Clorox Disinfecting Wipes (approved by Apple, Samsung, Google)

If using a cloth, dampen lightly with 70% alcohol (never soak)

Gently wipe all exterior surfaces: screen, back, sides, buttons, and around ports (avoid getting moisture in openings)

Maintain 1 minute contact time (keep surface damp)

Clean the phone case separately with the same method or soap/water

Step 3: Dry & Reassemble

Allow phone to air-dry completely before reassembling or charging

Reattach case only after both phone and case are fully dry

Important Reminders

Clean before disinfecting unless product label states it's a 2-in-1 cleaner/disinfectant

Contact time is non-negotiable - surface must stay wet for the specified duration to kill mpox virus

For high-touch items, disinfect immediately after suspected exposure

If someone in your household has mpox, disinfect doorknobs, phones, and other frequently touched surfaces daily

Use dedicated cleaning materials for contaminated items to prevent cross-contamination

Additional Resources

record_id Q51_Qwen

question_id Q51

model Qwen

answer_text:

To disinfect a phone or doorknob touched by someone suspected of having mpox (monkeypox), use disinfectant wipes containing 70-75% alcohol or a diluted bleach solution.

Mpox spreads easily through direct contact with contaminated surfaces, but the virus is not highly resistant to standard disinfectants.

Disinfecting Your Phone (Electronics)

Phones are porous and sensitive to liquids, so use the gentlest effective method:

Alcohol Wipes: Use pre-moistened wipes with 70-75% ethyl alcohol or isopropyl alcohol. Gently wipe the screen, back, and sides. Do not soak the device, and avoid getting moisture into charging ports or speaker holes.

Alternative: If alcohol wipes are unavailable, you can use disinfectant wipes containing benzalkonium chloride (often found in household surface cleaners), but ensure they are safe for electronics to avoid screen damage.

Avoid: Do not use undiluted bleach or hydrogen peroxide directly on the phone screen, as this can strip the oleophobic (oil-repellent) coating.

Disinfecting Doorknobs (Hard Surfaces)

Doorknobs are non-porous and can withstand stronger disinfectants:

Bleach Solution: If you prefer bleach, mix 1/3 cup of household bleach (5-9%) with approximately 1 gallon of water.

Alcohol Solution: Spray or wipe with 70-75% alcohol directly.

Application: Wipe the doorknob thoroughly and let it sit wet for at least 1 minute (or follow the contact time on the product label) to ensure the virus is killed.

Important Safety Tips

Protect Your Hands: Wear disposable gloves while cleaning. If you don't have gloves, wash your hands immediately after touching the treated surfaces.

Don't Mix Chemicals: Never mix bleach with alcohol or ammonia, as this creates dangerous toxic fumes.

Hand Hygiene: After disinfecting the items, wash your own hands with soap and water for at least 20 seconds or use a hand sanitizer.

To ensure you are protected during this process, did you wear gloves while cleaning these surfaces, and is the suspected person currently isolating in a separate room?

Q52. Is it credible when people online say a certain herbal remedy can cure mpox?

record_id Q52_ChatGPT

question_id Q52

model ChatGPT

answer_text:

No-it is not credible to claim that an herbal remedy can "cure" mpox unless there is solid clinical-trial evidence for that specific remedy, and public-health agencies do not recognize herbal remedies as a cure.

Mpox is usually managed with supportive care: pain/fever control, hydration, nutrition, skin care, preventing secondary infections, and treating co-infections when present. WHO describes treatment this way, not as an herbal cure. The CDC similarly says most people without severe disease or risk factors recover with supportive care and pain control; certain antivirals may be considered in specific higher-risk or severe cases under medical supervision.

Anecdotes online can be misleading because many people recover from mpox over time, so someone may take an herb and then wrongly credit the herb for the recovery. The FDA has warned about products sold with illegal claims to cure, treat, mitigate, or prevent mpox, and says such products have not been proven safe and effective for mpox.

Some remedies, like oatmeal baths or calamine/petroleum jelly, may help symptoms such as itching or discomfort, but that is different from curing the infection. Anyone with suspected mpox should contact a healthcare provider, avoid close contact until cleared, and be especially cautious if pregnant, immunocompromised, or having severe pain, eye symptoms, or widespread lesions.

record_id Q52_Gemini

question_id Q52

model Gemini

answer_text:

The Short Answer: No, It Is Not Credible

There is currently no scientific evidence that any herbal remedy can cure mpox. While these claims frequently circulate on social media, global health authorities like the World Health Organization (WHO) and the U.S. Food and Drug Administration (FDA) warn that they are unproven, unregulated, and potentially dangerous.

In fact, regulatory bodies like the FDA have actively issued warning letters to online companies illegally marketing items like essential oils, vitamins, and colloidal silver as mpox treatments or cures.

Why Online Herbal Claims Aren't Reliable

When an online post or video claims a specific plant can "wipe out" or "cure" a virus like mpox, it usually falls flat for a few critical reasons:

Lack of Clinical Testing: For a remedy to be proven effective, it must undergo rigorous, controlled clinical trials in humans to ensure it safely treats the virus. Social media claims usually rely on anecdotal stories or early-stage laboratory tests on cells, which rarely translate into real-world cures.

Debunked "Miracle" Plants: Viral videos frequently promote specific local plants. For instance, public health experts and fact-checkers have had to debunk widespread claims that the Mimosa pudica ("shame plant") could cure mpox, noting there is absolutely no scientific basis for it.

The "Natural" Recovery Illusion: Most people with mild cases of mpox recover on their own within 2 to 4 weeks with basic rest and hydration. If someone takes a herbal remedy and gets better, they often falsely credit the herb rather than their own immune system.

The Reality of Medical Research & Treatment

It is worth noting that organizations like the Africa Centres for Disease Control and Prevention (Africa CDC) are currently researching traditional African medicines and natural compounds to see if they can be refined into future treatments. However, this research is in its infancy, using data-driven screening and highly regulated clinical trials-it is a far cry from the unverified mixtures sold online.

If you or someone you know is looking into how mpox is actually managed, here is what standard medical care looks like:

Supportive Care: For most patients, treatment focuses entirely on soothing symptoms. This includes managing pain (with medications like ibuprofen or acetaminophen), protecting the skin rash, and staying hydrated.

Antiviral Medications: In severe cases, or for people with weakened immune systems, doctors may use antiviral medications like Tecovirimat (TPOXX). However, even these pharmaceuticals are tightly regulated and are still undergoing ongoing clinical trials to fully map out their efficacy against mpox.

Prevention: The JYNNEOS vaccine remains the primary medical defense for protecting people at high risk of exposure.

The bottom line: It's completely understandable to look for accessible solutions, but relying on internet remedies for a viral infection can delay proper medical care. If you suspect an infection, skip the social media advice and consult a licensed healthcare professional.

record_id Q52_Copilot

question_id Q52

model Copilot

answer_text:

Short answer: No - claims that a specific herbal remedy "cures" mpox are not credible unless supported by clinical trials. Most positive signals for plant compounds come from computer or lab studies, not human trials, so they are hypotheses, not proven cures.

Evidence summary

Claim Evidence type Strength What it actually shows Next step

Herbal compound binds viral protein In silico molecular docking Weak to preliminary Suggests a molecule might interfere with viral proteins in a computer model. Lab tests and animal studies

Plant extract inhibits virus in a dish In vitro cell studies Moderate but limited Shows activity under controlled lab conditions; dosing and safety in people unknown. Animal studies, then clinical trials

Herbal remedy cures mpox in people Clinical trial evidence None found No published randomized trials showing cure in humans as of current reviews.

Natural products as drug leads Review and research interest Promising for drug discovery Natural compounds are a source of candidates but require full drug development.

Why online "cure" claims are unreliable

Most research is preliminary. The bulk of published work on plant compounds and mpox is in silico (computer docking) or a few in vitro studies; these do not prove safety or effectiveness in people.

No clinical trial evidence for herbal cures. Recent systematic mappings and reviews found no clinical or robust in-vivo human trials demonstrating an herbal cure for mpox.

Promising leads are not the same as treatments. Reviews note natural products can be starting points for drug development, but that process requires animal studies, toxicity testing, and human trials.

Risks of relying on unproven herbal claims

Delay of effective care. Waiting for an unproven remedy can postpone antiviral treatment or medical care when needed.

Safety and interactions. Herbal products can cause side effects or interact with other medicines; dosing and purity are often unknown.

Misinformation spread. Anecdotes and social-media posts can amplify false certainty; scientific validation requires controlled trials.

Practical guidance

Talk to a clinician if you suspect mpox or have symptoms; clinicians can advise on testing, isolation, wound care, and approved antivirals (e.g., tecovirimat) when indicated.

Treat herbal claims as hypotheses until you see peer-reviewed clinical trial results showing safety and efficacy.

If you use an herbal product, tell your healthcare provider so they can check for interactions or harms.

Bottom line: Herbal remedies may yield promising molecules in the lab, but there is currently no credible clinical evidence that any herbal product cures mpox. Consult a healthcare professional for diagnosis and treatment.

Natural Compounds against Mpox: Mapping Evidence and Identifying Gaps CiteDrive

Natural compounds of medicinal plants as a source for novel anti-orthopoxvirus medications Life Medicine Oxford

A review of herbal therapeutics for the prevention and management of poxvirus infections Archives of Microbiology Springer Nature

record_id Q52_DeepSeek

question_id Q52

model DeepSeek

answer_text:

Simply put, claims that a specific herbal remedy can outright cure mpox are not credible. While there is genuine scientific interest in the potential of natural compounds, no herbal remedy has been proven to be a safe and effective cure for mpox.

Here is a more detailed breakdown to help you understand why.

What Science Actually Says

It's true that researchers are exploring the potential of natural compounds. Some studies have shown that certain plants may have antiviral properties that could be useful against mpox. For instance, some have investigated compounds derived from turmeric, curry leaves and olives, and traditional Chinese medicine-.

These preliminary findings, however, are the very first step in a long, rigorous process that can take years. They happen in a lab ("in-vitro" or "in-silico" studies) and don't prove that drinking a tea or taking a supplement will cure a person of a disease. The scientific consensus is clear:

No Proven Cure: There is currently no scientific evidence from clinical trials to support the claim that any specific herbal remedy can cure mpox-.

Supportive Use Only: While some natural products might help support the immune system or manage symptoms, they cannot replace proven medical treatments-. Experts advise that enhancing your immune system is not the same as having a cure for a specific virus-.

The Problem of Misinformation

The gap between promising research and a proven cure is where misinformation thrives. Health authorities are actively fighting back against this for several important reasons.

Fraudulent Products: The U.S. Food and Drug Administration (FDA) has repeatedly issued warnings to companies that illegally sell online "herbal natural remedies" with false claims of curing or treating mpox-. These products are often completely unregulated and have not been reviewed for safety or effectiveness-.

Health Risks: Relying on unverified herbal cures can be dangerous because it can delay someone from seeking proper medical care, which allows the disease to get worse and increases the risk of spreading it to others-. In some places, public health officials have even warned residents against turning to traditional healers for mpox treatment for this very reason-.

What the Experts Actually Recommend

So, if there's no herbal cure, what should you do if you think you have mpox? The guidelines from the World Health Organization (WHO) and the U.S. Centers for Disease Control and Prevention (CDC) focus entirely on evidence-based care.

Official Guidelines: The WHO and CDC provide guidelines for clinical management, which emphasize supportive care (like managing pain and fever), preventing complications, and infection control to stop the spread-.

Antiviral Medication: There is currently no antiviral treatment approved by the FDA specifically for mpox-. The drug tecovirimat (TPOXX) is approved for smallpox and is available for the treatment of mpox but only under a special expanded access (or "compassionate use") protocol through the CDC-. It's not a widely available drug that the general public can access.

In Summary: How to Spot a "Cure" Claim

Online health claims, especially for a serious disease like mpox, should be treated with caution. Here's a quick checklist to help you evaluate them:

Look for the Word "Cure": If a product, especially an herbal one, claims it can "cure" mpox, that is an immediate red flag. No such cure exists.

Check the Source: Information from major health organizations (WHO, CDC, national health services) is the most reliable. Be wary of information shared on social media or from companies directly selling a product.

Be Skeptical of Anecdotes: Personal testimonials and "miracle cures" are not a substitute for scientific evidence.

Where to Seek Reliable Information

If you are concerned about mpox, the best course of action is always to consult a healthcare professional. You can also find reliable, up-to-date information from official sources like the World Health Organization (WHO) and the U.S. Centers for Disease Control and Prevention (CDC).

I hope this helps you navigate the information online safely. If you have any other questions, feel free to ask.

record_id Q52_Doubao

question_id Q52

model Doubao

answer_text:

It is not credible at all to claim that any herbal remedy can cure mpox. Here are the clear facts:

Mpox is a viral infectious disease caused by the mpox virus. No herbal remedy or folk folk prescription has been scientifically proven or recognized by global health authorities (WHO, disease control agencies) to cure mpox.

There is no specific herbal medicine that can eliminate the mpox virus. The standard medical management of mpox is symptomatic supportive care (fever reduction, pain relief, wound care, preventing secondary bacterial infection), and professional medical intervention for severe cases. Specific antiviral drugs and vaccines are the only effective medical means for prevention and clinical treatment.

Online claims about herbal cures are mostly rumors and false marketing. They are often used to sell unregulated herbal products, misleading the public.

Blindly using unproven herbal remedies will only delay formal medical treatment, easily lead to aggravated symptoms, serious complications, and some herbal medicines also have toxic side effects that may damage the liver and kidneys or cause allergic reactions.

If someone has suspected mpox symptoms, the only correct approach is to seek medical treatment at a regular hospital immediately, follow official diagnosis and treatment guidelines, and never believe online herbal cure rumors.

record_id Q52_Qwen

question_id Q52

model Qwen

answer_text:

It is not credible to claim that a specific herbal remedy can completely "cure" mpox. Such claims usually lack scientific backing and may delay proper medical treatment.

Why Online Claims Are Unreliable

Most viral claims about "miracle cures" exploit the confusion between scientific research and actual clinical treatments.

Misinterpretation of Research: There is legitimate scientific research exploring plant-derived compounds (such as curcumin or resveratrol) against the mpox virus in laboratory settings. These studies suggest that certain plant molecules might inhibit viral entry or replication in a petri dish . However, this is early-stage research. It does not mean that simply eating these herbs or taking unregulated supplements will cure an active infection in the human body.

Lack of Clinical Evidence: For a treatment to be considered a "cure," it must pass rigorous clinical trials to prove it is safe and effective in humans. Currently, there is no herbal remedy that has received regulatory approval as a definitive cure for mpox.

Risks of "Secret Remedies": Relying on unverified (folk remedies) or "internet famous medicines" often leads to a false sense of security. This can result in delaying isolation or seeing a doctor, potentially leading to complications like bacterial superinfections or the spread of the virus to close contacts .

Evidence-Based Treatment for Mpox

The medical community relies on proven methods to manage mpox:

Symptomatic Treatment: Most healthy individuals recover on their own with supportive care. This includes isolating to prevent spread, managing fever and pain, and carefully cleaning skin lesions to avoid bacterial infection .

Antiviral Therapy: For severe cases or people with high risks (such as immunocompromised individuals), doctors may prescribe specific antiviral medications. Drugs like tecovirimat have been approved for treating monkeypox/mpox and are the standard of care for serious infections .

Conclusion

While nature may hold potential for future drug development, current online claims about herbal cures are misleading. The safest course of action is to focus on symptom relief, strict hygiene, and seeking professional medical advice if symptoms worsen.
