# Supplementary material for: Evaluating search-enabled large language model interfaces for mpox public health consultation: a guideline-based comparative study
Source: Front Public Health. 2026 Aug 26;14:1913260. doi: 10.3389/fpubh.2026.1913260 (PMC13553459; doi:10.3389/fpubh.2026.1913260)
Supplement: Supplementary file 4 [file Supplementary_file_4.DOCX]

**Rater Characteristics and Additional Statistical Results**

This supplementary material summarizes the characteristics of the five raters involved in model-response evaluation and provides additional statistical results, including inter-rater agreement and pairwise model comparisons.

**Table S1. Characteristics of the five raters involved in response evaluation**

| **Rater ID** | **Professional role** | **Specialty/background** | **Years of clinical** | **Main responsibility in this study** | **Standardized training before evaluation** | **Actual interface identity disclosed to the rater** |
| --- | --- | --- | --- | --- | --- | --- |
| Rater 1 | Infectious-disease physician | Infectious diseases | 15 years | Independent response evaluation only | Yes | No |
| Rater 2 | Infectious-disease physician | Infectious diseases | 22 years | Independent response evaluation only | Yes | No |
| Rater 3 | Infectious-disease specialist nurse | Infectious-disease nursing | 13 years | Independent response evaluation only | Yes | No |
| Rater 4 | Infectious-disease specialist nurse | Infectious-disease nursing | 12 years | Independent response evaluation only | Yes | No |
| Rater 5 | Infectious-disease education nurse | Infectious-disease nursing and patient education | 19 years | Independent response evaluation only | Yes | No |

Note: Actual interface names were replaced with coded labels (Model 1–Model 6), and the code key was not provided to the raters. The coded responses were not randomized within question-level blocks, and the effectiveness of identity masking was not formally assessed; therefore, complete assessor blinding could not be guaranteed.

**Additional statistical results**

**Table S2. Inter-rater agreement**

| **Metric** | **Agreement index** | **Estimate** | **95% CI** | ***P* value** | **Complete items** | **No. of raters** | **Note** |
| --- | --- | --- | --- | --- | --- | --- | --- |
| Safety | Fleiss' kappa | 0.869 | 0.800-0.923 | <0.001 | 312 | 5 | Primary agreement index for Safety; 95% CI based on 3000 nonparametric bootstrap resamples. A negative lower CI may occur when unsafe outcomes are extremely sparse because kappa can theoretically be below 0; this does not by itself indicate a coding error. |
| Accuracy | ICC(2,1) | 0.828 | 0.800-0.853 | <0.001 | 312 | 5 | Two-way random-effects, absolute-agreement, single-measure ICC. |
| Empathy | ICC(2,1) | 0.883 | 0.864-0.901 | <0.001 | 312 | 5 | Two-way random-effects, absolute-agreement, single-measure ICC. |
| DISCERN | ICC(2,1) | 0.868 | 0.847-0.888 | <0.001 | 312 | 5 | Two-way random-effects, absolute-agreement, single-measure ICC. |
| EQIP | ICC(2,1) | 0.856 | 0.833-0.878 | <0.001 | 312 | 5 | Two-way random-effects, absolute-agreement, single-measure ICC. |
| JAMA | ICC(2,1) | 0.838 | 0.812-0.862 | <0.001 | 312 | 5 | Two-way random-effects, absolute-agreement, single-measure ICC. |
| GQS | ICC(2,1) | 0.864 | 0.842-0.884 | <0.001 | 312 | 5 | Two-way random-effects, absolute-agreement, single-measure ICC. |

Note: Safety was assessed using Fleiss' kappa. Accuracy, Empathy, DISCERN, EQIP, JAMA and GQS were assessed using ICC(2,1).

**Table S3. Pairwise comparisons of safety**

| **Comparison** | ***N* pairs** | **Safe rate difference, %** | **Safe rate difference 95% CI** | **Safe to unsafe, *n*** | **Unsafe to safe, *n*** | **Matched OR** | **Matched OR 95% CI** | **Adjusted *P*** | **Note** |
| --- | --- | --- | --- | --- | --- | --- | --- | --- | --- |
| ChatGPT vs Gemini | 52 | 1.92 | -7.69-11.54 | 4 | 3 | 1.33 | 0.30-5.96 | 1.000 | Pairwise McNemar test |
| ChatGPT vs Copilot | 52 | 3.85 | -5.77-13.46 | 4 | 2 | 2.00 | 0.37-10.92 | 1.000 | Pairwise McNemar test |
| ChatGPT vs DeepSeek | 52 | 1.92 | -3.85-9.62 | 2 | 1 | 2.00 | 0.18-22.06 | 1.000 | Pairwise McNemar test |
| ChatGPT vs Doubao | 52 | 0.00 | -9.62-9.62 | 3 | 3 | 1.00 | 0.20-4.95 | 1.000 | Pairwise McNemar test |
| ChatGPT vs Qwen | 52 | 5.77 | -3.85-15.38 | 5 | 2 | 2.50 | 0.48-12.89 | 1.000 | Pairwise McNemar test |
| Gemini vs Copilot | 52 | 1.92 | -7.69-11.54 | 4 | 3 | 1.33 | 0.30-5.96 | 1.000 | Pairwise McNemar test |
| Gemini vs DeepSeek | 52 | 0.00 | -11.54-11.54 | 4 | 4 | 1.00 | 0.25-4.00 | 1.000 | Pairwise McNemar test |
| Gemini vs Doubao | 52 | -1.92 | -13.46-9.62 | 4 | 5 | 0.80 | 0.21-2.98 | 1.000 | Pairwise McNemar test |
| Gemini vs Qwen | 52 | 3.85 | -5.77-13.46 | 5 | 3 | 1.67 | 0.40-6.97 | 1.000 | Pairwise McNemar test |
| Copilot vs DeepSeek | 52 | -1.92 | -11.54-7.69 | 3 | 4 | 0.75 | 0.17-3.35 | 1.000 | Pairwise McNemar test |
| Copilot vs Doubao | 52 | -3.85 | -15.38-5.77 | 3 | 5 | 0.60 | 0.14-2.51 | 1.000 | Pairwise McNemar test |
| Copilot vs Qwen | 52 | 1.92 | -9.62-13.46 | 5 | 4 | 1.25 | 0.34-4.66 | 1.000 | Pairwise McNemar test |
| DeepSeek vs Doubao | 52 | -1.92 | -9.66-5.77 | 2 | 3 | 0.67 | 0.11-3.99 | 1.000 | Pairwise McNemar test |
| DeepSeek vs Qwen | 52 | 3.85 | -3.85-13.46 | 4 | 2 | 2.00 | 0.37-10.92 | 1.000 | Pairwise McNemar test |
| Doubao vs Qwen | 52 | 5.77 | -3.85-15.38 | 5 | 2 | 2.50 | 0.48-12.89 | 1.000 | Pairwise McNemar test |

Note: Pairwise comparisons were performed using McNemar tests with Benjamini-Hochberg correction. Safe-rate differences and their bootstrap 95% CIs are reported.

**Table S4. Significant pairwise comparisons for non-binary outcomes**

| **Metric** | **Comparison** | ***N* pairs** | **Mean difference** | **Mean difference 95% CI** | **Median difference** | **Median difference 95% CI** | **Rank-biserial correlation** | **Rank-biserial 95% CI** | **Adjusted *P*** |
| --- | --- | --- | --- | --- | --- | --- | --- | --- | --- |
| Accuracy | ChatGPT vs Copilot | 52 | 0.91 | 0.84-0.97 | 1.00 | 1.00-1.00 | 1.000 | 1.000-1.000 | <0.001 |
| Accuracy | ChatGPT vs DeepSeek | 52 | 0.95 | 0.90-1.01 | 1.00 | 1.00-1.00 | 1.000 | 1.000-1.000 | <0.001 |
| Accuracy | ChatGPT vs Doubao | 52 | 0.93 | 0.84-1.00 | 1.00 | 1.00-1.00 | 0.998 | 0.990-1.000 | <0.001 |
| Accuracy | ChatGPT vs Qwen | 52 | 1.11 | 1.01-1.21 | 1.00 | 1.00-1.00 | 1.000 | 1.000-1.000 | <0.001 |
| Accuracy | Gemini vs Copilot | 52 | 0.87 | 0.77-0.95 | 1.00 | 1.00-1.00 | 0.995 | 0.975-1.000 | <0.001 |
| Accuracy | Gemini vs DeepSeek | 52 | 0.91 | 0.82-0.99 | 1.00 | 1.00-1.00 | 0.998 | 0.989-1.000 | <0.001 |
| Accuracy | Gemini vs Doubao | 52 | 0.88 | 0.77-0.98 | 1.00 | 1.00-1.00 | 0.991 | 0.959-1.000 | <0.001 |
| Accuracy | Gemini vs Qwen | 52 | 1.06 | 0.97-1.18 | 1.00 | 0.90-1.00 | 1.000 | 1.000-1.000 | <0.001 |
| Accuracy | Copilot vs Qwen | 52 | 0.20 | 0.09-0.32 | 0.00 | 0.00-0.20 | 0.504 | 0.160-0.770 | 0.014 |
| Accuracy | DeepSeek vs Qwen | 52 | 0.15 | 0.06-0.26 | 0.00 | 0.00-0.10 | 0.517 | 0.151-0.796 | 0.017 |
| Accuracy | Doubao vs Qwen | 52 | 0.18 | 0.07-0.30 | 0.00 | 0.00-0.20 | 0.402 | 0.056-0.682 | 0.035 |
| Empathy | ChatGPT vs Copilot | 52 | 0.95 | 0.92-0.98 | 1.00 | 1.00-1.00 | 1.000 | 1.000-1.000 | <0.001 |
| Empathy | ChatGPT vs DeepSeek | 52 | 0.98 | 0.95-1.02 | 1.00 | 1.00-1.00 | 1.000 | 1.000-1.000 | <0.001 |
| Empathy | ChatGPT vs Doubao | 52 | 0.99 | 0.95-1.02 | 1.00 | 1.00-1.00 | 1.000 | 1.000-1.000 | <0.001 |
| Empathy | ChatGPT vs Qwen | 52 | 1.42 | 1.30-1.55 | 1.20 | 1.00-1.80 | 1.000 | 1.000-1.000 | <0.001 |
| Empathy | Gemini vs Copilot | 52 | 0.95 | 0.92-0.97 | 1.00 | 1.00-1.00 | 1.000 | 1.000-1.000 | <0.001 |
| Empathy | Gemini vs DeepSeek | 52 | 0.98 | 0.95-1.02 | 1.00 | 1.00-1.00 | 1.000 | 1.000-1.000 | <0.001 |
| Empathy | Gemini vs Doubao | 52 | 0.99 | 0.95-1.03 | 1.00 | 1.00-1.00 | 1.000 | 1.000-1.000 | <0.001 |
| Empathy | Gemini vs Qwen | 52 | 1.42 | 1.30-1.55 | 1.20 | 1.00-1.80 | 1.000 | 1.000-1.000 | <0.001 |
| Empathy | Copilot vs Qwen | 52 | 0.47 | 0.34-0.60 | 0.20 | 0.10-1.00 | 0.962 | 0.879-1.000 | <0.001 |
| Empathy | DeepSeek vs Qwen | 52 | 0.44 | 0.31-0.57 | 0.20 | 0.00-0.80 | 0.916 | 0.772-0.989 | <0.001 |
| Empathy | Doubao vs Qwen | 52 | 0.43 | 0.30-0.57 | 0.20 | 0.00-0.80 | 0.880 | 0.719-0.977 | <0.001 |
| DISCERN | ChatGPT vs Gemini | 52 | 6.24 | 5.46-7.03 | 5.80 | 5.00-6.80 | 0.997 | 0.983-1.000 | <0.001 |
| DISCERN | ChatGPT vs Copilot | 52 | 12.68 | 11.86-13.52 | 12.50 | 11.50-13.60 | 1.000 | 1.000-1.000 | <0.001 |
| DISCERN | ChatGPT vs DeepSeek | 52 | 15.30 | 14.65-15.95 | 15.00 | 14.40-15.60 | 1.000 | 1.000-1.000 | <0.001 |
| DISCERN | ChatGPT vs Doubao | 52 | 18.35 | 17.57-19.05 | 18.60 | 17.70-19.20 | 1.000 | 1.000-1.000 | <0.001 |
| DISCERN | ChatGPT vs Qwen | 52 | 22.14 | 21.44-22.83 | 22.20 | 21.60-22.80 | 1.000 | 1.000-1.000 | <0.001 |
| DISCERN | Gemini vs Copilot | 52 | 6.44 | 5.74-7.12 | 6.30 | 5.60-7.10 | 0.997 | 0.983-1.000 | <0.001 |
| DISCERN | Gemini vs DeepSeek | 52 | 9.06 | 8.32-9.82 | 8.80 | 8.30-9.40 | 1.000 | 1.000-1.000 | <0.001 |
| DISCERN | Gemini vs Doubao | 52 | 12.10 | 11.35-12.84 | 12.30 | 11.30-13.20 | 1.000 | 1.000-1.000 | <0.001 |
| DISCERN | Gemini vs Qwen | 52 | 15.90 | 15.14-16.70 | 16.00 | 14.80-17.20 | 1.000 | 1.000-1.000 | <0.001 |
| DISCERN | Copilot vs DeepSeek | 52 | 2.62 | 1.89-3.33 | 2.60 | 2.20-3.60 | 0.835 | 0.657-0.960 | <0.001 |
| DISCERN | Copilot vs Doubao | 52 | 5.66 | 4.88-6.43 | 5.70 | 5.10-6.30 | 0.991 | 0.964-1.000 | <0.001 |
| DISCERN | Copilot vs Qwen | 52 | 9.46 | 8.69-10.25 | 9.40 | 8.50-10.20 | 1.000 | 1.000-1.000 | <0.001 |
| DISCERN | DeepSeek vs Doubao | 52 | 3.04 | 2.30-3.76 | 3.10 | 2.50-4.00 | 0.858 | 0.692-0.980 | <0.001 |
| DISCERN | DeepSeek vs Qwen | 52 | 6.84 | 6.14-7.52 | 7.10 | 6.10-7.80 | 0.999 | 0.991-1.000 | <0.001 |
| DISCERN | Doubao vs Qwen | 52 | 3.80 | 3.15-4.43 | 3.60 | 2.90-4.30 | 0.978 | 0.927-1.000 | <0.001 |
| EQIP | ChatGPT vs Gemini | 52 | 5.90 | 4.69-7.17 | 6.50 | 5.00-7.00 | 0.900 | 0.750-1.000 | <0.001 |
| EQIP | ChatGPT vs Copilot | 52 | 16.25 | 15.08-17.40 | 16.00 | 15.00-17.00 | 1.000 | 1.000-1.000 | <0.001 |
| EQIP | ChatGPT vs DeepSeek | 52 | 20.94 | 19.94-21.83 | 21.00 | 20.00-22.00 | 1.000 | 1.000-1.000 | <0.001 |
| EQIP | ChatGPT vs Doubao | 52 | 23.62 | 22.54-24.62 | 23.50 | 23.00-24.00 | 1.000 | 1.000-1.000 | <0.001 |
| EQIP | ChatGPT vs Qwen | 52 | 29.67 | 28.52-30.81 | 29.50 | 28.50-31.00 | 1.000 | 1.000-1.000 | <0.001 |
| EQIP | Gemini vs Copilot | 52 | 10.35 | 9.19-11.48 | 10.50 | 10.00-11.00 | 0.991 | 0.970-1.000 | <0.001 |
| EQIP | Gemini vs DeepSeek | 52 | 15.04 | 13.79-16.23 | 15.00 | 14.00-16.50 | 1.000 | 1.000-1.000 | <0.001 |
| EQIP | Gemini vs Doubao | 52 | 17.71 | 16.48-18.96 | 18.00 | 17.00-19.00 | 1.000 | 1.000-1.000 | <0.001 |
| EQIP | Gemini vs Qwen | 52 | 23.77 | 22.65-24.85 | 24.00 | 22.00-25.00 | 1.000 | 1.000-1.000 | <0.001 |
| EQIP | Copilot vs DeepSeek | 52 | 4.69 | 3.58-5.81 | 4.00 | 3.50-6.00 | 0.903 | 0.777-0.983 | <0.001 |
| EQIP | Copilot vs Doubao | 52 | 7.37 | 6.29-8.38 | 8.00 | 7.00-9.00 | 0.988 | 0.953-1.000 | <0.001 |
| EQIP | Copilot vs Qwen | 52 | 13.42 | 12.13-14.73 | 13.00 | 12.00-15.00 | 1.000 | 1.000-1.000 | <0.001 |
| EQIP | DeepSeek vs Doubao | 52 | 2.67 | 1.60-3.79 | 3.00 | 2.00-3.00 | 0.685 | 0.446-0.881 | <0.001 |
| EQIP | DeepSeek vs Qwen | 52 | 8.73 | 7.75-9.75 | 8.00 | 7.00-9.00 | 1.000 | 1.000-1.000 | <0.001 |
| EQIP | Doubao vs Qwen | 52 | 6.06 | 4.88-7.25 | 6.00 | 4.00-7.00 | 0.959 | 0.876-1.000 | <0.001 |
| JAMA | ChatGPT vs Copilot | 52 | 0.39 | 0.25-0.54 | 0.00 | 0.00-1.00 | 0.816 | 0.585-0.951 | <0.001 |
| JAMA | ChatGPT vs DeepSeek | 52 | 0.97 | 0.91-1.02 | 1.00 | 1.00-1.00 | 1.000 | 1.000-1.000 | <0.001 |
| JAMA | ChatGPT vs Doubao | 52 | 1.01 | 0.98-1.05 | 1.00 | 1.00-1.00 | 1.000 | 1.000-1.000 | <0.001 |
| JAMA | ChatGPT vs Qwen | 52 | 0.99 | 0.95-1.03 | 1.00 | 1.00-1.00 | 1.000 | 1.000-1.000 | <0.001 |
| JAMA | Gemini vs Copilot | 52 | 0.40 | 0.26-0.54 | 0.00 | 0.00-0.80 | 0.875 | 0.665-0.979 | <0.001 |
| JAMA | Gemini vs DeepSeek | 52 | 0.98 | 0.92-1.04 | 1.00 | 1.00-1.00 | 1.000 | 1.000-1.000 | <0.001 |
| JAMA | Gemini vs Doubao | 52 | 1.02 | 0.98-1.06 | 1.00 | 1.00-1.00 | 1.000 | 1.000-1.000 | <0.001 |
| JAMA | Gemini vs Qwen | 52 | 1.00 | 0.96-1.04 | 1.00 | 1.00-1.00 | 1.000 | 1.000-1.000 | <0.001 |
| JAMA | Copilot vs DeepSeek | 52 | 0.58 | 0.44-0.72 | 0.80 | 0.20-1.00 | 0.951 | 0.860-0.994 | <0.001 |
| JAMA | Copilot vs Doubao | 52 | 0.62 | 0.48-0.75 | 0.90 | 0.20-1.00 | 0.992 | 0.962-1.000 | <0.001 |
| JAMA | Copilot vs Qwen | 52 | 0.60 | 0.45-0.75 | 1.00 | 0.20-1.00 | 0.929 | 0.818-0.990 | <0.001 |
| GQS | ChatGPT vs Gemini | 52 | 0.12 | 0.04-0.22 | 0.00 | 0.00-0.00 | 0.672 | 0.284-0.934 | 0.012 |
| GQS | ChatGPT vs Copilot | 52 | 0.98 | 0.95-1.02 | 1.00 | 1.00-1.00 | 1.000 | 1.000-1.000 | <0.001 |
| GQS | ChatGPT vs DeepSeek | 52 | 1.03 | 0.96-1.12 | 1.00 | 1.00-1.00 | 1.000 | 1.000-1.000 | <0.001 |
| GQS | ChatGPT vs Doubao | 52 | 0.99 | 0.96-1.02 | 1.00 | 1.00-1.00 | 1.000 | 1.000-1.000 | <0.001 |
| GQS | ChatGPT vs Qwen | 52 | 1.21 | 1.10-1.33 | 1.00 | 1.00-1.00 | 1.000 | 1.000-1.000 | <0.001 |
| GQS | Gemini vs Copilot | 52 | 0.86 | 0.76-0.95 | 1.00 | 1.00-1.00 | 0.991 | 0.959-1.000 | <0.001 |
| GQS | Gemini vs DeepSeek | 52 | 0.91 | 0.80-1.00 | 1.00 | 1.00-1.00 | 0.988 | 0.953-1.000 | <0.001 |
| GQS | Gemini vs Doubao | 52 | 0.87 | 0.77-0.95 | 1.00 | 1.00-1.00 | 0.995 | 0.975-1.000 | <0.001 |
| GQS | Gemini vs Qwen | 52 | 1.09 | 0.98-1.20 | 1.00 | 1.00-1.00 | 0.999 | 0.991-1.000 | <0.001 |
| GQS | Copilot vs Qwen | 52 | 0.23 | 0.12-0.35 | 0.00 | 0.00-0.20 | 0.724 | 0.435-0.920 | 0.001 |
| GQS | DeepSeek vs Qwen | 52 | 0.18 | 0.07-0.30 | 0.00 | 0.00-0.10 | 0.610 | 0.258-0.872 | 0.008 |
| GQS | Doubao vs Qwen | 52 | 0.22 | 0.11-0.35 | 0.00 | 0.00-0.00 | 0.723 | 0.372-0.935 | 0.004 |
| ARI | ChatGPT vs Doubao | 52 | -8.69 | -12.78--4.89 | -2.84 | -6.98--0.98 | -0.566 | -0.781--0.296 | 0.002 |
| ARI | Gemini vs Doubao | 52 | -8.41 | -12.29--5.02 | -2.93 | -6.77-0.42 | -0.563 | -0.771--0.299 | 0.002 |
| ARI | Copilot vs Doubao | 52 | -7.93 | -12.16--4.29 | -2.40 | -4.75--0.04 | -0.530 | -0.746--0.254 | 0.003 |
| ARI | DeepSeek vs Doubao | 52 | -7.51 | -11.28--4.10 | -3.42 | -7.65--0.83 | -0.604 | -0.820--0.351 | 0.002 |
| ARI | Doubao vs Qwen | 52 | 8.70 | 5.38-12.60 | 2.54 | 0.77-5.83 | 0.681 | 0.454-0.856 | <0.001 |
| CL | ChatGPT vs Gemini | 52 | -1.03 | -1.60--0.48 | -0.78 | -1.13--0.46 | -0.549 | -0.782--0.279 | <0.001 |
| CL | ChatGPT vs Copilot | 52 | -2.19 | -2.66--1.71 | -2.41 | -2.60--2.00 | -0.884 | -0.996--0.701 | <0.001 |
| CL | ChatGPT vs Doubao | 52 | -2.37 | -3.00--1.73 | -1.78 | -2.63--1.34 | -0.875 | -0.978--0.724 | <0.001 |
| CL | ChatGPT vs Qwen | 52 | -2.55 | -3.14--1.94 | -2.14 | -3.11--1.87 | -0.878 | -0.986--0.726 | <0.001 |
| CL | Gemini vs Copilot | 52 | -1.16 | -1.54--0.80 | -1.20 | -1.80--0.81 | -0.759 | -0.920--0.541 | <0.001 |
| CL | Gemini vs DeepSeek | 52 | 0.79 | 0.23-1.37 | 0.61 | -0.25-1.39 | 0.373 | 0.071-0.636 | 0.027 |
| CL | Gemini vs Doubao | 52 | -1.34 | -2.01--0.69 | -1.36 | -1.91--0.76 | -0.584 | -0.807--0.325 | <0.001 |
| CL | Gemini vs Qwen | 52 | -1.52 | -2.14--0.89 | -2.09 | -2.58--1.31 | -0.628 | -0.843--0.367 | <0.001 |
| CL | Copilot vs DeepSeek | 52 | 1.96 | 1.54-2.38 | 1.83 | 1.43-2.62 | 0.924 | 0.820-0.984 | <0.001 |
| CL | DeepSeek vs Doubao | 52 | -2.14 | -2.69--1.56 | -2.41 | -3.15--1.22 | -0.842 | -0.954--0.675 | <0.001 |
| CL | DeepSeek vs Qwen | 52 | -2.31 | -2.92--1.69 | -2.59 | -3.03--1.76 | -0.835 | -0.968--0.647 | <0.001 |
| FKGL | ChatGPT vs Gemini | 52 | -0.68 | -1.56-0.35 | -0.91 | -1.89--0.10 | -0.416 | -0.681--0.123 | 0.019 |
| FKGL | ChatGPT vs Copilot | 52 | -0.95 | -1.73--0.06 | -1.48 | -1.98--0.41 | -0.485 | -0.732--0.203 | 0.006 |
| FKGL | ChatGPT vs DeepSeek | 52 | -1.22 | -2.32--0.09 | -1.49 | -3.00-0.32 | -0.354 | -0.625--0.057 | 0.044 |
| FKGL | ChatGPT vs Doubao | 52 | -7.01 | -10.25--3.94 | -2.60 | -5.73--0.50 | -0.602 | -0.804--0.347 | 0.003 |
| FKGL | ChatGPT vs Qwen | 52 | -0.69 | -1.53-0.24 | -0.80 | -1.43-0.19 | -0.361 | -0.636--0.045 | 0.044 |
| FKGL | Gemini vs Doubao | 52 | -6.33 | -9.55--3.40 | -2.32 | -5.24-0.92 | -0.527 | -0.748--0.244 | 0.005 |
| FKGL | Copilot vs Doubao | 52 | -6.07 | -9.36--3.17 | -1.65 | -3.52--0.25 | -0.492 | -0.724--0.206 | 0.006 |
| FKGL | DeepSeek vs Doubao | 52 | -5.79 | -8.89--3.07 | -2.75 | -5.70--0.61 | -0.568 | -0.782--0.308 | 0.003 |
| FKGL | Doubao vs Qwen | 52 | 6.32 | 3.51-9.43 | 0.93 | 0.02-4.03 | 0.512 | 0.232-0.739 | 0.005 |
| GFI | ChatGPT vs Gemini | 52 | -0.69 | -1.52-0.34 | -1.02 | -1.47--0.45 | -0.418 | -0.689--0.125 | 0.016 |
| GFI | ChatGPT vs Copilot | 52 | -1.23 | -2.03--0.36 | -1.50 | -2.24--0.65 | -0.512 | -0.750--0.237 | 0.006 |
| GFI | ChatGPT vs DeepSeek | 52 | -1.37 | -2.52--0.20 | -1.29 | -3.22-0.41 | -0.348 | -0.620--0.042 | 0.049 |
| GFI | ChatGPT vs Doubao | 52 | -7.31 | -11.05--4.00 | -2.88 | -5.69--0.24 | -0.589 | -0.795--0.334 | 0.003 |
| GFI | ChatGPT vs Qwen | 52 | -0.95 | -1.75--0.01 | -1.07 | -2.10--0.14 | -0.464 | -0.708--0.181 | 0.011 |
| GFI | Gemini vs Doubao | 52 | -6.63 | -9.87--3.65 | -2.66 | -6.02-1.18 | -0.508 | -0.723--0.229 | 0.006 |
| GFI | Copilot vs Doubao | 52 | -6.09 | -9.37--2.95 | -1.67 | -4.19--0.19 | -0.450 | -0.698--0.171 | 0.011 |
| GFI | DeepSeek vs Doubao | 52 | -5.95 | -9.05--3.20 | -2.64 | -5.74--0.82 | -0.557 | -0.775--0.306 | 0.004 |
| GFI | Doubao vs Qwen | 52 | 6.37 | 3.37-9.56 | 0.82 | -0.67-4.86 | 0.446 | 0.145-0.686 | 0.011 |
| SMOG | ChatGPT vs Gemini | 52 | -0.69 | -1.21--0.09 | -0.71 | -1.33--0.36 | -0.514 | -0.772--0.229 | 0.004 |
| SMOG | ChatGPT vs Copilot | 52 | -1.16 | -1.67--0.61 | -1.19 | -1.72--0.81 | -0.643 | -0.852--0.386 | <0.001 |
| SMOG | ChatGPT vs DeepSeek | 52 | -0.92 | -1.61--0.24 | -0.60 | -2.02-0.28 | -0.358 | -0.621--0.043 | 0.041 |
| SMOG | ChatGPT vs Doubao | 52 | -4.22 | -6.06--2.47 | -1.82 | -3.82--0.34 | -0.618 | -0.816--0.371 | <0.001 |
| SMOG | ChatGPT vs Qwen | 52 | -0.84 | -1.35--0.30 | -0.79 | -1.53--0.40 | -0.547 | -0.779--0.271 | 0.002 |
| SMOG | Gemini vs Doubao | 52 | -3.53 | -5.37--1.82 | -1.89 | -3.72-0.55 | -0.491 | -0.723--0.192 | 0.005 |
| SMOG | Copilot vs Doubao | 52 | -3.06 | -4.93--1.40 | -0.97 | -2.42--0.00 | -0.406 | -0.663--0.102 | 0.020 |
| SMOG | DeepSeek vs Doubao | 52 | -3.30 | -4.94--1.80 | -1.88 | -3.95--0.50 | -0.559 | -0.778--0.295 | 0.002 |
| SMOG | Doubao vs Qwen | 52 | 3.38 | 1.86-5.06 | 0.63 | -0.64-3.01 | 0.435 | 0.136-0.684 | 0.014 |
| FRES | ChatGPT vs Gemini | 52 | 6.98 | 3.37-10.74 | 6.83 | 3.94-9.02 | 0.640 | 0.385-0.855 | <0.001 |
| FRES | ChatGPT vs Copilot | 52 | 10.63 | 7.80-13.38 | 10.76 | 8.23-13.39 | 0.845 | 0.646-0.987 | <0.001 |
| FRES | ChatGPT vs DeepSeek | 52 | 5.07 | 1.11-8.84 | 6.38 | -1.53-10.81 | 0.380 | 0.087-0.637 | 0.024 |
| FRES | ChatGPT vs Doubao | 52 | 25.54 | 16.13-35.48 | 14.84 | 6.00-24.80 | 0.750 | 0.543-0.907 | <0.001 |
| FRES | ChatGPT vs Qwen | 52 | 13.10 | 9.76-16.45 | 12.18 | 8.51-17.24 | 0.851 | 0.685-0.970 | <0.001 |
| FRES | Gemini vs Copilot | 52 | 3.65 | 0.96-6.27 | 4.54 | 1.82-7.25 | 0.419 | 0.128-0.681 | 0.013 |
| FRES | Gemini vs Doubao | 52 | 18.55 | 9.94-28.16 | 8.92 | 0.37-20.43 | 0.511 | 0.236-0.732 | 0.003 |
| FRES | Gemini vs Qwen | 52 | 6.11 | 2.09-9.76 | 7.04 | 3.98-9.80 | 0.472 | 0.192-0.739 | 0.005 |
| FRES | Copilot vs DeepSeek | 52 | -5.56 | -8.18--2.77 | -6.95 | -9.54--4.68 | -0.579 | -0.804--0.308 | <0.001 |
| FRES | Copilot vs Doubao | 52 | 14.90 | 6.05-24.57 | 2.39 | 0.01-11.70 | 0.369 | 0.064-0.630 | 0.026 |
| FRES | DeepSeek vs Doubao | 52 | 20.47 | 12.21-29.54 | 13.97 | 5.25-21.20 | 0.684 | 0.462-0.868 | <0.001 |
| FRES | DeepSeek vs Qwen | 52 | 8.03 | 4.50-11.42 | 8.01 | 2.64-12.37 | 0.630 | 0.386-0.832 | <0.001 |

Note: Pairwise comparisons were performed using paired Wilcoxon signed-rank tests with Benjamini-Hochberg correction. Paired mean/median differences and rank-biserial correlations are reported with bootstrap 95% CIs. Only significant comparisons are shown.

**Table S5. Significant pairwise comparisons of safety**

**Note**

No significant pairwise safety difference after Benjamini-Hochberg correction.

Note: Pairwise comparisons were performed using McNemar tests with Benjamini-Hochberg correction. Safe-rate differences and their bootstrap 95% CIs are reported. Only significant comparisons are shown.

**Table S6. All pairwise comparisons for non-binary outcomes**

| **Metric** | **Comparison** | ***N* pairs** | **Mean difference** | **Mean difference 95% CI** | **Median difference** | **Median difference 95% CI** | **Rank-biserial correlation** | **Rank-biserial 95% CI** | **Adjusted *P*** | **Note** |
| --- | --- | --- | --- | --- | --- | --- | --- | --- | --- | --- |
| Accuracy | ChatGPT vs Gemini | 52 | 0.05 | -0.05-0.14 | 0.00 | 0.00-0.00 | 0.261 | -0.258-0.758 | 0.428 | Paired Wilcoxon signed-rank test |
| Accuracy | ChatGPT vs Copilot | 52 | 0.91 | 0.84-0.97 | 1.00 | 1.00-1.00 | 1.000 | 1.000-1.000 | <0.001 | Paired Wilcoxon signed-rank test |
| Accuracy | ChatGPT vs DeepSeek | 52 | 0.95 | 0.90-1.01 | 1.00 | 1.00-1.00 | 1.000 | 1.000-1.000 | <0.001 | Paired Wilcoxon signed-rank test |
| Accuracy | ChatGPT vs Doubao | 52 | 0.93 | 0.84-1.00 | 1.00 | 1.00-1.00 | 0.998 | 0.990-1.000 | <0.001 | Paired Wilcoxon signed-rank test |
| Accuracy | ChatGPT vs Qwen | 52 | 1.11 | 1.01-1.21 | 1.00 | 1.00-1.00 | 1.000 | 1.000-1.000 | <0.001 | Paired Wilcoxon signed-rank test |
| Accuracy | Gemini vs Copilot | 52 | 0.87 | 0.77-0.95 | 1.00 | 1.00-1.00 | 0.995 | 0.975-1.000 | <0.001 | Paired Wilcoxon signed-rank test |
| Accuracy | Gemini vs DeepSeek | 52 | 0.91 | 0.82-0.99 | 1.00 | 1.00-1.00 | 0.998 | 0.989-1.000 | <0.001 | Paired Wilcoxon signed-rank test |
| Accuracy | Gemini vs Doubao | 52 | 0.88 | 0.77-0.98 | 1.00 | 1.00-1.00 | 0.991 | 0.959-1.000 | <0.001 | Paired Wilcoxon signed-rank test |
| Accuracy | Gemini vs Qwen | 52 | 1.06 | 0.97-1.18 | 1.00 | 0.90-1.00 | 1.000 | 1.000-1.000 | <0.001 | Paired Wilcoxon signed-rank test |
| Accuracy | Copilot vs DeepSeek | 52 | 0.04 | -0.02-0.12 | 0.00 | 0.00-0.00 | 0.170 | -0.273-0.569 | 0.528 | Paired Wilcoxon signed-rank test |
| Accuracy | Copilot vs Doubao | 52 | 0.02 | -0.04-0.08 | 0.00 | 0.00-0.00 | 0.032 | -0.338-0.405 | 0.873 | Paired Wilcoxon signed-rank test |
| Accuracy | Copilot vs Qwen | 52 | 0.20 | 0.09-0.32 | 0.00 | 0.00-0.20 | 0.504 | 0.160-0.770 | 0.014 | Paired Wilcoxon signed-rank test |
| Accuracy | DeepSeek vs Doubao | 52 | -0.03 | -0.11-0.05 | 0.00 | 0.00-0.00 | -0.097 | -0.540-0.328 | 0.707 | Paired Wilcoxon signed-rank test |
| Accuracy | DeepSeek vs Qwen | 52 | 0.15 | 0.06-0.26 | 0.00 | 0.00-0.10 | 0.517 | 0.151-0.796 | 0.017 | Paired Wilcoxon signed-rank test |
| Accuracy | Doubao vs Qwen | 52 | 0.18 | 0.07-0.30 | 0.00 | 0.00-0.20 | 0.402 | 0.056-0.682 | 0.035 | Paired Wilcoxon signed-rank test |
| Empathy | ChatGPT vs Gemini | 52 | 0.00 | -0.02-0.02 | 0.00 | 0.00-0.00 | 0.000 | -0.667-0.667 | 1.000 | Paired Wilcoxon signed-rank test |
| Empathy | ChatGPT vs Copilot | 52 | 0.95 | 0.92-0.98 | 1.00 | 1.00-1.00 | 1.000 | 1.000-1.000 | <0.001 | Paired Wilcoxon signed-rank test |
| Empathy | ChatGPT vs DeepSeek | 52 | 0.98 | 0.95-1.02 | 1.00 | 1.00-1.00 | 1.000 | 1.000-1.000 | <0.001 | Paired Wilcoxon signed-rank test |
| Empathy | ChatGPT vs Doubao | 52 | 0.99 | 0.95-1.02 | 1.00 | 1.00-1.00 | 1.000 | 1.000-1.000 | <0.001 | Paired Wilcoxon signed-rank test |
| Empathy | ChatGPT vs Qwen | 52 | 1.42 | 1.30-1.55 | 1.20 | 1.00-1.80 | 1.000 | 1.000-1.000 | <0.001 | Paired Wilcoxon signed-rank test |
| Empathy | Gemini vs Copilot | 52 | 0.95 | 0.92-0.97 | 1.00 | 1.00-1.00 | 1.000 | 1.000-1.000 | <0.001 | Paired Wilcoxon signed-rank test |
| Empathy | Gemini vs DeepSeek | 52 | 0.98 | 0.95-1.02 | 1.00 | 1.00-1.00 | 1.000 | 1.000-1.000 | <0.001 | Paired Wilcoxon signed-rank test |
| Empathy | Gemini vs Doubao | 52 | 0.99 | 0.95-1.03 | 1.00 | 1.00-1.00 | 1.000 | 1.000-1.000 | <0.001 | Paired Wilcoxon signed-rank test |
| Empathy | Gemini vs Qwen | 52 | 1.42 | 1.30-1.55 | 1.20 | 1.00-1.80 | 1.000 | 1.000-1.000 | <0.001 | Paired Wilcoxon signed-rank test |
| Empathy | Copilot vs DeepSeek | 52 | 0.03 | -0.00-0.07 | 0.00 | 0.00-0.00 | 0.439 | 0.000-0.821 | 0.099 | Paired Wilcoxon signed-rank test |
| Empathy | Copilot vs Doubao | 52 | 0.04 | -0.00-0.08 | 0.00 | 0.00-0.00 | 0.363 | -0.036-0.706 | 0.099 | Paired Wilcoxon signed-rank test |
| Empathy | Copilot vs Qwen | 52 | 0.47 | 0.34-0.60 | 0.20 | 0.10-1.00 | 0.962 | 0.879-1.000 | <0.001 | Paired Wilcoxon signed-rank test |
| Empathy | DeepSeek vs Doubao | 52 | 0.00 | -0.04-0.05 | 0.00 | 0.00-0.00 | 0.003 | -0.409-0.413 | 1.000 | Paired Wilcoxon signed-rank test |
| Empathy | DeepSeek vs Qwen | 52 | 0.44 | 0.31-0.57 | 0.20 | 0.00-0.80 | 0.916 | 0.772-0.989 | <0.001 | Paired Wilcoxon signed-rank test |
| Empathy | Doubao vs Qwen | 52 | 0.43 | 0.30-0.57 | 0.20 | 0.00-0.80 | 0.880 | 0.719-0.977 | <0.001 | Paired Wilcoxon signed-rank test |
| DISCERN | ChatGPT vs Gemini | 52 | 6.24 | 5.46-7.03 | 5.80 | 5.00-6.80 | 0.997 | 0.983-1.000 | <0.001 | Paired Wilcoxon signed-rank test |
| DISCERN | ChatGPT vs Copilot | 52 | 12.68 | 11.86-13.52 | 12.50 | 11.50-13.60 | 1.000 | 1.000-1.000 | <0.001 | Paired Wilcoxon signed-rank test |
| DISCERN | ChatGPT vs DeepSeek | 52 | 15.30 | 14.65-15.95 | 15.00 | 14.40-15.60 | 1.000 | 1.000-1.000 | <0.001 | Paired Wilcoxon signed-rank test |
| DISCERN | ChatGPT vs Doubao | 52 | 18.35 | 17.57-19.05 | 18.60 | 17.70-19.20 | 1.000 | 1.000-1.000 | <0.001 | Paired Wilcoxon signed-rank test |
| DISCERN | ChatGPT vs Qwen | 52 | 22.14 | 21.44-22.83 | 22.20 | 21.60-22.80 | 1.000 | 1.000-1.000 | <0.001 | Paired Wilcoxon signed-rank test |
| DISCERN | Gemini vs Copilot | 52 | 6.44 | 5.74-7.12 | 6.30 | 5.60-7.10 | 0.997 | 0.983-1.000 | <0.001 | Paired Wilcoxon signed-rank test |
| DISCERN | Gemini vs DeepSeek | 52 | 9.06 | 8.32-9.82 | 8.80 | 8.30-9.40 | 1.000 | 1.000-1.000 | <0.001 | Paired Wilcoxon signed-rank test |
| DISCERN | Gemini vs Doubao | 52 | 12.10 | 11.35-12.84 | 12.30 | 11.30-13.20 | 1.000 | 1.000-1.000 | <0.001 | Paired Wilcoxon signed-rank test |
| DISCERN | Gemini vs Qwen | 52 | 15.90 | 15.14-16.70 | 16.00 | 14.80-17.20 | 1.000 | 1.000-1.000 | <0.001 | Paired Wilcoxon signed-rank test |
| DISCERN | Copilot vs DeepSeek | 52 | 2.62 | 1.89-3.33 | 2.60 | 2.20-3.60 | 0.835 | 0.657-0.960 | <0.001 | Paired Wilcoxon signed-rank test |
| DISCERN | Copilot vs Doubao | 52 | 5.66 | 4.88-6.43 | 5.70 | 5.10-6.30 | 0.991 | 0.964-1.000 | <0.001 | Paired Wilcoxon signed-rank test |
| DISCERN | Copilot vs Qwen | 52 | 9.46 | 8.69-10.25 | 9.40 | 8.50-10.20 | 1.000 | 1.000-1.000 | <0.001 | Paired Wilcoxon signed-rank test |
| DISCERN | DeepSeek vs Doubao | 52 | 3.04 | 2.30-3.76 | 3.10 | 2.50-4.00 | 0.858 | 0.692-0.980 | <0.001 | Paired Wilcoxon signed-rank test |
| DISCERN | DeepSeek vs Qwen | 52 | 6.84 | 6.14-7.52 | 7.10 | 6.10-7.80 | 0.999 | 0.991-1.000 | <0.001 | Paired Wilcoxon signed-rank test |
| DISCERN | Doubao vs Qwen | 52 | 3.80 | 3.15-4.43 | 3.60 | 2.90-4.30 | 0.978 | 0.927-1.000 | <0.001 | Paired Wilcoxon signed-rank test |
| EQIP | ChatGPT vs Gemini | 52 | 5.90 | 4.69-7.17 | 6.50 | 5.00-7.00 | 0.900 | 0.750-1.000 | <0.001 | Paired Wilcoxon signed-rank test |
| EQIP | ChatGPT vs Copilot | 52 | 16.25 | 15.08-17.40 | 16.00 | 15.00-17.00 | 1.000 | 1.000-1.000 | <0.001 | Paired Wilcoxon signed-rank test |
| EQIP | ChatGPT vs DeepSeek | 52 | 20.94 | 19.94-21.83 | 21.00 | 20.00-22.00 | 1.000 | 1.000-1.000 | <0.001 | Paired Wilcoxon signed-rank test |
| EQIP | ChatGPT vs Doubao | 52 | 23.62 | 22.54-24.62 | 23.50 | 23.00-24.00 | 1.000 | 1.000-1.000 | <0.001 | Paired Wilcoxon signed-rank test |
| EQIP | ChatGPT vs Qwen | 52 | 29.67 | 28.52-30.81 | 29.50 | 28.50-31.00 | 1.000 | 1.000-1.000 | <0.001 | Paired Wilcoxon signed-rank test |
| EQIP | Gemini vs Copilot | 52 | 10.35 | 9.19-11.48 | 10.50 | 10.00-11.00 | 0.991 | 0.970-1.000 | <0.001 | Paired Wilcoxon signed-rank test |
| EQIP | Gemini vs DeepSeek | 52 | 15.04 | 13.79-16.23 | 15.00 | 14.00-16.50 | 1.000 | 1.000-1.000 | <0.001 | Paired Wilcoxon signed-rank test |
| EQIP | Gemini vs Doubao | 52 | 17.71 | 16.48-18.96 | 18.00 | 17.00-19.00 | 1.000 | 1.000-1.000 | <0.001 | Paired Wilcoxon signed-rank test |
| EQIP | Gemini vs Qwen | 52 | 23.77 | 22.65-24.85 | 24.00 | 22.00-25.00 | 1.000 | 1.000-1.000 | <0.001 | Paired Wilcoxon signed-rank test |
| EQIP | Copilot vs DeepSeek | 52 | 4.69 | 3.58-5.81 | 4.00 | 3.50-6.00 | 0.903 | 0.777-0.983 | <0.001 | Paired Wilcoxon signed-rank test |
| EQIP | Copilot vs Doubao | 52 | 7.37 | 6.29-8.38 | 8.00 | 7.00-9.00 | 0.988 | 0.953-1.000 | <0.001 | Paired Wilcoxon signed-rank test |
| EQIP | Copilot vs Qwen | 52 | 13.42 | 12.13-14.73 | 13.00 | 12.00-15.00 | 1.000 | 1.000-1.000 | <0.001 | Paired Wilcoxon signed-rank test |
| EQIP | DeepSeek vs Doubao | 52 | 2.67 | 1.60-3.79 | 3.00 | 2.00-3.00 | 0.685 | 0.446-0.881 | <0.001 | Paired Wilcoxon signed-rank test |
| EQIP | DeepSeek vs Qwen | 52 | 8.73 | 7.75-9.75 | 8.00 | 7.00-9.00 | 1.000 | 1.000-1.000 | <0.001 | Paired Wilcoxon signed-rank test |
| EQIP | Doubao vs Qwen | 52 | 6.06 | 4.88-7.25 | 6.00 | 4.00-7.00 | 0.959 | 0.876-1.000 | <0.001 | Paired Wilcoxon signed-rank test |
| JAMA | ChatGPT vs Gemini | 52 | -0.01 | -0.04-0.02 | 0.00 | 0.00-0.00 | -0.099 | -0.584-0.409 | 0.770 | Paired Wilcoxon signed-rank test |
| JAMA | ChatGPT vs Copilot | 52 | 0.39 | 0.25-0.54 | 0.00 | 0.00-1.00 | 0.816 | 0.585-0.951 | <0.001 | Paired Wilcoxon signed-rank test |
| JAMA | ChatGPT vs DeepSeek | 52 | 0.97 | 0.91-1.02 | 1.00 | 1.00-1.00 | 1.000 | 1.000-1.000 | <0.001 | Paired Wilcoxon signed-rank test |
| JAMA | ChatGPT vs Doubao | 52 | 1.01 | 0.98-1.05 | 1.00 | 1.00-1.00 | 1.000 | 1.000-1.000 | <0.001 | Paired Wilcoxon signed-rank test |
| JAMA | ChatGPT vs Qwen | 52 | 0.99 | 0.95-1.03 | 1.00 | 1.00-1.00 | 1.000 | 1.000-1.000 | <0.001 | Paired Wilcoxon signed-rank test |
| JAMA | Gemini vs Copilot | 52 | 0.40 | 0.26-0.54 | 0.00 | 0.00-0.80 | 0.875 | 0.665-0.979 | <0.001 | Paired Wilcoxon signed-rank test |
| JAMA | Gemini vs DeepSeek | 52 | 0.98 | 0.92-1.04 | 1.00 | 1.00-1.00 | 1.000 | 1.000-1.000 | <0.001 | Paired Wilcoxon signed-rank test |
| JAMA | Gemini vs Doubao | 52 | 1.02 | 0.98-1.06 | 1.00 | 1.00-1.00 | 1.000 | 1.000-1.000 | <0.001 | Paired Wilcoxon signed-rank test |
| JAMA | Gemini vs Qwen | 52 | 1.00 | 0.96-1.04 | 1.00 | 1.00-1.00 | 1.000 | 1.000-1.000 | <0.001 | Paired Wilcoxon signed-rank test |
| JAMA | Copilot vs DeepSeek | 52 | 0.58 | 0.44-0.72 | 0.80 | 0.20-1.00 | 0.951 | 0.860-0.994 | <0.001 | Paired Wilcoxon signed-rank test |
| JAMA | Copilot vs Doubao | 52 | 0.62 | 0.48-0.75 | 0.90 | 0.20-1.00 | 0.992 | 0.962-1.000 | <0.001 | Paired Wilcoxon signed-rank test |
| JAMA | Copilot vs Qwen | 52 | 0.60 | 0.45-0.75 | 1.00 | 0.20-1.00 | 0.929 | 0.818-0.990 | <0.001 | Paired Wilcoxon signed-rank test |
| JAMA | DeepSeek vs Doubao | 52 | 0.04 | -0.01-0.10 | 0.00 | 0.00-0.00 | 0.284 | -0.209-0.718 | 0.314 | Paired Wilcoxon signed-rank test |
| JAMA | DeepSeek vs Qwen | 52 | 0.02 | -0.04-0.09 | 0.00 | 0.00-0.00 | -0.036 | -0.500-0.374 | 0.880 | Paired Wilcoxon signed-rank test |
| JAMA | Doubao vs Qwen | 52 | -0.02 | -0.06-0.02 | 0.00 | 0.00-0.00 | -0.219 | -0.661-0.231 | 0.420 | Paired Wilcoxon signed-rank test |
| GQS | ChatGPT vs Gemini | 52 | 0.12 | 0.04-0.22 | 0.00 | 0.00-0.00 | 0.672 | 0.284-0.934 | 0.012 | Paired Wilcoxon signed-rank test |
| GQS | ChatGPT vs Copilot | 52 | 0.98 | 0.95-1.02 | 1.00 | 1.00-1.00 | 1.000 | 1.000-1.000 | <0.001 | Paired Wilcoxon signed-rank test |
| GQS | ChatGPT vs DeepSeek | 52 | 1.03 | 0.96-1.12 | 1.00 | 1.00-1.00 | 1.000 | 1.000-1.000 | <0.001 | Paired Wilcoxon signed-rank test |
| GQS | ChatGPT vs Doubao | 52 | 0.99 | 0.96-1.02 | 1.00 | 1.00-1.00 | 1.000 | 1.000-1.000 | <0.001 | Paired Wilcoxon signed-rank test |
| GQS | ChatGPT vs Qwen | 52 | 1.21 | 1.10-1.33 | 1.00 | 1.00-1.00 | 1.000 | 1.000-1.000 | <0.001 | Paired Wilcoxon signed-rank test |
| GQS | Gemini vs Copilot | 52 | 0.86 | 0.76-0.95 | 1.00 | 1.00-1.00 | 0.991 | 0.959-1.000 | <0.001 | Paired Wilcoxon signed-rank test |
| GQS | Gemini vs DeepSeek | 52 | 0.91 | 0.80-1.00 | 1.00 | 1.00-1.00 | 0.988 | 0.953-1.000 | <0.001 | Paired Wilcoxon signed-rank test |
| GQS | Gemini vs Doubao | 52 | 0.87 | 0.77-0.95 | 1.00 | 1.00-1.00 | 0.995 | 0.975-1.000 | <0.001 | Paired Wilcoxon signed-rank test |
| GQS | Gemini vs Qwen | 52 | 1.09 | 0.98-1.20 | 1.00 | 1.00-1.00 | 0.999 | 0.991-1.000 | <0.001 | Paired Wilcoxon signed-rank test |
| GQS | Copilot vs DeepSeek | 52 | 0.05 | -0.03-0.14 | 0.00 | 0.00-0.00 | 0.111 | -0.333-0.511 | 0.710 | Paired Wilcoxon signed-rank test |
| GQS | Copilot vs Doubao | 52 | 0.00 | -0.04-0.05 | 0.00 | 0.00-0.00 | 0.079 | -0.345-0.510 | 0.786 | Paired Wilcoxon signed-rank test |
| GQS | Copilot vs Qwen | 52 | 0.23 | 0.12-0.35 | 0.00 | 0.00-0.20 | 0.724 | 0.435-0.920 | 0.001 | Paired Wilcoxon signed-rank test |
| GQS | DeepSeek vs Doubao | 52 | -0.04 | -0.13-0.03 | 0.00 | 0.00-0.00 | -0.062 | -0.529-0.452 | 0.815 | Paired Wilcoxon signed-rank test |
| GQS | DeepSeek vs Qwen | 52 | 0.18 | 0.07-0.30 | 0.00 | 0.00-0.10 | 0.610 | 0.258-0.872 | 0.008 | Paired Wilcoxon signed-rank test |
| GQS | Doubao vs Qwen | 52 | 0.22 | 0.11-0.35 | 0.00 | 0.00-0.00 | 0.723 | 0.372-0.935 | 0.004 | Paired Wilcoxon signed-rank test |
| ARI | ChatGPT vs Gemini | 52 | -0.27 | -1.38-0.95 | -0.76 | -1.91-0.71 | -0.241 | -0.543-0.078 | 0.198 | Paired Wilcoxon signed-rank test |
| ARI | ChatGPT vs Copilot | 52 | -0.76 | -1.80-0.44 | -1.68 | -2.52-0.07 | -0.343 | -0.614--0.035 | 0.080 | Paired Wilcoxon signed-rank test |
| ARI | ChatGPT vs DeepSeek | 52 | -1.18 | -2.57-0.21 | -1.37 | -2.97-0.44 | -0.263 | -0.556-0.044 | 0.167 | Paired Wilcoxon signed-rank test |
| ARI | ChatGPT vs Doubao | 52 | -8.69 | -12.78--4.89 | -2.84 | -6.98--0.98 | -0.566 | -0.781--0.296 | 0.002 | Paired Wilcoxon signed-rank test |
| ARI | ChatGPT vs Qwen | 52 | 0.01 | -1.04-1.21 | 0.34 | -0.78-1.07 | -0.003 | -0.316-0.309 | 0.989 | Paired Wilcoxon signed-rank test |
| ARI | Gemini vs Copilot | 52 | -0.49 | -1.22-0.25 | -0.47 | -1.31-0.41 | -0.193 | -0.492-0.122 | 0.284 | Paired Wilcoxon signed-rank test |
| ARI | Gemini vs DeepSeek | 52 | -0.91 | -2.09-0.29 | -0.90 | -1.90-0.84 | -0.200 | -0.480-0.118 | 0.284 | Paired Wilcoxon signed-rank test |
| ARI | Gemini vs Doubao | 52 | -8.41 | -12.29--5.02 | -2.93 | -6.77-0.42 | -0.563 | -0.771--0.299 | 0.002 | Paired Wilcoxon signed-rank test |
| ARI | Gemini vs Qwen | 52 | 0.29 | -0.61-1.15 | 0.88 | -0.59-1.67 | 0.132 | -0.177-0.440 | 0.473 | Paired Wilcoxon signed-rank test |
| ARI | Copilot vs DeepSeek | 52 | -0.42 | -1.56-0.68 | -0.00 | -1.61-1.66 | -0.046 | -0.348-0.283 | 0.830 | Paired Wilcoxon signed-rank test |
| ARI | Copilot vs Doubao | 52 | -7.93 | -12.16--4.29 | -2.40 | -4.75--0.04 | -0.530 | -0.746--0.254 | 0.003 | Paired Wilcoxon signed-rank test |
| ARI | Copilot vs Qwen | 52 | 0.78 | -0.00-1.58 | 0.64 | -0.34-1.58 | 0.318 | 0.014-0.591 | 0.087 | Paired Wilcoxon signed-rank test |
| ARI | DeepSeek vs Doubao | 52 | -7.51 | -11.28--4.10 | -3.42 | -7.65--0.83 | -0.604 | -0.820--0.351 | 0.002 | Paired Wilcoxon signed-rank test |
| ARI | DeepSeek vs Qwen | 52 | 1.20 | 0.12-2.24 | 0.80 | -0.25-2.60 | 0.318 | 0.012-0.587 | 0.087 | Paired Wilcoxon signed-rank test |
| ARI | Doubao vs Qwen | 52 | 8.70 | 5.38-12.60 | 2.54 | 0.77-5.83 | 0.681 | 0.454-0.856 | <0.001 | Paired Wilcoxon signed-rank test |
| CL | ChatGPT vs Gemini | 52 | -1.03 | -1.60--0.48 | -0.78 | -1.13--0.46 | -0.549 | -0.782--0.279 | <0.001 | Paired Wilcoxon signed-rank test |
| CL | ChatGPT vs Copilot | 52 | -2.19 | -2.66--1.71 | -2.41 | -2.60--2.00 | -0.884 | -0.996--0.701 | <0.001 | Paired Wilcoxon signed-rank test |
| CL | ChatGPT vs DeepSeek | 52 | -0.24 | -0.84-0.39 | -0.45 | -0.90-0.77 | -0.120 | -0.430-0.200 | 0.522 | Paired Wilcoxon signed-rank test |
| CL | ChatGPT vs Doubao | 52 | -2.37 | -3.00--1.73 | -1.78 | -2.63--1.34 | -0.875 | -0.978--0.724 | <0.001 | Paired Wilcoxon signed-rank test |
| CL | ChatGPT vs Qwen | 52 | -2.55 | -3.14--1.94 | -2.14 | -3.11--1.87 | -0.878 | -0.986--0.726 | <0.001 | Paired Wilcoxon signed-rank test |
| CL | Gemini vs Copilot | 52 | -1.16 | -1.54--0.80 | -1.20 | -1.80--0.81 | -0.759 | -0.920--0.541 | <0.001 | Paired Wilcoxon signed-rank test |
| CL | Gemini vs DeepSeek | 52 | 0.79 | 0.23-1.37 | 0.61 | -0.25-1.39 | 0.373 | 0.071-0.636 | 0.027 | Paired Wilcoxon signed-rank test |
| CL | Gemini vs Doubao | 52 | -1.34 | -2.01--0.69 | -1.36 | -1.91--0.76 | -0.584 | -0.807--0.325 | <0.001 | Paired Wilcoxon signed-rank test |
| CL | Gemini vs Qwen | 52 | -1.52 | -2.14--0.89 | -2.09 | -2.58--1.31 | -0.628 | -0.843--0.367 | <0.001 | Paired Wilcoxon signed-rank test |
| CL | Copilot vs DeepSeek | 52 | 1.96 | 1.54-2.38 | 1.83 | 1.43-2.62 | 0.924 | 0.820-0.984 | <0.001 | Paired Wilcoxon signed-rank test |
| CL | Copilot vs Doubao | 52 | -0.18 | -0.77-0.40 | -0.13 | -0.81-0.83 | -0.062 | -0.350-0.254 | 0.699 | Paired Wilcoxon signed-rank test |
| CL | Copilot vs Qwen | 52 | -0.35 | -0.85-0.14 | -0.38 | -1.18-0.14 | -0.260 | -0.549-0.054 | 0.130 | Paired Wilcoxon signed-rank test |
| CL | DeepSeek vs Doubao | 52 | -2.14 | -2.69--1.56 | -2.41 | -3.15--1.22 | -0.842 | -0.954--0.675 | <0.001 | Paired Wilcoxon signed-rank test |
| CL | DeepSeek vs Qwen | 52 | -2.31 | -2.92--1.69 | -2.59 | -3.03--1.76 | -0.835 | -0.968--0.647 | <0.001 | Paired Wilcoxon signed-rank test |
| CL | Doubao vs Qwen | 52 | -0.18 | -0.81-0.43 | -0.19 | -0.79-0.22 | -0.103 | -0.411-0.208 | 0.558 | Paired Wilcoxon signed-rank test |
| FKGL | ChatGPT vs Gemini | 52 | -0.68 | -1.56-0.35 | -0.91 | -1.89--0.10 | -0.416 | -0.681--0.123 | 0.019 | Paired Wilcoxon signed-rank test |
| FKGL | ChatGPT vs Copilot | 52 | -0.95 | -1.73--0.06 | -1.48 | -1.98--0.41 | -0.485 | -0.732--0.203 | 0.006 | Paired Wilcoxon signed-rank test |
| FKGL | ChatGPT vs DeepSeek | 52 | -1.22 | -2.32--0.09 | -1.49 | -3.00-0.32 | -0.354 | -0.625--0.057 | 0.044 | Paired Wilcoxon signed-rank test |
| FKGL | ChatGPT vs Doubao | 52 | -7.01 | -10.25--3.94 | -2.60 | -5.73--0.50 | -0.602 | -0.804--0.347 | 0.003 | Paired Wilcoxon signed-rank test |
| FKGL | ChatGPT vs Qwen | 52 | -0.69 | -1.53-0.24 | -0.80 | -1.43-0.19 | -0.361 | -0.636--0.045 | 0.044 | Paired Wilcoxon signed-rank test |
| FKGL | Gemini vs Copilot | 52 | -0.26 | -0.84-0.36 | -0.22 | -0.67-0.46 | -0.120 | -0.422-0.189 | 0.522 | Paired Wilcoxon signed-rank test |
| FKGL | Gemini vs DeepSeek | 52 | -0.54 | -1.48-0.47 | -0.34 | -1.38-0.75 | -0.148 | -0.451-0.160 | 0.456 | Paired Wilcoxon signed-rank test |
| FKGL | Gemini vs Doubao | 52 | -6.33 | -9.55--3.40 | -2.32 | -5.24-0.92 | -0.527 | -0.748--0.244 | 0.005 | Paired Wilcoxon signed-rank test |
| FKGL | Gemini vs Qwen | 52 | -0.01 | -0.76-0.73 | 0.30 | -0.41-1.16 | -0.002 | -0.312-0.314 | 0.996 | Paired Wilcoxon signed-rank test |
| FKGL | Copilot vs DeepSeek | 52 | -0.27 | -1.17-0.56 | 0.01 | -1.27-1.59 | -0.009 | -0.324-0.312 | 0.996 | Paired Wilcoxon signed-rank test |
| FKGL | Copilot vs Doubao | 52 | -6.07 | -9.36--3.17 | -1.65 | -3.52--0.25 | -0.492 | -0.724--0.206 | 0.006 | Paired Wilcoxon signed-rank test |
| FKGL | Copilot vs Qwen | 52 | 0.25 | -0.39-0.89 | 0.20 | -0.37-0.82 | 0.145 | -0.170-0.440 | 0.456 | Paired Wilcoxon signed-rank test |
| FKGL | DeepSeek vs Doubao | 52 | -5.79 | -8.89--3.07 | -2.75 | -5.70--0.61 | -0.568 | -0.782--0.308 | 0.003 | Paired Wilcoxon signed-rank test |
| FKGL | DeepSeek vs Qwen | 52 | 0.53 | -0.28-1.39 | 0.39 | -0.52-1.44 | 0.160 | -0.151-0.456 | 0.456 | Paired Wilcoxon signed-rank test |
| FKGL | Doubao vs Qwen | 52 | 6.32 | 3.51-9.43 | 0.93 | 0.02-4.03 | 0.512 | 0.232-0.739 | 0.005 | Paired Wilcoxon signed-rank test |
| GFI | ChatGPT vs Gemini | 52 | -0.69 | -1.52-0.34 | -1.02 | -1.47--0.45 | -0.418 | -0.689--0.125 | 0.016 | Paired Wilcoxon signed-rank test |
| GFI | ChatGPT vs Copilot | 52 | -1.23 | -2.03--0.36 | -1.50 | -2.24--0.65 | -0.512 | -0.750--0.237 | 0.006 | Paired Wilcoxon signed-rank test |
| GFI | ChatGPT vs DeepSeek | 52 | -1.37 | -2.52--0.20 | -1.29 | -3.22-0.41 | -0.348 | -0.620--0.042 | 0.049 | Paired Wilcoxon signed-rank test |
| GFI | ChatGPT vs Doubao | 52 | -7.31 | -11.05--4.00 | -2.88 | -5.69--0.24 | -0.589 | -0.795--0.334 | 0.003 | Paired Wilcoxon signed-rank test |
| GFI | ChatGPT vs Qwen | 52 | -0.95 | -1.75--0.01 | -1.07 | -2.10--0.14 | -0.464 | -0.708--0.181 | 0.011 | Paired Wilcoxon signed-rank test |
| GFI | Gemini vs Copilot | 52 | -0.54 | -1.26-0.16 | -0.47 | -1.19-0.12 | -0.225 | -0.524-0.081 | 0.239 | Paired Wilcoxon signed-rank test |
| GFI | Gemini vs DeepSeek | 52 | -0.68 | -1.71-0.33 | -0.77 | -1.30-0.78 | -0.180 | -0.482-0.142 | 0.355 | Paired Wilcoxon signed-rank test |
| GFI | Gemini vs Doubao | 52 | -6.63 | -9.87--3.65 | -2.66 | -6.02-1.18 | -0.508 | -0.723--0.229 | 0.006 | Paired Wilcoxon signed-rank test |
| GFI | Gemini vs Qwen | 52 | -0.26 | -1.07-0.52 | -0.36 | -1.18-0.68 | -0.107 | -0.418-0.196 | 0.539 | Paired Wilcoxon signed-rank test |
| GFI | Copilot vs DeepSeek | 52 | -0.14 | -1.10-0.74 | 0.23 | -1.06-1.49 | 0.026 | -0.286-0.347 | 0.873 | Paired Wilcoxon signed-rank test |
| GFI | Copilot vs Doubao | 52 | -6.09 | -9.37--2.95 | -1.67 | -4.19--0.19 | -0.450 | -0.698--0.171 | 0.011 | Paired Wilcoxon signed-rank test |
| GFI | Copilot vs Qwen | 52 | 0.28 | -0.47-0.99 | 0.10 | -0.19-0.99 | 0.136 | -0.166-0.438 | 0.473 | Paired Wilcoxon signed-rank test |
| GFI | DeepSeek vs Doubao | 52 | -5.95 | -9.05--3.20 | -2.64 | -5.74--0.82 | -0.557 | -0.775--0.306 | 0.004 | Paired Wilcoxon signed-rank test |
| GFI | DeepSeek vs Qwen | 52 | 0.42 | -0.47-1.30 | 0.33 | -0.64-1.61 | 0.132 | -0.192-0.424 | 0.473 | Paired Wilcoxon signed-rank test |
| GFI | Doubao vs Qwen | 52 | 6.37 | 3.37-9.56 | 0.82 | -0.67-4.86 | 0.446 | 0.145-0.686 | 0.011 | Paired Wilcoxon signed-rank test |
| SMOG | ChatGPT vs Gemini | 52 | -0.69 | -1.21--0.09 | -0.71 | -1.33--0.36 | -0.514 | -0.772--0.229 | 0.004 | Paired Wilcoxon signed-rank test |
| SMOG | ChatGPT vs Copilot | 52 | -1.16 | -1.67--0.61 | -1.19 | -1.72--0.81 | -0.643 | -0.852--0.386 | <0.001 | Paired Wilcoxon signed-rank test |
| SMOG | ChatGPT vs DeepSeek | 52 | -0.92 | -1.61--0.24 | -0.60 | -2.02-0.28 | -0.358 | -0.621--0.043 | 0.041 | Paired Wilcoxon signed-rank test |
| SMOG | ChatGPT vs Doubao | 52 | -4.22 | -6.06--2.47 | -1.82 | -3.82--0.34 | -0.618 | -0.816--0.371 | <0.001 | Paired Wilcoxon signed-rank test |
| SMOG | ChatGPT vs Qwen | 52 | -0.84 | -1.35--0.30 | -0.79 | -1.53--0.40 | -0.547 | -0.779--0.271 | 0.002 | Paired Wilcoxon signed-rank test |
| SMOG | Gemini vs Copilot | 52 | -0.47 | -0.97-0.06 | -0.28 | -1.25-0.07 | -0.296 | -0.588-0.017 | 0.100 | Paired Wilcoxon signed-rank test |
| SMOG | Gemini vs DeepSeek | 52 | -0.23 | -0.91-0.44 | -0.32 | -0.72-0.43 | -0.107 | -0.404-0.205 | 0.581 | Paired Wilcoxon signed-rank test |
| SMOG | Gemini vs Doubao | 52 | -3.53 | -5.37--1.82 | -1.89 | -3.72-0.55 | -0.491 | -0.723--0.192 | 0.005 | Paired Wilcoxon signed-rank test |
| SMOG | Gemini vs Qwen | 52 | -0.15 | -0.72-0.41 | -0.06 | -0.77-0.68 | -0.074 | -0.380-0.229 | 0.692 | Paired Wilcoxon signed-rank test |
| SMOG | Copilot vs DeepSeek | 52 | 0.24 | -0.38-0.79 | 0.44 | -0.36-1.22 | 0.166 | -0.168-0.479 | 0.377 | Paired Wilcoxon signed-rank test |
| SMOG | Copilot vs Doubao | 52 | -3.06 | -4.93--1.40 | -0.97 | -2.42--0.00 | -0.406 | -0.663--0.102 | 0.020 | Paired Wilcoxon signed-rank test |
| SMOG | Copilot vs Qwen | 52 | 0.32 | -0.20-0.82 | 0.24 | -0.14-0.80 | 0.207 | -0.100-0.505 | 0.265 | Paired Wilcoxon signed-rank test |
| SMOG | DeepSeek vs Doubao | 52 | -3.30 | -4.94--1.80 | -1.88 | -3.95--0.50 | -0.559 | -0.778--0.295 | 0.002 | Paired Wilcoxon signed-rank test |
| SMOG | DeepSeek vs Qwen | 52 | 0.08 | -0.53-0.67 | 0.20 | -0.48-0.62 | 0.043 | -0.266-0.369 | 0.788 | Paired Wilcoxon signed-rank test |
| SMOG | Doubao vs Qwen | 52 | 3.38 | 1.86-5.06 | 0.63 | -0.64-3.01 | 0.435 | 0.136-0.684 | 0.014 | Paired Wilcoxon signed-rank test |
| FRES | ChatGPT vs Gemini | 52 | 6.98 | 3.37-10.74 | 6.83 | 3.94-9.02 | 0.640 | 0.385-0.855 | <0.001 | Paired Wilcoxon signed-rank test |
| FRES | ChatGPT vs Copilot | 52 | 10.63 | 7.80-13.38 | 10.76 | 8.23-13.39 | 0.845 | 0.646-0.987 | <0.001 | Paired Wilcoxon signed-rank test |
| FRES | ChatGPT vs DeepSeek | 52 | 5.07 | 1.11-8.84 | 6.38 | -1.53-10.81 | 0.380 | 0.087-0.637 | 0.024 | Paired Wilcoxon signed-rank test |
| FRES | ChatGPT vs Doubao | 52 | 25.54 | 16.13-35.48 | 14.84 | 6.00-24.80 | 0.750 | 0.543-0.907 | <0.001 | Paired Wilcoxon signed-rank test |
| FRES | ChatGPT vs Qwen | 52 | 13.10 | 9.76-16.45 | 12.18 | 8.51-17.24 | 0.851 | 0.685-0.970 | <0.001 | Paired Wilcoxon signed-rank test |
| FRES | Gemini vs Copilot | 52 | 3.65 | 0.96-6.27 | 4.54 | 1.82-7.25 | 0.419 | 0.128-0.681 | 0.013 | Paired Wilcoxon signed-rank test |
| FRES | Gemini vs DeepSeek | 52 | -1.92 | -5.60-2.00 | -0.75 | -7.60-2.68 | -0.139 | -0.441-0.173 | 0.385 | Paired Wilcoxon signed-rank test |
| FRES | Gemini vs Doubao | 52 | 18.55 | 9.94-28.16 | 8.92 | 0.37-20.43 | 0.511 | 0.236-0.732 | 0.003 | Paired Wilcoxon signed-rank test |
| FRES | Gemini vs Qwen | 52 | 6.11 | 2.09-9.76 | 7.04 | 3.98-9.80 | 0.472 | 0.192-0.739 | 0.005 | Paired Wilcoxon signed-rank test |
| FRES | Copilot vs DeepSeek | 52 | -5.56 | -8.18--2.77 | -6.95 | -9.54--4.68 | -0.579 | -0.804--0.308 | <0.001 | Paired Wilcoxon signed-rank test |
| FRES | Copilot vs Doubao | 52 | 14.90 | 6.05-24.57 | 2.39 | 0.01-11.70 | 0.369 | 0.064-0.630 | 0.026 | Paired Wilcoxon signed-rank test |
| FRES | Copilot vs Qwen | 52 | 2.47 | -0.48-5.47 | 2.27 | -1.12-6.16 | 0.247 | -0.061-0.533 | 0.132 | Paired Wilcoxon signed-rank test |
| FRES | DeepSeek vs Doubao | 52 | 20.47 | 12.21-29.54 | 13.97 | 5.25-21.20 | 0.684 | 0.462-0.868 | <0.001 | Paired Wilcoxon signed-rank test |
| FRES | DeepSeek vs Qwen | 52 | 8.03 | 4.50-11.42 | 8.01 | 2.64-12.37 | 0.630 | 0.386-0.832 | <0.001 | Paired Wilcoxon signed-rank test |
| FRES | Doubao vs Qwen | 52 | -12.44 | -21.33--4.20 | -0.44 | -12.68-5.47 | -0.261 | -0.543-0.067 | 0.118 | Paired Wilcoxon signed-rank test |

Note: Pairwise comparisons were performed using paired Wilcoxon signed-rank tests with Benjamini-Hochberg correction. Paired mean/median differences and rank-biserial correlations are reported with bootstrap 95% CIs.
